# Cascade Grignard Addition: Propargyl Claisen Rearrangement for the Stereoselective Synthesis of α‑Allene Quaternary Centers in Cyclohexanones

**DOI:** 10.1021/acs.orglett.5c02006

**Published:** 2025-06-18

**Authors:** Estefania Armendariz-Gonzalez, Adi Saputra, Edward W. Mureka, Cale M. Locicero, Gabrielle L. Womble, Gloria Tan, Abigail A. Watson, Frank R. Fronczek, Rendy Kartika

**Affiliations:** 232 Choppin Hall, Department of Chemistry, 5779 Louisiana State University, Baton Rouge, Louisiana 70803, United States

## Abstract

We report a new method for the enantio- and diastereoselective synthesis of α-allene quaternary centers in fully substituted cyclohexanones at the α-positions. This reaction involves asymmetric 1,2-carbonyl addition to 2-*O*-propargyl enones using a mixture of Grignard reagents and the PMP-H8-BINOL ligand. The resulting magnesium alkoxide chelate intermediate then activated the propargyl vinyl ether moiety, thereby triggering a cascade propargyl Claisen rearrangement in a diastereoselective manner. The synthetic applications of this method in the context of complex molecules are also demonstrated.

## Graphical abstract

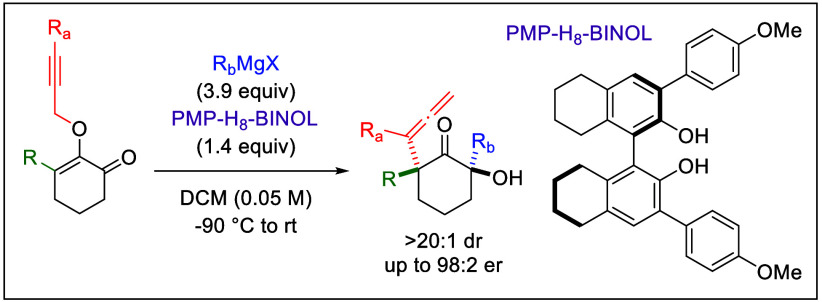

The Claisen rearrangement is a powerful method for the creation of carbon–carbon bonds at the α-position of carbonyl compounds.^1,2^ Proceeding through [3,3]-sigmatropic rearrangement of allyl vinyl ethers, this reaction has been proven invaluable for constructing carbon quaternary centers,^3^ which is challenging in organic synthesis.^4^ A notable variant of the Claisen rearrangement involves the conversion of propargyl vinyl ethers to yield an α-allene functionality.^5^ Allenes are characterized by two perpendicular double bonds that are linked to a central sp-hybridized carbon atom.^6^ This distinct structural feature provides allenes with unusual chemical reactivity that can be harnessed in various synthetic transformations.^7^ Nonetheless, the propargyl Claisen rearrangement presents unique challenges. Specifically, the transition state of this pericyclic reaction must navigate the geometric constraints imposed by the linear arrangements of the sp-hybridized carbons in the propargyl group.

The significance of propargyl Claisen rearrangement is evident in the stereoselective construction of α-allene quaternary centers, although such examples are rare (Scheme 1).^8^ For instance, Feng reported asymmetric propargyl Claisen rearrangement of *O*-propargyl β-ketoesters **1a** using Ni­(II)–ligand complex **2a**, which formed product **1b** in high enantioselectivity.^8a^ In a related approach, Xie and Guo employed Co­(II)–ligand **2b** as a catalyst for this transformation.^8b^ Within the context of diastereoselective propargyl Claisen rearrangement, advancements have been primarily associated with the total synthesis of natural products. In these cases, stereochemical information from conformationally restricted propargyl vinyl ether substrates is strategically deployed to direct the α-allene quaternary stereochemical outcome.^9^ For example, the propargyl Claisen rearrangement was a critical step in Ley’s total synthesis of azadirachtin, wherein complex substrate **3** was transformed to product **4** at 180 °C under microwave irradiation.^9a^

**Scheme 1:**
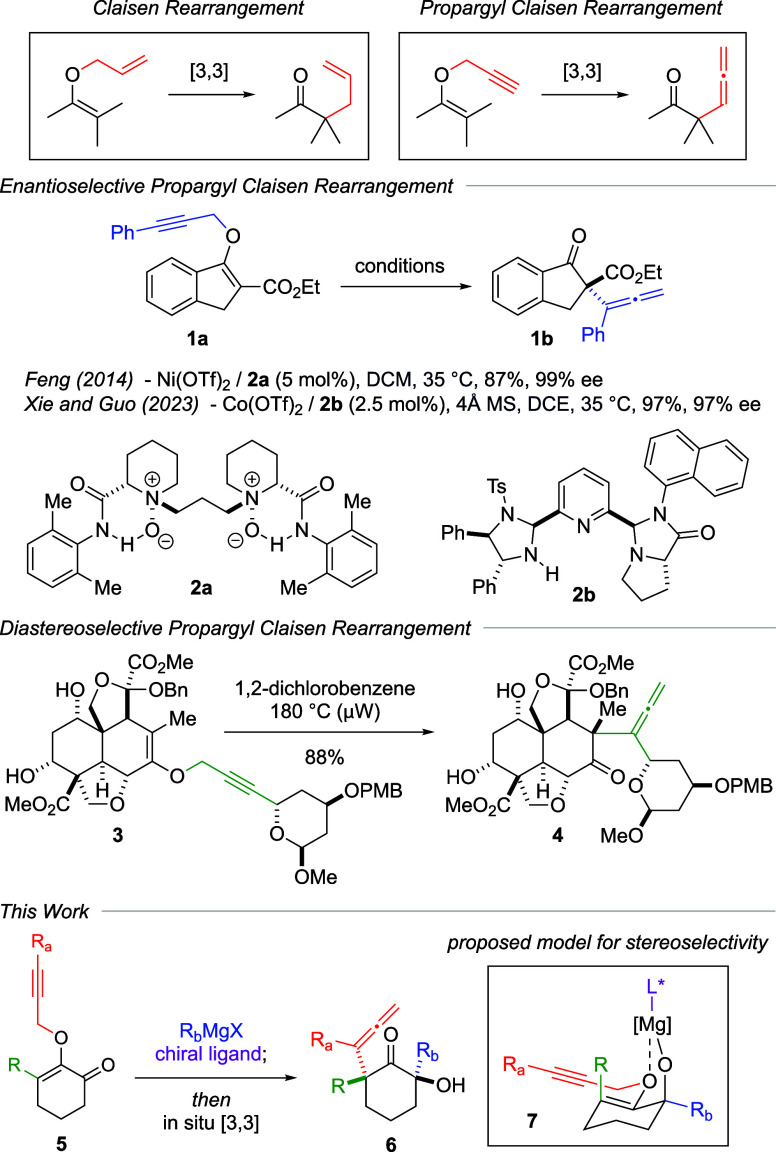
Synthesis of α-Allene Quaternary Centers via Propargyl Claisen Rearrangement

The limited examples of diastereoselective synthesis of α-allene quaternary centers via propargyl Claisen rearrangement prompted us to explore a new strategy with a focus on using simple monocyclic achiral propargyl vinyl ethers, specifically 2-*O*-propargyl enone **5**, as starting materials. We proposed that treatment of substrate **5** with a Grignard reagent would produce a magnesium-chelate intermediate **7** as a product of the 1,2-carbonyl addition reaction. This chelation would then activate the [3,3]-sigmatropic rearrangement, thereby forming the α-allene quaternary centers while also guiding diastereoselectivity. Scheme 2 presents our initial findings. To test our hypothesis, we introduced methylmagnesium bromide to a precooled solution of 2-*O*-propargyl enone **5a** in three different solvents, i.e., THF, Et_2_O, and DCM. Once the substrate was fully consumed, the reaction mixture was warmed to room temperature, which triggered the propargyl Claisen rearrangement to furnish α-allene ketone **6a**. Interestingly, DCM was found to be effective in generating the product in 82% yield as a single diastereomer. We then explored the temperature effects (0 and −78 °C) for the Grignard addition. Although the product yields were comparable, the lower temperature afforded cleaner crude reaction mixtures. Remarkably, the use of a slight excess of the Grignard reagent (1.3 equiv), which necessitated full consumption of substrate **5**, did not lead to overaddition to the emerging ketone functionality in **6a**.

**Scheme 2:**
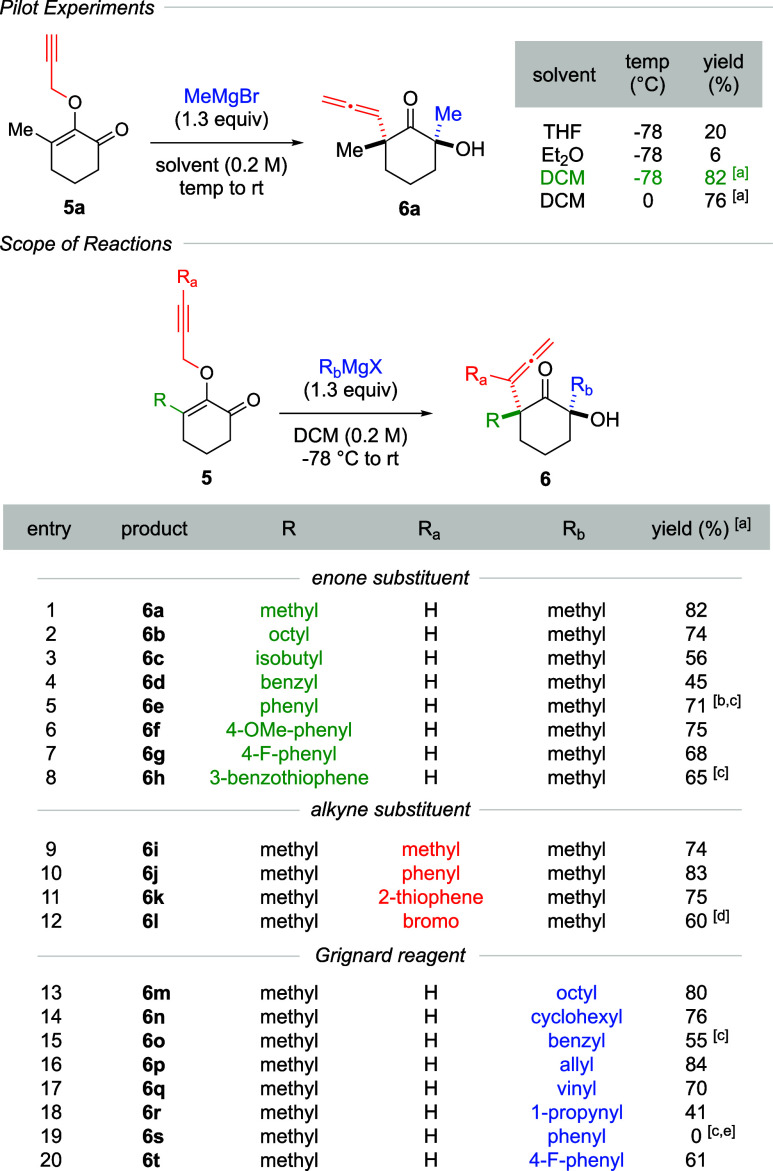
Racemic Synthesis

a Products were formed with >20:1 diastereomeric ratio based on ^1^H NMR analysis of the crude reaction mixtures.
b The Grignard addition was conducted at 0 °C.
c The propargyl Claisen rearrangement was performed at reflux.
d The reaction was performed with 1.1 equiv of Grignard reagent.
e The reaction yielded the 1,2-carbonyl addition product.

This simple procedure was found to be applicable across a broad scope of reactions. Our survey began with varying the R substituent in the enone moiety. When treated with methylmagnesium bromide, substrates containing long-chain octyl, branched isobutyl, and benzyl groups formed products **6b** to **6d** in moderate to good yields (entries 2–4). We then proceeded with aromatic substituents, including phenyl, 4-OMe-phenyl, 4-F-phenyl, and 3-benzothiophene (entries 5–8). In these cases, the resulting α-allene ketones **6e**–**6h** were isolated in 65–75% yields. Notably, certain aromatic rings seemed to affect the propargyl Claisen rearrangement, requiring warming the reaction mixtures to reflux. Continuing with the alkyne substituents, methyl, phenyl, 2-thiophene, and bromo groups were introduced at the R_a_ position, which decorated the α-allene group. As indicated in entries 9–12, ketones **6i**–**6l** were isolated with 60–83% yields. Lastly, various Grignard reagents were also examined (entries 13–20). Organomagnesium reagents bearing octyl, cyclohexyl, benzyl, and allyl groups were tolerated, furnishing α-allene ketones **6m**–**6p** in high yields. For benzyl-containing product **6o**, the propargyl Claisen rearrangement required reflux conditions. We noted differing reactivities between sp^2^ and sp hybridized nucleophiles. While vinylmagnesium bromide resulted in product **6q** in 70% yield, the 1-propynyl counterpart formed **6r** with a lower yield. An unexpected outcome was noted when comparing phenyl and 4-F-phenyl. While 4-F-phenylmagnesium bromide formed α-allene ketone **6t** in 61% yield, the phenyl variant only yielded the 1,2-carbonyl addition product. In fact, the propargyl Claisen rearrangement did not occur.

The enantioselective version of our method could be achieved by regulating the facial selectivity of the Grignard addition to the 2-*O*-propargyl enone substrates.^10^ In this pursuit, we took inspiration from Nakajima, who reported asymmetric 1,2-carbonyl addition to ketones by combining substituted BINOL ligands with a 3-fold excess of Grignard reagents.^10a^ For our studies, we opted to evaluate the H_8_–BINOL variant to simplify the synthesis of ligand libraries, thereby accelerating the optimization process. Results of our reaction optimization are summarized in Table 1. The pilot experiment involved the reaction of methylmagnesium bromide (3.9 equiv) to substrate **5a** in the presence of 9-anthracyl-H_8_-BINOL ligand **7a** (1.4 equiv) at −78 °C. Once the starting material was consumed, the mixtures were allowed to warm to room temperature to initiate the propargyl Claisen rearrangement. We conducted these reactions in two noncoordinating solvents, i.e., toluene and DCM (entries 1 and 2), and found that the use of DCM led to stronger enantioinduction (81:19 er) compared to toluene (64:36 er). Equally significant, the resulting product (+)-**6a** was formed as a single diastereomer. This promising outcome prompted us to screen various substituents in the H_8_–BINOL ligands. Other bulky groups, such as 9-phenanthryl **7b** and 1-pyrenyl **7c**, only marginally improved enantioselectivity (entries 3 and 4). Interestingly, similar trends were noted with smaller substituents in ligands **7d**–**7f** (entries 5–7), with the most intriguing result arising from a simple phenyl group in ligand **7f**, which afforded the α-allene ketone with 86:14 er.

**Table 1:**
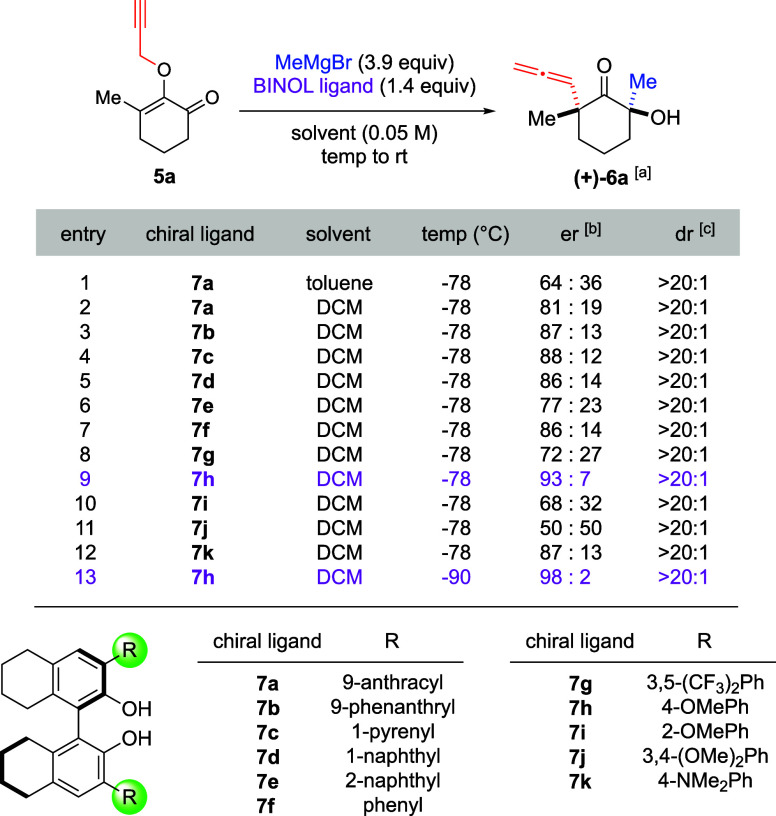
Reaction Optimization for Enantioselective Synthesis

a Yields were not quantified due to volatility of the products.

b Enantiomeric ratio was measured by chiral HPLC of the *p*-nitrobenzoate ester derivative of the products. See Supporting Information.

c Products were formed with >20:1 diastereomeric ratio based on ^1^H NMR analysis of the crude reaction mixtures.

The surprising efficacy of the phenyl group prompted an evaluation of its stereoelectronic effects. As shown in entries 8 and 9, the inclusion of 3,5-(CF_3_)_2_Ph groups in ligand **7g** resulted in an erosion of enantioselectivity. In contrast, 4-OMePh in ligand **7h** improved er to 93:7. The positioning of the methoxy group within the phenyl ring is crucial. For example, 2-OMePh in ligand **7i** (entry 10) dropped enantioselectivity to 68:32. Efforts to enhance enantioinduction by incorporating two methoxy groups at the 3,4-positions in ligand **7j** proved ineffective, as the resulting α-allene ketone was formed as a racemic mixture (entry 11). We also examined the 4-NMe_2_Ph variant, which yielded enantioselectivity comparable to that of the parent ligand **7f** (entry 12). Overall, our ligand screening identified PMP-H_8_-BINOL **7h** as the most effective ligand. The enantioselectivity was further enhanced by lowering the reaction temperature to −90 °C. Under these conditions, the α-allene quaternary center in ketone (+)-**6a** was produced with 98:2 er (entry 13).

Using these optimized conditions, we surveyed the scope of reaction by first assessing effects of the R substituent on the enone moiety with methylmagnesium bromide as the nucleophile (Scheme 3). Substrates featuring octyl and isobutyl groups afforded α-allene ketone (+)-**6b** and (+)-**6c** in 98% and 82% yields, respectively, with comparable 92:8 and 93:7 er. Similarly, the benzyl variant yielded product (+)-**6d** in 77% yield with a 94:6 er. Next, we examined aromatic and heteroaromatic substituents. Except the 4-OMe-phenyl in (−)-**6f**, which was isolated with 72:28 er, the phenyl (+)-**6e**, the 4-F-phenyl (+)-**6g**, and the 3-benzothiophene (+)-**6h** were obtained with good yields and enantioselectivity. Efforts to place substitutions at the R_a_ position of the resulting α-allene functionality with methyl, phenyl, 2-thiophene, and bromo were successful. As depicted in α-allene ketones (−)-**6i** to (+)-**6l**, these products were isolated in 72–93% yields with er ranging between 87:13 and 92:8. As detailed in Supporting Information, the absolute and relative stereochemistry of these enantiomerically enriched products was deduced by analogy using X-ray crystallography of (+)-**6g**, (+)-**6j**, and (+)-**6l**. Similar to the racemic synthesis, the propargyl Claisen rearrangement for some substrates required reflux conditions to proceed.

**Scheme 3:**
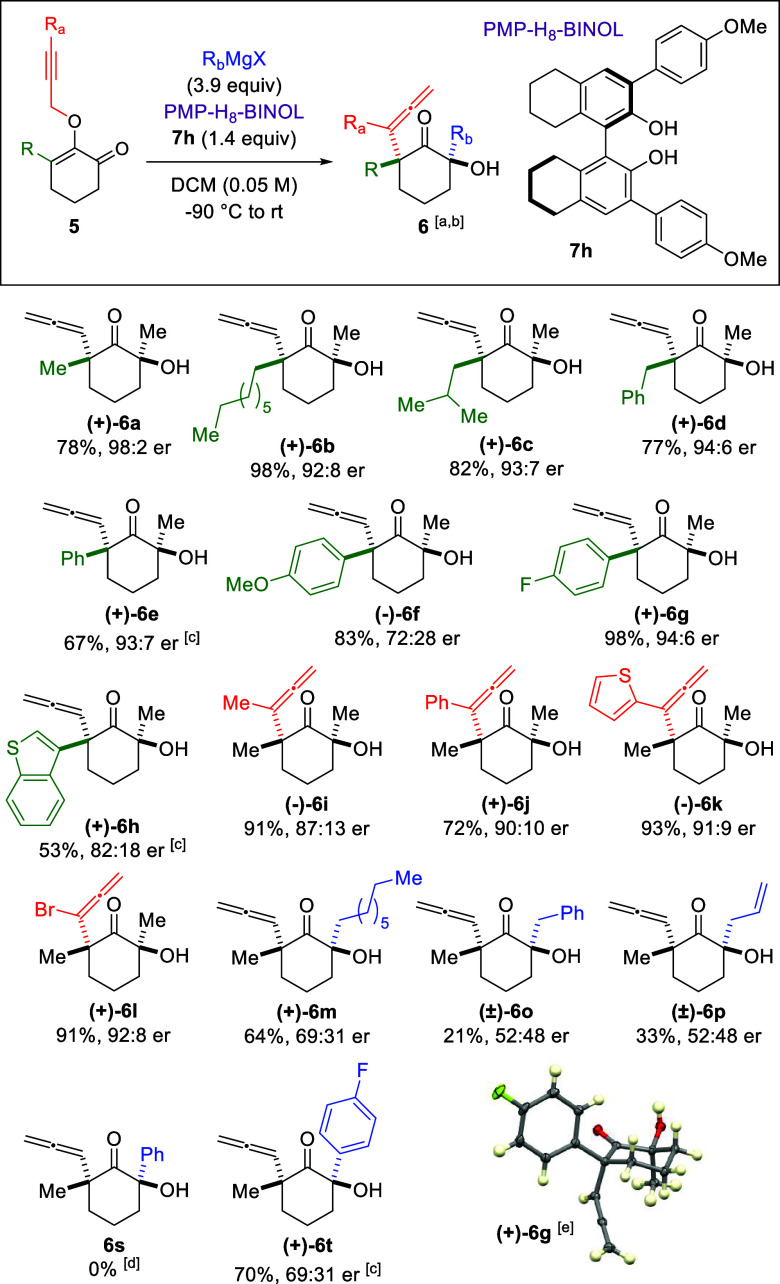
Enantioselective Synthesis

a Enantiomeric ratio was measured by chiral HPLC of the *p*-nitrobenzoate ester derivative of the products. See Supporting Information.
b Products were formed with >20:1 diastereomeric ratio based on ^1^H NMR analysis of the crude reaction mixtures.
c The propargyl Claisen rearrangement was performed at reflux.
d The reaction only yielded the 1,2-carbonyl addition product.
e The ellipsoid contour was set at a 50% probability level.

While the asymmetric conditions proved effective with methylmagnesium bromide, efforts to employ larger nucleophiles led to a loss in enantioinduction. For instance, octylmagnesium bromide yielded product (+)-**6m** with 69:31 er. Surprisingly, the benzyl and allyl counterparts formed α-allene ketones (±)-**6o** and (±)-**6p** as a racemic mixture in low yields.^12^ We also assessed aromatic nucleophiles, such as phenyl and 4-F-phenyl. While phenylmagnesium bromide did not lead to the propargyl Claisen rearrangement, which is consistent with the racemic synthesis (Scheme 2, entry 19), the 4-F-phenyl adduct (+)-**6t** was isolated in 70% yield with 69:31 er.

Scheme 4 showcases additional synthetic studies that feature our method. The utility of this magnesium-chelate activation of propargyl Claisen rearrangement within the context of complex molecules was exemplified through the functionalization of formestane-derived substrate (+)-**8** with methylmagnesium bromide. As shown in the resulting product (+)-**9a**, which was isolated in 66% yield as a single diastereomer, the reaction of (+)-**8** with 2.6 equiv of the Grignard reagent led to diastereoselective 1,2-carbonyl addition at both C3 and C17 positions, while installing the α-allene group at the C5 carbon. Subjecting the substrate with 1.1 equiv of methylmagnesium bromide remarkably led to methyl addition at the C3 carbonyl, followed by the propargyl Claisen rearrangement to furnish α-allene ketone (−)-**9b** in 40% yield, which was isolated as a single diastereomer. This intriguing chemoselectivity was most likely the result of the chelation effect between the magnesium metal and the two neighboring oxygen atoms at the C3 and C4 positions, which consequently differentiated the rate of the Grignard addition at the C3 carbonyl compared to that of the C17. Next, we carried out scale-up reactions of substrate **5j** on a one-gram quantity using both racemic and enantioselective protocols. As expected, the racemic conditions furnished α-allene ketone **6j** in 83% yield with >20:1 dr. The enantioselective version also proceeded smoothly to produce (+)-**6j** in 93% yield with 94:6 er and >20:1 dr. Significantly, the PMP-H_8_-BINOL ligand **7h** was successfully recovered in pure form with a mass recovery of 91%, thus underscoring the cost-effectiveness and practicality of our method.

**Scheme 4:**
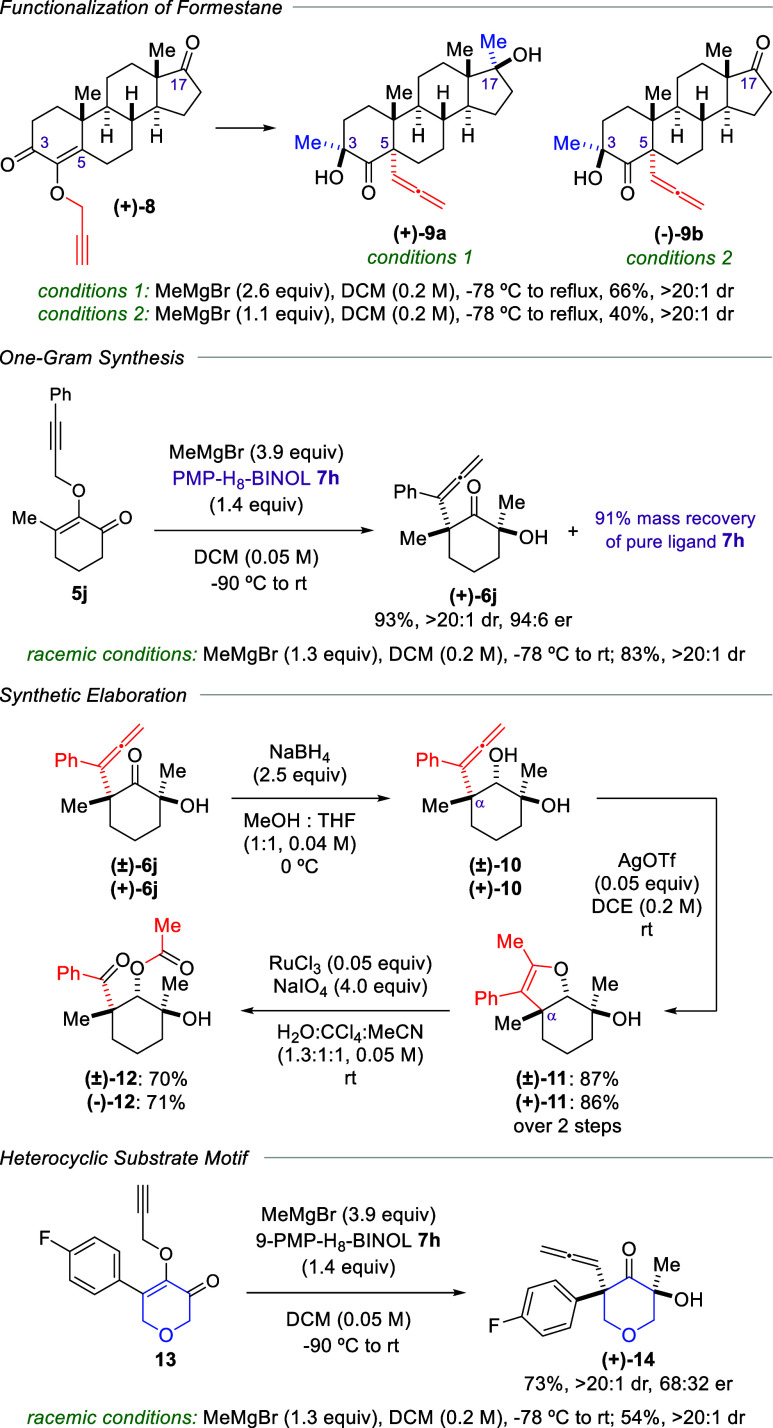
Additional Synthetic Studies

The α-allene ketone functionality in product **6j** was then subjected to further synthetic elaboration to create stereochemically complex derivatives. For example, carbonyl reduction of **6j** with NaBH_4_ produced stereotriad **10**, where the diastereochemical outcome was directed by the hydroxy stereocenter at the opposing α-position. Following this step, intramolecular cyclization catalyzed by AgOTf effectively yielded heterocycle **11**.^7g^ The electron-rich double bond in the resulting ring structure could then be cleaved oxidatively using catalytic RuCl_3_ and NaIO_4_ in wet reaction medium, which afforded highly functionalized product **12**.^13^ Both racemic and enantioenriched substrate (±)-**6j** and (+)-**6j** were subjected to this sequence, generating their respective products in comparably high yields. We also subjected our method to heterocyclic substrate motif **13**, which formed pyranone (+)-**14** as a single diastereomer, albeit with 68:32 er. This enantioinduction is surprisingly lower than that of the cyclohexanone counterpart (+)-**6g**.

In conclusion, we have developed enantio- and diastereoselective synthesis of α-allene quaternary centers in fully substituted cyclohexanones at the α-positions. Our method featured the reaction of simple 2-*O*-propargyl enones with a mixture of Grignard reagents and PMP-H_8_-BINOL ligand, resulting in a cascade sequence of 1,2-carbonyl addition and propargyl Claisen rearrangement. The utility of this chemistry was demonstrated through its synthetic studies in complex-molecule settings.

## Supplementary Material



## Data Availability

The data underlying this study are available in the published article and its Supporting Information.

## Notes

Experimental procedures, characterization data, chiral HPLC chromatograms, X-ray crystallographic data, and ^1^H and ^13^NMR spectra for all new compounds (PDF)
