# Supplementary material for: Cascade Grignard Addition: Propargyl Claisen Rearrangement for the Stereoselective Synthesis of α‑Allene Quaternary Centers in Cyclohexanones
Source: Org Lett. 2025 Jun 18;27(29):7767–72. doi: 10.1021/acs.orglett.5c02006 (PMC12305655; doi:10.1021/acs.orglett.5c02006)
Supplement: Supplementary file 1 [file ol5c02006_si_001.pdf]

Cascade Grignard Addition – Propargyl Claisen Rearrangement for the Stereoselective Synthesis of α-Allene Quaternary Centers in Cyclohexanones

Estefania Armendariz-Gonzalez,‡ Adi Saputra,‡ Edward W. Mureka, Cale M. Locicero, Gabrielle L. Womble, Gloria Tan, Abigail A. Watson, Frank R. Fronczek, and Rendy Kartika*

‡ Equal Contribution

Department of Chemistry

232 Choppin Hall

Louisiana State University

Baton Rouge, LA 70803, USA

email: rkartika@lsu.edu

SUPPORTING INFORMATION

Table of Content

1. General Information……………………………………………………………… SI-3

2. Experimental Data for Schemes 2 and 3 …………………………………………… SI-4

3. Experimental Data for Scheme 4 …………………………………………………… SI-64

4. Synthesis of Substrates …………………………………………………………… SI-78

5. X-Ray Crystallography Data ……………………………………………………… SI-92

6. ^{1}H And ^{13}C NMR Spectra ………………………………………………… SI-168

General Information

Unless otherwise noted, all materials were used as received from commercial suppliers without further purification. All anhydrous reactions were performed using oven-dried glassware, which was then cooled under vacuum and purged with nitrogen gas. Tetrahydrofuran (THF), dichloromethane (CH_2Cl_2), acetonitrile, toluene, diethyl ether (Et_2O), dimethylformamide (DMF), and *n*-hexanes were filtered through activated silica or 3Å molecular sieves under argon contained in a Solvent Purification System. All reactions were monitored by analytical thin layer chromatography (TLC Silica Gel 60 F_{254}, Glass Plates) and analyzed with 254 nm UV light and / or anisaldehyde – sulfuric acid or potassium permanganate treatment. Column chromatography was completed using silica gel (32-63 μ).

Unless otherwise noted, all ^1H and ^{13}C NMR spectra were recorded in $CDCl_3$ using a Bruker Ascend 400 spectrometer operating at 400 MHz for ^1H and 100 MHz for ^{13}C or Bruker Ascend 500 spectrometer operating at 500 MHz for ^1H and 125 MHz for ^{13}C. Chemical shifts (δ) are reported in ppm relative to residual $CHCl_3$ as an internal reference (^1H: 7.26 ppm, ^{13}C: 77.00 ppm). Coupling constants (*J*) are reported in Hertz (Hz). Peak multiplicity is indicated as follows: s (singlet), d (doublet), t (triplet), q (quartet), p (pentet), x (sextet), h (heptet), b (broad), and m (multiplet). FT-IR spectra were recorded on Bruker Tensor 27 spectrometer and OPUS 6.5 Data Collection Program, and absorption frequencies were reported in reciprocal centimeters (cm^{-1}). High Resolution Mass Spectrometry (HRMS) measurements using a time-of-flight (TOF) mass analyzer were performed at the Louisiana State University Mass Spectrometry Facility. X-ray structure analyses were performed by the Louisiana State University X-ray Structure Facility. HPLC analyses were performed using Vanquish Core system.

Experimental Data for Schemes 2 and 3

Compound (+)-6a

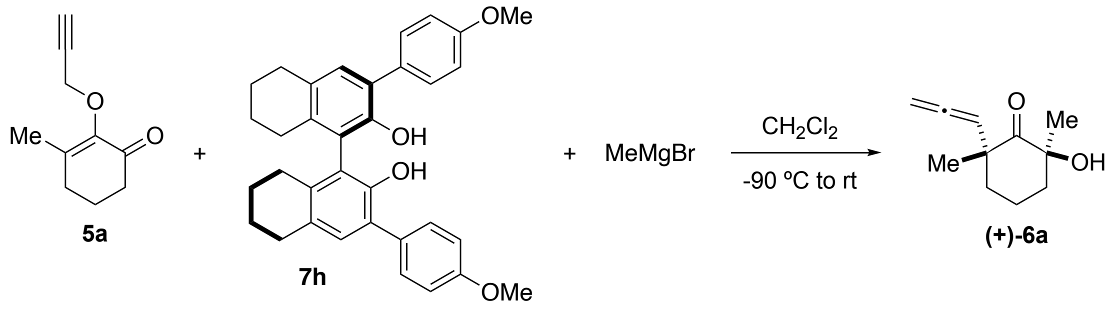

Procedure: Ligand **7h** (447 mg, 0.882 mmol) and ketone **5a** (104 mg, 0.630 mmol) were dissolved in CH_2Cl_2 (13 mL) in a round bottom flask. The solution was cooled to -90 °C, followed by the addition of methylmagnesium bromide (0.82 mL, 2.5 mmol, 3.0 M in Et_2O) dropwise. After stirring at -90 °C until the starting material was fully consumed as monitored by TLC, the reaction mixture was warmed to room temperature and stirred until the Claisen rearrangement of the 1,2-carbonyl addition intermediate was complete as monitored by TLC for a total reaction time of 19 hours. After cooling to 0 °C, the reaction was quenched with a saturated NH_4Cl solution (8 mL) and diluted with DI water (8 mL). The aqueous layer was extracted with CH_2Cl_2 (3 x 10 mL). The combined organic layers were dried over Na_2SO_4 and concentrated under vacuum. The crude material was purified by column chromatography using 100% CH_2Cl_2 for ligand **7h** recovery, followed by 80:20 hexanes : EtOAc to afford **(+)-6a** in 78% yield (89 mg, 0.49 mmol) as a colorless oil. ^1H NMR analysis of the crude reaction mixture indicated > 20:1 dr.

Rf: 0.7 in 80:20 hexanes : EtOAc

^{1}H NMR: (400 MHz, $CDCl_3$) δ = 5.14 (t, *J* = 6.7 Hz, 1H), 4.94 (dd, *J* = 11.1, 6.8 Hz, 1H), 4.87 (dd, *J* = 11.1, 6.6 Hz, 1H), 3.95 (s, 1H), 2.15 – 2.06 (m, 2H), 1.86 (tt, *J* = 12.9, 3.2 Hz, 1H), 1.75 (dd, *J* = 12.9, 3.5 Hz, 1H), 1.71 – 1.58 (m, 2H), 1.44 (s, 3H), 1.23 (s, 3H).

^{13}C NMR: (100 MHz, $CDCl_3$) δ = 215.6, 207.1, 95.4, 78.8, 76.5, 47.6, 41.1, 38.8, 27.9, 26.3, 19.2.

IR: *f* (cm^{-1}) = 3432, 3079, 2935, 2869, 1673, 1490, 1443, 1380, 1147, 758.

HRMS (ESI-TOF): *m/z* $[M+H]^+$ = 181.1223 calculated for $C_{11}H_{17}O_2$; found 181.1228.

Specific Rotation: $[\alpha]_{25}^{D}$ = +1.5 (c = 1.0 in $CHCl_3$)

HPLC ((+)-6a)-BzNO₂: (S,S)-Whelk-O1, hexane/isopropanol = 97.5/2.5, flow rate = 1.0 mL/min, λ = 254 nm, t_R = 11.3 min (major), 14.1 min (minor).

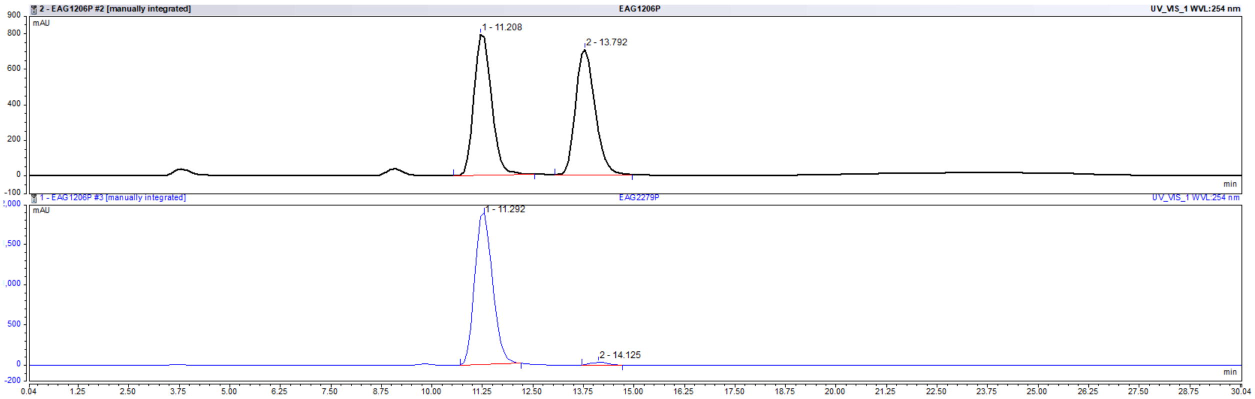

Retention Time (min)	Relative Area (%)	Area (mAU*min)	Height (mAU)
11.292	98.41	960.0803	1878.23
14.125	1.59	15.5596	31.48

Compound ((+)-6a)-BzNO$_2$

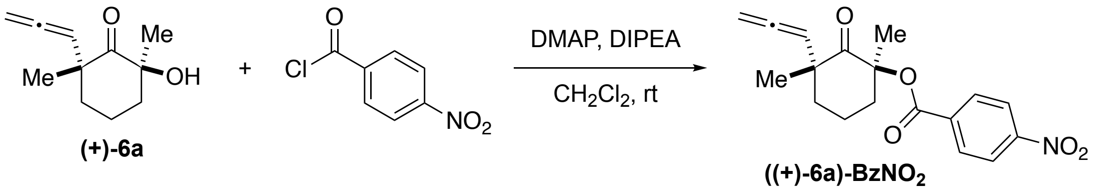

Procedure: Alcohol **(+)-6a** (30 mg, 0.17 mmol) was dissolved in CH_2Cl_2 (0.8 mL). *N*,*N*-Diisopropylethylamine (150 µL, 0.84 mmol) was added to the solution, followed by 4-nitrobenzoyl chloride (100 mg, 0.54 mmol) and 4-(dimethylamino)pyridine (21 mg, 0.17 mmol).

The reaction mixture was then stirred at room temperature until complete consumption of the starting material as monitored by TLC. The crude reaction mixture was concentrated under vacuum and then purified by column chromatography using 94:6 hexanes : EtOAc to afford compound **((+)-6a)-BzNO$_2$** in 95% yield (53 mg, 0.16 mmol) as a colorless oil.

Rf: 0.7 in 80:20 hexanes : EtOAc

^{1}H NMR: (400 MHz, $CDCl_3$) δ = 8.26 (dt, *J* = 9.0, 2.0 Hz, 2H), 8.18 (dt, *J* = 8.9, 1.7 Hz, 2H), 5.12 (t, *J* = 6.7 Hz, 1H), 4.89 (ddd, *J* = 21.0, 11.0, 6.8 Hz, 2H), 2.58 (td, *J* = 12.7, 4.7 Hz, 1H), 2.10 – 1.74 (m, 5H), 1.64 (s, 3H), 1.34 (s, 3H).

^{13}C NMR: (100 MHz, $CDCl_3$) δ = 208.4, 206.9, 163.4, 150.5, 135.7, 130.8, 123.4, 96.6, 83.6, 78.7, 48.9, 37.1, 36.0, 26.4, 25.9, 19.2.

IR: *f* (cm^{-1}) = 3113, 3078, 2964, 2932, 2870, 1709, 1607, 1525, 1348, 719.

HRMS (ESI-TOF): *m/z* $[M+H]^+$ = 330.1336 calculated for $C_{18}H_{20}NO_5$; found 330.1352.

Compound (±)-6a

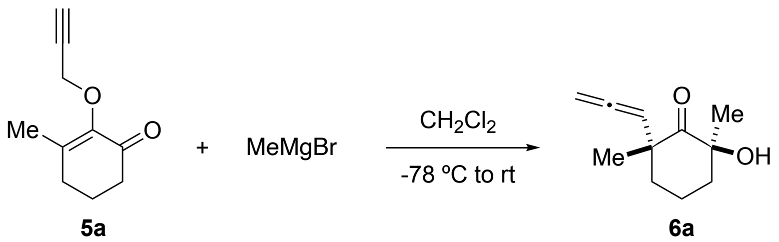

Procedure: Ketone **5a** (108 mg, 0.660 mmol) and CH_2Cl_2 (3.3 mL) were added into a round bottom flask. After cooling the solution to -78 ºC, methylmagnesium bromide (0.29 mL, 0.86 mmol, 3.0 M in Et_2O) was added dropwise. The reaction mixture was allowed to warm to room temperature and stirred for 25 hours, at which the Claisen rearrangement of the 1,2-carbonyl addition intermediate was complete as monitored by TLC. After cooling to 0 °C, the reaction was quenched with saturated NH_4Cl (5 mL), and the resulting mixture was extracted with CH_2Cl_2 (3 x

5 mL). The organic layers were combined, dried over Na_2SO_4, filtered, and then concentrated under vacuum. The crude material was purified with column chromatography using 93:7 hexanes : EtOAc to yield **6a** in 82% yield (98 mg, 0.66 mmol) as a colorless oil. ^{1}H NMR analysis of the crude reaction mixture indicated > 20:1 dr.

^{1}H NMR: (400 MHZ, $CDCl_3$) δ = 5.13 (t, *J* = 5.3 Hz, 1H), 4.93 (dd, *J* = 8.9, 5.4 Hz, 1H), 4.86 (dd, *J* = 8.9, 5.3 Hz, 1H), 2.09 (dp, *J* = 11.1, 2.5 Hz, 2H), 1.86 (qt, *J* = 10.3, 2.2 Hz, 1H), 1.74 (dd, *J* = 10.5, 3.0 Hz, 1H), 1.71 – 1.63 (m, 1H), 1.59 (td, *J* = 10.8, 3.2 Hz, 1H), 1.43 (s, 3H), 1.22 (s, 3H).

^{13}C NMR: (100 MHz, $CDCl_3$) δ = 215.6, 207.0, 95.4, 78.8, 76.4, 47.6, 41.0, 38.7, 27.9, 26.2, 19.2.

Compound (+)-6b

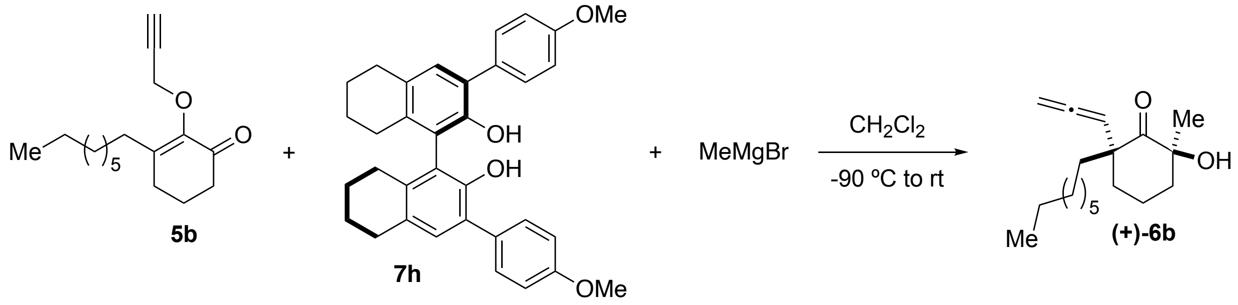

Procedure: Ligand **7h** (177 mg, 0.350 mmol) and ketone **5b** (66 mg, 0.25 mmol) were dissolved in CH_2Cl_2 (5.0 mL) in a round bottom flask. The solution was cooled to -90 °C, followed by the addition of methylmagnesium bromide (0.33 mL, 0.98 mmol, 3.0 M in Et_2O) dropwise. After stirring at -90 °C until the starting material was fully consumed as monitored by TLC, the reaction mixture was warmed to room temperature and stirred until the Claisen rearrangement of the 1,2-carbonyl addition intermediate was complete as monitored by TLC for a total reaction time of 22 hours. After cooling to 0 °C, the reaction was quenched with a saturated NH_4Cl solution (8 mL) and diluted with DI water (8 mL). The aqueous layer was extracted with CH_2Cl_2 (3 x 10 mL), and

the combined organic layers were dried over Na_2SO_4, and concentrated under vacuum. The crude material was purified by column chromatography using 100% CH_2Cl_2 for ligand **7h** recovery followed by 94:6 hexanes : EtOAc to afford **(+)-6b** in 98% yield (68 mg, 0.25 mmol) as a colorless oil. ^{1}H NMR analysis of the crude reaction mixture indicated > 20:1 dr.

Rf: 0.7 in 80:20 hexanes : EtOAc

^{1}H NMR: (400 MHz, $CDCl_3$) δ = 4.99 (td, *J* = 6.9, 1.1 Hz, 1H), 4.89 (qd, *J* = 11.2, 7.0 Hz, 2H), 3.88 (s, 1H), 2.14 – 2.03 (m, 2H), 1.92 – 1.77 (m, 1H), 1.76 – 1.64 (m, 3H), 1.62 – 1.51 (m, 2H), 1.42 (s, 3H), 1.26 (bs, 12H), 0.88 (t, *J* = 6.5 Hz, 3H).

^{13}C NMR: (100 MHz, $CDCl_3$) δ = 215.6, 207.7, 94.3, 78.4, 76.4, 50.8, 40.3, 38.5, 34.9, 31.9, 30.1, 29.5, 29.3, 28.3, 23.6, 22.6, 19.0, 14.1.

IR: *f* (cm^{-1}) = 3498, 2925, 2855, 1700, 1459, 1367, 1156, 1112, 846.

HRMS (ESI-TOF): *m/z* $[M+H]^+$ = 279.2319 calculated for $C_{18}H_{31}O_2$; found 279.2319.

Specific Rotation: $[\alpha]_{25}^{D}$ = +63.8 (c = 1.0 in $CHCl_3$)

HPLC ((+)-6b)-BzNO$_2$: (S,S)-Whelk-O1, hexane/isopropanol = 99.4/0.6, flow rate = 1.0 mL/min, λ = 254 nm, t_R = 14.9 min (major), 18.2 min (minor).

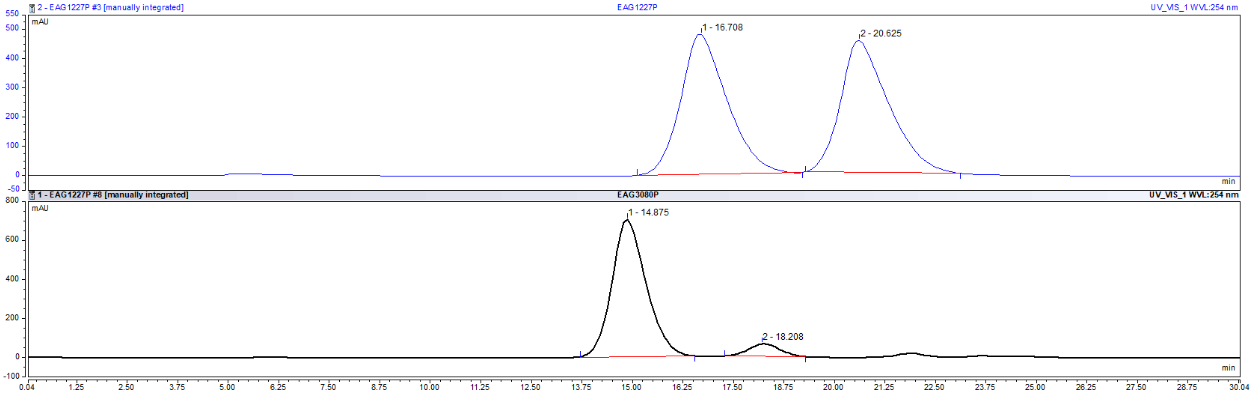

Retention Time (min)	Relative Area (%)	Area (mAU*min)	Height (mAU)
14.875	92.05	691.0858	703.20
18.208	7.95	59.6626	65.41

Compound ((+)-6b)-BzNO₂

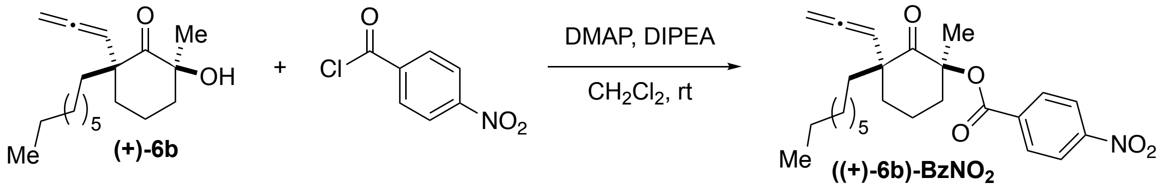

Procedure: Alcohol **(+)-6b** (21 mg, 0.074 mmol) was dissolved in CH_2Cl_2 (0.4 mL). *N*,*N*-Diisopropylethylamine (65 μL, 0.37 mmol) was then added to the solution, followed by 4-nitrobenzoyl chloride (44 mg, 0.24 mmol) and 4-(dimethylamino)pyridine (9.1 mg, 0.074 mmol). The reaction mixture was then stirred at room temperature until complete consumption of the starting material as monitored by TLC. The crude reaction mixture was concentrated under vacuum and then purified by column chromatography using 94:6 hexanes : EtOAc to afford compound **((+)-6b)-BzNO$_2$** in 67% yield (21 mg, 0.050 mmol) as a colorless oil.

Rf: 0.8 in 80:20 hexanes : EtOAc

^{1}H NMR: (400 MHz, $CDCl_3$) δ = 8.26 (dt, *J* = 9.0, 2.2 Hz, 2H), 8.18 (dt, *J* = 9.0, 2.1 Hz, 2H), 5.01 – 4.84 (m, 3H), 2.60 (td, *J* = 12.4, 4.8 Hz, 1H), 2.10 – 1.68 (m, 7H), 1.65 (s, 3H), 1.36 – 1.19 (m, 12 H), 0.87 (t, *J* = 6.6 Hz, 3H).

^{13}C NMR: (100 MHz, $CDCl_3$) δ = 208.5, 207.6, 163.3, 150.5, 135.8, 130.8, 123.4, 95.4, 83.8, 78.4, 52.2, 38.7, 36.4, 32.2, 31.9, 30.1, 29.5, 29.3, 26.3, 23.3, 22.7, 19.2, 14.1.

IR: *f* (cm^{-1}) = 3111, 3080, 2926, 2856, 1711, 1461, 1286, 1099, 719.

HRMS (ESI-TOF): *m/z* $[M+H]^+$ = 428.2431 calculated for $C_{25}H_{34}NO_5$; found 428.2417.

Compound (±)-6b

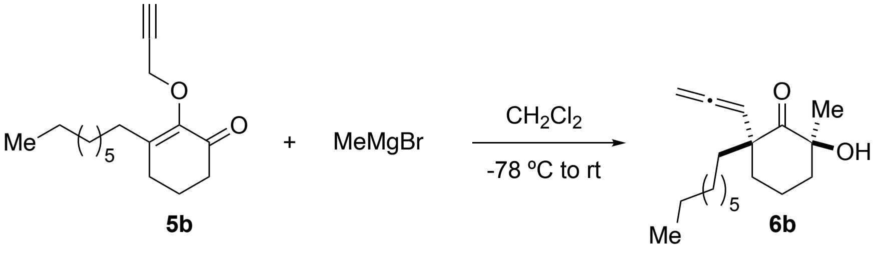

Procedure: Ketone **5b** (74 mg, 0.28 mmol) and CH_2Cl_2 (1.4 mL) were added into a round bottom flask. After cooling the solution to -78 ºC, methylmagnesium bromide (0.12 mL, 0.37 mmol, 3.0 M in Et_2O) was added dropwise. The reaction mixture was allowed to warm to room temperature and stirred for 21 hours, at which the Claisen rearrangement of the 1,2-carbonyl addition intermediate was complete as monitored by TLC. After cooling to 0 °C, the reaction was quenched with saturated NH_4Cl (5 mL), and the resulting mixture was extracted with CH_2Cl_2 (3 x 5 mL). The organic layers were combined, dried over Na_2SO_4, filtered, and then concentrated under vacuum. The crude material was purified with column chromatography using 94:6 hexanes : EtOAc to yield **6b** in 74% yield (58 mg, 0.21 mmol) as a colorless oil. ^{1}H NMR analysis of the crude reaction mixture indicated > 20:1 dr.

^{1}H NMR: (400 MHz, $CDCl_3$) δ = 4.99 (t, *J* = 6.6 Hz, 1H), 4.90 (qd, *J* = 11.2, 7.0 Hz, 2H), 3.90 (s, 1H), 2.19 – 2.03 (m, 2H), 1.92 – 1.77 (m, 2H), 1.76 – 1.65 (m, 3H), 1.63 – 1.51 (m, 2H), 1.42 (s, 3H), 1.34 – 1.20 (m, 12H), 0.88 (t, *J* = 6.4 Hz, 3H).

^{13}C NMR: (100 MHz, $CDCl_3$) δ = 215.7, 207.7, 94.3, 78.4, 76.4, 50.8, 40.4, 38.5, 34.9, 31.9, 30.2, 29.5, 29.3, 28.3, 23.6, 22.7, 19.1, 14.1.

Compound (+)-6c

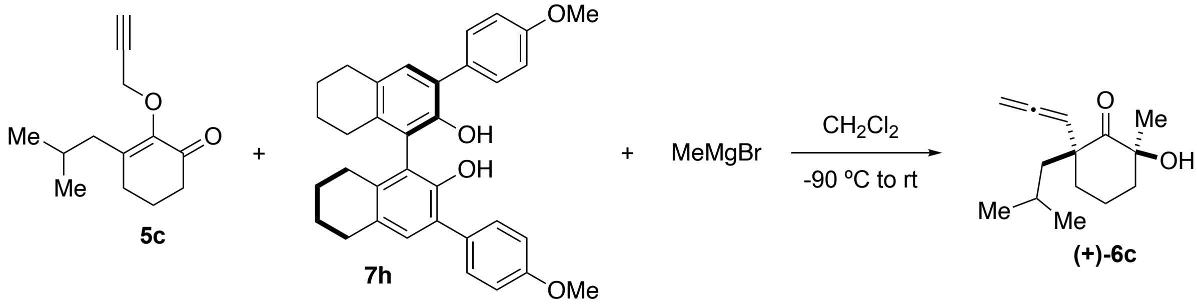

Procedure: Ligand **7h** (364 mg, 0.719 mmol) and ketone **5c** (106 mg, 0.513 mmol) were dissolved in CH_2Cl_2 (10 mL) in a round bottom flask. The solution was cooled to -90 °C, followed by the addition of methylmagnesium bromide (0.67 mL, 2.0 mmol, 3.0 M in Et_2O) dropwise. After stirring at -90 °C until the starting material was fully consumed as monitored by TLC, the reaction mixture was warmed to room temperature and stirred until the Claisen rearrangement of the 1,2-carbonyl addition intermediate was complete as monitored by TLC for a total reaction time of 23 hours. After cooling to 0 °C, the reaction was quenched with a saturated NH_4Cl solution (8 mL) and diluted with DI water (8 mL). The aqueous layer was extracted with CH_2Cl_2 (3 x 10 mL), and the combined organic layers were dried over Na_2SO_4, and concentrated under vacuum. The crude material was purified by column chromatography using 100% CH_2Cl_2 for ligand **7h** recovery followed by 94:6 hexanes : EtOAc to afford **(+)-6c** in 82% yield (93 mg, 0.42 mmol) as a yellow oil. ^{1}H NMR analysis of the crude reaction mixture indicated > 20:1 dr.

Rf: 0.7 in 80:20 hexanes : EtOAc

^{1}H NMR: (400 MHz, $CDCl_3$) δ = 5.00 (t, J = 6.7 Hz, 1H), 4.88 (ddd, J = 24.6, 11.2, 6.9 Hz, 2H), 3.88 (s, 1H), 2.08 (ddq, J = 22.0, 12.9, 2.8 Hz, 2H), 1.83 (tt, J = 13.3, 3.7 Hz, 1H), 1.77 – 1.53 (m, 6H), 1.39 (s, 3H), 0.89 (13.3, 6.0 Hz, 6H).

^{13}C NMR: (100 MHz, $CDCl_3$) δ = 215.4, 207.7, 94.9, 78.4, 76.3, 51.1, 46.7, 40.1, 34.9, 28.6, 24.8, 24.7, 24.3, 19.0.

IR: *ƒ* (cm^{-1}) = 3484, 2953, 2935, 2869, 1700, 1462, 1445, 1367, 1157.

HRMS (ESI-TOF): *m/z* $[M+H]^+$ = 223.1693 calculated for $C_{14}H_{23}O_2$; found 223.1701.

Specific Rotation: $[\alpha]_{25}^{D}$ = +94.2 (c = 1.0 in $CHCl_3$)

HPLC ((+)-6c)-BzNO₂: (S,S)-Whelk-O1, hexane/isopropanol = 99.0/1.0, flow rate = 1.0 mL/min then hexane/isopropanol = 99.2/0.8, flow rate = 1.0 mL/min, λ = 254 nm, t_R = 13.0 min (major), 14.8 min (minor).

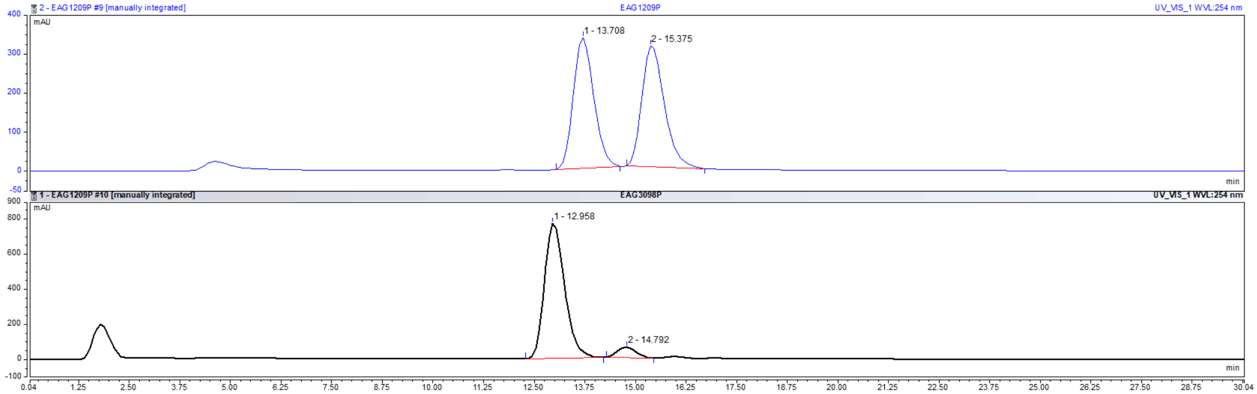

Retention Time (min)	Relative Area (%)	Area (mAU*min)	Height (mAU)
12.958	93.36	443.9189	766.63
14.792	6.64	31.5824	59.35

Compound ((+)-6c)-BzNO₂

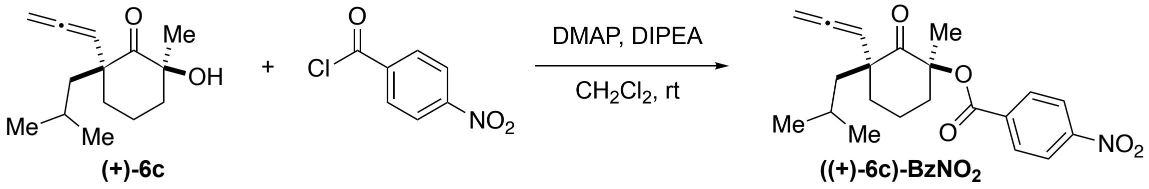

Procedure: Alcohol **(+)-6c** (25 mg, 0.11 mmol) was dissolved in CH_2Cl_2 (0.6 mL). *N*,*N*-Diisopropylethylamine (98 μL, 0.56 mmol) was then added to the solution, followed by 4-nitrobenzoyl chloride (67 mg, 0.36 mmol) and 4-(dimethylamino)pyridine (14 mg, 0.11 mmol). The reaction mixture was then stirred at room temperature until complete consumption of the

starting material as monitored by TLC. The crude reaction mixture was concentrated under vacuum and then purified by column chromatography using 94:6 hexanes : EtOAc to afford compound **((+)-6c)-BzNO$_2$** in 94% yield (37 mg, 0.11 mmol) as a pale yellow oil.

Rf: 0.7 in 80:20 hexanes : EtOAc

^{1}H NMR: (400 MHz, $CDCl_3$) δ = 8.26 (dt, *J* = 8.9, 2.2 Hz, 2H), 8.17 (dt, *J* = 8.8, 2.0 Hz, 2H), 5.00 (t, *J* = 6.7 Hz, 1H), 4.89 (ddd, *J* = 18.2, 11.2, 7.0 Hz, 2H), 2.57 (td, *J* = 12.4, 4.9 Hz, 1H), 2.15 (dt, *J* = 10.9, 3.5 Hz, 1H), 2.04 – 1.78 (m, 5H), 1.77 – 1.62 (m, 5H), 0.94 (dd, *J* = 6.5, 2 Hz, 6H).

^{13}C NMR: (100 MHz, $CDCl_3$) δ = 208.3, 207.7, 163.3, 150.5, 135.8, 130.8, 123.4, 95.7, 83.7, 78.3, 52.6, 47.0, 36.4, 32.6, 26.5, 25.2, 24.9, 24.0, 19.1.

IR: *f* (cm^{-1}) = 3055, 2954, 2937, 2869, 1708, 1526, 1464, 1374, 1374, 719.

HRMS (ESI-TOF): *m/z* $[M+H]^+$ = 372.1805 calculated for $C_{21}H_{26}NO_5$; found 372.1805.

Compound (±)-6c

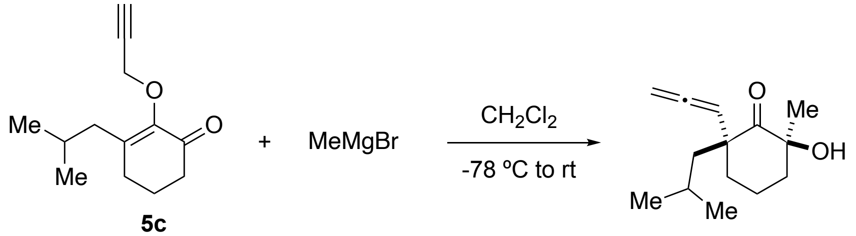

5c

6c

Procedure: Ketone **5c** (148 mg, 0.716 mmol) and CH_2Cl_2 (3.6 mL) were added into a round bottom flask. After cooling the solution to -78 ºC, methylmagnesium bromide (0.31 mL, 0.93 mmol, 3.0 M in Et_2O) was added dropwise. The reaction mixture was allowed to warm to room temperature and stirred for 21 hours, at which the Claisen rearrangement of the 1,2-carbonyl addition intermediate was complete as monitored by TLC. After cooling to 0 °C, the reaction was quenched with saturated NH_4Cl (5 mL), and the resulting mixture was extracted with CH_2Cl_2 (3 x

5 mL). The organic layers were combined, dried over Na_2SO_4, filtered, and then concentrated under vacuum. The crude material was purified with column chromatography using 92:8 hexanes : EtOAc to yield **6a** in 56% yield (88 mg, 0.40 mmol) as a yellow oil. ^{1}H NMR analysis of the crude reaction mixture indicated > 20:1 dr.

^{1}H NMR: (400 MHz, $CDCl_3$) δ = 5.01 (td, *J* = 6.7, 1.0 Hz, 1H), 4.89 (qd, *J* = 11.2, 6.9 Hz, 2H), 3.87 (s, 1H), 2.16 – 2.03 (m, 2H), 1.93 – 1.79 (m, 1H), 1.77 – 1.55 (m, 6H), 1.40 (s, 3H), 0.90 (dd, *J* = 13.4, 6.3 Hz, 6H).

^{13}C NMR: (100 MHz, $CDCl_3$) δ = 215.4, 207.7, 95.0, 78.4, 76.3, 51.2, 46.7, 40.1, 34.9, 28.6, 24.9, 24.8, 24.3, 19.0.

Compound (+)-6d

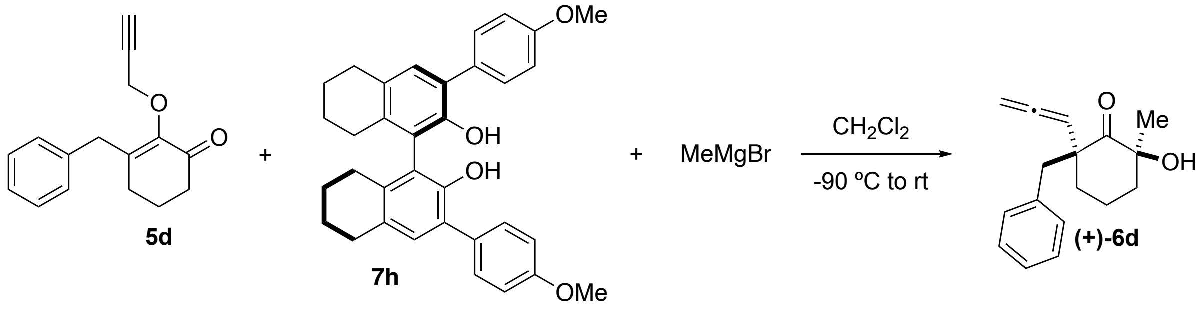

Procedure: Ligand **7h** (363 mg, 0.716 mmol) and ketone **5d** (123 mg, 0.511 mmol) were dissolved in CH_2Cl_2 (10 mL) in a round bottom flask. The solution was cooled to -90 °C, followed by the addition of methylmagnesium bromide (0.66 mL, 2.0 mmol, 3.0 M in Et_2O) dropwise. After stirring at -90 °C until the starting material was fully consumed as monitored by TLC, the reaction mixture was warmed to room temperature and stirred until the Claisen rearrangement of the 1,2-carbonyl addition intermediate was complete as monitored by TLC for a total reaction time of 19 hours. After cooling to 0 °C, the reaction was quenched with a saturated NH_4Cl solution (8 mL) and diluted with DI water (8 mL). The aqueous layer was extracted with CH_2Cl_2 (3 x 10 mL), and

the combined organic layers were dried over Na_2SO_4, and concentrated under vacuum. The crude material was purified by column chromatography using 100% CH_2Cl_2 for ligand **7h** recovery followed by 85:15 hexanes : EtOAc to afford **(+)-6d** in 77% yield (101 mg, 0.394 mmol) as a yellow oil. ^{1}H NMR analysis of the crude reaction mixture indicated > 20:1 dr.

Rf: 0.4 in 80:20 hexanes : EtOAc

^{1}H NMR: (400 MHz, $CDCl_3$) δ = 7.20 – 7.12 (m, 3H), 7.04 (dt, *J* = 6.3, 2.0 Hz, 2H), 4.94 (td, *J* = 6.8, 1.1 Hz, 1H), 4.79 (dd, *J* = 11.2, 6.7 Hz, 1H), 4.57 (dd, *J* = 11.2, 6.7 Hz, 1H), 3.85 (s, 1H), 2.99 (d, *J* = 13.6 Hz, 1H), 2.85 (d, *J* = 13.6 Hz, 1H), 1.99 (dtd, *J* = 11.5, 4.2, 3.5 Hz, 1H), 1.76 – 1.47 (m, 5H), 1.37 (s, 3H).

^{13}C NMR: (100 MHz, $CDCl_3$) δ = 215.0, 207.8, 137.0, 131.1, 127.6, 126.3, 93.8, 78.6, 76.6, 51.7, 44.0, 40.4, 35.1, 28.5, 18.8.

IR: *f* (cm^{-1}) = 3497, 3028, 2935, 2861, 1699, 1445, 1367, 1154, 702.

HRMS (ESI-TOF): *m/z* $[M+H]^+$ = 257.1536 calculated for $C_{17}H_{21}O_2$; found 257.1543.

Specific Rotation: $[\alpha]_{25}^{D}$ = +54.4 (c = 1.0 in $CHCl_3$)

HPLC ((+)-6d)-BzNO$_2$: (S,S)-Whelk-O1, hexane/isopropanol = 97.5/2.5, flow rate = 1.0 mL/min, λ = 254 nm, t_R = 14.0 min (major), 17.2 min (minor).

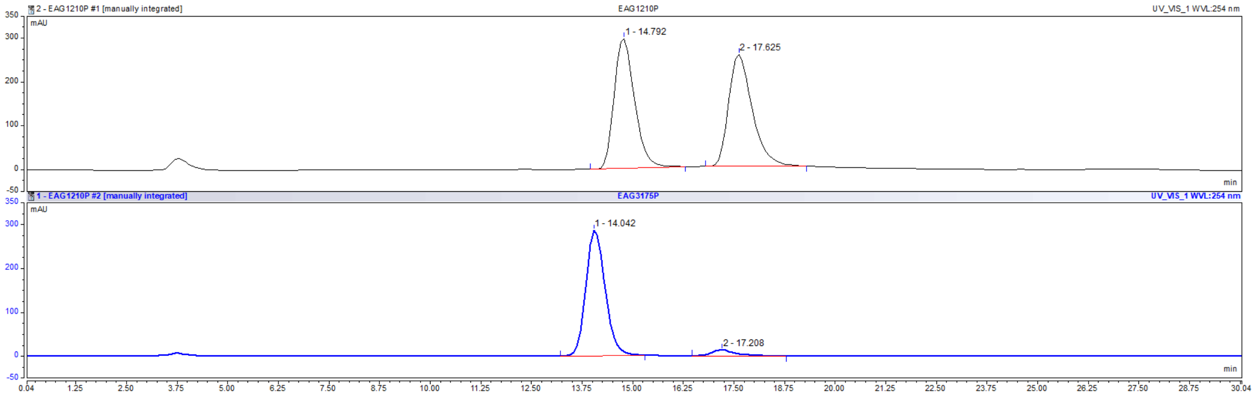

Retention Time (min)	Relative Area (%)	Area (mAU*min)	Height (mAU)
14.042	93.95	159.4065	286.03
17.208	6.05	10.2622	14.27

Compound ((+)-6d)-BzNO₂

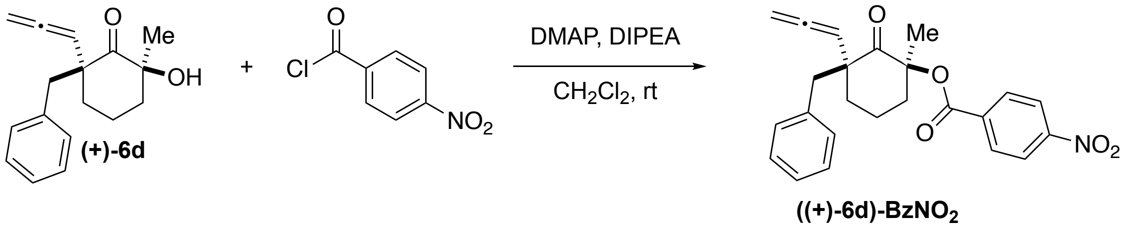

Procedure: Alcohol **(+)-6d** (25 mg, 0.10 mmol) was dissolved in CH_2Cl_2 (0.5 mL). *N*,*N*-Diisopropylethylamine (84 μL, 0.48 mmol) was then added to the solution, followed by 4-nitrobenzoyl chloride (57 mg, 0.31 mmol) and 4-(dimethylamino)pyridine (12 mg, 0.10 mmol). The reaction mixture was then stirred at room temperature until complete consumption of the starting material as monitored by TLC. The crude reaction mixture was concentrated under vacuum and then purified by column chromatography using 93:7 hexanes : EtOAc to afford compound **((+)-6d)-BzNO$_2$** in 97% yield (38 mg, 0.094 mmol) as a yellow solid.

Rf: 0.6 in 80:20 hexanes : EtOAc

^{1}H NMR: (400 MHz, $CDCl_3$) δ = 8.21 (dt, *J* = 8.9, 2.1 Hz, 2H), 8.12 (dt, *J* = 9.1, 2.2 Hz, 2H), 7.22 – 7.08 (m, 5H), 4.91 (t, *J* = 6.7 Hz, 1H), 4.76 (dd, *J* = 11.1, 6.7 Hz, 1H), 4.54 (dd, *J* = 11.2, 6.7 Hz, 1H), 3.24 (d, *J* = 13.4 Hz, 1H), 2.85 (d, *J* = 13.4 Hz, 1H), 2.49 (pd, *J* = 6.4, 6.4 Hz, 1H), 1.98 – 1.82 (m, 2H), 1.70 (ddt, *J* = 14.1, 10.7, 3.0 Hz, 2H), 1.64 – 1.56 (m, 4H).

^{13}C NMR: (100 MHz, $CDCl_3$) δ = 207.9, 163.4, 150.6, 136.9, 135.6, 131.2, 130.8, 127.5, 126.3, 123.4, 94.5, 83.6, 78.4, 52.8, 44.1, 36.6, 32.5, 26.3, 18.8.

IR: *f* (cm^{-1}) = 3083, 3028, 2938, 2870, 1708, 1526, 1452, 1348, 1283.

HRMS (ESI-TOF): *m/z* $[M+H]^+$ = 406.1649 calculated for $C_{24}H_{24}NO_5$; found 406.1652.

Compound (±)-6d

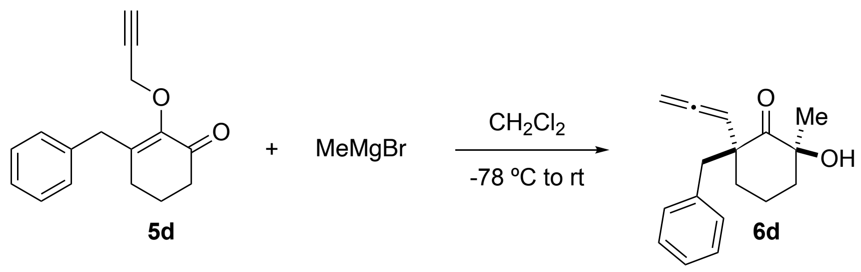

Procedure: Ketone **5d** (98 mg, 0.41 mmol) and CH_2Cl_2 (2.0 mL) were added into a round bottom flask. After cooling the solution to -78 ºC, methylmagnesium bromide (0.18 mL, 0.53 mmol, 3.0 M in Et_2O) was added dropwise. The reaction mixture was allowed to warm to room temperature and stirred for 18 hours, at which the Claisen rearrangement of the 1,2-carbonyl addition intermediate was complete as monitored by TLC. After cooling to 0 °C, the reaction was quenched with saturated NH_4Cl (5 mL), and the resulting mixture was extracted with CH_2Cl_2 (3 x 5 mL). The organic layers were combined, dried over Na_2SO_4, filtered, and then concentrated under vacuum. The crude material was purified with column chromatography using 88:12 hexanes : EtOAc to yield **6d** in 45% yield (47 mg, 0.18 mmol) as a colorless oil. ^{1}H NMR analysis of the crude reaction mixture indicated > 20:1 dr.

^{1}H NMR: (400 MHz, $CDCl_3$) δ = 7.33 – 7.17 (m, 3H), 7.14 (dt, *J* = 6.3, 1.9 Hz, 2H), 5.04 (td, *J* = 6.8, 1.0 Hz), 4.90 (dd, *J* = 11.4, 7.0 Hz, 1H), 4.67 (dd, *J* = 11.2, 6.7 Hz, 1H), 3.94 (s, 1H), 3.09 (d, *J* = 13.6 Hz, 1H), 2.95 (d, *J* = 13.6 Hz, 1H), 2.13 – 2.05 (m, 1H), 1.86 – 1.61 (m, 5H), 1.47 (s, 3H).

^{13}C NMR: (100 MHz, $CDCl_3$) δ = 215.1, 207.9, 137.0, 131.1, 127.7, 126.3, 93.9, 78.6, 76.6, 51.7, 44.0, 40.4, 35.1, 29.5, 18.8.

Compound (+)-6e

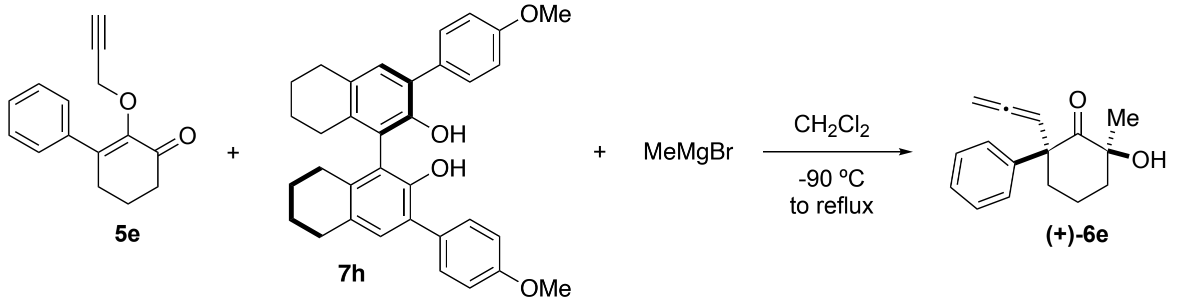

Procedure: Ligand **7h** (317 mg, 0.623 mmol) and ketone **5e** (101 mg, 0.447 mmol) were dissolved in CH_2Cl_2 (12.5 mL) in a round bottom flask. The solution was cooled to -90 °C, followed by the addition of methylmagnesium bromide (0.60 mL, 1.74 mmol, 3.0 M in Et_2O) dropwise. After stirring at -90 °C until the starting material was fully consumed as monitored by TLC, the reaction mixture was warmed to reflux in an oil bath and stirred until the Claisen rearrangement of the 1,2-carbonyl addition intermediate was complete as monitored by TLC for a total reaction time of 24 hours. After cooling to 0 °C, the reaction was quenched with a saturated NH_4Cl solution (8 mL) and diluted with DI water (8 mL). The aqueous layer was extracted with CH_2Cl_2 (3 x 10 mL), and the combined organic layers were dried over Na_2SO_4, and concentrated under vacuum. The crude material was purified by column chromatography using 100% CH_2Cl_2 for ligand **7h** recovery followed by 90:10 hexanes : EtOAc to afford **(+)-6e** in 67% yield (69 mg, 0.30 mmol) as a colorless oil. ^{1}H NMR analysis of the crude reaction mixture indicated > 20:1 dr.

Rf: 0.4 in 80:20 hexanes : EtOAc

^{1}H NMR: (500 MHz, $CDCl_3$) δ = 7.36 – 7.32 (m, 2H), 7.27 – 7.24 (m, 3H), 5.40 (td, *J* = 6.8, 1.2 Hz, 1H), 5.01 (qd, *J* = 11.4, 6.8 Hz, 2H), 3.57 (s, 1H), 2.30 – 2.17 (m, 2H), 2.16 – 2.09, (m, 1H), 2.02 – 1.90 (m, 1H), 1.86 – 1.80 (m, 2H), 1.51 (s, 3H).

^{13}C NMR: (125 MHz, $CDCl_3$) δ = 214.4, 208.3, 143.4, 128.2, 127.6, 127.0, 94.4, 79.1, 76.6, 57.2, 39.3, 38.9, 28.8, 19.1.

IR: f (cm^{-1}) = 3509, 3058, 2931, 2863, 1702, 1514, 1445, 1244, 1067, 979.

HRMS (ESI-TOF): m/z $[M+H]^+$ = 243.1380 calculated for $C_{16}H_{19}O_2$; found 243.1385.

Specific Rotation: $[\alpha]_{25}^{D}$ = +32 (c = 1.0 in $CHCl_3$)

HPLC ((+)-6e)-BzNO₂: (S,S)-Whelk-O1, hexane/isopropanol = 97.5/2.5, flow rate = 1.0 mL/min, λ = 254 nm, t_R = 17.0 min (major), 19.9 min (minor).

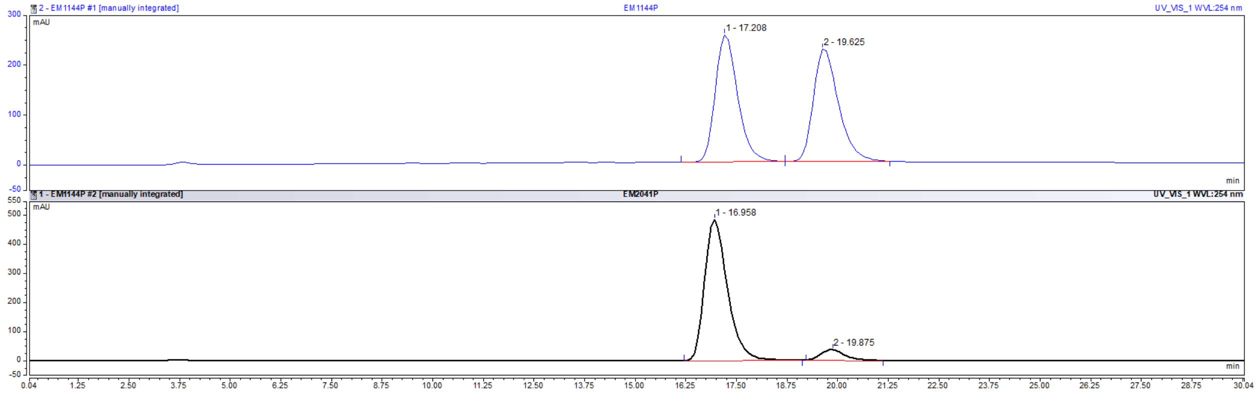

Retention Time (min)	Relative Area (%)	Area (mAU*min)	Height (mAU)
16.958	92.74	318.9538	483.48
19.875	7.26	24.9804	35.77

Compound ((+)-6e)-BzNO₂

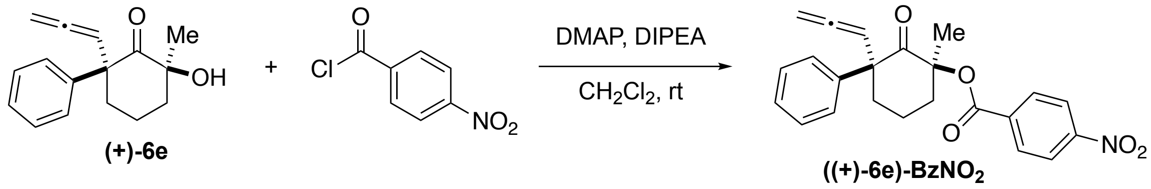

Procedure: Alcohol **(+)-6e** (22 mg, 0.12 mmol) was dissolved in CH_2Cl_2 (0.5 mL). *N*,*N*-Diisopropylethylamine (110 µL, 0.619 mmol) was then added to the solution, followed by 4-nitrobenzoyl chloride (74 mg, 0.40 mmol) and 4-(dimethylamino)pyridine (15 mg, 0.12 mmol). The reaction mixture was then stirred at room temperature until complete consumption of the starting material as monitored by TLC. The crude reaction mixture was concentrated under vacuum

and then purified by column chromatography using 95:5 hexanes : EtOAc to afford compound **((+)-6e)-BzNO$_2$** in 74% yield (30 mg, 0.08 mmol) as a colorless oil.

Rf: 0.8 in 80:20 hexanes : EtOAc

^{1}H NMR: (400 MHz, CDCl$_3$) δ = 8.23 (dt, *J* = 9.2, 2.3 Hz, 2H), 8.08 (dt, *J* = 9.2, 2.2 Hz, 2H), 7.50 – 7.45 (m, 2H), 7.30 – 7.26 (m, 2H), 7.15 (tt, *J* = 6.9, 1.2 Hz, 1H), 5.43 (td, *J* = 6.7, 1.0 Hz, 1H), 4.99 (qd, *J* = 11.4, 6.8 Hz, 2H), 2.87 – 2.78 (m, 1H), 2.49 – 2.41 (m, 1H), 2.23 – 2.17 (m, 1H), 2.07 – 1.96 (m, 3H), 1.77 (s, 3H).

^{13}C NMR: (100 MHz, CDCl$_3$) δ = 208.2, 206.7, 163.3, 150.5, 143.6, 135.6, 130.8, 128.2, 127.6, 126.7, 123.2, 95.6, 84.5, 79.0, 58.6, 37.9, 37.1, 26.1, 19.6.

IR: *f* (cm^{-1}) = 2987, 2955, 2945, 2930, 1727, 1710, 1601, 1525, 1283, 892, 839.

HRMS (ESI-TOF): *m/z* [M+H]+ = 392.1492 calculated for $C_{23}H_{22}NO_5$; found 392.1505.

Compound (±)-6e

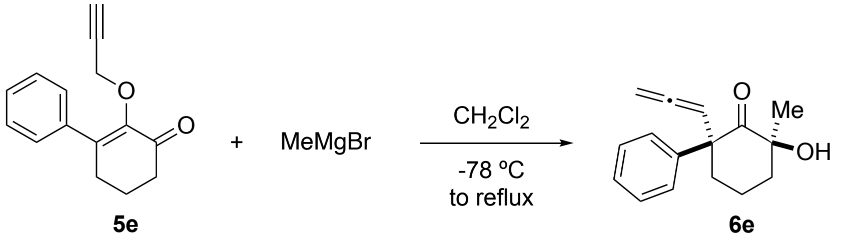

Procedure: Ketone **5e** (154 mg, 0.677 mmol) and CH_2Cl_2 (3.4 mL) were added into a round bottom flask. After cooling the solution to 0 ºC, methylmagnesium bromide (0.30 mL, 0.88 mmol, 3.0 M in Et_2O) was added dropwise. The reaction mixture was allowed to warm to reflux in an oil bath and stirred for 72 hours at which the Claisen rearrangement of the 1,2-carbonyl addition intermediate was complete as monitored by TLC. After cooling to 0 °C, the reaction was quenched with saturated NH_4Cl (5 mL), and the resulting mixture was extracted with CH_2Cl_2 (3 x 5 mL). The organic layers were combined, dried over Na_2SO_4, filtered, and then concentrated under

vacuum. The crude material was purified with column chromatography using 98:2 hexanes : EtOAc to yield **6e** in 71% yield (117 mg, 0.482 mmol) as a colorless oil. ^{1}H NMR analysis of the crude reaction mixture indicated > 20:1 dr.

^{1}H NMR: (500 MHz, $CDCl_3$) δ = 7.29 – 7.21 (m, 2H), 7.21 – 7.14 (m, 3H), 5.32 (td, *J* = 6.7, 1.2 Hz, 1H), 4.92 (qd, *J* = 11.4, 6.7 Hz, 2H), 3.47 (d, *J* = 0.8 Hz, 1H), 2.24 – 2.09 (m, 2H), 2.08 – 1.98 (m, 1H), 1.95 –1.80 (m, 2H), 1.79 – 1.69 (m, 1H),z 1.42 (s, 3H).

^{13}C NMR: (125 MHz, $CDCl_3$) δ = 214.3, 208.3, 143.4, 128.2, 127.6, 126.9, 94.5, 79.0, 76.5, 57.2, 39.3, 38.8, 28.8, 19.1.

Compound (-)-6f

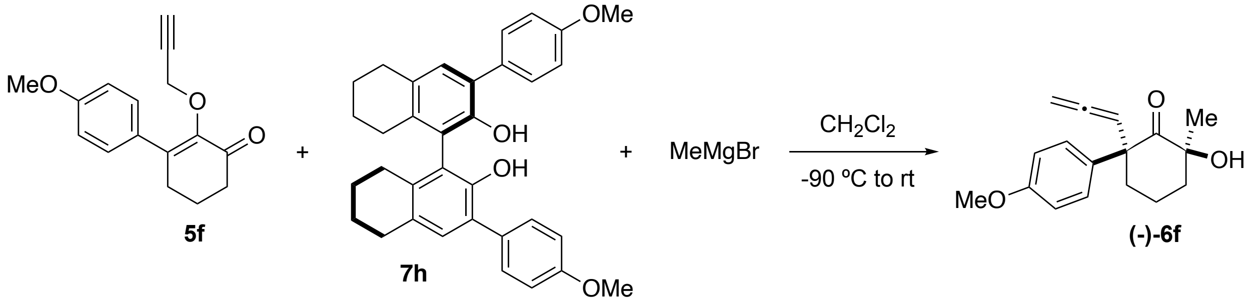

Procedure: Ligand **7h** (296 mg, 0.584 mmol) and ketone **5f** (107 mg, 0.417 mmol) were dissolved in CH_2Cl_2 (8.4 mL) in a round bottom flask. The solution was cooled to -90 °C, followed by the addition of methylmagnesium bromide (0.54 mL, 1.6 mmol, 3.0 M in Et_2O) dropwise. After stirring at -90 °C until the starting material was fully consumed as monitored by TLC, the reaction mixture was warmed to room temperature and stirred until the Claisen rearrangement of the 1,2-carbonyl addition intermediate was complete as monitored by TLC for a total reaction time of 41 hours. After cooling to 0 °C, the reaction was quenched with a saturated NH_4Cl solution (8 mL) and diluted with DI water (8 mL). The aqueous layer was extracted with CH_2Cl_2 (3 x 10 mL), and the combined organic layers were dried over Na_2SO_4, and concentrated under vacuum. The crude

material was purified by column chromatography using 100% CH_2Cl_2 for ligand **7h** recovery followed by 90:10 hexanes : EtOAc to afford **(-)-6f** in 83% yield (94 mg, 0.35 mmol) as a yellow oil. ^{1}H NMR analysis of the crude reaction mixture indicated > 20:1 dr.

Rf: 0.4 in 80:20 hexanes : EtOAc

^{1}H NMR: (400 MHz, $CDCl_3$) δ = 7.16 (dt, *J* = 10.0, 3.4 Hz, 2H), 6.86 (dt, *J* = 9.9, 3.4 Hz, 2H), 5.37 (td, *J* = 6.7, 1.0 Hz, 1H), 4.98 (qd, *J* = 11.3, 6.7 Hz, 2H), 3.79 (s, 3H), 3.57 (s, 1H), 2.28 – 2.17 (m, 2H), 2.16 – 2.05 (m, 1H), 2.00 – 1.89 (m, 2H), 1.87 – 1.77 (m, 1H), 1.49 (s, 3H).

^{13}C NMR: (100 MHz, $CDCl_3$) δ = 214.4, 208.1, 158.4, 135.3, 128.6, 113.6, 94.7, 79.0, 76.6, 56.5, 55.2, 39.4, 38.8, 28.7, 19.1.

IR: *ƒ* (cm^{-1}) = 3487, 2933, 2866, 2836, 1953, 1703, 1461, 1443, 1181, 1033.

HRMS (ESI-TOF): *m/z* $[M+H]^+$ = 273.1485 calculated for $C_{17}H_{21}O_3$; found 273.1491.

Specific Rotation: $[\alpha]_{25}^{D}$ = -22 (c = 1.0 in $CHCl_3$)

HPLC ((+)-6f)-BzNO$_2$: IC-3, hexane/isopropanol = 92/8, flow rate = 1.2 mL/min, λ = 254 nm, t_R = 16.0 min (major), 21.0 min (minor).

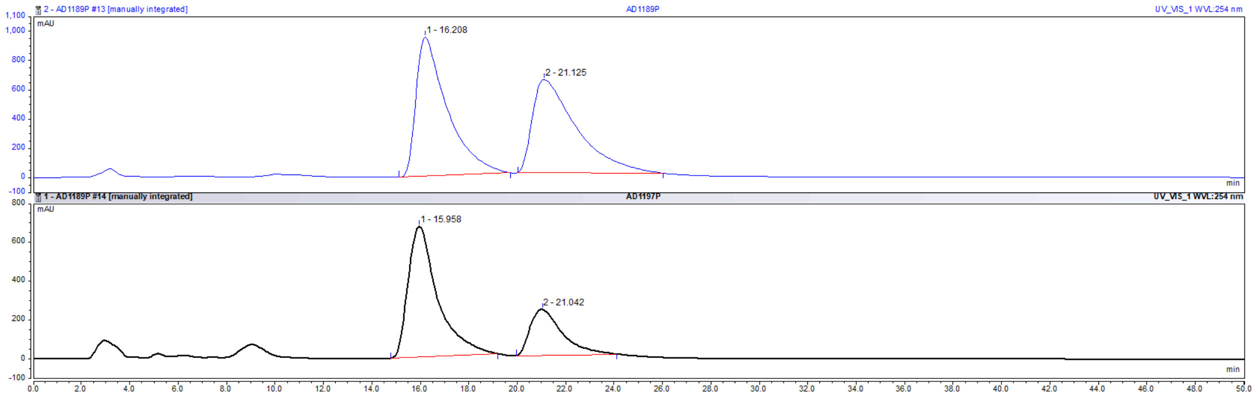

Retention Time (min)	Relative Area (%)	Area (mAU*min)	Height (mAU)
15.958	72.37	949.6140	668.97
21.042	27.63	362.5550	234.95

Compound ((-)-6f)-BzNO₂

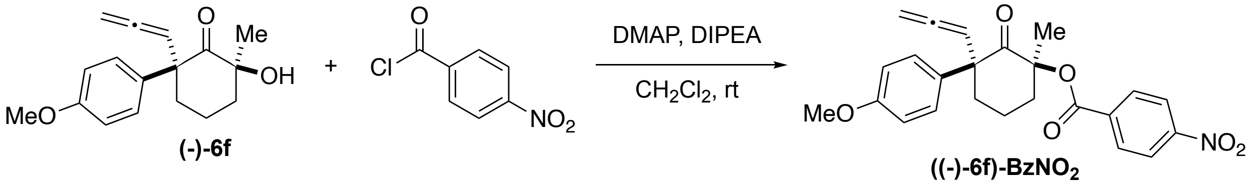

Procedure: Alcohol **(-)-6f** (13 mg, 0.045 mmol) was dissolved in CH_2Cl_2 (2.0 mL). *N,N*-Diisopropylethylamine (40 μL, 0.23 mmol) was then added to the solution, followed by 4-nitrobenzoyl chloride (26 mg, 0.14 mmol) and 4-(dimethylamino)pyridine (5.7 mg, 0.046 mmol). The reaction mixture was then stirred at room temperature until complete consumption of the starting material as monitored by TLC. The crude reaction mixture was concentrated under vacuum and then purified by column chromatography using 80:20 hexanes : EtOAc to afford compound **((-)-6f)-BzNO$_2$** in 93% yield (21 mg, 0.050 mmol) as a yellow solid.

Rf: 0.5 in 80:20 hexanes : EtOAc

^{1}H NMR: (400 MHz, $CDCl_3$) δ = 8.23 (dt, *J* = 9.1, 2.3 Hz, 2H), 8.08 (dt, *J* = 9.1, 2.2 Hz, 2H), 7.36 (dt, *J* = 9.9, 3.2 Hz, 2H), 6.80 (dt, *J* = 10.0, 3.3 Hz, 2H), 5.41 (td, *J* = 6.7, 0.96 Hz, 1H), 4.97 (ddd, *J* = 15.2, 11.2, 6.8 Hz, 2H), 3.71 (s, 3H), 2.85 – 2.73 (m, 1H), 2.49 – 2.39 (m, 1H), 2.21 – 2.13 (m, 1H), 2.07 – 1.95 (m, 3H), 1.75 (s, 3H).

^{13}C NMR: (100 MHz, $CDCl_3$) δ = 208.0, 206.9, 163.3, 158.2, 150.5, 135.6, 135.5, 130.8, 128.7, 123.2, 113.5, 95.9, 84.5, 78.9, 57.9, 55.1, 37.9, 37.3, 25.9, 19.5.

IR: *f* (cm^{-1}) = 2939, 2872, 2836, 1953, 1712, 1527, 1289, 1184.

HRMS (ESI-TOF): *m/z* $[M+H]^+$ = 422.1598 calculated for $C_{24}H_{24}NO_6$; found 422.1604.

Compound (±)-6f

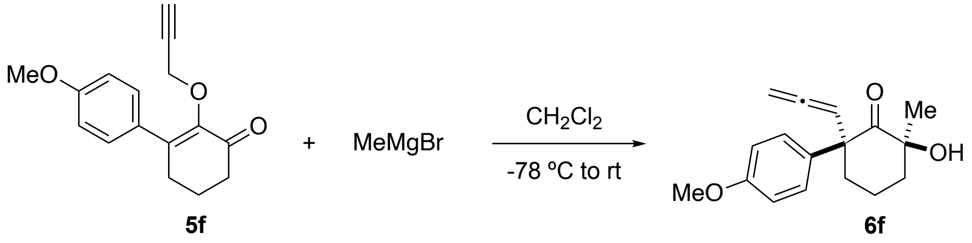

Procedure: Ketone **5f** (150 mg, 0.530 mmol) and CH_2Cl_2 (2.7 mL) were added into a round bottom flask. After cooling the solution to -78 ºC, methylmagnesium bromide (0.23 mL, 0.69 mmol, 3.0 M in Et_2O) was added dropwise. The reaction mixture was allowed to warm to room temperature and stirred for 24 hours, at which the Claisen rearrangement of the 1,2-carbonyl addition intermediate was complete as monitored by TLC. After cooling to 0 °C, the reaction was quenched with saturated NH_4Cl (5 mL), and the resulting mixture was extracted with CH_2Cl_2 (3 x 5 mL). The organic layers were combined, dried over Na_2SO_4, filtered, and then concentrated under vacuum. The crude material was purified with column chromatography using 80:20 hexanes : EtOAc to yield **6f** in 75% yield (119 mg, 0.437 mmol) as a colorless oil. ^{1}H NMR analysis of the crude reaction mixture indicated > 20:1 dr.

^{1}H NMR: (400 MHz, $CDCl_3$) δ = 7.17 (dt, *J* = 10.0, 3.4 Hz, 2H), 6.86 (dt, *J* = 9.9, 3.4 Hz, 2H), 5.38 (td, *J* = 6.7, 1.0 Hz, 1H), 4.98 (qd, *J* = 11.3, 6.7 Hz, 2H), 3.80 (s, 3H), 3.56 (s, 1H), 2.29 – 2.02 (m, 3H), 2.01 – 1.87 (m, 2H), 2.00 – 1.87 (m, 1H), 1.86 – 1.76 (m, 1H), 1.49 (s, 3H).

^{13}C NMR: (100 MHz, $CDCl_3$) δ = 214.5, 208.2, 158.4, 135.3, 128.6, 113.6, 94.7, 79.0, 76.6, 56.6, 55.2, 39.4, 38.8, 28.7, 19.1.

Compound (+)-6g

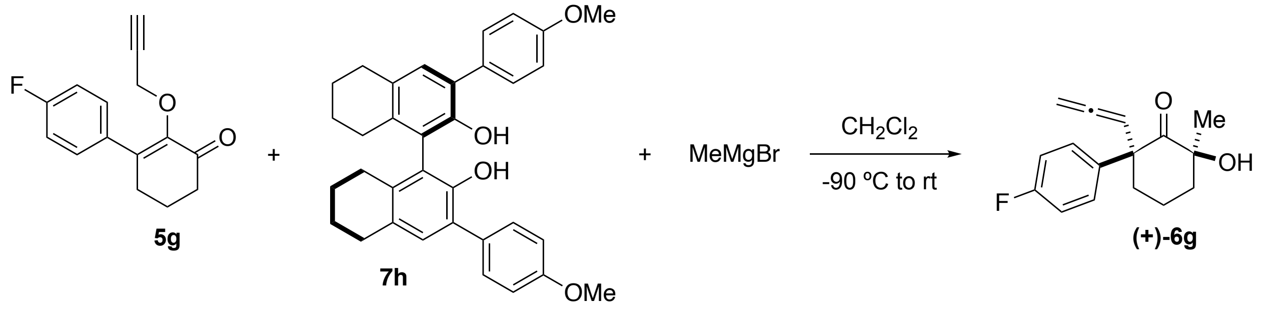

Procedure: Ligand **7h** (304 mg, 0.601 mmol) and ketone **5g** (105 mg, 0.429 mmol) were dissolved in CH_2Cl_2 (8.6 mL) in a round bottom flask. The solution was cooled to -90 °C, followed by the addition of methylmagnesium bromide (0.56 mL, 1.7 mmol, 3.0 M in Et_2O) dropwise. After stirring at -90 °C until the starting material was fully consumed as monitored by TLC, the reaction mixture was warmed to room temperature and stirred until the Claisen rearrangement of the 1,2-carbonyl addition intermediate was complete as monitored by TLC for a total reaction time of 19 hours. After cooling to 0 °C, the reaction was quenched with a saturated NH_4Cl solution (8 mL) and diluted with DI water (8 mL). The aqueous layer was extracted with CH_2Cl_2 (3 x 10 mL), and the combined organic layers were dried over Na_2SO_4, and concentrated under vacuum. The crude material was purified by column chromatography using 100% CH_2Cl_2 for ligand **7h** recovery followed by 90:10 hexanes : EtOAc to afford **(+)-6g** in 98% yield (109 mg, 0.419 mmol) as a colorless oil. ^{1}H NMR analysis of the crude reaction mixture indicated > 20:1 dr.

Rf: 0.4 in 80:20 hexanes : EtOAc

^{1}H NMR: (400 MHz, $CDCl_3$) δ = 7.24 – 7.18 (m, 2H), 7.04 – 6.98 (m, 2H), 5.35 (td, J = 6.6, 1.2 Hz, 1H), 5.02 (ddd, J = 20.2, 11.6, 6.8 Hz, 2H), 3.58 (s, 1H), 2.30 – 2.21 (m, 1H), 2.19 – 2.08 (m, 2H), 2.01 – 1.91 (m, 2H), 1.87 – 1.77 (m, 1H), 1.50 (s, 3H).

^{13}C NMR: (100 MHz, $CDCl_3$) δ = 214.4, 208.3, 162.6, 160.7, 139.2, 139.2, 129.3, 129.3, 115.0, 114.9, 94.3, 79.3, 76.6, 56.8, 39.4, 39.3, 28.9, 19.2.

IR: f (cm^{-1}) = 3498, 3062, 2933, 2866, 1704, 1600, 1508, 1460, 1227, 833.

HRMS (ESI-TOF): m/z $[M+H]^+$ = 261.1285 calculated for $C_{16}H_{18}FO_2$; found 261.1278.

Specific Rotation: $[\alpha]_{25}{}^{D}$ = +51 (c = 1.0 in $CHCl_3$)

HPLC ((+)-6g)-BzNO$_2$: IC-3, hexane/isopropanol = 90.0/10, flow rate = 1.0 mL/min, λ = 254 nm, t_R = 15.5 min (minor), 28.5 min (major).

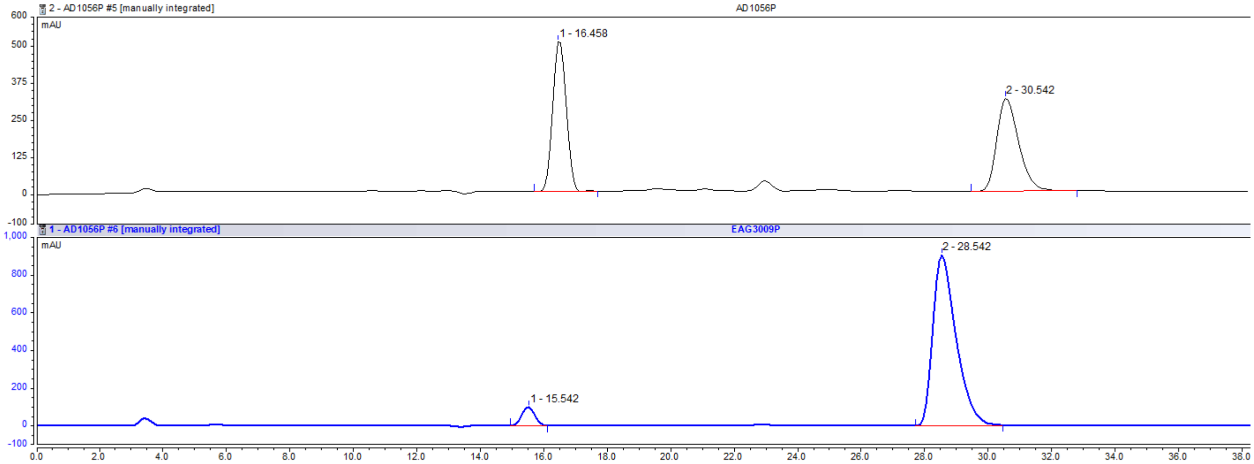

Retention Time (min)	Relative Area (%)	Area (mAU*min)	Height (mAU)
15.542	5.86	47.87619	98.68
28.542	94.14	766.6234	904.73

X-Ray Crystallography: The ellipsoid contour was set at a 50% probability level.

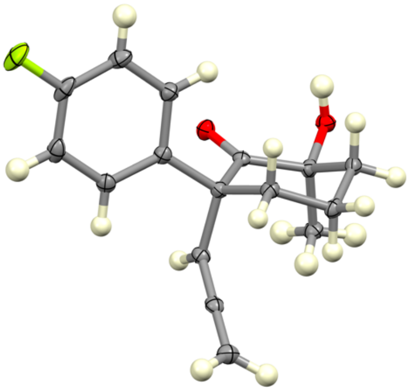

Compound ((+)-6g) -$BzNO_2$

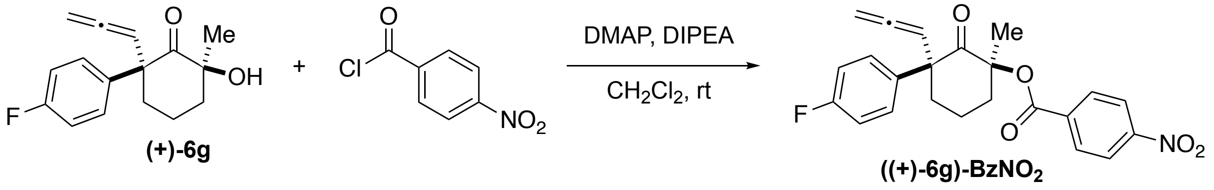

Procedure: Alcohol **(+)-6a** (74 mg, 0.28 mmol) was dissolved in CH_2Cl_2 (1.4 mL). *N,N*-Diisopropylethylamine (25 μL, 1.4 mmol) was then added to the solution, followed by 4-nitrobenzoyl chloride (169 mg, 0.907 mmol) and 4-(dimethylamino)pyridine (35 mg, 0.28 mmol). The reaction mixture was then stirred at room temperature until complete consumption of the starting material as monitored by TLC. The crude reaction mixture was concentrated under vacuum and then purified by column chromatography using 93:7 hexanes : EtOAc to afford compound **((+)-6g)-$BzNO_2$** in 40% yield (46 mg, 0.11 mmol) as a light orange solid.

Rf: 0.6 in 80:20 hexanes : EtOAc

^{1}H NMR: (400 MHz, $CDCl_3$) δ = 8.3 (dt, *J* = 9.1, 2.3 Hz, 2H), 8.15 (dt, *J* = 9.1, 2.2 Hz, 2H), 7.45 (ddd, *J* = 14.1, 11.5, 6.9 Hz, 2H), 6.98 (ddt, *J* = 10.0, 8.5, 3.3 Hz, 2H), 5.35 (td, *J* = 6.7, 1.2 Hz, 1H), 5.02 (ddd, *J* = 14.1, 11.5, 6.9 Hz, 2H), 2.80 (td, *J* = 12.8, 4.4 Hz, 1H), 2.40 (td, *J* = 12.8, 4.4 Hz, 1H), 2.19 (dp, *J* = 14.2, 2.8, 1H), 2.11 – 1.90 (m, 3H), 1.76 (s, 3H).

^{13}C NMR: (100 MHz, $CDCl_3$) δ = 208.3, 206.9, 163.4, 162.8, 160.4, 150.6, 139.7, 139.7, 135.5, 130.8, 129.5, 129.4, 123.3, 115.0, 114.8, 95.2, 84.3, 79.2, 58.3, 38.3, 36.9, 26.4, 19.6.

IR: *f* (cm^{-1}) = 3114, 3077, 2939, 2872, 1710, 1604, 1526, 1509, 1285, 719.

HRMS (ESI-TOF): *m/z* $[M+H]^+$ = 410.1398 calculated for $C_{23}H_{21}FNO_5$; found 410.1413.

Compound (±)-6g

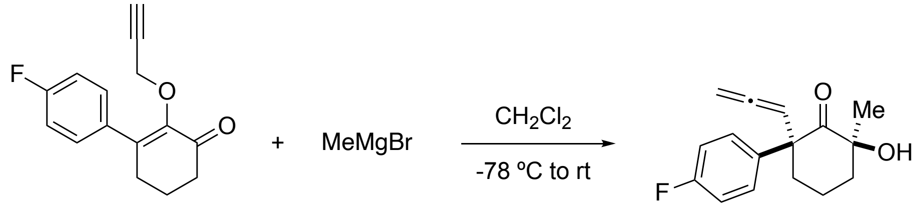

5g 6g

Procedure: Ketone **5g** (136 mg, 0.409 mmol) and CH_2Cl_2 (2.8 mL) were added into a round bottom flask. After cooling the solution to -78 ºC, methylmagnesium bromide (0.24 mL, 0.72 mmol, 3.0 M in Et_2O) was added dropwise. The reaction mixture was allowed to warm to room temperature and stirred for 24 hours, at which the Claisen rearrangement of the 1,2-carbonyl addition intermediate was complete as monitored by TLC. After cooling to 0 °C, the reaction was quenched with saturated NH_4Cl (5 mL), and the resulting mixture was extracted with CH_2Cl_2 (3 x 5 mL). The organic layers were combined, dried over Na_2SO_4, filtered, and then concentrated under vacuum. The crude material was purified with column chromatography using 80:20 hexanes : EtOAc to yield **6g** in 68% yield (98 mg, 0.38 mmol) as a colorless oil. ^{1}H NMR analysis of the crude reaction mixture indicated > 20:1 dr.

^{1}H NMR: (400 MHz, $CDCl_3$) δ = 7.21 (ddd, *J* = 7.0, 5.2, 2.1 Hz, 2H), 7.00 (ddd, *J* = 8.6, 6.6, 2.2 Hz, 2H), 5.35 (td, *J* = 6.7, 1.2 Hz, 1H), 5.0 (ddd, *J* = 20.2, 11.5, 6.8 Hz, 2H), 3.56 (s, 1H), 2.30 – 2.21 (m, 1H), 2.19 – 2.07 (m, 2H), 2.02 – 1.90 (m, 2H), 1.88 – 1.77 (m, 1H), 1.50 (s, 3H).

^{13}C NMR: (100 MHz, $CDCl_3$) δ =214.3, 208.3, 162.8, 160.4, 139.2, 139.1, 129.3, 129.2, 115.0, 114.8, 94.4, 79.2, 76.6, 56.8, 39.4, 39.2, 28.8, 19.1.

Compound (+)-6h

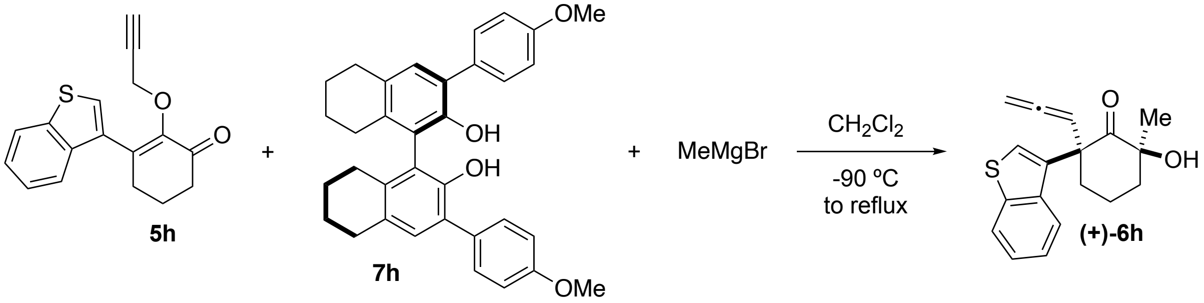

Procedure: Ligand **7h** (339 mg, 0.495 mmol) and ketone **5h** (135 mg, 0.354 mmol) were dissolved in CH_2Cl_2 (9.6 mL) in a round bottom flask. The solution was cooled to -90 °C, followed by the addition of methylmagnesium bromide (0.62 mL, 1.38 mmol, 3.0 M in Et_2O) dropwise. After stirring at -90 °C until the starting material was fully consumed as monitored by TLC, the reaction mixture was warmed to reflux in an oil bath and stirred until the Claisen rearrangement of the 1,2-carbonyl addition intermediate was complete as monitored by TLC for a total reaction time of 24 hours. After cooling to 0 °C, the reaction was quenched with a saturated NH_4Cl solution (8 mL) and diluted with DI water (8 mL). The aqueous layer was extracted with CH_2Cl_2 (3 x 10 mL), and the combined organic layers were dried over Na_2SO_4, and concentrated under vacuum. The crude material was purified by column chromatography using 100% CH_2Cl_2 for ligand **7h** recovery followed by 95:5 hexanes : EtOAc to afford **(+)-6h** in 53% yield (75 mg, 0.25 mmol) as a colorless oil. ^{1}H NMR analysis of the crude reaction mixture indicated > 20:1 dr.

Rf: 0.6 in 80:20 hexanes : EtOAc

^{1}H NMR: (400 MHz, $CDCl_3$) δ = 7.87 – 7.82 (m, 1H), 7.35 – 7.29 (m, 4H), 5.45 (td, J = 6.7, 1.4 Hz, 1H), 5.08 (qd, J = 11.6, 6.7 Hz, 2H), 3.74 (s, 1H), 2.44 (dddd, J = 14.1, 12.6, 4.5, 1.4 Hz, 1H), 2.26 – 2.16 (m, 3H), 2.10 – 1.95 (m, 1H), 1.87 (dp, J = 15.8, 3.9 Hz, 1H), 1.56 (s, 3H).

^{13}C NMR: (100 MHz, $CDCl_3$) δ = 213.7, 208.4, 141.4, 139.0, 136.1, 124.4, 124.0, 123.8, 123.4, 123.3, 94.0, 79.7, 76.6, 54.6, 39.1, 36.5, 29.2, 19.5.

IR: f (cm^{-1}) = 3507, 3060, 2865, 1954, 1705, 1457, 1427, 1366, 1150, 1064.

HRMS (ESI-TOF): m/z [M+H]$^+$ = 299.1100 calculated for $C_{18}H_{19}O_2S$; found 299.1106.

Specific Rotation: $[\alpha]_{25}^{D}$ = +40 (c = 1.0 in $CHCl_3$)

HPLC ((+)-6h)-BzNO₂: (S,S)-Whelk-O1, hexane/isopropanol = 97.5/2.5, flow rate = 1.0 mL/min, λ = 254 nm, t_R = 15.9 min (minor), 19.4 min (major).

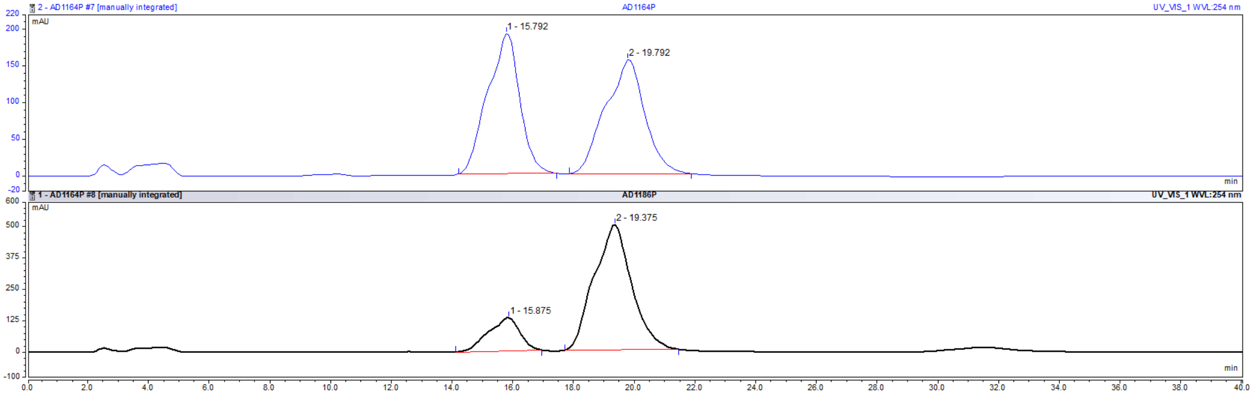

Retention Time (min)	Relative Area (%)	Area (mAU*min)	Height (mAU)
15.875	18.04	157.2720	131.94
19.375	81.96	714.4335	497.00

Compound ((+)-6h)-BzNO₂

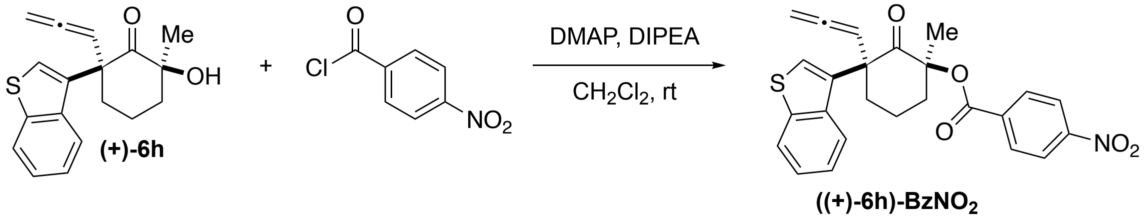

Procedure: Alcohol **(+)-6h** (35 mg, 0.12 mmol) was dissolved in CH_2Cl_2 (0.6 mL). *N*,*N*-Diisopropylethylamine (100 µL, 0.576 mmol) was then added to the solution, followed by 4-nitrobenzoyl chloride (64 mg, 0.35 mmol) and 4-(dimethylamino)pyridine (14 mg, 0.16 mmol). The reaction mixture was then stirred at room temperature until complete consumption of the

starting material as monitored by TLC. The crude reaction mixture was concentrated under vacuum and then purified by column chromatography using 95:5 hexanes : EtOAc to afford compound **((+)-6h)-BzNO$_2$** in 72% yield (39 mg, 0.81 mmol) as a yellow oil.

Rf: 0.4 in 80:20 hexanes : EtOAc

^{1}H NMR: (400 MHz, $CDCl_3$) δ = 8.08 (d, *J* = 9.2 Hz, 2H), 7.94 (d, *J* = 8.2 Hz, 1H), 7.64 (dd, *J* = 7.9, 4.5 Hz, 3H), 7.41 – 7.33 (m, 2H), 7.30 (dd, *J* = 7.6, 7.6 Hz, 1H), 5.86 (t, *J* = 6.7 Hz, 1H), 4.81 (dd, *J* = 11.2, 6.7 Hz, 1H), 4.70 (dd, *J* = 11.2, 6.6 Hz, 1H), 2.96 – 2.88 (m, 1H), 2.80 – 2.72 (m, 1H), 2.33 – 2.19 (m, 2H), 2.07 – 1.95 (m, 2H), 1.79 (s, 3H).

^{13}C NMR: (100 MHz, $CDCl_3$) δ = 207.6, 204.9, 162.9, 150.3, 140.7, 137.4, 136.1, 135.0, 130.4, 124.9, 124.0, 123.7, 123.1, 123.0, 122.5, 95.7, 85.9, 78.9, 56.1, 38.7, 37.4, 24.3, 18.9.

IR: *f* (cm^{-1}) = 2959, 2923, 2853, 1954, 1731, 1715, 1607, 1524, 1347, 1282, 1101, 1014, 852.

HRMS (ESI-TOF): *m/z* $[M+H]^+$ = 448.1213 calculated for $C_{25}H_{22}NO_5S$; found 448.1219.

Compound (±)-6h

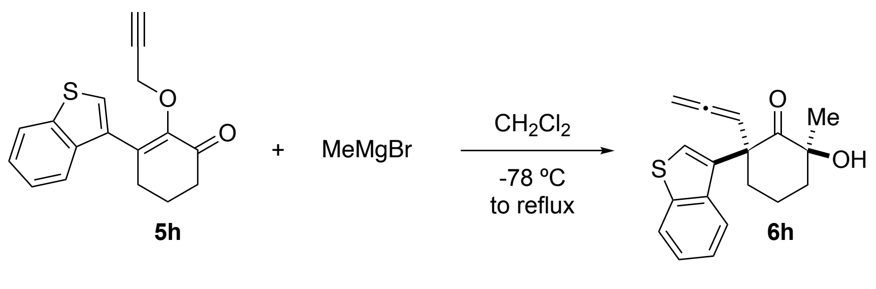

Procedure: Ketone **5h** (153 mg, 0.540 mmol) and CH_2Cl_2 (2.7 mL) were added into a round bottom flask. After cooling the solution to -78 ºC, methylmagnesium bromide (0.23 mL, 0.702 mmol, 3.0 M in Et_2O) was added dropwise. The reaction mixture was allowed to warm to reflux in an oil bath and stirred for 17 hours, at which the Claisen rearrangement of the 1,2-carbonyl addition intermediate was complete as monitored by TLC. After cooling to 0 °C, the reaction was

quenched with saturated NH_4Cl (5 mL), and the resulting mixture was extracted with CH_2Cl_2 (3 x 5 mL). The organic layers were combined, dried over Na_2SO_4, filtered, and then concentrated under vacuum. The crude material was purified with column chromatography using 80:20 hexanes : EtOAc to yield **6h** in 65% yield (100 mg, 0.482 mmol) as a white solid. ^{1}H NMR analysis of the crude reaction mixture indicated > 20:1 dr.

^{1}H NMR: (400 MHz, $CDCl_3$) δ = 7.90 – 7.84 (m, 1H), 7.39 – 7.30 (m, 4H), 5.48 (td, J = 6.7, 1.4 Hz, 1H), 5.10 (qd, J = 11.6, 6.7 Hz, 2H), 3.78 (s, 1H), 2.47 (dddd, J = 14.4, 12.8, 4.5, 1.4 Hz, 1H), 2.31 – 2.16 (m, 3H), 2.03 (dddd, J = 16.1, 12.7, 5.3, 3.8 Hz, 1H), 1.92 – 1.83 (m, 1H), 1.55 (s, 3H).

^{13}C NMR: (100 MHz, $CDCl_3$) δ = 213.6, 208.3, 141.4, 139.0, 136.0, 124.3, 124.0, 123.8, 123.4, 123.3, 93.9, 79.6, 76.6, 54.5, 39.1, 36.4, 29.2, 19.4.

Compound (-)-6i

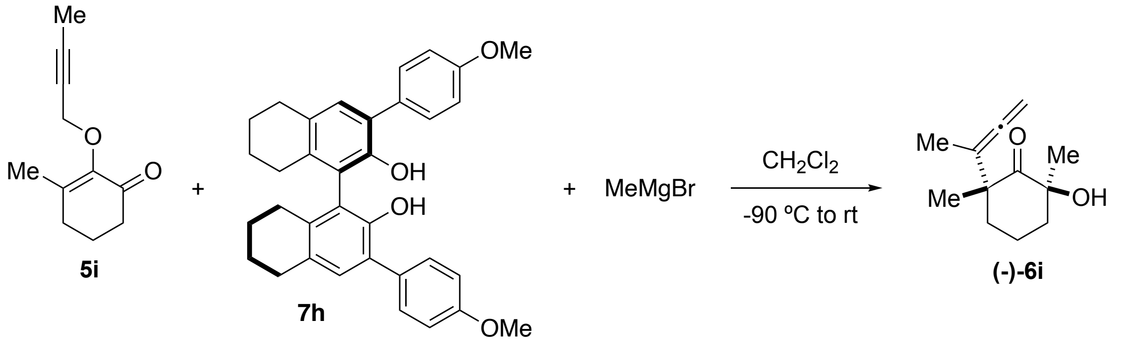

Procedure: Ligand **7h** (398 mg, 0.786 mmol) and ketone **5i** (100 mg, 0.562 mmol) were dissolved in CH_2Cl_2 (11 mL) in a round bottom flask. The solution was cooled to -90 °C, followed by the addition of methylmagnesium bromide (0.73 mL, 2.2 mmol, 3.0 M in Et_2O) dropwise. After stirring at -90 °C until the starting material was fully consumed as monitored by TLC, the reaction mixture was warmed to room temperature and stirred until the Claisen rearrangement of the 1,2-carbonyl addition intermediate was complete as monitored by TLC for a total reaction time of 20

hours. After cooling to 0 °C, the reaction was quenched with a saturated NH_4Cl solution (8 mL) and diluted with DI water (8 mL). The aqueous layer was extracted with CH_2Cl_2 (3 x 10 mL), and the combined organic layers were dried over Na_2SO_4, and concentrated under vacuum. The crude material was purified by column chromatography using 100% CH_2Cl_2 for ligand **7h** recovery followed by 90:10 hexanes : EtOAc to afford **(-)-6i** in 91% yield (99 mg, 0.51 mmol) as a colorless oil. ^{1}H NMR analysis of the crude reaction mixture indicated > 20:1 dr.

Rf: 0.6 in 80:20 hexanes : EtOAc

^{1}H NMR: (400 MHz, $CDCl_3$) δ = 4.92 – 4.76 (m, 2H), 4.07 (s, 1H), 2.24 (m, 1H), 2.08 (m, 1H), 1.90 (m, 1H), 1.63 (m, 6H), 1.43 (s, 3H), 1.21 (s, 3H).

^{13}C NMR: (100 MHz, $CDCl_3$) δ = 216.2, 205.5, 101.6, 77.2, 77.1, 50.6, 42.0, 39.0, 26.5, 24.7, 19.4, 15.5.

IR: *f* (cm^{-1}) = 3500, 2969, 2932, 2867, 1698, 1447, 1370, 1011.

HRMS (ESI-TOF): *m/z* $[M+H]^+$ = 195.1380 calculated for $C_{12}H_{19}O_2$; found 195.1361.

Specific Rotation: $[\alpha]_{25}^{D}$ = -162 (c = 1.0 in $CHCl_3$)

HPLC ((-)-6i)-BzNO$_2$: IC-3, hexane/isopropanol = 90.0/10, flow rate = 1.0 mL/min, λ = 254 nm, t_R = 11.5 min (major), 20.6 min (minor).

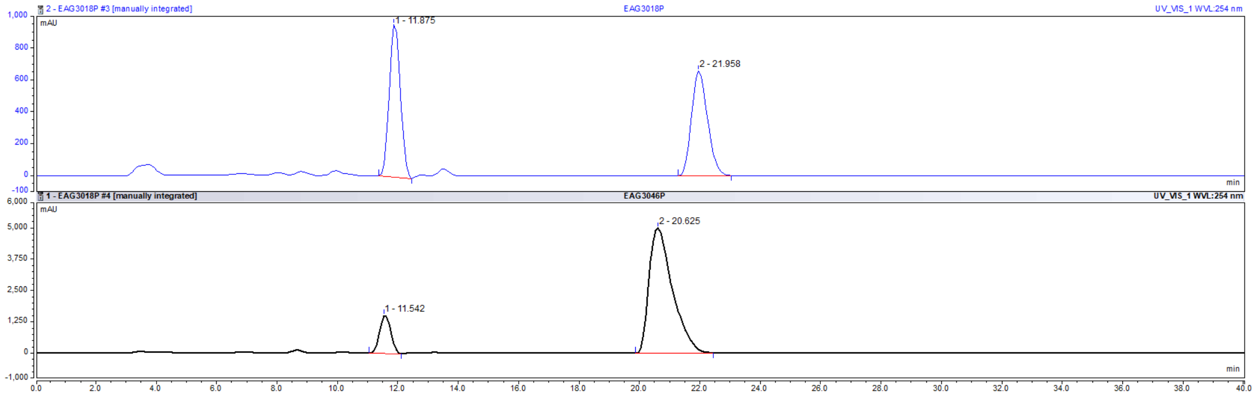

Retention Time (min)	Relative Area (%)	Area (mAU*min)	Height (mAU)
11.542	12.71	649.8131	1502.63
20.625	87.29	4461.5445	4979.64

Compound ((-)-6i)-BzNO₂

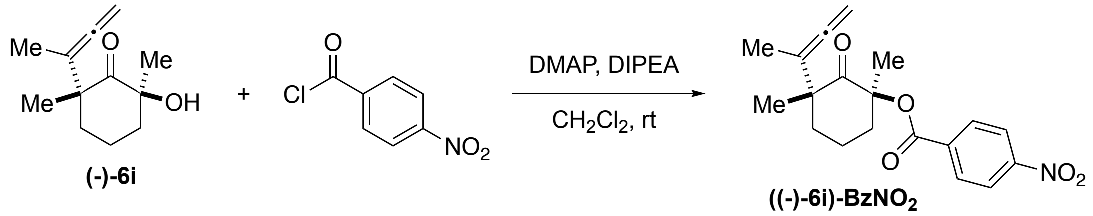

Procedure: Alcohol **(-)-6i** (37 mg, 0.19 mmol) was dissolved in CH_2Cl_2 (0.9 mL). *N*,*N*-Diisopropylethylamine (164 µL, 0.939 mmol) was then added to the solution, followed by 4-nitrobenzoyl chloride (112 mg, 0.601 mmol) and 4-(dimethylamino)pyridine (23 mg, 0.19 mmol). The reaction mixture was then stirred at room temperature until complete consumption of the starting material as monitored by TLC. The crude reaction mixture was concentrated under vacuum and then purified by column chromatography using 93:7 hexanes : EtOAc to afford compound **((-)-6i)-BzNO₂** in 99% yield (62 mg, 0.18 mmol) as a colorless oil.

Rf: 0.7 in 80:20 hexanes : EtOAc

^{1}H NMR: (400 MHz, $CDCl_3$) δ = 8.26 (dt, *J* = 8.9, 2.2 Hz, 2H), 8.18 (dt, *J* = 9.0, 2.1 Hz, 2H), 4.84 (dq, *J* = 10.3, 3.1 Hz, 1H), 4.77 (dq, *J* = 10.3, 3.1, Hz, 1H), 2.65 (td, *J* = 12.5, 4.0 Hz, 1H), 2.24 – 2.15 (m, 1H), 2.07 – 1.81 (m, 3H), 1.79 – 1.71 (m, 1H), 1.66 (s, 3H), 1.62 (t, *J* = 3.1 Hz, 3H), 1.30 (s, 3H).

^{13}C NMR: (100 MHz, $CDCl_3$) δ = 207.2, 205.9, 163.3, 150.5, 136.0, 130.8, 123.4, 101.6, 85.0, 76.9, 51.2, 37.6, 36.3, 25.0, 24.8, 19.3, 15.1.

IR: *f* (cm^{-1}) = 3080, 3055, 2965, 2933, 1709, 1526, 1448, 1349, 1283, 718.

HRMS (ESI-TOF): *m/z* $[M+H]^+$ = 344.1492 calculated for $C_{19}H_{22}NO_5$; found 344.1494.

Compound (±)-6i

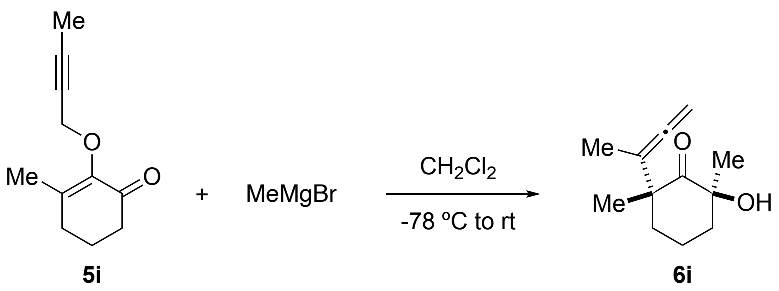

Procedure: Ketone **5i** (102 mg, 0.571 mmol) and CH_2Cl_2 (2.9 mL) were added into a round bottom flask. After cooling the solution to -78 ºC, methylmagnesium bromide (0.25 mL, 0.74 mmol, 3.0 M in Et_2O) was added dropwise. The reaction mixture was allowed to warm to room temperature and stirred for 20 hours, at which the Claisen rearrangement of the 1,2-carbonyl addition intermediate was complete as monitored by TLC. After cooling to 0 °C, the reaction was quenched with saturated NH_4Cl (5 mL), and the resulting mixture was extracted with CH_2Cl_2 (3 x 5 mL). The organic layers were combined, dried over Na_2SO_4, filtered, and then concentrated under vacuum. The crude material was purified with column chromatography using 90:10 hexanes : EtOAc to yield **6a** in 74% yield (82 mg, 0.42 mmol) as a colorless oil. ^{1}H NMR analysis of the crude reaction mixture indicated > 20:1 dr.

^{1}H NMR: (400 MHz, $CDCl_3$) δ = 4.88 (dq, *J* = 10.2, 3.12 Hz, 1H), 4.79 (dq, *J* = 10.3, 3.0 Hz, 1H), 4.07 (s, 1H), 2.28 – 2.20 (m, 1H), 2.08 (dq, *J* = 12.9, 3.2 Hz, 1H), 1.97 – 1.83 (m, 1H), 1.75 – 1.64 (m, 1H), 1.63 – 1.52 (m, 5H), 1.43 (s, 3H), 1.21 (s, 3H).

^{13}C NMR: (100 MHz, $CDCl_3$) δ = 216.2, 205.5, 101.6, 77.2, 77.1, 50.6, 42.0, 39.0, 26.5, 24.7, 19.3, 15.5.

Compound (+)-6j

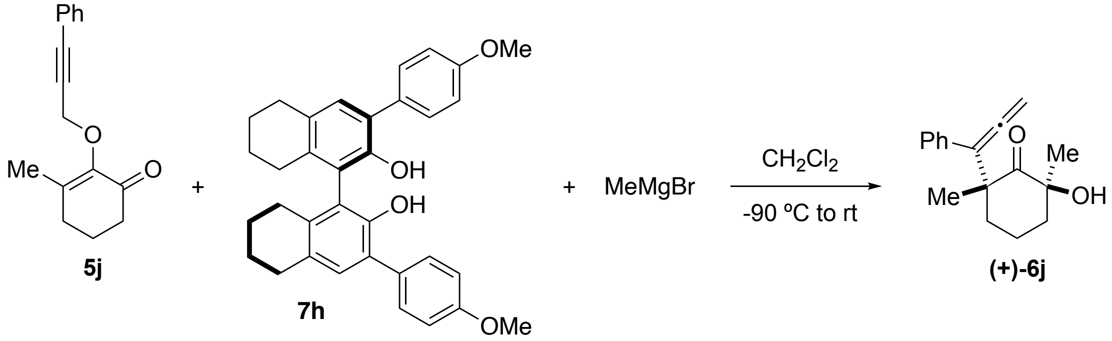

Procedure: Ligand **7h** (269 mg, 0.532 mmol) and ketone **5j** (91 mg, 0.38 mmol) were dissolved in CH_2Cl_2 (7.6 mL) in a round bottom flask. The solution was cooled to -90 °C, followed by the addition of methylmagnesium bromide (0.49 mL, 1.5 mmol, 3.0 M in Et_2O) dropwise. After stirring at -90 °C until the starting material was fully consumed as monitored by TLC, the reaction mixture was warmed to room temperature and stirred until the Claisen rearrangement of the 1,2-carbonyl addition intermediate was complete as monitored by TLC for a total reaction time of 20 hours. After cooling to 0 °C, the reaction was quenched with a saturated NH_4Cl solution (8 mL) and diluted with DI water (8 mL). The aqueous layer was extracted with CH_2Cl_2 (3 x 10 mL), and the combined organic layers were dried over Na_2SO_4, and concentrated under vacuum. The crude material was purified by column chromatography using 100% CH_2Cl_2 for ligand **7h** recovery followed by 85:15 hexanes : EtOAc to afford **(+)-6j** in 72% yield (70 mg, 0.27 mmol) as a pale yellow solid. ^{1}H NMR analysis of the crude reaction mixture indicated > 20:1 dr.

Rf: 0.7 in 80:20 hexanes : EtOAc

^{1}H NMR: (400 MHz, $CDCl_3$) δ = 7.33 – 7.23 (m, 4H), 7.19 (tt, *J* = 6.3, 1.8 Hz, 1H), 5.28 (d, *J* = 12.2 Hz, 1H), 5.20 (d, *J* = 12.2 Hz, 1H), 4.21 (s, 1H), 2.44 (dq, *J* = 14.8, 3.9 Hz, 1H), 2.13 (dq, *J* = 12.7, 3.3 Hz, 1H), 1.95 (qdd, *J* = 13.6, 3.6, 2.6 Hz, 1H), 1.70 (td, *J* = 13.1, 4.2 Hz, 1H), 1.64 – 1.52 (m, 2H), 1.49 (s, 3H), 1.22 (s, 3H).

^{13}C NMR: (100 MHz, $CDCl_3$) δ = 217.1, 208.2, 135.1, 128.5, 127.3, 127.1, 109.9, 80.2, 77.8, 51.0, 44.2, 41.9, 26.8, 25.4, 19.6.

IR: *ƒ* (cm^{-1}) = 3360, 3096, 3031, 2995, 2932, 1702, 1496, 1446, 1168, 698.

HRMS (ESI-TOF): *m/z* $[M+H]^+$ = 257.1536 calculated for $C_{17}H_{21}O_2$; found 257.1535.

Specific Rotation: $[\alpha]_{25}^{D}$ = +80 (c = 1.0 in $CHCl_3$)

HPLC (+)-6j: IC-3, hexane/isopropanol = 99.0/1.0, flow rate = 1.0 mL/min, λ = 254 nm, t_R = 18.3 min (minor), 20.125 min (major).

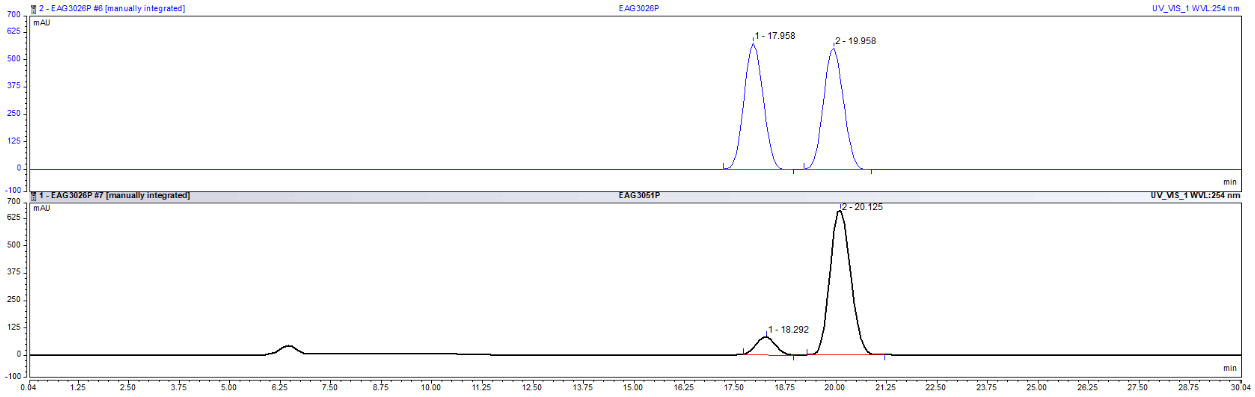

Retention Time (min)	Relative Area (%)	Area (mAU*min)	Height (mAU)
18.292	10.22	44.6198	80.39
20.125	89.78	391.8514	663.66

X-Ray Crystallography: The ellipsoid contour was set at a 50% probability level.

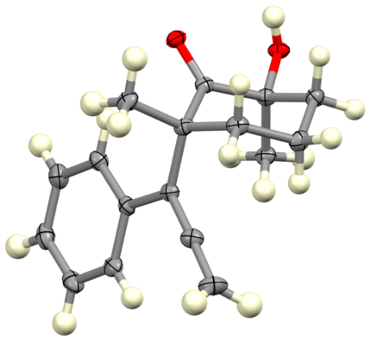

Compound (±)-6j

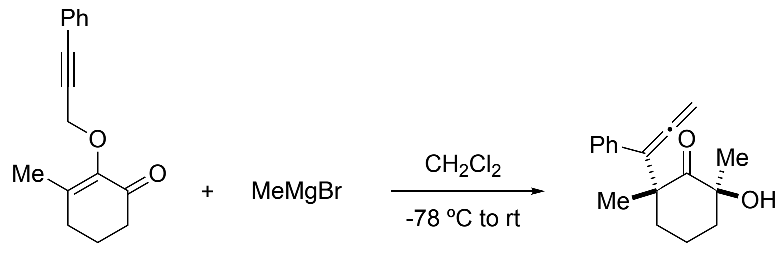

Procedure: Ketone **5j** (94 mg, 0.39 mmol) and CH_2Cl_2 (2.0 mL) were added into a round bottom flask. After cooling the solution to -78 ºC, methylmagnesium bromide (0.17 mL, 0.51 mmol, 3.0 M in Et_2O) was added dropwise. The reaction mixture was allowed to warm to room temperature and stirred for 26 hours, at which the Claisen rearrangement of the 1,2-carbonyl addition intermediate was complete as monitored by TLC. After cooling to 0 °C, the reaction was quenched with saturated NH_4Cl (5 mL), and the resulting mixture was extracted with CH_2Cl_2 (3 x 5 mL). The organic layers were combined, dried over Na_2SO_4, filtered, and then concentrated under vacuum. The crude material was purified with column chromatography using 90:10 hexanes : EtOAc to yield **6j** in 83% yield (83 mg, 0.32 mmol) as a colorless oil. ^{1}H NMR analysis of the crude reaction mixture indicated > 20:1 dr.

^{1}H NMR: (400 MHz, $CDCl_3$) δ = 7.34 – 7.24 (m, 4H), 7.19 (tt, *J* = 6.3, 1.5 Hz, 1H), 5.28 (d, *J* = 12.2 Hz, 1H), 5.20 (d, *J* = 12.2 Hz, 1H), 4.21 (s, 1H), 2.44 (dt, *J* = 14.8, 3.9 Hz, 1H), 2.13 (dq, *J* = 12.7, 2.8 Hz, 1H), 1.95 (qt, *J* = 13.5, 2.9 Hz, 1H), 1.70 (td, *J* = 14.9, 3.9 Hz, 1H), 1.64 – 1.53 (m, 2H), 1.49 (s, 3H), 1.23 (s, 3H).

^{13}C NMR: (100 MHz, $CDCl_3$) δ = 217.1, 208.3, 135.1, 128.5, 127.4, 127.1, 109.9, 80.2, 77.8, 51.0, 44.2, 41.9, 26.8, 25.4, 19.6.

Compound (-)-6k

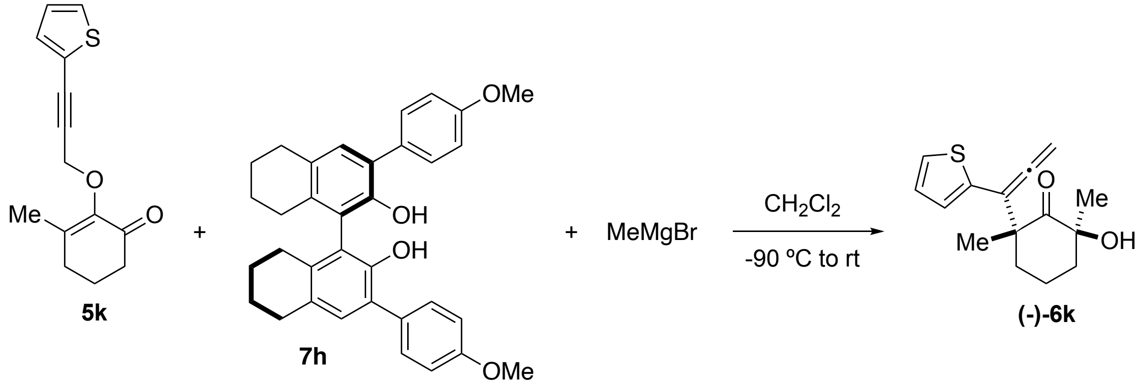

Procedure: Ligand **7h** (391 mg, 0.772 mmol) and ketone **5k** (136 mg, 0.552 mmol) were dissolved in CH_2Cl_2 (11 mL) in a round bottom flask. The solution was cooled to -90 °C, followed by the addition of methylmagnesium bromide (0.72 mL, 2.15 mmol, 3.0 M in Et_2O) dropwise. After stirring at -90 °C until the starting material was fully consumed as monitored by TLC, the reaction mixture was warmed to room temperature and stirred until the Claisen rearrangement of the 1,2-carbonyl addition intermediate was complete as monitored by TLC for a total reaction time of 22 hours. After cooling to 0 °C, the reaction was quenched with a saturated NH_4Cl solution (8 mL) and diluted with DI water (8 mL). The aqueous layer was extracted with CH_2Cl_2 (3 x 10 mL), and the combined organic layers were dried over Na_2SO_4, and concentrated under vacuum. The crude material was purified by column chromatography using 100% CH_2Cl_2 for ligand **7h** recovery followed by 95:5 hexanes : EtOAc to afford **(-)-6k** in 93% yield (134 mg, 0.511 mmol) as a white solid. ^{1}H NMR analysis of the crude reaction mixture indicated > 20:1 dr.

Rf: 0.4 in 80:20 hexanes : EtOAc

^{1}H NMR: (400 MHz, $CDCl_3$) δ = 7.15 – 7.11 (m, 1H), 6.89 – 6.85 (m, 2H), 5.36 (d, J = 12.7 Hz, 1H), 5.28 (d, J = 12.8 Hz, 1H), 4.18 (s, 1H), 2.38 (dq, J = 14.8, 3.7 Hz, 1H), 2.13 (dq, J = 12.8, 3.0 Hz, 1H), 1.99 – 1.88 (m, 1H), 1.74 – 1.65 (m, 1H), 1.64 – 1.55 (m, 2H), 1.51 (s, 3H), 1.26 (s, 3H).

^{13}C NMR: (400 MHz, $CDCl_3$) δ = 216.7, 207.3, 138.7, 127.6, 124.9, 124.8, 105.8, 81.3, 77.8, 51.2, 43.9, 41.4, 26.2, 25.0, 19.5.

IR: *f* (cm^{-1}) = 3484, 2972, 2933, 2868, 1933, 1699, 1449, 1375, 1012.

HRMS (ESI-TOF): *m/z* $[M+H]^+$ = 263.1100 calculated for $C_{15}H_{19}O_2S$; found 263.1109.

Specific Rotation: $[\alpha]_{25}^{D}$ = -36 (c = 1.0 in $CHCl_3$)

HPLC ((-)-6k)-BzNO₂: IC-3, hexane/isopropanol = 90/10, flow rate = 1.0 mL/min, λ = 254 nm, t_R = 15.7 min (major), 18.3 min (minor).

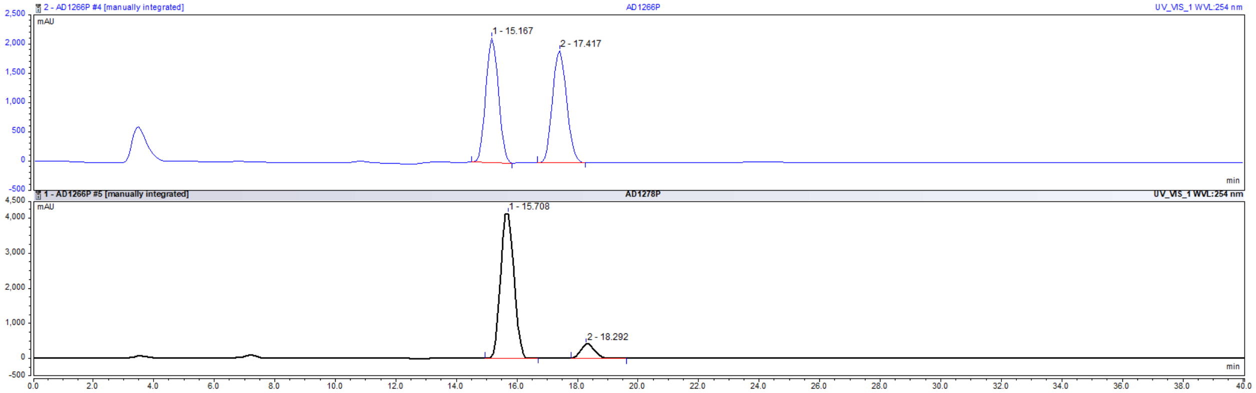

Retention Time (min)	Relative Area (%)	Area (mAU*min)	Height (mAU)
15.708	90.92	2111.9126	4133.45
18.292	9.08	210.8516	396.40

Compound ((-)-6k)-BzNO₂

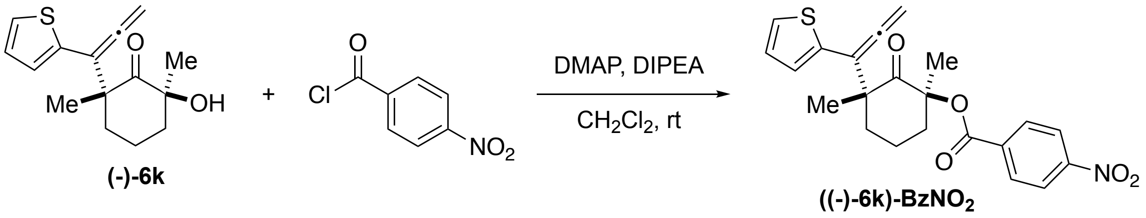

Procedure: Alcohol **(-)-6k** (49 mg, 0.19 mmol) was dissolved in CH_2Cl_2 (0.9 mL). *N*,*N*-Diisopropylethylamine (164 μL, 0.940 mmol) was then added to the solution, followed by 4-

nitrobenzoyl chloride (112 mg, 0.601 mmol) and 4-(dimethylamino)pyridine (23 mg, 0.19 mmol). The reaction mixture was then stirred at room temperature until complete consumption of the starting material as monitored by TLC. The crude reaction mixture was concentrated under vacuum and then purified by column chromatography using 90:10 hexanes : EtOAc to afford compound **((-)-6k)-BzNO$_2$** in 72% yield (56 mg, 0.14 mmol) as a yellow oil.

Rf: 0.7 in 80:20 hexanes : EtOAc

^{1}H NMR: (400 MHz, CDCl$_3$) δ = 8.20 (t, *J* = 2.2 Hz, 1H), 8.18 (t, *J* = 2.1 Hz, 1H), 8.12 (t, *J* = 2.1 Hz, 1H), 8.10 (t, *J* = 1.9 Hz, 1H), 7.19 (s, 1H), 6.94 (dd, *J* = 3.6, 1.0 Hz, 1H), 6.88 (dd, *J* = 5.2, 3.7 Hz, 1H), 5.20 (d, *J* = 12.5 Hz, 1H), 5.11 (d, *J* = 12.5 Hz, 1H), 2.53 (dd, *J* = 12.9, 4.9 Hz, 1H), 2.30 – 2.21 (m, 1H), 2.09 – 1.89 (m, 2H), 1.83 – 1.77 (m, 1H), 1.76 – 1.67 (m, 1H), 1.57 (s, 3H), 1.47 (s, 3H).

^{13}C NMR: (100 MHz, CDCl$_3$) δ = 207.8, 206.6, 163.3, 150.5, 137.4, 136.2, 130.8, 127.6, 126.1, 124.9, 123.4, 105.4, 86.4, 80.2, 52.2, 39.1, 38.6, 26.0, 23.9, 19.1.

IR: *f* (cm^{-1}) = 2936, 2869, 1714, 1526, 1348, 1282, 1102, 868.

HRMS (ESI-TOF): *m/z* [M+H]$^+$ = 412.1213 calculated for $C_{22}H_{22}NO_5S$; found 412.1221.

Compound (±)-6k

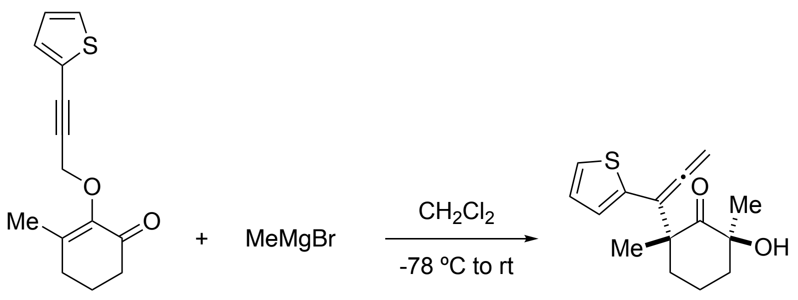

5k 6k

Procedure: Ketone **5k** (33 mg, 0.13 mmol) and CH_2Cl_2 (0.7 mL) were added into a round bottom flask. After cooling the solution to -78 ºC, methylmagnesium bromide (60 μL, 0.17 mmol, 3.0 M

in Et_2O) was added dropwise. The reaction mixture was allowed to warm to room temperature and stirred for 19 hours, at which the Claisen rearrangement of the 1,2-carbonyl addition intermediate was complete as monitored by TLC. After cooling to 0 °C, the reaction was quenched with saturated NH_4Cl (5 mL), and the resulting mixture was extracted with CH_2Cl_2 (3 x 5 mL). The organic layers were combined, dried over Na_2SO_4, filtered, and then concentrated under vacuum. The crude material was purified with column chromatography using 90:10 hexanes : EtOAc to yield **6k** in 75% yield (26 mg, 0.099 mmol) as a white solid. ^{1}H NMR analysis of the crude reaction mixture indicated > 20:1 dr.

^{1}H NMR: (500 MHz, $CDCl_3$) δ = 7.13 (dd, *J* = 4.8, 1.3 Hz, 1H), 6.87 (qd, *J* = 3.9, 1.9 Hz, 2H), 5.37 (d, *J* = 12.7 Hz, 1H), 5.29 (d, *J* = 12.7 Hz, 1H), 4.20 (d, *J* = 0.9 Hz, 1H), 2.38 (dq, *J* = 14.1, 3.6, 2.9 Hz, 1H), 2.13 (dq, *J* = 12.9, 3.2 Hz, 1H), 2.01 – 1.88 (m, 1H), 1.69 (td, *J* = 13.3, 4.2 Hz, 1H), 1.64 – 1.54 (m, 2H), 1.51 (s, 3H), 1.26 (s, 3H).

^{13}C NMR: (125 MHz, $CDCl_3$) δ = 216.7, 207.2, 138.7, 127.6, 124.9, 124.8, 105.8, 81.3, 77.8, 51.2, 43.9, 41.4, 26.2, 25.0, 19.5.

Compound (+)-6l

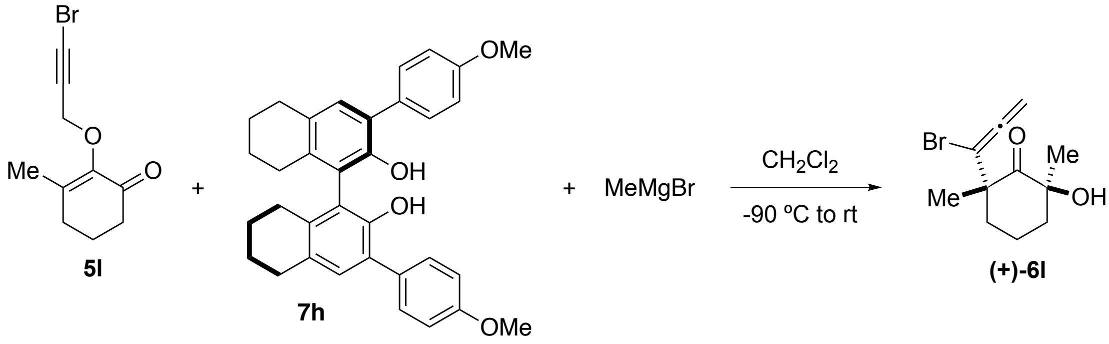

Procedure: Ligand **7h** (293 mg, 0.578 mmol) and ketone **5l** (100 mg, 0.413 mmol) were dissolved in CH_2Cl_2 (8.3 mL) in a round bottom flask. The solution was cooled to -90 °C, followed by the

addition of methylmagnesium bromide (0.54 mL, 1.6 mmol, 3.0 M in Et_2O) dropwise. After stirring at -90 °C until the starting material was fully consumed as monitored by TLC, the reaction mixture was warmed to room temperature and stirred until the Claisen rearrangement of the 1,2-carbonyl addition intermediate was complete as monitored by TLC for a total reaction time of 19 hours. After cooling to 0 °C, the reaction was quenched with a saturated NH_4Cl solution (8 mL) and diluted with DI water (8 mL). The aqueous layer was extracted with CH_2Cl_2 (3 x 10 mL), and the combined organic layers were dried over Na_2SO_4, and concentrated under vacuum. The crude material was purified by column chromatography using 100% CH_2Cl_2 for ligand **7h** recovery followed by 85:15 hexanes : EtOAc to afford **(+)-6l** in 91% yield (98 mg, 0.38 mmol) as a yellow oil. ^{1}H NMR analysis of the crude reaction mixture indicated > 20:1 dr.

Rf: 0.3 in 80:20 hexanes : EtOAc

^{1}H NMR: (500 MHz, $CDCl_3$) δ = 5.10 (d, J = 11.4 Hz, 1H), 5.01 (d, J = 11.4 Hz, 1H), 4.04 (s, 1H), 2.23 (dq, J = 14.3, 3.0 Hz, 1H), 2.13 (dq, J = 13.0, 3.1 Hz, 1H), 1.89 (qt, J = 12.4, 3.1 Hz, 1H), 1.70 (dddd, J = 13.5, 6.6, 4.0, 4.0 Hz, 2H), 1.67 – 1.60, (m, 1H), 1.57 (s, 3H), 1.37 (s, 3H).

^{13}C NMR: (125 MHz, $CDCl_3$) δ = 212.6, 203.3, 96.5, 83.5, 53.2, 42.4, 39.3, 29.7, 26.3, 25.5, 19.2.

IR: f (cm^{-1}) = 3502, 2975, 2933, 2870, 1703, 1430, 1373, 1011, 708.

HRMS (ESI-TOF): m/z $[M+H]^+$ = 259.0328 calculated for $C_{11}H_{16}BrO_2$; found 259.0335.

Specific Rotation: $[\alpha]_{25}^{D}$ = +102 (c = 1.0 in $CHCl_3$)

HPLC ((+)-6l)-BzNO$_2$: IC-3, hexane/isopropanol = 92.5/7.5, flow rate = 1.0 mL/min, λ = 254 nm, t_R = 17.5 min (minor), 26.8 min (major).

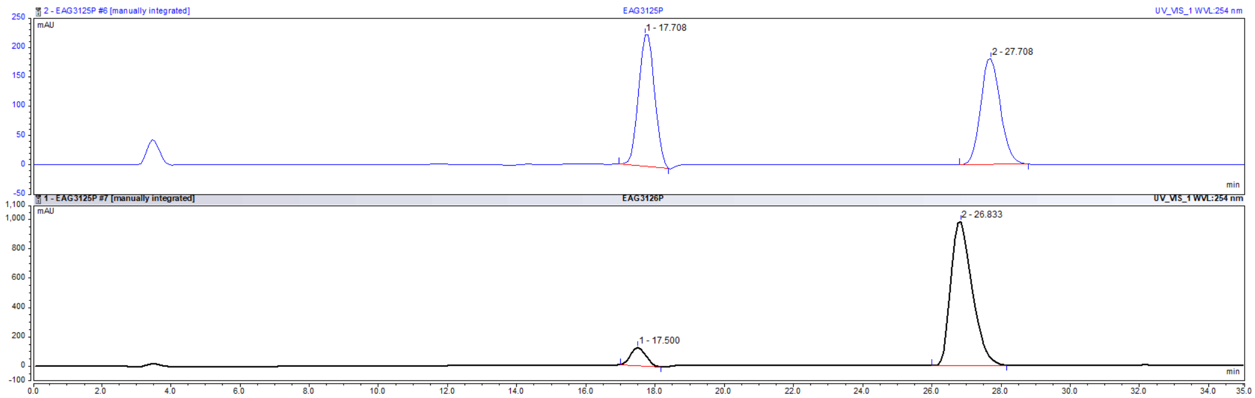

Retention Time (min)	Relative Area (%)	Area (mAU*min)	Height (mAU)
17.500	8.22	63.1328	123.84
26.833	91.78	705.1140	982.47

X-Ray Crystallography: The ellipsoid contour was set at a 50% probability level.

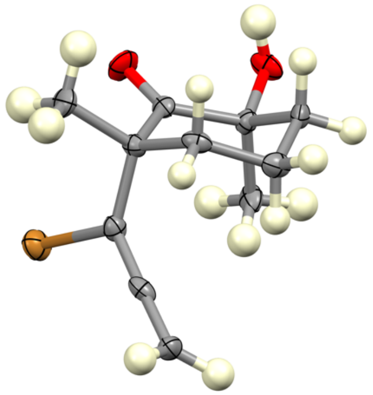

Compound ((+)-6l)-BzNO$_2$

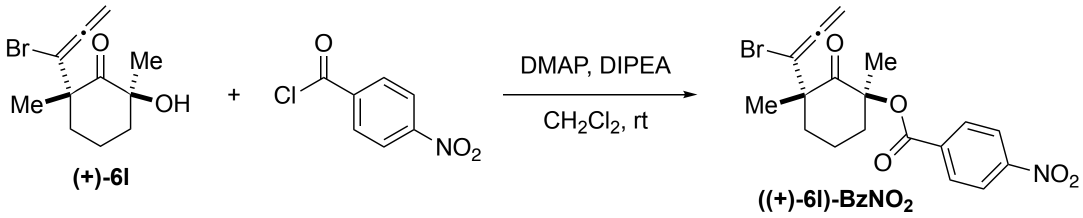

Procedure: Alcohol **(+)-6l** (35 mg, 0.14 mmol) was dissolved in CH_2Cl_2 (0.7 mL). *N*,*N*-Diisopropylethylamine (118 µL, 0.675 mmol) was then added to the solution, followed by 4-

nitrobenzoyl chloride (80 mg, 0.43 mmol) and 4-(dimethylamino)pyridine (17 mg, 0.14 mmol). The reaction mixture was then stirred at room temperature until complete consumption of the starting material as monitored by TLC. The crude reaction mixture was concentrated under vacuum and then purified by column chromatography using 94:6 hexanes : EtOAc to afford compound **((+)-6l)-BzNO$_2$** in 97% yield (54 mg, 0.13 mmol) as a yellow solid.

Rf: 0.5 in 80:20 hexanes : EtOAc

^{1}H NMR: (400 MHz, CDCl$_3$) δ = 8.26 (dt, *J* = 9.0, 2.2 Hz, 2H), 8.17 (dt, *J* = 9.0, 2.1 Hz, 2H), 5.00 (d, *J* = 11.4 Hz, 1H), 4.91 (d, *J* = 11.3 Hz, 1H), 2.62 (td, *J* = 12.9, 5.2 Hz, 1H), 2.28 (dddd, *J* = 13.8, 4.8, 2.5, 2.5 Hz, 1H), 2.07 (dtd, *J* = 12.9, 3.6, 2.4 Hz, 1H), 1.94 (ddd, *J* = 12.7, 12.7, 3.3 Hz, 1H) 1.94 – 1.84 (m, 1H), 1.84 – 1.76 (m, 1H), 1.72 (s, 3H), 1.42 (s, 3H).

^{13}C NMR: (100 MHz, CDCl$_3$) δ = 203.9, 203.8, 163.3, 150.5, 135.8, 130.8, 123.4, 97.2, 84.6, 83.0, 54.1, 37.6, 36.4, 25.4, 25.3, 19.1.

IR: *f* (cm^{-1}) = 3112, 3078, 2937, 2870, 1713, 1526, 1451, 1281, 718.

HRMS (ESI-TOF): *m/z* $[M+H]^+$ = 408.0441 calculated for $C_{18}H_{19}BrNO_5$; found 408.0451.

Compound (±)-6l

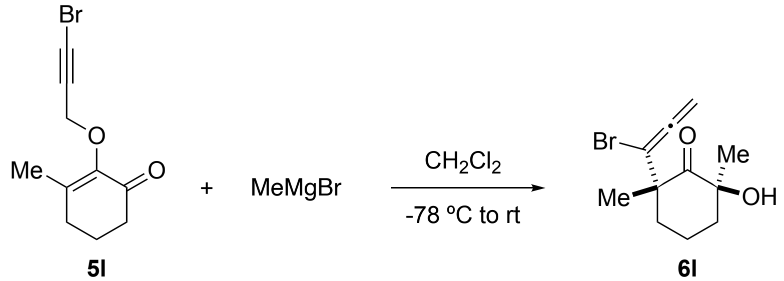

Procedure: Ketone **5l** (104 mg, 0.427 mmol) and CH_2Cl_2 (2.1 mL) were added into a round bottom flask. After cooling the solution to -78 ºC, methylmagnesium bromide (0.16 mL, 0.47 mmol, 3.0 M in Et_2O) was added dropwise. The reaction mixture was allowed to warm to room temperature

and stirred for 20 hours, at which the Claisen rearrangement of the 1,2-carbonyl addition intermediate was complete as monitored by TLC. After cooling to 0 °C, the reaction was quenched with saturated NH_4Cl (5 mL), and the resulting mixture was extracted with CH_2Cl_2 (3 x 5 mL). The organic layers were combined, dried over Na_2SO_4, filtered, and then concentrated under vacuum. The crude material was purified with column chromatography using 87.5:12.5 hexanes : EtOAc to yield **6l** in 60% yield (66 mg, 0.26 mmol) as a dark oil. ^{1}H NMR analysis of the crude reaction mixture indicated > 20:1 dr.

^{1}H NMR: (400 MHz, $CDCl_3$) δ = 5.07 (d, *J* = 11.4 Hz, 1H), 4.98 (d, *J* = 11.4 Hz, 1H), 3.96 (s, 1H), 2.25 (dq, *J* = 14.1, 2.8 Hz, 1H), 2.12 (dq, *J* = 13.0, 3.1 Hz, 1H), 1.97 – 1.83 (m, 1H), 1.76 – 1.59 (m, 3H), 1.56 (s, 3H), 1.37 (s, 3H).

^{13}C NMR: (100 MHz, $CDCl_3$) δ = 212.4, 203.5, 96.7, 83.2, 77.2, 53.3, 42.4, 39.4, 26.3, 25.6, 19.2.

Compound (+)-6m

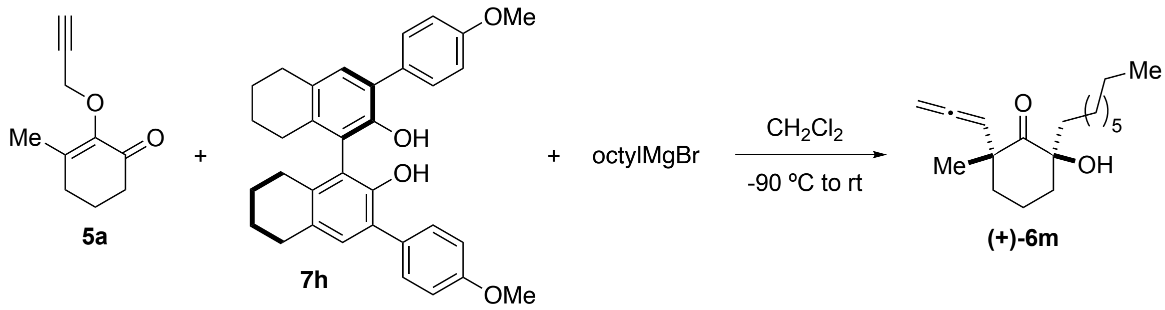

Procedure: Ligand **7h** (434 mg, 0.857 mmol) and ketone **5a** (101 mg, 0.612 mmol) were dissolved in CH_2Cl_2 (12 mL) in a round bottom flask. The solution was cooled to -90 °C, followed by the addition of octylmagnesium bromide (1.2 mL, 2.39 mmol, 2.0 M in Et_2O) dropwise. After stirring at -90 °C until the starting material was fully consumed as monitored by TLC, the reaction mixture was warmed to room temperature and stirred until the Claisen rearrangement of the 1,2-carbonyl addition intermediate was complete as monitored by TLC for a total reaction time of 25 hours.

After cooling to 0 °C, the reaction was quenched with a saturated NH_4Cl solution (8 mL) and diluted with DI water (8 mL). The aqueous layer was extracted with CH_2Cl_2 (3 x 10 mL), and the combined organic layers were dried over Na_2SO_4, and concentrated under vacuum. The crude material was purified by column chromatography using 100% CH_2Cl_2 for ligand **7h** recovery followed by 98:2 hexanes : EtOAc to afford **(+)-6m** in 64% yield (109 mg, 0.391 mmol) as a yellow oil. ^{1}H NMR analysis of the crude reaction mixture indicated > 20:1 dr.

Rf: 0.8 in 80:20 hexanes : EtOAc

^{1}H NMR: (400 MHz, $CDCl_3$) δ = 5.15 (t, *J* = 6.7, 1H), 4.95 (dd, *J* = 11.2, 6.8 Hz, 1H), 4.87 (dd, *J* = 11.1, 6.5 Hz, 1H), 3.96 (s, 1H), 2.18 – 2.08 (m, 2H), 1.97 – 1.85 (m, 2H), 1.71 – 1.58 (m, 4H), 1.46 – 1.36 (m, 1H), 1.29 – 1.20 (m, 12H), 0.97 – 0.89 (m, 1H), 0.87 (t, *J* = 6.5 Hz, 3H).

^{13}C NMR: (100 MHz, $CDCl_3$) δ = 215.1, 207.2, 94.3, 79.1, 78.7, 47.4, 40.3, 39.2, 39.0, 31.9, 29.8, 29.4, 29.2, 26.2, 22.9, 22.7, 19.0, 14.1.

IR: *f* (cm^{-1}) = 2926, 2854, 1953, 1711, 1608, 1527, 1456, 1409, 1280, 1112, 1101.

HRMS (ESI-TOF): *m/z* [M+H]+ = 279.2334 calculated for $C_{18}H_{31}O_2$; found 279.2323.

Specific Rotation: $[\alpha]_{25}^{D}$ = +19.2 (c = 1.0 in $CHCl_3$).

HPLC ((+)-6m)-BzNO$_2$: (S,S)-Whelk-O1, hexane/isopropanol = 99.0/1.0, flow rate = 1.0 mL/min, λ = 254 nm, t_R = 12.1 min (major), 14.8 min (minor).

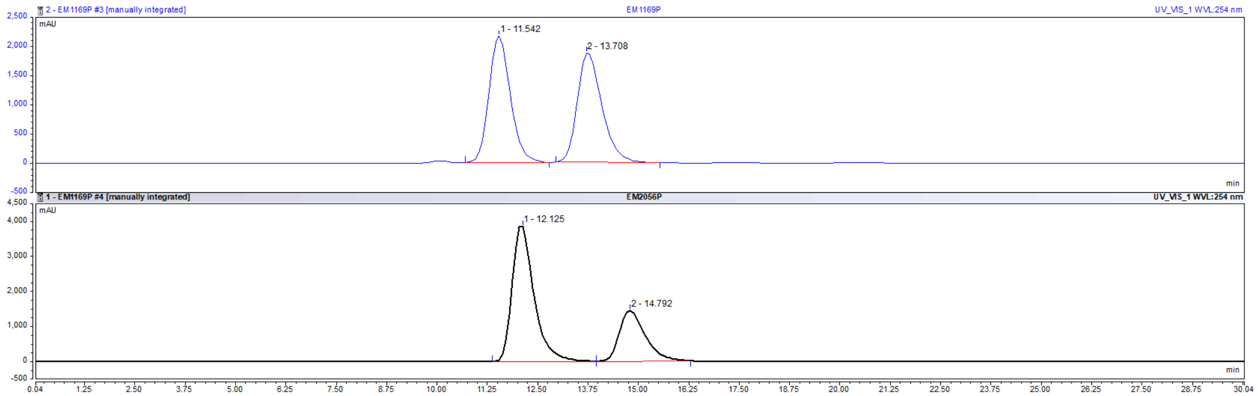

Retention Time (min)	Relative Area (%)	Area (mAU*min)	Height (mAU)
12.125	69.48	2430.7401	3847.27
14.792	30.52	1067.5137	1443.63

Compound ((+)-6m)-BzNO$_2$

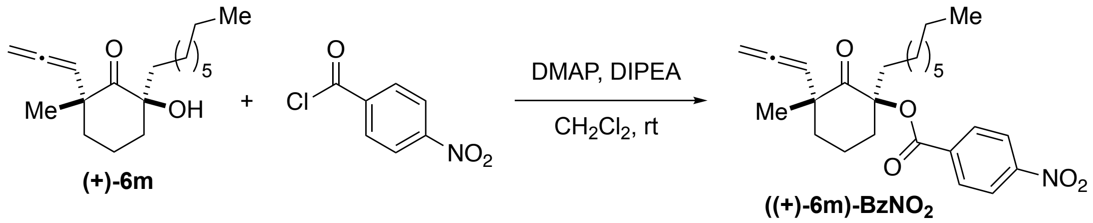

Procedure: Alcohol **(+)-6m** (39 mg, 0.14 mmol) was dissolved in CH_2Cl_2 (0.5 mL). *N,N*-Diisopropylethylamine (121 μL, 0.693 mmol) was then added to the solution, followed by 4-nitrobenzoyl chloride (82 mg, 0.44 mmol) and 4-(dimethylamino)pyridine (17 mg, 0.14 mmol). The reaction mixture was then stirred at room temperature until complete consumption of the starting material as monitored by TLC. The crude reaction mixture was concentrated under vacuum and then purified by column chromatography using 95:5 hexanes : EtOAc to afford compound **((+)-6m)-BzNO$_2$** in 74% yield (32 mg, 0.070 mmol) as a yellow oil.

Rf: 0.6 in 80:20 hexanes : EtOAc

^{1}H NMR: (400 MHz, $CDCl_3$) δ = 8.27 (dt, *J* = 9.1, 2.2 Hz, 2H), 8.15 (dt, *J* = 9.1, 2.2 Hz, 2H), 5.11 (t, *J* = 6.6 Hz, 1H), 4.91 (qd, *J* = 11.0, 6.8 Hz, 2H), 2.58 (td, *J* = 12.6, 4.6 Hz, 1H), 2.17 – 1.74 (m, 7H), 1.35 – 1.24 (m, 15H), 0.90 (t, *J* = 5.3 Hz, 3H).

^{13}C NMR: (100 MHz, $CDCl_3$) δ = 207.2, 207.0, 163.4, 150.5, 136.1, 130.7, 123.5, 95.9, 86.2, 78.7, 48.6, 37.6, 36.0, 34.6, 31.8, 29.7, 29.4, 29.2, 26.6, 23.0, 22.7, 19.1, 14.1.

IR: *ƒ* (cm^{-1}) = 2987, 2955, 2945, 2930, 1950, 1727, 1710, 1601, 1525, 1283, 892, 839, 716, 418.

HRMS (ESI-TOF): *m/z* [M+H]+ = 428.2431 calculated for $C_{25}H_{34}NO_5$; found 428.2445.

Compound (±)-6m

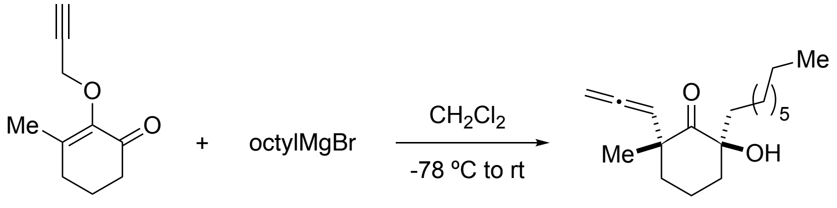

Procedure: Ketone **5a** (152 mg, 0.928 mmol) and CH_2Cl_2 (4.6 mL) were added into a round bottom flask. After cooling the solution to -78 ºC, octylmagnesium bromide (0.60 mL, 1.21 mmol, 2.0 M in Et_2O) was added dropwise. The reaction mixture was allowed to warm to room temperature and stirred for 25 hours, at which the Claisen rearrangement of the 1,2-carbonyl addition intermediate was complete as monitored by TLC. After cooling to 0 °C, the reaction was quenched with saturated NH_4Cl (5 mL), and the resulting mixture was extracted with CH_2Cl_2 (3 x 5 mL). The organic layers were combined, dried over Na_2SO_4, filtered, and then concentrated under vacuum. The crude material was purified with column chromatography using 98:2 hexanes : EtOAc to yield **6b** in 80% yield (206 mg, 0.740 mmol) as a colorless oil. ^{1}H NMR analysis of the crude reaction mixture indicated > 20:1 dr.

Rf: 0.8 in 80:20 Hexanes : EtOAc

^{1}H NMR: (500 MHZ, $CDCl_3$) δ = 5.14 (t, *J* = 6.7 Hz, 1H), 4.94 (dd, *J* = 11.1, 6.8 Hz, 1H), 4.86 (dd, *J* = 11.1, 6.6 Hz, 1H), 3.96 (s, 1H), 2.12 (ddq, *J* = 15.2, 10.5, 2.4 Hz, 2H), 1.97 – 1.84 (m, 2H), 1.69 – 1.54 (m, 4H), 1.42 – 1.35 (m, 1H), 1.27 – 1.17 (m, 13H), 0.94 – 0.88 (m, 1H), 0.86, (t, *J* = 5.4 Hz, 3H).

^{13}C NMR: (125 MHz, $CDCl_3$) δ = 215.0, 207.2, 94.3, 79.1, 78.7, 47.4, 40.3, 39.2, 39.0, 31.8, 29.8, 29.4, 29.2, 26.1, 22.9, 22.6, 18.9, 14.1.

Compound (±)-6n

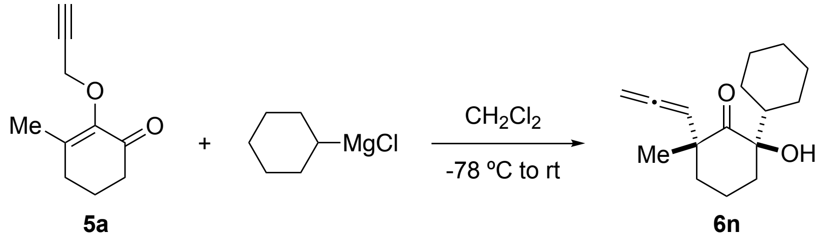

Procedure: Ketone **5a** (88 mg, 0.54 mmol) and CH_2Cl_2 (2.7 mL) were added into a round bottom flask. After cooling the solution to -78 ºC, cyclohexylmagnesium chloride (0.35 mL, 0.70 mmol, 2.0 M in Et_2O) was added dropwise. The reaction mixture was allowed to warm to room temperature and stirred for 26 hours, at which the Claisen rearrangement of the 1,2-carbonyl addition intermediate was complete as monitored by TLC. After cooling to 0 °C, the reaction was quenched with saturated NH_4Cl (5 mL), and the resulting mixture was extracted with CH_2Cl_2 (3 x 5 mL). The organic layers were combined, dried over Na_2SO_4, filtered, and then concentrated under vacuum. The crude material was purified with column chromatography using 95:5 hexanes : EtOAc to yield **6n** in 76% yield (101 mg, 0.407 mmol) as a colorless oil. ^{1}H NMR analysis of the crude reaction mixture indicated > 20:1 dr.

Rf: 0.7 in 80:20 Hexanes : EtOAc

^{1}H NMR: (400 MHz, $CDCl_3$) δ = 5.19 (td, *J* = 6.7, 0.6 Hz, 1H), 4.95 (dd, *J* = 11.1, 6.8 Hz, 1H), 4.87 (dd, *J* = 11.0, 6.6 Hz, 1H), 3.84 (s, 1H), 2.41 (dq, *J* = 13.8, 3.1 Hz, 1H), 2.17 – 2.09 (m, 1H), 2.04 – 1.95 (m, 1H), 1.90 – 1.75 (m, 3H), 1.74 – 1.67 (m, 1H), 1.65 – 1.52 (m, 3H), 1.41 (td, *J* = 13.8, 3.6 Hz, 1H), 1.30 – 1.07 (m, 9H).

^{13}C NMR: (100 MHz, $CDCl_3$) δ = 215.3, 207.2, 94.3, 81.5, 76.7, 47.6, 41.3, 39.6, 36.5, 26.8, 26.7, 26.5, 26.3, 26.2, 25.0, 18.4.

IR: *f* (cm^{-1}) = 3479, 3059, 2935, 2871, 1729, 1371, 1225, 1023, 752.

HRMS (ESI-TOF): *m/z* [M+H]+ = 249.1849 calculated for $C_{16}H_{25}O_2$; found 249.1857.

Compound (±)-6o

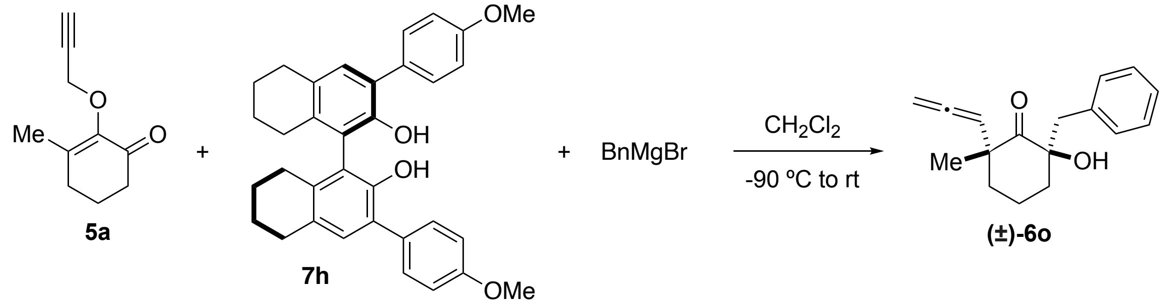

Procedure: Ligand **7h** (455mg, 0.899 mmol) and ketone **5a** (105 mg, 0.642 mmol) were dissolved in CH_2Cl_2 (13 mL) in a round bottom flask. The solution was cooled to -90 °C, followed by the addition of benzylmagnesium chloride (2.5 mL, 2.5 mmol, 1.0 M in Et_2O) dropwise. After stirring at -90 °C until the starting material was fully consumed as monitored by TLC, the reaction mixture was warmed to room temperature and stirred until the Claisen rearrangement of the 1,2-carbonyl addition intermediate was complete as monitored by TLC for a total reaction time of 42 hours. After cooling to 0 °C, the reaction was quenched with a saturated NH_4Cl solution (8 mL) and diluted with DI water (8 mL). The aqueous layer was extracted with CH_2Cl_2 (3 x 10 mL), and the combined organic layers were dried over Na_2SO_4, and concentrated under vacuum. The crude material was purified by column chromatography using 100% CH_2Cl_2 for ligand **7h** recovery followed by 95:5 hexanes : EtOAc to afford **(±)-6o** in 21% yield (34 mg, 0.13 mmol) as a colorless oil. ^{1}H NMR analysis of the crude reaction mixture indicated > 20:1 dr.

Rf: 0.7 in 80:20 hexanes : EtOAc

^{1}H NMR: (400 MHz, $CDCl_3$) δ = 7.24 – 7.13 (m, 5H), 5.18 (td, *J* = 6.7, 0.8 Hz, 1H), 4.96 (dd, *J* = 11.2, 6.8 Hz, 1H), 4.87 (dd, *J* = 11.2, 6.5 Hz, 1H), 3.12 (d, *J* = 13.9 Hz, 1H), 2.83 (d, *J* = 13.9 Hz, 1H), 2.18 – 2.09 (m, 1H), 2.07 – 1.99 (m, 1H), 1.98 – 1.87 (m, 1H), 1.65 – 1.50 (m, 3H), 1.19 (s, 3H).

^{13}C NMR: (400 MHz, $CDCl_3$) δ = 214.8, 207.2, 135.6, 130.7, 128.0, 126.8, 95.8, 79.0, 78.6, 47.6, 44.6, 38.6, 38.0, 26.6, 18.7.

IR: *f* (cm^{-1}) = 3484, 3062, 3028, 2932, 2868, 1701, 1454, 1376, 1001, 701.

HRMS (ESI-TOF): *m/z* [M+H]+ = 257.1536 calculated for $C_{17}H_{21}O_2$; found 257.1544.

HPLC ((±)-6m)-BzNO$_2$: (S,S)-Whelk-O1, hexane/isopropanol = 97.5/2.5, flow rate = 1.0 mL/min, λ = 254 nm, t_R = 14.0 min, 20.0 min.

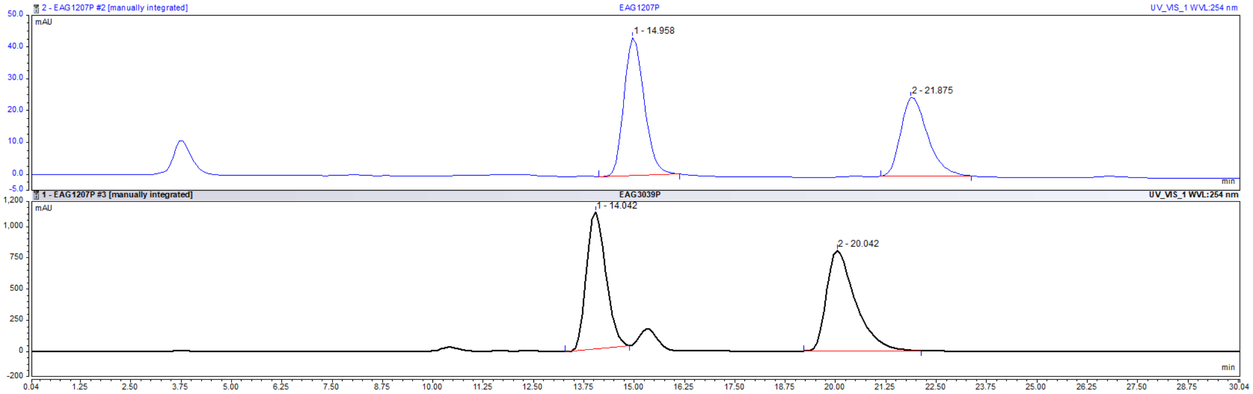

Retention Time (min)	Relative Area (%)	Area (mAU*min)	Height (mAU)
14.042	47.56	607.6113	1091.75
20.042	52.44	669.8804	804.69

Compound ((±)-6o)-BzNO$_2$

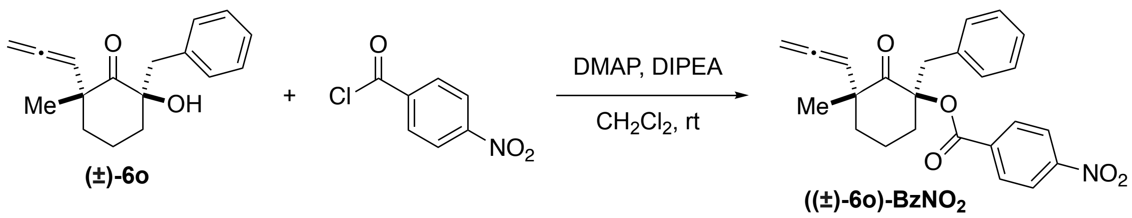

Procedure: Alcohol **(±)-6o** (34 mg, 0.13 mmol) was dissolved in CH_2Cl_2 (0.7 mL). *N*,*N*-Diisopropylethylamine (115 µL, 0.661 mmol) was then added to the solution, followed by 4-nitrobenzoyl chloride (79 mg, 0.42 mmol) and 4-(dimethylamino)pyridine (16 mg, 0.13 mmol).

The reaction mixture was then stirred at room temperature until complete consumption of the starting material as monitored by TLC. The crude reaction mixture was concentrated under vacuum and then purified by column chromatography using 95:5 hexanes : EtOAc to afford compound **((±)-6o)-BzNO$_2$** in 79% yield (42 mg, 0.10 mmol) as a yellow solid.

Rf: 0.7 in 80:20 hexanes : EtOAc

^{1}H NMR: (400 MHz, $CDCl_3$) δ = 8.28 (dt, *J* = 9.2, 2.5 Hz, 2H), 8.13 (dt, *J* = 9.2, 2.4 Hz, 2H), 7.42 – 7.29 (m, 5H), 5.21 (t, *J* = 6.7 Hz, 1H), 4.96 (qd, *J* = 11.1, 6.8 Hz, 2H), 3.24 (d, *J* = 14.4, Hz, 1H), 3.16 (d, *J* = 14.4 Hz, 1H), 2.36 – 2.26 (m, 1H), 2.17 – 1.93 (m, 4H), 1.86 – 1.76 (m, 1H), 1.40 (s, 3H).

^{13}C NMR: (100 MHz, $CDCl_3$) δ = 208.3, 206.9, 163.5, 150.5, 135.8, 135.0, 131.0, 130.7, 128.3, 127.3, 123.5, 96.9, 85.1, 78.8, 49.0, 42.6, 35.9, 32.2, 26.8, 18.9.

IR: *f* (cm^{-1}) = 3111, 3085, 3029, 2931, 2868, 1709, 1526, 1454, 1348, 719.

HRMS (ESI-TOF): *m/z* [M+H]+ = 406.1649 calculated for $C_{24}H_{24}NO_5$; found 406.1644.

Compound (±)-6o

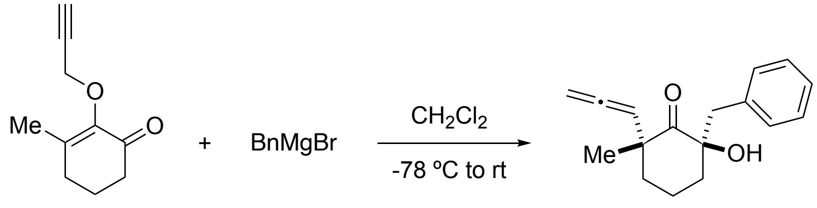

5a **6o**

Procedure: Ketone **5a** (102 mg, 0.619 mmol) and CH_2Cl_2 (3.1 mL) were added into a round bottom flask. After cooling the solution to -78 ºC, benzylmagnesium bromide (0.58 mL, 0.805 mmol, 1.4 M in THF) was added dropwise. The reaction mixture was allowed to warm to reflux in an oil bath and stirred for 99 hours, at which the Claisen rearrangement of the 1,2-carbonyl addition intermediate was complete as monitored by TLC. After cooling to 0 °C, the reaction was

quenched with saturated NH_4Cl (5 mL), and the resulting mixture was extracted with CH_2Cl_2 (3 x 5 mL). The organic layers were combined, dried over Na_2SO_4, filtered, and then concentrated under vacuum. The crude material was purified with column chromatography using 92:8 hexanes : EtOAc to yield **6o** in 55% yield (88 mg, 0.34 mmol) as a cloudy white oil. ^{1}H NMR analysis of the crude reaction mixture indicated > 20:1 dr.

^{1}H NMR: (400 MHz, $CDCl_3$) δ = 7.24 – 7.13 (m, 5H), 5.18 (td, *J* = 6.7, 0.8 Hz, 1H), 4.96 (dd, *J* = 11.2, 6.8 Hz, 1H), 4.87 (dd, *J* = 11.2, 6.5 Hz, 1H), 3.12 (d, *J* = 13.9 Hz, 1H), 2.83 (d, *J* = 13.9 Hz, 1H), 2.18 – 2.09 (m, 1H), 2.07 – 1.99 (m, 1H), 1.98 – 1.87 (m, 1H), 1.65 – 1.50 (m, 3H), 1.19 (s, 3H).

^{13}C NMR: (400 MHz, $CDCl_3$) δ = 214.8, 207.2, 135.6, 130.7, 128.0, 126.8, 95.8, 79.0, 78.6, 47.6, 44.6, 38.6, 38.0, 26.6, 18.7.

Compound (±)-6p

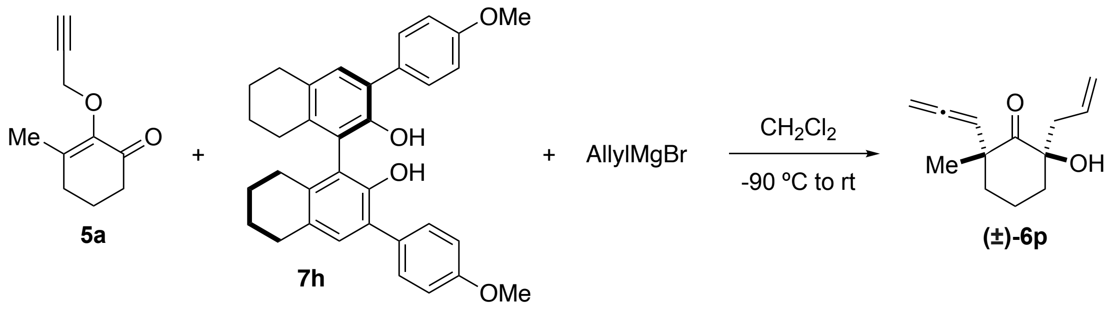

Procedure: Ligand **7h** (459 mg, 0.905 mmol) and ketone **5a** (106 mg, 0.647 mmol) were dissolved in CH_2Cl_2 (13 mL) in a round bottom flask. The solution was cooled to -90 °C, followed by the addition of allylmagnesium bromide (2.5 mL, 2.5 mmol, 1.0 M in Et_2O) dropwise. After stirring at -90 °C until the starting material was fully consumed as monitored by TLC, the reaction mixture was warmed to room temperature and stirred until the Claisen rearrangement of the 1,2-carbonyl addition intermediate was complete as monitored by TLC for a total reaction time of 17 hours.

After cooling to 0 °C, the reaction was quenched with a saturated NH_4Cl solution (8 mL) and diluted with DI water (8 mL). The aqueous layer was extracted with CH_2Cl_2 (3 x 10 mL), and the combined organic layers were dried over Na_2SO_4, and concentrated under vacuum. The crude material was purified by column chromatography using 100% CH_2Cl_2 to afford **(±)-6p** in 33% yield (43 mg, 0.21 mmol) as a colorless oil. ^{1}H NMR analysis of the crude reaction mixture indicated > 20:1 dr.

Rf: 0.7 in 80:20 hexanes : EtOAc

^{1}H NMR: (400 MHz, $CDCl_3$) δ = 5.67 (dddd, *J* = 16.9, 10.4, 7.7, 6.4 Hz, 1H), 5.18 – 5.06 (m, 3H), 4.97 (dd, *J* = 11.2, 6.8 Hz, 1H), 4.89 (dd, *J* = 11.2, 6.6 Hz, 1H), 3.84 (s, 1H), 2.66 (dt, *J* = 6.4, 1.4 Hz, 1H), 2.45 (dt, *J* = 7.6, 1.2 Hz, 1H), 2.15 (ddq, *J* = 18.7, 12.9, 2.8 Hz, 2H), 1.96 – 1.82 (m, 1H), 1.75 – 1.56 (m, 3H), 1.22 (s, 3H).

^{13}C NMR: (100 MHz, $CDCl_3$) δ = 214.3, 207.1, 132.2, 119.1, 94.7, 78.9, 78.2, 47.5, 43.7, 39.2, 38.5, 26.3, 18.7.

IR: *f* (cm^{-1}) = 3486, 3076, 2976, 2932, 2866, 1699, 1639, 1453, 1374, 1004.

HRMS (ESI-TOF): *m/z* [M+H]+ = 207.1380 calculated for $C_{13}H_{19}O_2$; found 207.1389.

HPLC ((±)-6p)-BzNO$_2$: (S,S)-Whelk-O1, hexane/isopropanol = 97.5/2.5, flow rate = 1.0 mL/min, λ = 254 nm, t_R = 10.9 min, 13.3 min.

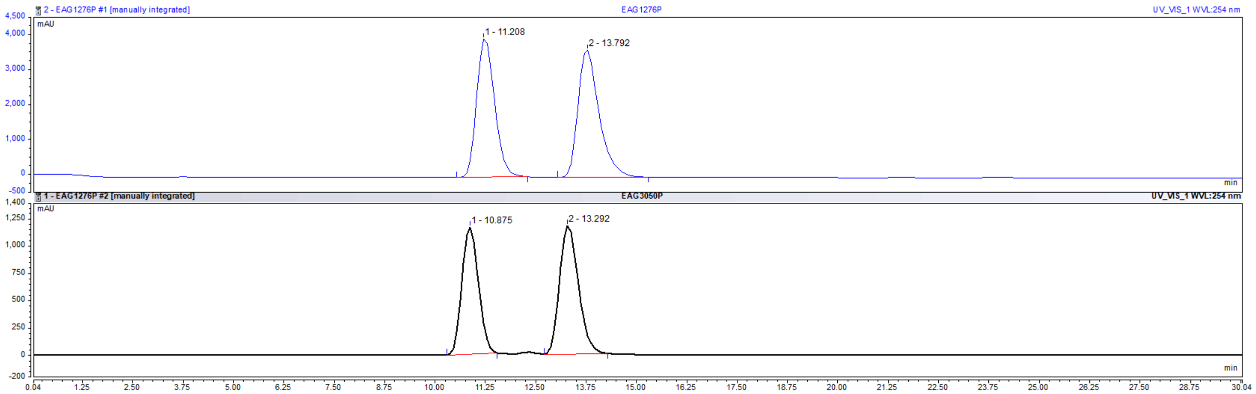

Retention Time (min)	Relative Area (%)	Area (mAU*min)	Height (mAU)
14.042	47.56	607.6113	1091.75
20.042	52.44	669.8804	804.69

Compound ((±)-6p)-BzNO$_2$

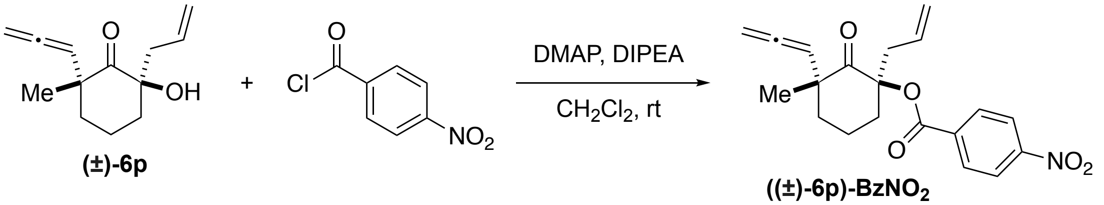

Procedure: Alcohol **(±)-6p** (37 mg, 0.18 mmol) was dissolved in CH_2Cl_2 (0.9 mL). *N*,*N*-Diisopropylethylamine (158 µL, 0.907 mmol) was then added to the solution, followed by 4-nitrobenzoyl chloride (108 mg, 0.580 mmol) and 4-(dimethylamino)pyridine (22 mg, 0.18 mmol). The reaction mixture was then stirred at room temperature until complete consumption of the starting material as monitored by TLC. The crude reaction mixture was concentrated under vacuum and then purified by column chromatography using 94:6 hexanes : EtOAc to afford compound **((±)-6p)-BzNO$_2$** in 78% yield (50 mg, 0.14 mmol) as a colorless oil.

Rf: 0.6 in 80:20 hexanes : EtOAc

^{1}H NMR: (400 MHz, $CDCl_3$) δ = 8.26 (dt, *J* = 9.0, 2.2 Hz, 2H), 8.15 (dt, *J* = 9.1, 2.2 Hz, 2H), 5.90 (dddd, *J* = 16.8, 10.2, 7.6, 6.4 Hz, 1H), 5.26 – 5.17 (m, 2H), 5.11 (t, *J* = 6.7 Hz, 1H), 4.91 (qd, *J* = 11.0, 6.8 Hz, 2H), 2.72 (qdt, *J* = 14.7, 6.5, 1.9 Hz, 2H), 2.51 (td, *J* = 13.0, 4.8 Hz, 1H), 2.17 (dq, *J* = 13.0, 3.6 Hz, 1H), 2.10 – 2.04 (m, 1H), 2.01 – 1.82 (m, 2H), 1.81 – 1.73 (m, 1H), 1.34 (s, 3H).

^{13}C NMR: (100 MHz, $CDCl_3$) δ = 207.1, 206.9, 163.2, 150.5, 135.8, 131.5, 130.7, 123.4, 119.6, 96.1, 85.2, 78.8, 48.7, 41.7, 35.9, 33.8, 26.6, 18.8.

IR: *f* (cm^{-1}) = 3078, 2965, 2932, 2875, 1712, 1527, 1455, 1285, 858, 718.

HRMS (ESI-TOF): *m/z* [M+H]+ = 356.1492 calculated for $C_{20}H_{22}NO_5$; found 356.1462.

Compound (±)-6p

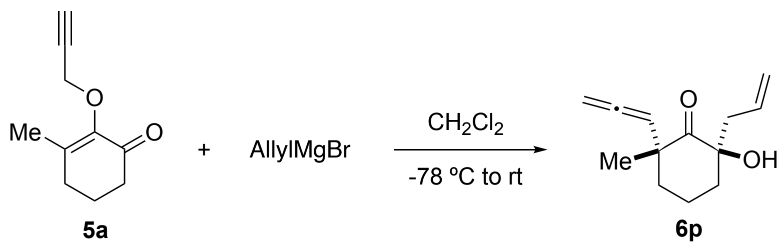

Procedure: Ketone **5a** (153 mg, 0.934 mmol) and CH_2Cl_2 (4.7 mL) were added into a round bottom flask. After cooling the solution to -78 ºC, allylmagnesium bromide (1.2 mL, 1.2 mmol, 1.0 M in Et_2O) was added dropwise. The reaction mixture was allowed to warm to room temperature and stirred for 26 hours, at which the Claisen rearrangement of the 1,2-carbonyl addition intermediate was complete as monitored by TLC. After cooling to 0 °C, the reaction was quenched with saturated NH_4Cl (5 mL), and the resulting mixture was extracted with CH_2Cl_2 (3 x 5 mL). The organic layers were combined, dried over Na_2SO_4, filtered, and then concentrated under vacuum. The crude material was purified with column chromatography using 95:5 hexanes : EtOAc to yield **6p** in 84% yield (162 mg, 0.784 mmol) as a colorless oil. ^{1}H NMR analysis of the crude reaction mixture indicated > 20:1 dr.

Rf: 0.6 in 80:20 Hexanes : EtOAc

^{1}H NMR: (400 MHz, $CDCl_3$) δ = 5.67 (dddd, *J* = 16.9, 10.4, 7.7, 6.4 Hz, 1H), 5.18 – 5.06 (m, 3H), 4.97 (dd, *J* = 11.2, 6.8 Hz, 1H), 4.89 (dd, *J* = 11.2, 6.6 Hz, 1H), 3.84 (s, 1H), 2.66 (dt, *J* = 6.4, 1.4 Hz, 1H), 2.45 (dt, *J* = 7.6, 1.2 Hz, 1H), 2.15 (ddq, *J* = 18.7, 12.9, 2.8 Hz, 2H), 1.96 – 1.82 (m, 1H), 1.75 – 1.56 (m, 3H), 1.22 (s, 3H).

^{13}C NMR: (100 MHz, $CDCl_3$) δ = 214.3, 207.1, 132.2, 119.1, 94.7, 78.9, 78.2, 47.5, 43.7, 39.2, 38.5, 26.3, 18.7.

Compound (±)-6q

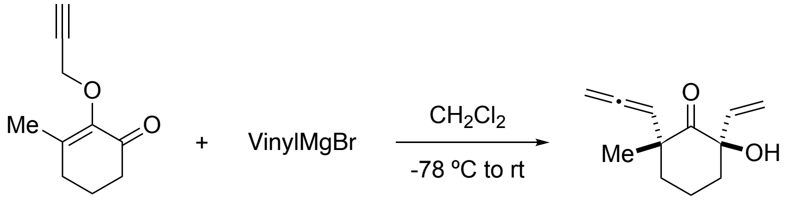

5a **6q**

Procedure: Ketone **5a** (150 mg, 0.914 mmol) and CH_2Cl_2 (4.6 mL) were added into a round bottom flask. After cooling the solution to -78 ºC, vinylmagnesium bromide (1.57 mL, 1.10 mmol, 0.7 M in THF) was added dropwise. The reaction mixture was allowed to warm to room temperature and stirred for 25 hours, at which the Claisen rearrangement of the 1,2-carbonyl addition intermediate was complete as monitored by TLC. After cooling to 0 °C, the reaction was quenched with saturated NH_4Cl (5 mL), and the resulting mixture was extracted with CH_2Cl_2 (3 x 5 mL). The organic layers were combined, dried over Na_2SO_4, filtered, and then concentrated under vacuum. The crude material was purified with column chromatography using 98:2 hexanes : EtOAc to yield **6q** in 70% yield (123 mg, 0.641 mmol) as a colorless oil. ^{1}H NMR analysis of the crude reaction mixture indicated > 20:1 dr.

Rf: 0.6 in 80:20 Hexanes : EtOAc

^{1}H NMR: (400 MHz, $CDCl_3$) δ = 6.20 (dd, *J* = 17.0, 10.5 Hz, 1H), 5.41 (dd, *J* = 17.0, 1.3 Hz, 1H), 5.14 (dd, *J* = 10.5, 1.3 Hz, 1H), 5.08 (td, *J* = 6.7, 1.0 Hz, 1H), 4.91 (dd, *J* = 11.1, 6.8 Hz, 1H), 4.83 (dd, *J* = 11.1, 6.6 Hz, 1H), 4.21 (s, 1H), 2.23 (dq, *J* = 13.0, 3.0 Hz, 1H), 2.12 (dq, *J* = 14.4, 4.2 Hz, 1H), 2.04– 1.92 (m, 1H),1.77 (dd, 13.2, 3.8 Hz,1H), 1.73 – 1.59 (m, 2H), 1.23 (s, 3H).

^{13}C NMR: (100 MHz, $CDCl_3$) δ = 212.4, 207.4, 138.1, 115.0, 95.0, 79.0, 78.7, 47.8, 40.1, 38.8, 26.0, 18.5.

IR: *f* (cm^{-1}) = 3484, 2932, 2870, 1703, 1452, 1375, 1247, 1157, 1087, 992.

HRMS (ESI-TOF): *m/z* [M+H]+ = 193.1223 calculated for $C_{12}H_{17}O_2$; found 193.1221.

Compound (±)-6r

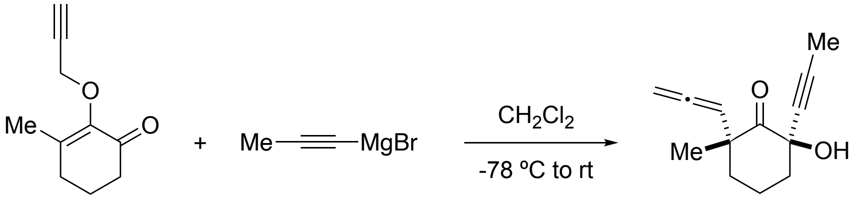

Procedure: Ketone **5a** (228 mg, 1.39 mmol) and CH_2Cl_2 (6.9 mL) were added into a round bottom flask. After cooling the solution to -78 ºC, 1-propynylmagnesium bromide (3.61 mL, 1.80 mmol, 0.5 M in THF) was added dropwise. The reaction mixture was allowed to warm to room temperature and stirred for 25 hours, at which the Claisen rearrangement of the 1,2-carbonyl addition intermediate was complete as monitored by TLC. After cooling to 0 °C, the reaction was quenched with saturated NH_4Cl (5 mL), and the resulting mixture was extracted with CH_2Cl_2 (3 x 5 mL). The organic layers were combined, dried over Na_2SO_4, filtered, and then concentrated under vacuum. The crude material was purified with column chromatography using 80:20 hexanes : EtOAc to yield **6r** in 41% yield (110 mg, 0.534 mmol) as a colorless oil. ^{1}H NMR analysis of the crude reaction mixture indicated > 20:1 dr.

Rf: 0.5 in 80:20 Hexanes : EtOAc

^{1}H NMR: (400 MHz, $CDCl_3$) δ = 5.55 (t, *J* = 6.6 Hz, 1H), 4.88 (qd, *J* = 10.8, 6.8 Hz, 2H), 4.24 (s, 1H), 2.39 (dq, *J* = 12.5, 3.2 Hz, 1H), 2.25 – 2.06 (m, 2H), 1.84 (s, 3H), 1.71 – 1.54 (m, 3H), 1.23 (s, 3H).

^{13}C NMR: (100 MHz, $CDCl_3$) δ = 209.5, 206.8, 96.3, 84.1, 79.5, 78.6, 72.4, 47.8, 41.8, 39.6, 25.2, 18.8, 3.7.

IR: *f* (cm^{-1}) = 3485, 3015, 2937, 2867, 1960, 1710, 1453, 1357, 1220, 1107, 1038, 993.

HRMS (ESI-TOF): *m/z* [M+H]+ = 205.1223 calculated for $C_{13}H_{17}O_2$; found 205.1225.

Compound (+)-6t

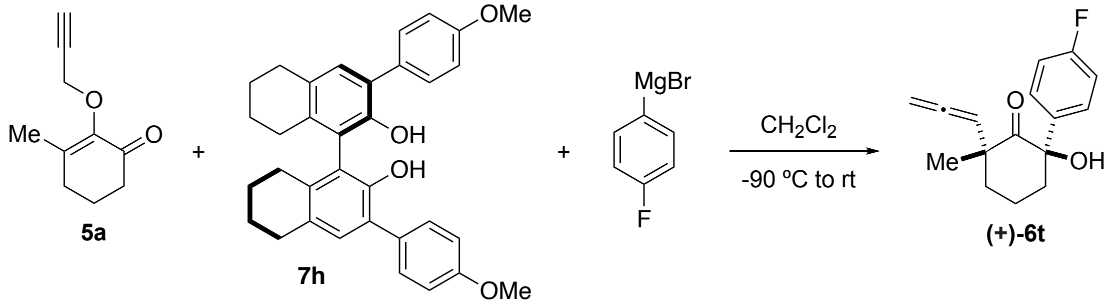

Procedure: Ligand **7h** (432 mg, 0.852 mmol) and ketone **5a** (100 mg, 0.609 mmol) were dissolved in CH_2Cl_2 (12 mL) in a round bottom flask. The solution was cooled to -90 °C, followed by the addition of 4-fluorophenylmagnesium bromide (1.2 mL, 2.38 mmol, 2.0 M in Et_2O) dropwise. After stirring at -90 °C until the starting material was fully consumed as monitored by TLC, the reaction mixture was warmed to reflux in an oil bath and stirred until the Claisen rearrangement of the 1,2-carbonyl addition intermediate was complete as monitored by TLC for a total reaction time of 25 hours. After cooling to 0 °C, the reaction was quenched with a saturated NH_4Cl solution (8 mL) and diluted with DI water (8 mL). The aqueous layer was extracted with CH_2Cl_2 (3 x 10 mL), and the combined organic layers were dried over Na_2SO_4, and concentrated under vacuum. The crude material was purified by column chromatography using 100% CH_2Cl_2 for ligand **7h** recovery followed by 80:20 hexanes : EtOAc to afford **(+)-6t** in 70% yield (110 mg, 0.426 mmol) as a yellow oil. ^{1}H NMR analysis of the crude reaction mixture indicated > 20:1 dr.

Rf: 0.5 in 80:20 hexanes : EtOAc

^{1}H NMR: (400 MHz, $CDCl_3$) δ = 7.32– 7.27 (m, 2H), 7.02 (ddd, *J* = 8.6, 6.7, 2.2 Hz, 2H), 4.68 (s, 1H), 4.62 – 4.56 (m, 3H), 2.92 (dq, *J* = 13.8, 3.0 Hz, 1H), 2.09 – 1.99 (m, 2H), 1.93 (ddd, *J* = 13.7, 13.7, 3.8 Hz, 1H), 1.84 – 1.75 (m, 1H), 1.74 – 1.65 (1H), 1.24 (s, 3H).

^{13}C NMR: (100 MHz, $CDCl_3$) δ = 213.5, 206.7, 163.6, 161.1, 136.3, 136.3, 128.1, 128.0, 115.6, 115.4, 94.9, 78.4, 78.2, 47.9, 39.1, 37.4, 25.2, 19.0.

IR: *f* (cm^{-1}) = 3476, 2931, 2872, 2855, 1954, 1700, 1602, 1509, 1462, 1452, 1229, 1095.

HRMS (ESI-TOF): *m/z* [M+H]+ = 261.1285 calculated for $C_{16}H_{18}FO_2$; found 261.1291.

Specific Rotation: $[\alpha]_{25}^{D}$ = +56.2 (c = 1.0 in $CHCl_3$)

HPLC ((+)-6t)-BzNO₂: (S,S)-Whelk-O1, hexane/isopropanol = 97.5/2.5, flow rate = 1.0 mL/min, λ = 254 nm, t_R = 15.9 min (major), 18.2 min (minor).

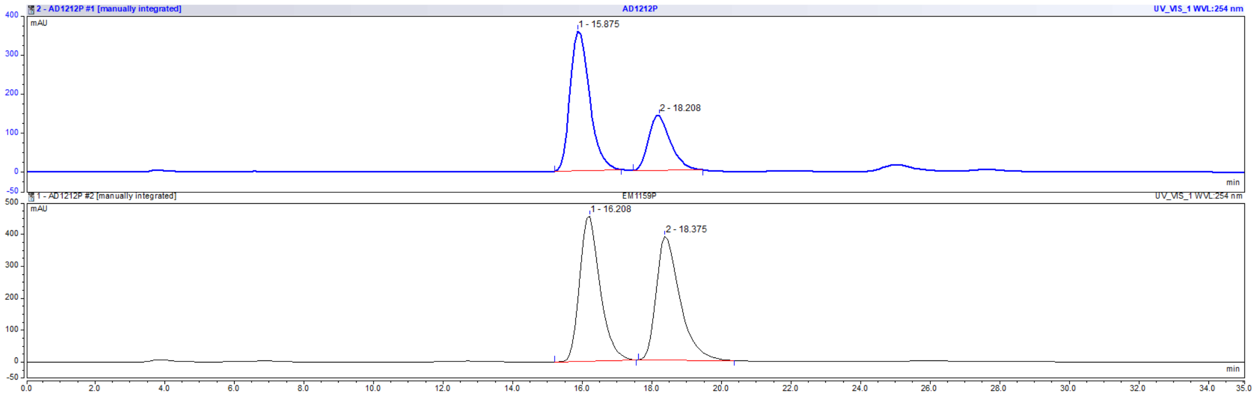

Retention Time (min)	Relative Area (%)	Area (mAU*min)	Height (mAU)
15.875	69.22	231.9013	357.43
18.208	30.78	103.1264	140.91

Compound ((+)- 6t)-BzNO₂

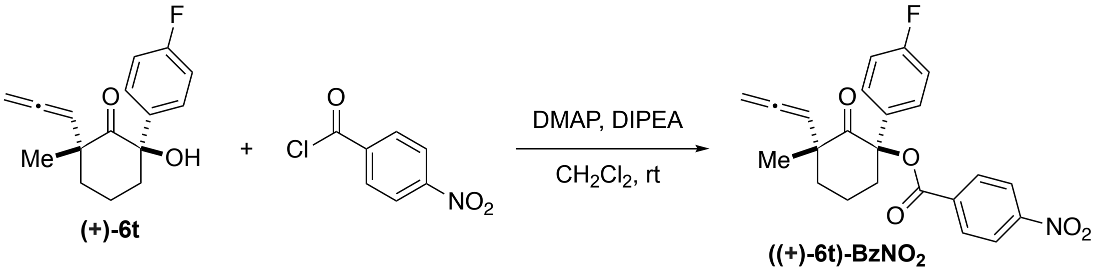

Procedure: Alcohol **(+)-6t** (64 mg, 0.25 mmol) was dissolved in CH_2Cl_2 (2.0 mL). *N*,*N*-Diisopropylethylamine (214 μL, 1.25 mmol) was then added to the solution, followed by 4-nitrobenzoyl chloride (137 mg, 0.737 mmol) and 4-(dimethylamino)pyridine (30 mg, 0.25 mmol). The reaction mixture was then stirred at room temperature until complete consumption of the

starting material as monitored by TLC. The crude reaction mixture was concentrated under vacuum and then purified by column chromatography using 90:10 hexanes : EtOAc to afford compound **((±)-6t)-BzNO$_2$** in 83% yield (83 mg, 0.20 mmol) as a white solid.

Rf: 0.6 in 80:20 (Hex:EtOAc)

^{1}H NMR: (400 MHz, $CDCl_3$) δ = 8.25 (dt, *J* = 8.9, 2.1 Hz, 2H), 8.14 (dt, *J* = 9.1, 2.1 Hz, 2H), 7.64 (ddd, *J* = 7.0, 5.2, 2.1 Hz, 2H), 7.13 – 7.06 (m, 2H), 4.67 (t, *J* = 6.7 Hz, 1H), 4.60 (dd, *J* = 10.9, 6.8 Hz, 1H), 4.47 (dd, *J* = 11.0, 6.6 Hz, 1H), 2.97 – 2.83 (m, 2H), 2.26 – 2.10 (m, 1H), 2.09 – 1.92 (m, 3H), 1.33 (s, 3H).

^{13}C NMR: (100 MHz, $CDCl_3$) δ = 206.9, 205.5, 164.1, 163.5, 161.7, 150.6, 135.7, 132.9, 132.8, 130.8, 129.4, 129.3, 123.5, 115.6, 115.4, 95.5, 85.2, 78.2, 49.4, 36.4, 33.8, 26.0, 19.7.

IR: *f* (cm^{-1}) = 2987, 2955, 2945, 2930, 1950, 1727, 1710, 1601, 1525, 1283, 892.

HRMS (ESI-TOF): *m/z* $[M+H]^+$ = 410.1398 calculated for $C_{23}H_{21}FNO_5$; found 410.1431.

Compound (±)-6t

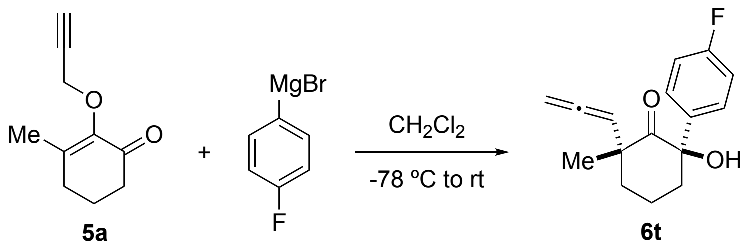

Procedure: Ketone **5a** (150 mg, 0.914 mmol) and CH_2Cl_2 (4.6 mL) were added into a round bottom flask. After cooling the solution to -78 ºC, 4-fluorophenylmagnesium bromide (0.6 mL, 1.2 mmol, 2.0 M in Et_2O) was added dropwise. The reaction mixture was allowed to warm to room temperature and stirred for 25 hours, at which the Claisen rearrangement of the 1,2-carbonyl addition intermediate was complete as monitored by TLC. After cooling to 0 °C, the reaction was quenched with saturated NH_4Cl (5 mL), and the resulting mixture was extracted with CH_2Cl_2 (3 x

5 mL). The organic layers were combined, dried over Na_2SO_4, filtered, and then concentrated under vacuum. The crude material was purified with column chromatography using 80:20 hexanes : EtOAc to yield **6t** in 61% yield (144 mg, 0.553 mmol) as a colorless oil. ^{1}H NMR analysis of the crude reaction mixture indicated > 20:1 dr.

^{1}H NMR: (400 MHz, $CDCl_3$) δ = 7.34 – 7.23 (m, 2H), 7.07 – 6.99 (m, 2H), 4.68 (s, 1H), 4.63 – 4.55 (m, 3H), 2.93 (dq, *J* = 14.1, 3.2 Hz, 1H), 2.05 (dddd, *J* = 18.0, 10.0, 3.9, 2.1 Hz, 2H), 1.93 (td, *J* = 13.6, 4.2 Hz, 1H), 1.85 – 1.75 (m, 1H), 1.70 (ddd, *J* = 13.7, 13.7, 5.3 Hz, 1H), 1.24 (s, 3H).

^{13}C NMR: (100 MHz, $CDCl_3$) δ = 213.5, 206.7, 163.6, 161.1, 136.4, 136.3, 128.1, 128.0, 115.6, 115.4, 94.9, 78.4, 78.2, 47.9, 39.1, 37.4, 25.2, 19.0.

Experimental Data for Scheme 4

Compound (+)-6j

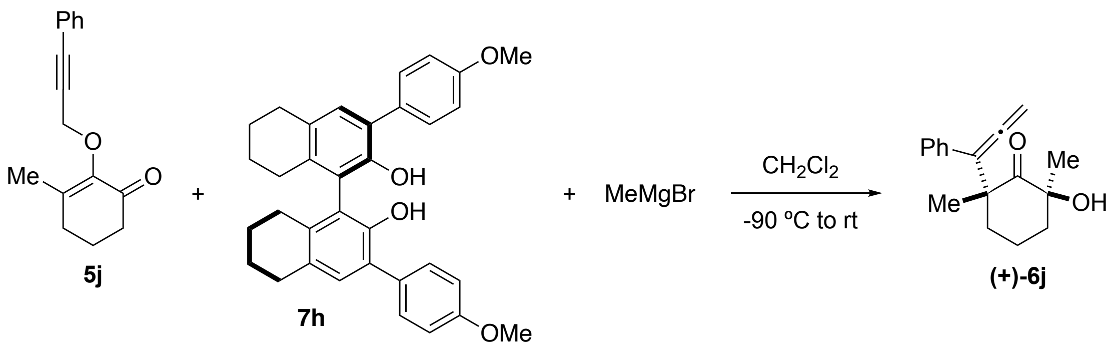

Procedure: Ligand **7h** (3.19 g, 4.49 mmol) and ketone **5j** (1.08 g, 4.49 mmol) were dissolved in CH_2Cl_2 (90 mL) in a round bottom flask. After cooling the solution to -90 °C, methylmagnesium bromide (5.84 mL, 17.5 mmol, 3.0 M in Et_2O) dropwise. After stirring at -90 °C until the starting material was fully consumed as monitored by TLC, the reaction mixture was warmed to room temperature and stirred until the Claisen rearrangement of the 1,2-carbonyl addition intermediate was complete as monitored by TLC for a total reaction time of 7 hours. After cooling to 0 °C, the reaction was quenched with a saturated NH_4Cl solution (50 mL). The aqueous layer was extracted with CH_2Cl_2 (3 x 50 mL), and the combined organic layers were dried over Na_2SO_4, and concentrated under vacuum. The crude material was purified by column chromatography using 100% CH_2Cl_2 for ligand **7h** recovery followed by 80:20 hexanes : EtOAc to afford **(+)-6j** in 93% yield (1.02 g, 4.17 mmol) as a colorless oil. ^1H NMR analysis of the crude reaction mixture indicated > 20:1 dr. Percent recovery of pure ligand **7h** after column chromatography was 91% (2.92 g, 5.76 mmol).

HPLC (+)-6j: IC-3, hexane/isopropanol = 99.0/1.0, flow rate = 1.0 mL/min, λ = 254 nm, t_R = 18.5 min (minor), 20.4 min (major).

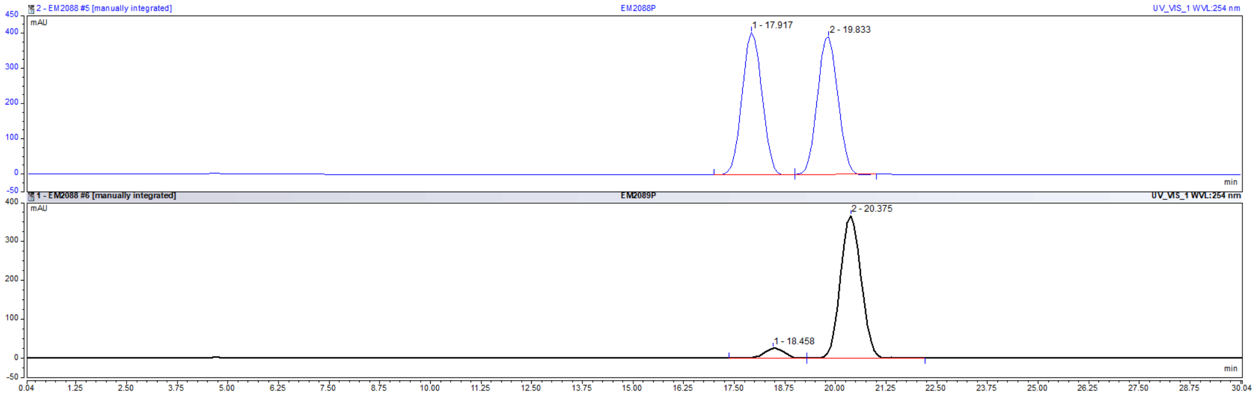

Retention Time (min)	Relative Area (%)	Area (mAU*min)	Height (mAU)
18.458	6.29	14.5584	24.97
20.375	93.71	216.9601	363.78

Compound (±)-6j

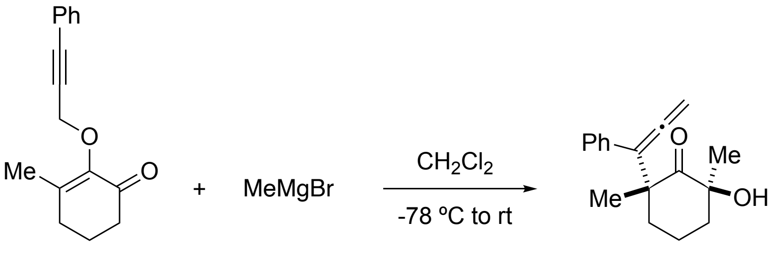

Procedure: Ketone **5j** (1.12 g, 4.66 mmol) and CH_2Cl_2 (23 mL) were added into a round bottom flask. After cooling the solution to -78 ºC, methylmagnesium bromide (2.0 mL, 6.0 mmol, 3.0 M in Et_2O) was added dropwise. The reaction mixture was allowed to warm to room temperature and stirred for 7 hours, at which the Claisen rearrangement of the 1,2-carbonyl addition intermediate was complete as monitored by TLC. After cooling to 0 °C, the reaction was quenched with saturated NH_4Cl (50 mL), and the resulting mixture was extracted with CH_2Cl_2 (3 x 50 mL). The organic layers were combined, dried over Na_2SO_4, filtered, and then concentrated under vacuum. The crude material was purified with column chromatography using 80:20 hexanes : EtOAc to

yield **6j** in 83% yield (996 mg, 3.88 mmol) as a colorless oil. ^{1}H NMR analysis of the crude reaction mixture indicated > 20:1 dr.

Compound (+)-9a

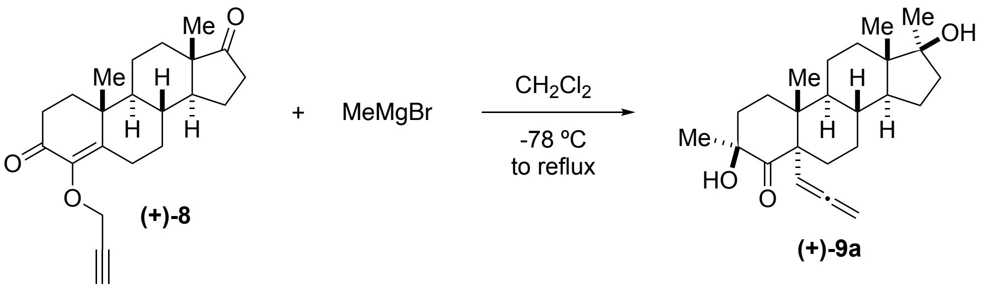

Procedure: Ketone **(+)-8** (80 mg, 0.24 mmol) and CH_2Cl_2 (1.2 mL) were added into a round bottom flask. After cooling the solution to -78 ºC, methylmagnesium bromide (0.20 mL, 0.61 mmol, 3.0 M in Et_2O) was added dropwise. The reaction mixture was allowed to stir at -78 ºC for 30 minutes until the starting material was fully consumed as monitored by TLC. The round bottom flask was warmed to room temperature and then heated in an oil bath to bring the reaction mixture to gentle reflux for 71 hours, at which the Claisen rearrangement of the 1,2-carbonyl addition intermediate was complete as monitored by TLC. After cooling to 0 °C, the reaction was quenched with saturated NH_4Cl (5 mL), and the resulting mixture was extracted with CH_2Cl_2 (3 x 5 mL). The organic layers were combined, dried over Na_2SO_4, filtered, and then concentrated under vacuum. The crude material was purified with column chromatography using 60:40 hexanes : EtOAc to yield **(+)-9a** in 66% yield (58 mg, 0.14 mmol) as a white crystalline solid. ^{1}H NMR analysis of the crude reaction mixture indicated > 20:1 dr.

Rf: 0.6 in 70:30 hexanes : EtOAc (TLC was run twice)

^{1}H NMR: (400 MHz, $CDCl_3$) δ = 5.53 (t, *J* = 6.8 Hz, 1H), 4.81 (dd, *J* = 11.5, 6.9 Hz, 1H), 4.70 (dd, *J* = 11.5, 6.8 Hz, 1H), 3.98 (s, 1H), 2.05 (ddd, *J* = 14.1, 5.0, 2.7 Hz, 1H), 1.96 (td, *J* = 13.6,

5.8 Hz, 1H), 1.83 – 1.61 (m, 7H), 1.60 – 1.46 (m, 5H), 1.40 (s, 3H), 1.36 – 1.20 (m, 9H), 0.89 (s, 3H), 0.84 (s, 3H).

^{13}C NMR: (100 MHz, $CDCl_3$) δ = 214.5, 208.5, 91.7, 81.5, 77.9, 74.8, 54.9, 50.4, 46.3, 45.6, 42.0, 39.0, 36.6, 35.4, 31.7, 31.1, 28.4, 26.9, 25.8, 25.4, 23.0, 21.1, 17.0, 14.1.

IR: *f* (cm^{-1}) = 3518, 2967, 2939, 2884, 2864, 1684, 1452, 1357, 1243, 712.

HRMS (ESI-TOF): *m/z* $[M+H]^+$ = 373.2737 calculated for $C_{24}H_{37}O_3$; found 373.2739.

Specific Rotation: $[\alpha]_{25}^{D}$ = +29 (c = 1.0 in $CHCl_3$)

X-Ray Crystallography: The ellipsoid contour was set at a 50% probability level.

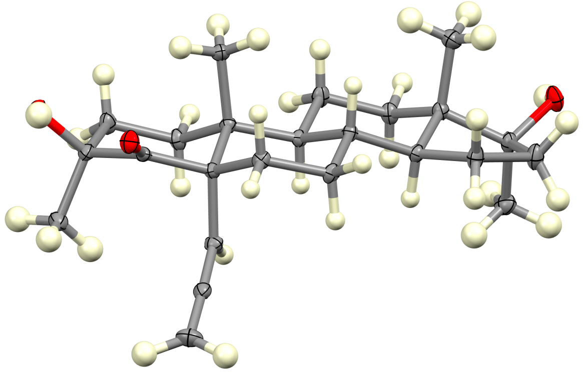

Compound (-)-9b

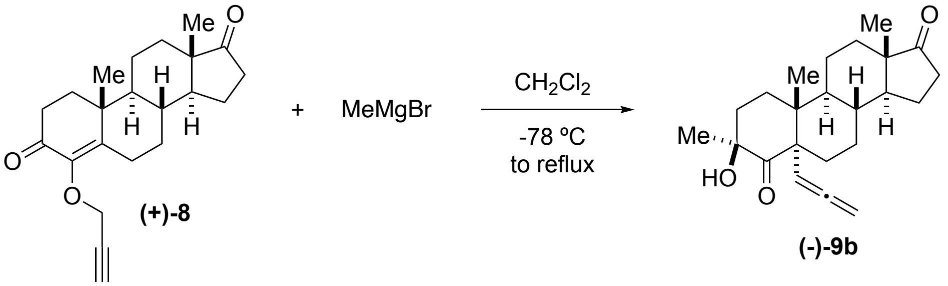

Procedure: Ketone **(+)-8** (68 mg, 0.20 mmol) and CH_2Cl_2 (1.0 mL) were added into a round bottom flask. After cooling the solution to -78 ºC, methylmagnesium bromide (73 μL, 0.22 mmol, 3.0 M in Et_2O) was added dropwise. The reaction mixture was allowed to stir at -78 ºC for 30

minutes until the starting material was fully consumed as monitored by TLC. The round bottom flask was warmed to room temperature and then heated in an oil bath to bring the reaction mixture to gentle reflux for 72 hours, at which the Claisen rearrangement of the 1,2-carbonyl addition intermediate was complete as monitored by TLC. After cooling to 0 °C, the reaction was quenched with saturated NH_4Cl (5 mL), and the resulting mixture was extracted with CH_2Cl_2 (3 x 5 mL). The organic layers were combined, dried over Na_2SO_4, filtered, and then concentrated under vacuum. The crude material was purified with column chromatography using 96:4 dichloromethane : acetone to yield **(-)-9b** in 40% yield (29 mg, 0.079 mmol) as a white crystalline solid.

Rf: 0.4 in 96:4 dichloromethane : acetone

^{1}H NMR: (400 MHz, $CDCl_3$) δ = 5.51 (t, *J* = 6.8 Hz, 1H), 4.82 (dd, *J* = 11.6, 6.8 Hz, 1H), 4.72 (dd, *J* = 11.6, 6.8 Hz, 1H), 3.96 (s, 1H), 2.44 (dd, *J* = 19.4, 9.0 Hz, 1H), 2.13 – 2.02 (m, 2H), 2.01 – 1.90 (m, 2H), 1.89 – 1.72 (m, 4H), 1.71 – 1.58 (m, 4H), 1.52 (tt, *J* = 12.4, 8.9 Hz, 1H), 1.41 (s, 3H), 1.38 – 1.23 (m, 5H), 0.90 (s, 3H), 0.86 (s, 3H).

^{13}C NMR: (100 MHz, $CDCl_3$) δ = 220.5, 214.2, 208.6, 91.5, 78.0, 74.8, 54.8, 51.2, 47.8, 46.4, 42.0, 36.5, 35.8, 34.2, 31.6, 31.1, 28.4, 26.6, 24.6, 21.6, 20.8, 17.0, 13.9.

IR: *f* (cm^{-1}) = 2949, 2921, 2854, 1729, 1686, 1449, 1371, 1259, 1006.

HRMS (ESI-TOF): *m/z* $[M+H]^+$ = 357.2424 calculated for $C_{23}H_{33}O_3$; found 357.2435.

Specific Rotation: $[\alpha]_{25}^{D}$ = +4.4 (c = 1.0 in $CHCl_3$)

X-Ray Crystallography: The ellipsoid contour was set at a 50% probability level.

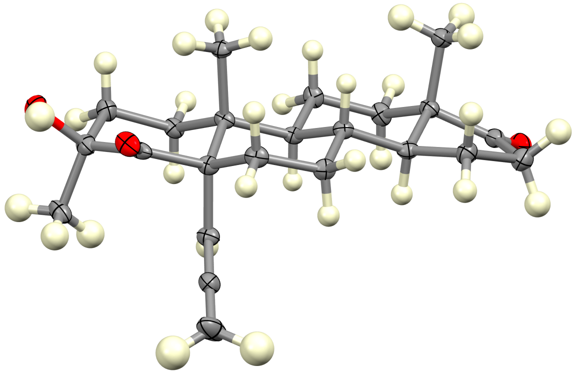

Compound (+)-10

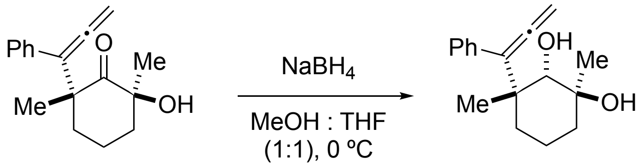

Procedure: Ketone **(+)-6j** (208 mg, 0.812 mmol) was added into a round bottom flask and dissolved in a mixture of THF (10 mL) and MeOH (10 mL). After cooling the solution to 0 ºC, $NaBH_4$ (77 mg, 2.0 mmol) was added in two portions within 10 minutes. The reaction mixture was stirred for 40 minutes until completion of the reaction as monitored by TLC. The reaction was quenched with water (5 mL), and the resulting mixture was extracted with CH_2Cl_2 (3 x 5 mL). The crude reaction mixture was concentrated under vacuum and carried to the next step without further purification.

Rf: 0.4 in 80:20 Hexanes : EtOAc

^{1}H NMR: (400 MHz, $CDCl_3$) δ = 7.36 – 7.24 (m, 5H), 4.89 (dd, *J* = 12.3, 10.9 Hz, 2H), 3.34 (d, *J* = 3.3 Hz, 1H), 2.15 (d, *J* = 3.4 Hz, 1H), 1.86 – 1.66 (m, 4H), 1.53 – 1.42 (m, 3H), 1.35 (s, 3H), 1.33 (s, 3H).

^{13}C NMR: (100 MHz, $CDCl_3$) δ = 207.7, 136.2, 129.2, 128.2, 127.2, 112.7, 76.4, 73.2, 42.8, 34.2, 31.8, 29.2, 24.1, 18.2.

IR: *ƒ* (cm^{-1}) = 3435, 3058, 2930, 2871, 1449, 1375, 1261, 1084, 1029, 702.

HRMS (ESI-TOF): *m/z* [M-H_2O+H]+ = 241.1587 calculated for $C_{17}H_{21}O$; found 241.1592.

Compound (+)-11

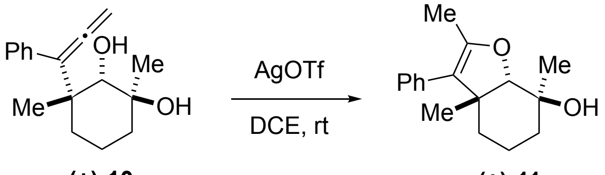

Procedure: Alcohol **(+)-10** (209 mg, 0.808 mmol) was dissolved in DCE (4.0 ml, 0.2 M). Ag(OTf) (10 mg, 0.040 mmol) was then added. The reaction mixture was stirred at room temperature for 10 minutes until completion of the reaction as monitored by TLC. The crude reaction mixture was concentrated under vacuum and then purified by column chromatography using 90:10 hexanes : EtOAc to afford compound **(+)-11** in 86% yield (179 mg, 0.693 mmol) as a white solid.

Rf: 0.5 in 80:20 Hexanes : EtOAc

^{1}H NMR: (400 MHz, $CDCl_3$) δ = 7.31 (t, *J* = 7.4 Hz, 2H), 7.21 (ddt, *J* = 6.7, 6.7, 1.4 Hz, 1H), 7.18 – 7.13 (m, 2H), 3.70 (s, 1H), 1.88 – 1.78 (m, 4H), 1.72 – 1.59 (m, 3H), 1.57 – 1.43 (m, 2H), 1.40 (s, 3H), 1.22 (s, 3H), 1.21 (s, 1H).

^{13}C NMR: (100 MHz, $CDCl_3$) δ = 149.2, 135.3, 129.2, 128.0, 125.9, 122.9, 91.7, 70.7, 47.2, 35.2, 34.4, 29.0, 23.4, 16.9, 12.8.

IR: *ƒ* (cm^{-1}) = 3449, 3053, 2928, 2866, 1657, 5099, 1453, 1381, 1260, 699.

HRMS (ESI-TOF): *m/z* [M-H_2O+H]+ = 241.1587 calculated for $C_{17}H_{21}O$; found 241.1596.

Compound (-)-12

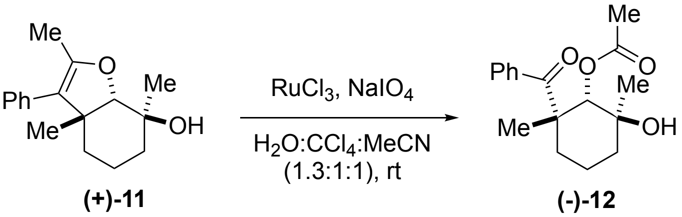

Procedure: Compound **(+)-11** (117 mg, 0.449 mmol) was dissolved in a mixture of $H_2O/CCl_4/CH_3CN$ (1.3:1:1, 0.05 M). $NaIO_4$ (384 mg, 1.80 mmol) was then added, followed by $RuCl_3$ (4.7 mg, 0.022 mmol). The reaction mixture was stirred at room temperature for 3 hours until the completion of reaction as monitored by TLC. The reaction mixture was quenched with saturated aqueous $Na_2S_2O_3$ (5 mL), stirred for 15 min, and then extracted with CH_2Cl_2 (3 x 5 mL). The organic layers were combined, dried over Na_2SO_4, filtered, and then concentrated under vacuum. The crude material was purified with column chromatography using 70:30 hexanes : EtOAc to yield **(-)-12** in 71% yield (93 mg, 0.32 mmol) as a dark oil.

Rf: 0.2 in 80:20 Hexanes : EtOAc

^{1}H NMR: (400 MHz, $CDCl_3$) δ = 7.69 (d, *J* = 7.8 Hz, 2H), 7.45 (t, *J* = 7.0 Hz, 1H), 7.37 (t, *J* = 7.4 Hz, 2H), 5.40 (s, 1H), 1.98 (ddd, *J* = 13.3, 13.3, 3.6 Hz, 1H), 1.93 – 1.80 (m, 1H), 1.77 (s, 3H), 1.71 (d, *J* = 13.4, Hz, 1H), 1.65 (s, 3H), 1.62 – 1.54 (m, 2H), 1.52 (dd, *J* = 4.9, 4.9 Hz, 1H), 1.10 (s, 3H).

^{13}C NMR: (100 MHz, $CDCl_3$) δ = 206.6, 169.2, 138.2, 131.3, 128.0, 127.8, 77.7, 72.7, 51.3, 34.7, 29.9, 28.7, 22.1, 20.7, 17.0.

IR: *f* (cm^{-1}) = 3479, 3059, 2935, 2871, 1729, 1673, 1371, 1225, 1165, 752.

HRMS (ESI-TOF): *m/z* [M-H_2O+H]+ = 273.1485 calculated for $C_{17}H_{21}O_3$; found 273.1490.

Compound (±)-10

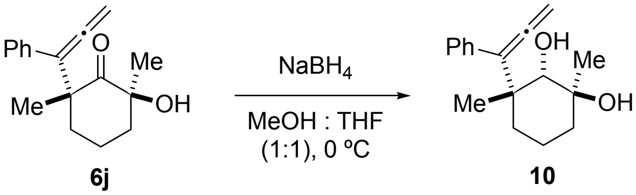

Procedure: Ketone **6j** (206 mg, 0.805 mmol) was added into a round bottom flask and dissolved in a mixture of THF (10 mL) and MeOH (10 mL). After cooling the solution to 0 ºC, $NaBH_4$ (76 mg, 2.0 mmol) was added in two portions within 10 minutes. The reaction mixture was stirred for 40 minutes until completion of the reaction as monitored by TLC. The reaction was quenched with water (5 mL), and the resulting mixture was extracted with CH_2Cl_2 (3 x 5 mL). The crude reaction mixture was concentrated under vacuum and carried to the next step without further purification.

Compound (±)-11

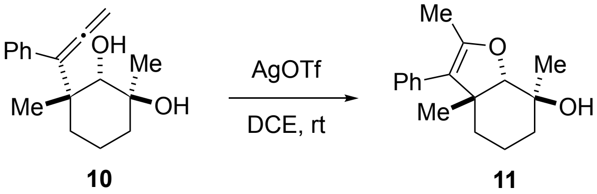

Procedure: Alcohol **10** (206 mg, 0.799 mmol) was dissolved in DCE (4.0 ml, 0.2 M). Ag(OTf) (10 mg, 0.040 mmol) was then added. The reaction mixture was stirred at room temperature for 10 minutes until completion of the reaction as monitored by TLC. The crude reaction mixture was concentrated under vacuum and then purified by column chromatography using 90:10 hexanes : EtOAc to afford compound **11** in 87% yield (180 mg, 0.697 mmol) as a white solid.

X-Ray Crystallography: The ellipsoid contour was set at a 50% probability level.

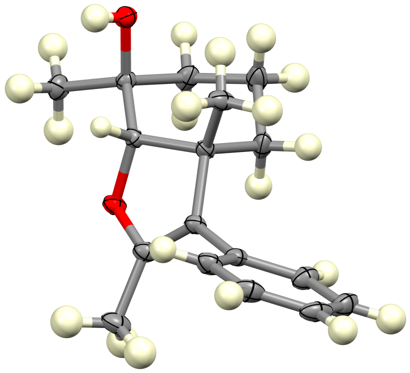

Compound (±)-12

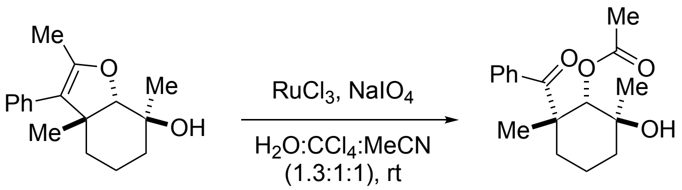

11 **12**

Procedure: Compound **11** (122 mg, 0.473 mmol) was dissolved in a mixture of $H_2O/CCl_4/CH_3CN$ (1.3:1:1, 0.05 M). $NaIO_4$ (404 mg, 1.89 mmol) was then added, followed by $RuCl_3$ (4.9 mg, 0.024 mmol). The reaction mixture was stirred at room temperature for 3 hours until the completion of reaction as monitored by TLC. The reaction mixture was quenched with saturated aqueous $Na_2S_2O_3$ (5 mL), stirred for 15 min, and then extracted with CH_2Cl_2 (3 x 5 mL). The organic layers were combined, dried over Na_2SO_4, filtered, and then concentrated under vacuum. The crude material was purified with column chromatography using 70:30 hexanes : EtOAc to yield **12** in 70% yield (96 mg, 0.32 mmol) as a dark oil.

X-Ray Crystallography: The ellipsoid contour was set at a 50% probability level.

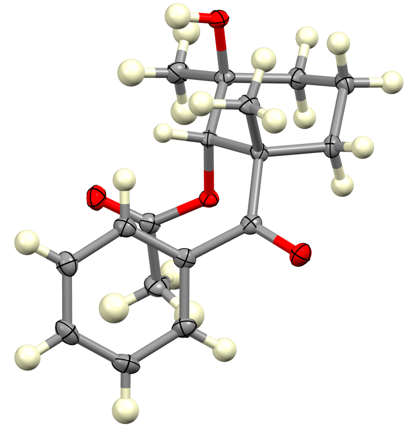

Compound (+)-14

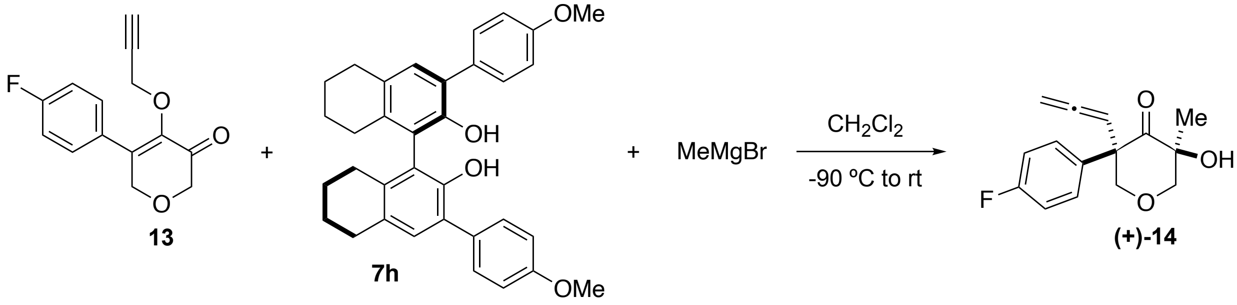

Procedure: Ligand **7h** (181 mg, 0.357 mmol) and ketone **13** (63 mg, 0.26 mmol) were dissolved in CH_2Cl_2 (5.1 mL) in a round bottom flask. The solution was cooled to -90 °C, followed by the addition of methylmagnesium bromide (0.33 mL, 0.99 mmol, 3.0 M in Et_2O) dropwise. After stirring at -90 °C until the starting material was fully consumed as monitored by TLC, the reaction mixture was warmed to room temperature and stirred until the Claisen rearrangement of the 1,2-carbonyl addition intermediate was complete as monitored by TLC for a total reaction time of 69 hours. After cooling to 0 °C, the reaction was quenched with a saturated NH_4Cl solution (8 mL) and diluted with DI water (8 mL). The aqueous layer was extracted with CH_2Cl_2 (3 x 10 mL). The combined organic layers were dried over Na_2SO_4 and concentrated under vacuum. The crude

material was purified by column chromatography using 100% CH_2Cl_2 for ligand **7h** recovery, followed by 80:20 hexanes : EtOAc to afford **(+)-14** in 73% yield (49 mg, 0.19 mmol) as a yellow oil. ^{1}H NMR analysis of the crude reaction mixture indicated > 20:1 dr.

Rf: 0.4 in 70:30 hexanes : EtOAc

^{1}H NMR: (400 MHz, $CDCl_3$) δ = 7.25 – 7.18 (m, 2H), 7.01 – 6.95 (m, 2H), 5.41 (t, *J* = 6.7 Hz, 1H), 4.90 (qd, *J* = 11.6, 8.0 Hz, 2H), 4.02 (s, 2H), 3.87 (d, *J* = 11.6 Hz, 1H), 3.72 (d, *J* = 11.6 Hz, 1H), 3.08 (s, 1H), 1.41 (s, 3H)

^{13}C NMR: (100 MHz, $CDCl_3$) δ = 209.2, 208.8, 163.3, 160.9, 134.0, 134.0, 129.7, 129.7, 115.6, 115.4, 92.5, 79.3, 77.8, 76.7, 75.0, 57.7, 25.2.

IR: *ƒ* (cm^{-1}) = 3428, 3062, 2964, 2928, 2857, 1717, 1451, 1376, 1131, 835

HRMS (ESI-TOF): *m/z* $[M+H]^+$ = 263.1078 calculated for $C_{15}H_{16}FO_3$; found 263.1086.

Specific Rotation: $[\alpha]_{25}^{D}$ = + 6.8 (c = 1.0 in $CHCl_3$)

HPLC ((+)-14)-BzNO$_2$: IC-3, hexane/isopropanol = 90/10, flow rate = 1.0 mL/min, λ = 254 nm, t_R = 11.5 min (major), 14.3 min (minor).

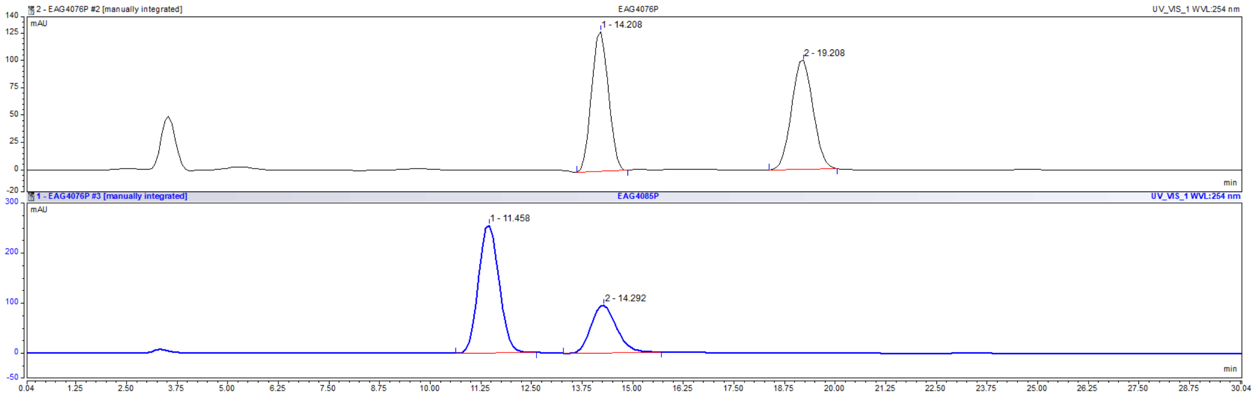

Retention Time (min)	Relative Area (%)	Area (mAU*min)	Height (mAU)
11.458	68.12	149.3606	255.12
14.292	31.88	69.8992	95.39

Compound ((+)-14)-BzNO₂

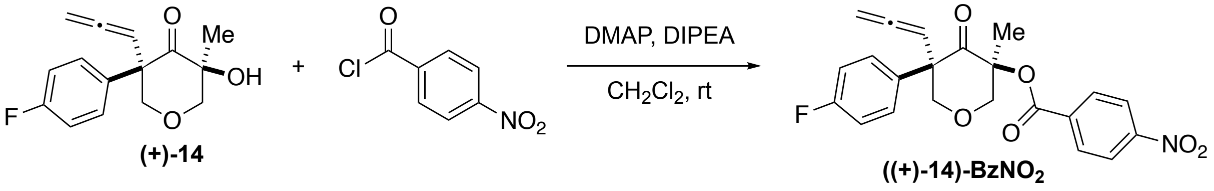

Procedure: Alcohol **(+)-14** (15 mg, 0.055 mmol) was dissolved in CH_2Cl_2 (0.3 mL). *N,N*-Diisopropylethylamine (48 µL, 0.28 mmol) was added to the solution, followed by 4- nitrobenzoyl chloride (33 mg, 0.18 mmol) and 4-(dimethylamino)pyridine (6.8 mg, 0.055 mmol). The reaction mixture was then stirred at room temperature until complete consumption of the starting material as monitored by TLC. The crude reaction mixture was concentrated under vacuum and then purified by column chromatography using 85:15 hexanes : EtOAc to afford compound **((+)-14)-BzNO₂** in 88% yield (20 mg, 0.049 mmol) as a white solid.

Rf: 0.7 in 70:30 hexanes : EtOAc

^{1}H NMR: (500 MHz, $CDCl_3$) δ = 8.29 – 8.24 (m, 2H), 8.16 – 8.10 (m, 2H), 7.53 – 7.45 (m, 2H), 7.02 – 6.94 (m, 2H), 5.46 (t, *J* = 6.6 Hz, 1H), 5.03 (qd, *J* = 11.7, 6.8 Hz, 2H), 4.54 (d, *J* = 11.1 Hz, 1H), 4.21 (d, *J* = 12.2 Hz, 1H), 4.11 (d, *J* = 12.0 Hz, 1H), 3.93 (d, *J* = 11.1 Hz, 1H).

^{13}C NMR: (125 MHz, $CDCl_3$) δ = 209.0, 203.6, 163.5, 163.0, 161.0, 150.8, 134.5, 134.2, 134.2, 131.0, 130.3, 130.3, 123.4, 115.3, 115.1, 93.2, 81.0, 79.4, 76.0, 74.4, 59.4, 24.6.

IR: *f* (cm^{-1}) = 3109, 2987, 2961, 2862, 1726, 1711, 1346, 1289, 1231, 837.

HRMS (ESI-TOF): *m/z* $[M+H]^+$ = 412.1191 calculated for $C_{22}H_{19}FNO_6$; found 412.1198.

Compound (±)-14

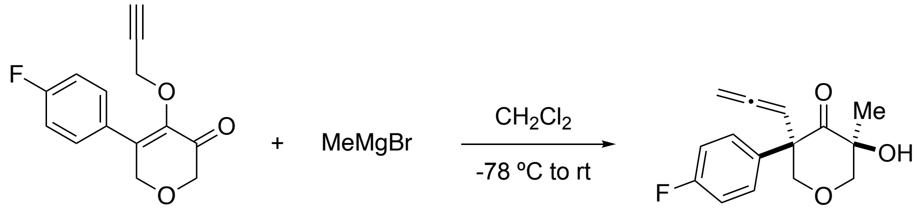

Procedure: Ketone **13** (31 mg, 0.124 mmol) and CH_2Cl_2 (0.63 mL) were added into a round bottom flask. After cooling the solution to -78 ºC, methylmagnesium bromide (0.54 mL, 0.16 mmol, 3.0 M in Et_2O) was added dropwise. The reaction mixture was allowed to warm to room temperature and stirred for 65 hours, at which the Claisen rearrangement of the 1,2-carbonyl addition intermediate was complete as monitored by TLC. After cooling to 0 °C, the reaction was quenched with saturated NH_4Cl (5 mL), and the resulting mixture was extracted with CH_2Cl_2 (3 x 5 mL). The organic layers were combined, dried over Na_2SO_4, filtered, and then concentrated under vacuum. The crude material was purified with column chromatography using 80:20 hexanes : EtOAc to yield **14** in 54% yield (18 mg, 0.068 mmol) as a yellow oil. ^{1}H NMR analysis of the crude reaction mixture indicated > 20:1 dr.

^{1}H NMR: (400 MHZ, $CDCl_3$) δ =7.25 – 7.18 (m, 2H), 7.01 – 6.94 (m, 2H), 5.41 (t, *J* = 6.7 Hz), 4.90 (qd, *J* = 11.6, 6.8 Hz, 2H), 4.02 (s, 2H), 3.87 (d, *J* = 11.6 Hz, 1H), 3.72 (d, *J* = 11.6 Hz, 1H), 3.08 (s, 1H), 1.42 (s, 3H).

^{13}C NMR: (100 MHz, $CDCl_3$) δ = 209.2, 208.8, 163.3, 160.9, 134.0, 129.7, 129.7, 115.6, 115.4, 92.5, 79.3, 77.8, 76.7, 75.0, 57.7, 25.2.

Synthesis of Substrates

Compound 5a

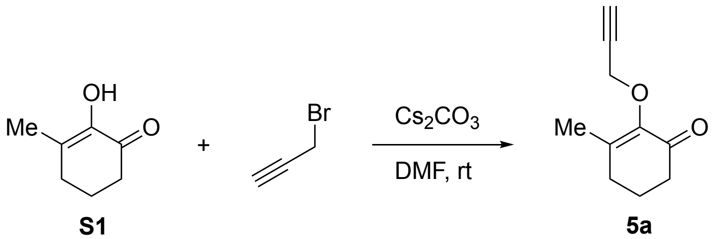

Procedure: Starting material **S1** (3.00 g, 23.8 mmol) was dissolved in DMF (119 mL). Propargyl bromide (5.30 mL, 47.6 mmol) and Cs_2CO_3 (15.5 g, 47.6 mmol) were added to the solution. The reaction mixture was stirred at room temperature until the starting material was fully consumed as monitored by TLC. The reaction mixture was diluted with DI water (20 mL). The aqueous layer was then extracted with EtOAc (3 x 20 mL). The combined organic layers were dried over Na_2SO_4 and then concentrated under vacuum. The crude material was purified with column chromatography using 87.5:12.5 hexanes : EtOAc to yield compound **5a** in 78% yield (3.05 g, 18.6 mmol) as a yellow oil.

Rf: 0.4 in 80:20 Hexanes : EtOAc

^{1}H NMR: (400 MHz, $CDCl_3$) δ = 4.57 (dd, J = 2.2, 1.7 Hz, 2H), 2.45 – 2.36 (m, 5H), 1.97 – 1.88 (m, 5H).

^{13}C NMR: (100 MHz, $CDCl_3$) δ = 194.4, 148.2, 146.3, 79.6, 74.8, 58.7, 38.5, 31.5, 22.0, 18.3.

IR: f (cm^{-1}) = 3261, 2937, 2870, 2829, 1667, 1631, 1147, 1380, 1302, 988.

HRMS (ESI-TOF): m/z $[M+H]^+$ = 165.0910 calculated for $C_{10}H_{13}O_2$ found 165.0915.

Compound 5b

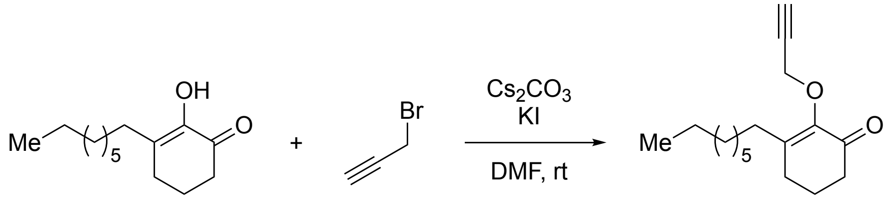

S2 **5b**

Procedure: Starting material **S2** (587 mg, 2.62 mmol) was dissolved in DMF (26 mL). Propargyl bromide (583 µL, 5.23 mmol), Cs_2CO_3 (1.71 g, 5.23 mmol), and potassium iodide (43 mg, 0.26 mmol) were added to the solution. The reaction mixture was stirred at room temperature until the starting material was fully consumed as monitored by TLC. The reaction mixture was diluted with DI water (20 mL). The aqueous layer was then extracted with EtOAc (3 x 20 mL). The combined organic layers were dried over Na_2SO_4 and then concentrated under vacuum. The crude material was purified with column chromatography using 92:8 hexanes : EtOAc to yield compound **5b** in 59% yield (408 mg, 1.55 mmol) as a yellow oil.

Rf: 0.6 in 80:20 hexanes : EtOAc

^{1}H NMR: (400 MHz, $CDCl_3$) δ = 4.61 (d, *J* = 2.4 Hz, 2H), 2.49 – 2.33 (m, 7H), 1.94 (p, *J* = 6.3 Hz, 2H), 1.51 – 1.41 (m, 2H), 1.37 – 1.20 (m, 10H), 0.88 (t, *J* = 5.8 Hz, 3H).

^{13}C NMR: (100 MHz, $CDCl_3$) δ = 194.8, 151.9, 146.0, 79.6, 74.9, 58.8, 38.7, 31.8, 31.7, 29.7, 29.4, 29.4, 29.2, 27.5, 22.6, 22.3, 14.1.

IR: *f* (cm^{-1}) = 2953, 2923, 2854, 1674, 1457, 1370, 1141, 1093.

HRMS (ESI-TOF): *m/z* $[M+H]^+$ = 263.2006 calculated for $C_{17}H_{27}O_2$ found 263.2017.

Compound 5c

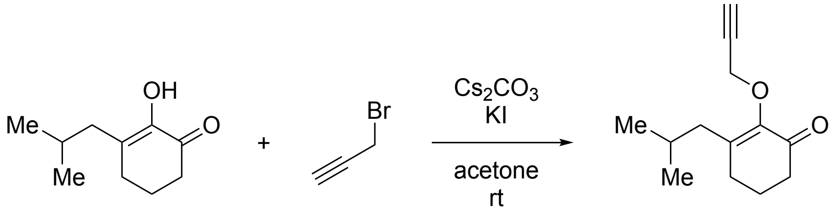

Procedure: Starting material **S3** (150 mg, 0.892 mmol) was dissolved in acetone (8.9 mL). Propargyl bromide (199 µL, 1.78 mmol), Cs_2CO_3 (581 mg, 1.78 mmol), and potassium iodide (15 mg, 0.089 mmol) were added to the solution. The reaction mixture was stirred at room temperature until the starting material was fully consumed as monitored by TLC. The reaction mixture was diluted with DI water (20 mL). The aqueous layer was then extracted with CH_2Cl_2 (3 x 20 mL). The combined organic layers dried over Na_2SO_4 and then concentrated under vacuum. The crude material was purified with column chromatography using 80:20 hexanes : EtOAc to yield compound **5c** in 90% yield (165 mg, 0.798 mmol) as a yellow oil.

Rf: 0.5 in 80:20 hexanes : EtOAc

^{1}H NMR: (400 MHz, $CDCl_3$) δ = 4.60 (d, J = 2.4 Hz, 2H), 2.41 (dddd, J = 17.4, 7.4, 7.4, 6.12 Hz, 5H), 2.28 (d, J = 7.6 Hz, 2H), 1.90 (dp, J = 23, 6.3 Hz, 3H), 0.91 (d, J = 6.6 Hz, 6H).

^{13}C NMR: (100 MHz, $CDCl_3$) δ = 194.7, 150.6, 146.9, 79.6, 74.9, 58.7, 40.7, 38.7, 29.8, 26.9, 22.7, 22.3.

IR: f (cm^{-1}) = 3269, 2955, 2869, 1671, 1621, 1453, 1368, 1144.

HRMS (ESI-TOF): m/z $[M+H]^+$ = 207.1380 calculated for $C_{13}H_{19}O_2$ found 207.1381.

Compound 5d

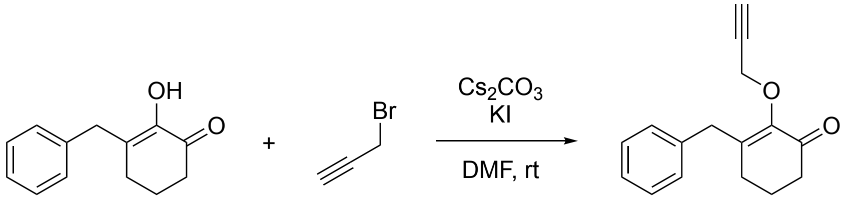

S4 5d

Procedure: Starting material **S4** (139 mg, 0.687 mmol) was dissolved in DMF (6.9 mL). Propargyl bromide (153 μL, 1.37 mmol), Cs_2CO_3 (448 mg, 1.37 mmol), and potassium iodide (11 mg, 0.069 mmol) were added to the solution. The reaction mixture was stirred at room temperature until the starting material was fully consumed as monitored by TLC. The reaction mixture was diluted with DI water (20 mL). The aqueous layer was then extracted with EtOAc (3 x 20 mL). The combined organic layers were dried over Na_2SO_4 and then concentrated under vacuum. The crude material was purified with column chromatography using 80:20 hexanes : EtOAc to yield compound **5d** in 74% yield (123 mg, 0.511 mmol) as a yellow solid.

Rf: 0.5 in 80:20 hexanes : EtOAc

^{1}H NMR: (400 MHz, $CDCl_3$) δ = 7.32 – 7.19 (m, 5H), 4.72(d, J = 2.4 Hz, 2H), 3.75 (s, 2H), 2.46 (m, 3H), 2.31 (t, J = 6.0 Hz, 2H), 1.89 (p, J = 6.4 Hz, 2H).

^{13}C NMR: (100 MHz, $CDCl_3$) δ = 195.0, 149.1, 146.1, 138.2, 129.1, 128.5, 126.4, 79.6, 75.3, 59.0, 38.7, 37.6, 28.9, 22.2.

IR: f (cm^{-1}) = 3085, 3061, 3027, 2940, 2891, 2868, 1671, 1452, 1304, 1116.

HRMS (ESI-TOF): m/z $[M+H]^+$ = 241.1223 calculated for $C_{16}H_{17}O_2$ found 241.1234.

Compound 5e

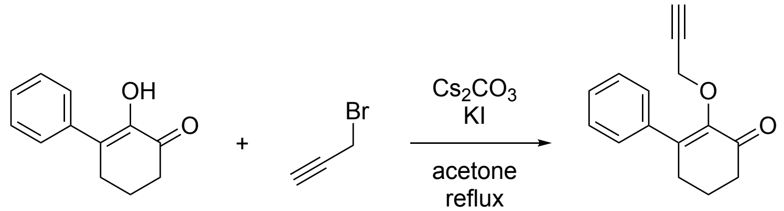

S5 **5e**

Procedure: Starting material **S5** (1.74 g, 9.24 mmol) was dissolved in acetone (23 mL). Propargyl bromide (2.06 g, 18.5 mmol), K_2CO_3 (3.83 g, 27.7 mmol), and potassium iodide (154 mg, 0.924 mmol) were added to the solution. The reaction mixture was stirred at reflux in an oil bath until the starting material was fully consumed as monitored by TLC. After cooled to room temperature, the reaction mixture was concentrated under vacuum and diluted with DI water (20 mL). The aqueous layer was then extracted with CH_2Cl_2 (3 x 20 mL). The combined organic layers were dried over Na_2SO_4 and then concentrated under vacuum. The crude material was purified with column chromatography using 93:7 hexanes : EtOAc to yield compound **5e** in 71% yield (1.48 g, 6.54 mmol) as a yellow oil.

Rf: 0.4 in 80:20 hexanes : EtOAc

^{1}H NMR: (400 MHz, $CDCl_3$) δ = 7.53 – 7.51 (m, 2H), 7.39 – 7.29 (m, 3H), 4.54 (d, *J* = 2.5 Hz, 2H), 2.79 (t, *J* = 6.0 Hz, 2H), 2.56 (t, *J* = 6.6, 2H), 2.33 (t, *J* = 4.9 Hz, 1H), 2.07 (p, *J* = 6.3, 2H).

^{13}C NMR: (100 MHz, $CDCl_3$) δ = 195.4, 145.8, 145.5, 137.2, 128.5, 128.0, 127.9, 78.8, 75.4, 58.6, 38.5, 31.0, 22.4.

IR: *f* (cm^{-1}) = 3287, 3056, 2941, 2868, 2121,1668, 1600, 1302, 1132, 983.

HRMS (ESI-TOF): *m/z* $[M+H]^+$ = 227.1067 calculated for $C_{15}H_{15}O_2$; found 227.1072.

Compound 5f

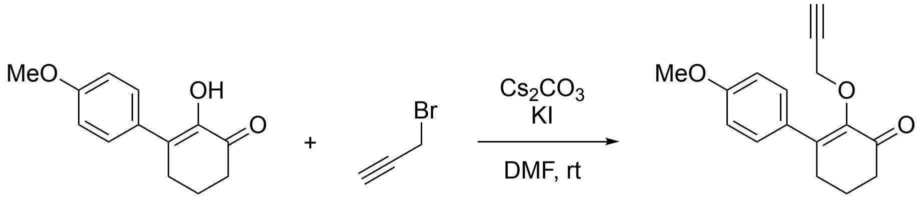

S6 5f

Procedure: Starting material **S6** (658 mg, 3.01 mmol) was dissolved in acetone (15 mL). Propargyl bromide (518 mL, 6.01 mmol), Cs_2CO_3 (2.94 g, 9.02 mmol), and potassium iodide (100 mg, 0.601 mmol) were added to the solution. The reaction mixture was stirred at room temperature until the starting material was fully consumed as monitored by TLC. The reaction mixture was diluted with DI water (20 mL). The aqueous layer was then extracted with CH_2Cl_2 (3 x 20 mL). The combined organic layers were dried over Na_2SO_4 and then concentrated under vacuum. The crude material was purified with column chromatography using 80:20 hexanes : EtOAc to yield compound **5f** in 83% yield (569 mg, 2.15 mmol) as a yellow solid.

Rf: 0.4 in 80:20 hexanes : EtOAc

^{1}H NMR: (400 MHz, $CDCl_3$) δ = 7.57 (dt, *J* = 9.9, 3.1 Hz, 2H), 6.90 (dt, *J* = 10.0, 3.2 Hz, 2H), 4.54 (d, *J* = 2.4 Hz, 2H), 3.83 (s, 3H), 2.79 (t, *J* = 6.0 Hz, 2H), 2.56 (dd, *J* = 7.5, 6.1 Hz, 2H), 2.33 (t, *J* = 4.9 Hz, 1H), 2.07 (p, *J* = 6.3 Hz, 2H).

^{13}C NMR: (100 MHz, $CDCl_3$) δ = 195.4, 159.9, 145.7, 145.0, 129.9, 129.3, 113.4, 79.0, 75.2, 58.6, 55.2, 38.5, 30.8, 22.4.

IR: *f* (cm^{-1}) = 3284, 2939, 2868, 2838, 1965, 1604, 1250, 1181, 1032, 830.

HRMS (ESI-TOF): *m/z* $[M+H]^+$ = 257.1172 calculated for $C_{16}H_{17}O_3$; found 257.1178.

Compound 5g

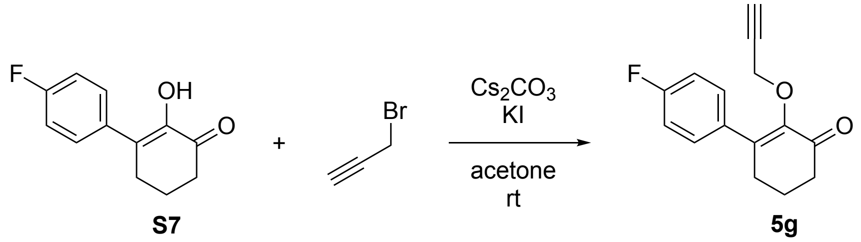

Procedure: Starting material **S7** (665 mg, 3.22 mmol) was dissolved in acetone (22 mL). Propargyl bromide (718 µL, 6.45 mmol), Cs_2CO_3 (3.15 g, 9.67 mmol), and potassium iodide (54 mg, 0.32) were added to the solution. The reaction mixture was stirred at reflux in an oil bath until the starting material was fully consumed as monitored by TLC. After cooled to room temperature, the reaction mixture was concentrated under vacuum and diluted with DI water (20 mL). The aqueous layer was then extracted with CH_2Cl_2 (3 x 20 mL). The combined organic layers were dried over Na_2SO_4 and then concentrated under vacuum. The crude material was purified with column chromatography using 90:10 hexanes : EtOAc to yield compound **5g** in 71% yield (560 mg, 0.560 mmol) as a yellow oil.

Rf: 0.6 in 70:30 hexanes : EtOAc

^{1}H NMR: (400 MHz, $CDCl_3$) δ = 7.53 (ddt, *J* = 5.2, 5.2, 3.1 Hz, 2H), 7.03 (tdd, *J* = 8.5, 5.3, 3.1 Hz, 2H), 4.54 (d, *J* = 2.5 Hz, 2H), 2.75 (t, *J* = 6.0 Hz, 2H), 2.55 (dd, *J* = 7.6, 6.2 Hz, 2H), 2.32 (t, *J* = 2.5 Hz, 1H), 2.08 (p, *J* = 6.2 Hz, 2H).

^{13}C NMR: (100 MHz, $CDCl_3$) δ = 195.3, 163.8, 161.3, 145.8, 144.3, 133.1, 133.1, 130.2, 130.1, 115.0, 114.8, 78.8, 75.5, 58.6, 38.5, 30.9, 22.3.

IR: *f* (cm^{-1}) = 3293, 2943, 2870, 1670, 1508, 1407, 1368, 1307, 833.

HRMS (ESI-TOF): *m/z* $[M+H]^+$ = 245.0972 calculated for $C_{15}H_{14}FO_2$; found 245.0971.

Compound 5h

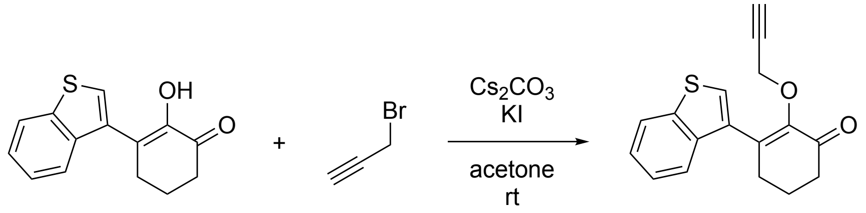

Procedure: Starting material **S8** (178 mg, 0.729 mmol) was dissolved in acetone (5.0 mL). Propargyl bromide (152 µL , 2.19 mmol), Cs_2CO_3 (712 mg, 2.19 mmol), and potassium iodide (36 mg, 0.22) were added to the solution. The reaction mixture was stirred at reflux in an oil bath until the starting material was fully consumed as monitored by TLC. After cooled to room temperature, the reaction mixture was concentrated under vacuum and diluted with DI water (20 mL). The aqueous layer was then extracted with CH_2Cl_2 (3 x 20 mL). The combined organic layers were over Na_2SO_4 and then concentrated under vacuum. The crude material was purified with column chromatography using 90:10 hexanes : EtOAc to yield compound **5h** in 79% yield (163 mg, 0.557 mmol) as a yellow solid.

Rf: 0.4 in 80:20 hexanes : EtOAc

^{1}H NMR: (400 MHz, $CDCl_3$) δ = 7.89 – 7.85 (m, 1H), 7.73 – 7.69 (m, 1H), 7.53 (s, 1H), 7.42 – 7.34 (m, 2H), 4.45 (d, J = 2.4 Hz, 2H), 2.86 (t, J = 6.0 Hz, 2H), 2.66 (dd, J = 7.5, 6.1 Hz, 2H), 2.24 (t, J = 2.4 Hz, 1H), 2.18 (p, J = 6.5 Hz, 2H).

^{13}C NMR: (100 MHz, $CDCl_3$) δ = 195.2, 146.8, 141.5, 139.9, 137.2, 133.3, 126.0, 124.5, 124.2, 123.7, 122.7, 78.9, 75.4, 58.8, 38.8, 31.8, 22.7.

IR: f (cm^{-1}) = 2984, 2938, 2864, 2828, 2118, 1669, 1615, 1426, 1294, 1128, 978.

HRMS (ESI-TOF): m/z [M+H]$^+$ = 283.0787 calculated for $C_{17}H_{15}O_2S$; found 283.0793.

Compound 5i

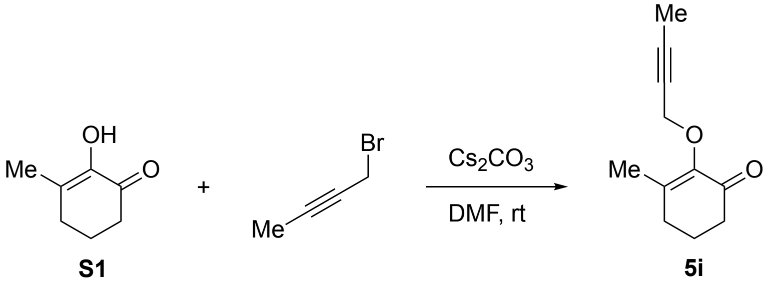

Procedure: Starting material **S1** (300 mg, 2.38 mmol) was dissolved in DMF (4.8 mL). 1-bromo-2-butyne (422 µL, 4.76 mmol) and Cs_2CO_3 (1.55 g, 4.76 mmol) were added to the solution. The reaction mixture was stirred at room temperature until the starting material was fully consumed as monitored by TLC. The reaction mixture was diluted with DI water (20 mL). The aqueous layer was then extracted with EtOAc (3 x 20 mL). The combined organic layers were dried over Na_2SO_4 and then concentrated under vacuum. The crude material was purified with column chromatography using 87.5:12.5 hexanes : EtOAc to yield compound **5i** in 84% yield (357 mg, 2.00 mmol) as a yellow oil.

Rf: 0.3 in 80:20 Hexanes : EtOAc

^{1}H NMR: (400 MHz, $CDCl_3$) δ = 4.51 (m, 2H), 2.42 (p, J = 6.6 Hz, 4H), 1.98 – 1.91 (m, 5H), 1.81 (td, J = 2.5, 0.8 Hz, 3H).

^{13}C NMR: (100 MHz, $CDCl_3$) δ = 194.8, 148.1, 146.7, 82.9, 75.0, 59.6, 38.5, 31.5, 22.0, 18.2, 3.5.

IR: f (cm^{-1}) = 3455, 2939, 2923, 2874, 1671, 1631, 1381, 1303, 1133.

HRMS (ESI-TOF): m/z [M+H]$^+$ = 179.1067 calculated for $C_{11}H_{15}O_2$; found 179.1075.

Compound 5j

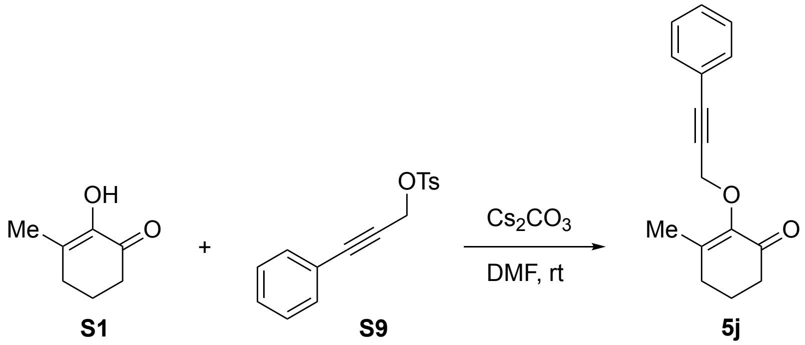

Procedure: Starting material **S1** (300 mg, 2.38 mmol) was dissolved in DMF (4.8 mL). Propargylic tosylate **S9** (1.36 g, 4.76 mmol) and Cs_2CO_3 (1.55 g, 4.76 mmol) were added to the solution. The reaction mixture was then stirred at room temperature until the starting material was fully consumed as monitored by TLC. The reaction mixture was diluted with DI water (20 mL). The aqueous layer was then extracted with EtOAc (3 x 20 mL). The combined organic layers were dried over Na_2SO_4 and then concentrated under vacuum. The crude material was purified with column chromatography using 70% CH_2Cl_2 : hexanes then 90:10 hexanes : EtOAc to 80:20 hexanes : EtOAc afford **5j** in 78% yield (444 mg, 1.85 mmol) as a yellow oil.

Rf: 0.5 in 80:20 Hexanes : EtOAc

^{1}H NMR: (400 MHz, $CDCl_3$) δ = 7.33 (m, 5H), 4.82 (s, 2H), 2.45 (dt, *J* = 19.9, 6.7 Hz, 4H), 2.03 (s, 3H), 1.96 (p, *J* = 6.4 Hz, 2H).

^{13}C NMR: (100 MHz, $CDCl_3$) δ = 194.7, 148.5, 146.5, 131.5, 128.4, 128.3, 122.6, 86.8, 85.0, 59.6, 38.6, 31.6, 22.1, 18.4.

IR: *f* (cm^{-1}) = 3433, 3079, 3064, 2935, 2869, 1673, 1443, 1380, 1147, 758.

HRMS (ESI-TOF): *m/z* $[M+H]^+$ = 241.1223 calculated for $C_{16}H_{17}O_2$; found 241.1232.

Compound 5k

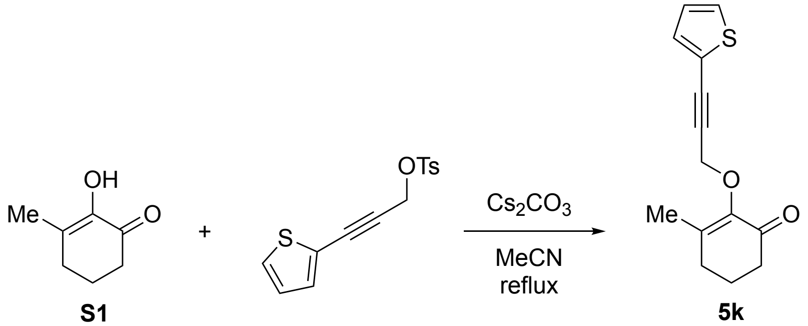

Procedure: Starting material **S1** (501 mg, 3.96 mmol) was dissolved in MeCN (8.0 mL). Propargylic tosylate **S10** (1.74 g, 5.95 mmol) and Cs_2CO_3 (3.87 g, 11.9 mmol) were added to the solution. The reaction mixture was stirred at reflux in an oil bath until the starting material was fully consumed as monitored by TLC. After cooled to room temperature, the reaction mixture was concentrated under vacuum and diluted with DI water (20 mL). The aqueous layer was then extracted with EtOAc (3 x 20 mL). The combined organic layers were dried over Na_2SO_4 and then concentrated under vacuum. The crude material was purified with column chromatography using 90:10 hexanes : EtOAc to afford **5k** in 59% yield (580 mg, 2.53 mmol) as an orange oil.

Rf: 0.5 in 70:30 Hexanes : EtOAc

^{1}H NMR: (500 MHz, $CDCl_3$) δ = 7.25 (dd, *J* = 5.2, 1.2 Hz, 1H), 7.17 (dd, J = 5.2, 1.2 Hz, 1H), 6.96 (dd, *J* = 5.2, 3.6 Hz, 1H), 4.82 (s, 2H) , 2.47 (dd, *J* = 7.5, 5.3 Hz, 2H) , 2.42 (dd, *J* = 6.5, 2.5 Hz, 2H), 2.01 (t, *J* = 1.0 Hz, 3H), 1.99 – 1.94 (m, 2H).

^{13}C NMR: (125 MHz, $CDCl_3$) δ =194.6, 148.6, 146.4, 132.1, 127.3, 127.0, 122.5, 89.1, 80.1, 59.6, 38.6, 31.6, 22.1, 18.4.

IR: *f* (cm^{-1}) = 3103, 2886, 2827, 2219, 1669, 1379, 1189, 1129, 848.

HRMS (ESI-TOF): *m/z* $[M+H]^+$ = 247.0787 calculated for $C_{14}H_{15}O_2S$; found 247.0806.

Compound 5l

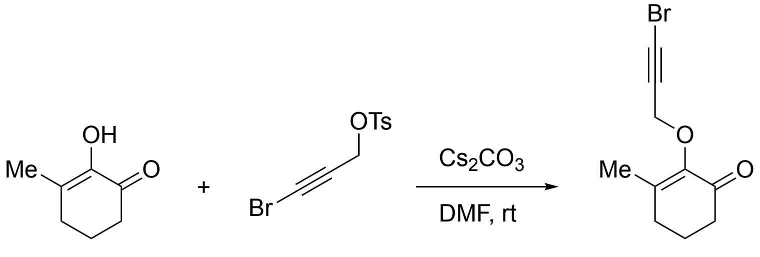

Procedure: Starting material **S1** (500 mg, 3.96 mmol) was dissolved in DMF (7.9 mL). Propargylic tosylate **S11** (2.29 g, 7.93 mmol) and Cs_2CO_3 (2.58 g, 7.93 mmol) were added to the solution. The reaction mixture was stirred at room temperature until the starting material was fully consumed as monitored by TLC. The reaction mixture was diluted with DI water (20 mL). The aqueous layer was then extracted with EtOAc (3 x 20 mL). The combined organic layers were dried over Na_2SO_4 and then concentrated under vacuum. The crude material was purified with column chromatography using 85:15 hexanes : EtOAc afford **5l** in 35% yield (340 mg, 1.40 mmol) as a yellow oil.

Rf: 0.5 in 80:20 Hexanes : EtOAc

^{1}H NMR: (400 MHz, $CDCl_3$) δ = 4.61 (s, 2H), 2.46 – 2.38 (m, 4H), 1.98 – 1.91 (m, 5H).

^{13}C NMR: (100 MHz, $CDCl_3$) δ = 194.3, 148.3, 146.2, 76.1, 59.7, 46.5, 38.5, 31.5, 22.0, 18.2.

IR: *f* (cm^{-1}) = 2938, 2869, 1670, 1632, 1430, 1360, 1191, 971.

HRMS (ESI-TOF): *m/z* $[M+H]^+$ = 243.0015 calculated for $C_{10}H_{12}BrO_2$; found 243.0025.

Compound (+)-8

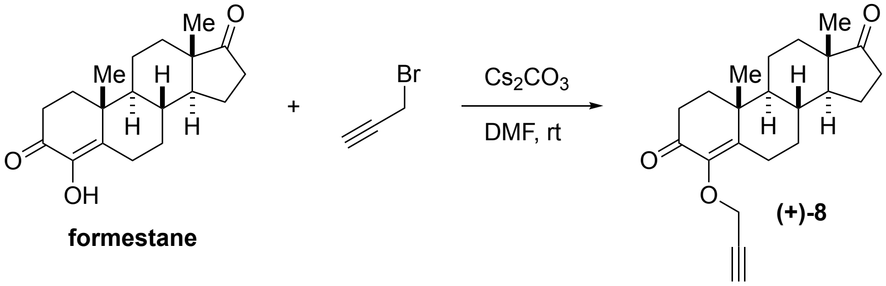

Procedure: Formestane (303 mg, 1.00 mmol) was dissolved in DMF (10 mL). Propargyl bromide (223 µL, 2.00 mmol) and Cs_2CO_3 (653 mg, 2.00 mmol) were added to the solution. The reaction mixture was stirred at room temperature until the starting material was fully consumed as monitored by TLC. The reaction mixture was diluted with DI water (20 mL). The aqueous layer was then extracted with EtOAc (3 x 20 mL). The combined organic layers were dried over Na_2SO_4 and then concentrated under vacuum. The crude material was purified with column chromatography using 80:20 hexanes : EtOAc to yield compound **(+)-8** in 83% yield (283 mg, 0.827 mmol) as a white solid.

Rf: 0.5 in 80:20 hexanes : EtOAc

^{1}H NMR: (400 MHz, $CDCl_3$) δ = 4.61 (t, *J* = 2.5 Hz, 2H), 3.27 (dd, *J* = 14.3, 1.7 Hz, 1H), 2.56 – 2.37 (m, 4H), 2.10 (ddd, *J* = 19.2, 9.0, 9.0 Hz, 1H), 2.04 – 1.93 (m, 4H), 1.87 (dt, *J* = 12.7, 3.2 Hz, 1H), 1.77 – 1.65 (m, 3H), 1.63 – 1.50 (m, 2H), 1.43 (qd, *J* = 13.1, 4.2 Hz, 1H), 1.33 – 1.21 (m, 4H), 1.12 – 0.98 (m, 2H), 0.92 (s, 3H).

^{13}C NMR: (100 MHz, $CDCl_3$) δ = 220.3, 193.7, 156.4, 143.6, 79.5, 75.0, 59.0, 54.2, 50.9, 47.4, 39.0, 35.7, 34.7, 34.7, 34.1, 31.3, 30.4, 23.8, 21.7, 20.3, 17.6, 13.7.

IR: *f* (cm^{-1}) = 3252, 2941, 2888, 2857, 1735, 1673, 1608, 1451, 1370, 1087.

HRMS (ESI-TOF): *m/z* $[M+H]^+$ = 341.2111 calculated for $C_{22}H_{29}O_3$; found 341.2120.

Specific Rotation: $[\alpha]_{25}^{D}$ = +49 (c = 1.0 in $CHCl_3$)

Compound 13

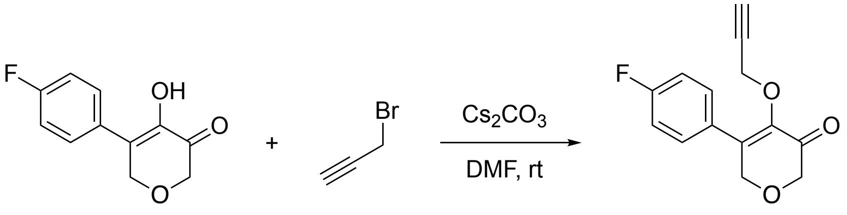

Procedure: Starting material **S12** (373 mg, 1.79 mmol) was dissolved in DMF (18 mL). Propargyl bromide (399 µL, 3.58 mmol) and Cs_2CO_3 (1.17 g, 3.58 mmol) were added to the solution. The reaction mixture was stirred at room temperature until the starting material was fully consumed as monitored by TLC. The reaction mixture was diluted with DI water (20 mL). The aqueous layer was then extracted with EtOAc (3 x 20 mL). The combined organic layers were dried over Na_2SO_4 and then concentrated under vacuum. The crude material was purified with column chromatography using 85:15 hexanes : EtOAc to yield compound **13** in 27% yield (121 mg, 0.490 mmol) as a yellow oil.

Rf: 0.4 in 80:20 Hexanes : EtOAc

^{1}H NMR: (400 MHz, $CDCl_3$) δ = 7.60 – 7.55 (m, 2H), 7.14 – 7.07 (m, 2H), 4.74 (s, 2H), 4.70 (d, J = 2.4 Hz, 2H), 4.30 (s, 2H), 2.41 (t, J = 2.4 Hz, 1H).

^{13}C NMR: (100 MHz, $CDCl_3$) δ = 191.4, 164.5, 162.0, 143.5, 141.5, 130.5, 130.4, 128.5, 128.5, 115.7, 115.5, 78.4, 76.1, 73.0, 68.5, 59.1.

IR: f (cm^{-1}) = 3298, 3064, 2961, 2925, 2851, 2819, 1685, 1327, 1239, 835.

HRMS (ESI-TOF): *m/z* $[M+H]^+$ = 247.0765 calculated for $C_{14}H_{12}FO_3$ found 247.0770.

X-Ray Crystallography Data

Compound (+)-6g

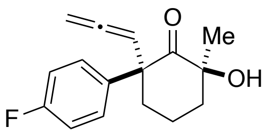

(+)-6g

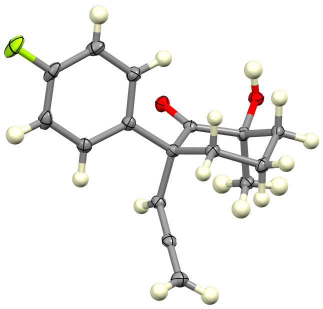

Sample Name: CCDC 2426289 / Armend7
Crystal Growth: Slow evaporation using a mixture of hexanes and dichloromethane
Ellipsoid Contour: Set at a 50% probability level

Crystal data

$C_{16}H_{17}FO_2$	D_x = 1.288 Mg m^{-3}
M_r = 260.29	Cu *K*a radiation, l = 1.54184 Å
Orthorhombic, $P2_12_12_1$	Cell parameters from 9931 reflections
a = 6.3225 (7) Å	q = 2.9–79.6°
b = 13.9838 (16) Å	m = 0.76 mm^{-1}
c = 15.1812 (17) Å	*T* = 100 K
V = 1342.2 (3) Å^3	Needle fragment, colourless
Z = 4	0.47 × 0.45 × 0.41 mm
F(000) = 552	

Data collection

Bruker D8 Venture DUO with Photon III C14 diffractometer	2823 reflections with *I* > 2s(*I*)
Radiation source: ImS 3.0 microfocus	R_{int} = 0.028
f and w scans	q_{max} = 79.6°, q_{min} = 5.8°
Absorption correction: multi-scan *SADABS* (Krause *et al.*, 2015)	*h* = -8 - 7
T_{min} = 0.642, T_{max} = 0.745	*k* = -17 - 16
16221 measured reflections	*l* = -19 - 19
2826 independent reflections	

Refinement

Refinement on F^2	H atoms treated by a mixture of independent and constrained refinement
Least-squares matrix: full	$w = 1/[s^2(F_o^2) + (0.033P)^2 + 0.3062P]$ where $P = (F_o^2 + 2F_c^2)/3$
$R[F^2 > 2s(F^2)] = 0.027$	$(D/s)_{max} = 0.001$
$wR(F^2) = 0.070$	$Dr_{max} = 0.24$ e Å^{-3}
$S = 1.09$	$Dr_{min} = -0.16$ e Å^{-3}
2826 reflections	Extinction correction: *SHELXL2019*/1 (Sheldrick 2019), $Fc^* = kFc[1+0.001xFc^2l^3/\sin(2q)]^{-1/4}$
183 parameters	Extinction coefficient: 0.0236 (15)
0 restraints	Absolute structure: Flack x determined using 1137 quotients [(I+)-(I-)]/[(I+)+(I-)] (Parsons, Flack and Wagner, Acta Cryst. B69 (2013) 249-259).
Hydrogen site location: mixed	Absolute structure parameter: 0.04 (3)

Fractional atomic coordinates and isotropic or equivalent isotropic displacement parameters (Å^2) for (Armend7)

	x	*y*	*z*	U_{iso}*/U_{eq}
F1	0.7534 (2)	0.30449 (7)	0.52221 (6)	0.0347 (3)
O1	0.57851 (17)	0.72715 (7)	0.48913 (7)	0.0186 (3)
O2	0.74127 (18)	0.89796 (7)	0.44298 (7)	0.0189 (2)
H20H	0.854 (4)	0.8732 (16)	0.4657 (14)	0.028*
C1	0.6125 (2)	0.73785 (10)	0.41081 (9)	0.0146 (3)
C2	0.6655 (2)	0.83823 (10)	0.37401 (9)	0.0155 (3)
C3	0.8298 (2)	0.83132 (10)	0.29983 (10)	0.0183 (3)
H3A	0.847205	0.895058	0.272378	0.022*
H3B	0.967839	0.812444	0.325261	0.022*
C4	0.7673 (3)	0.75935 (10)	0.22919 (9)	0.0182 (3)
H4A	0.875127	0.758939	0.181919	0.022*
H4B	0.630289	0.778265	0.202825	0.022*
C5	0.7483 (2)	0.65963 (10)	0.26884 (9)	0.0163 (3)
H5A	0.889175	0.639339	0.290291	0.020*
H5B	0.704846	0.614493	0.221977	0.020*
C6	0.5887 (2)	0.65274 (10)	0.34564 (9)	0.0150 (3)
C7	0.3598 (2)	0.65935 (11)	0.31314 (9)	0.0164 (3)
H7	0.252805	0.673329	0.355167	0.020*
C8	0.3023 (2)	0.64693 (10)	0.23111 (10)	0.0173 (3)

C9	0.2458 (3)	0.63545 (12)	0.14968 (10)	0.0218 (3)
H9A	0.213 (4)	0.5729 (15)	0.1249 (14)	0.026*
H9B	0.243 (4)	0.6898 (15)	0.1108 (13)	0.026*
C10	0.4596 (2)	0.88425 (11)	0.34191 (10)	0.0195 (3)
H10A	0.406929	0.849328	0.290424	0.029*
H10B	0.486395	0.951001	0.325723	0.029*
H10C	0.353849	0.881863	0.389043	0.029*
C11	0.6250 (3)	0.55874 (10)	0.39516 (9)	0.0162 (3)
C12	0.8199 (3)	0.54403 (11)	0.43673 (10)	0.0193 (3)
H12	0.923041	0.593437	0.435928	0.023*
C13	0.8653 (3)	0.45828 (12)	0.47919 (10)	0.0238 (3)
H13	0.998236	0.448199	0.506813	0.029*
C14	0.7117 (3)	0.38845 (11)	0.48001 (10)	0.0245 (4)
C15	0.5176 (3)	0.39972 (11)	0.44079 (10)	0.0232 (3)
H15	0.414846	0.350188	0.442876	0.028*
C16	0.4750 (3)	0.48597 (11)	0.39772 (9)	0.0187 (3)
H16	0.341995	0.495010	0.369831	0.022*

Atomic displacement parameters (Å^2) for (Armend7)

	U^{11}	U^{22}	U^{33}	U^{12}	U^{13}	U^{23}
F1	0.0617 (7)	0.0145 (4)	0.0278 (5)	0.0047 (5)	-0.0073 (5)	0.0062 (4)
O1	0.0204 (6)	0.0199 (5)	0.0155 (5)	-0.0001 (4)	0.0022 (4)	0.0005 (4)
O2	0.0201 (5)	0.0152 (5)	0.0213 (5)	-0.0002 (4)	-0.0041 (5)	-0.0011 (4)
C1	0.0106 (6)	0.0159 (7)	0.0172 (6)	0.0018 (5)	-0.0003 (5)	0.0015 (5)
C2	0.0172 (7)	0.0131 (6)	0.0163 (6)	0.0005 (6)	-0.0009 (5)	0.0006 (5)
C3	0.0167 (7)	0.0184 (7)	0.0197 (7)	-0.0017 (6)	0.0018 (6)	0.0047 (5)
C4	0.0188 (7)	0.0203 (7)	0.0156 (6)	0.0014 (6)	0.0031 (6)	0.0035 (5)
C5	0.0159 (6)	0.0171 (6)	0.0159 (6)	0.0025 (6)	0.0009 (5)	0.0004 (5)
C6	0.0137 (6)	0.0150 (6)	0.0162 (6)	-0.0005 (6)	-0.0003 (5)	0.0003 (5)
C7	0.0141 (6)	0.0173 (6)	0.0178 (7)	0.0009 (6)	0.0004 (5)	-0.0004 (5)
C8	0.0123 (6)	0.0170 (6)	0.0227 (7)	0.0010 (5)	0.0003 (5)	0.0008 (5)
C9	0.0198 (7)	0.0262 (8)	0.0194 (7)	0.0013 (6)	-0.0013	-0.0009

					(6)	(6)
C10	0.0188 (7)	0.0171 (7)	0.0224 (7)	0.0014 (6)	-0.0030 (6)	0.0018 (6)
C11	0.0198 (8)	0.0143 (7)	0.0144 (6)	-0.0002 (5)	0.0003 (6)	-0.0011 (5)
C12	0.0196 (7)	0.0173 (7)	0.0211 (7)	0.0002 (6)	-0.0021 (6)	0.0008 (6)
C13	0.0299 (8)	0.0199 (7)	0.0217 (7)	0.0054 (6)	-0.0056 (7)	0.0008 (6)
C14	0.0450 (10)	0.0123 (7)	0.0163 (7)	0.0038 (7)	-0.0017 (7)	0.0013 (5)
C15	0.0364 (9)	0.0158 (7)	0.0174 (7)	-0.0057 (6)	0.0013 (7)	-0.0011 (6)
C16	0.0227 (8)	0.0178 (7)	0.0155 (7)	-0.0029 (6)	0.0000 (6)	-0.0021 (5)

Geometric parameters (Å, º) for (Armend7)

F1—C14	1.3632 (17)	C7—C8	1.309 (2)
O1—C1	1.2174 (18)	C7—H7	0.9500
O2—C2	1.4226 (17)	C8—C9	1.297 (2)
O2—H20H	0.86 (2)	C9—H9A	0.98 (2)
C1—C2	1.5475 (19)	C9—H9B	0.96 (2)
C1—C6	1.555 (2)	C10—H10A	0.9800
C2—C10	1.531 (2)	C10—H10B	0.9800
C2—C3	1.535 (2)	C10—H10C	0.9800
C3—C4	1.523 (2)	C11—C16	1.392 (2)
C3—H3A	0.9900	C11—C12	1.400 (2)
C3—H3B	0.9900	C12—C13	1.391 (2)
C4—C5	1.524 (2)	C12—H12	0.9500
C4—H4A	0.9900	C13—C14	1.377 (3)
C4—H4B	0.9900	C13—H13	0.9500
C5—C6	1.5447 (19)	C14—C15	1.373 (3)
C5—H5A	0.9900	C15—C16	1.398 (2)
C5—H5B	0.9900	C15—H15	0.9500
C6—C11	1.532 (2)	C16—H16	0.9500
C6—C7	1.5322 (19)		
C2—O2—H20H	109.6 (15)	C5—C6—C1	111.68 (12)
O1—C1—C2	120.15 (12)	C8—C7—C6	124.09 (13)
O1—C1—C6	120.68 (13)	C8—C7—H7	118.0

C2—C1—C6	119.04 (11)	C6—C7—H7	118.0
O2—C2—C10	105.89 (11)	C9—C8—C7	179.48 (17)
O2—C2—C3	110.41 (12)	C8—C9—H9A	122.6 (12)
C10—C2—C3	111.60 (12)	C8—C9—H9B	119.5 (12)
O2—C2—C1	109.86 (11)	H9A—C9—H9B	117.8 (17)
C10—C2—C1	108.19 (12)	C2—C10—H10A	109.5
C3—C2—C1	110.75 (11)	C2—C10—H10B	109.5
C4—C3—C2	112.47 (12)	H10A—C10—H10B	109.5
C4—C3—H3A	109.1	C2—C10—H10C	109.5
C2—C3—H3A	109.1	H10A—C10—H10C	109.5
C4—C3—H3B	109.1	H10B—C10—H10C	109.5
C2—C3—H3B	109.1	C16—C11—C12	118.69 (13)
H3A—C3—H3B	107.8	C16—C11—C6	122.62 (14)
C3—C4—C5	110.31 (12)	C12—C11—C6	118.65 (13)
C3—C4—H4A	109.6	C13—C12—C11	121.13 (15)
C5—C4—H4A	109.6	C13—C12—H12	119.4
C3—C4—H4B	109.6	C11—C12—H12	119.4
C5—C4—H4B	109.6	C14—C13—C12	118.03 (15)
H4A—C4—H4B	108.1	C14—C13—H13	121.0
C4—C5—C6	114.00 (12)	C12—C13—H13	121.0
C4—C5—H5A	108.8	F1—C14—C15	118.39 (15)
C6—C5—H5A	108.8	F1—C14—C13	118.58 (16)
C4—C5—H5B	108.8	C15—C14—C13	123.02 (14)
C6—C5—H5B	108.8	C14—C15—C16	118.28 (15)
H5A—C5—H5B	107.6	C14—C15—H15	120.9
C11—C6—C7	110.58 (12)	C16—C15—H15	120.9
C11—C6—C5	109.04 (12)	C11—C16—C15	120.84 (15)
C7—C6—C5	111.73 (11)	C11—C16—H16	119.6
C11—C6—C1	109.27 (11)	C15—C16—H16	119.6
C7—C6—C1	104.48 (11)		
O1—C1—C2—O2	21.27 (18)	C11—C6—C7—C8	-104.79 (16)
C6—C1—C2—O2	-162.77 (12)	C5—C6—C7—C8	16.9 (2)
O1—C1—C2—C10	-93.88 (16)	C1—C6—C7—C8	137.76 (15)
C6—C1—C2—C10	82.08 (15)	C7—C6—C11—C16	8.27 (19)
O1—C1—C2—C3	143.51 (13)	C5—C6—C11—C16	-114.95 (15)
C6—C1—C2—C3	-40.53 (17)	C1—C6—C11—C16	122.73 (14)

O2—C2—C3—C4	173.51 (12)	C7—C6—C11—C12	-174.19 (13)
C10—C2—C3—C4	-69.01 (15)	C5—C6—C11—C12	62.59 (16)
C1—C2—C3—C4	51.60 (16)	C1—C6—C11—C12	-59.72 (17)
C2—C3—C4—C5	-61.15 (16)	C16—C11—C12—C13	0.6 (2)
C3—C4—C5—C6	57.39 (16)	C6—C11—C12—C13	-177.01 (14)
C4—C5—C6—C11	-165.21 (12)	C11—C12—C13—C14	-0.7 (2)
C4—C5—C6—C7	72.26 (16)	C12—C13—C14—F1	-179.32 (14)
C4—C5—C6—C1	-44.36 (16)	C12—C13—C14—C15	0.2 (2)
O1—C1—C6—C11	-26.45 (18)	F1—C14—C15—C16	179.86 (13)
C2—C1—C6—C11	157.61 (13)	C13—C14—C15—C16	0.4 (2)
O1—C1—C6—C7	91.89 (16)	C12—C11—C16—C15	-0.1 (2)
C2—C1—C6—C7	-84.05 (14)	C6—C11—C16—C15	177.48 (13)
O1—C1—C6—C5	-147.17 (14)	C14—C15—C16—C11	-0.4 (2)
C2—C1—C6—C5	36.89 (17)		

Hydrogen-bond geometry (Å, º) for (Armend7)

D—H···*A*	*D*—H	H···*A*	*D*···*A*	*D*—H···*A*
O2—H20*H*···O1^{i}	0.86 (2)	2.11 (2)	2.9444 (15)	162 (2)

Symmetry code: (i) *x*+1/2, -*y*+3/2, -*z*+1.

Compound (+)-6j

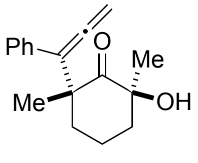

(+)-6j

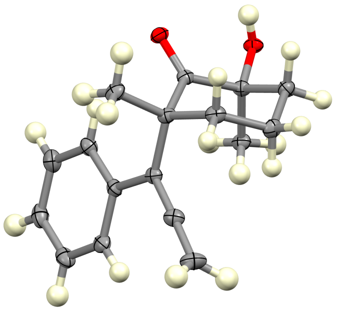

Sample Name: CCDC 2426290 / Armend8 (EAG3027P)
Crystal Growth: Slow evaporation using a mixture of hexanes and dichloromethane
Ellipsoid Contour: Set at a 50% probability level

Crystal data

$C_{17}H_{20}O_2$	$F(000) = 828$
$M_r = 256.33$	$D_x = 1.228$ Mg m^{-3}
Monoclinic, $P2_1$	Cu *Ka* radiation, l = 1.54184 Å
a = 14.8906 (6) Å	Cell parameters from 9941 reflections
b = 7.3763 (3) Å	q = 3.5–79.4°
c = 19.2990 (7) Å	m = 0.62 mm^{-1}
b = 101.153 (2)°	*T* = 100 K
V = 2079.72 (14) Å^3	Lath, colourless
Z = 6	0.24 × 0.09 × 0.04 mm

Data collection

Bruker D8 Venture DUO with Photon III C14 diffractometer	7877 reflections with $I > 2s(I)$
Radiation source: ImS 3.0 microfocus	$R_{int} = 0.107$
Absorption correction: multi-scan *SADABS* (Krause *et al.*, 2015)	$q_{max} = 79.9°$, $q_{min} = 2.3°$
$T_{min} = 0.793$, $T_{max} = 0.976$	*h* = -19 - 18
44024 measured reflections	*k* = -9 - 9
8724 independent reflections	*l* = -24 - 24

Refinement

Refinement on F^2	Hydrogen site location: mixed
Least-squares matrix: full	H atoms treated by a mixture of independent and constrained refinement

$R[F^2 > 2s(F^2)] = 0.035$	$w = 1/[s^2(F_o^2) + (0.0424P)^2 + 0.0942P]$ where $P = (F_o^2 + 2F_c^2)/3$
$wR(F^2) = 0.085$	$(D/s)_{max} < 0.001$
$S = 1.05$	$Dr_{max} = 0.20$ e Å^{-3}
8724 reflections	$Dr_{min} = -0.20$ e Å^{-3}
547 parameters	Absolute structure: Flack x determined using 3226 quotients [(I+)-(I-)]/[(I+)+(I-)] (Parsons, Flack and Wagner, Acta Cryst. B69 (2013) 249-259).
1 restraint	Absolute structure parameter: 0.04 (8)

Fractional atomic coordinates and isotropic or equivalent isotropic displacement parameters (Å^2) for (3027P)

	x	*y*	*z*	U_{iso}*/U_{eq}
O5	0.45140 (10)	0.7241 (2)	0.73373 (7)	0.0184 (3)
O6	0.33857 (10)	0.9597 (2)	0.77384 (7)	0.0184 (3)
H60H	0.317 (2)	0.864 (5)	0.7527 (16)	0.028*
C35	0.47036 (13)	0.7622 (3)	0.79615 (10)	0.0143 (4)
C36	0.41093 (14)	0.8986 (3)	0.82771 (10)	0.0150 (4)
C37	0.37217 (14)	0.8001 (3)	0.88607 (10)	0.0182 (4)
H37A	0.339908	0.889142	0.910874	0.022*
H37B	0.326844	0.708799	0.863956	0.022*
C38	0.44599 (15)	0.7062 (3)	0.93984 (10)	0.0192 (4)
H38A	0.417183	0.640170	0.974520	0.023*
H38B	0.488022	0.798128	0.965710	0.023*
C39	0.50018 (15)	0.5733 (3)	0.90323 (10)	0.0181 (4)
H39A	0.458245	0.477017	0.880551	0.022*
H39B	0.547544	0.514964	0.939434	0.022*
C40	0.54759 (14)	0.6612 (3)	0.84635 (9)	0.0150 (4)
C41	0.62816 (14)	0.7842 (3)	0.88046 (10)	0.0163 (4)
C42	0.67316 (14)	0.9118 (3)	0.83737 (10)	0.0166 (4)
C43	0.64432 (14)	0.9336 (3)	0.76421 (10)	0.0176 (4)
H43	0.595783	0.860846	0.739663	0.021*
C44	0.68568 (14)	1.0603 (3)	0.72701 (11)	0.0191 (4)
H44	0.664167	1.074386	0.677657	0.023*
C45	0.75777 (16)	1.1660 (3)	0.76101 (12)	0.0220 (4)
H45	0.785572	1.252693	0.735463	0.026*
C46	0.78883 (15)	1.1428 (3)	0.83338 (12)	0.0235 (4)
H46	0.839026	1.212622	0.857237	0.028*

C47	0.74697 (16)	1.0187 (3)	0.87078 (11)	0.0212 (4)
H47	0.768715	1.005719	0.920148	0.025*
C48	0.66153 (15)	0.7686 (3)	0.94874 (11)	0.0201 (4)
C49	0.69482 (16)	0.7558 (3)	1.01605 (12)	0.0252 (5)
H49A	0.740 (2)	0.662 (4)	1.0362 (16)	0.030*
H49B	0.670 (2)	0.841 (4)	1.0509 (16)	0.030*
C50	0.46338 (14)	1.0689 (3)	0.85591 (10)	0.0182 (4)
H50A	0.510050	1.037970	0.897387	0.027*
H50B	0.420873	1.157848	0.869254	0.027*
H50C	0.492977	1.120486	0.819244	0.027*
C51	0.58536 (16)	0.5083 (3)	0.80602 (11)	0.0206 (4)
H51A	0.626695	0.431784	0.839487	0.031*
H51B	0.618939	0.560878	0.771942	0.031*
H51C	0.534527	0.434668	0.780932	0.031*
O3	0.22967 (11)	0.4232 (2)	0.82111 (7)	0.0188 (3)
O4	0.24863 (10)	0.6425 (2)	0.71649 (7)	0.0169 (3)
H40H	0.271 (2)	0.537 (5)	0.7188 (15)	0.025*
C18	0.15794 (14)	0.4865 (3)	0.78959 (9)	0.0140 (4)
C19	0.15734 (14)	0.6142 (3)	0.72630 (10)	0.0148 (4)
C20	0.10111 (15)	0.5215 (3)	0.66048 (10)	0.0188 (4)
H20A	0.094609	0.605820	0.619915	0.023*
H20B	0.134547	0.413026	0.648946	0.023*
C21	0.00600 (15)	0.4645 (3)	0.67110 (10)	0.0188 (4)
H21A	-0.026382	0.400327	0.628468	0.023*
H21B	-0.030025	0.573558	0.677997	0.023*
C22	0.01352 (15)	0.3398 (3)	0.73555 (10)	0.0184 (4)
H22A	0.045237	0.227038	0.726265	0.022*
H22B	-0.048949	0.306276	0.741413	0.022*
C23	0.06551 (14)	0.4242 (3)	0.80594 (9)	0.0150 (4)
C24	0.00918 (14)	0.5773 (3)	0.83151 (10)	0.0154 (4)
C25	0.04826 (15)	0.6995 (3)	0.89188 (10)	0.0166 (4)
C26	0.13903 (16)	0.6904 (3)	0.92741 (10)	0.0201 (4)
H26	0.179206	0.604152	0.913104	0.024*
C27	0.17164 (16)	0.8070 (3)	0.98401 (10)	0.0229 (4)
H27	0.233867	0.799901	1.007316	0.027*
C28	0.11421 (18)	0.9324 (3)	1.00633 (11)	0.0248 (5)
H28	0.136476	1.010184	1.045079	0.030*
C29	0.02353 (18)	0.9431 (3)	0.97134 (11)	0.0247 (5)
H29	-0.016562	1.028668	0.986142	0.030*

C30	-0.00844 (16)	0.8291 (3)	0.91494 (10)	0.0202 (4)
H30	-0.070396	0.838870	0.891225	0.024*
C31	-0.07811 (15)	0.5942 (3)	0.80362 (11)	0.0193 (4)
C32	-0.16425 (17)	0.6211 (3)	0.77765 (14)	0.0280 (5)
H32A	-0.211 (2)	0.554 (5)	0.7973 (17)	0.034*
H32B	-0.185 (2)	0.709 (5)	0.7382 (17)	0.034*
C33	0.12072 (15)	0.8026 (3)	0.73862 (10)	0.0179 (4)
H33A	0.055227	0.794581	0.739382	0.027*
H33B	0.129971	0.883925	0.700450	0.027*
H33C	0.153480	0.850199	0.783917	0.027*
C34	0.08013 (16)	0.2747 (3)	0.86244 (11)	0.0202 (4)
H34A	0.021001	0.220717	0.865831	0.030*
H34B	0.108279	0.327072	0.908160	0.030*
H34C	0.120430	0.180987	0.849359	0.030*
O1	0.21530 (11)	0.0817 (2)	0.63499 (8)	0.0210 (3)
O2	0.33889 (11)	0.3057 (2)	0.70412 (7)	0.0194 (3)
H20H	0.331 (2)	0.205 (5)	0.7263 (17)	0.029*
C1	0.27925 (13)	0.1074 (3)	0.60586 (9)	0.0151 (4)
C2	0.35433 (14)	0.2451 (3)	0.63706 (10)	0.0161 (4)
C3	0.44858 (14)	0.1548 (3)	0.64634 (10)	0.0177 (4)
H3A	0.496576	0.248133	0.659705	0.021*
H3B	0.454852	0.065729	0.685359	0.021*
C4	0.46365 (14)	0.0584 (3)	0.57937 (10)	0.0183 (4)
H4A	0.524420	-0.000955	0.588319	0.022*
H4B	0.462274	0.148058	0.540990	0.022*
C5	0.38884 (15)	-0.0837 (3)	0.55690 (10)	0.0186 (4)
H5A	0.392241	-0.174321	0.595161	0.022*
H5B	0.400902	-0.147605	0.514524	0.022*
C6	0.29095 (14)	-0.0043 (3)	0.54014 (10)	0.0157 (4)
C7	0.27473 (14)	0.1114 (3)	0.47215 (10)	0.0165 (4)
C8	0.19531 (14)	0.2383 (3)	0.45397 (10)	0.0159 (4)
C9	0.19392 (16)	0.3652 (3)	0.39985 (11)	0.0222 (4)
H9	0.244107	0.370572	0.376111	0.027*
C10	0.12116 (18)	0.4828 (3)	0.38014 (12)	0.0272 (5)
H10	0.121715	0.566569	0.342848	0.033*
C11	0.04742 (16)	0.4795 (3)	0.41432 (11)	0.0241 (4)
H11	-0.002710	0.559978	0.400741	0.029*
C12	0.04834 (16)	0.3569 (3)	0.46852 (11)	0.0233 (4)
H12	-0.001353	0.354628	0.492842	0.028*

C13	0.12075 (15)	0.2365 (3)	0.48815 (10)	0.0207 (4)
H13	0.119466	0.152176	0.525161	0.025*
C14	0.32878 (15)	0.0877 (3)	0.42641 (10)	0.0208 (4)
C15	0.38223 (19)	0.0598 (4)	0.38138 (12)	0.0303 (5)
H15A	0.435 (3)	0.139 (5)	0.3810 (17)	0.036*
H15B	0.368 (2)	-0.054 (5)	0.3472 (18)	0.036*
C16	0.34812 (15)	0.4147 (3)	0.59114 (10)	0.0182 (4)
H16A	0.366850	0.385212	0.546498	0.027*
H16B	0.388594	0.508560	0.615945	0.027*
H16C	0.284915	0.459188	0.581490	0.027*
C17	0.22371 (16)	-0.1639 (3)	0.52986 (12)	0.0232 (4)
H17A	0.239141	-0.246685	0.494207	0.035*
H17B	0.161275	-0.118322	0.514123	0.035*
H17C	0.227738	-0.228697	0.574690	0.035*

Atomic displacement parameters (Å^2) for (3027P)

	U^{11}	U^{22}	U^{33}	U^{12}	U^{13}	U^{23}
O5	0.0182 (7)	0.0203 (7)	0.0160 (6)	-0.0042 (6)	0.0017 (5)	-0.0029 (5)
O6	0.0172 (7)	0.0155 (7)	0.0204 (6)	0.0000 (6)	-0.0018 (5)	0.0017 (5)
C35	0.0156 (9)	0.0106 (8)	0.0165 (8)	-0.0050 (7)	0.0025 (7)	0.0002 (7)
C36	0.0146 (9)	0.0137 (9)	0.0151 (8)	-0.0004 (7)	-0.0006 (7)	0.0011 (7)
C37	0.0181 (9)	0.0190 (10)	0.0183 (8)	-0.0022 (8)	0.0054 (7)	-0.0002 (7)
C38	0.0236 (10)	0.0194 (9)	0.0150 (8)	-0.0033 (8)	0.0046 (7)	0.0016 (7)
C39	0.0224 (10)	0.0128 (9)	0.0188 (8)	-0.0021 (8)	0.0034 (7)	0.0030 (7)
C40	0.0161 (9)	0.0133 (9)	0.0148 (8)	-0.0018 (7)	0.0009 (7)	-0.0004 (7)
C41	0.0157 (9)	0.0138 (9)	0.0184 (8)	0.0000 (7)	0.0011 (7)	-0.0001 (7)
C42	0.0154 (9)	0.0146 (9)	0.0191 (8)	-0.0003 (7)	0.0021 (7)	-0.0020 (7)
C43	0.0148 (9)	0.0170 (9)	0.0204 (9)	-0.0019 (7)	0.0020 (7)	-0.0016 (7)
C44	0.0167 (9)	0.0202 (10)	0.0210 (9)	0.0008 (8)	0.0049 (7)	0.0004 (8)

C45	0.0203 (10)	0.0182 (10)	0.0288 (10)	-0.0023 (8)	0.0081 (8)	-0.0008 (8)
C46	0.0198 (10)	0.0205 (10)	0.0292 (10)	-0.0060 (8)	0.0021 (8)	-0.0049 (8)
C47	0.0201 (10)	0.0201 (10)	0.0218 (9)	-0.0027 (8)	0.0006 (8)	-0.0031 (8)
C48	0.0187 (10)	0.0180 (9)	0.0227 (9)	-0.0023 (8)	0.0017 (8)	0.0000 (8)
C49	0.0240 (11)	0.0280 (11)	0.0210 (9)	-0.0042 (9)	-0.0023 (8)	0.0020 (9)
C50	0.0201 (10)	0.0133 (9)	0.0208 (9)	-0.0017 (8)	0.0030 (7)	-0.0032 (7)
C51	0.0232 (10)	0.0146 (9)	0.0235 (9)	0.0013 (8)	0.0035 (8)	-0.0020 (7)
O3	0.0170 (7)	0.0204 (7)	0.0181 (6)	0.0028 (6)	0.0009 (5)	0.0010 (5)
O4	0.0145 (7)	0.0150 (7)	0.0220 (6)	-0.0001 (6)	0.0053 (5)	-0.0002 (5)
C18	0.0171 (9)	0.0102 (8)	0.0142 (8)	0.0003 (7)	0.0023 (7)	-0.0032 (6)
C19	0.0151 (9)	0.0130 (9)	0.0167 (8)	-0.0014 (7)	0.0039 (7)	0.0006 (7)
C20	0.0196 (10)	0.0204 (10)	0.0161 (8)	0.0000 (8)	0.0025 (7)	-0.0013 (7)
C21	0.0175 (10)	0.0206 (10)	0.0168 (8)	-0.0021 (8)	-0.0003 (7)	-0.0023 (7)
C22	0.0175 (9)	0.0154 (9)	0.0213 (9)	-0.0033 (7)	0.0013 (7)	-0.0029 (7)
C23	0.0170 (9)	0.0113 (8)	0.0168 (8)	-0.0015 (7)	0.0033 (7)	0.0010 (7)
C24	0.0188 (9)	0.0119 (8)	0.0163 (8)	-0.0009 (7)	0.0052 (7)	0.0017 (7)
C25	0.0218 (10)	0.0134 (9)	0.0157 (8)	-0.0006 (8)	0.0060 (7)	0.0016 (7)
C26	0.0229 (10)	0.0202 (10)	0.0177 (8)	-0.0010 (8)	0.0056 (8)	-0.0014 (8)
C27	0.0287 (11)	0.0231 (10)	0.0166 (8)	-0.0049 (9)	0.0040 (8)	-0.0003 (8)
C28	0.0392 (13)	0.0192 (10)	0.0166 (8)	-0.0040 (9)	0.0067 (8)	-0.0028 (8)
C29	0.0389 (13)	0.0162 (10)	0.0211 (9)	0.0022 (9)	0.0109 (9)	0.0006 (8)
C30	0.0272	0.0147 (9)	0.0200 (9)	0.0022 (8)	0.0074 (8)	0.0029 (8)

	(11)					
C31	0.0213 (10)	0.0141 (9)	0.0234 (9)	-0.0012 (8)	0.0066 (8)	-0.0018 (7)
C32	0.0200 (11)	0.0237 (11)	0.0393 (12)	0.0030 (9)	0.0033 (9)	-0.0038 (10)
C33	0.0201 (10)	0.0126 (9)	0.0222 (9)	0.0011 (7)	0.0071 (7)	0.0024 (7)
C34	0.0234 (10)	0.0140 (9)	0.0230 (9)	0.0000 (8)	0.0040 (8)	0.0036 (8)
O1	0.0196 (7)	0.0220 (7)	0.0223 (6)	0.0006 (6)	0.0060 (5)	0.0065 (6)
O2	0.0262 (8)	0.0162 (7)	0.0169 (6)	0.0018 (6)	0.0067 (5)	-0.0003 (5)
C1	0.0156 (9)	0.0116 (8)	0.0173 (8)	0.0023 (7)	0.0008 (7)	0.0052 (7)
C2	0.0195 (10)	0.0116 (9)	0.0175 (8)	0.0009 (7)	0.0045 (7)	0.0003 (7)
C3	0.0153 (9)	0.0176 (9)	0.0192 (8)	-0.0003 (8)	0.0009 (7)	0.0003 (7)
C4	0.0166 (9)	0.0177 (9)	0.0206 (9)	0.0035 (8)	0.0035 (7)	-0.0016 (8)
C5	0.0199 (10)	0.0132 (9)	0.0222 (9)	0.0044 (8)	0.0024 (7)	0.0006 (7)
C6	0.0163 (9)	0.0116 (8)	0.0187 (8)	0.0017 (7)	0.0022 (7)	0.0016 (7)
C7	0.0161 (9)	0.0157 (9)	0.0169 (8)	0.0006 (7)	0.0010 (7)	0.0004 (7)
C8	0.0171 (9)	0.0142 (9)	0.0156 (8)	0.0008 (7)	0.0010 (7)	-0.0001 (7)
C9	0.0234 (11)	0.0215 (10)	0.0230 (9)	0.0043 (9)	0.0078 (8)	0.0059 (8)
C10	0.0299 (12)	0.0250 (11)	0.0273 (10)	0.0072 (10)	0.0067 (9)	0.0115 (9)
C11	0.0234 (11)	0.0229 (10)	0.0239 (10)	0.0078 (9)	-0.0006 (8)	0.0014 (8)
C12	0.0187 (10)	0.0280 (11)	0.0228 (9)	0.0051 (9)	0.0030 (8)	0.0019 (8)
C13	0.0195 (10)	0.0225 (10)	0.0194 (8)	0.0013 (8)	0.0019 (8)	0.0058 (8)
C14	0.0211 (10)	0.0216 (10)	0.0188 (9)	0.0058 (8)	0.0019 (7)	0.0020 (8)
C15	0.0301 (12)	0.0365 (13)	0.0262 (10)	0.0131 (11)	0.0103 (9)	0.0076 (10)
C16	0.0236 (10)	0.0114 (8)	0.0198 (8)	-0.0003 (8)	0.0047 (8)	0.0030 (7)
C17	0.0223	0.0155 (9)	0.0301	-0.0035	0.0013 (8)	0.0017 (8)

	(10)		(10)	(8)		

Geometric parameters (Å, º) for (3027P)

O5—C35	1.216 (2)	C25—C26	1.394 (3)
O6—C36	1.418 (2)	C25—C30	1.403 (3)
O6—H60H	0.85 (3)	C26—C27	1.401 (3)
C35—C36	1.541 (3)	C26—H26	0.9500
C35—C40	1.545 (3)	C27—C28	1.384 (3)
C36—C50	1.523 (3)	C27—H27	0.9500
C36—C37	1.543 (3)	C28—C29	1.390 (4)
C37—C38	1.525 (3)	C28—H28	0.9500
C37—H37A	0.9900	C29—C30	1.385 (3)
C37—H37B	0.9900	C29—H29	0.9500
C38—C39	1.527 (3)	C30—H30	0.9500
C38—H38A	0.9900	C31—C32	1.299 (3)
C38—H38B	0.9900	C32—H32A	0.99 (4)
C39—C40	1.557 (3)	C32—H32B	1.00 (3)
C39—H39A	0.9900	C33—H33A	0.9800
C39—H39B	0.9900	C33—H33B	0.9800
C40—C51	1.537 (3)	C33—H33C	0.9800
C40—C41	1.546 (3)	C34—H34A	0.9800
C41—C48	1.320 (3)	C34—H34B	0.9800
C41—C42	1.497 (3)	C34—H34C	0.9800
C42—C43	1.403 (3)	O1—C1	1.211 (3)
C42—C47	1.404 (3)	O2—C2	1.429 (2)
C43—C44	1.392 (3)	O2—H20H	0.88 (4)
C43—H43	0.9500	C1—C2	1.544 (3)
C44—C45	1.385 (3)	C1—C6	1.550 (3)
C44—H44	0.9500	C2—C16	1.525 (3)
C45—C46	1.394 (3)	C2—C3	1.532 (3)
C45—H45	0.9500	C3—C4	1.529 (3)
C46—C47	1.386 (3)	C3—H3A	0.9900
C46—H46	0.9500	C3—H3B	0.9900
C47—H47	0.9500	C4—C5	1.530 (3)
C48—C49	1.301 (3)	C4—H4A	0.9900
C49—H49A	0.99 (3)	C4—H4B	0.9900
C49—H49B	1.04 (3)	C5—C6	1.546 (3)
C50—H50A	0.9800	C5—H5A	0.9900
C50—H50B	0.9800	C5—H5B	0.9900

C50—H50C	0.9800	C6—C17	1.533 (3)
C51—H51A	0.9800	C6—C7	1.545 (3)
C51—H51B	0.9800	C7—C14	1.316 (3)
C51—H51C	0.9800	C7—C8	1.496 (3)
O3—C18	1.215 (3)	C8—C13	1.397 (3)
O4—C19	1.424 (2)	C8—C9	1.399 (3)
O4—H40H	0.84 (3)	C9—C10	1.383 (3)
C18—C23	1.540 (3)	C9—H9	0.9500
C18—C19	1.541 (3)	C10—C11	1.386 (4)
C19—C33	1.528 (3)	C10—H10	0.9500
C19—C20	1.540 (3)	C11—C12	1.381 (3)
C20—C21	1.529 (3)	C11—H11	0.9500
C20—H20A	0.9900	C12—C13	1.392 (3)
C20—H20B	0.9900	C12—H12	0.9500
C21—C22	1.533 (3)	C13—H13	0.9500
C21—H21A	0.9900	C14—C15	1.303 (3)
C21—H21B	0.9900	C15—H15A	0.98 (4)
C22—C23	1.557 (3)	C15—H15B	1.07 (4)
C22—H22A	0.9900	C16—H16A	0.9800
C22—H22B	0.9900	C16—H16B	0.9800
C23—C34	1.537 (3)	C16—H16C	0.9800
C23—C24	1.543 (3)	C17—H17A	0.9800
C24—C31	1.313 (3)	C17—H17B	0.9800
C24—C25	1.499 (3)	C17—H17C	0.9800
C36—O6—H60H	104 (2)	C25—C24—C23	122.36 (17)
O5—C35—C36	120.10 (17)	C26—C25—C30	117.55 (18)
O5—C35—C40	120.79 (18)	C26—C25—C24	123.16 (18)
C36—C35—C40	118.81 (15)	C30—C25—C24	119.29 (19)
O6—C36—C50	105.31 (16)	C25—C26—C27	120.7 (2)
O6—C36—C35	109.31 (15)	C25—C26—H26	119.7
C50—C36—C35	112.69 (16)	C27—C26—H26	119.7
O6—C36—C37	110.27 (16)	C28—C27—C26	120.7 (2)
C50—C36—C37	111.46 (16)	C28—C27—H27	119.6
C35—C36—C37	107.80 (16)	C26—C27—H27	119.6
C38—C37—C36	112.87 (17)	C27—C28—C29	119.2 (2)
C38—C37—H37A	109.0	C27—C28—H28	120.4
C36—C37—H37A	109.0	C29—C28—H28	120.4
C38—C37—H37B	109.0	C30—C29—C28	120.1 (2)

C36—C37—H37B	109.0	C30—C29—H29	120.0
H37A—C37—H37B	107.8	C28—C29—H29	120.0
C37—C38—C39	110.66 (16)	C29—C30—C25	121.8 (2)
C37—C38—H38A	109.5	C29—C30—H30	119.1
C39—C38—H38A	109.5	C25—C30—H30	119.1
C37—C38—H38B	109.5	C32—C31—C24	176.4 (2)
C39—C38—H38B	109.5	C31—C32—H32A	119.5 (19)
H38A—C38—H38B	108.1	C31—C32—H32B	122 (2)
C38—C39—C40	114.29 (16)	H32A—C32—H32B	119 (3)
C38—C39—H39A	108.7	C19—C33—H33A	109.5
C40—C39—H39A	108.7	C19—C33—H33B	109.5
C38—C39—H39B	108.7	H33A—C33—H33B	109.5
C40—C39—H39B	108.7	C19—C33—H33C	109.5
H39A—C39—H39B	107.6	H33A—C33—H33C	109.5
C51—C40—C35	109.71 (15)	H33B—C33—H33C	109.5
C51—C40—C41	108.45 (17)	C23—C34—H34A	109.5
C35—C40—C41	113.97 (15)	C23—C34—H34B	109.5
C51—C40—C39	108.16 (16)	H34A—C34—H34B	109.5
C35—C40—C39	105.01 (16)	C23—C34—H34C	109.5
C41—C40—C39	111.38 (15)	H34A—C34—H34C	109.5
C48—C41—C42	119.13 (18)	H34B—C34—H34C	109.5
C48—C41—C40	119.01 (18)	C2—O2—H20H	104 (2)
C42—C41—C40	121.74 (16)	O1—C1—C2	119.82 (17)
C43—C42—C47	117.22 (19)	O1—C1—C6	121.31 (18)
C43—C42—C41	123.26 (18)	C2—C1—C6	118.76 (17)
C47—C42—C41	119.51 (18)	O2—C2—C16	105.49 (16)
C44—C43—C42	121.07 (19)	O2—C2—C3	109.53 (15)
C44—C43—H43	119.5	C16—C2—C3	112.06 (17)
C42—C43—H43	119.5	O2—C2—C1	109.13 (16)
C45—C44—C43	120.87 (19)	C16—C2—C1	110.79 (15)
C45—C44—H44	119.6	C3—C2—C1	109.72 (16)
C43—C44—H44	119.6	C4—C3—C2	112.59 (16)
C44—C45—C46	118.8 (2)	C4—C3—H3A	109.1
C44—C45—H45	120.6	C2—C3—H3A	109.1
C46—C45—H45	120.6	C4—C3—H3B	109.1
C47—C46—C45	120.5 (2)	C2—C3—H3B	109.1
C47—C46—H46	119.8	H3A—C3—H3B	107.8
C45—C46—H46	119.8	C3—C4—C5	109.89 (17)
C46—C47—C42	121.5 (2)	C3—C4—H4A	109.7

C46—C47—H47	119.2	C5—C4—H4A	109.7
C42—C47—H47	119.2	C3—C4—H4B	109.7
C49—C48—C41	179.1 (2)	C5—C4—H4B	109.7
C48—C49—H49A	122.7 (18)	H4A—C4—H4B	108.2
C48—C49—H49B	119.4 (17)	C4—C5—C6	113.89 (17)
H49A—C49—H49B	118 (2)	C4—C5—H5A	108.8
C36—C50—H50A	109.5	C6—C5—H5A	108.8
C36—C50—H50B	109.5	C4—C5—H5B	108.8
H50A—C50—H50B	109.5	C6—C5—H5B	108.8
C36—C50—H50C	109.5	H5A—C5—H5B	107.7
H50A—C50—H50C	109.5	C17—C6—C7	108.87 (16)
H50B—C50—H50C	109.5	C17—C6—C5	107.57 (16)
C40—C51—H51A	109.5	C7—C6—C5	111.91 (16)
C40—C51—H51B	109.5	C17—C6—C1	109.87 (17)
H51A—C51—H51B	109.5	C7—C6—C1	112.19 (15)
C40—C51—H51C	109.5	C5—C6—C1	106.31 (15)
H51A—C51—H51C	109.5	C14—C7—C8	118.93 (18)
H51B—C51—H51C	109.5	C14—C7—C6	118.77 (18)
C19—O4—H40H	104 (2)	C8—C7—C6	122.11 (17)
O3—C18—C23	121.11 (17)	C13—C8—C9	117.42 (19)
O3—C18—C19	120.07 (19)	C13—C8—C7	123.20 (18)
C23—C18—C19	118.43 (16)	C9—C8—C7	119.37 (19)
O4—C19—C33	105.42 (15)	C10—C9—C8	121.5 (2)
O4—C19—C20	109.65 (15)	C10—C9—H9	119.2
C33—C19—C20	112.26 (16)	C8—C9—H9	119.2
O4—C19—C18	109.62 (15)	C9—C10—C11	120.6 (2)
C33—C19—C18	112.35 (15)	C9—C10—H10	119.7
C20—C19—C18	107.52 (16)	C11—C10—H10	119.7
C21—C20—C19	112.43 (16)	C12—C11—C10	118.6 (2)
C21—C20—H20A	109.1	C12—C11—H11	120.7
C19—C20—H20A	109.1	C10—C11—H11	120.7
C21—C20—H20B	109.1	C11—C12—C13	121.2 (2)
C19—C20—H20B	109.1	C11—C12—H12	119.4
H20A—C20—H20B	107.8	C13—C12—H12	119.4
C20—C21—C22	110.45 (17)	C12—C13—C8	120.66 (19)
C20—C21—H21A	109.6	C12—C13—H13	119.7
C22—C21—H21A	109.6	C8—C13—H13	119.7
C20—C21—H21B	109.6	C15—C14—C7	178.5 (3)
C22—C21—H21B	109.6	C14—C15—H15A	120 (2)

H21A—C21—H21B	108.1	C14—C15—H15B	117.4 (19)
C21—C22—C23	114.44 (16)	H15A—C15—H15B	122 (3)
C21—C22—H22A	108.6	C2—C16—H16A	109.5
C23—C22—H22A	108.6	C2—C16—H16B	109.5
C21—C22—H22B	108.6	H16A—C16—H16B	109.5
C23—C22—H22B	108.6	C2—C16—H16C	109.5
H22A—C22—H22B	107.6	H16A—C16—H16C	109.5
C34—C23—C18	110.47 (16)	H16B—C16—H16C	109.5
C34—C23—C24	108.02 (15)	C6—C17—H17A	109.5
C18—C23—C24	114.19 (16)	C6—C17—H17B	109.5
C34—C23—C22	108.08 (16)	H17A—C17—H17B	109.5
C18—C23—C22	104.69 (15)	C6—C17—H17C	109.5
C24—C23—C22	111.23 (16)	H17A—C17—H17C	109.5
C31—C24—C25	117.78 (18)	H17B—C17—H17C	109.5
C31—C24—C23	119.64 (17)		
O5—C35—C36—O6	0.2 (2)	C21—C22—C23—C24	70.1 (2)
C40—C35—C36—O6	-173.56 (16)	C34—C23—C24—C31	-104.0 (2)
O5—C35—C36—C50	-116.5 (2)	C18—C23—C24—C31	132.72 (19)
C40—C35—C36—C50	69.7 (2)	C22—C23—C24—C31	14.5 (3)
O5—C35—C36—C37	120.09 (19)	C34—C23—C24—C25	70.7 (2)
C40—C35—C36—C37	-53.7 (2)	C18—C23—C24—C25	-52.6 (2)
O6—C36—C37—C38	171.26 (17)	C22—C23—C24—C25	-170.87 (17)
C50—C36—C37—C38	-72.2 (2)	C31—C24—C25—C26	177.7 (2)
C35—C36—C37—C38	52.0 (2)	C23—C24—C25—C26	3.0 (3)
C36—C37—C38—C39	-56.3 (2)	C31—C24—C25—C30	-1.6 (3)
C37—C38—C39—C40	58.3 (2)	C23—C24—C25—C30	-176.34 (17)
O5—C35—C40—C51	-4.4 (3)	C30—C25—C26—C27	0.0 (3)
C36—C35—C40—	169.39 (17)	C24—C25—C26—	-179.35 (19)

C51		C27	
O5—C35—C40—C41	117.5 (2)	C25—C26—C27—C28	0.7 (3)
C36—C35—C40—C41	-68.8 (2)	C26—C27—C28—C29	-0.7 (3)
O5—C35—C40—C39	-120.38 (19)	C27—C28—C29—C30	0.1 (3)
C36—C35—C40—C39	53.4 (2)	C28—C29—C30—C25	0.7 (3)
C38—C39—C40—C51	-170.74 (17)	C26—C25—C30—C29	-0.7 (3)
C38—C39—C40—C35	-53.6 (2)	C24—C25—C30—C29	178.69 (19)
C38—C39—C40—C41	70.2 (2)	O1—C1—C2—O2	7.3 (2)
C51—C40—C41—C48	-102.8 (2)	C6—C1—C2—O2	-168.86 (15)
C35—C40—C41—C48	134.7 (2)	O1—C1—C2—C16	-108.5 (2)
C39—C40—C41—C48	16.1 (3)	C6—C1—C2—C16	75.4 (2)
C51—C40—C41—C42	73.2 (2)	O1—C1—C2—C3	127.28 (19)
C35—C40—C41—C42	-49.3 (2)	C6—C1—C2—C3	-48.8 (2)
C39—C40—C41—C42	-167.91 (17)	O2—C2—C3—C4	170.40 (16)
C48—C41—C42—C43	178.1 (2)	C16—C2—C3—C4	-72.9 (2)
C40—C41—C42—C43	2.1 (3)	C1—C2—C3—C4	50.6 (2)
C48—C41—C42—C47	-3.1 (3)	C2—C3—C4—C5	-57.5 (2)
C40—C41—C42—C47	-179.08 (19)	C3—C4—C5—C6	60.5 (2)
C47—C42—C43—C44	-1.9 (3)	C4—C5—C6—C17	-171.41 (17)
C41—C42—C43—C44	176.9 (2)	C4—C5—C6—C7	69.1 (2)
C42—C43—C44—C45	1.3 (3)	C4—C5—C6—C1	-53.8 (2)
C43—C44—C45—	0.3 (3)	O1—C1—C6—C17	-10.8 (2)

C46			
C44—C45—C46—C47	-1.3 (3)	C2—C1—C6—C17	165.31 (16)
C45—C46—C47—C42	0.6 (4)	O1—C1—C6—C7	110.5 (2)
C43—C42—C47—C46	1.0 (3)	C2—C1—C6—C7	-73.4 (2)
C41—C42—C47—C46	-177.9 (2)	O1—C1—C6—C5	-126.88 (19)
O3—C18—C19—O4	-2.0 (2)	C2—C1—C6—C5	49.2 (2)
C23—C18—C19—O4	-174.92 (15)	C17—C6—C7—C14	-98.7 (2)
O3—C18—C19—C33	-118.8 (2)	C5—C6—C7—C14	20.1 (3)
C23—C18—C19—C33	68.2 (2)	C1—C6—C7—C14	139.5 (2)
O3—C18—C19—C20	117.15 (19)	C17—C6—C7—C8	76.4 (2)
C23—C18—C19—C20	-55.8 (2)	C5—C6—C7—C8	-164.83 (17)
O4—C19—C20—C21	172.51 (16)	C1—C6—C7—C8	-45.4 (2)
C33—C19—C20—C21	-70.7 (2)	C14—C7—C8—C13	161.8 (2)
C18—C19—C20—C21	53.4 (2)	C6—C7—C8—C13	-13.3 (3)
C19—C20—C21—C22	-56.4 (2)	C14—C7—C8—C9	-17.7 (3)
C20—C21—C22—C23	57.8 (2)	C6—C7—C8—C9	167.28 (19)
O3—C18—C23—C34	-2.2 (2)	C13—C8—C9—C10	-0.8 (3)
C19—C18—C23—C34	170.68 (16)	C7—C8—C9—C10	178.6 (2)
O3—C18—C23—C24	119.82 (19)	C8—C9—C10—C11	0.7 (4)
C19—C18—C23—C24	-67.3 (2)	C9—C10—C11—C12	0.2 (4)
O3—C18—C23—C22	-118.29 (19)	C10—C11—C12—C13	-1.0 (3)
C19—C18—C23—C22	54.6 (2)	C11—C12—C13—C8	0.9 (3)

C21—C22—C23—C34	-171.43 (18)	C9—C8—C13—C12	0.1 (3)
C21—C22—C23—C18	-53.7 (2)	C7—C8—C13—C12	-179.41 (19)

Hydrogen-bond geometry (Å, º) for (3027P)

D—H···*A*	*D*—H	H···*A*	*D*···*A*	*D*—H···*A*
O6—H60*H*···O4	0.85 (3)	1.98 (4)	2.814 (2)	168 (3)
C37—H37*B*···O3	0.99	2.60	3.574 (3)	169
O4—H40*H*···O2	0.84 (3)	2.03 (4)	2.857 (2)	166 (3)
C33—H33*B*···O1^{i}	0.98	2.44	3.363 (3)	157
O2—H20*H*···O6^{ii}	0.88 (4)	2.02 (4)	2.886 (2)	168 (3)

Symmetry codes: (i) *x*, *y*+1, *z*; (ii) *x*, *y*-1, *z*.

Compound (+)-6l

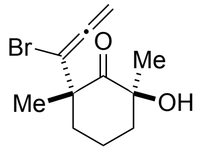

(+)-6l

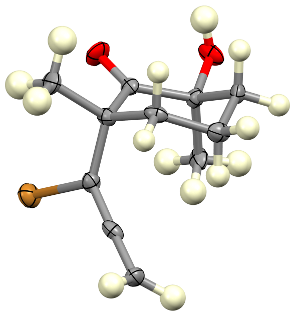

Sample Name: CCDC 2426291 / Armend13A (EAG3124P)
Crystal Growth: Slow evaporation using a mixture of hexanes and dichloromethane
Ellipsoid Contour: Set at a 50% probability level

Crystal data

$C_{11}H_{15}BrO_2$	$F(000)$ = 1584
M_r = 259.14	D_x = 1.504 Mg m^{-3}
Monoclinic, $P2_1$	Cu *K*a radiation, l = 1.54184 Å
a = 8.4246 (3) Å	Cell parameters from 9662 reflections
b = 37.9595 (14) Å	q = 4.7–79.0°
c = 10.7376 (4) Å	m = 4.68 mm^{-1}
b = 90.643 (2)°	*T* = 100 K
V = 3433.6 (2) Å^3	Plate fragment, colourless
Z = 12	0.17 × 0.07 × 0.06 mm

Data collection

Bruker D8 Venture DUO with Photon III C14 diffractometer	14194 reflections with *I* > 2s(*I*)
Radiation source: ImS 3.0 microfocus	R_{int} = 0.075
f and w scans	q_{max} = 80.1°, q_{min} = 4.1°
Absorption correction: multi-scan *SADABS* (Krause *et al.*, 2015)	*h* = -10 - 10
T_{min} = 0.569, T_{max} = 0.766	*k* = -48 - 47
81971 measured reflections	*l* = -13 - 11
14580 independent reflections	

Refinement

Refinement on F^2	Hydrogen site location: mixed
Least-squares matrix: full	H atoms treated by a mixture of independent and constrained refinement

$R[F^2 > 2s(F^2)] = 0.045$	$w = 1/[s^2(F_o^2) + (0.0344P)^2 + 10.9871P]$<br>where $P = (F_o^2 + 2F_c^2)/3$
$wR(F^2) = 0.109$	$(D/s)_{max} = 0.001$
$S = 1.09$	$Dr_{max} = 0.78$ e Å^{-3}
14580 reflections	$Dr_{min} = -1.39$ e Å^{-3}
811 parameters	Absolute structure: Flack x determined using 6319 quotients [(I+)-(I-)]/[(I+)+(I-)] (Parsons, Flack and Wagner, Acta Cryst. B69 (2013) 249-259).
67 restraints	Absolute structure parameter: 0.096 (6)

Fractional atomic coordinates and isotropic or equivalent isotropic displacement parameters (Å^2) for (EAG3124P)

	x	*y*	*z*	U_{iso}*/U_{eq}
Br3	-0.20413 (10)	0.33172 (2)	0.46198 (8)	0.0332 (2)
O5	-0.0536 (6)	0.38179 (14)	0.2225 (5)	0.0250 (11)
O6	0.2031 (6)	0.35404 (13)	0.1162 (4)	0.0195 (9)
H6O	0.133732	0.341410	0.081483	0.029*
C23	0.0538 (8)	0.37292 (17)	0.2936 (6)	0.0172 (13)
C24	0.1918 (8)	0.35044 (17)	0.2490 (6)	0.0190 (13)
C25	0.3496 (8)	0.36364 (19)	0.3033 (6)	0.0206 (13)
H25A	0.432986	0.345953	0.286470	0.025*
H25B	0.379392	0.385707	0.260394	0.025*
C26	0.3447 (8)	0.3705 (2)	0.4428 (7)	0.0226 (14)
H26A	0.448445	0.379978	0.471212	0.027*
H26B	0.325914	0.348057	0.487153	0.027*
C27	0.2138 (8)	0.39665 (19)	0.4744 (7)	0.0207 (14)
H27A	0.238423	0.419579	0.435142	0.025*
H27B	0.213284	0.400254	0.565707	0.025*
C28	0.0459 (8)	0.38481 (18)	0.4312 (6)	0.0185 (13)
C29	0.1592 (9)	0.31187 (17)	0.2831 (7)	0.0202 (13)
H29A	0.160874	0.309243	0.373855	0.030*
H29B	0.241119	0.296803	0.246829	0.030*
H29C	0.054814	0.304922	0.250218	0.030*
C30	-0.0093 (9)	0.3545 (2)	0.5137 (7)	0.0232 (14)
C31	0.0543 (9)	0.34430 (19)	0.6183 (7)	0.0223 (14)
C32	0.1172 (9)	0.3342 (2)	0.7247 (7)	0.0273 (15)
H32A	0.203 (9)	0.317 (2)	0.720 (10)	0.041*
H32B	0.088 (13)	0.350 (2)	0.793 (7)	0.041*

C33	-0.0709 (10)	0.4156 (2)	0.4430 (8)	0.0298 (17)
H33A	-0.088016	0.420765	0.531224	0.045*
H33B	-0.172202	0.409283	0.403192	0.045*
H33C	-0.027030	0.436467	0.401982	0.045*
Br5	0.97463 (9)	0.71983 (2)	0.72971 (7)	0.02320 (15)
O9	0.6825 (6)	0.68170 (13)	0.5460 (5)	0.0204 (10)
O10	0.7550 (6)	0.61432 (12)	0.5143 (5)	0.0207 (10)
H10O	0.713658	0.617129	0.584469	0.031*
C56	0.8162 (8)	0.67644 (17)	0.5091 (6)	0.0164 (13)
C57	0.8834 (8)	0.63859 (17)	0.5004 (6)	0.0156 (12)
C58	0.9518 (9)	0.63272 (19)	0.3720 (6)	0.0206 (14)
H58A	0.864279	0.632454	0.309697	0.025*
H58B	1.004590	0.609426	0.369843	0.025*
C59	1.0717 (9)	0.6612 (2)	0.3366 (6)	0.0244 (15)
H59A	1.112888	0.656386	0.252277	0.029*
H59B	1.162371	0.660808	0.396005	0.029*
C60	0.9931 (9)	0.6979 (2)	0.3384 (6)	0.0239 (15)
H60A	1.074198	0.715816	0.318379	0.029*
H60B	0.910383	0.698761	0.272296	0.029*
C61	0.9171 (9)	0.70758 (18)	0.4631 (7)	0.0219 (14)
C62	1.0057 (8)	0.63260 (18)	0.6044 (6)	0.0186 (13)
H62A	1.094432	0.649015	0.594323	0.028*
H62B	1.045121	0.608342	0.600389	0.028*
H62C	0.956056	0.636607	0.685317	0.028*
C63	1.0465 (9)	0.71630 (18)	0.5599 (6)	0.0196 (13)
C64	1.1939 (9)	0.72425 (19)	0.5371 (7)	0.0255 (15)
C65	1.3394 (10)	0.7320 (2)	0.5154 (8)	0.0280 (16)
H65A	1.425 (9)	0.714 (2)	0.531 (10)	0.042*
H65B	1.375 (12)	0.7548 (16)	0.482 (9)	0.042*
C66	0.8127 (10)	0.7404 (2)	0.4466 (9)	0.0319 (18)
H66A	0.876865	0.759788	0.414204	0.048*
H66B	0.768475	0.747124	0.527169	0.048*
H66C	0.725979	0.735173	0.387810	0.048*
Br6	0.85229 (9)	0.66402 (3)	1.01212 (7)	0.03187 (19)
O11	0.6904 (6)	0.61589 (13)	0.7771 (5)	0.0228 (10)
O12	0.4357 (6)	0.64329 (13)	0.6623 (4)	0.0191 (9)
H12O	0.522121	0.648784	0.629207	0.029*
C45	0.5849 (8)	0.62516 (17)	0.8449 (6)	0.0169 (12)
C46	0.4486 (8)	0.64784 (18)	0.7946 (6)	0.0177 (13)

C47	0.2889 (8)	0.63667 (19)	0.8454 (6)	0.0201 (13)
H47A	0.256668	0.614243	0.805402	0.024*
H47B	0.208688	0.654693	0.822811	0.024*
C48	0.2901 (8)	0.6317 (2)	0.9875 (7)	0.0228 (14)
H48A	0.312420	0.654521	1.028945	0.027*
H48B	0.184840	0.623357	1.014947	0.027*
C49	0.4178 (9)	0.6048 (2)	1.0242 (7)	0.0277 (16)
H49A	0.418152	0.602105	1.115914	0.033*
H49B	0.388789	0.581714	0.987589	0.033*
C50	0.5858 (9)	0.6142 (2)	0.9832 (7)	0.0251 (15)
C51	0.4855 (10)	0.68650 (18)	0.8242 (7)	0.0245 (15)
H51A	0.488356	0.689878	0.914668	0.037*
H51B	0.403035	0.701552	0.787311	0.037*
H51C	0.588846	0.692778	0.789487	0.037*
C52	0.6504 (9)	0.6450 (2)	1.0601 (7)	0.0269 (16)
C53	0.5853 (9)	0.6581 (3)	1.1620 (7)	0.0319 (18)
C54	0.5186 (11)	0.6697 (4)	1.2621 (8)	0.049 (3)
H54B	0.441 (13)	0.689 (3)	1.253 (13)	0.074*
H54A	0.534 (17)	0.657 (3)	1.341 (8)	0.074*
C55	0.6972 (11)	0.5826 (2)	1.0022 (9)	0.038 (2)
H55A	0.697711	0.575707	1.090171	0.057*
H55B	0.805000	0.589150	0.977673	0.057*
H55C	0.660138	0.562839	0.951006	0.057*
Br1	-0.35312 (9)	0.27869 (2)	0.19343 (7)	0.02356 (15)
O1	-0.0471 (6)	0.31178 (13)	0.0074 (5)	0.0221 (10)
O2	-0.1134 (6)	0.37842 (13)	-0.0371 (5)	0.0240 (10)
H2O	-0.075616	0.381832	-0.108166	0.036*
C1	-0.1821 (8)	0.31701 (19)	-0.0304 (6)	0.0183 (13)
C2	-0.2447 (8)	0.35468 (18)	-0.0466 (6)	0.0177 (13)
C3	-0.3155 (9)	0.3590 (2)	-0.1785 (7)	0.0295 (17)
H3A	-0.370542	0.381975	-0.184472	0.035*
H3B	-0.228484	0.358970	-0.239735	0.035*
C4	-0.4334 (9)	0.3293 (2)	-0.2112 (7)	0.0309 (17)
H4A	-0.524132	0.330264	-0.153451	0.037*
H4B	-0.474984	0.332907	-0.296845	0.037*
C5	-0.3550 (10)	0.2937 (2)	-0.2022 (7)	0.0304 (17)
H5A	-0.268719	0.292498	-0.263892	0.036*
H5B	-0.434031	0.275361	-0.224564	0.036*
C6	-0.2847 (8)	0.28509 (18)	-0.0712 (6)	0.0205 (14)

C7	-0.3656 (8)	0.36284 (18)	0.0544 (7)	0.0203 (13)
H7A	-0.459406	0.347886	0.042594	0.030*
H7B	-0.396894	0.387663	0.048991	0.030*
H7C	-0.318104	0.358201	0.136542	0.030*
C8	-0.4173 (8)	0.27923 (19)	0.0216 (6)	0.0198 (13)
C9	-0.5650 (9)	0.27163 (18)	-0.0038 (7)	0.0242 (15)
C10	-0.7113 (10)	0.2636 (2)	-0.0294 (8)	0.0317 (17)
H10A	-0.748 (13)	0.2399 (15)	-0.054 (10)	0.048*
H10B	-0.795 (10)	0.281 (2)	-0.043 (10)	0.048*
C11	-0.1824 (10)	0.2516 (2)	-0.0750 (9)	0.0336 (19)
H11A	-0.247675	0.231671	-0.103211	0.050*
H11B	-0.139970	0.246608	0.008573	0.050*
H11C	-0.094460	0.255027	-0.132632	0.050*
Br2	0.10654 (10)	0.53919 (2)	0.16495 (7)	0.02929 (18)
O3	0.1377 (7)	0.44726 (16)	0.1739 (5)	0.0305 (12)
O4	0.3555 (6)	0.41948 (14)	0.0298 (6)	0.0273 (11)
H4O	0.296207	0.402825	0.051344	0.041*
C12	0.1297 (8)	0.45696 (17)	0.0672 (6)	0.0167 (12)
C13	0.2618 (8)	0.44698 (18)	-0.0239 (6)	0.0181 (13)
C14	0.1894 (9)	0.43432 (19)	-0.1464 (6)	0.0214 (14)
H14A	0.136277	0.411399	-0.132731	0.026*
H14B	0.275252	0.430562	-0.207189	0.026*
C15	0.0690 (10)	0.46038 (19)	-0.2011 (7)	0.0248 (15)
H15A	0.122012	0.483129	-0.218022	0.030*
H15B	0.026104	0.451122	-0.280797	0.030*
C16	-0.0653 (9)	0.46603 (18)	-0.1107 (7)	0.0217 (14)
H16A	-0.122965	0.443511	-0.100468	0.026*
H16B	-0.140594	0.483295	-0.147231	0.026*
C17	-0.0119 (8)	0.47927 (17)	0.0186 (6)	0.0179 (13)
C18	0.3746 (9)	0.4781 (2)	-0.0397 (7)	0.0249 (15)
H18A	0.320405	0.496939	-0.086148	0.037*
H18B	0.468445	0.470519	-0.085603	0.037*
H18C	0.407263	0.486977	0.042403	0.037*
C19	0.0363 (9)	0.51751 (18)	0.0104 (6)	0.0197 (13)
C20	0.0318 (8)	0.53810 (18)	-0.0877 (7)	0.0203 (13)
C21	0.0236 (10)	0.55748 (19)	-0.1860 (7)	0.0236 (15)
H21B	0.105 (9)	0.559 (3)	-0.251 (7)	0.035*
H21A	-0.083 (6)	0.568 (2)	-0.194 (9)	0.035*
C22	-0.1501 (10)	0.4749 (2)	0.1082 (8)	0.0296 (17)

H22A	-0.241644	0.488357	0.077462	0.044*
H22B	-0.118601	0.483541	0.190816	0.044*
H22C	-0.178572	0.449898	0.113781	0.044*
Br4	0.57275 (13)	0.45328 (2)	0.64280 (7)	0.0371 (2)
O7	0.5895 (10)	0.54591 (17)	0.6902 (6)	0.0453 (17)
O8	0.3165 (8)	0.57430 (16)	0.6160 (7)	0.0449 (18)
H8O	0.378538	0.591354	0.627356	0.067*
C34	0.5471 (9)	0.53805 (19)	0.5867 (6)	0.0236 (14)
C35	0.3860 (9)	0.5500 (2)	0.5329 (8)	0.0286 (17)
C36	0.4108 (10)	0.5668 (2)	0.4043 (8)	0.0293 (17)
H36A	0.306183	0.572240	0.366041	0.035*
H36B	0.469320	0.589269	0.414687	0.035*
C37	0.5025 (10)	0.54280 (19)	0.3183 (7)	0.0269 (16)
H37A	0.442129	0.520735	0.304856	0.032*
H37B	0.515369	0.554457	0.236546	0.032*
C38	0.6643 (9)	0.53420 (18)	0.3729 (7)	0.0221 (14)
H38A	0.727058	0.556197	0.378757	0.026*
H38B	0.719867	0.518159	0.315182	0.026*
C39	0.6597 (8)	0.51687 (18)	0.5029 (7)	0.0195 (13)
C40	0.2697 (10)	0.5189 (2)	0.5273 (11)	0.043 (2)
H40A	0.312357	0.500472	0.473033	0.065*
H40B	0.167189	0.526930	0.493963	0.065*
H40C	0.255323	0.509335	0.611199	0.065*
C41	0.6007 (8)	0.47890 (17)	0.4893 (6)	0.0183 (13)
C42	0.5730 (8)	0.46138 (17)	0.3872 (7)	0.0198 (13)
C43	0.5420 (10)	0.4447 (2)	0.2855 (7)	0.0282 (16)
H43B	0.621 (10)	0.429 (2)	0.246 (9)	0.042*
H43A	0.443 (8)	0.450 (3)	0.240 (9)	0.042*
C44	0.8273 (10)	0.5162 (2)	0.5571 (10)	0.039 (2)
H44A	0.896415	0.502660	0.501871	0.059*
H44B	0.826041	0.505047	0.639430	0.059*
H44C	0.867402	0.540300	0.564772	0.059*

Atomic displacement parameters (Å^2) for (EAG3124P)

	U^{11}	U^{22}	U^{33}	U^{12}	U^{13}	U^{23}
Br3	0.0253 (4)	0.0494 (5)	0.0249 (4)	-0.0163 (4)	0.0000 (3)	-0.0012 (4)
O5	0.022 (3)	0.029 (3)	0.024 (3)	0.003 (2)	-0.003 (2)	-0.002 (2)
O6	0.022 (2)	0.019 (2)	0.017 (2)	-0.0047 (19)	0.0036 (18)	-0.0020 (18)

C23	0.016 (3)	0.015 (3)	0.021 (3)	-0.001 (2)	-0.002 (2)	0.000 (2)
C24	0.020 (3)	0.016 (3)	0.021 (3)	-0.001 (3)	-0.005 (3)	-0.002 (2)
C25	0.018 (3)	0.024 (3)	0.020 (3)	-0.004 (3)	0.002 (3)	-0.002 (3)
C26	0.018 (3)	0.029 (4)	0.021 (3)	-0.001 (3)	-0.008 (3)	-0.003 (3)
C27	0.020 (3)	0.023 (3)	0.019 (3)	-0.003 (3)	-0.004 (3)	-0.007 (3)
C28	0.018 (3)	0.022 (3)	0.016 (3)	0.002 (3)	-0.001 (2)	-0.004 (2)
C29	0.025 (4)	0.015 (3)	0.021 (3)	-0.003 (3)	0.002 (3)	-0.002 (3)
C30	0.018 (3)	0.025 (3)	0.026 (4)	-0.003 (3)	-0.002 (3)	-0.003 (3)
C31	0.021 (3)	0.028 (4)	0.018 (3)	-0.003 (3)	0.004 (3)	-0.006 (3)
C32	0.025 (4)	0.029 (4)	0.028 (4)	-0.003 (3)	0.004 (3)	0.004 (3)
C33	0.027 (4)	0.029 (4)	0.033 (4)	0.012 (3)	0.002 (3)	-0.004 (3)
Br5	0.0256 (4)	0.0219 (3)	0.0220 (3)	-0.0030 (3)	-0.0008 (3)	-0.0039 (3)
O9	0.019 (2)	0.021 (2)	0.021 (2)	0.0028 (19)	0.0021 (19)	-0.0011 (19)
O10	0.020 (2)	0.018 (2)	0.024 (3)	-0.0052 (18)	0.0009 (19)	-0.0049 (19)
C56	0.017 (3)	0.022 (3)	0.011 (3)	-0.001 (2)	-0.004 (2)	-0.001 (2)
C57	0.018 (3)	0.015 (3)	0.013 (3)	0.001 (2)	0.000 (2)	-0.002 (2)
C58	0.025 (4)	0.027 (3)	0.010 (3)	-0.002 (3)	0.004 (2)	-0.005 (2)
C59	0.022 (3)	0.039 (4)	0.011 (3)	-0.006 (3)	0.000 (2)	0.000 (3)
C60	0.026 (4)	0.037 (4)	0.009 (3)	-0.008 (3)	0.002 (3)	0.010 (3)
C61	0.025 (4)	0.017 (3)	0.024 (3)	-0.003 (3)	-0.007 (3)	0.009 (3)
C62	0.021 (3)	0.019 (3)	0.016 (3)	0.001 (3)	-0.003 (2)	-0.001 (2)
C63	0.026 (3)	0.018 (3)	0.015 (3)	-0.002 (3)	-0.002 (2)	-0.004 (2)
C64	0.027 (4)	0.021 (3)	0.028 (4)	-0.004 (3)	-0.005 (3)	0.002 (3)
C65	0.028 (4)	0.029 (4)	0.027 (4)	0.000 (3)	-0.005 (3)	0.001 (3)
C66	0.034 (4)	0.018 (4)	0.043 (5)	0.000 (3)	-0.009 (4)	0.008 (3)
Br6	0.0214 (4)	0.0512 (5)	0.0230 (4)	0.0007 (4)	-0.0014 (3)	-0.0032 (3)
O11	0.018 (2)	0.027 (3)	0.024 (3)	0.004 (2)	0.004 (2)	0.001 (2)
O12	0.020 (2)	0.026 (2)	0.011 (2)	-0.0021 (19)	0.0017 (17)	-0.0013 (18)
C45	0.018 (3)	0.017 (3)	0.016 (3)	0.001 (2)	0.001 (2)	0.002 (2)
C46	0.020 (3)	0.021 (3)	0.011 (3)	0.004 (3)	0.000 (2)	0.002 (2)
C47	0.016 (3)	0.026 (3)	0.018 (3)	0.000 (3)	0.004 (2)	-0.002 (3)
C48	0.012 (3)	0.035 (4)	0.022 (3)	0.000 (3)	0.002 (3)	0.007 (3)
C49	0.025 (4)	0.031 (4)	0.027 (4)	0.003 (3)	0.007 (3)	0.012 (3)
C50	0.023 (4)	0.029 (4)	0.023 (4)	0.009 (3)	0.001 (3)	0.004 (3)
C51	0.030 (4)	0.017 (3)	0.026 (4)	0.006 (3)	-0.006 (3)	-0.003 (3)
C52	0.018 (3)	0.046 (5)	0.017 (3)	0.009 (3)	-0.003 (3)	0.008 (3)

C53	0.015 (3)	0.059 (5)	0.021 (4)	0.004 (3)	-0.001 (3)	-0.003 (3)
C54	0.024 (4)	0.102 (9)	0.022 (4)	0.002 (5)	0.002 (3)	-0.013 (5)
C55	0.039 (5)	0.039 (5)	0.037 (5)	0.018 (4)	0.007 (4)	0.019 (4)
Br1	0.0279 (4)	0.0205 (3)	0.0223 (3)	0.0001 (3)	-0.0025 (3)	0.0023 (3)
O1	0.019 (2)	0.022 (2)	0.026 (3)	0.0033 (19)	-0.0017 (19)	-0.005 (2)
O2	0.021 (3)	0.024 (2)	0.027 (3)	-0.004 (2)	0.002 (2)	0.002 (2)
C1	0.016 (3)	0.028 (4)	0.010 (3)	-0.001 (3)	-0.001 (2)	-0.002 (2)
C2	0.010 (3)	0.020 (3)	0.023 (3)	-0.003 (2)	0.001 (2)	0.006 (3)
C3	0.023 (4)	0.044 (5)	0.021 (4)	-0.003 (3)	-0.008 (3)	0.016 (3)
C4	0.019 (4)	0.050 (5)	0.023 (4)	-0.007 (3)	-0.007 (3)	0.012 (3)
C5	0.024 (4)	0.041 (4)	0.026 (4)	-0.006 (3)	0.001 (3)	-0.010 (3)
C6	0.019 (3)	0.025 (4)	0.017 (3)	-0.002 (3)	0.002 (2)	-0.007 (3)
C7	0.021 (3)	0.017 (3)	0.024 (3)	0.005 (3)	0.001 (3)	0.002 (3)
C8	0.022 (3)	0.018 (3)	0.020 (3)	-0.001 (3)	-0.002 (2)	-0.003 (3)
C9	0.025 (4)	0.017 (3)	0.031 (4)	0.003 (3)	0.003 (3)	-0.003 (3)
C10	0.020 (4)	0.037 (4)	0.037 (5)	-0.004 (3)	-0.003 (3)	0.001 (4)
C11	0.025 (4)	0.025 (4)	0.051 (5)	0.003 (3)	0.005 (4)	-0.016 (4)
Br2	0.0384 (4)	0.0270 (4)	0.0223 (4)	-0.0018 (3)	-0.0049 (3)	-0.0055 (3)
O3	0.036 (3)	0.037 (3)	0.018 (2)	0.007 (2)	0.002 (2)	0.012 (2)
O4	0.023 (3)	0.020 (2)	0.039 (3)	0.007 (2)	-0.005 (2)	0.000 (2)
C12	0.013 (3)	0.017 (3)	0.021 (3)	-0.002 (2)	0.002 (2)	-0.001 (2)
C13	0.015 (3)	0.021 (3)	0.018 (3)	0.003 (3)	0.003 (2)	0.001 (2)
C14	0.025 (4)	0.023 (3)	0.016 (3)	0.000 (3)	0.007 (3)	-0.004 (3)
C15	0.033 (4)	0.021 (3)	0.020 (3)	-0.002 (3)	-0.006 (3)	0.001 (3)
C16	0.021 (3)	0.017 (3)	0.026 (4)	-0.001 (3)	-0.006 (3)	0.002 (3)
C17	0.015 (3)	0.016 (3)	0.023 (3)	0.002 (2)	0.001 (2)	0.001 (2)
C18	0.016 (3)	0.027 (4)	0.032 (4)	-0.004 (3)	0.009 (3)	-0.007 (3)
C19	0.022 (3)	0.020 (3)	0.017 (3)	0.003 (3)	-0.002 (3)	-0.001 (2)
C20	0.019 (3)	0.014 (3)	0.028 (3)	0.001 (3)	-0.005 (3)	-0.005 (3)
C21	0.033 (4)	0.020 (3)	0.018 (3)	0.001 (3)	0.003 (3)	0.002 (3)
C22	0.024 (4)	0.031 (4)	0.034 (4)	0.002 (3)	0.014 (3)	0.000 (3)
Br4	0.0661 (6)	0.0270 (4)	0.0182 (4)	-0.0081 (4)	-0.0029 (4)	0.0052 (3)
O7	0.074 (5)	0.038 (3)	0.024 (3)	-0.001 (3)	-0.002 (3)	-0.014 (3)
O8	0.035 (3)	0.028 (3)	0.073 (5)	-0.003 (3)	0.033 (3)	-0.022 (3)
C34	0.034 (4)	0.017 (3)	0.020 (3)	-0.005 (3)	0.003 (3)	-0.007 (3)
C35	0.021 (4)	0.020 (3)	0.044 (5)	-0.002 (3)	0.010 (3)	-0.014 (3)
C36	0.026 (4)	0.021 (3)	0.040 (4)	0.005 (3)	-0.010 (3)	-0.004 (3)
C37	0.042 (4)	0.018 (3)	0.021 (3)	0.002 (3)	-0.003 (3)	0.003 (3)

C38	0.024 (3)	0.018 (3)	0.025 (3)	-0.004 (3)	0.009 (3)	-0.008 (3)
C39	0.018 (3)	0.017 (3)	0.024 (3)	-0.002 (3)	-0.001 (3)	-0.004 (3)
C40	0.020 (4)	0.027 (4)	0.084 (8)	-0.007 (3)	0.020 (4)	-0.014 (4)
C41	0.018 (3)	0.017 (3)	0.020 (3)	0.001 (2)	-0.003 (3)	-0.003 (2)
C42	0.018 (3)	0.012 (3)	0.029 (4)	-0.003 (2)	0.003 (3)	0.003 (2)
C43	0.036 (4)	0.023 (4)	0.026 (4)	-0.011 (3)	0.005 (3)	-0.004 (3)
C44	0.029 (4)	0.021 (4)	0.066 (6)	0.000 (3)	-0.019 (4)	-0.014 (4)

Geometric parameters (Å, º) for (EAG3124P)

Br3—C30	1.932 (7)	Br1—C8	1.917 (7)
O5—C23	1.224 (8)	O1—C1	1.220 (8)
O6—C24	1.436 (8)	O2—C2	1.430 (8)
O6—H6O	0.8400	O2—H2O	0.8400
C23—C24	1.524 (10)	C1—C2	1.533 (9)
C23—C28	1.547 (9)	C1—C6	1.548 (9)
C24—C25	1.530 (9)	C2—C7	1.528 (9)
C24—C29	1.535 (9)	C2—C3	1.540 (9)
C25—C26	1.520 (9)	C3—C4	1.539 (11)
C25—H25A	0.9900	C3—H3A	0.9900
C25—H25B	0.9900	C3—H3B	0.9900
C26—C27	1.525 (10)	C4—C5	1.509 (13)
C26—H26A	0.9900	C4—H4A	0.9900
C26—H26B	0.9900	C4—H4B	0.9900
C27—C28	1.551 (9)	C5—C6	1.555 (11)
C27—H27A	0.9900	C5—H5A	0.9900
C27—H27B	0.9900	C5—H5B	0.9900
C28—C30	1.527 (10)	C6—C8	1.521 (9)
C28—C33	1.534 (10)	C6—C11	1.538 (10)
C29—H29A	0.9800	C7—H7A	0.9800
C29—H29B	0.9800	C7—H7B	0.9800
C29—H29C	0.9800	C7—H7C	0.9800
C30—C31	1.298 (11)	C8—C9	1.303 (10)
C31—C32	1.312 (11)	C9—C10	1.296 (11)
C32—H32A	0.98 (4)	C10—H10A	0.99 (4)
C32—H32B	0.98 (4)	C10—H10B	0.98 (4)
C33—H33A	0.9800	C11—H11A	0.9800
C33—H33B	0.9800	C11—H11B	0.9800
C33—H33C	0.9800	C11—H11C	0.9800
Br5—C63	1.932 (7)	Br2—C19	1.939 (7)

O9—C56	1.215 (9)	O3—C12	1.205 (9)
O10—C57	1.430 (8)	O4—C13	1.427 (8)
O10—H10O	0.8400	O4—H4O	0.8400
C56—C61	1.541 (9)	C12—C13	1.537 (9)
C56—C57	1.547 (9)	C12—C17	1.549 (9)
C57—C58	1.517 (9)	C13—C14	1.522 (10)
C57—C62	1.528 (9)	C13—C18	1.528 (10)
C58—C59	1.531 (10)	C14—C15	1.529 (10)
C58—H58A	0.9900	C14—H14A	0.9900
C58—H58B	0.9900	C14—H14B	0.9900
C59—C60	1.541 (11)	C15—C16	1.514 (11)
C59—H59A	0.9900	C15—H15A	0.9900
C59—H59B	0.9900	C15—H15B	0.9900
C60—C61	1.535 (10)	C16—C17	1.539 (10)
C60—H60A	0.9900	C16—H16A	0.9900
C60—H60B	0.9900	C16—H16B	0.9900
C61—C66	1.534 (10)	C17—C19	1.510 (9)
C61—C63	1.534 (9)	C17—C22	1.527 (10)
C62—H62A	0.9800	C18—H18A	0.9800
C62—H62B	0.9800	C18—H18B	0.9800
C62—H62C	0.9800	C18—H18C	0.9800
C63—C64	1.304 (11)	C19—C20	1.312 (10)
C64—C65	1.284 (12)	C20—C21	1.288 (10)
C65—H65A	0.99 (4)	C21—H21B	0.99 (4)
C65—H65B	0.99 (4)	C21—H21A	0.98 (4)
C66—H66A	0.9800	C22—H22A	0.9800
C66—H66B	0.9800	C22—H22B	0.9800
C66—H66C	0.9800	C22—H22C	0.9800
Br6—C52	1.924 (8)	Br4—C41	1.931 (7)
O11—C45	1.207 (9)	O7—C34	1.201 (9)
O12—C46	1.434 (8)	O8—C35	1.415 (9)
O12—H12O	0.8400	O8—H8O	0.8400
C45—C46	1.529 (9)	C34—C35	1.538 (11)
C45—C50	1.542 (10)	C34—C39	1.541 (10)
C46—C47	1.518 (9)	C35—C40	1.535 (11)
C46—C51	1.533 (9)	C35—C36	1.538 (12)
C47—C48	1.537 (10)	C36—C37	1.516 (11)
C47—H47A	0.9900	C36—H36A	0.9900
C47—H47B	0.9900	C36—H36B	0.9900

C48—C49	1.532 (10)	C37—C38	1.513 (11)
C48—H48A	0.9900	C37—H37A	0.9900
C48—H48B	0.9900	C37—H37B	0.9900
C49—C50	1.529 (11)	C38—C39	1.544 (10)
C49—H49A	0.9900	C38—H38A	0.9900
C49—H49B	0.9900	C38—H38B	0.9900
C50—C52	1.526 (12)	C39—C44	1.522 (10)
C50—C55	1.536 (10)	C39—C41	1.531 (9)
C51—H51A	0.9800	C40—H40A	0.9800
C51—H51B	0.9800	C40—H40B	0.9800
C51—H51C	0.9800	C40—H40C	0.9800
C52—C53	1.326 (11)	C41—C42	1.301 (10)
C53—C54	1.296 (12)	C42—C43	1.286 (11)
C54—H54B	0.98 (4)	C43—H43B	0.98 (4)
C54—H54A	0.98 (4)	C43—H43A	0.98 (4)
C55—H55A	0.9800	C44—H44A	0.9800
C55—H55B	0.9800	C44—H44B	0.9800
C55—H55C	0.9800	C44—H44C	0.9800
C24—O6—H6O	109.5	C2—O2—H2O	109.5
O5—C23—C24	121.3 (6)	O1—C1—C2	120.5 (6)
O5—C23—C28	118.4 (6)	O1—C1—C6	118.8 (6)
C24—C23—C28	120.3 (6)	C2—C1—C6	120.5 (6)
O6—C24—C23	108.5 (5)	O2—C2—C7	110.1 (6)
O6—C24—C25	106.3 (6)	O2—C2—C1	108.3 (5)
C23—C24—C25	111.1 (5)	C7—C2—C1	109.9 (5)
O6—C24—C29	110.0 (5)	O2—C2—C3	106.8 (5)
C23—C24—C29	108.7 (6)	C7—C2—C3	112.2 (6)
C25—C24—C29	112.3 (6)	C1—C2—C3	109.4 (6)
C26—C25—C24	113.5 (6)	C4—C3—C2	112.0 (6)
C26—C25—H25A	108.9	C4—C3—H3A	109.2
C24—C25—H25A	108.9	C2—C3—H3A	109.2
C26—C25—H25B	108.9	C4—C3—H3B	109.2
C24—C25—H25B	108.9	C2—C3—H3B	109.2
H25A—C25—H25B	107.7	H3A—C3—H3B	107.9
C25—C26—C27	111.0 (6)	C5—C4—C3	111.1 (6)
C25—C26—H26A	109.4	C5—C4—H4A	109.4
C27—C26—H26A	109.4	C3—C4—H4A	109.4
C25—C26—H26B	109.4	C5—C4—H4B	109.4

C27—C26—H26B	109.4	C3—C4—H4B	109.4
H26A—C26—H26B	108.0	H4A—C4—H4B	108.0
C26—C27—C28	113.9 (6)	C4—C5—C6	114.1 (6)
C26—C27—H27A	108.8	C4—C5—H5A	108.7
C28—C27—H27A	108.8	C6—C5—H5A	108.7
C26—C27—H27B	108.8	C4—C5—H5B	108.7
C28—C27—H27B	108.8	C6—C5—H5B	108.7
H27A—C27—H27B	107.7	H5A—C5—H5B	107.6
C30—C28—C33	109.0 (6)	C8—C6—C11	108.2 (6)
C30—C28—C23	110.6 (6)	C8—C6—C1	109.9 (5)
C33—C28—C23	109.6 (6)	C11—C6—C1	110.1 (6)
C30—C28—C27	109.1 (6)	C8—C6—C5	110.4 (6)
C33—C28—C27	109.8 (6)	C11—C6—C5	110.9 (6)
C23—C28—C27	108.8 (5)	C1—C6—C5	107.3 (6)
C24—C29—H29A	109.5	C2—C7—H7A	109.5
C24—C29—H29B	109.5	C2—C7—H7B	109.5
H29A—C29—H29B	109.5	H7A—C7—H7B	109.5
C24—C29—H29C	109.5	C2—C7—H7C	109.5
H29A—C29—H29C	109.5	H7A—C7—H7C	109.5
H29B—C29—H29C	109.5	H7B—C7—H7C	109.5
C31—C30—C28	127.0 (7)	C9—C8—C6	127.0 (7)
C31—C30—Br3	117.1 (6)	C9—C8—Br1	117.2 (6)
C28—C30—Br3	115.7 (5)	C6—C8—Br1	115.5 (5)
C30—C31—C32	179.3 (9)	C10—C9—C8	179.2 (8)
C31—C32—H32A	116 (6)	C9—C10—H10A	124 (7)
C31—C32—H32B	112 (6)	C9—C10—H10B	124 (7)
H32A—C32—H32B	130 (9)	H10A—C10—H10B	111 (9)
C28—C33—H33A	109.5	C6—C11—H11A	109.5
C28—C33—H33B	109.5	C6—C11—H11B	109.5
H33A—C33—H33B	109.5	H11A—C11—H11B	109.5
C28—C33—H33C	109.5	C6—C11—H11C	109.5
H33A—C33—H33C	109.5	H11A—C11—H11C	109.5
H33B—C33—H33C	109.5	H11B—C11—H11C	109.5
C57—O10—H10O	109.5	C13—O4—H4O	109.5
O9—C56—C61	119.7 (6)	O3—C12—C13	119.8 (6)
O9—C56—C57	120.9 (6)	O3—C12—C17	121.5 (6)
C61—C56—C57	119.3 (6)	C13—C12—C17	118.7 (6)
O10—C57—C58	107.2 (5)	O4—C13—C14	109.4 (6)
O10—C57—C62	109.4 (5)	O4—C13—C18	105.6 (6)

C58—C57—C62	112.5 (6)	C14—C13—C18	113.1 (6)
O10—C57—C56	108.3 (5)	O4—C13—C12	109.0 (6)
C58—C57—C56	109.5 (5)	C14—C13—C12	110.0 (6)
C62—C57—C56	109.8 (5)	C18—C13—C12	109.6 (6)
C57—C58—C59	112.4 (6)	C13—C14—C15	112.7 (6)
C57—C58—H58A	109.1	C13—C14—H14A	109.1
C59—C58—H58A	109.1	C15—C14—H14A	109.1
C57—C58—H58B	109.1	C13—C14—H14B	109.1
C59—C58—H58B	109.1	C15—C14—H14B	109.1
H58A—C58—H58B	107.9	H14A—C14—H14B	107.8
C58—C59—C60	110.5 (6)	C16—C15—C14	110.1 (6)
C58—C59—H59A	109.6	C16—C15—H15A	109.6
C60—C59—H59A	109.6	C14—C15—H15A	109.6
C58—C59—H59B	109.6	C16—C15—H15B	109.6
C60—C59—H59B	109.6	C14—C15—H15B	109.6
H59A—C59—H59B	108.1	H15A—C15—H15B	108.2
C61—C60—C59	114.3 (5)	C15—C16—C17	114.3 (6)
C61—C60—H60A	108.7	C15—C16—H16A	108.7
C59—C60—H60A	108.7	C17—C16—H16A	108.7
C61—C60—H60B	108.7	C15—C16—H16B	108.7
C59—C60—H60B	108.7	C17—C16—H16B	108.7
H60A—C60—H60B	107.6	H16A—C16—H16B	107.6
C66—C61—C63	107.8 (6)	C19—C17—C22	110.4 (6)
C66—C61—C60	109.8 (6)	C19—C17—C16	109.7 (6)
C63—C61—C60	110.0 (6)	C22—C17—C16	108.4 (6)
C66—C61—C56	110.0 (6)	C19—C17—C12	109.8 (6)
C63—C61—C56	109.8 (5)	C22—C17—C12	108.5 (6)
C60—C61—C56	109.4 (6)	C16—C17—C12	110.0 (5)
C57—C62—H62A	109.5	C13—C18—H18A	109.5
C57—C62—H62B	109.5	C13—C18—H18B	109.5
H62A—C62—H62B	109.5	H18A—C18—H18B	109.5
C57—C62—H62C	109.5	C13—C18—H18C	109.5
H62A—C62—H62C	109.5	H18A—C18—H18C	109.5
H62B—C62—H62C	109.5	H18B—C18—H18C	109.5
C64—C63—C61	126.4 (7)	C20—C19—C17	127.9 (6)
C64—C63—Br5	118.1 (5)	C20—C19—Br2	116.1 (5)
C61—C63—Br5	115.3 (5)	C17—C19—Br2	116.0 (5)
C65—C64—C63	179.6 (10)	C21—C20—C19	177.8 (8)
C64—C65—H65A	120 (6)	C20—C21—H21B	126 (6)

C64—C65—H65B	124 (7)	C20—C21—H21A	110 (6)
H65A—C65—H65B	115 (9)	H21B—C21—H21A	123 (8)
C61—C66—H66A	109.5	C17—C22—H22A	109.5
C61—C66—H66B	109.5	C17—C22—H22B	109.5
H66A—C66—H66B	109.5	H22A—C22—H22B	109.5
C61—C66—H66C	109.5	C17—C22—H22C	109.5
H66A—C66—H66C	109.5	H22A—C22—H22C	109.5
H66B—C66—H66C	109.5	H22B—C22—H22C	109.5
C46—O12—H12O	109.5	C35—O8—H8O	109.5
O11—C45—C46	120.4 (6)	O7—C34—C35	121.8 (7)
O11—C45—C50	120.4 (6)	O7—C34—C39	119.4 (7)
C46—C45—C50	119.1 (6)	C35—C34—C39	118.7 (6)
O12—C46—C47	105.3 (5)	O8—C35—C40	105.0 (6)
O12—C46—C45	109.3 (5)	O8—C35—C34	108.9 (7)
C47—C46—C45	112.4 (6)	C40—C35—C34	110.4 (7)
O12—C46—C51	109.5 (5)	O8—C35—C36	110.9 (7)
C47—C46—C51	111.8 (6)	C40—C35—C36	112.2 (8)
C45—C46—C51	108.4 (6)	C34—C35—C36	109.4 (6)
C46—C47—C48	113.2 (6)	C37—C36—C35	111.9 (6)
C46—C47—H47A	108.9	C37—C36—H36A	109.2
C48—C47—H47A	108.9	C35—C36—H36A	109.2
C46—C47—H47B	108.9	C37—C36—H36B	109.2
C48—C47—H47B	108.9	C35—C36—H36B	109.2
H47A—C47—H47B	107.7	H36A—C36—H36B	107.9
C49—C48—C47	109.5 (6)	C38—C37—C36	110.9 (6)
C49—C48—H48A	109.8	C38—C37—H37A	109.5
C47—C48—H48A	109.8	C36—C37—H37A	109.5
C49—C48—H48B	109.8	C38—C37—H37B	109.5
C47—C48—H48B	109.8	C36—C37—H37B	109.5
H48A—C48—H48B	108.2	H37A—C37—H37B	108.0
C50—C49—C48	114.8 (6)	C37—C38—C39	114.2 (6)
C50—C49—H49A	108.6	C37—C38—H38A	108.7
C48—C49—H49A	108.6	C39—C38—H38A	108.7
C50—C49—H49B	108.6	C37—C38—H38B	108.7
C48—C49—H49B	108.6	C39—C38—H38B	108.7
H49A—C49—H49B	107.6	H38A—C38—H38B	107.6
C52—C50—C49	110.4 (6)	C44—C39—C41	108.6 (6)
C52—C50—C55	108.2 (7)	C44—C39—C34	111.1 (6)
C49—C50—C55	110.2 (7)	C41—C39—C34	110.2 (6)

C52—C50—C45	108.3 (6)	C44—C39—C38	108.7 (7)
C49—C50—C45	110.2 (6)	C41—C39—C38	109.1 (5)
C55—C50—C45	109.5 (6)	C34—C39—C38	109.1 (6)
C46—C51—H51A	109.5	C35—C40—H40A	109.5
C46—C51—H51B	109.5	C35—C40—H40B	109.5
H51A—C51—H51B	109.5	H40A—C40—H40B	109.5
C46—C51—H51C	109.5	C35—C40—H40C	109.5
H51A—C51—H51C	109.5	H40A—C40—H40C	109.5
H51B—C51—H51C	109.5	H40B—C40—H40C	109.5
C53—C52—C50	125.9 (8)	C42—C41—C39	128.0 (6)
C53—C52—Br6	117.0 (7)	C42—C41—Br4	116.1 (5)
C50—C52—Br6	117.0 (5)	C39—C41—Br4	115.8 (5)
C54—C53—C52	177.6 (11)	C43—C42—C41	178.2 (8)
C53—C54—H54B	117 (9)	C42—C43—H43B	122 (7)
C53—C54—H54A	120 (9)	C42—C43—H43A	119 (6)
H54B—C54—H54A	122 (10)	H43B—C43—H43A	119 (9)
C50—C55—H55A	109.5	C39—C44—H44A	109.5
C50—C55—H55B	109.5	C39—C44—H44B	109.5
H55A—C55—H55B	109.5	H44A—C44—H44B	109.5
C50—C55—H55C	109.5	C39—C44—H44C	109.5
H55A—C55—H55C	109.5	H44A—C44—H44C	109.5
H55B—C55—H55C	109.5	H44B—C44—H44C	109.5
O5—C23—C24—O6	21.6 (9)	O1—C1—C2—O2	12.3 (8)
C28—C23—C24—O6	-158.9 (6)	C6—C1—C2—O2	-163.1 (6)
O5—C23—C24—C25	138.1 (7)	O1—C1—C2—C7	-108.0 (7)
C28—C23—C24—C25	-42.4 (8)	C6—C1—C2—C7	76.6 (7)
O5—C23—C24—C29	-97.9 (8)	O1—C1—C2—C3	128.5 (7)
C28—C23—C24—C29	81.5 (7)	C6—C1—C2—C3	-47.0 (8)
O6—C24—C25—C26	165.4 (6)	O2—C2—C3—C4	167.3 (6)
C23—C24—C25—C26	47.6 (8)	C7—C2—C3—C4	-72.0 (9)
C29—C24—C25—C26	-74.3 (8)	C1—C2—C3—C4	50.2 (8)
C24—C25—C26—C27	-56.5 (8)	C2—C3—C4—C5	-58.4 (9)
C25—C26—C27—C28	57.8 (8)	C3—C4—C5—C6	59.0 (9)

O5—C23—C28—C30	102.2 (7)	O1—C1—C6—C8	110.0 (7)
C24—C23—C28—C30	-77.3 (7)	C2—C1—C6—C8	-74.5 (7)
O5—C23—C28—C33	-18.0 (9)	O1—C1—C6—C11	-9.1 (9)
C24—C23—C28—C33	162.5 (6)	C2—C1—C6—C11	166.4 (6)
O5—C23—C28—C27	-138.0 (6)	O1—C1—C6—C5	-129.9 (7)
C24—C23—C28—C27	42.5 (8)	C2—C1—C6—C5	45.6 (8)
C26—C27—C28—C30	72.0 (7)	C4—C5—C6—C8	70.0 (8)
C26—C27—C28—C33	-168.6 (6)	C4—C5—C6—C11	-170.1 (7)
C26—C27—C28—C23	-48.7 (8)	C4—C5—C6—C1	-49.8 (8)
C33—C28—C30—C31	-106.9 (9)	C11—C6—C8—C9	-102.0 (9)
C23—C28—C30—C31	132.5 (8)	C1—C6—C8—C9	137.7 (8)
C27—C28—C30—C31	13.0 (10)	C5—C6—C8—C9	19.5 (10)
C33—C28—C30—Br3	68.0 (7)	C11—C6—C8—Br1	71.7 (7)
C23—C28—C30—Br3	-52.6 (7)	C1—C6—C8—Br1	-48.6 (7)
C27—C28—C30—Br3	-172.2 (5)	C5—C6—C8—Br1	-166.8 (5)
O9—C56—C57—O10	12.6 (8)	O3—C12—C13—O4	14.7 (9)
C61—C56—C57—O10	-163.7 (5)	C17—C12—C13—O4	-165.0 (5)
O9—C56—C57—C58	129.3 (7)	O3—C12—C13—C14	134.7 (7)
C61—C56—C57—C58	-47.1 (8)	C17—C12—C13—C14	-45.0 (8)
O9—C56—C57—C62	-106.8 (7)	O3—C12—C13—C18	-100.4 (8)
C61—C56—C57—C62	76.8 (7)	C17—C12—C13—C18	79.9 (7)
O10—C57—C58—C59	170.2 (6)	O4—C13—C14—C15	172.1 (6)
C62—C57—C58—C59	-69.4 (7)	C18—C13—C14—C15	-70.5 (8)

C56—C57—C58—C59	52.9 (8)	C12—C13—C14—C15	52.4 (8)
C57—C58—C59—C60	-59.1 (7)	C13—C14—C15—C16	-59.8 (8)
C58—C59—C60—C61	56.3 (8)	C14—C15—C16—C17	57.4 (8)
C59—C60—C61—C66	-167.8 (6)	C15—C16—C17—C19	73.5 (7)
C59—C60—C61—C63	73.8 (7)	C15—C16—C17—C22	-165.8 (6)
C59—C60—C61—C56	-47.0 (8)	C15—C16—C17—C12	-47.3 (8)
O9—C56—C61—C66	-12.1 (9)	O3—C12—C17—C19	101.5 (8)
C57—C56—C61—C66	164.3 (6)	C13—C12—C17—C19	-78.8 (7)
O9—C56—C61—C63	106.4 (7)	O3—C12—C17—C22	-19.3 (9)
C57—C56—C61—C63	-77.2 (7)	C13—C12—C17—C22	160.4 (6)
O9—C56—C61—C60	-132.7 (7)	O3—C12—C17—C16	-137.7 (7)
C57—C56—C61—C60	43.7 (8)	C13—C12—C17—C16	42.0 (8)
C66—C61—C63—C64	-101.7 (9)	C22—C17—C19—C20	-117.4 (9)
C60—C61—C63—C64	18.0 (10)	C16—C17—C19—C20	2.0 (10)
C56—C61—C63—C64	138.5 (8)	C12—C17—C19—C20	123.0 (8)
C66—C61—C63—Br5	72.2 (7)	C22—C17—C19—Br2	60.2 (7)
C60—C61—C63—Br5	-168.1 (5)	C16—C17—C19—Br2	179.7 (5)
C56—C61—C63—Br5	-47.6 (7)	C12—C17—C19—Br2	-59.4 (7)
O11—C45—C46—O12	22.6 (9)	O7—C34—C35—O8	8.2 (11)
C50—C45—C46—O12	-157.3 (6)	C39—C34—C35—O8	-169.0 (6)
O11—C45—C46—C47	139.2 (7)	O7—C34—C35—C40	-106.6 (9)

C50—C45—C46—C47	-40.7 (8)	C39—C34—C35—C40	76.3 (9)
O11—C45—C46—C51	-96.6 (8)	O7—C34—C35—C36	129.5 (8)
C50—C45—C46—C51	83.4 (8)	C39—C34—C35—C36	-47.6 (8)
O12—C46—C47—C48	167.1 (6)	O8—C35—C36—C37	173.3 (7)
C45—C46—C47—C48	48.2 (8)	C40—C35—C36—C37	-69.7 (9)
C51—C46—C47—C48	-74.0 (7)	C34—C35—C36—C37	53.1 (8)
C46—C47—C48—C49	-56.7 (8)	C35—C36—C37—C38	-59.6 (8)
C47—C48—C49—C50	57.6 (9)	C36—C37—C38—C39	57.4 (8)
C48—C49—C50—C52	71.4 (8)	O7—C34—C39—C44	-13.0 (10)
C48—C49—C50—C55	-169.2 (7)	C35—C34—C39—C44	164.3 (7)
C48—C49—C50—C45	-48.2 (9)	O7—C34—C39—C41	107.5 (8)
O11—C45—C50—C52	99.1 (8)	C35—C34—C39—C41	-75.3 (8)
C46—C45—C50—C52	-81.0 (8)	O7—C34—C39—C38	-132.8 (7)
O11—C45—C50—C49	-140.0 (7)	C35—C34—C39—C38	44.4 (8)
C46—C45—C50—C49	39.9 (9)	C37—C38—C39—C44	-169.2 (6)
O11—C45—C50—C55	-18.6 (10)	C37—C38—C39—C41	72.6 (7)
C46—C45—C50—C55	161.3 (7)	C37—C38—C39—C34	-47.8 (8)
C49—C50—C52—C53	10.9 (10)	C44—C39—C41—C42	-111.5 (9)
C55—C50—C52—C53	-109.7 (9)	C34—C39—C41—C42	126.5 (8)
C45—C50—C52—C53	131.7 (8)	C38—C39—C41—C42	6.7 (10)
C49—C50—C52—Br6	-174.0 (5)	C44—C39—C41—Br4	65.6 (7)

C55—C50—C52—Br6	65.4 (7)	C34—C39—C41—Br4	-56.3 (7)
C45—C50—C52—Br6	-53.2 (7)	C38—C39—C41—Br4	-176.1 (5)

Hydrogen-bond geometry (Å, º) for (EAG3124P)

D—H···*A*	*D*—H	H···*A*	*D*···*A*	*D*—H···*A*
O6—H6*O*···O1	0.84	2.05	2.886 (7)	176
O10—H10*O*···O11	0.84	2.08	2.880 (7)	159
C62—H62*B*···O8^{i}	0.98	2.63	3.429 (10)	139
C62—H62*C*···O11	0.98	2.58	3.318 (9)	132
O12—H12*O*···O9	0.84	2.05	2.840 (7)	156
C47—H47*A*···O8	0.99	2.59	3.426 (9)	142
C51—H51*B*···Br1^{ii}	0.98	2.97	3.678 (7)	130
C7—H7*B*···O4^{iii}	0.98	2.42	3.193 (8)	136
C7—H7*C*···O5	0.98	2.56	3.252 (9)	127
O4—H4*O*···O6	0.84	2.13	2.951 (7)	165
C14—H14*B*···Br4^{iv}	0.99	3.12	4.029 (7)	154
C18—H18*B*···Br4^{iv}	0.98	3.12	3.928 (7)	140
O8—H8*O*···O12	0.84	2.06	2.847 (8)	155
C36—H36*A*···Br2	0.99	3.00	3.759 (8)	135

Symmetry codes: (i) *x*+1, *y*, *z*; (ii) -*x*, *y*+1/2, -*z*+1; (iii) *x*-1, *y*, *z*; (iv) *x*, *y*, *z*-1.

Compound (+)-9a

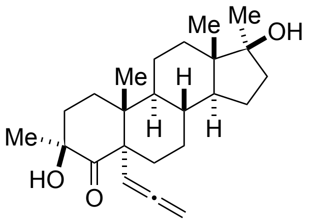

(+)-9a

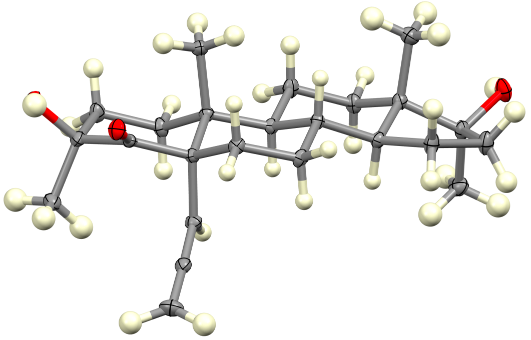

Sample Name: CCDC 2426292 / Armend10 (EAG3071P)
Crystal Growth: Slow evaporation using a mixture of hexanes and dichloromethane
Ellipsoid Contour: Set at a 50% probability level

Crystal data

$C_{24}H_{36}O_3$	D_x = 1.230 Mg m^{-3}
M_r = 372.53	Cu *K*a radiation, l = 1.54184 Å
Orthorhombic, $P2_12_12_1$	Cell parameters from 9387 reflections
a = 6.4724 (2) Å	q = 4.3–78.5°
b = 10.9824 (3) Å	m = 0.62 mm^{-1}
c = 28.2896 (8) Å	*T* = 100 K
V = 2010.90 (10) Å^3	Lath fragment, colourless
Z = 4	0.20 × 0.18 × 0.08 mm
F(000) = 816	

Data collection

Bruker D8 Venture DUO with Photon III C14 diffractometer	4166 reflections with *I* > 2s(*I*)
Radiation source: ImS 3.0 microfocus	R_{int} = 0.030
f and w scans	q_{max} = 79.1°, q_{min} = 3.1°
Absorption correction: multi-scan *SADABS* (Krause *et al.*, 2015)	*h* = -7 - 8
T_{min} = 0.840, T_{max} = 0.952	*k* = -13 - 13
47662 measured reflections	*l* = -35 - 35
4211 independent reflections	

Refinement

Refinement on F^2	Hydrogen site location: mixed
Least-squares matrix: full	H atoms treated by a mixture of independent and constrained refinement
$R[F^2 > 2s(F^2)] = 0.027$	$w = 1/[s^2(F_o^2) + (0.0439P)^2 + 0.3654P]$ where $P = (F_o^2 + 2F_c^2)/3$
$wR(F^2) = 0.075$	$(D/s)_{max} = 0.001$
$S = 1.07$	$Dr_{max} = 0.23$ e Å^{-3}
4211 reflections	$Dr_{min} = -0.18$ e Å^{-3}
260 parameters	Absolute structure: Flack x determined using 1718 quotients [(I+)-(I-)]/[(I+)+(I-)] (Parsons, Flack and Wagner, Acta Cryst. B69 (2013) 249-259).
0 restraints	Absolute structure parameter: -0.01 (3)

Fractional atomic coordinates and isotropic or equivalent isotropic displacement parameters (Å^2) for (EAG3071)

	x	*y*	*z*	$U_{iso}*/U_{eq}$
O1	0.65772 (17)	1.06531 (9)	0.58832 (4)	0.0208 (2)
H10H	0.579 (4)	1.1065 (19)	0.6059 (8)	0.031*
O2	0.18863 (17)	0.17374 (10)	0.73342 (4)	0.0211 (2)
H20H	0.255 (4)	0.122 (2)	0.7160 (7)	0.032*
O3	0.38779 (16)	0.18850 (9)	0.65363 (4)	0.0187 (2)
C1	0.5222 (2)	0.74081 (12)	0.58062 (4)	0.0130 (3)
H1	0.380836	0.747622	0.566594	0.016*
C2	0.6745 (2)	0.76007 (13)	0.53981 (5)	0.0195 (3)
H2A	0.626966	0.717948	0.510849	0.023*
H2B	0.813883	0.729908	0.548226	0.023*
C3	0.6755 (3)	0.90036 (13)	0.53280 (5)	0.0210 (3)
H3A	0.818984	0.931518	0.533147	0.025*
H3B	0.611855	0.921696	0.502070	0.025*
C4	0.5499 (2)	0.95724 (12)	0.57390 (5)	0.0164 (3)
C5	0.5511 (2)	0.85414 (12)	0.61206 (5)	0.0139 (3)
C6	0.3786 (2)	0.85588 (12)	0.64916 (5)	0.0156 (3)
H6A	0.244693	0.871145	0.633297	0.019*
H6B	0.403649	0.923395	0.671624	0.019*
C7	0.3663 (2)	0.73517 (12)	0.67663 (5)	0.0164 (3)
H7A	0.246306	0.737945	0.698308	0.020*
H7B	0.492482	0.725853	0.696063	0.020*
C8	0.3448 (2)	0.62370 (12)	0.64377 (4)	0.0122 (3)

H8	0.215660	0.636250	0.624987	0.015*
C9	0.5261 (2)	0.62064 (12)	0.60779 (4)	0.0124 (3)
H9	0.658934	0.615548	0.625738	0.015*
C10	0.5127 (2)	0.51116 (12)	0.57441 (4)	0.0143 (3)
H10A	0.393672	0.522346	0.552852	0.017*
H10B	0.639481	0.507767	0.554882	0.017*
C11	0.4879 (2)	0.39071 (12)	0.60075 (4)	0.0136 (3)
H11A	0.613522	0.374449	0.619706	0.016*
H11B	0.471524	0.323599	0.577691	0.016*
C12	0.2982 (2)	0.39518 (12)	0.63350 (4)	0.0120 (3)
C13	0.3182 (2)	0.50126 (12)	0.67058 (4)	0.0120 (3)
C14	0.1211 (2)	0.50132 (12)	0.70126 (5)	0.0160 (3)
H14A	0.125166	0.571911	0.722999	0.019*
H14B	-0.001142	0.510819	0.680538	0.019*
C15	0.0981 (2)	0.38406 (13)	0.73025 (5)	0.0183 (3)
H15A	-0.038758	0.384741	0.745864	0.022*
H15B	0.204482	0.383874	0.755407	0.022*
C16	0.1184 (2)	0.26606 (12)	0.70158 (5)	0.0150 (3)
C17	0.2790 (2)	0.27667 (12)	0.66153 (4)	0.0132 (3)
C18	0.1035 (2)	0.40816 (12)	0.60264 (5)	0.0147 (3)
H18	0.014879	0.475609	0.608196	0.018*
C19	0.0536 (2)	0.33162 (13)	0.56930 (5)	0.0161 (3)
C20	0.0025 (3)	0.25352 (15)	0.53690 (5)	0.0239 (3)
H20A	0.059 (3)	0.2632 (19)	0.5045 (7)	0.029*
H20B	-0.088 (3)	0.1844 (18)	0.5450 (7)	0.029*
C21	0.3330 (2)	0.99408 (13)	0.55799 (5)	0.0209 (3)
H21A	0.256272	0.921441	0.548137	0.031*
H21B	0.343074	1.050865	0.531365	0.031*
H21C	0.260469	1.033556	0.584259	0.031*
C22	0.7632 (2)	0.85499 (14)	0.63673 (5)	0.0198 (3)
H22A	0.777648	0.929809	0.655322	0.030*
H22B	0.872858	0.851473	0.612886	0.030*
H22C	0.774057	0.784235	0.657695	0.030*
C23	0.5053 (2)	0.47795 (13)	0.70322 (5)	0.0157 (3)
H23A	0.506746	0.392265	0.712890	0.024*
H23B	0.494777	0.529877	0.731291	0.024*
H23C	0.633204	0.496917	0.686199	0.024*
C24	-0.0882 (2)	0.22576 (13)	0.68054 (5)	0.0198 (3)
H24A	-0.146432	0.292182	0.661626	0.030*

H24B	-0.184008	0.204957	0.706119	0.030*
H24C	-0.066580	0.154309	0.660383	0.030*

Atomic displacement parameters (Å^2) for (EAG3071)

	U^{11}	U^{22}	U^{33}	U^{12}	U^{13}	U^{23}
O1	0.0254 (6)	0.0138 (5)	0.0232 (5)	-0.0061 (4)	0.0061 (4)	-0.0032 (4)
O2	0.0209 (5)	0.0228 (5)	0.0196 (5)	0.0033 (5)	-0.0006 (4)	0.0095 (4)
O3	0.0214 (5)	0.0132 (5)	0.0214 (5)	0.0023 (4)	0.0015 (4)	0.0012 (4)
C1	0.0142 (6)	0.0130 (6)	0.0117 (6)	0.0003 (5)	0.0010 (5)	0.0002 (5)
C2	0.0262 (7)	0.0158 (6)	0.0164 (6)	-0.0001 (6)	0.0079 (6)	0.0013 (5)
C3	0.0293 (8)	0.0166 (7)	0.0170 (6)	-0.0030 (6)	0.0074 (6)	0.0009 (5)
C4	0.0206 (7)	0.0128 (6)	0.0156 (6)	-0.0031 (5)	0.0023 (5)	-0.0001 (5)
C5	0.0147 (6)	0.0130 (6)	0.0141 (6)	-0.0014 (5)	0.0014 (5)	-0.0004 (5)
C6	0.0186 (7)	0.0124 (6)	0.0159 (6)	-0.0008 (5)	0.0044 (5)	-0.0018 (5)
C7	0.0214 (7)	0.0143 (6)	0.0134 (6)	-0.0016 (5)	0.0048 (5)	-0.0019 (5)
C8	0.0117 (6)	0.0130 (6)	0.0119 (5)	0.0003 (5)	0.0012 (5)	-0.0003 (5)
C9	0.0123 (6)	0.0127 (6)	0.0123 (5)	0.0005 (5)	0.0018 (5)	0.0003 (5)
C10	0.0172 (6)	0.0138 (6)	0.0119 (6)	0.0017 (5)	0.0031 (5)	0.0000 (5)
C11	0.0150 (6)	0.0129 (6)	0.0128 (6)	0.0022 (5)	0.0011 (5)	-0.0007 (5)
C12	0.0121 (6)	0.0121 (6)	0.0118 (5)	0.0002 (5)	-0.0007 (5)	0.0000 (5)
C13	0.0123 (6)	0.0125 (6)	0.0112 (5)	-0.0006 (5)	0.0000 (5)	-0.0009 (5)
C14	0.0163 (6)	0.0152 (6)	0.0167 (6)	-0.0015 (5)	0.0051 (5)	-0.0010 (5)
C15	0.0207 (7)	0.0188 (6)	0.0153 (6)	-0.0037 (6)	0.0049 (5)	0.0009 (5)
C16	0.0140 (6)	0.0157 (6)	0.0153 (6)	-0.0009 (5)	-0.0013 (5)	0.0047 (5)
C17	0.0126 (6)	0.0133 (6)	0.0136 (5)	-0.0010 (5)	-0.0038 (5)	-0.0008 (5)

C18	0.0148 (6)	0.0136 (6)	0.0157 (6)	0.0011 (5)	-0.0026 (5)	0.0007 (5)
C19	0.0146 (6)	0.0174 (6)	0.0161 (6)	-0.0004 (5)	-0.0019 (5)	0.0045 (5)
C20	0.0221 (7)	0.0260 (8)	0.0234 (7)	-0.0012 (7)	-0.0067 (6)	-0.0062 (6)
C21	0.0267 (8)	0.0150 (6)	0.0210 (7)	0.0013 (6)	-0.0049 (6)	0.0015 (5)
C22	0.0171 (7)	0.0218 (7)	0.0204 (6)	-0.0034 (6)	-0.0018 (5)	-0.0001 (5)
C23	0.0167 (6)	0.0168 (6)	0.0135 (6)	-0.0018 (5)	-0.0029 (5)	0.0007 (5)
C24	0.0148 (6)	0.0216 (7)	0.0230 (7)	-0.0032 (6)	-0.0018 (5)	0.0048 (5)

Geometric parameters (Å, º) for (EAG3071)

O1—C4	1.4361 (16)	C11—C12	1.5391 (17)
O1—H10H	0.84 (2)	C11—H11A	0.9900
O2—C16	1.4303 (16)	C11—H11B	0.9900
O2—H20H	0.86 (2)	C12—C17	1.5291 (17)
O3—C17	1.2181 (17)	C12—C18	1.5397 (17)
C1—C9	1.5276 (17)	C12—C13	1.5730 (17)
C1—C2	1.5326 (18)	C13—C14	1.5428 (18)
C1—C5	1.5413 (17)	C13—C23	1.5446 (18)
C1—H1	1.0000	C14—C15	1.5341 (18)
C2—C3	1.5535 (19)	C14—H14A	0.9900
C2—H2A	0.9900	C14—H14B	0.9900
C2—H2B	0.9900	C15—C16	1.5344 (18)
C3—C4	1.5502 (19)	C15—H15A	0.9900
C3—H3A	0.9900	C15—H15B	0.9900
C3—H3B	0.9900	C16—C24	1.5293 (19)
C4—C21	1.529 (2)	C16—C17	1.5417 (18)
C4—C5	1.5643 (18)	C18—C19	1.3042 (19)
C5—C6	1.5325 (18)	C18—H18	0.9500
C5—C22	1.5402 (18)	C19—C20	1.298 (2)
C6—C7	1.5388 (18)	C20—H20A	0.99 (2)
C6—H6A	0.9900	C20—H20B	0.99 (2)
C6—H6B	0.9900	C21—H21A	0.9800
C7—C8	1.5432 (17)	C21—H21B	0.9800
C7—H7A	0.9900	C21—H21C	0.9800

C7—H7B	0.9900	C22—H22A	0.9800
C8—C13	1.5534 (17)	C22—H22B	0.9800
C8—C9	1.5538 (17)	C22—H22C	0.9800
C8—H8	1.0000	C23—H23A	0.9800
C9—C10	1.5314 (17)	C23—H23B	0.9800
C9—H9	1.0000	C23—H23C	0.9800
C10—C11	1.5267 (17)	C24—H24A	0.9800
C10—H10A	0.9900	C24—H24B	0.9800
C10—H10B	0.9900	C24—H24C	0.9800
C4—O1—H10H	108.6 (15)	C12—C11—H11B	109.6
C16—O2—H20H	105.2 (14)	H11A—C11—H11B	108.1
C9—C1—C2	119.19 (11)	C17—C12—C11	110.50 (10)
C9—C1—C5	113.90 (10)	C17—C12—C18	107.82 (10)
C2—C1—C5	104.19 (11)	C11—C12—C18	108.34 (10)
C9—C1—H1	106.2	C17—C12—C13	106.93 (9)
C2—C1—H1	106.2	C11—C12—C13	111.07 (10)
C5—C1—H1	106.2	C18—C12—C13	112.13 (10)
C1—C2—C3	103.63 (11)	C14—C13—C23	108.19 (10)
C1—C2—H2A	111.0	C14—C13—C8	111.46 (10)
C3—C2—H2A	111.0	C23—C13—C8	110.38 (10)
C1—C2—H2B	111.0	C14—C13—C12	107.91 (10)
C3—C2—H2B	111.0	C23—C13—C12	109.90 (10)
H2A—C2—H2B	109.0	C8—C13—C12	108.95 (10)
C4—C3—C2	107.56 (11)	C15—C14—C13	112.36 (11)
C4—C3—H3A	110.2	C15—C14—H14A	109.1
C2—C3—H3A	110.2	C13—C14—H14A	109.1
C4—C3—H3B	110.2	C15—C14—H14B	109.1
C2—C3—H3B	110.2	C13—C14—H14B	109.1
H3A—C3—H3B	108.5	H14A—C14—H14B	107.9
O1—C4—C21	108.13 (11)	C14—C15—C16	114.71 (10)
O1—C4—C3	106.93 (12)	C14—C15—H15A	108.6
C21—C4—C3	111.54 (12)	C16—C15—H15A	108.6
O1—C4—C5	113.56 (11)	C14—C15—H15B	108.6
C21—C4—C5	113.54 (11)	C16—C15—H15B	108.6
C3—C4—C5	102.91 (10)	H15A—C15—H15B	107.6
C6—C5—C22	109.82 (11)	O2—C16—C24	108.53 (11)
C6—C5—C1	108.48 (10)	O2—C16—C15	107.03 (10)
C22—C5—C1	112.00 (11)	C24—C16—C15	112.05 (12)

C6—C5—C4	117.38 (11)	O2—C16—C17	107.59 (11)
C22—C5—C4	108.23 (11)	C24—C16—C17	108.98 (10)
C1—C5—C4	100.70 (10)	C15—C16—C17	112.48 (11)
C5—C6—C7	111.88 (11)	O3—C17—C12	122.29 (12)
C5—C6—H6A	109.2	O3—C17—C16	117.68 (12)
C7—C6—H6A	109.2	C12—C17—C16	120.03 (11)
C5—C6—H6B	109.2	C19—C18—C12	123.58 (12)
C7—C6—H6B	109.2	C19—C18—H18	118.2
H6A—C6—H6B	107.9	C12—C18—H18	118.2
C6—C7—C8	112.58 (10)	C20—C19—C18	178.58 (15)
C6—C7—H7A	109.1	C19—C20—H20A	119.2 (12)
C8—C7—H7A	109.1	C19—C20—H20B	119.8 (12)
C6—C7—H7B	109.1	H20A—C20—H20B	121.0 (17)
C8—C7—H7B	109.1	C4—C21—H21A	109.5
H7A—C7—H7B	107.8	C4—C21—H21B	109.5
C7—C8—C13	113.75 (10)	H21A—C21—H21B	109.5
C7—C8—C9	110.10 (11)	C4—C21—H21C	109.5
C13—C8—C9	112.63 (10)	H21A—C21—H21C	109.5
C7—C8—H8	106.6	H21B—C21—H21C	109.5
C13—C8—H8	106.6	C5—C22—H22A	109.5
C9—C8—H8	106.6	C5—C22—H22B	109.5
C1—C9—C10	111.52 (10)	H22A—C22—H22B	109.5
C1—C9—C8	107.37 (10)	C5—C22—H22C	109.5
C10—C9—C8	112.22 (11)	H22A—C22—H22C	109.5
C1—C9—H9	108.5	H22B—C22—H22C	109.5
C10—C9—H9	108.5	C13—C23—H23A	109.5
C8—C9—H9	108.5	C13—C23—H23B	109.5
C11—C10—C9	112.65 (10)	H23A—C23—H23B	109.5
C11—C10—H10A	109.1	C13—C23—H23C	109.5
C9—C10—H10A	109.1	H23A—C23—H23C	109.5
C11—C10—H10B	109.1	H23B—C23—H23C	109.5
C9—C10—H10B	109.1	C16—C24—H24A	109.5
H10A—C10—H10B	107.8	C16—C24—H24B	109.5
C10—C11—C12	110.48 (10)	H24A—C24—H24B	109.5
C10—C11—H11A	109.6	C16—C24—H24C	109.5
C12—C11—H11A	109.6	H24A—C24—H24C	109.5
C10—C11—H11B	109.6	H24B—C24—H24C	109.5
C9—C1—C2—C3	-161.84 (12)	C10—C11—C12—	64.61 (13)

		C18	
C5—C1—C2—C3	-33.52 (14)	C10—C11—C12—C13	-58.97 (13)
C1—C2—C3—C4	7.92 (16)	C7—C8—C13—C14	61.08 (14)
C2—C3—C4—O1	139.88 (13)	C9—C8—C13—C14	-172.75 (10)
C2—C3—C4—C21	-102.08 (14)	C7—C8—C13—C23	-59.19 (14)
C2—C3—C4—C5	19.98 (15)	C9—C8—C13—C23	66.98 (13)
C9—C1—C5—C6	-58.72 (14)	C7—C8—C13—C12	-179.95 (11)
C2—C1—C5—C6	169.81 (11)	C9—C8—C13—C12	-53.78 (13)
C9—C1—C5—C22	62.65 (14)	C17—C12—C13—C14	-60.49 (13)
C2—C1—C5—C22	-68.82 (13)	C11—C12—C13—C14	178.87 (10)
C9—C1—C5—C4	177.46 (11)	C18—C12—C13—C14	57.49 (13)
C2—C1—C5—C4	45.99 (12)	C17—C12—C13—C23	57.28 (13)
O1—C4—C5—C6	87.57 (15)	C11—C12—C13—C23	-63.36 (13)
C21—C4—C5—C6	-36.51 (16)	C18—C12—C13—C23	175.26 (11)
C3—C4—C5—C6	-157.22 (12)	C17—C12—C13—C8	178.34 (10)
O1—C4—C5—C22	-37.36 (15)	C11—C12—C13—C8	57.70 (13)
C21—C4—C5—C22	-161.43 (11)	C18—C12—C13—C8	-63.68 (13)
C3—C4—C5—C22	77.85 (13)	C23—C13—C14—C15	-55.18 (14)
O1—C4—C5—C1	-154.98 (11)	C8—C13—C14—C15	-176.72 (11)
C21—C4—C5—C1	80.94 (13)	C12—C13—C14—C15	63.69 (14)
C3—C4—C5—C1	-39.77 (13)	C13—C14—C15—C16	-51.11 (16)
C22—C5—C6—C7	-69.62 (14)	C14—C15—C16—O2	153.69 (12)
C1—C5—C6—C7	53.08 (14)	C14—C15—C16—C24	-87.45 (14)
C4—C5—C6—C7	166.25 (11)	C14—C15—C16—C17	35.73 (16)
C5—C6—C7—C8	-54.32 (15)	C11—C12—C17—O3	-7.92 (17)
C6—C7—C8—C13	-176.37 (11)	C18—C12—C17—O3	110.31 (14)
C6—C7—C8—C9	56.14 (14)	C13—C12—C17—O3	-128.92 (13)
C2—C1—C9—C10	-52.13 (16)	C11—C12—C17—C16	171.71 (10)

C5—C1—C9—C10	-175.82 (11)	C18—C12—C17—C16	-70.06 (14)
C2—C1—C9—C8	-175.45 (11)	C13—C12—C17—C16	50.71 (14)
C5—C1—C9—C8	60.86 (14)	O2—C16—C17—O3	23.85 (16)
C7—C8—C9—C1	-57.37 (13)	C24—C16—C17—O3	-93.64 (14)
C13—C8—C9—C1	174.52 (10)	C15—C16—C17—O3	141.48 (13)
C7—C8—C9—C10	179.74 (10)	O2—C16—C17—C12	-155.80 (11)
C13—C8—C9—C10	51.63 (14)	C24—C16—C17—C12	86.72 (14)
C1—C9—C10—C11	-172.53 (11)	C15—C16—C17—C12	-38.17 (15)
C8—C9—C10—C11	-52.01 (15)	C17—C12—C18—C19	-62.76 (16)
C9—C10—C11—C12	55.81 (14)	C11—C12—C18—C19	56.85 (17)
C10—C11—C12—C17	-177.48 (10)	C13—C12—C18—C19	179.79 (13)

Hydrogen-bond geometry (Å, º) for (EAG3071)

D—H···*A*	*D*—H	H···*A*	*D*···*A*	*D*—H···*A*
O1—H10*H*···O3^{i}	0.84 (2)	2.04 (2)	2.8803 (15)	173 (2)
O2—H20*H*···O3	0.86 (2)	2.09 (2)	2.6044 (15)	117.1 (18)

Symmetry code: (i) *x*, *y*+1, *z*.

Compound (-)-9b

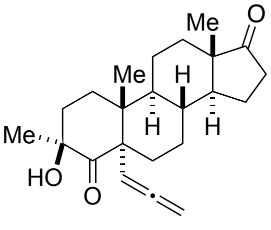

(-)-9b

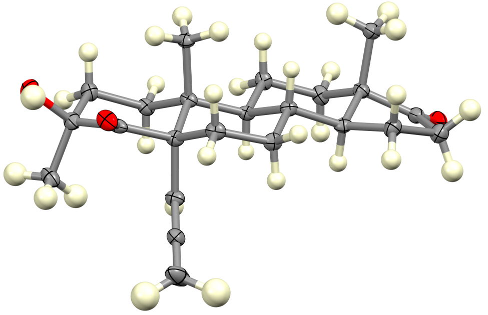

Sample Name: CCDC 2426293 / Armend11 (EDG3071A)
Crystal Growth: Slow evaporation using a mixture of hexanes and dichloromethane
Ellipsoid Contour: Set at a 50% probability level

Crystal data

$C_{23}H_{32}O_3$	*F*(000) = 388
M_r = 356.48	D_x = 1.191 Mg m^{-3}
Monoclinic, *P*2$_1$	Cu *K*a radiation, l = 1.54184 Å
a = 6.0995 (2) Å	Cell parameters from 9802 reflections
b = 15.2588 (5) Å	q = 4.1–79.0°
c = 10.7216 (4) Å	m = 0.60 mm^{-1}
b = 94.849 (2)°	*T* = 100 K
V = 994.30 (6) Å^3	Needle, colourless
Z = 2	0.25 × 0.15 × 0.01 mm

Data collection

Bruker D8 Venture DUO with Photon III C14 diffractometer	4010 reflections with *I* > 2s(*I*)
Radiation source: ImS 3.0 microfocus	R_{int} = 0.049
f and w scans	q_{max} = 79.7°, q_{min} = 4.1°
Absorption correction: multi-scan *SADABS* (Krause *et al.*, 2015)	*h* = -6 - 7
T_{min} = 0.766, T_{max} = 0.994	*k* = -19 - 19
19933 measured reflections	*l* = -13 - 13
4185 independent reflections	

Refinement

Refinement on F^2	Hydrogen site location: mixed

Least-squares matrix: full	H atoms treated by a mixture of independent and constrained refinement
$R[F^2 > 2s(F^2)] = 0.033$	$w = 1/[s^2(F_o^2) + (0.0451P)^2 + 0.1345P]$ where $P = (F_o^2 + 2F_c^2)/3$
$wR(F^2) = 0.086$	$(D/s)_{max} < 0.001$
$S = 1.07$	$Dr_{max} = 0.34$ e Å^{-3}
4185 reflections	$Dr_{min} = -0.20$ e Å^{-3}
247 parameters	Absolute structure: Flack x determined using 1784 quotients [(I+)-(I-)]/[(I+)+(I-)] (Parsons, Flack and Wagner, Acta Cryst. B69 (2013) 249-259).
1 restraint	Absolute structure parameter: 0.03 (9)

Fractional atomic coordinates and isotropic or equivalent isotropic displacement parameters (Å^2) for (EDG3071A)

	x	*y*	*z*	U_{iso}*/U_{eq}
O1	0.3945 (3)	0.80900 (10)	0.93127 (14)	0.0291 (3)
O2	0.9298 (2)	0.17117 (10)	0.64222 (15)	0.0278 (3)
H20H	1.032 (6)	0.183 (2)	0.599 (3)	0.042*
O3	1.1118 (2)	0.31271 (10)	0.56023 (13)	0.0248 (3)
C1	0.7371 (3)	0.67393 (13)	0.77916 (17)	0.0180 (4)
H1	0.610818	0.676764	0.713433	0.022*
C2	0.8701 (3)	0.75829 (13)	0.7655 (2)	0.0225 (4)
H2A	0.893828	0.769814	0.676747	0.027*
H2B	1.014428	0.755218	0.814990	0.027*
C3	0.7213 (4)	0.82887 (14)	0.8176 (2)	0.0265 (4)
H3A	0.809819	0.870480	0.871893	0.032*
H3B	0.641500	0.861999	0.748527	0.032*
C4	0.5599 (3)	0.77882 (13)	0.89306 (18)	0.0218 (4)
C5	0.6375 (3)	0.68440 (12)	0.90563 (18)	0.0178 (4)
C6	0.4598 (3)	0.61544 (13)	0.91701 (19)	0.0199 (4)
H6A	0.335980	0.626370	0.853081	0.024*
H6B	0.402833	0.619625	1.000584	0.024*
C7	0.5527 (3)	0.52281 (12)	0.89887 (18)	0.0177 (4)
H7A	0.431610	0.479666	0.899536	0.021*
H7B	0.661488	0.509109	0.970010	0.021*
C8	0.6638 (3)	0.51336 (12)	0.77556 (17)	0.0148 (3)
H8	0.547310	0.524621	0.706092	0.018*
C9	0.8436 (3)	0.58475 (12)	0.76497 (17)	0.0160 (4)
H9	0.960346	0.576584	0.835281	0.019*

C10	0.9498 (3)	0.57755 (13)	0.64104 (18)	0.0193 (4)
H10A	0.840749	0.595096	0.571896	0.023*
H10B	1.074692	0.619054	0.642286	0.023*
C11	1.0326 (3)	0.48577 (13)	0.61508 (18)	0.0198 (4)
H11A	1.156153	0.470879	0.677321	0.024*
H11B	1.087909	0.484166	0.530920	0.024*
C13	0.7550 (3)	0.41976 (12)	0.75375 (17)	0.0151 (4)
C14	0.5753 (3)	0.34902 (12)	0.75785 (18)	0.0175 (4)
H14A	0.509217	0.352772	0.838907	0.021*
H14B	0.457678	0.360565	0.690466	0.021*
C15	0.6653 (3)	0.25638 (13)	0.74195 (19)	0.0208 (4)
H15A	0.541547	0.214249	0.738465	0.025*
H15B	0.766399	0.241714	0.816155	0.025*
C16	0.7887 (3)	0.24558 (13)	0.6236 (2)	0.0206 (4)
C17	0.9326 (3)	0.32555 (13)	0.59927 (17)	0.0188 (4)
C18	0.8478 (3)	0.41782 (12)	0.62173 (17)	0.0171 (4)
C19	0.6682 (3)	0.43625 (13)	0.51424 (18)	0.0202 (4)
H19	0.518092	0.432984	0.531246	0.024*
C20	0.7150 (3)	0.45610 (13)	0.40118 (19)	0.0232 (4)
C21	0.7658 (5)	0.47669 (17)	0.2890 (2)	0.0369 (5)
H21A	0.773 (5)	0.434 (2)	0.217 (3)	0.055*
H21B	0.785 (6)	0.545 (2)	0.263 (3)	0.055*
C22	0.8106 (3)	0.68191 (14)	1.01937 (18)	0.0212 (4)
H22A	0.741029	0.698120	1.095185	0.032*
H22B	0.929108	0.723405	1.006161	0.032*
H22C	0.871616	0.622642	1.028934	0.032*
C23	0.9415 (3)	0.39729 (12)	0.85593 (17)	0.0179 (4)
H23A	1.031074	0.349431	0.826517	0.027*
H23B	0.877143	0.379169	0.932580	0.027*
H23C	1.034183	0.449049	0.873318	0.027*
C24	0.6266 (3)	0.22892 (14)	0.5083 (2)	0.0248 (4)
H24A	0.508884	0.272756	0.505330	0.037*
H24B	0.563065	0.170206	0.513875	0.037*
H24C	0.704600	0.233172	0.432297	0.037*

Atomic displacement parameters (Å^2) for (EDG3071A)

	U^{11}	U^{22}	U^{33}	U^{12}	U^{13}	U^{23}
O1	0.0303 (8)	0.0287 (8)	0.0286 (8)	0.0090 (6)	0.0042 (6)	-0.0050 (6)

O2	0.0250 (7)	0.0248 (8)	0.0338 (8)	0.0092 (6)	0.0031 (6)	-0.0034 (6)
O3	0.0150 (6)	0.0328 (8)	0.0269 (7)	0.0042 (5)	0.0038 (5)	-0.0076 (6)
C1	0.0142 (8)	0.0218 (9)	0.0178 (9)	0.0005 (7)	-0.0001 (6)	0.0018 (7)
C2	0.0232 (9)	0.0198 (9)	0.0248 (10)	-0.0010 (7)	0.0028 (7)	0.0021 (7)
C3	0.0290 (10)	0.0217 (9)	0.0284 (10)	0.0010 (8)	0.0002 (8)	0.0015 (8)
C4	0.0239 (9)	0.0227 (10)	0.0181 (9)	0.0032 (8)	-0.0032 (7)	-0.0029 (7)
C5	0.0171 (8)	0.0196 (9)	0.0166 (9)	0.0011 (7)	0.0006 (7)	-0.0022 (7)
C6	0.0157 (8)	0.0226 (9)	0.0221 (10)	0.0001 (7)	0.0052 (7)	-0.0042 (7)
C7	0.0155 (8)	0.0198 (9)	0.0187 (9)	-0.0016 (7)	0.0067 (6)	-0.0028 (7)
C8	0.0118 (7)	0.0193 (9)	0.0134 (8)	-0.0002 (6)	0.0013 (6)	-0.0009 (6)
C9	0.0121 (8)	0.0191 (9)	0.0169 (9)	-0.0011 (6)	0.0019 (6)	0.0010 (7)
C10	0.0170 (8)	0.0222 (9)	0.0194 (9)	-0.0024 (7)	0.0052 (7)	0.0021 (7)
C11	0.0144 (8)	0.0266 (10)	0.0193 (9)	0.0006 (7)	0.0062 (6)	-0.0001 (7)
C13	0.0107 (7)	0.0201 (9)	0.0147 (8)	0.0009 (6)	0.0021 (6)	-0.0020 (6)
C14	0.0145 (8)	0.0200 (9)	0.0183 (9)	-0.0013 (6)	0.0023 (6)	-0.0025 (7)
C15	0.0199 (8)	0.0199 (9)	0.0229 (10)	-0.0007 (7)	0.0027 (7)	-0.0032 (7)
C16	0.0168 (8)	0.0212 (9)	0.0235 (10)	0.0045 (7)	-0.0006 (7)	-0.0043 (7)
C17	0.0148 (8)	0.0277 (10)	0.0134 (8)	0.0045 (7)	-0.0008 (6)	-0.0037 (7)
C18	0.0125 (8)	0.0225 (9)	0.0163 (9)	0.0017 (7)	0.0022 (6)	-0.0013 (7)
C19	0.0170 (8)	0.0256 (10)	0.0181 (9)	0.0033 (7)	0.0013 (6)	-0.0017 (7)
C20	0.0249 (9)	0.0220 (10)	0.0220 (10)	-0.0018 (7)	-0.0022 (7)	-0.0019 (7)

C21	0.0500 (14)	0.0396 (13)	0.0206 (10)	-0.0175 (11)	0.0008 (9)	0.0030 (9)
C22	0.0225 (9)	0.0221 (9)	0.0185 (9)	-0.0001 (7)	-0.0017 (7)	-0.0022 (7)
C23	0.0144 (8)	0.0211 (9)	0.0177 (9)	0.0014 (6)	-0.0011 (6)	0.0004 (7)
C24	0.0202 (8)	0.0288 (11)	0.0250 (10)	0.0016 (7)	0.0000 (7)	-0.0103 (8)

Geometric parameters (Å, º) for (EDG3071A)

O1—C4	1.211 (3)	C11—C18	1.538 (3)
O2—C16	1.429 (2)	C11—H11A	0.9900
O2—H20H	0.83 (3)	C11—H11B	0.9900
O3—C17	1.219 (2)	C13—C14	1.542 (2)
C1—C9	1.521 (3)	C13—C23	1.549 (2)
C1—C2	1.535 (3)	C13—C18	1.568 (2)
C1—C5	1.540 (3)	C14—C15	1.531 (3)
C1—H1	1.0000	C14—H14A	0.9900
C2—C3	1.543 (3)	C14—H14B	0.9900
C2—H2A	0.9900	C15—C16	1.538 (3)
C2—H2B	0.9900	C15—H15A	0.9900
C3—C4	1.531 (3)	C15—H15B	0.9900
C3—H3A	0.9900	C16—C24	1.537 (3)
C3—H3B	0.9900	C16—C17	1.538 (3)
C4—C5	1.519 (3)	C17—C18	1.526 (3)
C5—C6	1.523 (3)	C18—C19	1.547 (2)
C5—C22	1.545 (2)	C19—C20	1.304 (3)
C6—C7	1.541 (3)	C19—H19	0.9500
C6—H6A	0.9900	C20—C21	1.305 (3)
C6—H6B	0.9900	C21—H21A	1.02 (4)
C7—C8	1.543 (2)	C21—H21B	1.08 (4)
C7—H7A	0.9900	C22—H22A	0.9800
C7—H7B	0.9900	C22—H22B	0.9800
C8—C9	1.557 (2)	C22—H22C	0.9800
C8—C13	1.558 (2)	C23—H23A	0.9800
C8—H8	1.0000	C23—H23B	0.9800
C9—C10	1.530 (2)	C23—H23C	0.9800
C9—H9	1.0000	C24—H24A	0.9800
C10—C11	1.522 (3)	C24—H24B	0.9800

C10—H10A	0.9900	C24—H24C	0.9800
C10—H10B	0.9900		
C16—O2—H20H	103 (2)	C10—C11—H11B	109.5
C9—C1—C2	120.46 (14)	C18—C11—H11B	109.5
C9—C1—C5	112.60 (15)	H11A—C11—H11B	108.1
C2—C1—C5	104.37 (15)	C14—C13—C23	107.82 (15)
C9—C1—H1	106.2	C14—C13—C8	111.95 (13)
C2—C1—H1	106.2	C23—C13—C8	110.30 (14)
C5—C1—H1	106.2	C14—C13—C18	108.91 (14)
C1—C2—C3	102.65 (15)	C23—C13—C18	109.56 (13)
C1—C2—H2A	111.2	C8—C13—C18	108.26 (14)
C3—C2—H2A	111.2	C15—C14—C13	112.36 (14)
C1—C2—H2B	111.2	C15—C14—H14A	109.1
C3—C2—H2B	111.2	C13—C14—H14A	109.1
H2A—C2—H2B	109.2	C15—C14—H14B	109.1
C4—C3—C2	105.51 (16)	C13—C14—H14B	109.1
C4—C3—H3A	110.6	H14A—C14—H14B	107.9
C2—C3—H3A	110.6	C14—C15—C16	113.33 (16)
C4—C3—H3B	110.6	C14—C15—H15A	108.9
C2—C3—H3B	110.6	C16—C15—H15A	108.9
H3A—C3—H3B	108.8	C14—C15—H15B	108.9
O1—C4—C5	126.36 (19)	C16—C15—H15B	108.9
O1—C4—C3	125.5 (2)	H15A—C15—H15B	107.7
C5—C4—C3	108.09 (16)	O2—C16—C24	108.67 (16)
C4—C5—C6	116.35 (16)	O2—C16—C17	107.98 (15)
C4—C5—C1	99.41 (15)	C24—C16—C17	109.42 (17)
C6—C5—C1	109.59 (15)	O2—C16—C15	107.50 (16)
C4—C5—C22	106.29 (15)	C24—C16—C15	110.78 (15)
C6—C5—C22	111.32 (16)	C17—C16—C15	112.38 (16)
C1—C5—C22	113.46 (15)	O3—C17—C18	121.88 (18)
C5—C6—C7	110.58 (15)	O3—C17—C16	118.15 (18)
C5—C6—H6A	109.5	C18—C17—C16	119.97 (15)
C7—C6—H6A	109.5	C17—C18—C11	110.81 (14)
C5—C6—H6B	109.5	C17—C18—C19	106.15 (14)
C7—C6—H6B	109.5	C11—C18—C19	108.57 (15)
H6A—C6—H6B	108.1	C17—C18—C13	108.22 (15)
C6—C7—C8	112.76 (16)	C11—C18—C13	110.54 (14)
C6—C7—H7A	109.0	C19—C18—C13	112.47 (13)

C8—C7—H7A	109.0	C20—C19—C18	122.54 (17)
C6—C7—H7B	109.0	C20—C19—H19	118.7
C8—C7—H7B	109.0	C18—C19—H19	118.7
H7A—C7—H7B	107.8	C19—C20—C21	178.8 (2)
C7—C8—C9	111.12 (14)	C20—C21—H21A	125 (2)
C7—C8—C13	113.75 (14)	C20—C21—H21B	120 (2)
C9—C8—C13	111.59 (13)	H21A—C21—H21B	115 (3)
C7—C8—H8	106.6	C5—C22—H22A	109.5
C9—C8—H8	106.6	C5—C22—H22B	109.5
C13—C8—H8	106.6	H22A—C22—H22B	109.5
C1—C9—C10	111.51 (15)	C5—C22—H22C	109.5
C1—C9—C8	108.02 (14)	H22A—C22—H22C	109.5
C10—C9—C8	111.44 (14)	H22B—C22—H22C	109.5
C1—C9—H9	108.6	C13—C23—H23A	109.5
C10—C9—H9	108.6	C13—C23—H23B	109.5
C8—C9—H9	108.6	H23A—C23—H23B	109.5
C11—C10—C9	113.33 (16)	C13—C23—H23C	109.5
C11—C10—H10A	108.9	H23A—C23—H23C	109.5
C9—C10—H10A	108.9	H23B—C23—H23C	109.5
C11—C10—H10B	108.9	C16—C24—H24A	109.5
C9—C10—H10B	108.9	C16—C24—H24B	109.5
H10A—C10—H10B	107.7	H24A—C24—H24B	109.5
C10—C11—C18	110.85 (14)	C16—C24—H24C	109.5
C10—C11—H11A	109.5	H24A—C24—H24C	109.5
C18—C11—H11A	109.5	H24B—C24—H24C	109.5
C9—C1—C2—C3	-166.79 (16)	C9—C8—C13—C23	62.64 (18)
C5—C1—C2—C3	-39.12 (17)	C7—C8—C13—C18	176.08 (14)
C1—C2—C3—C4	17.56 (19)	C9—C8—C13—C18	-57.22 (17)
C2—C3—C4—O1	-167.01 (19)	C23—C13—C14—C15	-56.12 (19)
C2—C3—C4—C5	10.2 (2)	C8—C13—C14—C15	-177.62 (14)
O1—C4—C5—C6	26.4 (3)	C18—C13—C14—C15	62.69 (18)
C3—C4—C5—C6	-150.79 (17)	C13—C14—C15—C16	-54.1 (2)
O1—C4—C5—C1	143.91 (19)	C14—C15—C16—O2	158.81 (15)

C3—C4—C5—C1	-33.31 (18)	C14—C15—C16—C24	-82.6 (2)
O1—C4—C5—C22	-98.1 (2)	C14—C15—C16—C17	40.1 (2)
C3—C4—C5—C22	84.64 (18)	O2—C16—C17—O3	22.9 (2)
C9—C1—C5—C4	176.89 (14)	C24—C16—C17—O3	-95.18 (19)
C2—C1—C5—C4	44.55 (16)	C15—C16—C17—O3	141.32 (17)
C9—C1—C5—C6	-60.66 (18)	O2—C16—C17—C18	-158.22 (16)
C2—C1—C5—C6	166.99 (15)	C24—C16—C17—C18	83.66 (19)
C9—C1—C5—C22	64.4 (2)	C15—C16—C17—C18	-39.8 (2)
C2—C1—C5—C22	-67.90 (18)	O3—C17—C18—C11	-11.7 (2)
C4—C5—C6—C7	167.11 (16)	C16—C17—C18—C11	169.55 (16)
C1—C5—C6—C7	55.41 (19)	O3—C17—C18—C19	106.05 (19)
C22—C5—C6—C7	-70.9 (2)	C16—C17—C18—C19	-72.76 (19)
C5—C6—C7—C8	-54.0 (2)	O3—C17—C18—C13	-133.00 (17)
C6—C7—C8—C9	54.22 (19)	C16—C17—C18—C13	48.2 (2)
C6—C7—C8—C13	-178.84 (14)	C10—C11—C18—C17	-178.43 (15)
C2—C1—C9—C10	-53.7 (2)	C10—C11—C18—C19	65.36 (19)
C5—C1—C9—C10	-177.56 (14)	C10—C11—C18—C13	-58.45 (19)
C2—C1—C9—C8	-176.49 (16)	C14—C13—C18—C17	-56.84 (17)
C5—C1—C9—C8	59.67 (18)	C23—C13—C18—C17	60.88 (18)
C7—C8—C9—C1	-55.45 (18)	C8—C13—C18—C17	-178.79 (13)
C13—C8—C9—C1	176.44 (13)	C14—C13—C18—C11	-178.36 (15)
C7—C8—C9—C10	-178.26 (15)	C23—C13—C18—	-60.64 (19)

		C11	
C13—C8—C9—C10	53.63 (18)	C8—C13—C18—C11	59.69 (17)
C1—C9—C10—C11	-172.60 (14)	C14—C13—C18—C19	60.10 (18)
C8—C9—C10—C11	-51.8 (2)	C23—C13—C18—C19	177.82 (16)
C9—C10—C11—C18	54.5 (2)	C8—C13—C18—C19	-61.85 (18)
C7—C8—C13—C14	56.01 (18)	C17—C18—C19—C20	-76.7 (2)
C9—C8—C13—C14	-177.29 (15)	C11—C18—C19—C20	42.4 (2)
C7—C8—C13—C23	-64.05 (18)	C13—C18—C19—C20	165.09 (19)

Hydrogen-bond geometry (Å, º) for (EDG3071A)

D—H···*A*	*D*—H	H···*A*	*D*···*A*	*D*—H···*A*
O2—H20*H*···O3	0.83 (3)	2.09 (3)	2.614 (2)	121 (3)

Compound (±)-11

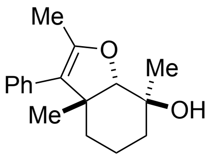

(±)-11

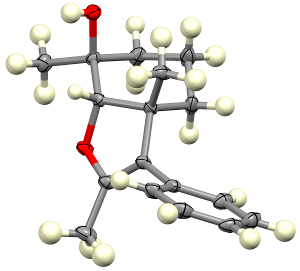

Sample Name: CCDC 2426294 / Armend15 (EAG3179P)
Crystal Growth: Slow evaporation using a mixture of hexanes and dichloromethane
Ellipsoid Contour: Set at a 50% probability level

Crystal data

$C_{17}H_{22}O_2$	$F(000) = 2240$
$M_r = 258.34$	$D_x = 1.104$ Mg m^{-3}
Monoclinic, *I*2/*a*	Cu *K*a radiation, l = 1.54184 Å
a = 29.7947 (9) Å	Cell parameters from 9819 reflections
b = 7.1252 (2) Å	q = 3.0–74.3°
c = 30.0312 (6) Å	m = 0.55 mm^{-1}
b = 102.794 (2)°	*T* = 100 K
V = 6217.1 (3) Å^3	Plate, colourless
Z = 16	0.33 × 0.20 × 0.04 mm

Data collection

Bruker D8 Venture DUO with Photon III C14 diffractometer	5746 reflections with *I* > 2s(*I*)
Radiation source: ImS 3.0 microfocus	$R_{int} = 0.061$
f and w scans	$q_{max} = 74.5°$, $q_{min} = 3.0°$
Absorption correction: multi-scan *SADABS* (Krause *et al.*, 2015)	*h* = -37 - 37
$T_{min} = 0.788$, $T_{max} = 0.978$	*k* = -8 - 8
76137 measured reflections	*l* = -36 - 37
6318 independent reflections	

Refinement

Refinement on F^2	0 restraints
Least-squares matrix: full	Hydrogen site location: inferred from neighbouring sites
$R[F^2 > 2s(F^2)] = 0.052$	H-atom parameters constrained

$wR(F^2) = 0.128$	$w = 1/[s^2(F_o^2) + (0.0418P)^2 + 9.5135P]$ where $P = (F_o^2 + 2F_c^2)/3$
$S = 1.11$	$(D/s)_{max} = 0.001$
6318 reflections	$Dr_{max} = 0.29$ e Å^{-3}
351 parameters	$Dr_{min} = -0.27$ e Å^{-3}

Fractional atomic coordinates and isotropic or equivalent isotropic displacement parameters (Å^2) for (EAG3179P)

	x	*y*	*z*	U_{iso}*/U_{eq}
O1	0.60192 (4)	0.27712 (16)	0.54906 (4)	0.0250 (3)
O2	0.70025 (4)	0.56375 (17)	0.53494 (4)	0.0261 (3)
H2OH	0.696096	0.565035	0.506353	0.039*
C1	0.62776 (5)	0.4476 (2)	0.54459 (6)	0.0223 (3)
H1	0.615701	0.500110	0.513348	0.027*
C2	0.67858 (5)	0.3986 (2)	0.54906 (6)	0.0247 (3)
C3	0.70246 (6)	0.3622 (3)	0.59838 (7)	0.0348 (4)
H3A	0.689930	0.246128	0.609094	0.042*
H3B	0.735759	0.342888	0.600421	0.042*
C4	0.69586 (6)	0.5245 (3)	0.62898 (6)	0.0358 (4)
H4A	0.708480	0.640698	0.618438	0.043*
H4B	0.712872	0.498999	0.660636	0.043*
C5	0.64473 (6)	0.5522 (3)	0.62820 (6)	0.0316 (4)
H5A	0.641314	0.660893	0.647712	0.038*
H5B	0.633138	0.439747	0.641453	0.038*
C6	0.61495 (5)	0.5855 (2)	0.57977 (5)	0.0215 (3)
C7	0.56557 (5)	0.5159 (2)	0.57708 (6)	0.0217 (3)
C8	0.56237 (5)	0.3399 (2)	0.56178 (6)	0.0228 (3)
C9	0.68374 (6)	0.2354 (3)	0.51799 (7)	0.0335 (4)
H9A	0.669597	0.122902	0.527752	0.050*
H9B	0.716492	0.211819	0.519720	0.050*
H9C	0.668472	0.266097	0.486465	0.050*
C10	0.61803 (6)	0.7891 (2)	0.56495 (7)	0.0290 (4)
H10A	0.606271	0.871979	0.585804	0.043*
H10B	0.599587	0.805418	0.533853	0.043*
H10C	0.650194	0.820723	0.565690	0.043*
C11	0.52564 (6)	0.1956 (2)	0.55837 (6)	0.0266 (4)
H11A	0.499978	0.247673	0.569931	0.040*
H11B	0.538052	0.085540	0.576568	0.040*
H11C	0.514707	0.158476	0.526383	0.040*

C12	0.52922 (5)	0.6275 (2)	0.59122 (6)	0.0214 (3)
C13	0.48699 (6)	0.6582 (2)	0.56072 (6)	0.0254 (3)
H13	0.481052	0.600647	0.531470	0.030*
C14	0.45358 (6)	0.7721 (3)	0.57271 (7)	0.0311 (4)
H14	0.425345	0.794119	0.551425	0.037*
C15	0.46143 (6)	0.8531 (3)	0.61546 (7)	0.0319 (4)
H15	0.438524	0.930117	0.623694	0.038*
C16	0.50272 (6)	0.8221 (2)	0.64637 (6)	0.0297 (4)
H16	0.507969	0.876580	0.675947	0.036*
C17	0.53644 (6)	0.7116 (2)	0.63420 (6)	0.0253 (3)
H17	0.564869	0.693005	0.655437	0.030*
O3	0.83593 (4)	0.85541 (17)	0.66435 (4)	0.0313 (3)
O4	0.79594 (4)	0.60144 (16)	0.55683 (4)	0.0253 (3)
H40H	0.767454	0.594736	0.555053	0.038*
C18	0.82565 (6)	0.6950 (2)	0.63397 (6)	0.0242 (3)
H18	0.797691	0.631699	0.640116	0.029*
C19	0.81436 (5)	0.7613 (2)	0.58416 (6)	0.0240 (3)
C20	0.85788 (6)	0.8183 (3)	0.56848 (6)	0.0292 (4)
H20A	0.849953	0.844495	0.535281	0.035*
H20B	0.870485	0.934855	0.584399	0.035*
C21	0.89425 (6)	0.6652 (3)	0.57826 (6)	0.0310 (4)
H21A	0.921634	0.705373	0.567127	0.037*
H21B	0.882018	0.549550	0.561629	0.037*
C22	0.90827 (6)	0.6234 (3)	0.62922 (6)	0.0286 (4)
H22A	0.922514	0.737003	0.645409	0.034*
H22B	0.931699	0.522549	0.634390	0.034*
C23	0.86700 (6)	0.5621 (2)	0.64974 (6)	0.0243 (3)
C24	0.87770 (6)	0.6106 (2)	0.70076 (6)	0.0294 (4)
C25	0.86132 (7)	0.7827 (3)	0.70506 (6)	0.0339 (4)
C26	0.77926 (6)	0.9193 (2)	0.57686 (6)	0.0300 (4)
H26A	0.792371	1.028885	0.594837	0.045*
H26B	0.751561	0.878078	0.586613	0.045*
H26C	0.771213	0.952832	0.544416	0.045*
C27	0.85408 (6)	0.3572 (2)	0.63895 (6)	0.0275 (4)
H27A	0.879812	0.276299	0.653338	0.041*
H27B	0.847227	0.338300	0.605813	0.041*
H27C	0.826897	0.325547	0.650780	0.041*
C28	0.86783 (9)	0.9167 (3)	0.74393 (8)	0.0513 (6)
H28A	0.892354	0.870997	0.768918	0.077*

H28B	0.839109	0.927369	0.754537	0.077*
H28C	0.876325	1.040102	0.733940	0.077*
C29	0.90378 (7)	0.4911 (3)	0.73767 (6)	0.0321 (4)
C30	0.88923 (7)	0.4733 (3)	0.77892 (7)	0.0375 (4)
H30	0.862983	0.540919	0.783061	0.045*
C31	0.91268 (8)	0.3583 (3)	0.81367 (7)	0.0452 (5)
H31	0.902465	0.349217	0.841403	0.054*
C32	0.95051 (9)	0.2575 (3)	0.80846 (7)	0.0497 (6)
H32	0.966233	0.178306	0.832321	0.060*
C33	0.96555 (9)	0.2723 (3)	0.76799 (8)	0.0474 (6)
H33	0.991744	0.203490	0.764153	0.057*
C34	0.94223 (7)	0.3880 (3)	0.73302 (7)	0.0373 (4)
H34	0.952757	0.396854	0.705457	0.045*

Atomic displacement parameters (Å^2) for (EAG3179P)

	U^{11}	U^{22}	U^{33}	U^{12}	U^{13}	U^{23}
O1	0.0201 (5)	0.0180 (5)	0.0391 (7)	-0.0028 (4)	0.0113 (5)	-0.0035 (5)
O2	0.0236 (6)	0.0258 (6)	0.0308 (6)	-0.0036 (5)	0.0099 (5)	0.0004 (5)
C1	0.0210 (7)	0.0196 (8)	0.0273 (8)	-0.0023 (6)	0.0072 (6)	-0.0004 (6)
C2	0.0186 (7)	0.0210 (8)	0.0359 (9)	-0.0016 (6)	0.0090 (7)	0.0009 (7)
C3	0.0236 (8)	0.0379 (10)	0.0421 (11)	0.0029 (7)	0.0055 (8)	0.0115 (8)
C4	0.0246 (9)	0.0531 (12)	0.0274 (9)	-0.0060 (8)	0.0013 (7)	0.0058 (8)
C5	0.0264 (8)	0.0425 (11)	0.0256 (9)	-0.0081 (8)	0.0054 (7)	-0.0001 (8)
C6	0.0199 (7)	0.0207 (8)	0.0246 (8)	-0.0026 (6)	0.0066 (6)	-0.0021 (6)
C7	0.0188 (7)	0.0217 (8)	0.0251 (8)	-0.0014 (6)	0.0056 (6)	-0.0009 (6)
C8	0.0175 (7)	0.0229 (8)	0.0285 (8)	-0.0005 (6)	0.0062 (6)	0.0004 (6)
C9	0.0262 (9)	0.0257 (9)	0.0522 (12)	0.0024 (7)	0.0162 (8)	-0.0028 (8)
C10	0.0299 (9)	0.0224 (8)	0.0378 (10)	-0.0035 (7)	0.0145 (7)	-0.0032 (7)
C11	0.0218 (8)	0.0214 (8)	0.0371 (9)	-0.0039 (6)	0.0077 (7)	-0.0006

						(7)
C12	0.0200 (7)	0.0177 (7)	0.0279 (8)	-0.0028 (6)	0.0081 (6)	-0.0003 (6)
C13	0.0218 (8)	0.0243 (8)	0.0304 (9)	-0.0024 (6)	0.0063 (7)	-0.0034 (7)
C14	0.0190 (8)	0.0286 (9)	0.0454 (11)	-0.0002 (7)	0.0062 (7)	-0.0021 (8)
C15	0.0247 (8)	0.0239 (8)	0.0511 (11)	-0.0012 (7)	0.0164 (8)	-0.0068 (8)
C16	0.0326 (9)	0.0246 (9)	0.0353 (9)	-0.0065 (7)	0.0150 (7)	-0.0075 (7)
C17	0.0238 (8)	0.0244 (8)	0.0278 (8)	-0.0035 (6)	0.0059 (6)	-0.0026 (7)
O3	0.0371 (7)	0.0210 (6)	0.0311 (7)	-0.0005 (5)	-0.0023 (5)	-0.0032 (5)
O4	0.0184 (5)	0.0258 (6)	0.0297 (6)	-0.0022 (4)	0.0013 (5)	-0.0015 (5)
C18	0.0240 (8)	0.0186 (8)	0.0286 (8)	-0.0038 (6)	0.0028 (6)	-0.0005 (6)
C19	0.0193 (7)	0.0215 (8)	0.0289 (9)	-0.0014 (6)	0.0006 (6)	0.0024 (6)
C20	0.0226 (8)	0.0301 (9)	0.0332 (9)	-0.0048 (7)	0.0023 (7)	0.0089 (7)
C21	0.0195 (8)	0.0382 (10)	0.0354 (10)	-0.0010 (7)	0.0065 (7)	0.0090 (8)
C22	0.0201 (8)	0.0273 (9)	0.0356 (10)	-0.0021 (6)	0.0003 (7)	0.0056 (7)
C23	0.0237 (8)	0.0192 (8)	0.0272 (8)	-0.0030 (6)	-0.0006 (6)	0.0034 (6)
C24	0.0312 (9)	0.0244 (9)	0.0291 (9)	-0.0056 (7)	-0.0014 (7)	0.0011 (7)
C25	0.0392 (10)	0.0267 (9)	0.0311 (9)	-0.0033 (8)	-0.0023 (8)	-0.0008 (7)
C26	0.0255 (8)	0.0241 (8)	0.0370 (10)	0.0012 (7)	-0.0004 (7)	0.0022 (7)
C27	0.0287 (8)	0.0214 (8)	0.0307 (9)	-0.0031 (7)	0.0026 (7)	0.0007 (7)
C28	0.0729 (16)	0.0336 (11)	0.0388 (12)	0.0006 (11)	-0.0063 (11)	-0.0090 (9)
C29	0.0375 (10)	0.0226 (8)	0.0300 (9)	-0.0090 (7)	-0.0057 (7)	0.0012 (7)
C30	0.0439	0.0315	0.0324	-0.0135 (8)	-0.0017 (8)	0.0030

	(11)	(10)	(10)			(8)
C31	0.0629 (14)	0.0345 (11)	0.0315 (10)	-0.0218 (10)	-0.0037 (9)	0.0063 (8)
C32	0.0745 (16)	0.0248 (10)	0.0359 (11)	-0.0086 (10)	-0.0174 (10)	0.0068 (8)
C33	0.0597 (14)	0.0283 (10)	0.0431 (12)	0.0051 (9)	-0.0128 (10)	0.0001 (9)
C34	0.0458 (11)	0.0277 (9)	0.0318 (10)	0.0009 (8)	-0.0053 (8)	0.0008 (7)

Geometric parameters (Å, º) for (EAG3179P)

O1—C8	1.3905 (19)	O3—C25	1.387 (2)
O1—C1	1.4601 (19)	O3—C18	1.452 (2)
O2—C2	1.4497 (19)	O4—C19	1.440 (2)
O2—H2OH	0.8400	O4—H4OH	0.8400
C1—C2	1.530 (2)	C18—C19	1.533 (2)
C1—C6	1.551 (2)	C18—C23	1.543 (2)
C1—H1	1.0000	C18—H18	1.0000
C2—C3	1.517 (3)	C19—C26	1.519 (2)
C2—C9	1.520 (2)	C19—C20	1.529 (2)
C3—C4	1.517 (3)	C20—C21	1.520 (2)
C3—H3A	0.9900	C20—H20A	0.9900
C3—H3B	0.9900	C20—H20B	0.9900
C4—C5	1.531 (2)	C21—C22	1.524 (3)
C4—H4A	0.9900	C21—H21A	0.9900
C4—H4B	0.9900	C21—H21B	0.9900
C5—C6	1.545 (2)	C22—C23	1.555 (2)
C5—H5A	0.9900	C22—H22A	0.9900
C5—H5B	0.9900	C22—H22B	0.9900
C6—C10	1.526 (2)	C23—C27	1.526 (2)
C6—C7	1.538 (2)	C23—C24	1.534 (2)
C7—C8	1.332 (2)	C24—C25	1.337 (3)
C7—C12	1.480 (2)	C24—C29	1.475 (2)
C8—C11	1.489 (2)	C25—C28	1.487 (3)
C9—H9A	0.9800	C26—H26A	0.9800
C9—H9B	0.9800	C26—H26B	0.9800
C9—H9C	0.9800	C26—H26C	0.9800
C10—H10A	0.9800	C27—H27A	0.9800
C10—H10B	0.9800	C27—H27B	0.9800

C10—H10C	0.9800	C27—H27C	0.9800
C11—H11A	0.9800	C28—H28A	0.9800
C11—H11B	0.9800	C28—H28B	0.9800
C11—H11C	0.9800	C28—H28C	0.9800
C12—C17	1.396 (2)	C29—C34	1.393 (3)
C12—C13	1.400 (2)	C29—C30	1.406 (3)
C13—C14	1.392 (2)	C30—C31	1.387 (3)
C13—H13	0.9500	C30—H30	0.9500
C14—C15	1.380 (3)	C31—C32	1.375 (4)
C14—H14	0.9500	C31—H31	0.9500
C15—C16	1.385 (3)	C32—C33	1.389 (4)
C15—H15	0.9500	C32—H32	0.9500
C16—C17	1.387 (2)	C33—C34	1.394 (3)
C16—H16	0.9500	C33—H33	0.9500
C17—H17	0.9500	C34—H34	0.9500
C8—O1—C1	104.71 (12)	C25—O3—C18	104.95 (13)
C2—O2—H20H	109.5	C19—O4—H40H	109.5
O1—C1—C2	109.51 (13)	O3—C18—C19	109.93 (13)
O1—C1—C6	104.47 (12)	O3—C18—C23	104.32 (13)
C2—C1—C6	118.19 (14)	C19—C18—C23	118.45 (14)
O1—C1—H1	108.1	O3—C18—H18	107.9
C2—C1—H1	108.1	C19—C18—H18	107.9
C6—C1—H1	108.1	C23—C18—H18	107.9
O2—C2—C3	106.34 (14)	O4—C19—C26	109.92 (13)
O2—C2—C9	109.23 (14)	O4—C19—C20	106.05 (14)
C3—C2—C9	112.27 (15)	C26—C19—C20	111.25 (14)
O2—C2—C1	106.59 (13)	O4—C19—C18	106.62 (13)
C3—C2—C1	111.45 (14)	C26—C19—C18	111.37 (14)
C9—C2—C1	110.68 (14)	C20—C19—C18	111.37 (13)
C4—C3—C2	111.44 (15)	C21—C20—C19	111.53 (14)
C4—C3—H3A	109.3	C21—C20—H20A	109.3
C2—C3—H3A	109.3	C19—C20—H20A	109.3
C4—C3—H3B	109.3	C21—C20—H20B	109.3
C2—C3—H3B	109.3	C19—C20—H20B	109.3
H3A—C3—H3B	108.0	H20A—C20—H20B	108.0
C3—C4—C5	110.55 (16)	C20—C21—C22	111.05 (16)
C3—C4—H4A	109.5	C20—C21—H21A	109.4
C5—C4—H4A	109.5	C22—C21—H21A	109.4

C3—C4—H4B	109.5	C20—C21—H21B	109.4
C5—C4—H4B	109.5	C22—C21—H21B	109.4
H4A—C4—H4B	108.1	H21A—C21—H21B	108.0
C4—C5—C6	113.34 (14)	C21—C22—C23	112.83 (13)
C4—C5—H5A	108.9	C21—C22—H22A	109.0
C6—C5—H5A	108.9	C23—C22—H22A	109.0
C4—C5—H5B	108.9	C21—C22—H22B	109.0
C6—C5—H5B	108.9	C23—C22—H22B	109.0
H5A—C5—H5B	107.7	H22A—C22—H22B	107.8
C10—C6—C7	114.19 (14)	C27—C23—C24	114.49 (14)
C10—C6—C5	111.19 (14)	C27—C23—C18	112.05 (13)
C7—C6—C5	110.08 (13)	C24—C23—C18	98.26 (14)
C10—C6—C1	111.34 (13)	C27—C23—C22	111.66 (15)
C7—C6—C1	97.85 (12)	C24—C23—C22	108.95 (14)
C5—C6—C1	111.55 (14)	C18—C23—C22	110.69 (13)
C8—C7—C12	127.15 (15)	C25—C24—C29	126.87 (17)
C8—C7—C6	108.45 (14)	C25—C24—C23	107.67 (15)
C12—C7—C6	124.35 (14)	C29—C24—C23	125.38 (16)
C7—C8—O1	113.41 (14)	C24—C25—O3	113.38 (16)
C7—C8—C11	132.04 (15)	C24—C25—C28	132.67 (18)
O1—C8—C11	114.44 (14)	O3—C25—C28	113.77 (17)
C2—C9—H9A	109.5	C19—C26—H26A	109.5
C2—C9—H9B	109.5	C19—C26—H26B	109.5
H9A—C9—H9B	109.5	H26A—C26—H26B	109.5
C2—C9—H9C	109.5	C19—C26—H26C	109.5
H9A—C9—H9C	109.5	H26A—C26—H26C	109.5
H9B—C9—H9C	109.5	H26B—C26—H26C	109.5
C6—C10—H10A	109.5	C23—C27—H27A	109.5
C6—C10—H10B	109.5	C23—C27—H27B	109.5
H10A—C10—H10B	109.5	H27A—C27—H27B	109.5
C6—C10—H10C	109.5	C23—C27—H27C	109.5
H10A—C10—H10C	109.5	H27A—C27—H27C	109.5
H10B—C10—H10C	109.5	H27B—C27—H27C	109.5
C8—C11—H11A	109.5	C25—C28—H28A	109.5
C8—C11—H11B	109.5	C25—C28—H28B	109.5
H11A—C11—H11B	109.5	H28A—C28—H28B	109.5
C8—C11—H11C	109.5	C25—C28—H28C	109.5
H11A—C11—H11C	109.5	H28A—C28—H28C	109.5
H11B—C11—H11C	109.5	H28B—C28—H28C	109.5

C17—C12—C13	118.02 (15)	C34—C29—C30	117.49 (18)
C17—C12—C7	121.57 (15)	C34—C29—C24	122.47 (18)
C13—C12—C7	120.35 (15)	C30—C29—C24	120.01 (18)
C14—C13—C12	120.84 (16)	C31—C30—C29	120.9 (2)
C14—C13—H13	119.6	C31—C30—H30	119.5
C12—C13—H13	119.6	C29—C30—H30	119.5
C15—C14—C13	120.07 (17)	C32—C31—C30	120.8 (2)
C15—C14—H14	120.0	C32—C31—H31	119.6
C13—C14—H14	120.0	C30—C31—H31	119.6
C14—C15—C16	119.96 (16)	C31—C32—C33	119.43 (19)
C14—C15—H15	120.0	C31—C32—H32	120.3
C16—C15—H15	120.0	C33—C32—H32	120.3
C15—C16—C17	120.10 (17)	C32—C33—C34	120.0 (2)
C15—C16—H16	119.9	C32—C33—H33	120.0
C17—C16—H16	119.9	C34—C33—H33	120.0
C16—C17—C12	120.99 (16)	C29—C34—C33	121.3 (2)
C16—C17—H17	119.5	C29—C34—H34	119.3
C12—C17—H17	119.5	C33—C34—H34	119.3
C8—O1—C1—C2	158.19 (13)	C25—O3—C18—C19	-158.59 (14)
C8—O1—C1—C6	30.67 (15)	C25—O3—C18—C23	-30.60 (17)
O1—C1—C2—O2	168.33 (12)	O3—C18—C19—O4	-168.94 (12)
C6—C1—C2—O2	-72.28 (18)	C23—C18—C19—O4	71.35 (17)
O1—C1—C2—C3	-76.05 (17)	O3—C18—C19—C26	-49.03 (18)
C6—C1—C2—C3	43.3 (2)	C23—C18—C19—C26	-168.74 (14)
O1—C1—C2—C9	49.65 (18)	O3—C18—C19—C20	75.81 (17)
C6—C1—C2—C9	169.04 (15)	C23—C18—C19—C20	-43.9 (2)
O2—C2—C3—C4	62.07 (17)	O4—C19—C20—C21	-63.86 (18)
C9—C2—C3—C4	-178.53 (14)	C26—C19—C20—C21	176.64 (15)
C1—C2—C3—C4	-53.71 (19)	C18—C19—C20—C21	51.7 (2)
C2—C3—C4—C5	61.5 (2)	C19—C20—C21—C22	-60.3 (2)
C3—C4—C5—C6	-57.0 (2)	C20—C21—C22—C23	58.2 (2)
C4—C5—C6—C10	-80.6 (2)	O3—C18—C23—C27	153.19 (14)
C4—C5—C6—C7	151.85 (16)	C19—C18—C23—	-84.24 (18)

		C27	
C4—C5—C6—C1	44.4 (2)	O3—C18—C23—C24	32.48 (15)
O1—C1—C6—C10	-151.63 (13)	C19—C18—C23—C24	155.06 (14)
C2—C1—C6—C10	86.38 (18)	O3—C18—C23—C22	-81.45 (16)
O1—C1—C6—C7	-31.77 (15)	C19—C18—C23—C22	41.12 (19)
C2—C1—C6—C7	-153.75 (14)	C21—C22—C23—C27	78.56 (18)
O1—C1—C6—C5	83.51 (15)	C21—C22—C23—C24	-154.00 (15)
C2—C1—C6—C5	-38.47 (19)	C21—C22—C23—C18	-47.0 (2)
C10—C6—C7—C8	140.67 (15)	C27—C23—C24—C25	-142.94 (16)
C5—C6—C7—C8	-93.45 (17)	C18—C23—C24—C25	-24.07 (18)
C1—C6—C7—C8	22.98 (17)	C22—C23—C24—C25	91.24 (18)
C10—C6—C7—C12	-41.6 (2)	C27—C23—C24—C29	40.2 (2)
C5—C6—C7—C12	84.29 (19)	C18—C23—C24—C29	159.10 (17)
C1—C6—C7—C12	-159.27 (15)	C22—C23—C24—C29	-85.6 (2)
C12—C7—C8—O1	176.94 (15)	C29—C24—C25—O3	-176.59 (17)
C6—C7—C8—O1	-5.40 (19)	C23—C24—C25—O3	6.6 (2)
C12—C7—C8—C11	-7.2 (3)	C29—C24—C25—C28	8.8 (4)
C6—C7—C8—C11	170.46 (17)	C23—C24—C25—C28	-168.0 (2)
C1—O1—C8—C7	-16.41 (18)	C18—O3—C25—C24	15.6 (2)
C1—O1—C8—C11	166.96 (14)	C18—O3—C25—C28	-168.76 (18)
C8—C7—C12—C17	125.25 (19)	C25—C24—C29—C34	-137.4 (2)
C6—C7—C12—C17	-52.1 (2)	C23—C24—C29—C34	38.8 (3)
C8—C7—C12—C13	-57.6 (2)	C25—C24—C29—C30	44.6 (3)
C6—C7—C12—C13	125.11 (17)	C23—C24—C29—C30	-139.13 (18)

C17—C12—C13—C14	1.1 (2)	C34—C29—C30—C31	0.5 (3)
C7—C12—C13—C14	-176.16 (16)	C24—C29—C30—C31	178.56 (17)
C12—C13—C14—C15	-1.4 (3)	C29—C30—C31—C32	-0.6 (3)
C13—C14—C15—C16	0.5 (3)	C30—C31—C32—C33	0.5 (3)
C14—C15—C16—C17	0.8 (3)	C31—C32—C33—C34	-0.3 (3)
C15—C16—C17—C12	-1.1 (3)	C30—C29—C34—C33	-0.3 (3)
C13—C12—C17—C16	0.2 (2)	C24—C29—C34—C33	-178.29 (18)
C7—C12—C17—C16	177.40 (15)	C32—C33—C34—C29	0.2 (3)

Hydrogen-bond geometry (Å, º) for (EAG3179P)

D—H···*A*	*D*—H	H···*A*	*D*···*A*	*D*—H···*A*
O2—H20*H*···O4^{i}	0.84	1.98	2.7962 (17)	164
O4—H40*H*···O2	0.84	1.97	2.7935 (16)	166

Symmetry code: (i) -*x*+3/2, *y*, -*z*+1.

Compound (±)-12

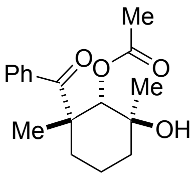

(±)-12

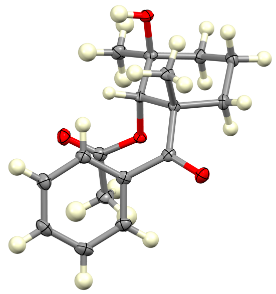

Sample Name: CCDC 2426295 / Armend17
Crystal Growth: Slow evaporation using a mixture of hexanes and dichloromethane
Ellipsoid Contour: Set at a 50% probability level

Crystal data

$C_{17}H_{22}O_4$	D_x = 1.282 Mg m^{-3}
M_r = 290.34	Cu *K*a radiation, l = 1.54184 Å
Orthorhombic, *Pbca*	Cell parameters from 9887 reflections
a = 10.8438 (6) Å	q = 2.8–78.8°
b = 8.8468 (5) Å	m = 0.73 mm^{-1}
c = 31.3666 (16) Å	*T* = 100 K
V = 3009.1 (3) Å^3	Prism, colourless
Z = 8	0.23 × 0.13 × 0.12 mm
F(000) = 1248	

Data collection

Bruker D8 Venture DUO with Photon III C14 diffractometer	3061 reflections with $I > 2s(I)$
Radiation source: ImS 3.0 microfocus	R_{int} = 0.033
f and w scans	q_{max} = 79.2°, q_{min} = 2.8°
Absorption correction: multi-scan *SADABS* (Krause *et al.*, 2015)	*h* = -11 - 13
T_{min} = 0.813, T_{max} = 0.917	*k* = -11 - 8
23300 measured reflections	*l* = -36 - 39
3209 independent reflections	

Refinement

Refinement on F^2	0 restraints
Least-squares matrix: full	Hydrogen site location: mixed

$R[F^2 > 2s(F^2)] = 0.034$	H atoms treated by a mixture of independent and constrained refinement
$wR(F^2) = 0.088$	$w = 1/[s^2(F_o^2) + (0.0404P)^2 + 1.327P]$ where $P = (F_o^2 + 2F_c^2)/3$
$S = 1.04$	$(D/s)_{max} < 0.001$
3209 reflections	$Dr_{max} = 0.31$ e Å^{-3}
196 parameters	$Dr_{min} = -0.25$ e Å^{-3}

Fractional atomic coordinates and isotropic or equivalent isotropic displacement parameters (Å^2) for (Armend17)

	x	y	z	U_{iso}*/U_{eq}
O1	0.42011 (7)	0.03627 (8)	0.43620 (2)	0.01919 (17)
H1OH	0.3491 (15)	0.0396 (18)	0.4245 (5)	0.029*
O2	0.50593 (6)	0.42524 (8)	0.40691 (2)	0.01510 (16)
O3	0.30805 (7)	0.49585 (9)	0.39711 (3)	0.02276 (18)
O4	0.70988 (7)	0.32625 (9)	0.32071 (3)	0.02315 (18)
C1	0.47368 (9)	0.26638 (11)	0.39960 (3)	0.0136 (2)
H1	0.392181	0.261270	0.384757	0.016*
C2	0.46292 (9)	0.18716 (11)	0.44350 (3)	0.0159 (2)
C3	0.58876 (10)	0.17120 (12)	0.46473 (3)	0.0183 (2)
H3A	0.621783	0.272755	0.471467	0.022*
H3B	0.579729	0.114831	0.491814	0.022*
C4	0.67883 (10)	0.08821 (12)	0.43574 (3)	0.0194 (2)
H4A	0.646870	-0.014224	0.429464	0.023*
H4B	0.759255	0.077303	0.450354	0.023*
C5	0.69630 (9)	0.17522 (12)	0.39418 (3)	0.0179 (2)
H5A	0.753566	0.118147	0.375603	0.022*
H5B	0.735005	0.273965	0.400622	0.022*
C6	0.57471 (9)	0.20290 (11)	0.36968 (3)	0.0144 (2)
C7	0.37269 (10)	0.26907 (13)	0.47287 (3)	0.0214 (2)
H7A	0.404960	0.369708	0.479649	0.032*
H7B	0.362430	0.211022	0.499238	0.032*
H7C	0.292761	0.279054	0.458559	0.032*
C8	0.53297 (10)	0.05433 (11)	0.34785 (3)	0.0182 (2)
H8A	0.590337	0.029220	0.324768	0.027*
H8B	0.449893	0.067536	0.336120	0.027*
H8C	0.532268	-0.027702	0.368840	0.027*
C9	0.60422 (9)	0.31601 (11)	0.33359 (3)	0.0161 (2)
C10	0.50837 (10)	0.41460 (12)	0.31303 (3)	0.0169 (2)

C11	0.38731 (10)	0.36975 (12)	0.30472 (3)	0.0192 (2)
H11	0.359024	0.273841	0.314199	0.023*
C12	0.30797 (11)	0.46530 (13)	0.28257 (3)	0.0226 (2)
H12	0.226453	0.433031	0.276232	0.027*
C13	0.34722 (11)	0.60726 (13)	0.26971 (3)	0.0242 (2)
H13	0.292893	0.671816	0.254448	0.029*
C14	0.46620 (12)	0.65497 (13)	0.27920 (4)	0.0257 (2)
H14	0.492196	0.753704	0.271386	0.031*
C15	0.54699 (10)	0.55861 (13)	0.30005 (3)	0.0212 (2)
H15	0.629117	0.590422	0.305587	0.025*
C16	0.41469 (10)	0.52812 (11)	0.40381 (3)	0.0174 (2)
C17	0.46152 (11)	0.68528 (12)	0.41001 (4)	0.0260 (2)
H17A	0.398388	0.757693	0.400913	0.039*
H17B	0.536325	0.699877	0.392966	0.039*
H17C	0.480583	0.701430	0.440205	0.039*

Atomic displacement parameters (Å^2) for (Armend17)

	U^{11}	U^{22}	U^{33}	U^{12}	U^{13}	U^{23}
O1	0.0196 (4)	0.0151 (4)	0.0229 (4)	-0.0039 (3)	-0.0028 (3)	0.0026 (3)
O2	0.0155 (3)	0.0113 (3)	0.0185 (3)	-0.0002 (3)	0.0011 (3)	-0.0019 (3)
O3	0.0174 (4)	0.0217 (4)	0.0293 (4)	0.0042 (3)	0.0019 (3)	-0.0029 (3)
O4	0.0179 (4)	0.0259 (4)	0.0257 (4)	-0.0004 (3)	0.0052 (3)	0.0014 (3)
C1	0.0137 (4)	0.0108 (4)	0.0164 (4)	-0.0007 (3)	-0.0005 (3)	-0.0014 (3)
C2	0.0172 (5)	0.0141 (5)	0.0163 (4)	-0.0016 (4)	0.0001 (4)	-0.0004 (4)
C3	0.0195 (5)	0.0193 (5)	0.0161 (4)	-0.0004 (4)	-0.0031 (4)	-0.0001 (4)
C4	0.0178 (5)	0.0199 (5)	0.0206 (5)	0.0031 (4)	-0.0040 (4)	-0.0004 (4)
C5	0.0141 (5)	0.0197 (5)	0.0199 (5)	0.0023 (4)	-0.0009 (4)	-0.0022 (4)
C6	0.0145 (4)	0.0132 (4)	0.0155 (4)	0.0008 (4)	0.0002 (4)	-0.0014 (3)
C7	0.0223 (5)	0.0238 (5)	0.0181 (5)	0.0014 (4)	0.0049 (4)	0.0006 (4)
C8	0.0212 (5)	0.0141 (5)	0.0194 (5)	0.0002 (4)	-0.0008	-0.0031

					(4)	(4)
C9	0.0176 (5)	0.0151 (5)	0.0157 (4)	-0.0014 (4)	0.0010 (4)	-0.0042 (4)
C10	0.0207 (5)	0.0179 (5)	0.0121 (4)	0.0008 (4)	0.0021 (4)	-0.0013 (4)
C11	0.0220 (5)	0.0176 (5)	0.0181 (5)	0.0002 (4)	-0.0003 (4)	-0.0005 (4)
C12	0.0232 (5)	0.0243 (5)	0.0202 (5)	0.0024 (4)	-0.0031 (4)	-0.0015 (4)
C13	0.0311 (6)	0.0242 (5)	0.0174 (5)	0.0077 (5)	-0.0014 (4)	0.0027 (4)
C14	0.0349 (6)	0.0210 (5)	0.0213 (5)	-0.0002 (5)	0.0029 (5)	0.0066 (4)
C15	0.0235 (5)	0.0225 (5)	0.0176 (5)	-0.0028 (4)	0.0026 (4)	0.0024 (4)
C16	0.0200 (5)	0.0158 (5)	0.0163 (5)	0.0026 (4)	0.0042 (4)	-0.0009 (3)
C17	0.0293 (6)	0.0147 (5)	0.0338 (6)	0.0016 (4)	0.0034 (5)	-0.0030 (4)

Geometric parameters (Å, º) for (Armend17)

O1—C2	1.4317 (12)	C7—H7A	0.9800
O1—H10H	0.853 (17)	C7—H7B	0.9800
O2—C16	1.3478 (12)	C7—H7C	0.9800
O2—C1	1.4663 (11)	C8—H8A	0.9800
O3—C16	1.2095 (14)	C8—H8B	0.9800
O4—C9	1.2182 (13)	C8—H8C	0.9800
C1—C6	1.5480 (13)	C9—C10	1.5023 (14)
C1—C2	1.5493 (13)	C10—C11	1.3959 (15)
C1—H1	1.0000	C10—C15	1.4015 (15)
C2—C3	1.5249 (14)	C11—C12	1.3919 (15)
C2—C7	1.5268 (14)	C11—H11	0.9500
C3—C4	1.5231 (14)	C12—C13	1.3860 (16)
C3—H3A	0.9900	C12—H12	0.9500
C3—H3B	0.9900	C13—C14	1.3897 (18)
C4—C5	1.5257 (14)	C13—H13	0.9500
C4—H4A	0.9900	C14—C15	1.3864 (16)
C4—H4B	0.9900	C14—H14	0.9500
C5—C6	1.5455 (13)	C15—H15	0.9500
C5—H5A	0.9900	C16—C17	1.4929 (15)

C5—H5B	0.9900	C17—H17A	0.9800
C6—C9	1.5445 (14)	C17—H17B	0.9800
C6—C8	1.5497 (13)	C17—H17C	0.9800
C2—O1—H10H	109.2 (11)	H7A—C7—H7B	109.5
C16—O2—C1	117.45 (8)	C2—C7—H7C	109.5
O2—C1—C6	105.89 (7)	H7A—C7—H7C	109.5
O2—C1—C2	108.22 (7)	H7B—C7—H7C	109.5
C6—C1—C2	115.34 (8)	C6—C8—H8A	109.5
O2—C1—H1	109.1	C6—C8—H8B	109.5
C6—C1—H1	109.1	H8A—C8—H8B	109.5
C2—C1—H1	109.1	C6—C8—H8C	109.5
O1—C2—C3	105.88 (8)	H8A—C8—H8C	109.5
O1—C2—C7	109.34 (8)	H8B—C8—H8C	109.5
C3—C2—C7	110.73 (8)	O4—C9—C10	117.72 (9)
O1—C2—C1	107.70 (8)	O4—C9—C6	119.08 (9)
C3—C2—C1	111.26 (8)	C10—C9—C6	123.19 (9)
C7—C2—C1	111.70 (8)	C11—C10—C15	119.02 (10)
C4—C3—C2	110.96 (8)	C11—C10—C9	124.46 (9)
C4—C3—H3A	109.4	C15—C10—C9	116.47 (9)
C2—C3—H3A	109.4	C12—C11—C10	120.12 (10)
C4—C3—H3B	109.4	C12—C11—H11	119.9
C2—C3—H3B	109.4	C10—C11—H11	119.9
H3A—C3—H3B	108.0	C13—C12—C11	120.38 (11)
C3—C4—C5	110.27 (8)	C13—C12—H12	119.8
C3—C4—H4A	109.6	C11—C12—H12	119.8
C5—C4—H4A	109.6	C12—C13—C14	119.87 (10)
C3—C4—H4B	109.6	C12—C13—H13	120.1
C5—C4—H4B	109.6	C14—C13—H13	120.1
H4A—C4—H4B	108.1	C15—C14—C13	120.06 (11)
C4—C5—C6	113.50 (8)	C15—C14—H14	120.0
C4—C5—H5A	108.9	C13—C14—H14	120.0
C6—C5—H5A	108.9	C14—C15—C10	120.48 (11)
C4—C5—H5B	108.9	C14—C15—H15	119.8
C6—C5—H5B	108.9	C10—C15—H15	119.8
H5A—C5—H5B	107.7	O3—C16—O2	123.71 (9)
C9—C6—C5	106.88 (8)	O3—C16—C17	124.59 (10)
C9—C6—C1	110.85 (8)	O2—C16—C17	111.70 (9)
C5—C6—C1	111.09 (8)	C16—C17—H17A	109.5

C9—C6—C8	106.62 (8)	C16—C17—H17B	109.5
C5—C6—C8	109.55 (8)	H17A—C17—H17B	109.5
C1—C6—C8	111.65 (8)	C16—C17—H17C	109.5
C2—C7—H7A	109.5	H17A—C17—H17C	109.5
C2—C7—H7B	109.5	H17B—C17—H17C	109.5
C16—O2—C1—C6	134.16 (8)	C2—C1—C6—C8	78.28 (10)
C16—O2—C1—C2	-101.64 (9)	C5—C6—C9—O4	23.51 (12)
O2—C1—C2—O1	174.62 (7)	C1—C6—C9—O4	144.71 (9)
C6—C1—C2—O1	-67.05 (10)	C8—C6—C9—O4	-93.58 (11)
O2—C1—C2—C3	-69.76 (10)	C5—C6—C9—C10	-156.92 (9)
C6—C1—C2—C3	48.57 (11)	C1—C6—C9—C10	-35.72 (12)
O2—C1—C2—C7	54.54 (10)	C8—C6—C9—C10	85.99 (11)
C6—C1—C2—C7	172.88 (8)	O4—C9—C10—C11	142.69 (10)
O1—C2—C3—C4	61.04 (10)	C6—C9—C10—C11	-36.89 (14)
C7—C2—C3—C4	179.46 (9)	O4—C9—C10—C15	-34.60 (13)
C1—C2—C3—C4	-55.69 (11)	C6—C9—C10—C15	145.82 (9)
C2—C3—C4—C5	60.40 (11)	C15—C10—C11—C12	2.17 (15)
C3—C4—C5—C6	-57.29 (11)	C9—C10—C11—C12	-175.06 (9)
C4—C5—C6—C9	169.60 (8)	C10—C11—C12—C13	-1.92 (16)
C4—C5—C6—C1	48.56 (11)	C11—C12—C13—C14	-0.40 (16)
C4—C5—C6—C8	-75.26 (10)	C12—C13—C14—C15	2.45 (17)
O2—C1—C6—C9	-43.38 (10)	C13—C14—C15—C10	-2.19 (16)
C2—C1—C6—C9	-163.01 (8)	C11—C10—C15—C14	-0.12 (15)
O2—C1—C6—C5	75.30 (9)	C9—C10—C15—C14	177.32 (9)
C2—C1—C6—C5	-44.33 (11)	C1—O2—C16—O3	3.09 (14)
O2—C1—C6—C8	-162.09 (8)	C1—O2—C16—C17	-177.38 (8)

Hydrogen-bond geometry (Å, º) for (Armend17)

D—H···*A*	*D*—H	H···*A*	*D*···*A*	*D*—H···*A*
O1—H10*H*···O3^{i}	0.853 (17)	1.948 (17)	2.7842 (11)	166.5 (15)
C3—H3*B*···O1^{ii}	0.99	2.62	3.6105 (13)	174

Symmetry codes: (i) -*x*+1/2, *y*-1/2, *z*; (ii) -*x*+1, -*y*, -*z*+1.

5a

^{1}H NMR
(400 MHz, $CDCl_3$)

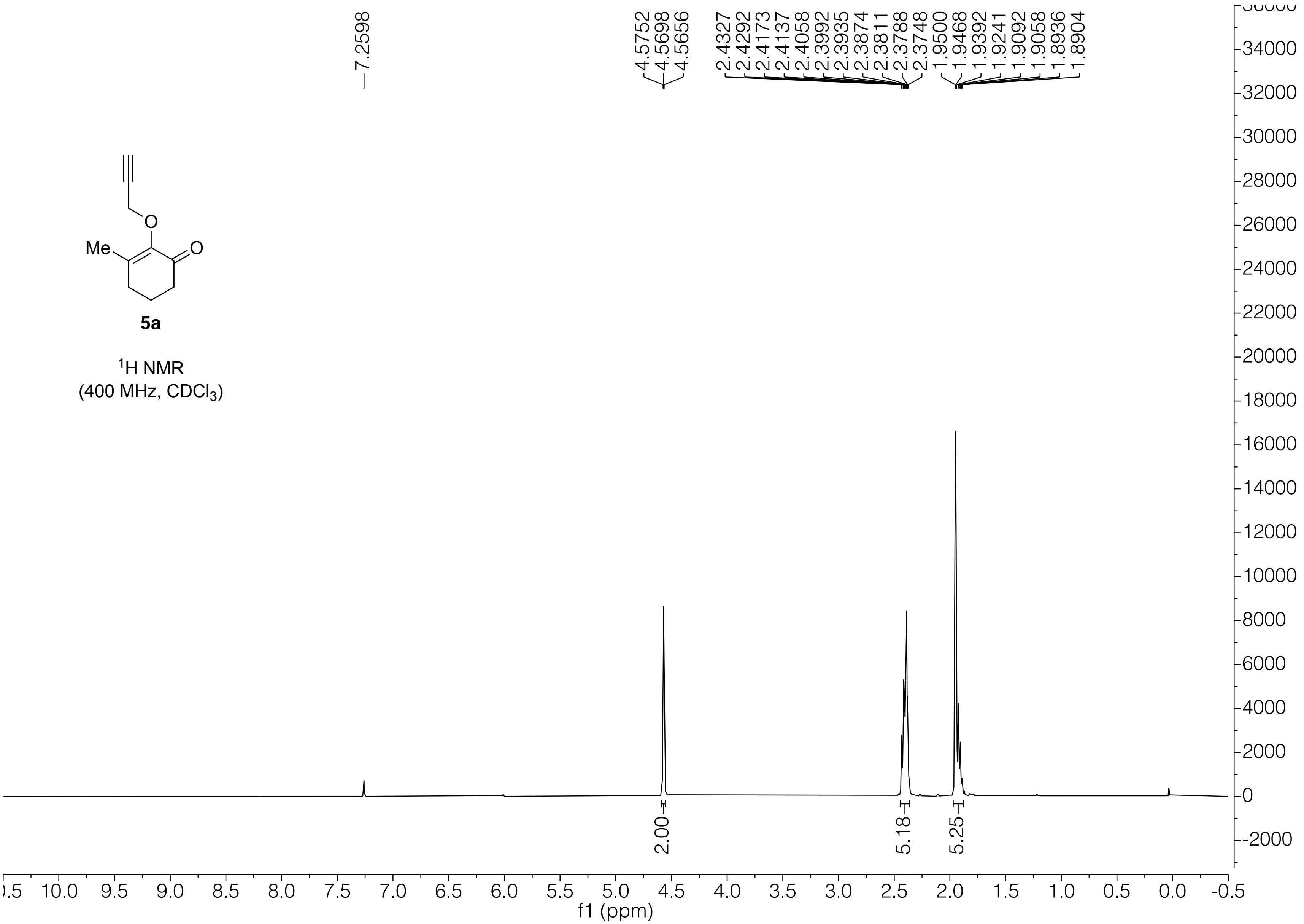

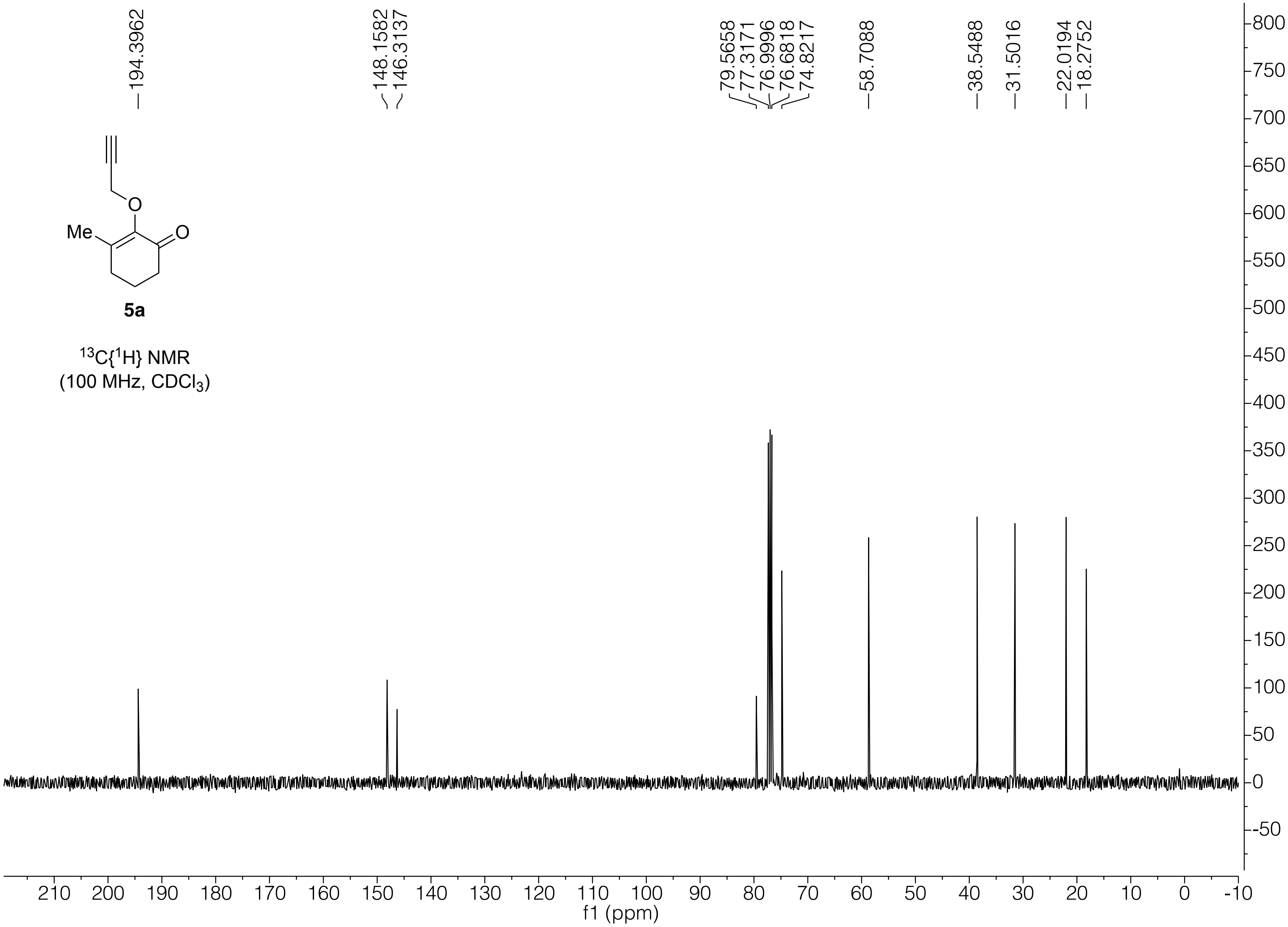
194.3962
148.1582
146.3137
79.5658
77.3171
76.9996
76.6818
74.8217
58.7088
38.5488
31.5016
22.0194
18.2752
Me
O
O
5a
$^{13}C\{^{1}H\}$ NMR
(100 MHz, $CDCl_3$)
f1 (ppm)

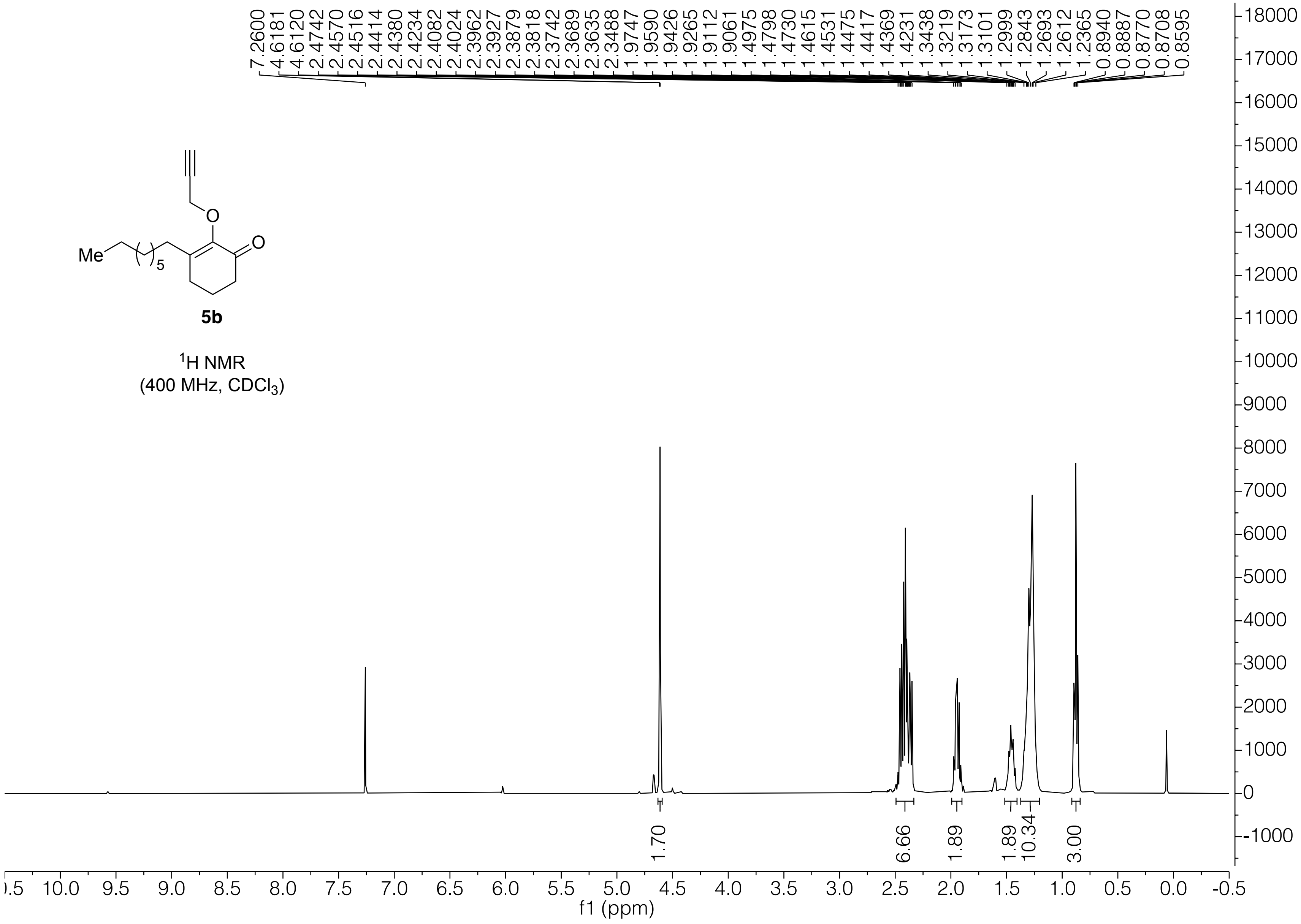

7.2600 4.6181 4.6120 2.4742 2.4570 2.4516 2.4414 2.4380 2.4234 2.4082 2.4024 2.3962 2.3927 2.3879 2.3818 2.3742 2.3689 2.3635 2.3488 1.9747 1.9590 1.9426 1.9265 1.9112 1.9061 1.4975 1.4798 1.4730 1.4615 1.4531 1.4475 1.4417 1.4369 1.4231 1.3438 1.3219 1.3173 1.3101 1.2999 1.2843 1.2693 1.2612 1.2365 0.8940 0.8887 0.8770 0.8708 0.8595
Me
O
O
5
5b
^{1}H NMR
(400 MHz, $CDCl_3$)
1.70
6.66
1.89
1.89
10.34
3.00
f1 (ppm)

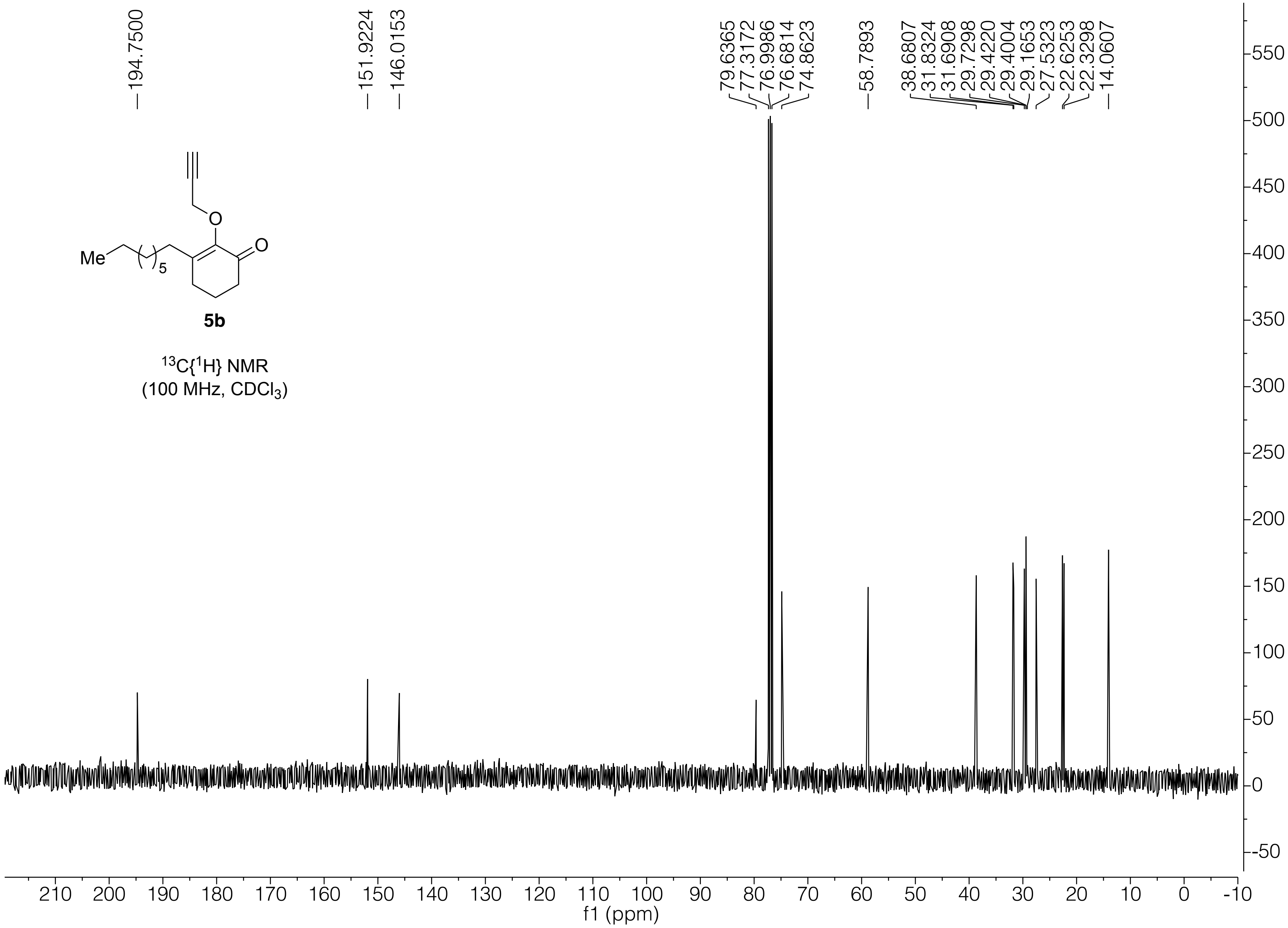
194.7500
151.9224
146.0153
79.6365
77.3172
76.9986
76.6814
74.8623
58.7893
38.6807
31.8324
31.6908
29.7298
29.4220
29.4004
29.1653
27.5323
22.6253
22.3298
14.0607
Me
O
O
5
5b
13C{1H} NMR
(100 MHz, CDCl3)
f1 (ppm)

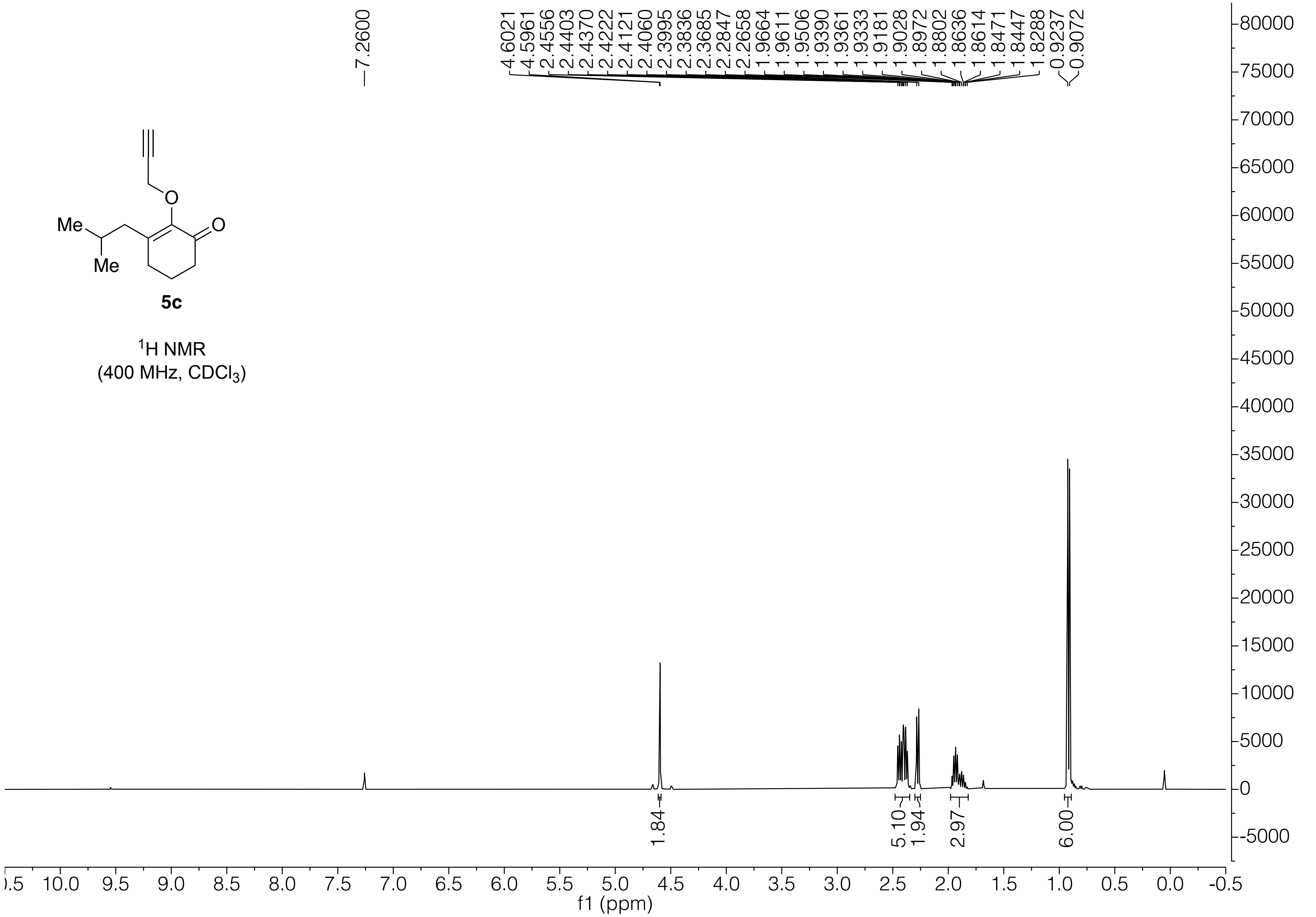

7.2600
4.6021
4.5961
2.4556
2.4403
2.4370
2.4222
2.4121
2.4060
2.3995
2.3836
2.3685
2.2847
2.2658
1.9664
1.9611
1.9506
1.9390
1.9361
1.9333
1.9181
1.9028
1.8972
1.8802
1.8636
1.8614
1.8471
1.8447
1.8288
0.9237
0.9072
Me
Me
O
O
5c
^{1}H NMR
(400 MHz, $CDCl_3$)
1.84
5.10
1.94
2.97
6.00
f1 (ppm)

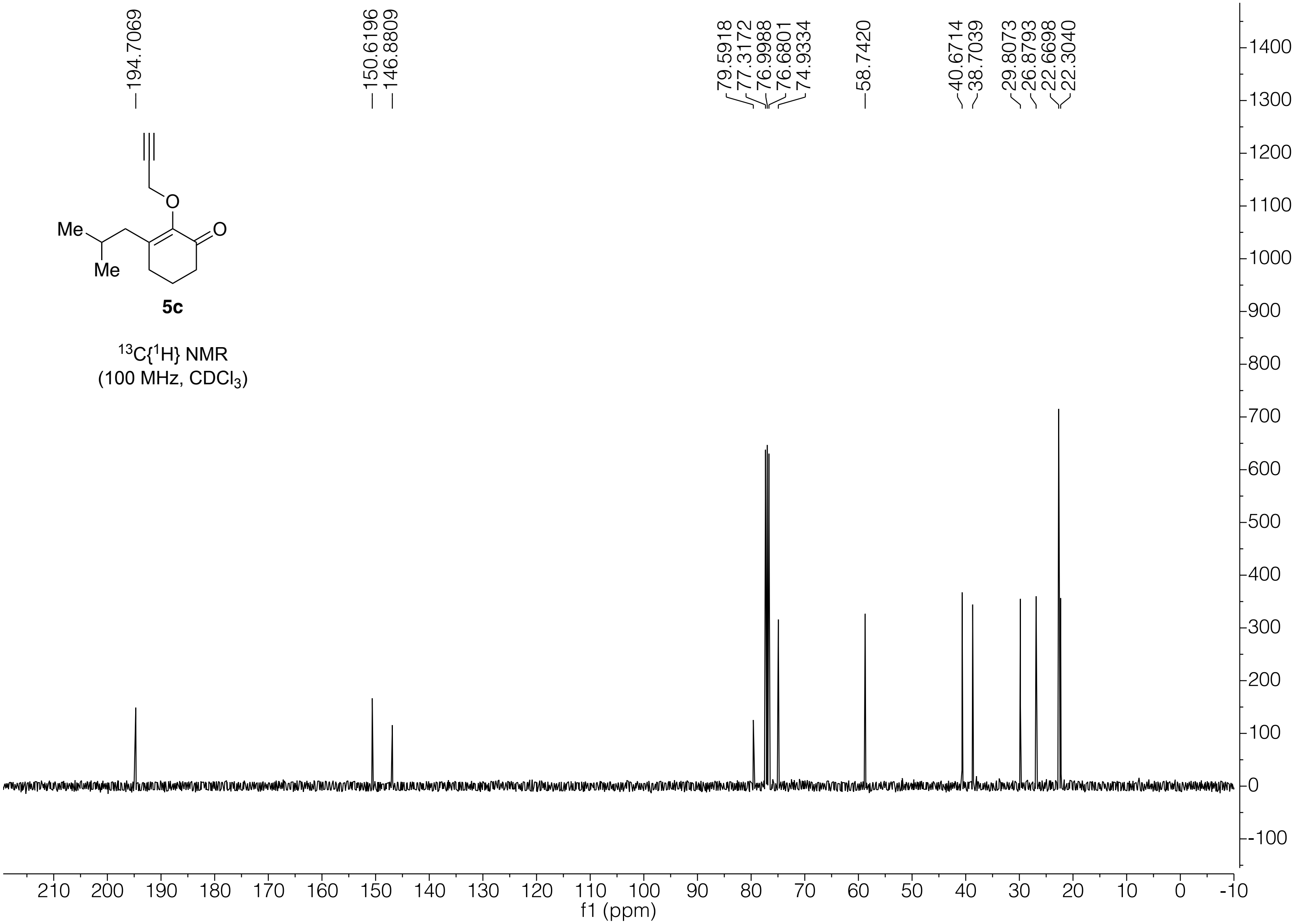
194.7069
150.6196
146.8809
79.5918
77.3172
76.9988
76.6801
74.9334
58.7420
40.6714
38.7039
29.8073
26.8793
22.6698
22.3040
Me
Me
O
O
5c
$^{13}C\{^1H\}$ NMR
(100 MHz, $CDCl_3$)
f1 (ppm)

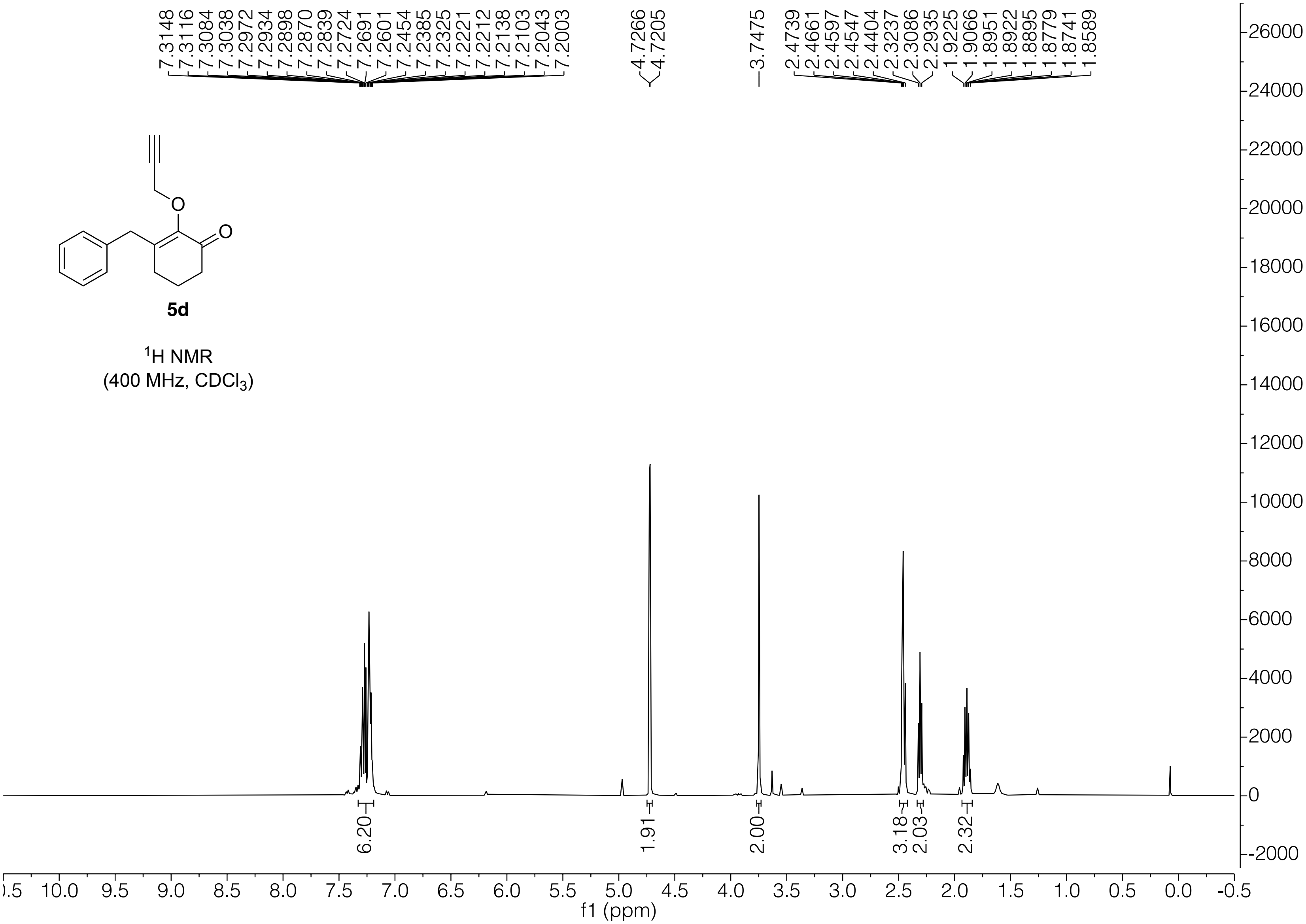
7.3148
7.3116
7.3084
7.3038
7.2972
7.2934
7.2898
7.2870
7.2839
7.2724
7.2691
7.2601
7.2454
7.2385
7.2325
7.2221
7.2212
7.2138
7.2103
7.2043
7.2003
4.7266
4.7205
3.7475
2.4739
2.4661
2.4597
2.4547
2.4404
2.3237
2.3086
2.2935
1.9225
1.9066
1.8951
1.8922
1.8895
1.8779
1.8741
1.8589
5d
1H NMR
(400 MHz, CDCl3)
6.20
1.91
2.00
3.18
2.03
2.32
f1 (ppm)

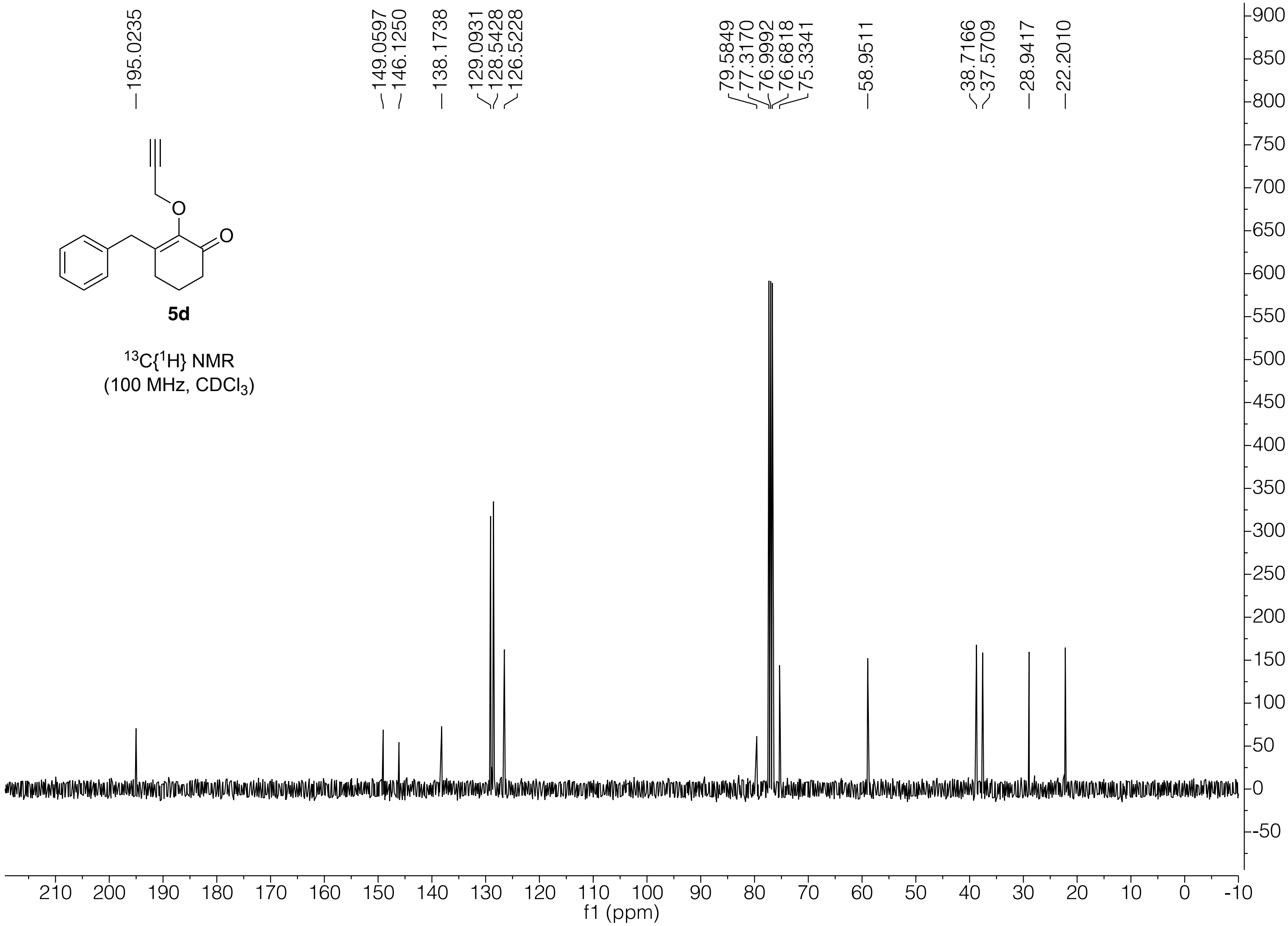
195.0235
149.0597
146.1250
138.1738
129.0931
128.5428
126.5228
79.5849
77.3170
76.9992
76.6818
75.3341
58.9511
38.7166
37.5709
28.9417
22.2010
5d
$^{13}C\{^1H\}$ NMR
(100 MHz, $CDCl_3$)
f1 (ppm)

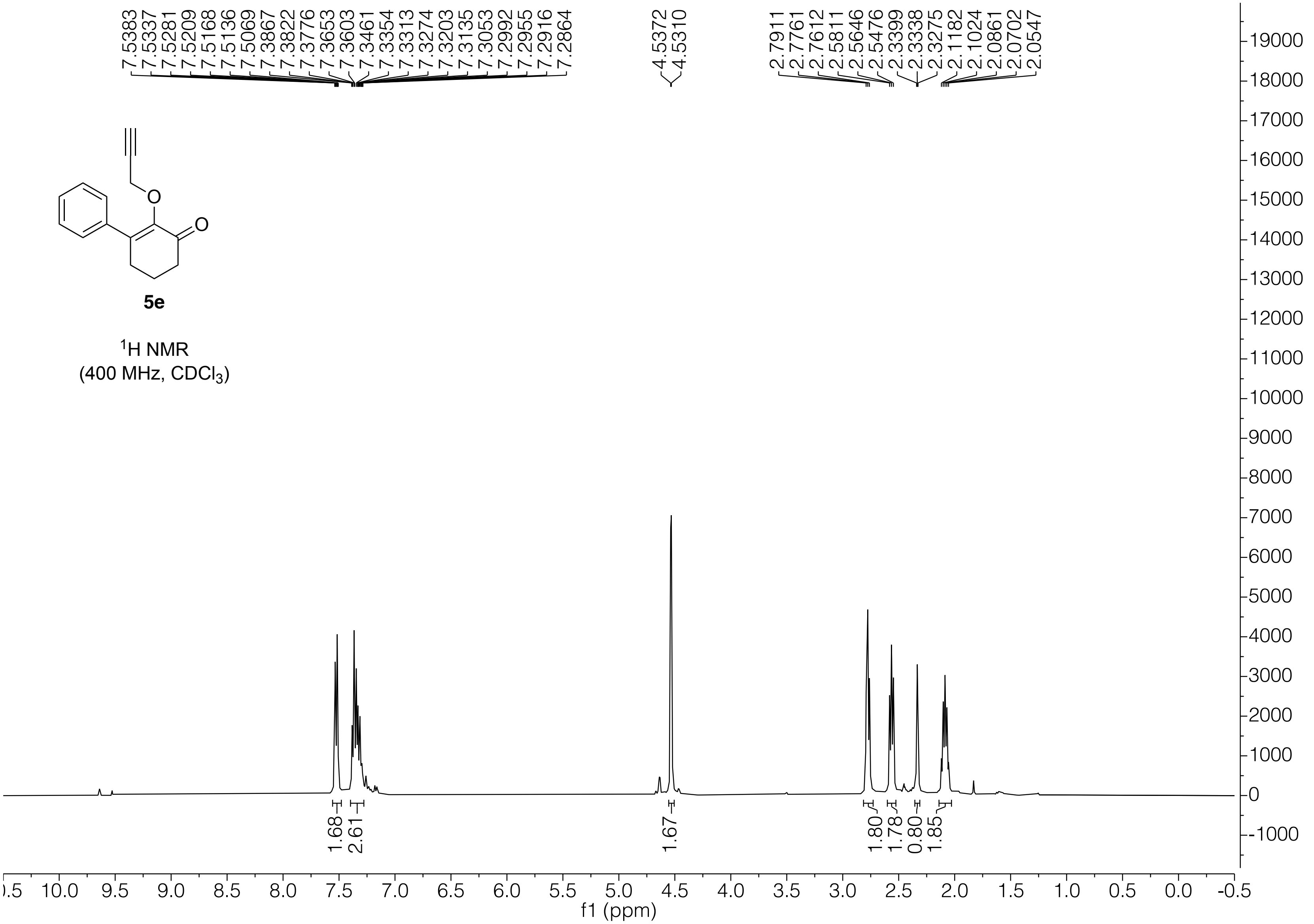
7.5383
7.5337
7.5281
7.5209
7.5168
7.5136
7.5069
7.3867
7.3822
7.3776
7.3653
7.3603
7.3461
7.3354
7.3313
7.3274
7.3203
7.3135
7.3053
7.2992
7.2955
7.2916
7.2864
4.5372
4.5310
2.7911
2.7761
2.7612
2.5811
2.5646
2.5476
2.3399
2.3338
2.3275
2.1182
2.1024
2.0861
2.0702
2.0547
O
O
5e
^{1}H NMR
(400 MHz, $CDCl_3$)
1.68
2.61
1.67
1.80
1.78
0.80
1.85
f1 (ppm)

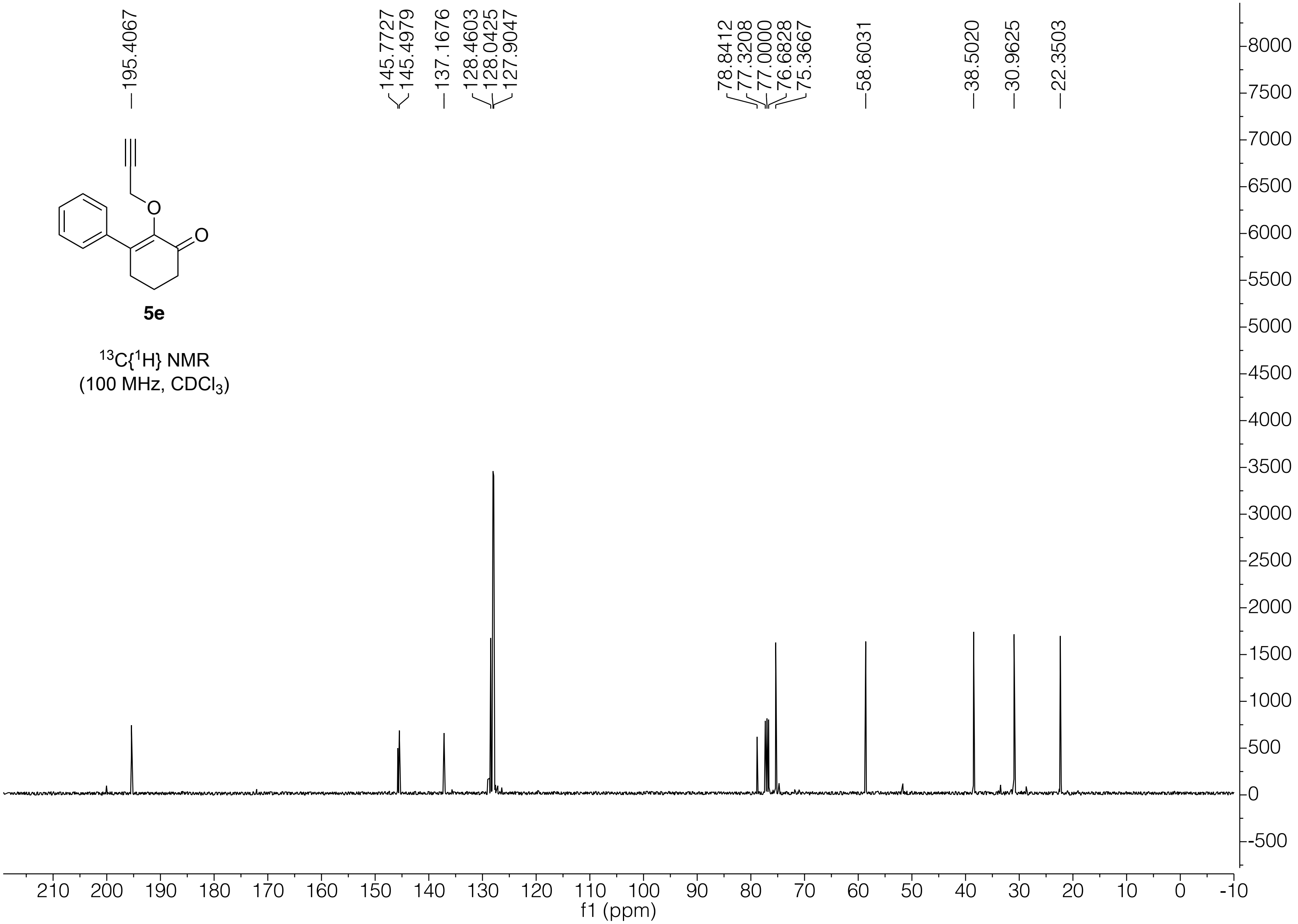
195.4067
145.7727
145.4979
137.1676
128.4603
128.0425
127.9047
78.8412
77.3208
77.0000
76.6828
75.3667
58.6031
38.5020
30.9625
22.3503
5e
$^{13}C\{^1H\}$ NMR
(100 MHz, $CDCl_3$)
f1 (ppm)

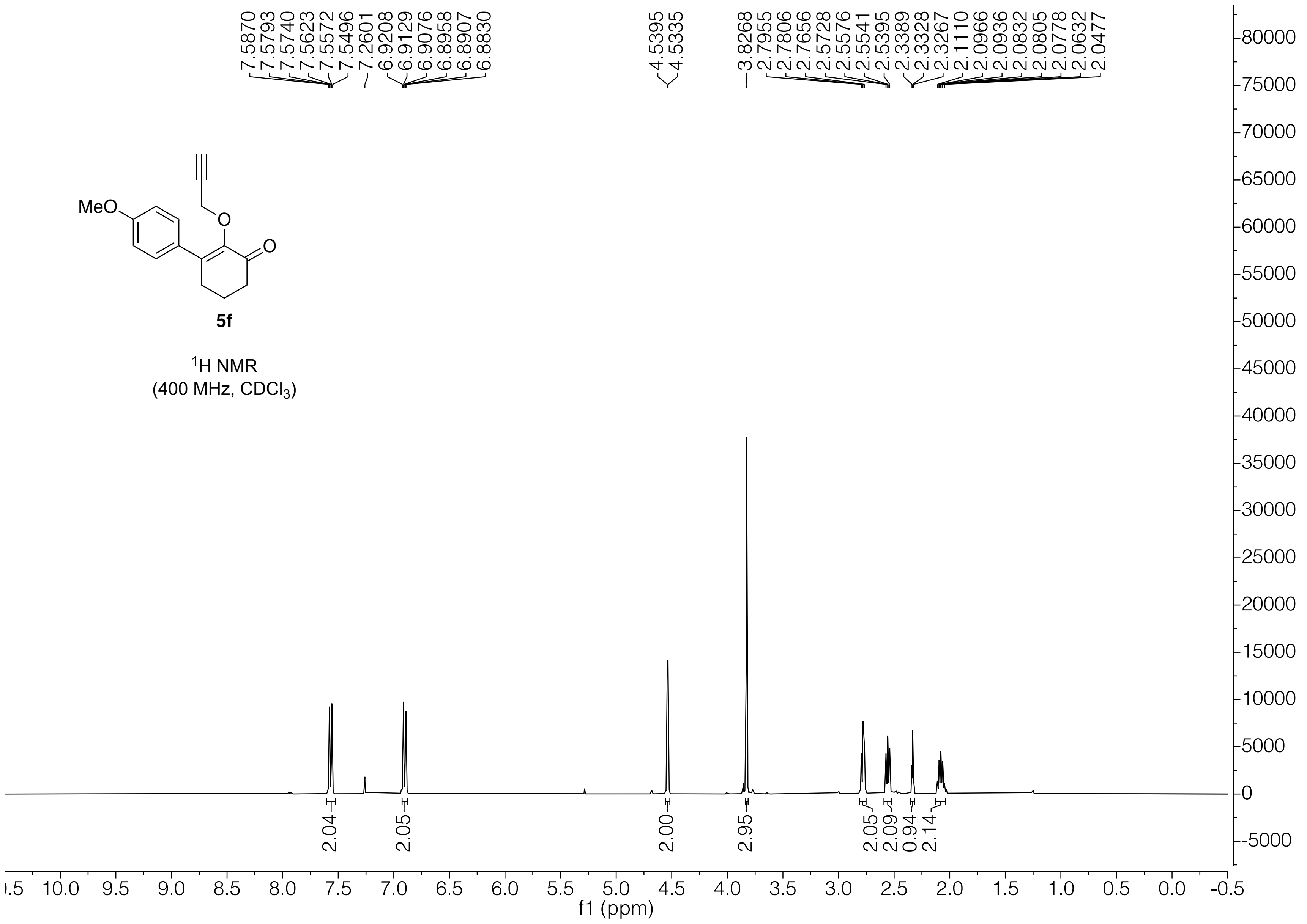
7.5870
7.5793
7.5740
7.5623
7.5572
7.5496
7.2601
6.9208
6.9129
6.9076
6.8958
6.8907
6.8830
4.5395
4.5335
3.8268
2.7955
2.7806
2.7656
2.5728
2.5576
2.5541
2.5395
2.3389
2.3328
2.3267
2.1110
2.0966
2.0936
2.0832
2.0805
2.0778
2.0632
2.0477
MeO
O
O
5f
1H NMR
(400 MHz, CDCl3)
2.04
2.05
2.00
2.95
2.05
2.09
0.94
2.14
f1 (ppm)

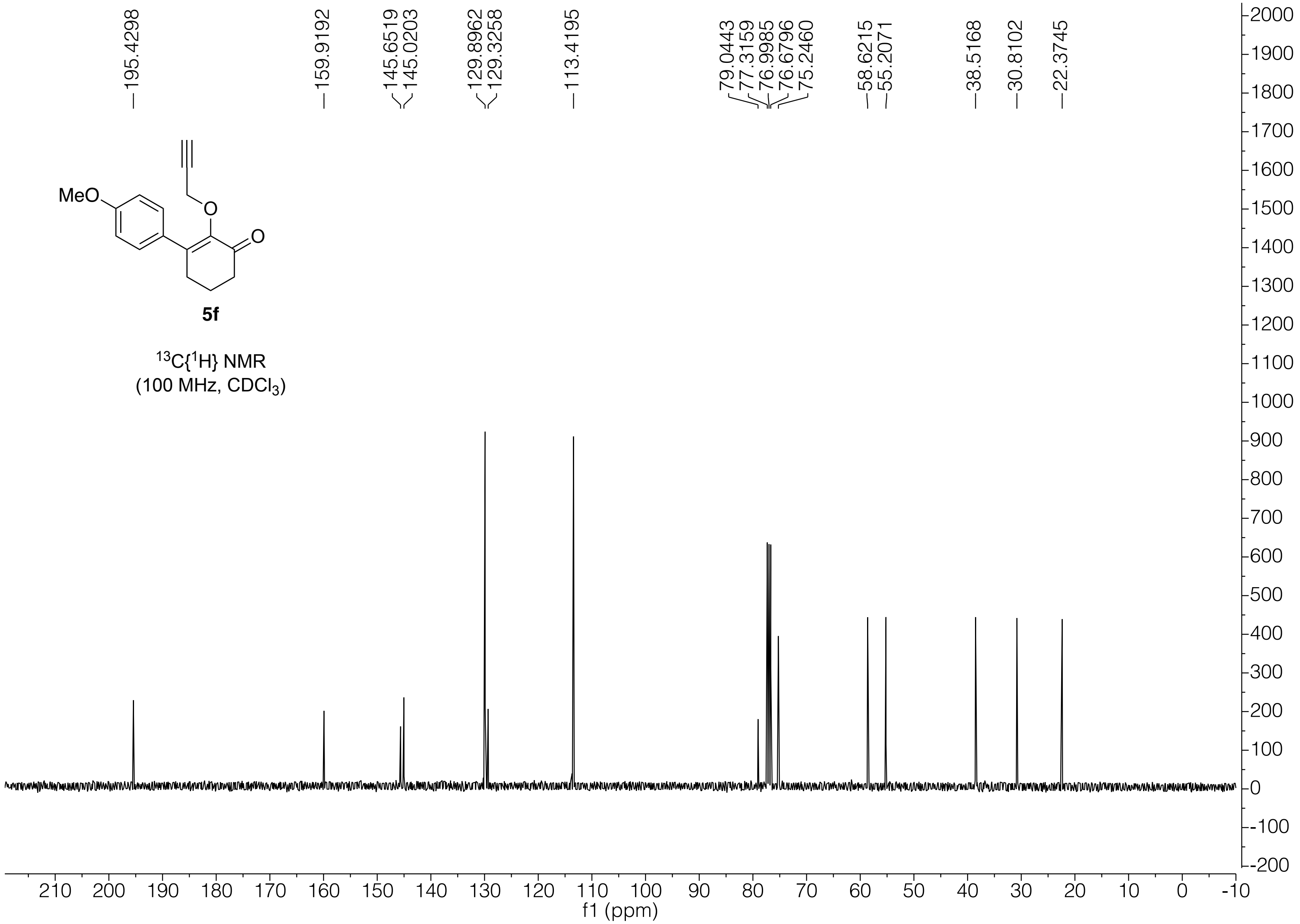

195.4298
159.9192
145.6519
145.0203
129.8962
129.3258
113.4195
79.0443
77.3159
76.9985
76.6796
75.2460
58.6215
55.2071
38.5168
30.8102
22.3745
MeO
O
O
5f
$^{13}C\{^1H\}$ NMR
(100 MHz, $CDCl_3$)
f1 (ppm)

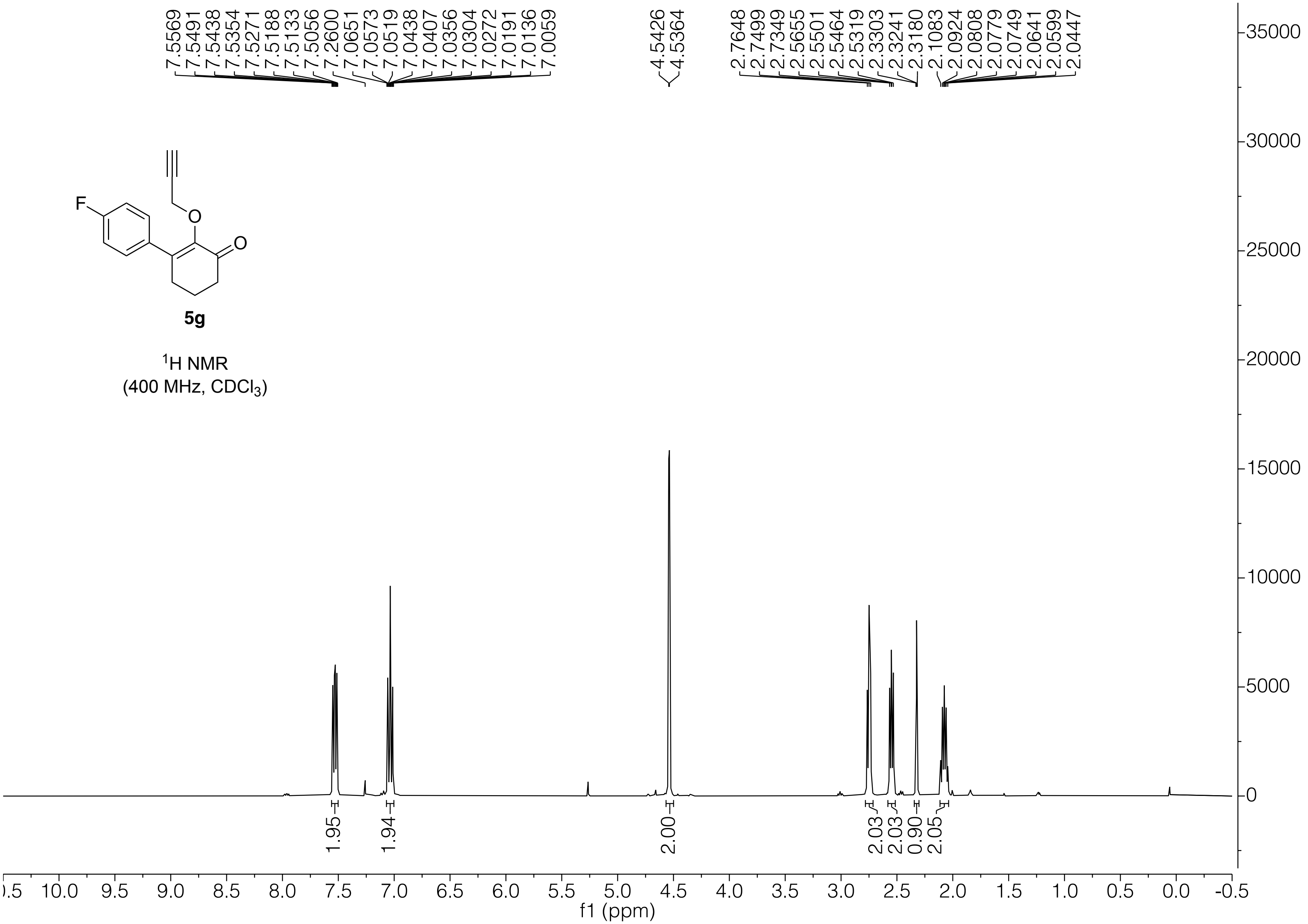
7.5569
7.5491
7.5438
7.5354
7.5271
7.5188
7.5133
7.5056
7.2600
7.0651
7.0573
7.0519
7.0438
7.0407
7.0356
7.0304
7.0272
7.0191
7.0136
7.0059
4.5426
4.5364
2.7648
2.7499
2.7349
2.5655
2.5501
2.5464
2.5319
2.3303
2.3241
2.3180
2.1083
2.0924
2.0808
2.0779
2.0749
2.0641
2.0599
2.0447
F
O
O
5g
^{1}H NMR
(400 MHz, $CDCl_3$)
1.95
1.94
2.00
2.03
2.03
0.90
2.05
f1 (ppm)

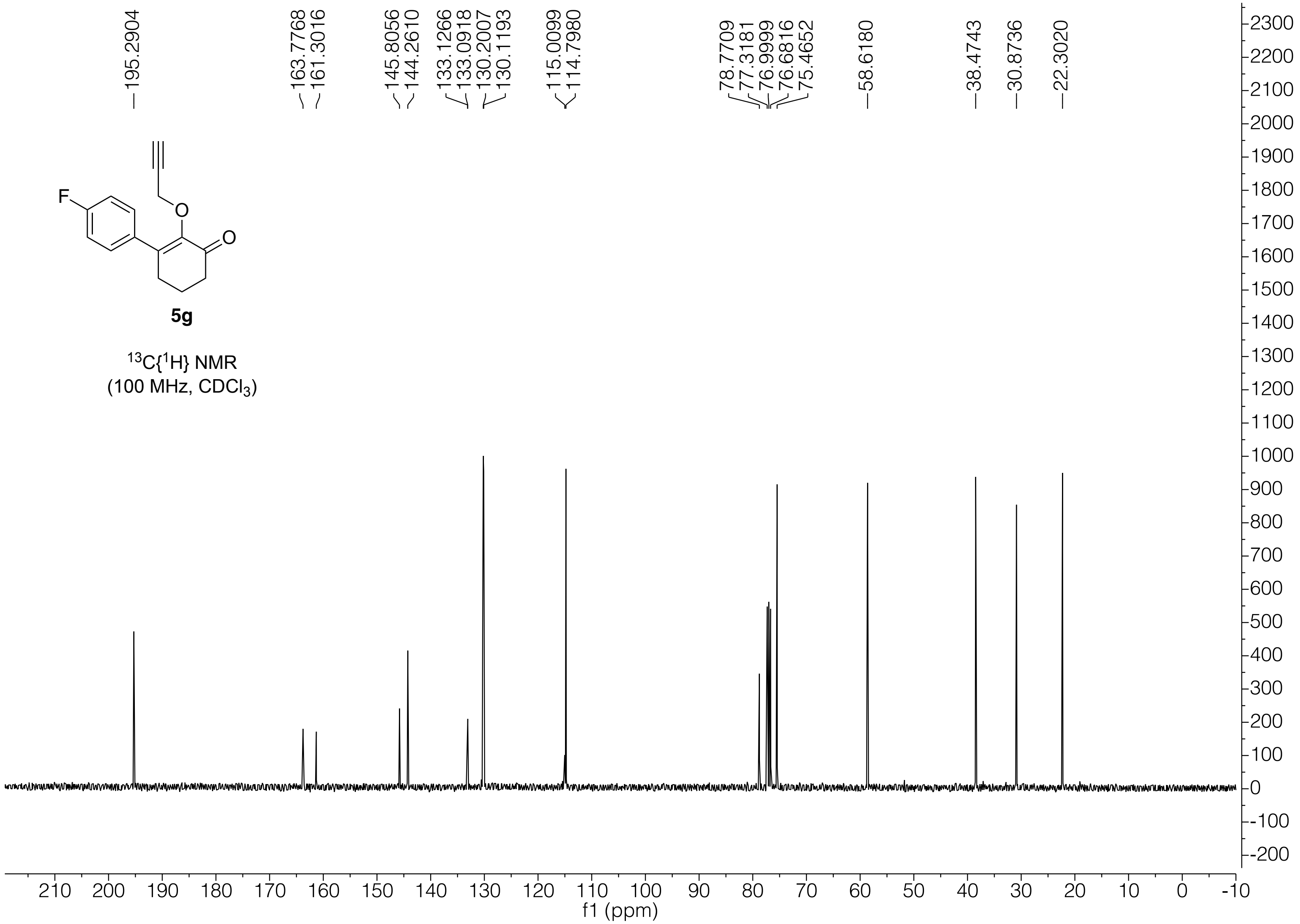
195.2904
163.7768
161.3016
145.8056
144.2610
133.1266
133.0918
130.2007
130.1193
115.0099
114.7980
78.7709
77.3181
76.9999
76.6816
75.4652
58.6180
38.4743
30.8736
22.3020
F
O
O
5g
13C{1H} NMR
(100 MHz, CDCl3)
f1 (ppm)

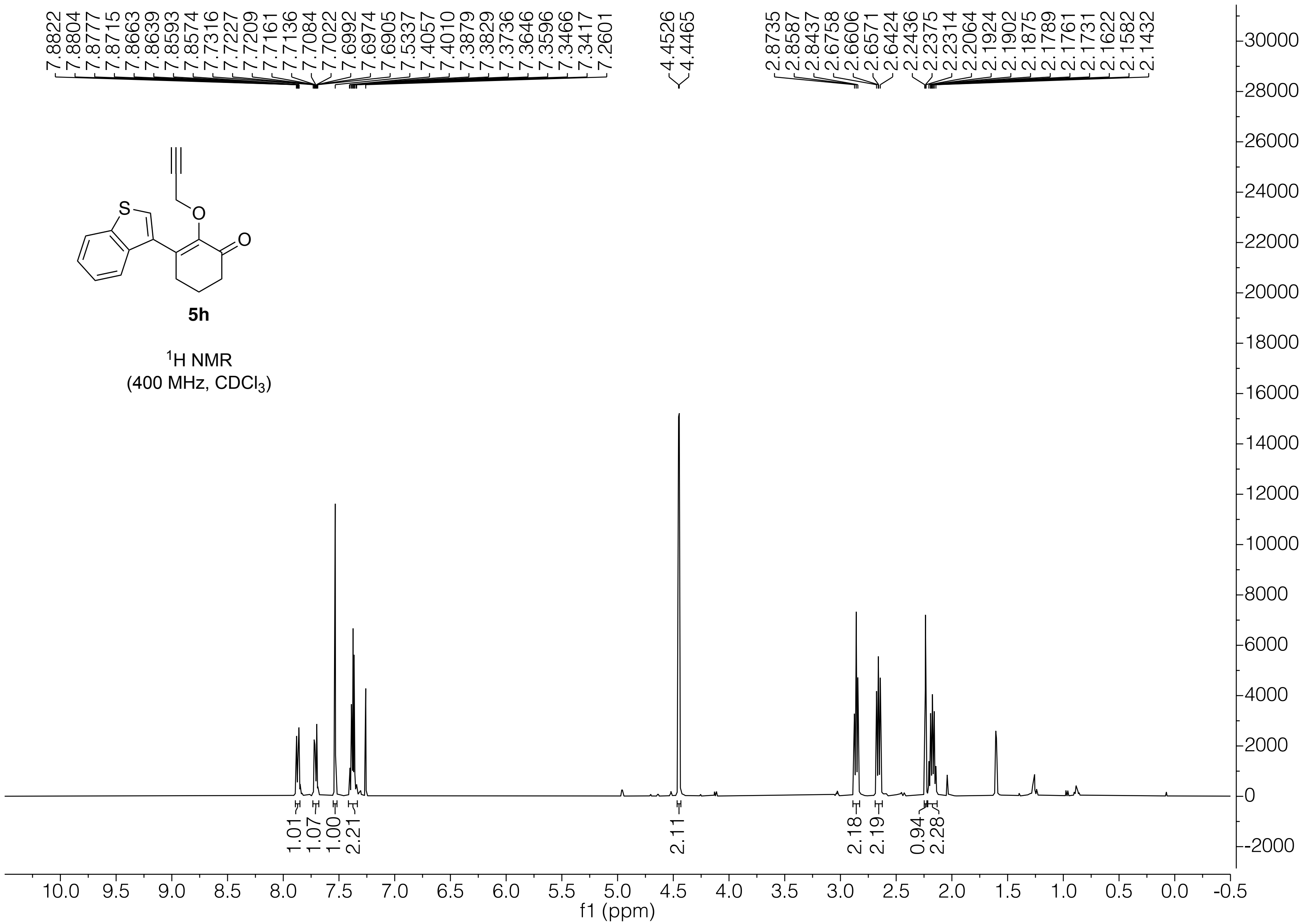
7.8822
7.8804
7.8777
7.8715
7.8663
7.8639
7.8593
7.8574
7.7316
7.7227
7.7209
7.7161
7.7136
7.7084
7.7022
7.6992
7.6974
7.6905
7.5337
7.4057
7.4010
7.3879
7.3829
7.3736
7.3646
7.3596
7.3466
7.3417
7.2601
4.4526
4.4465
2.8735
2.8587
2.8437
2.6758
2.6606
2.6571
2.6424
2.2436
2.2375
2.2314
2.2064
2.1924
2.1902
2.1875
2.1789
2.1761
2.1731
2.1622
2.1582
2.1432
S
O
O
5h
^{1}H NMR
(400 MHz, $CDCl_3$)
1.01
1.07
1.00
2.21
2.11
2.18
2.19
0.94
2.28
f1 (ppm)

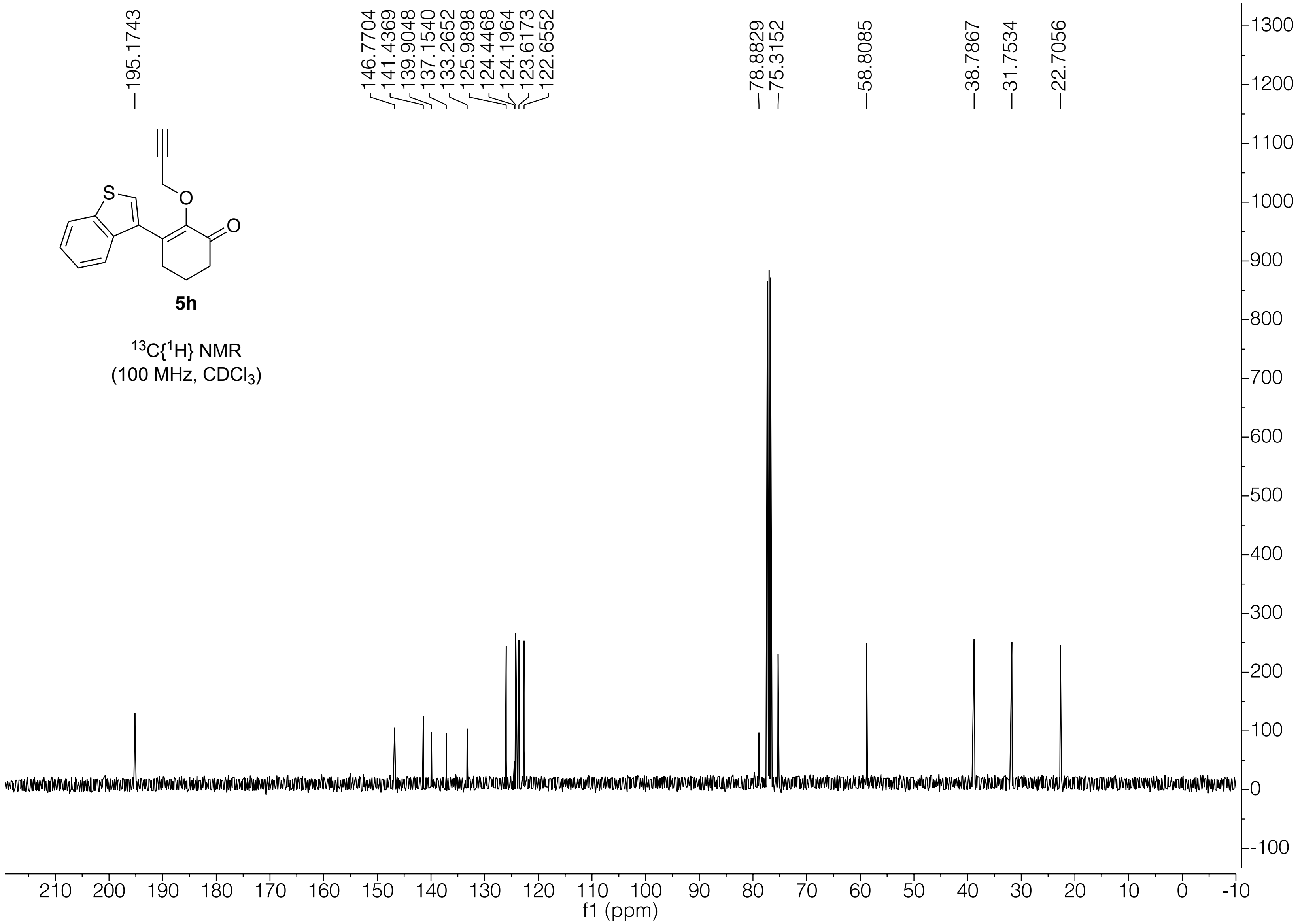
195.1743
146.7704
141.4369
139.9048
137.1540
133.2652
125.9898
124.4468
124.1964
123.6173
122.6552
78.8829
75.3152
58.8085
38.7867
31.7534
22.7056
S
O
O
5h
$^{13}C\{^{1}H\}$ NMR
(100 MHz, $CDCl_3$)
f1 (ppm)

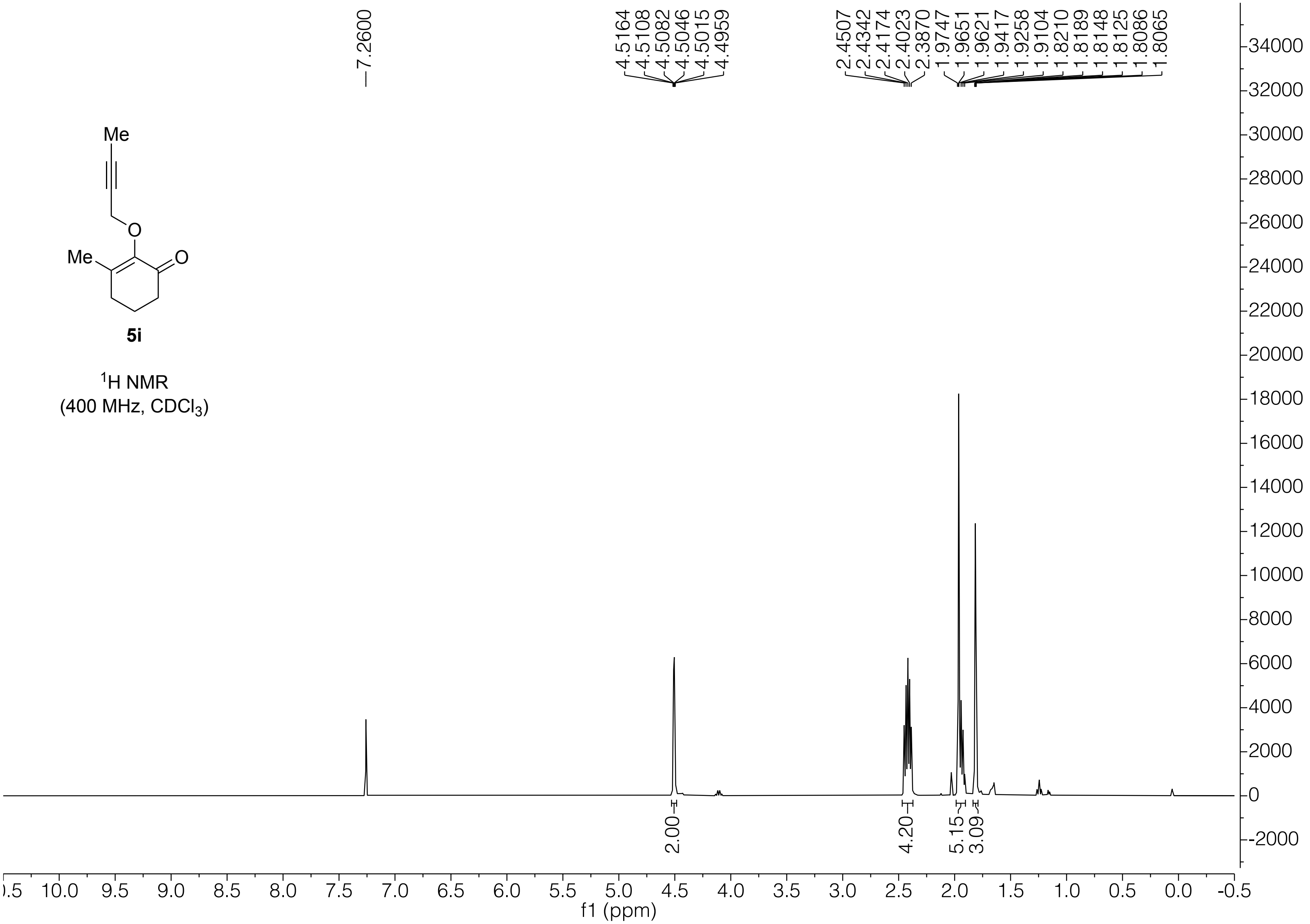

Me
O
Me
O
5i
^{1}H NMR
(400 MHz, CDCl$_3$)
7.2600
4.5164
4.5108
4.5082
4.5046
4.5015
4.4959
2.4507
2.4342
2.4174
2.4023
2.3870
1.9747
1.9651
1.9621
1.9417
1.9258
1.9104
1.8210
1.8189
1.8148
1.8125
1.8086
1.8065
2.00
4.20
5.15
3.09
f1 (ppm)

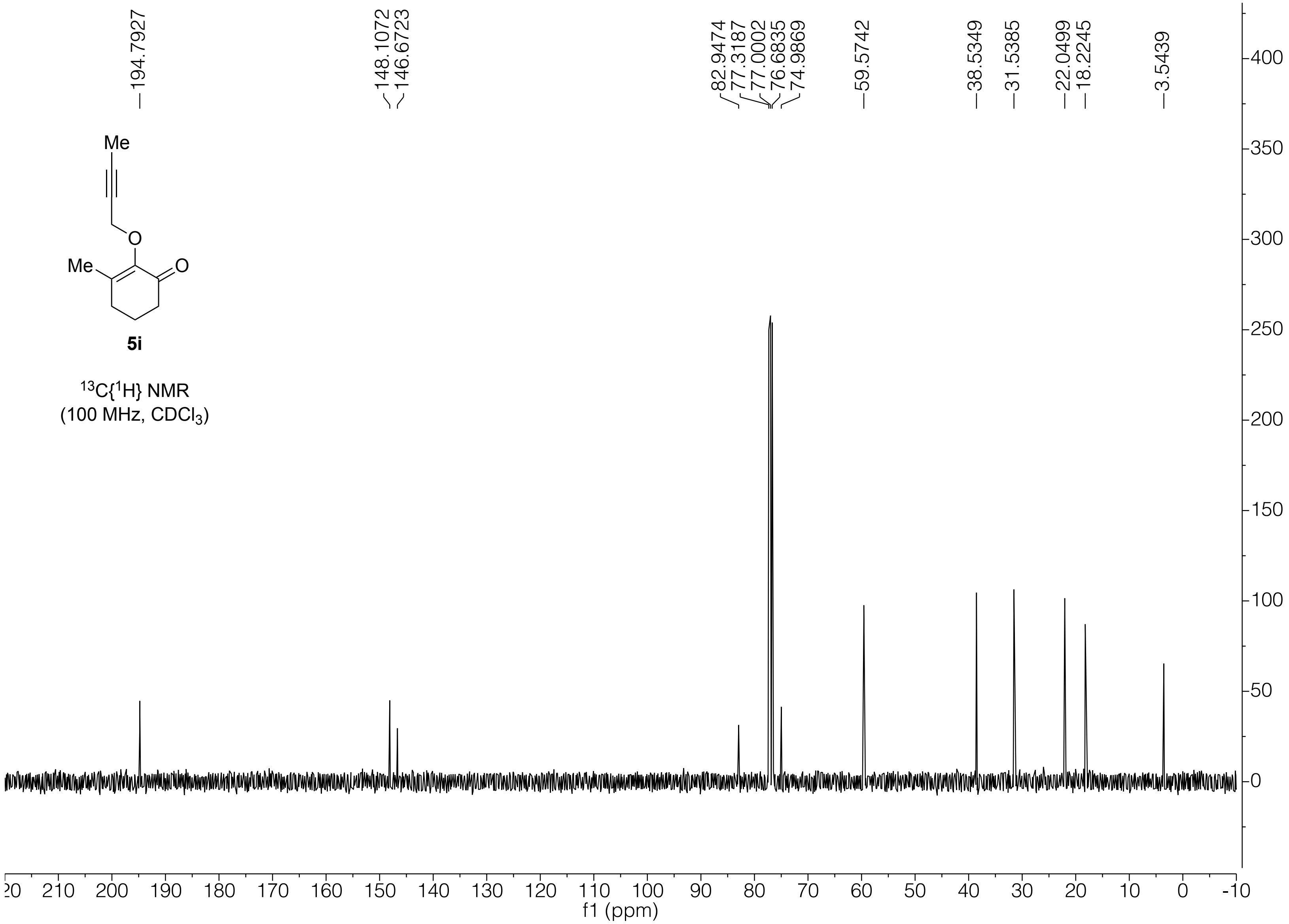

194.7927
148.1072
146.6723
82.9474
77.3187
77.0002
76.6835
74.9869
59.5742
38.5349
31.5385
22.0499
18.2245
3.5439
Me
O
Me
O
5i
$^{13}C\{^1H\}$ NMR
(100 MHz, $CDCl_3$)
f1 (ppm)

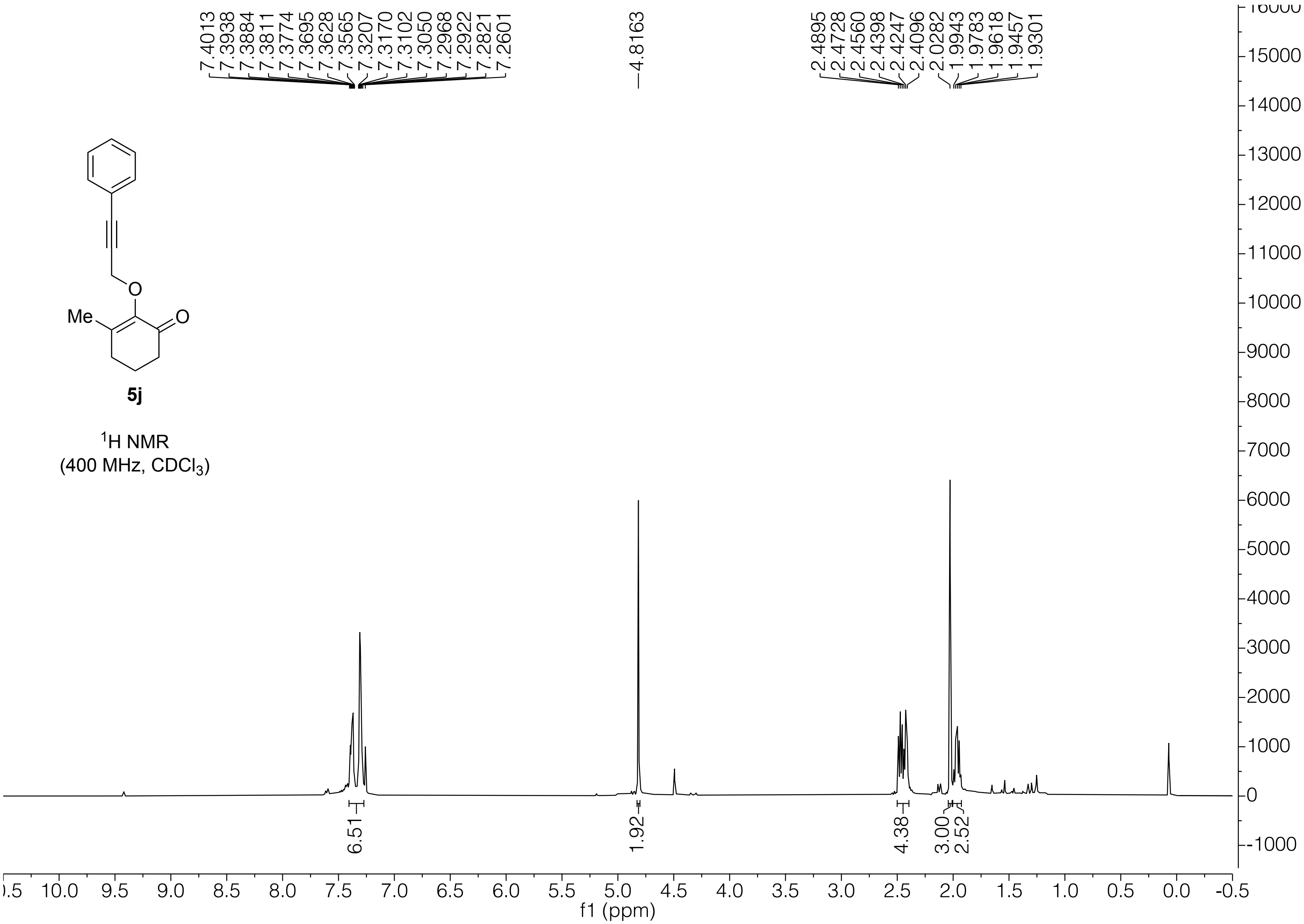

Me
O
O
5j
1H NMR
(400 MHz, CDCl3)
7.4013
7.3938
7.3884
7.3811
7.3774
7.3695
7.3628
7.3565
7.3207
7.3170
7.3102
7.3050
7.2968
7.2922
7.2821
7.2601
4.8163
2.4895
2.4728
2.4560
2.4398
2.4247
2.4096
2.0282
1.9943
1.9783
1.9618
1.9457
1.9301
6.51
1.92
4.38
3.00
2.52
f1 (ppm)

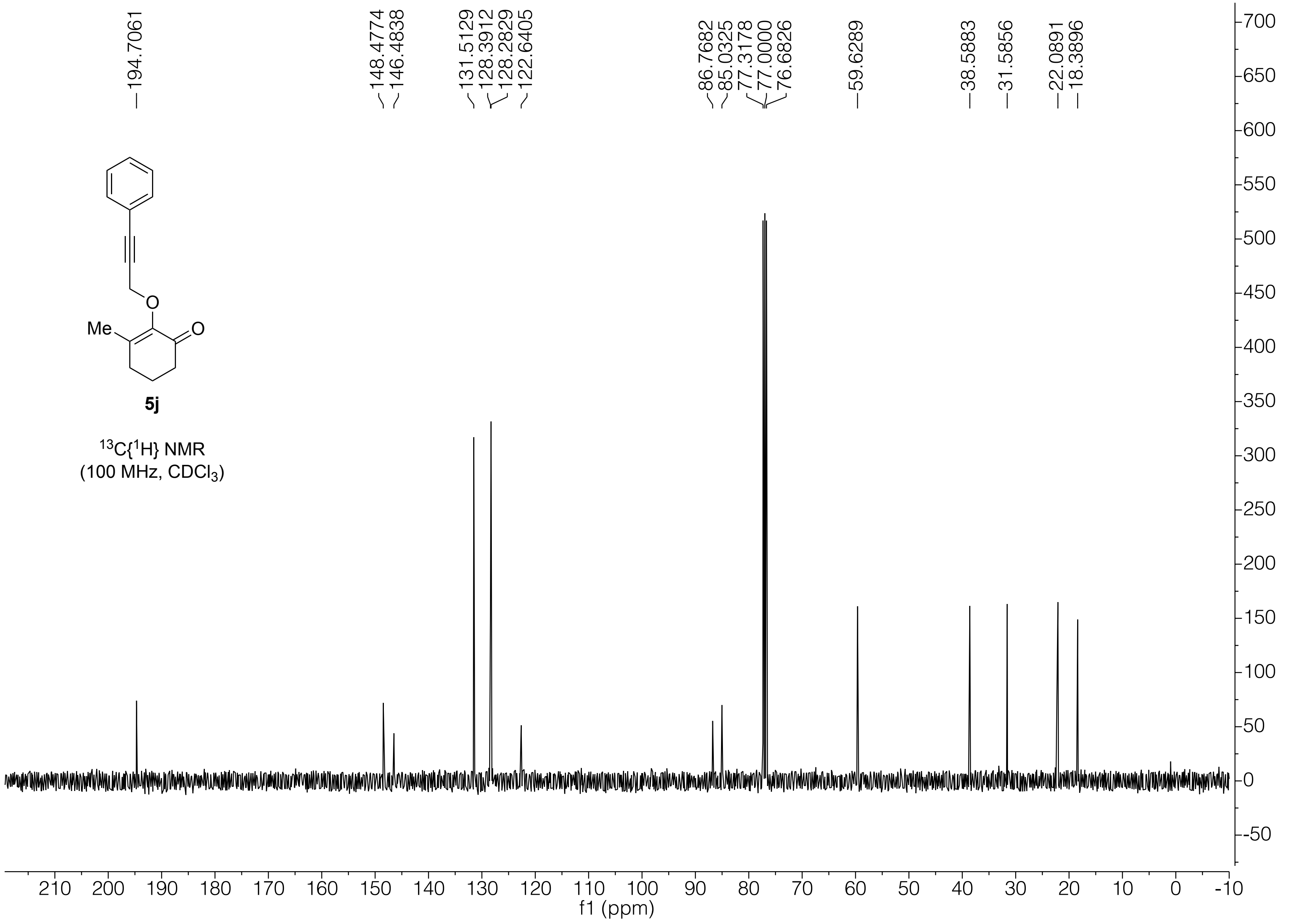
194.7061
148.4774
146.4838
131.5129
128.3912
128.2829
122.6405
86.7682
85.0325
77.3178
77.0000
76.6826
59.6289
38.5883
31.5856
22.0891
18.3896
Me
O
O
5j
13C{1H} NMR
(100 MHz, CDCl3)
210 200 190 180 170 160 150 140 130 120 110 100 90 80 70 60 50 40 30 20 10 0 -10
f1 (ppm)
700 650 600 550 500 450 400 350 300 250 200 150 100 50 0 -50

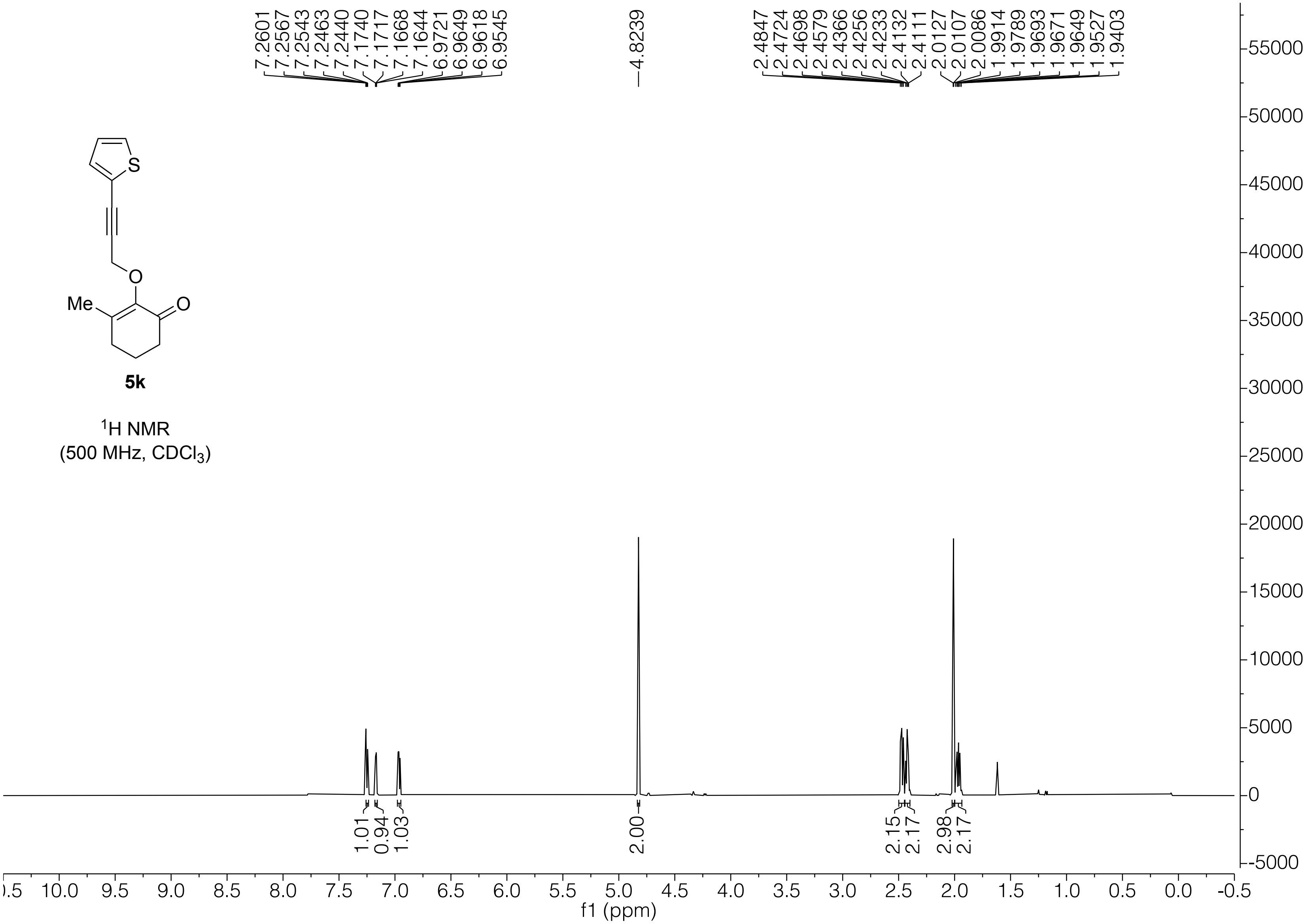

7.2601
7.2567
7.2543
7.2463
7.2440
7.1740
7.1717
7.1668
7.1644
6.9721
6.9649
6.9618
6.9545
4.8239
2.4847
2.4724
2.4698
2.4579
2.4366
2.4256
2.4233
2.4132
2.4111
2.0127
2.0107
2.0086
1.9914
1.9789
1.9693
1.9671
1.9649
1.9527
1.9403
S
O
Me
O
5k
¹H NMR
(500 MHz, CDCl3)
1.01
0.94
1.03
2.00
2.15
2.17
2.98
2.17
f1 (ppm)

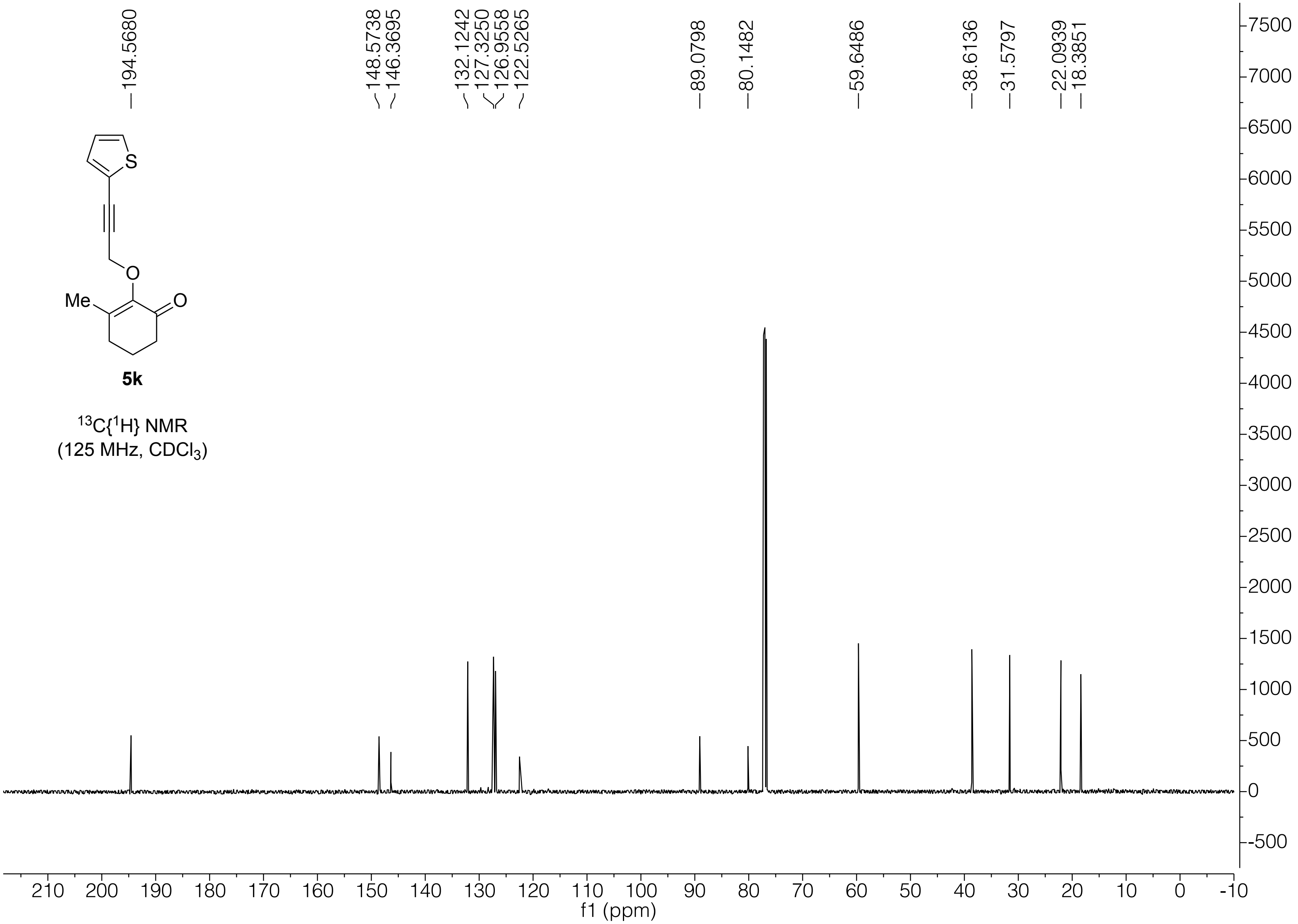

194.5680
148.5738
146.3695
132.1242
127.3250
126.9558
122.5265
89.0798
80.1482
59.6486
38.6136
31.5797
22.0939
18.3851
S
O
Me
O
5k
$^{13}C\{^1H\}$ NMR
(125 MHz, $CDCl_3$)
7500
7000
6500
6000
5500
5000
4500
4000
3500
3000
2500
2000
1500
1000
500
0
-500
210 200 190 180 170 160 150 140 130 120 110 100 90 80 70 60 50 40 30 20 10 0 -10
f1 (ppm)

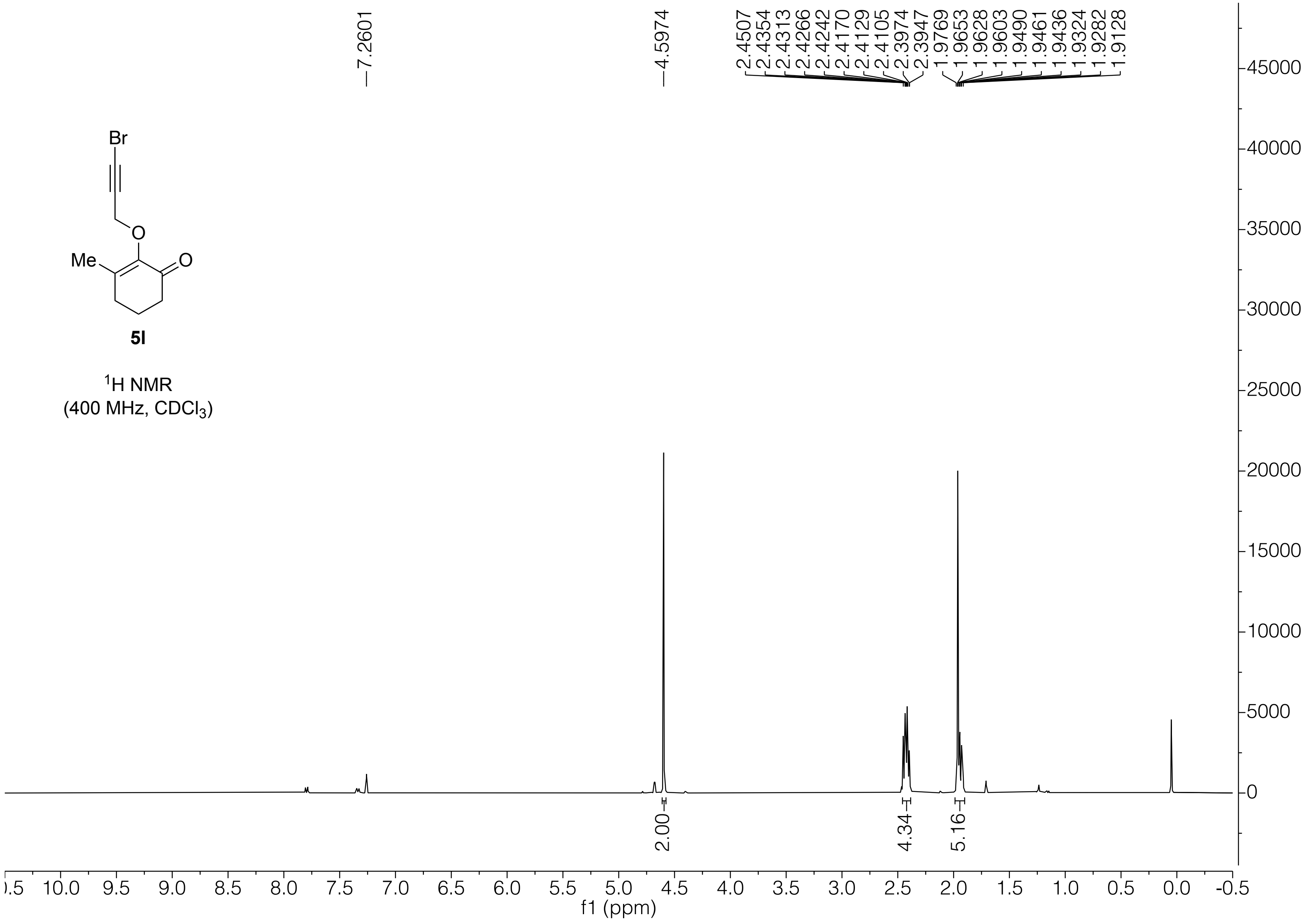

Br
O
Me
O
5l
1H NMR
(400 MHz, CDCl3)
7.2601
4.5974
2.4507
2.4354
2.4313
2.4266
2.4242
2.4170
2.4129
2.4105
2.3974
2.3947
1.9769
1.9653
1.9628
1.9603
1.9490
1.9461
1.9436
1.9324
1.9282
1.9128
2.00
4.34
5.16
45000
40000
35000
30000
25000
20000
15000
10000
5000
0
10.0
9.5
9.0
8.5
8.0
7.5
7.0
6.5
6.0
5.5
5.0
4.5
4.0
3.5
3.0
2.5
2.0
1.5
1.0
0.5
0.0
-0.5
f1 (ppm)

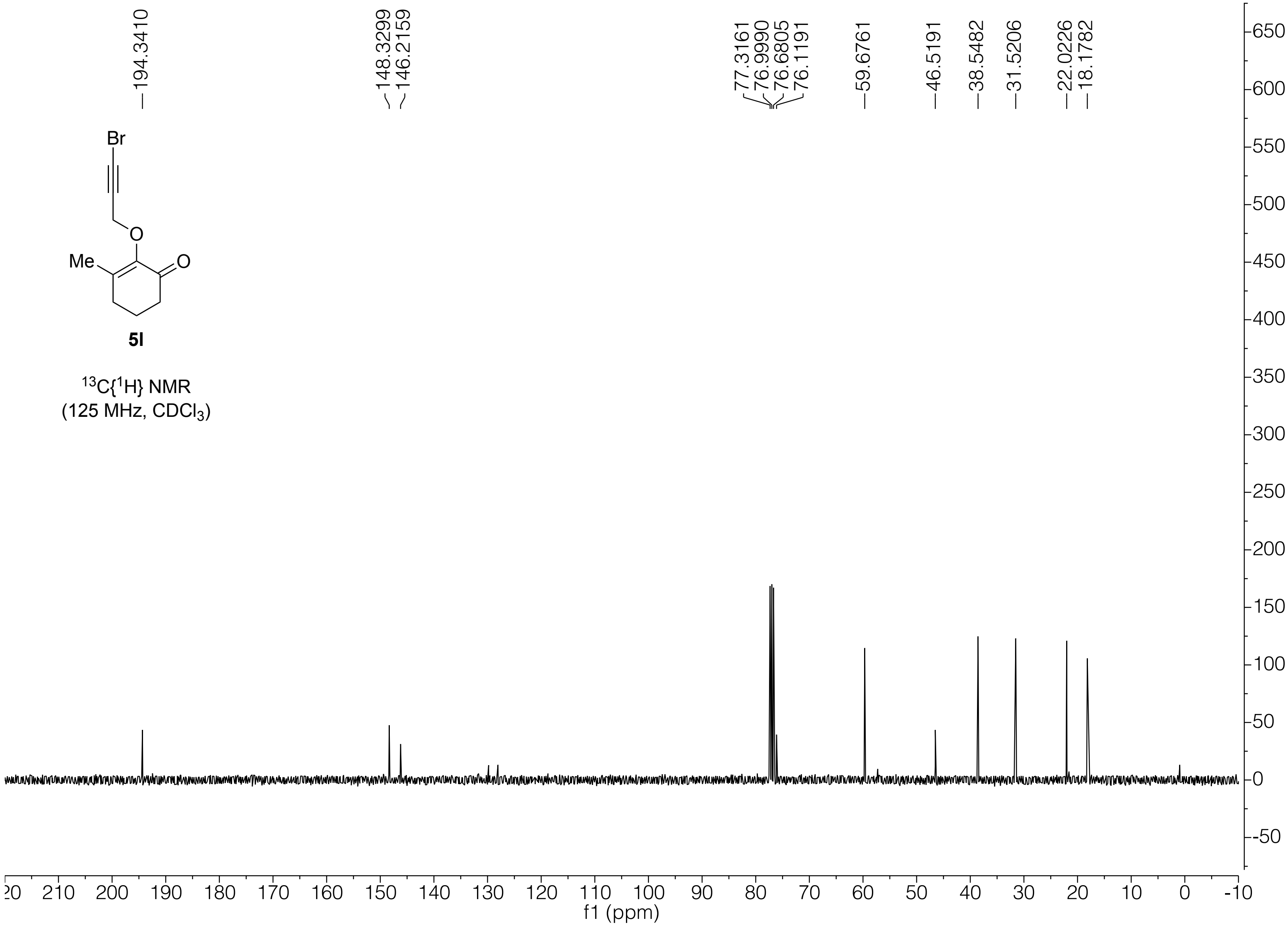
194.3410
148.3299
146.2159
77.3161
76.9990
76.6805
76.1191
59.6761
46.5191
38.5482
31.5206
22.0226
18.1782
Br
O
Me
O
5l
$^{13}C\{^1H\}$ NMR
(125 MHz, $CDCl_3$)
650
600
550
500
450
400
350
300
250
200
150
100
50
0
-50
20
210
200
190
180
170
160
150
140
130
120
110
100
90
80
70
60
50
40
30
20
10
0
-10
f1 (ppm)

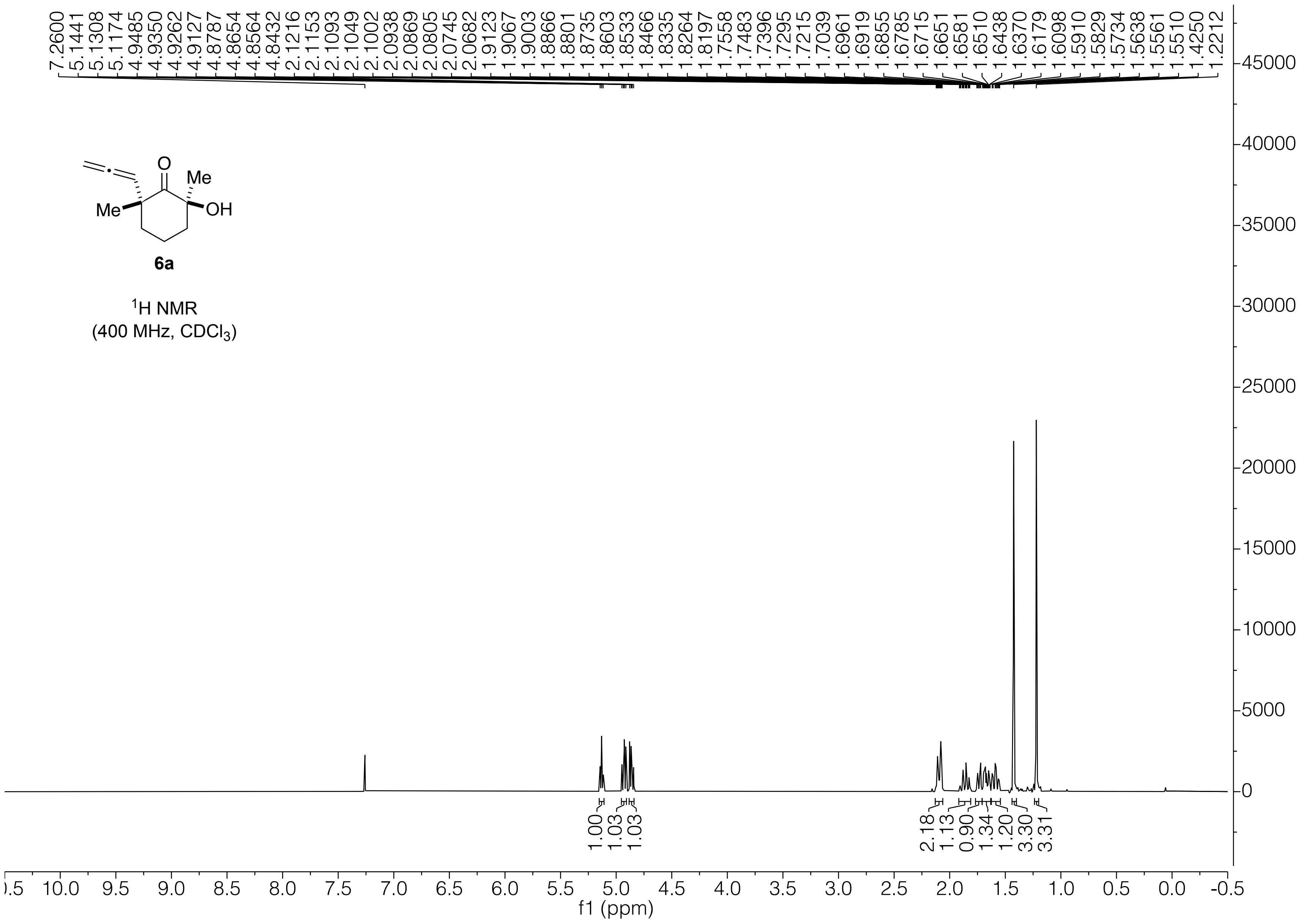
6a
^{1}H NMR
(400 MHz, $CDCl_3$)
f1 (ppm)

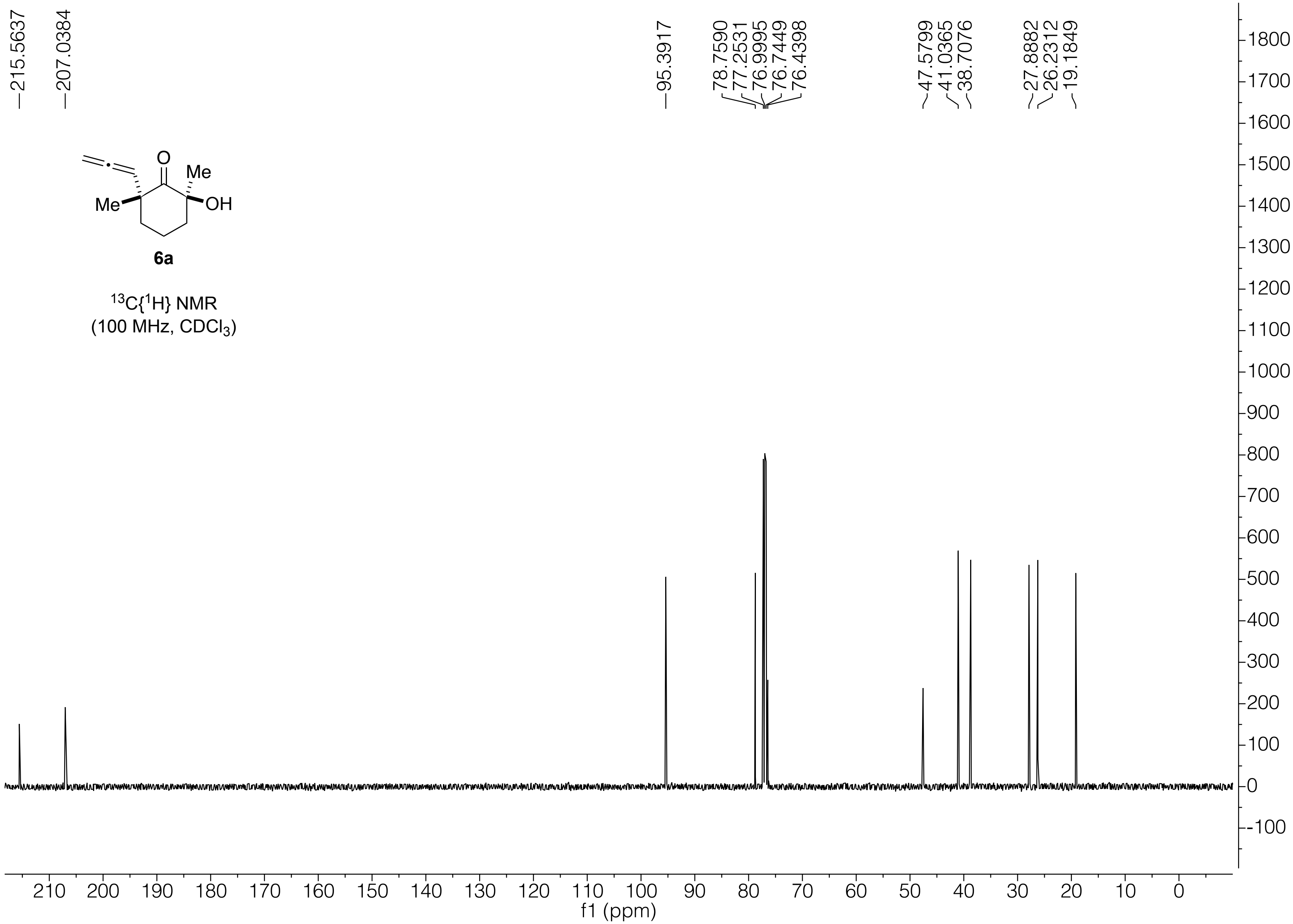

215.5637
207.0384
95.3917
78.7590
77.2531
76.9995
76.7449
76.4398
47.5799
41.0365
38.7076
27.8882
26.2312
19.1849
O
Me
Me
OH
6a
$^{13}C\{^{1}H\}$ NMR
(100 MHz, $CDCl_3$)
f1 (ppm)

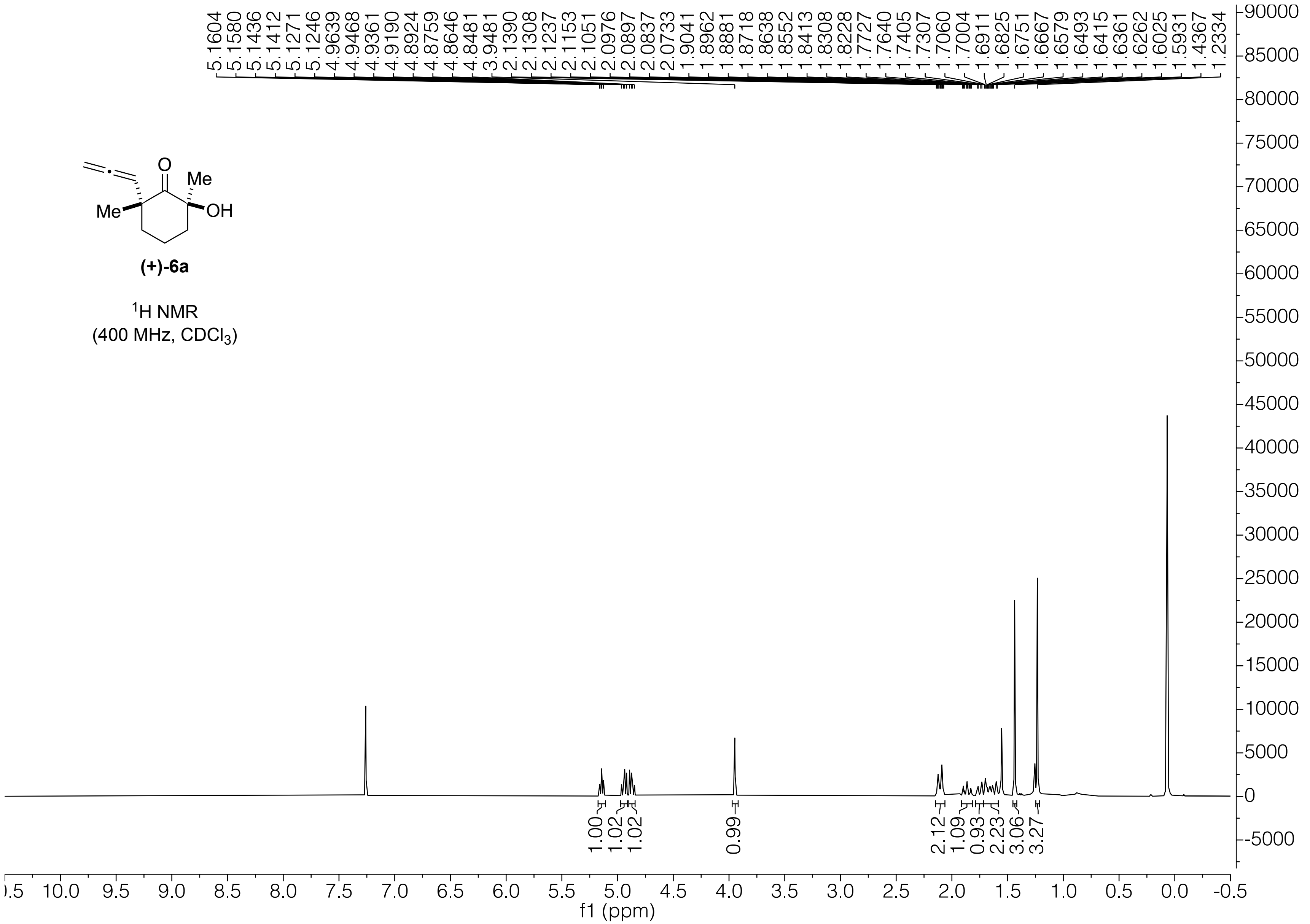

(+)-6a

^1H NMR
(400 MHz, $CDCl_3$)

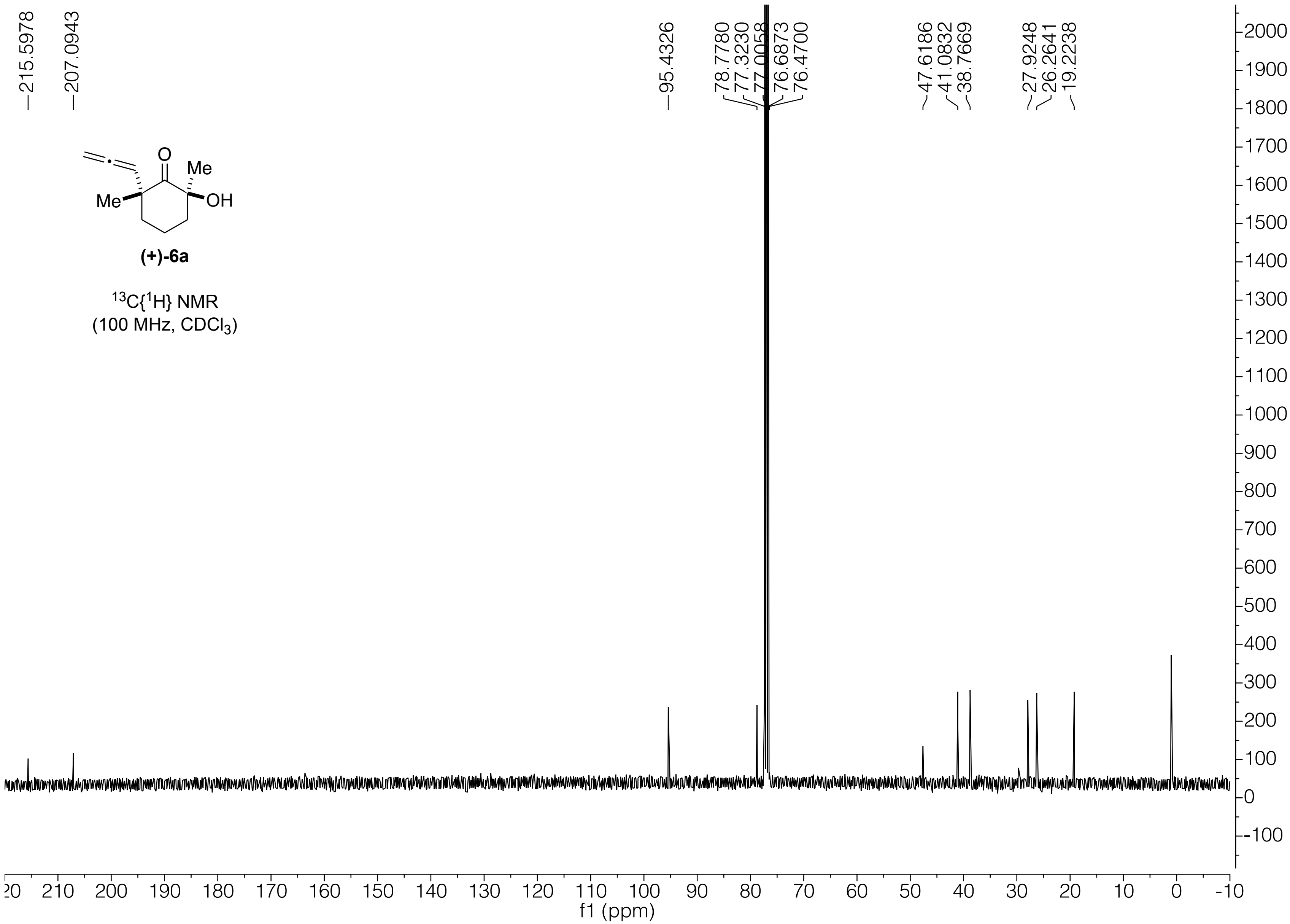

215.5978
207.0943
95.4326
78.7780
77.3230
77.0058
76.6873
76.4700
47.6186
41.0832
38.7669
27.9248
26.2641
19.2238
O
Me
Me
OH
(+)-6a
13C{1H} NMR
(100 MHz, CDCl3)
f1 (ppm)

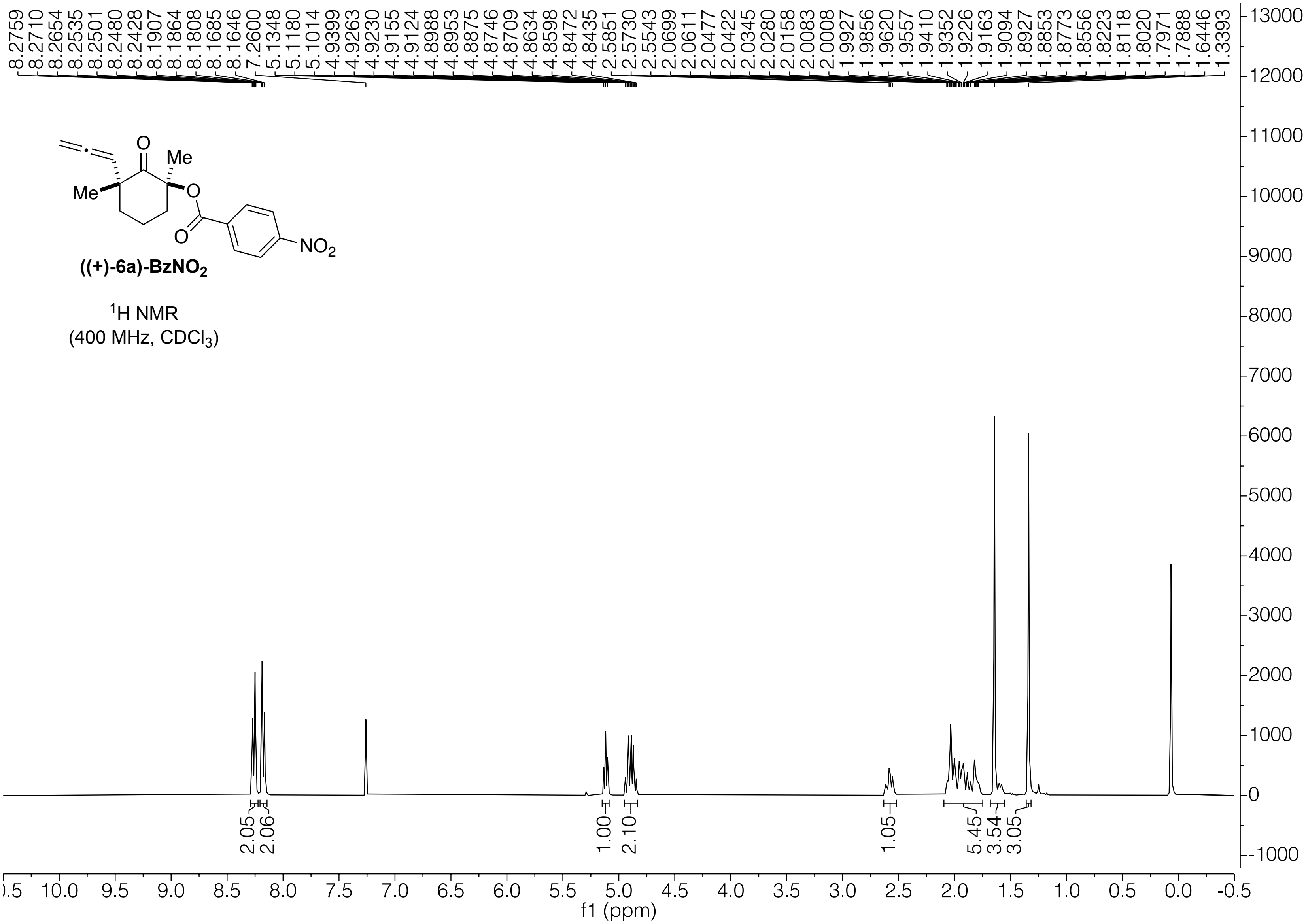
8.2759
8.2710
8.2654
8.2535
8.2501
8.2480
8.2428
8.1907
8.1864
8.1808
8.1685
8.1646
7.2600
5.1348
5.1180
5.1014
4.9399
4.9263
4.9230
4.9155
4.9124
4.8988
4.8953
4.8875
4.8746
4.8709
4.8634
4.8598
4.8472
4.8435
2.5851
2.5730
2.5543
2.0699
2.0611
2.0477
2.0422
2.0345
2.0280
2.0158
2.0083
2.0008
1.9927
1.9856
1.9620
1.9557
1.9410
1.9352
1.9226
1.9163
1.9094
1.8927
1.8853
1.8773
1.8556
1.8223
1.8118
1.8020
1.7971
1.7888
1.6446
1.3393
O
Me
Me
O
O
NO2
((+)-6a)-BzNO2
1H NMR
(400 MHz, CDCl3)
2.05
2.06
1.00
2.10
1.05
5.45
3.54
3.05
f1 (ppm)

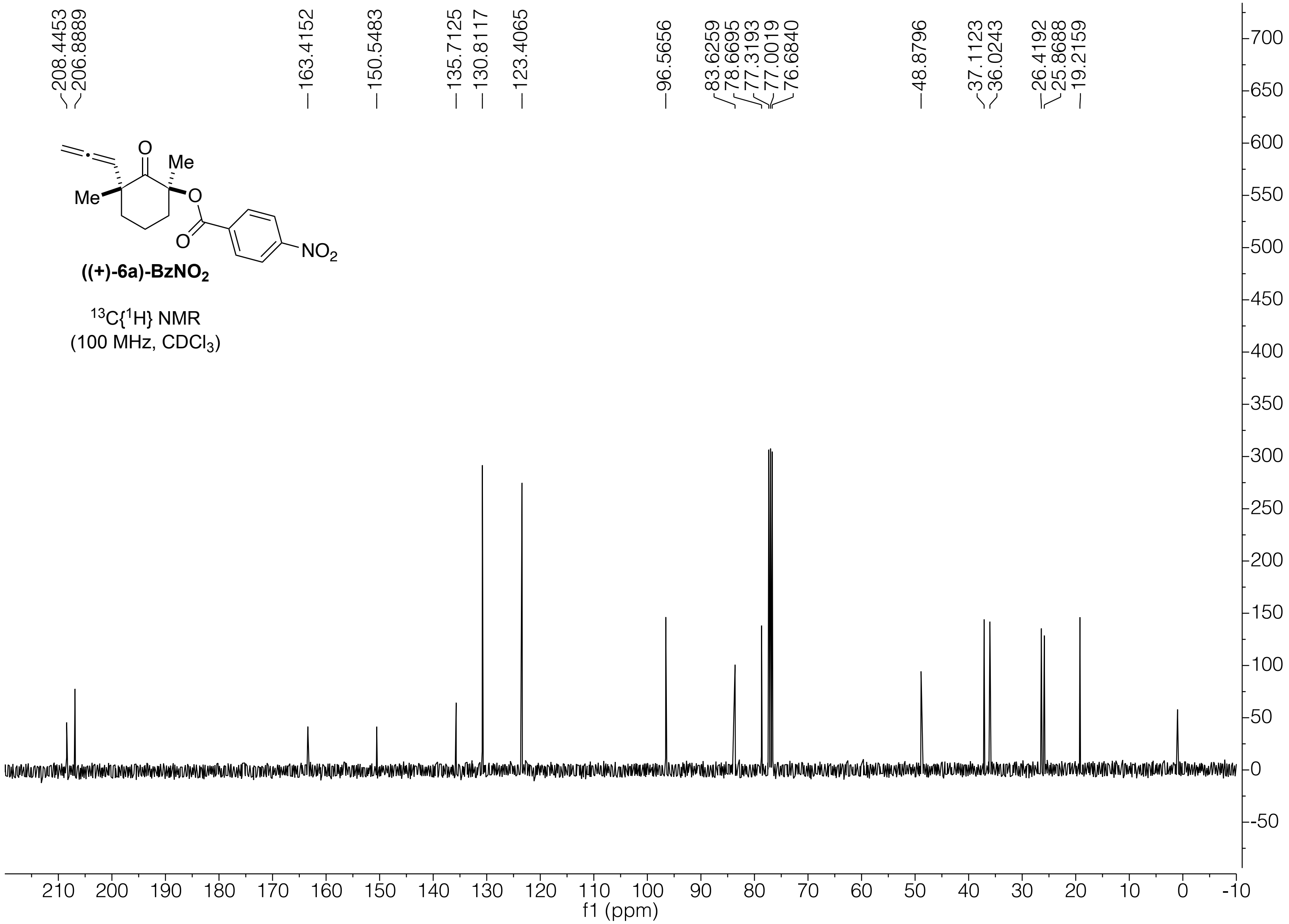
208.4453
206.8889
163.4152
150.5483
135.7125
130.8117
123.4065
96.5656
83.6259
78.6695
77.3193
77.0019
76.6840
48.8796
37.1123
36.0243
26.4192
25.8688
19.2159
O
Me
Me
O
O
NO_2
((+)-6a)-$BzNO_2$
$^{13}C\{^1H\}$ NMR
(100 MHz, $CDCl_3$)
700
650
600
550
500
450
400
350
300
250
200
150
100
50
0
-50
210 200 190 180 170 160 150 140 130 120 110 100 90 80 70 60 50 40 30 20 10 0 -10
f1 (ppm)

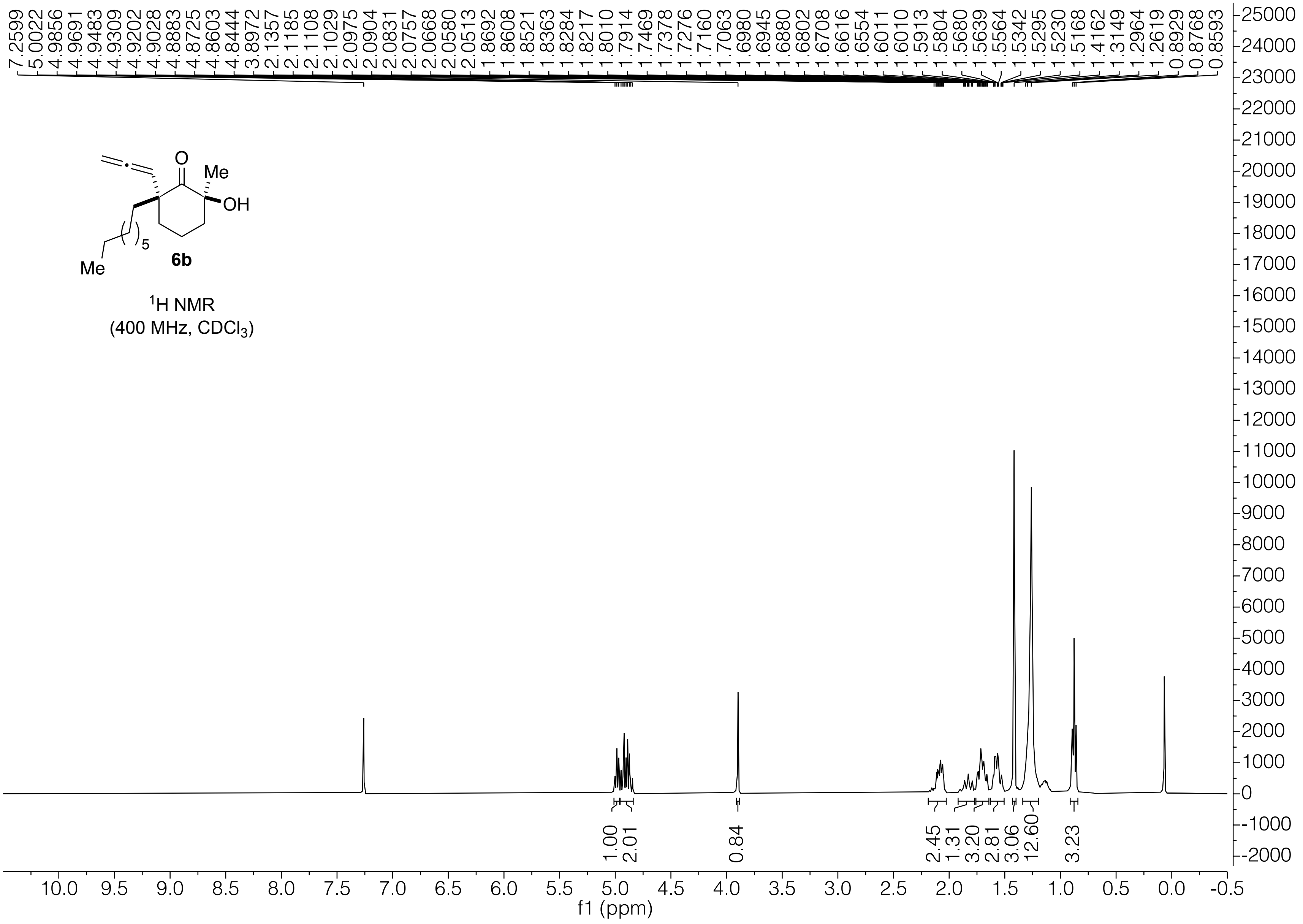

6b
¹H NMR
(400 MHz, CDCl3)
f1 (ppm)

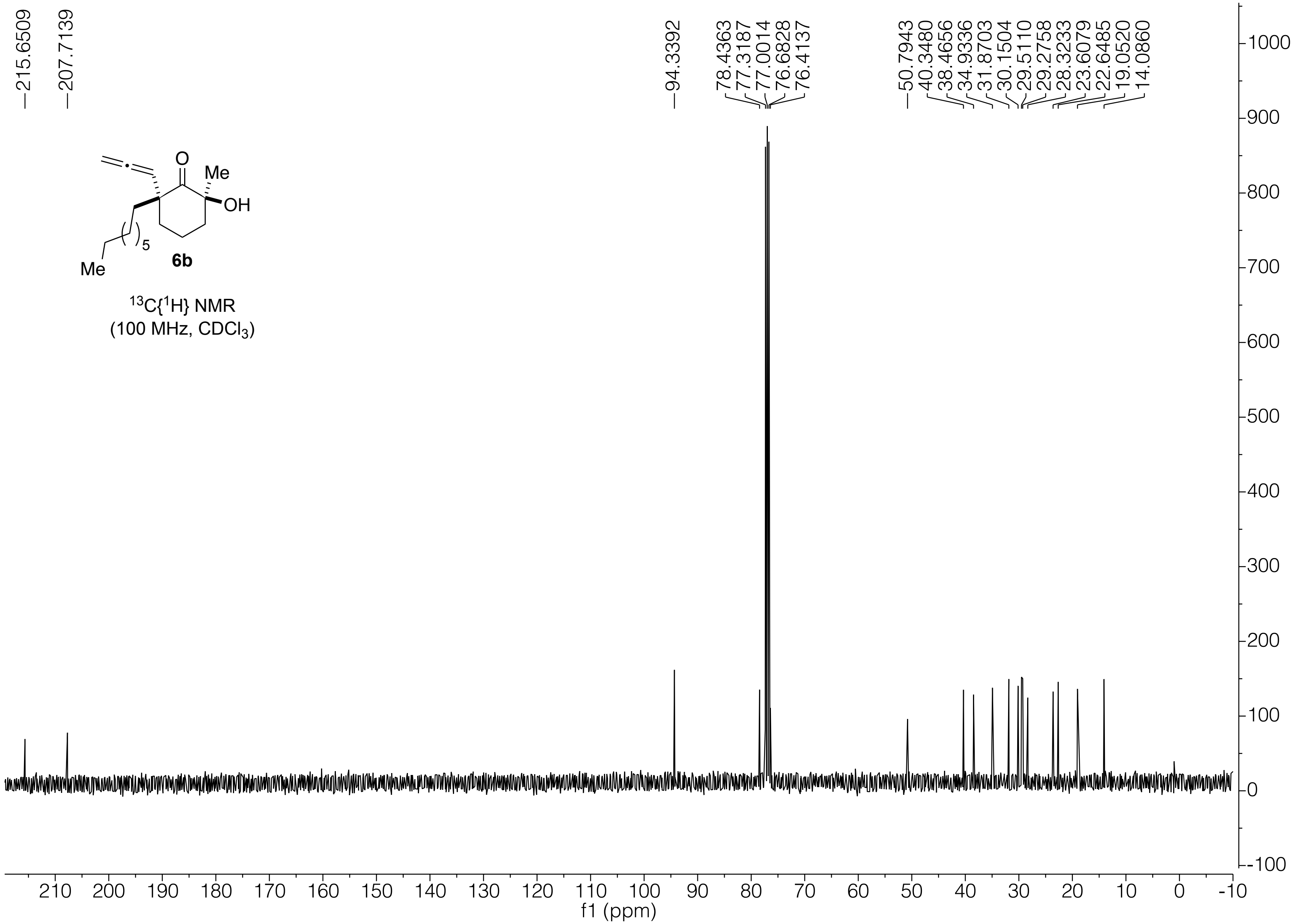
215.6509
207.7139
94.3392
78.4363
77.3187
77.0014
76.6828
76.4137
50.7943
40.3480
38.4656
34.9336
31.8703
30.1504
29.5110
29.2758
28.3233
23.6079
22.6485
19.0520
14.0860
O
Me
OH
5
Me
6b
13C{1H} NMR
(100 MHz, CDCl3)
f1 (ppm)

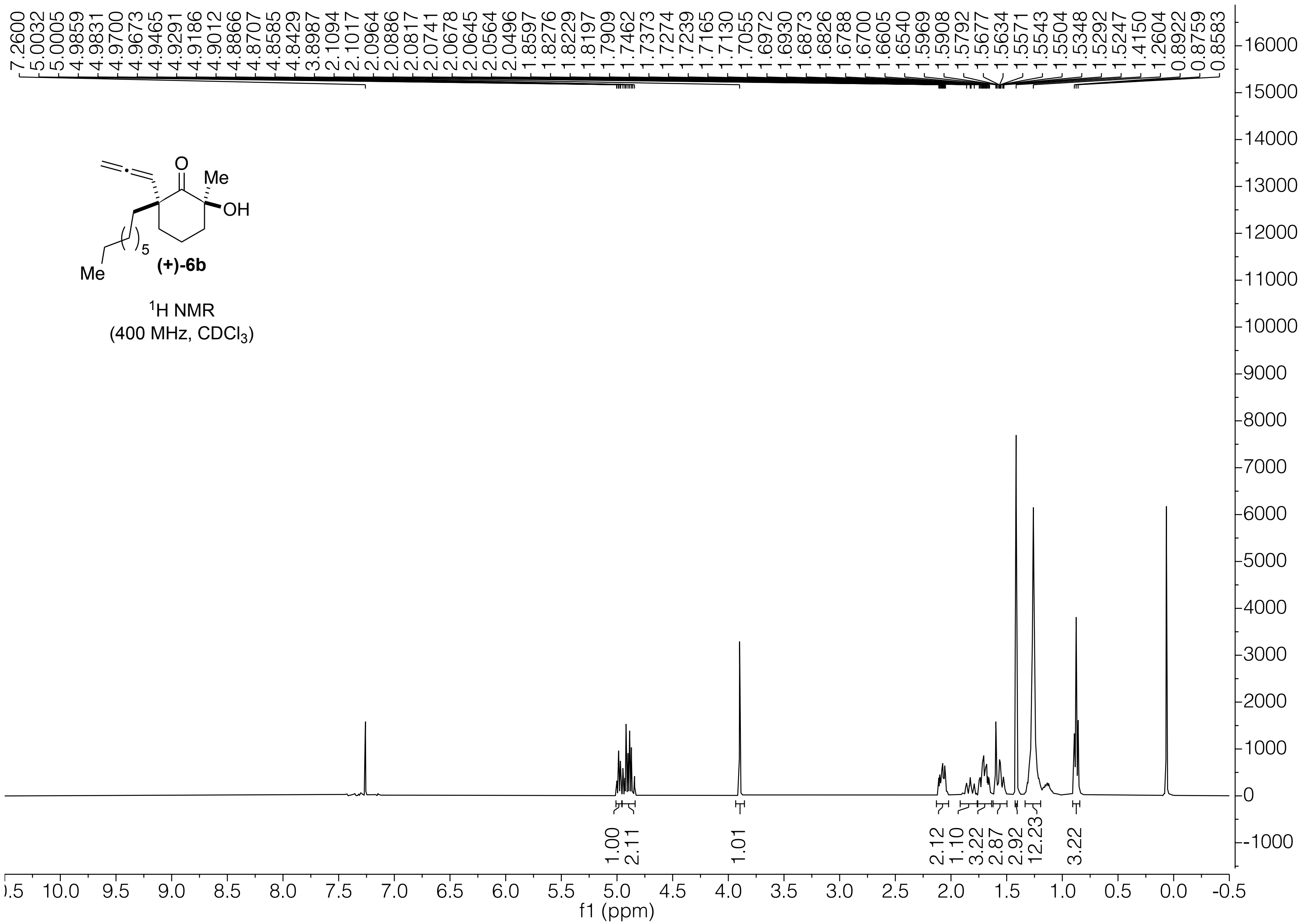

7.2600
5.0032
5.0005
4.9859
4.9831
4.9700
4.9673
4.9465
4.9291
4.9186
4.9012
4.8866
4.8707
4.8585
4.8429
3.8987
2.1094
2.1017
2.0964
2.0886
2.0817
2.0741
2.0678
2.0645
2.0564
2.0496
1.8597
1.8276
1.8229
1.8197
1.7909
1.7462
1.7373
1.7274
1.7239
1.7165
1.7130
1.7055
1.6972
1.6930
1.6873
1.6826
1.6788
1.6700
1.6605
1.6540
1.5969
1.5908
1.5792
1.5677
1.5634
1.5571
1.5543
1.5504
1.5348
1.5292
1.5247
1.4150
1.2604
0.8922
0.8759
0.8583
(+)-6b
^{1}H NMR
(400 MHz, $CDCl_3$)
1.00
2.11
1.01
2.12
1.10
3.22
2.87
2.92
12.23
3.22
f1 (ppm)

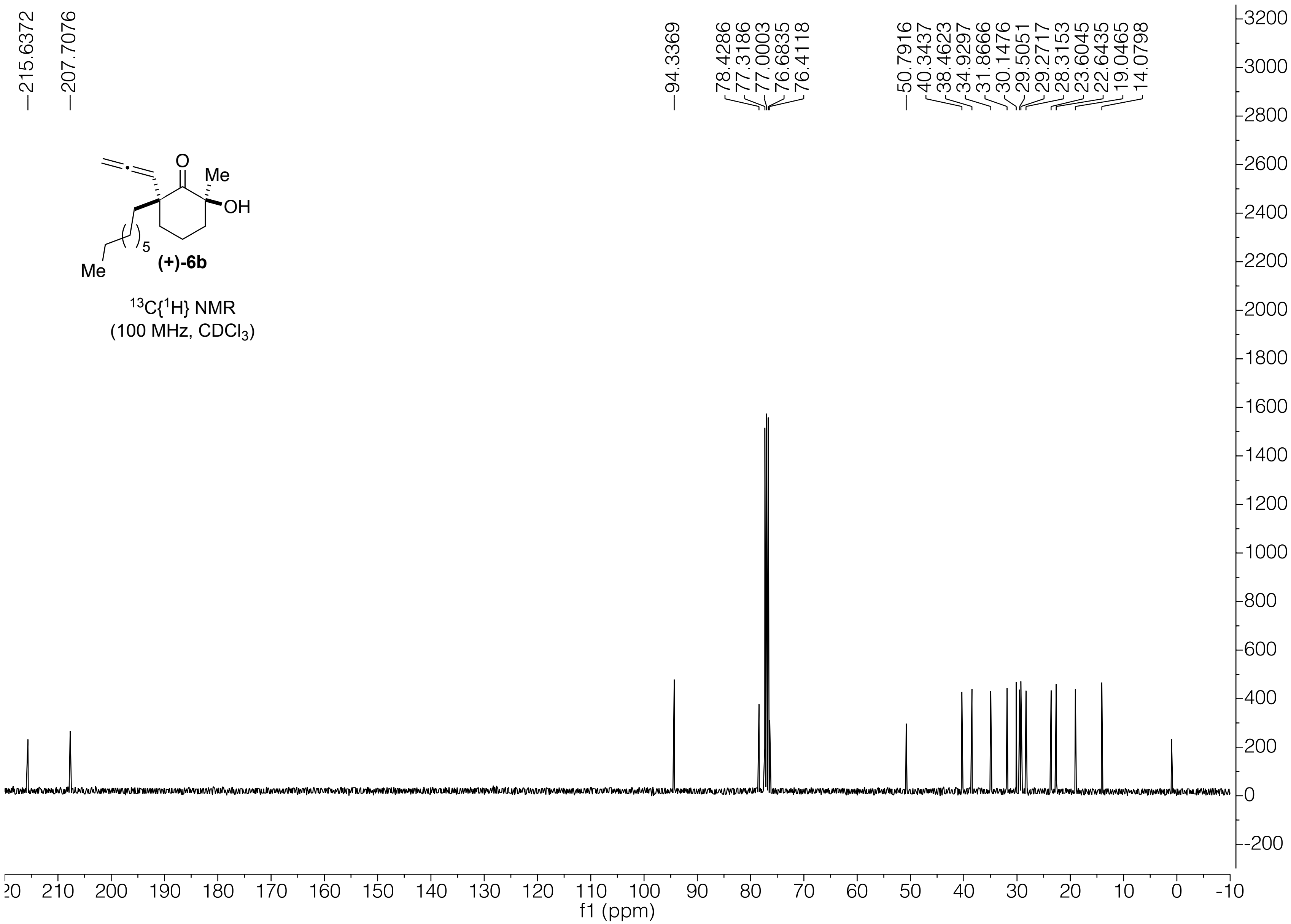

215.6372
207.7076
94.3369
78.4286
77.3186
77.0003
76.6835
76.4118
50.7916
40.3437
38.4623
34.9297
31.8666
30.1476
29.5051
29.2717
28.3153
23.6045
22.6435
19.0465
14.0798
O
Me
OH
Me
5
(+)-6b
$^{13}C\{^1H\}$ NMR
(100 MHz, $CDCl_3$)
f1 (ppm)

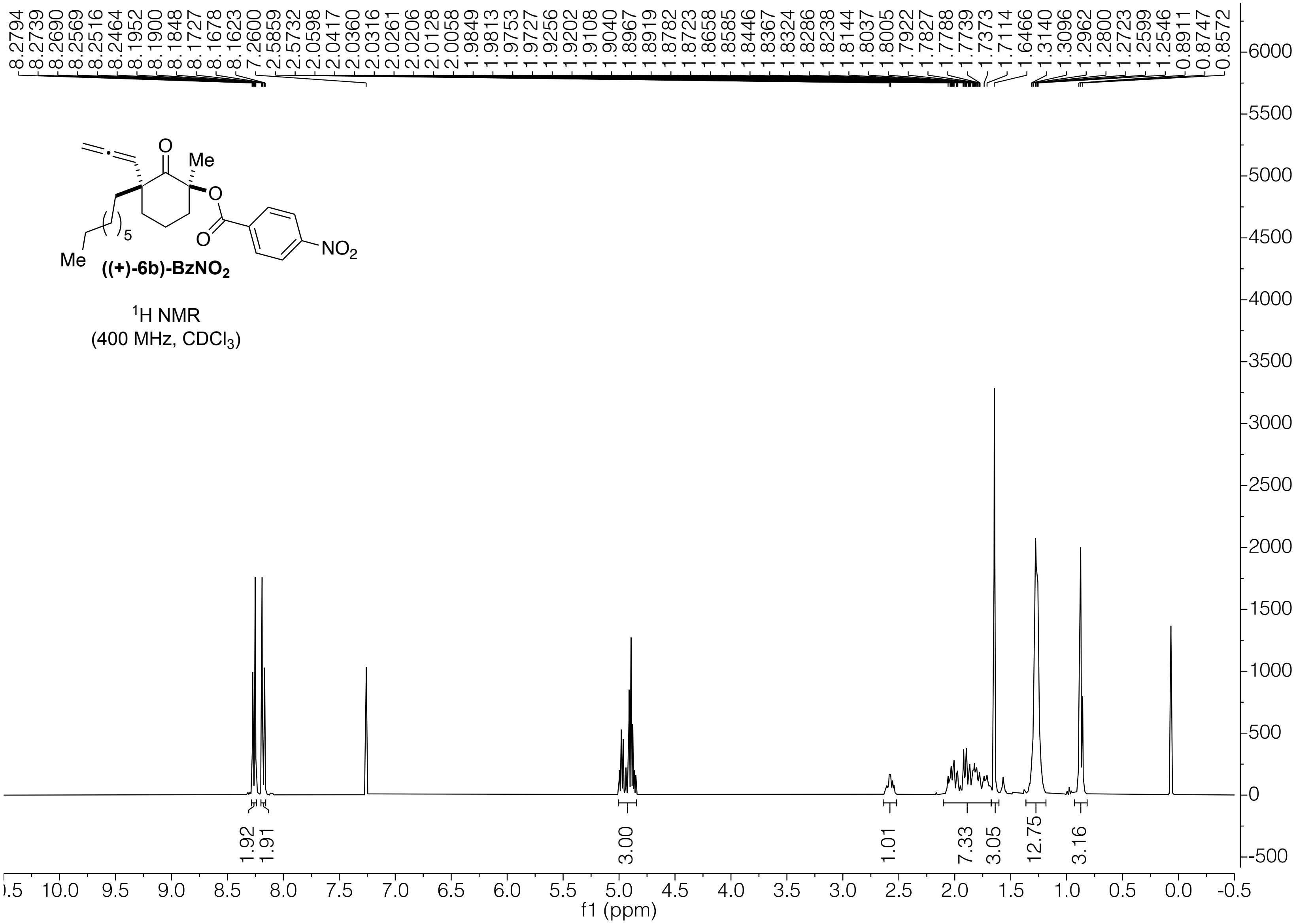
8.2794
8.2739
8.2690
8.2569
8.2516
8.2464
8.1952
8.1900
8.1848
8.1727
8.1678
8.1623
7.2600
2.5859
2.5732
2.0598
2.0417
2.0360
2.0316
2.0261
2.0206
2.0128
2.0058
1.9849
1.9813
1.9753
1.9727
1.9256
1.9202
1.9108
1.9040
1.8967
1.8919
1.8782
1.8723
1.8658
1.8585
1.8446
1.8367
1.8324
1.8286
1.8238
1.8144
1.8037
1.8005
1.7922
1.7827
1.7788
1.7739
1.7373
1.7114
1.6466
1.3140
1.3096
1.2962
1.2800
1.2723
1.2599
1.2546
0.8911
0.8747
0.8572
O
Me
O
O
NO2
5
Me
((+)-6b)-BzNO2
1H NMR
(400 MHz, CDCl3)
1.92
1.91
3.00
1.01
7.33
3.05
12.75
3.16
f1 (ppm)

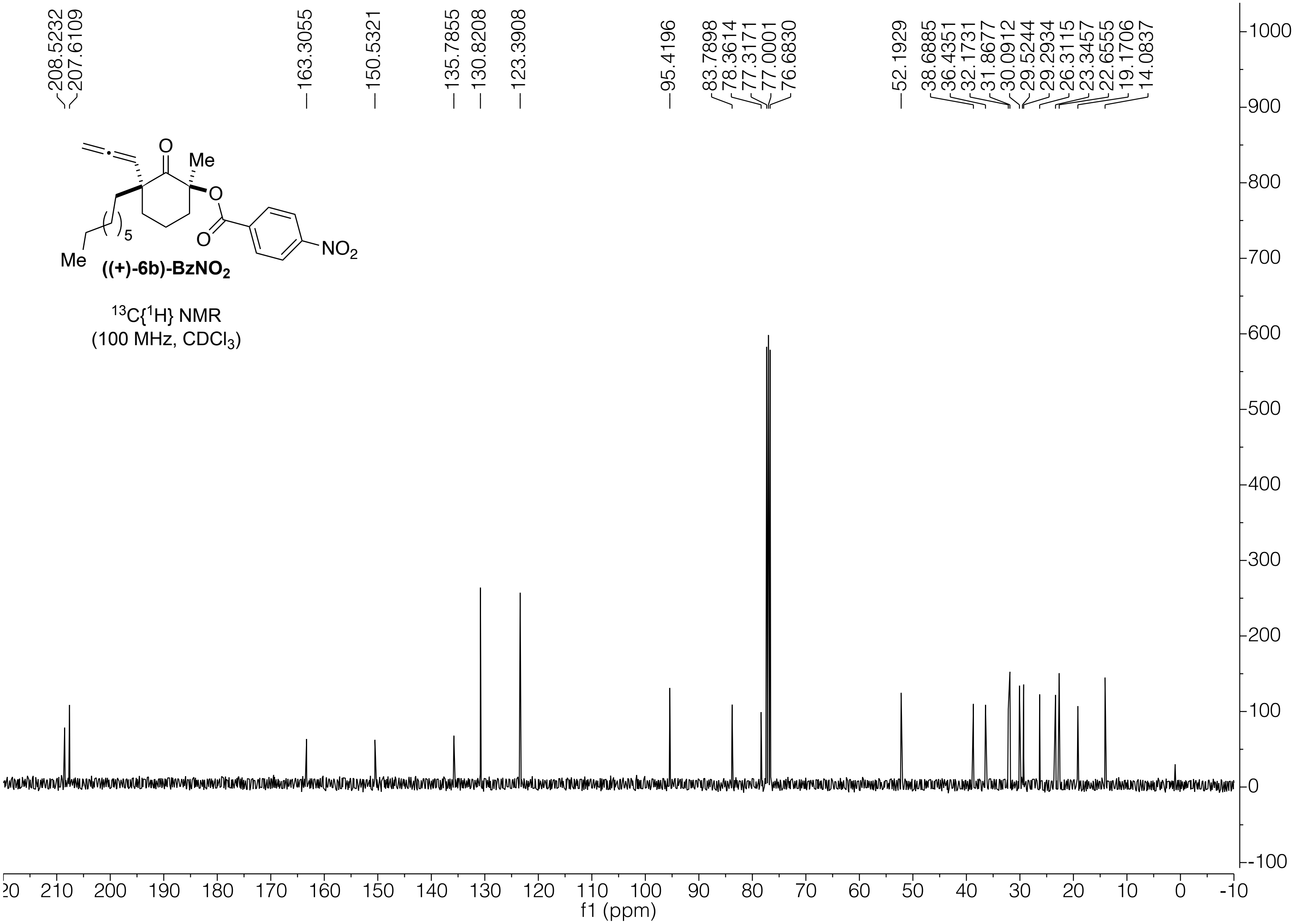

208.5232
207.6109
163.3055
150.5321
135.7855
130.8208
123.3908
95.4196
83.7898
78.3614
77.3171
77.0001
76.6830
52.1929
38.6885
36.4351
32.1731
31.8677
30.0912
29.5244
29.2934
26.3115
23.3457
22.6555
19.1706
14.0837
O
Me
O
O
NO2
5
Me
((+)-6b)-BzNO2
13C{1H} NMR
(100 MHz, CDCl3)
f1 (ppm)

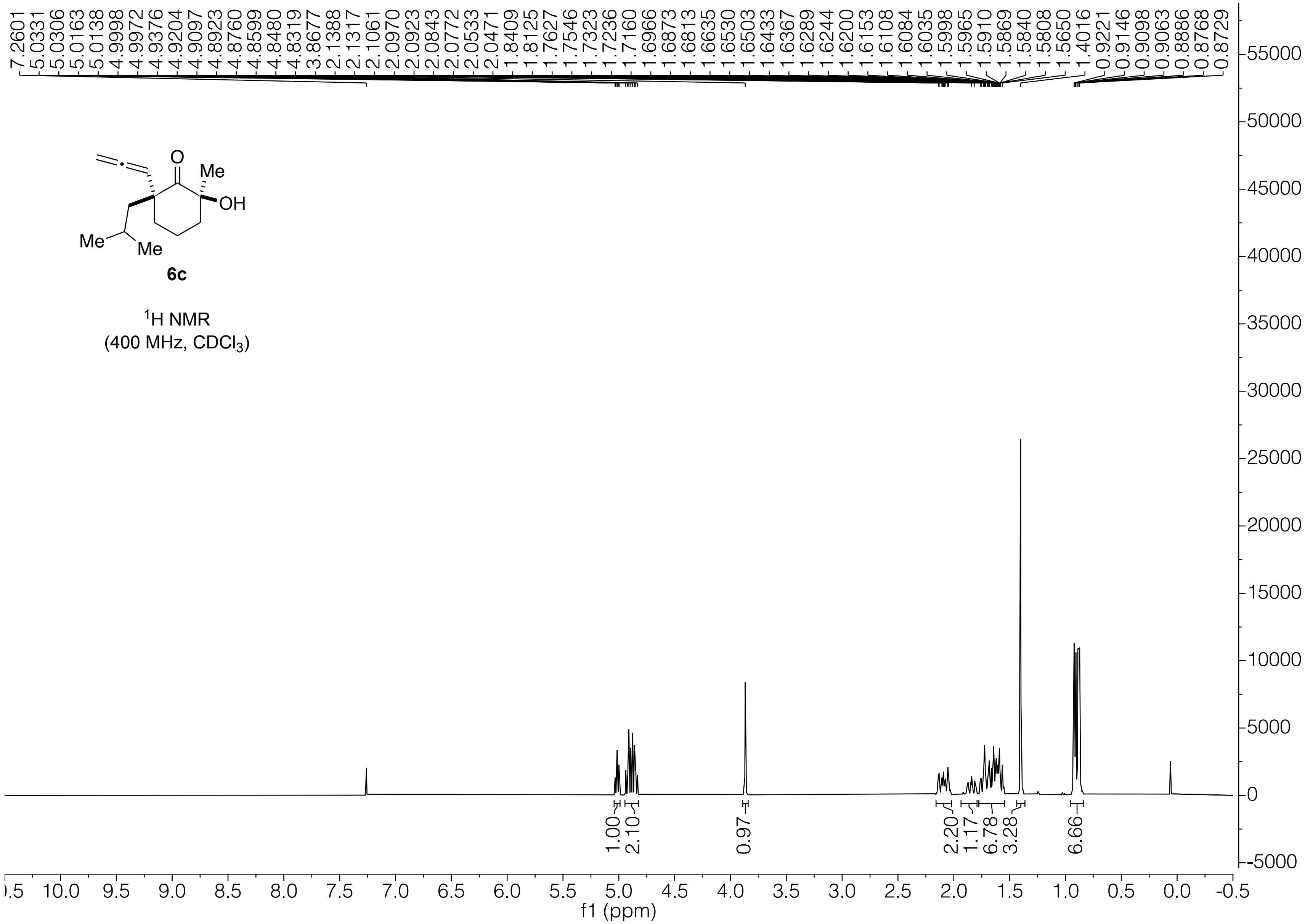
7.2601 5.0331 5.0306 5.0163 5.0138 4.9998 4.9972 4.9376 4.9204 4.9097 4.8923 4.8760 4.8599 4.8480 4.8319 3.8677 2.1388 2.1317 2.1061 2.0970 2.0923 2.0843 2.0772 2.0533 2.0471 1.8409 1.8125 1.7627 1.7546 1.7323 1.7236 1.7160 1.6966 1.6873 1.6813 1.6635 1.6530 1.6503 1.6433 1.6367 1.6289 1.6244 1.6200 1.6153 1.6108 1.6084 1.6035 1.5998 1.5965 1.5910 1.5869 1.5840 1.5808 1.5650 1.4016 0.9221 0.9146 0.9098 0.9063 0.8886 0.8768 0.8729
O
Me
OH
Me
Me
6c
^{1}H NMR
(400 MHz, $CDCl_3$)
1.00
2.10
0.97
2.20
1.17
6.78
3.28
6.66
f1 (ppm)

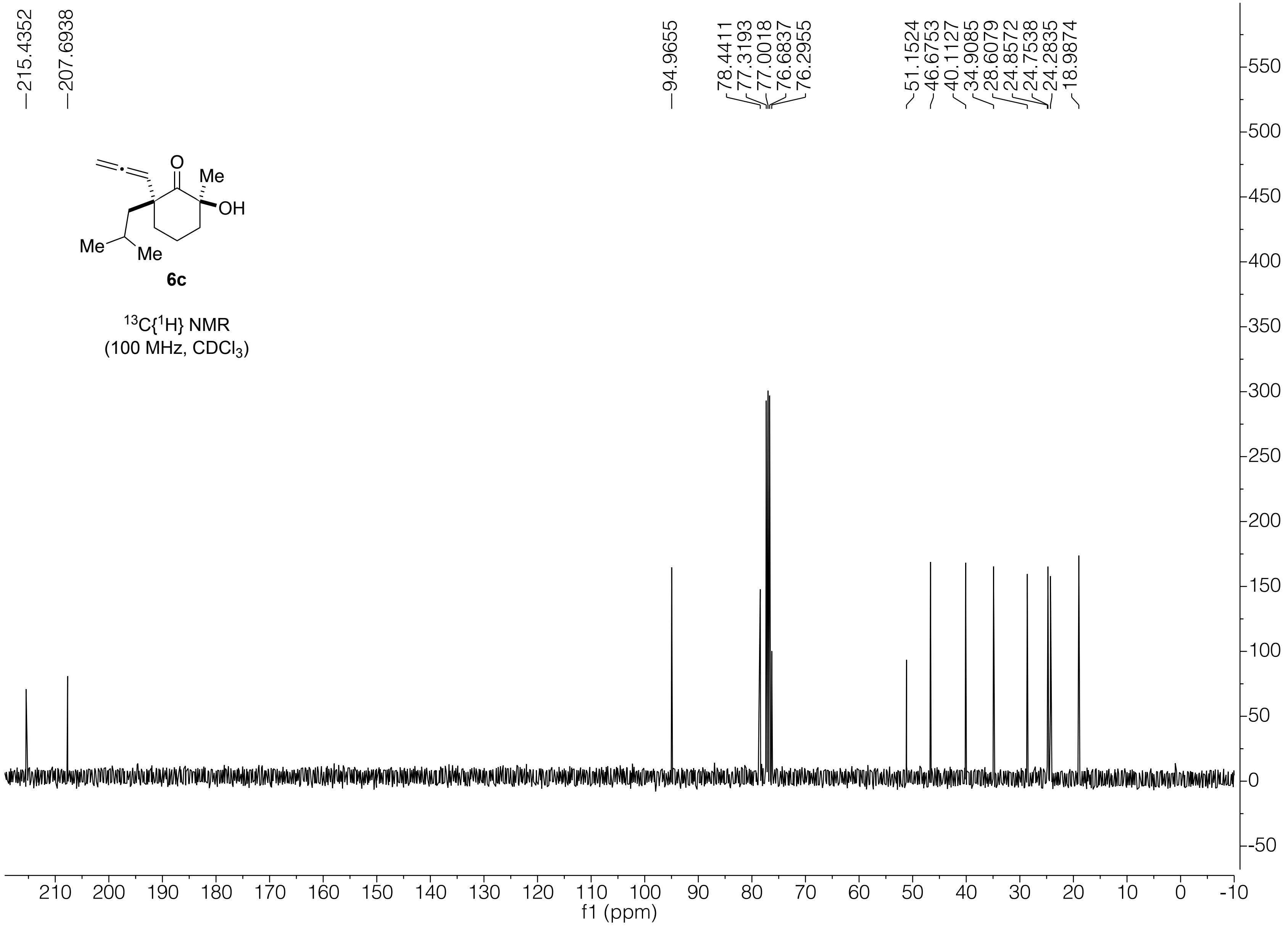

215.4352
207.6938
94.9655
78.4411
77.3193
77.0018
76.6837
76.2955
51.1524
46.6753
40.1127
34.9085
28.6079
24.8572
24.7538
24.2835
18.9874
O
Me
OH
Me
Me
6c
$^{13}C\{^1H\}$ NMR
(100 MHz, $CDCl_3$)
f1 (ppm)

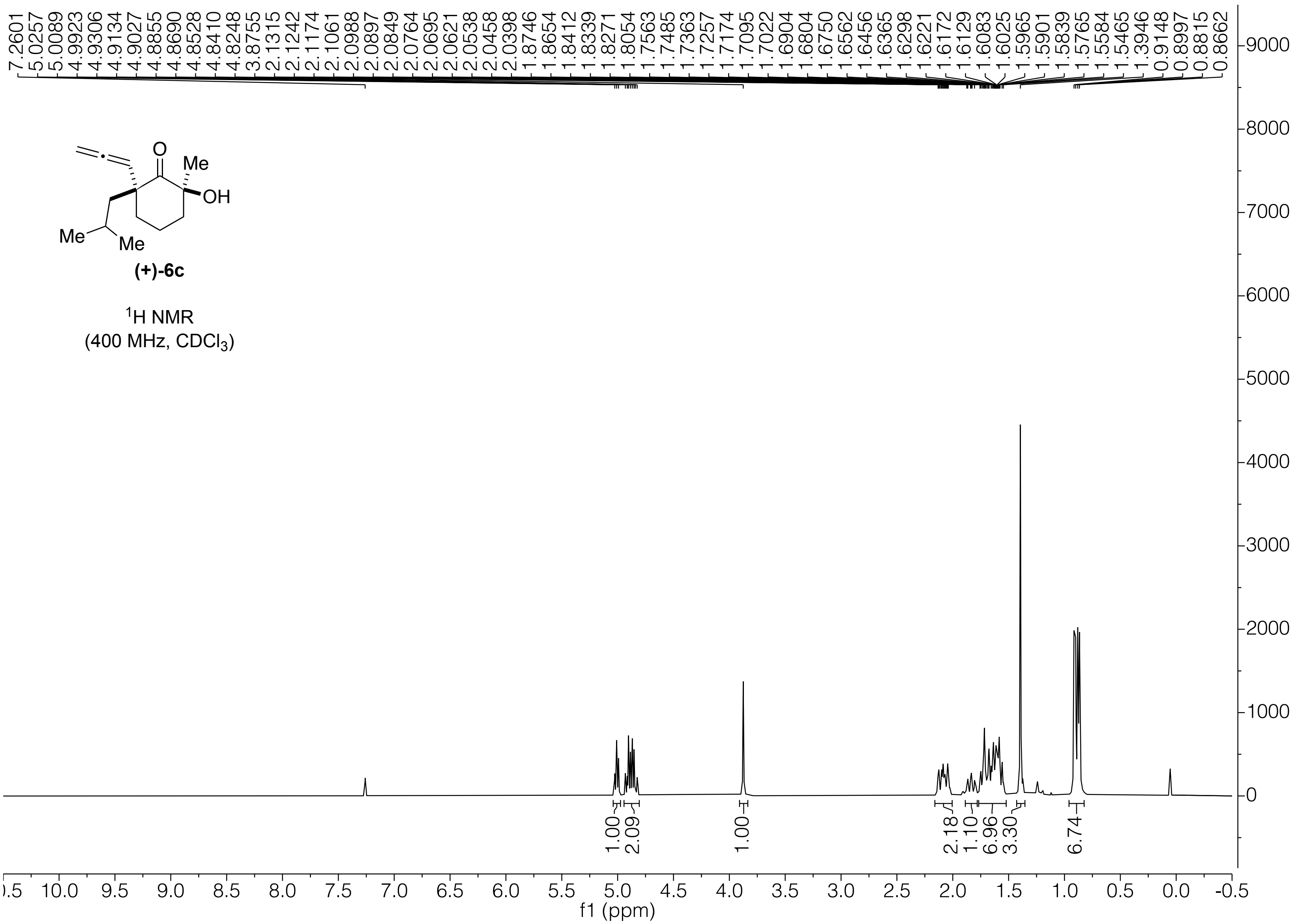

7.2601
5.0257
5.0089
4.9923
4.9306
4.9134
4.9027
4.8855
4.8690
4.8528
4.8410
4.8248
3.8755
2.1315
2.1242
2.1174
2.1061
2.0988
2.0897
2.0849
2.0764
2.0695
2.0621
2.0538
2.0458
2.0398
1.8746
1.8654
1.8412
1.8339
1.8271
1.8054
1.7563
1.7485
1.7363
1.7257
1.7174
1.7095
1.7022
1.6904
1.6804
1.6750
1.6562
1.6456
1.6365
1.6298
1.6221
1.6172
1.6129
1.6083
1.6025
1.5965
1.5901
1.5839
1.5765
1.5584
1.5465
1.3946
0.9148
0.8997
0.8815
0.8662
O
Me
OH
Me
Me
(+)-6c
^{1}H NMR
(400 MHz, $CDCl_3$)
1.00
2.09
1.00
2.18
1.10
6.96
3.30
6.74
f1 (ppm)

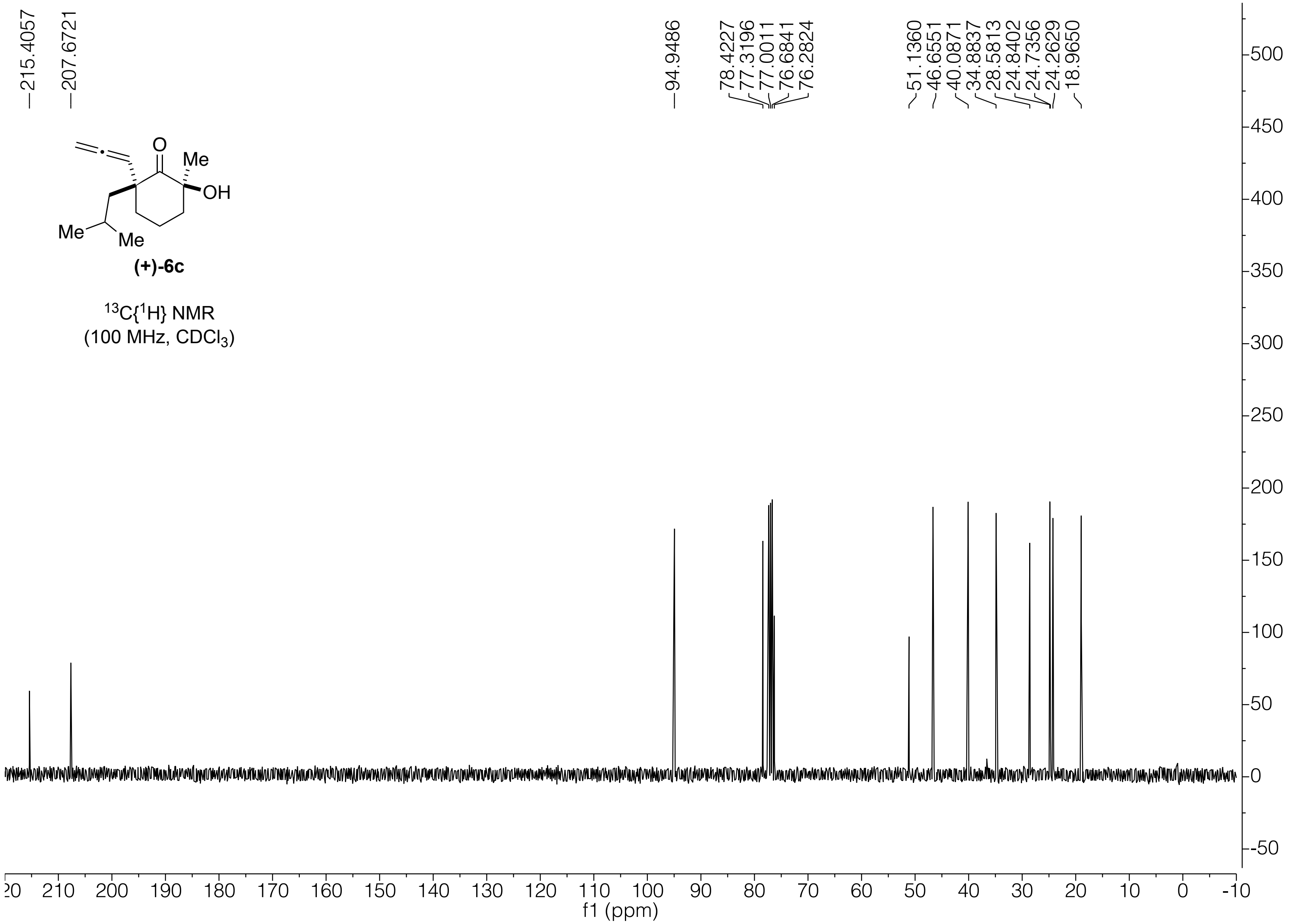
215.4057
207.6721
94.9486
78.4227
77.3196
77.0011
76.6841
76.2824
51.1360
46.6551
40.0871
34.8837
28.5813
24.8402
24.7356
24.2629
18.9650
O
Me
OH
Me
Me
(+)-6c
$^{13}C\{^{1}H\}$ NMR
(100 MHz, $CDCl_3$)
f1 (ppm)

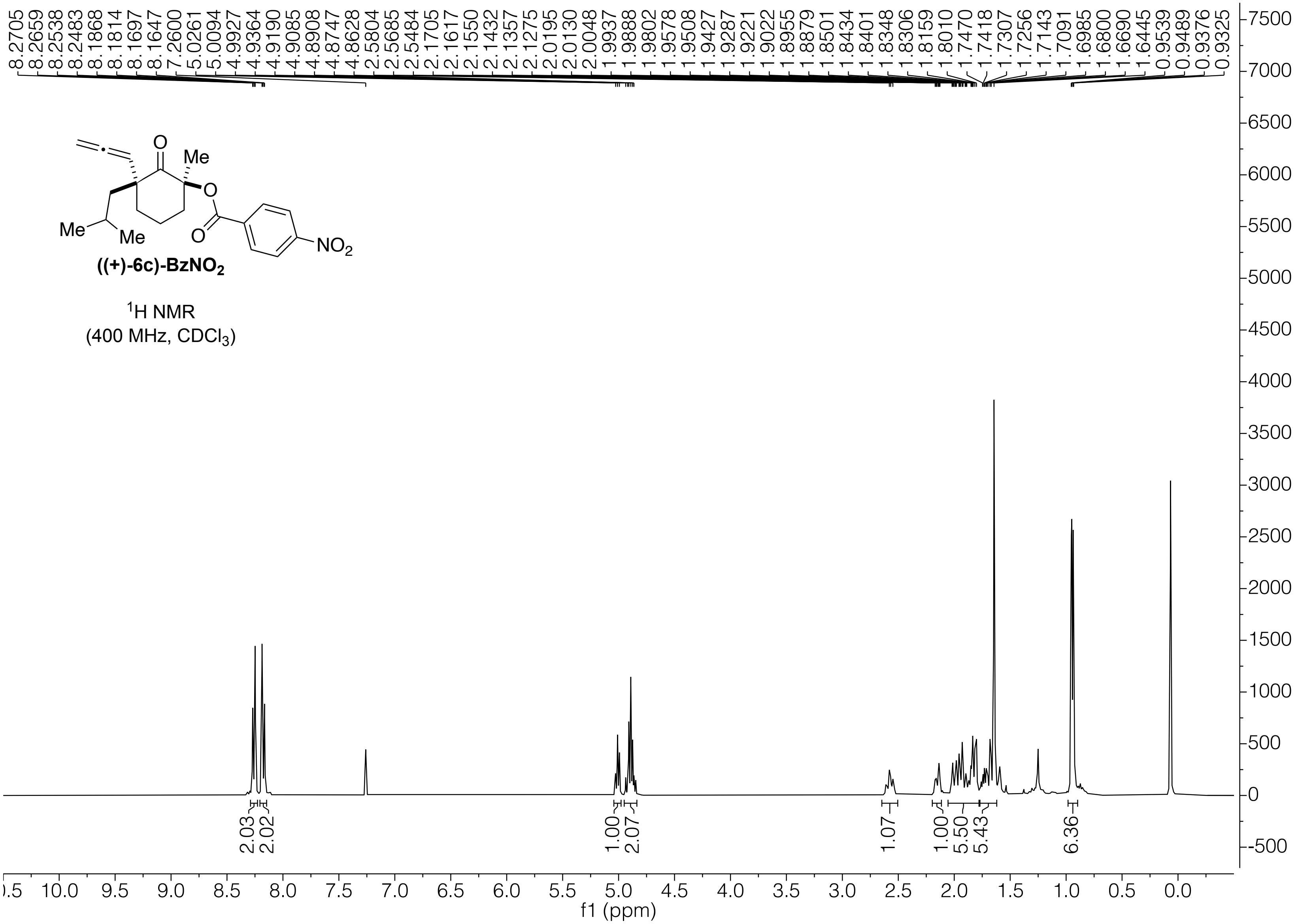
((+)-6c)-BzNO2
1H NMR
(400 MHz, CDCl3)
f1 (ppm)

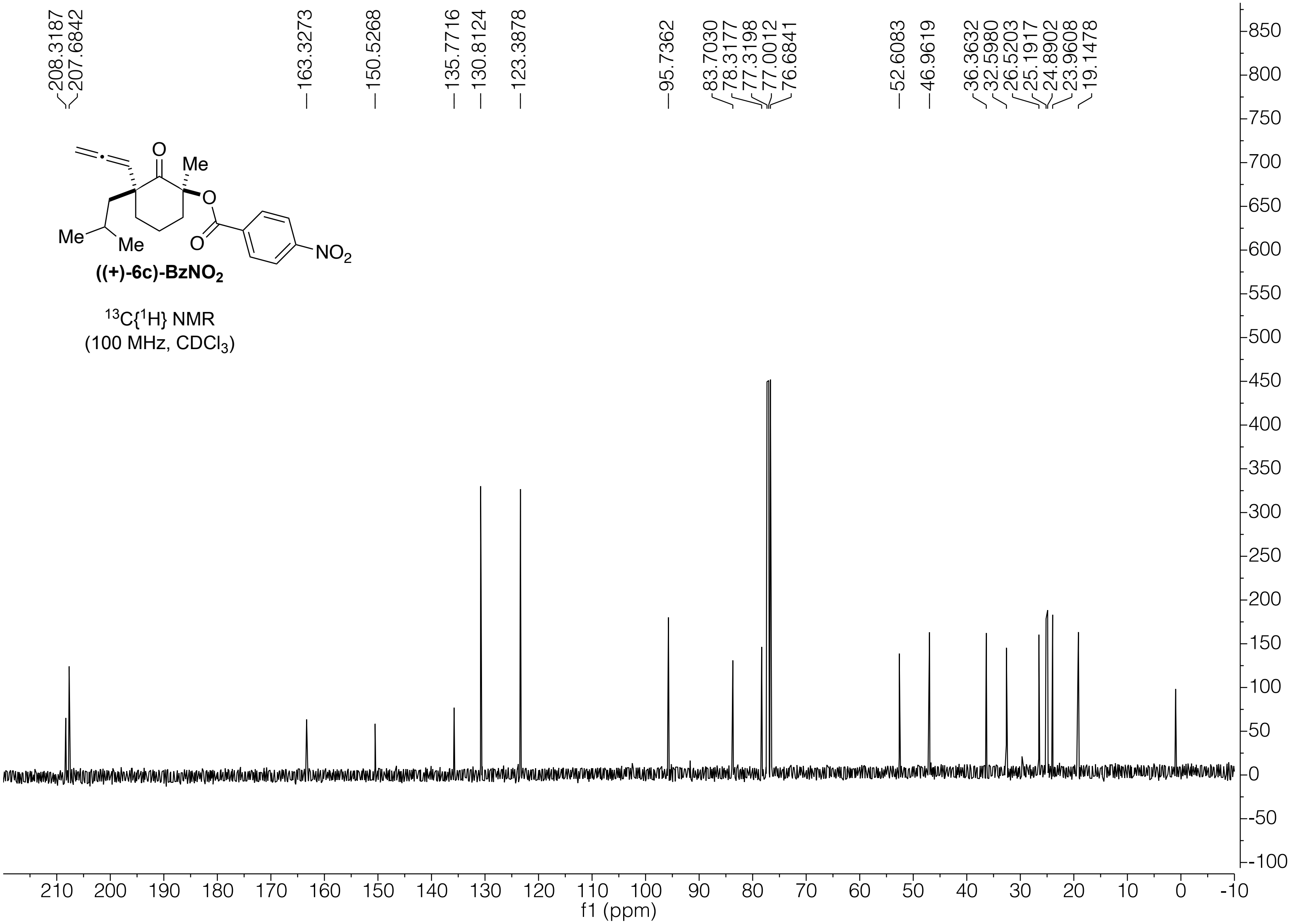

208.3187
207.6842
163.3273
150.5268
135.7716
130.8124
123.3878
95.7362
83.7030
78.3177
77.3198
77.0012
76.6841
52.6083
46.9619
36.3632
32.5980
26.5203
25.1917
24.8902
23.9608
19.1478
Me
O
Me
Me
O
O
NO_2
((+)-6c)-$BzNO_2$
$^{13}C\{^1H\}$ NMR
(100 MHz, $CDCl_3$)
f1 (ppm)

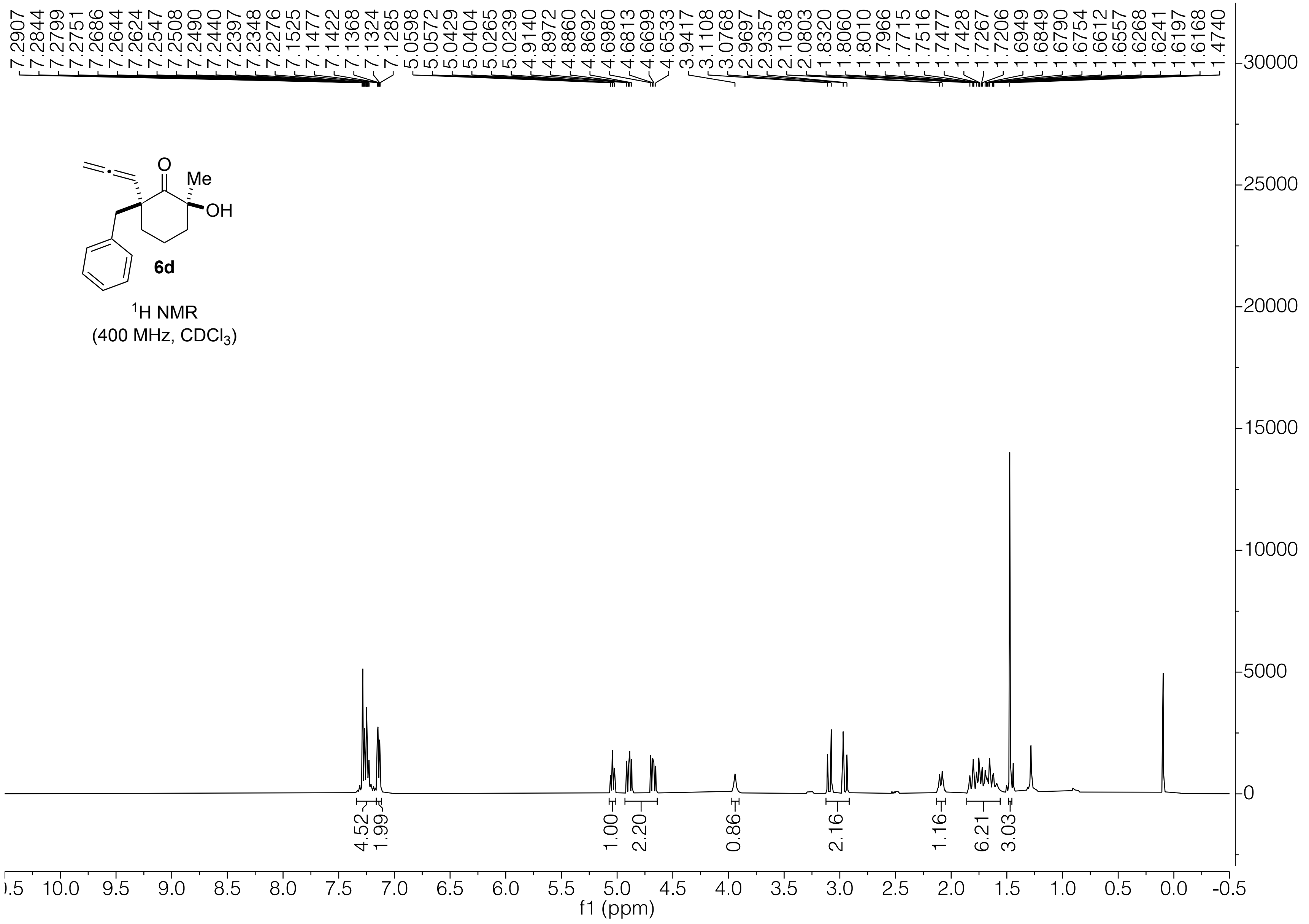

6d
^{1}H NMR
(400 MHz, CDCl$_3$)
f1 (ppm)

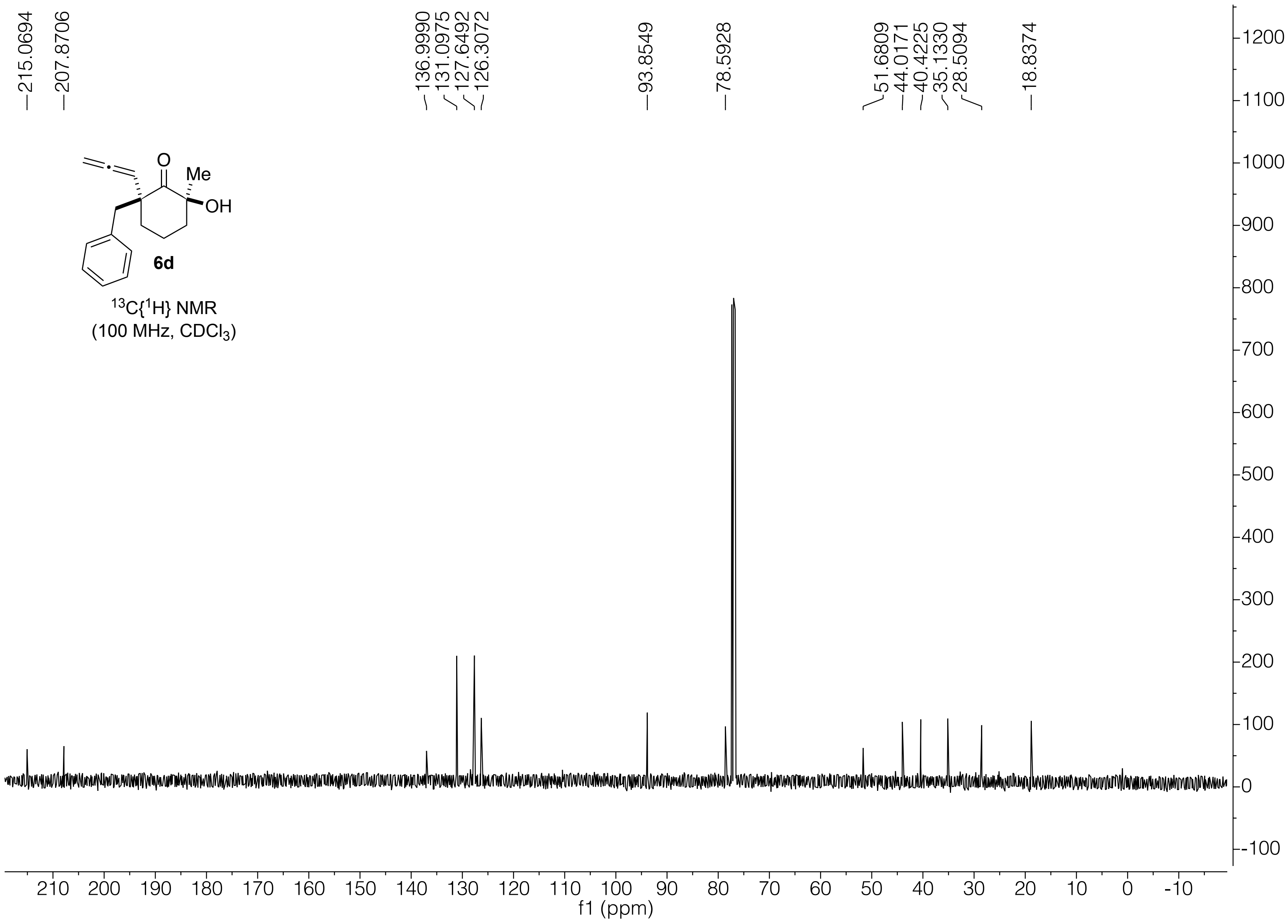
215.0694
207.8706
136.9990
131.0975
127.6492
126.3072
93.8549
78.5928
51.6809
44.0171
40.4225
35.1330
28.5094
18.8374
O
Me
OH
6d
$^{13}C\{^1H\}$ NMR
(100 MHz, $CDCl_3$)
f1 (ppm)

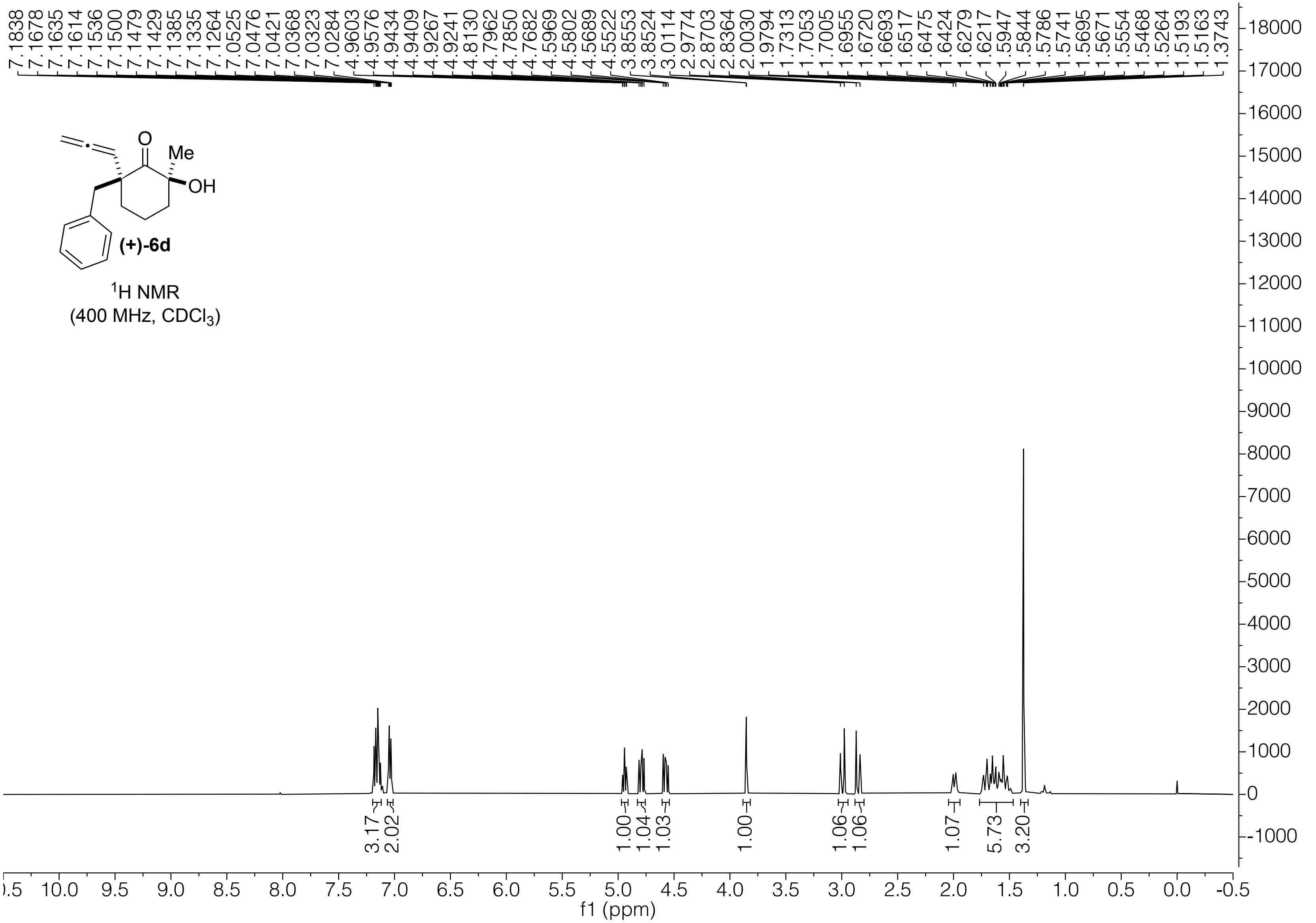
7.1838 7.1678 7.1635 7.1614 7.1536 7.1500 7.1479 7.1429 7.1385 7.1335 7.1264 7.0525 7.0476 7.0421 7.0368 7.0323 7.0284 4.9603 4.9576 4.9434 4.9409 4.9267 4.9241 4.8130 4.7962 4.7850 4.7682 4.5969 4.5802 4.5689 4.5522 3.8553 3.8524 3.0114 2.9774 2.8703 2.8364 2.0030 1.9794 1.7313 1.7053 1.7005 1.6955 1.6720 1.6693 1.6517 1.6475 1.6424 1.6279 1.6217 1.5947 1.5844 1.5786 1.5741 1.5695 1.5671 1.5554 1.5468 1.5264 1.5193 1.5163 1.3743
Me
OH
O
(+)-6d
^{1}H NMR
(400 MHz, CDCl$_3$)
3.17
2.02
1.00
1.04
1.03
1.00
1.06
1.06
1.07
5.73
3.20
f1 (ppm)

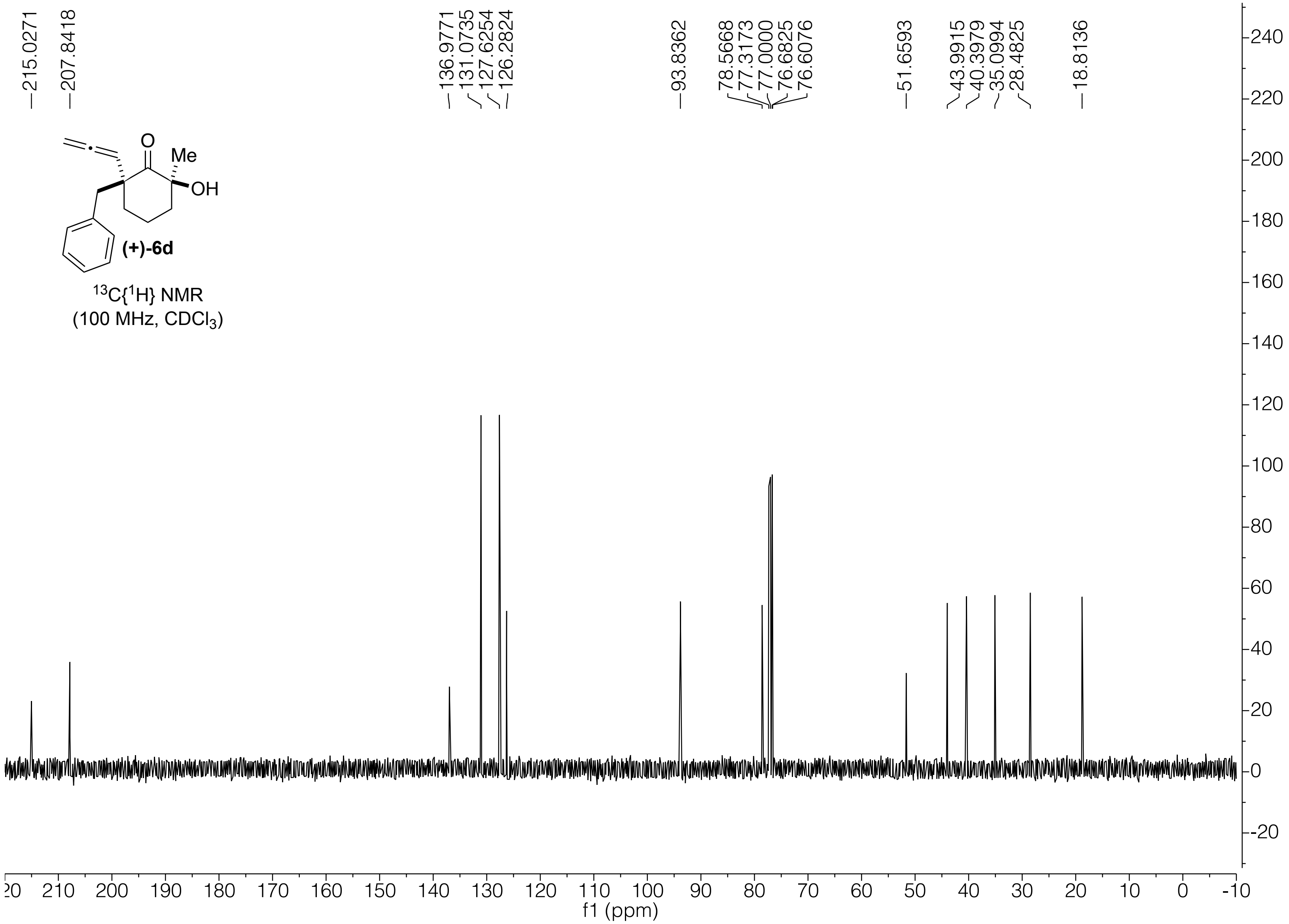

215.0271
207.8418
136.9771
131.0735
127.6254
126.2824
93.8362
78.5668
77.3173
77.0000
76.6825
76.6076
51.6593
43.9915
40.3979
35.0994
28.4825
18.8136
O
Me
OH
(+)-6d
$^{13}C\{^{1}H\}$ NMR
(100 MHz, $CDCl_3$)
f1 (ppm)

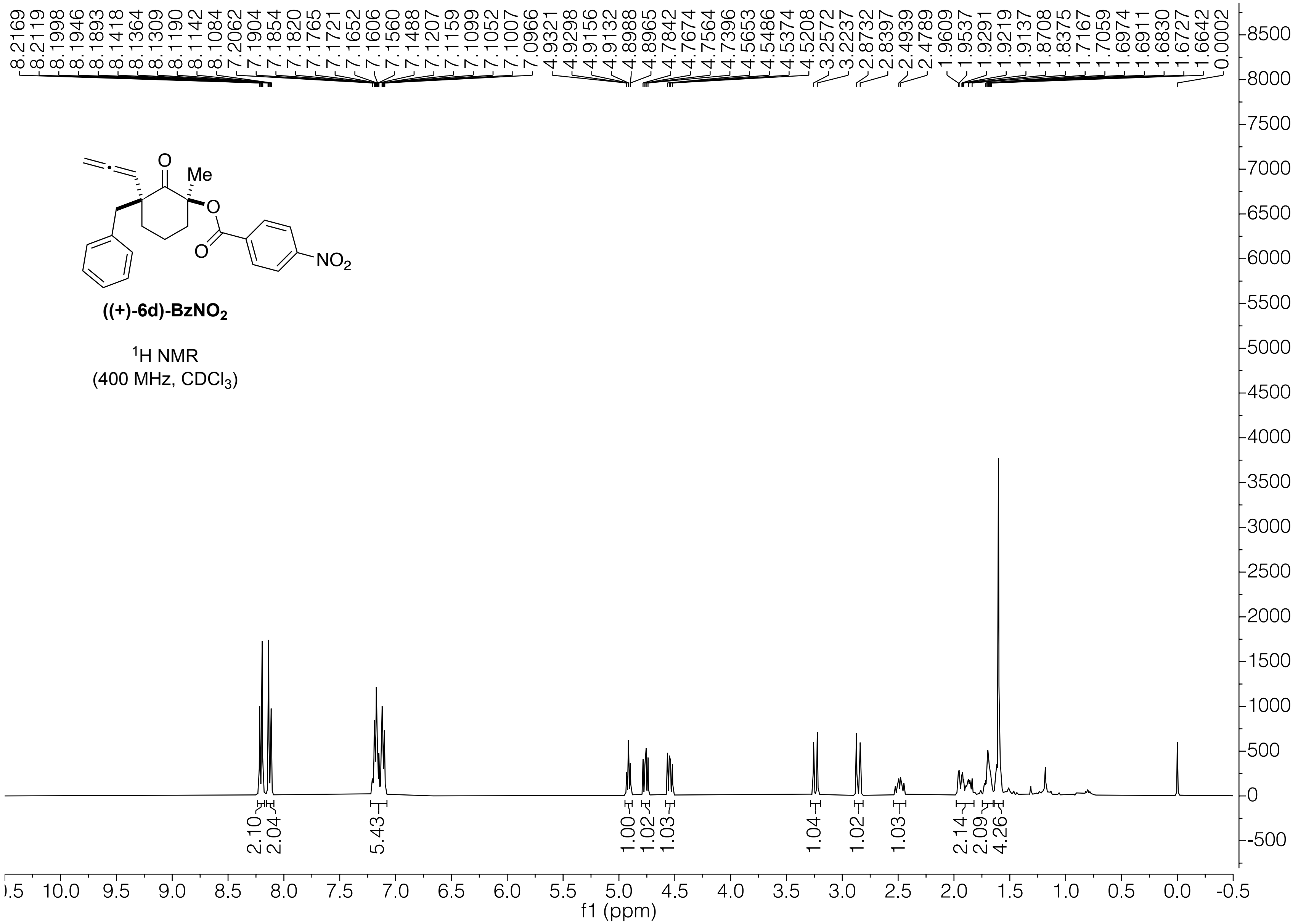

8.2169
8.2119
8.1998
8.1946
8.1893
8.1418
8.1364
8.1309
8.1190
8.1142
8.1084
7.2062
7.1904
7.1854
7.1820
7.1765
7.1721
7.1652
7.1606
7.1560
7.1488
7.1207
7.1159
7.1099
7.1052
7.1007
7.0966
4.9321
4.9298
4.9156
4.9132
4.8988
4.8965
4.7842
4.7674
4.7564
4.7396
4.5653
4.5486
4.5374
4.5208
3.2572
3.2237
2.8732
2.8397
2.4939
2.4789
1.9609
1.9537
1.9291
1.9219
1.9137
1.8708
1.8375
1.7167
1.7059
1.6974
1.6911
1.6830
1.6727
1.6642
0.0002
O
Me
O
O
NO2
((+)-6d)-BzNO2
1H NMR
(400 MHz, CDCl3)
2.10
2.04
5.43
1.00
1.02
1.03
1.04
1.02
1.03
2.14
2.09
4.26
f1 (ppm)

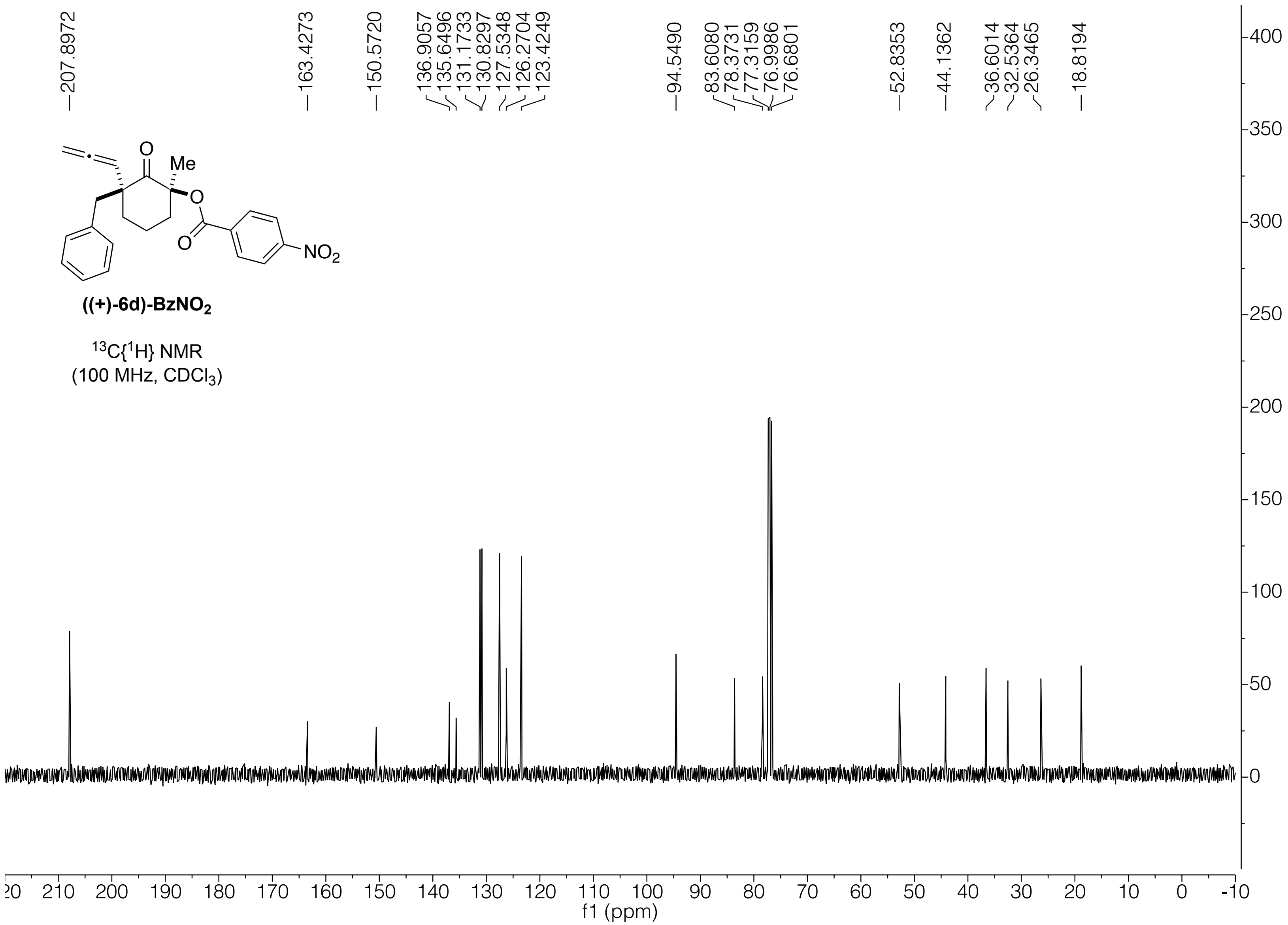

207.8972
163.4273
150.5720
136.9057
135.6496
131.1733
130.8297
127.5348
126.2704
123.4249
94.5490
83.6080
78.3731
77.3159
76.9986
76.6801
52.8353
44.1362
36.6014
32.5364
26.3465
18.8194
Me
O
O
O
NO2
((+)-6d)-BzNO2
13C{1H} NMR
(100 MHz, CDCl3)
f1 (ppm)

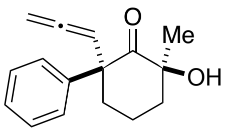

6e

^{1}H NMR
(500 MHz, $CDCl_3$)

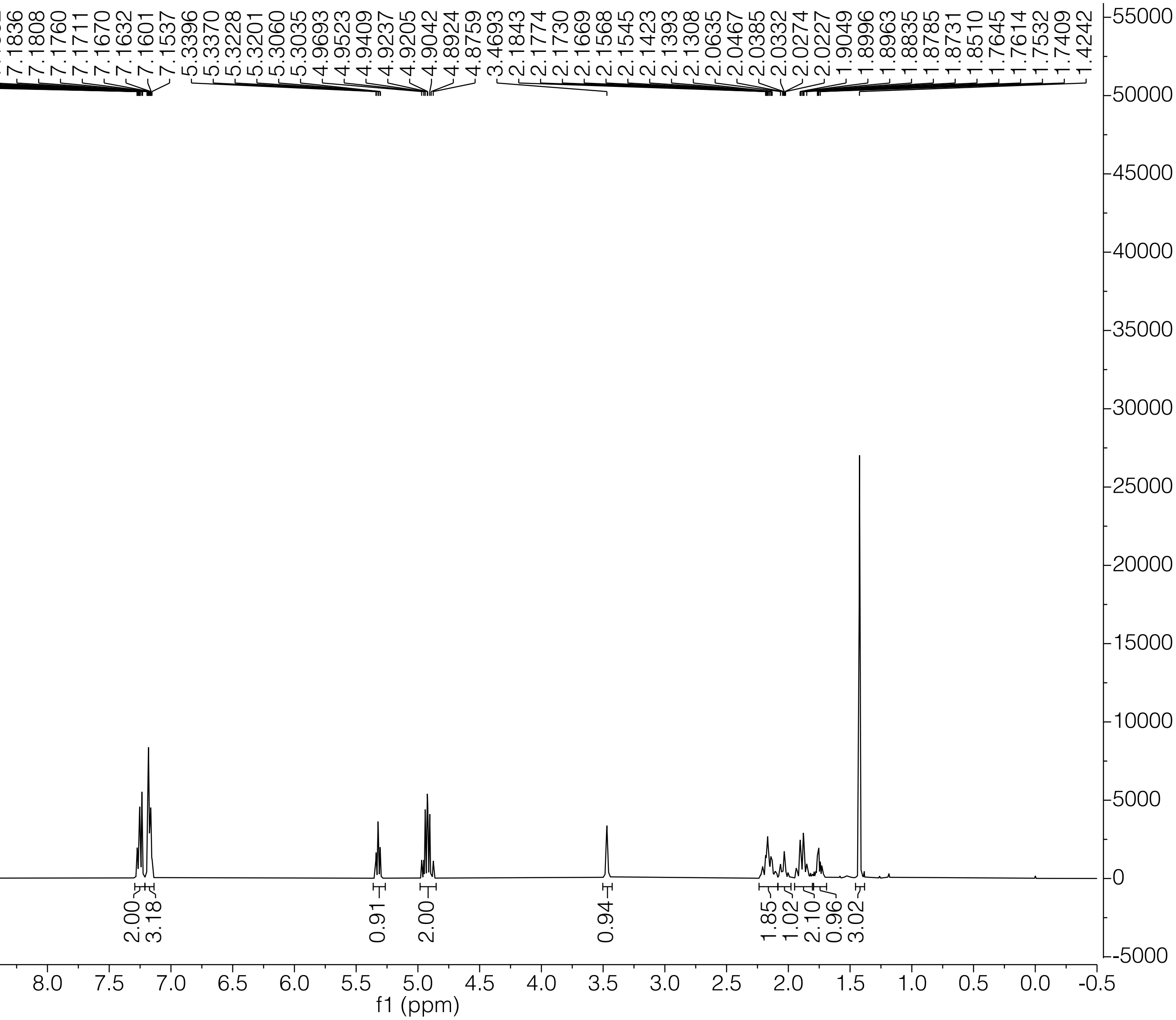

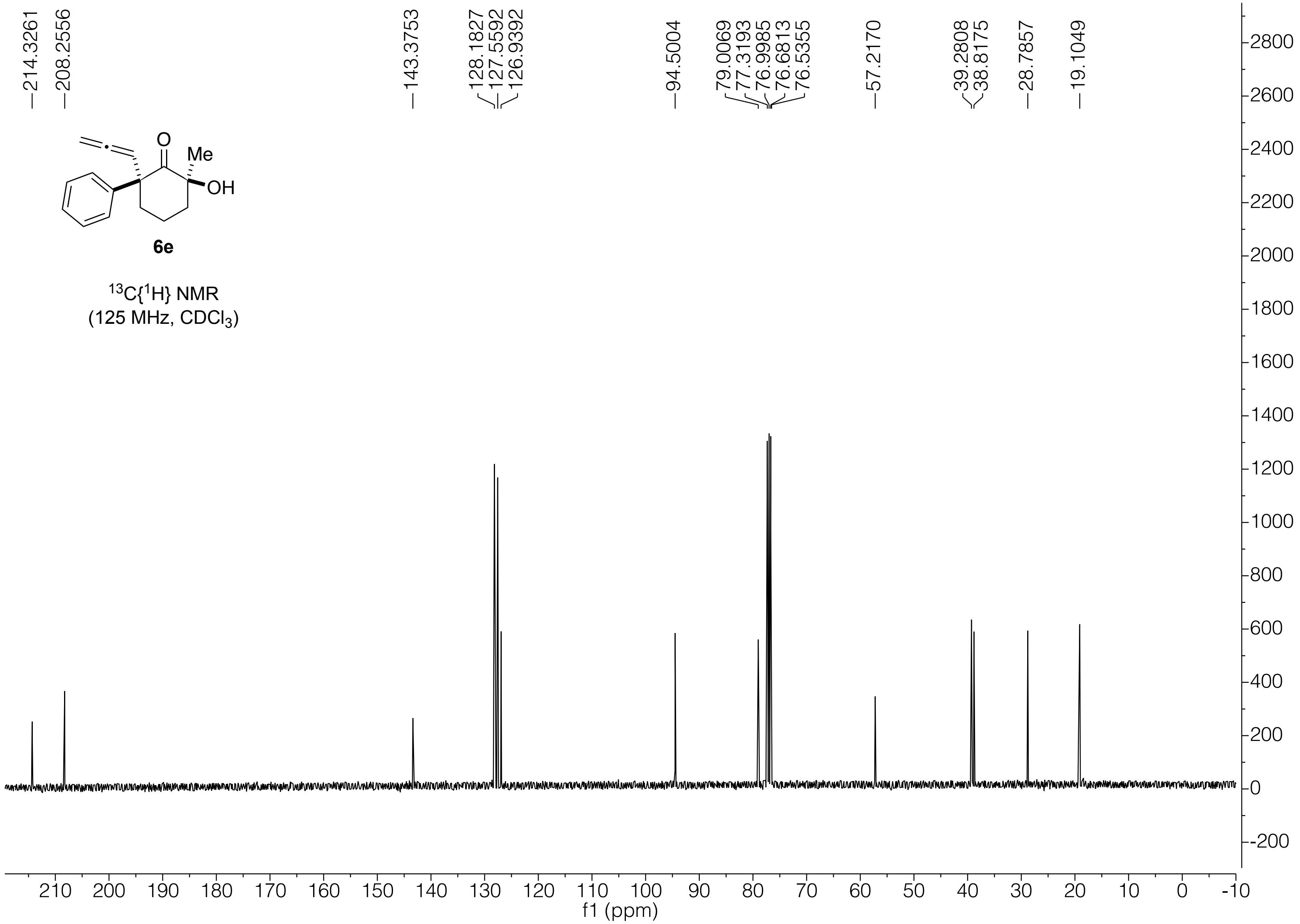
214.3261
208.2556
143.3753
128.1827
127.5592
126.9392
94.5004
79.0069
77.3193
76.9985
76.6813
76.5355
57.2170
39.2808
38.8175
28.7857
19.1049
O
Me
OH
6e
$^{13}C\{^1H\}$ NMR
(125 MHz, $CDCl_3$)
f1 (ppm)

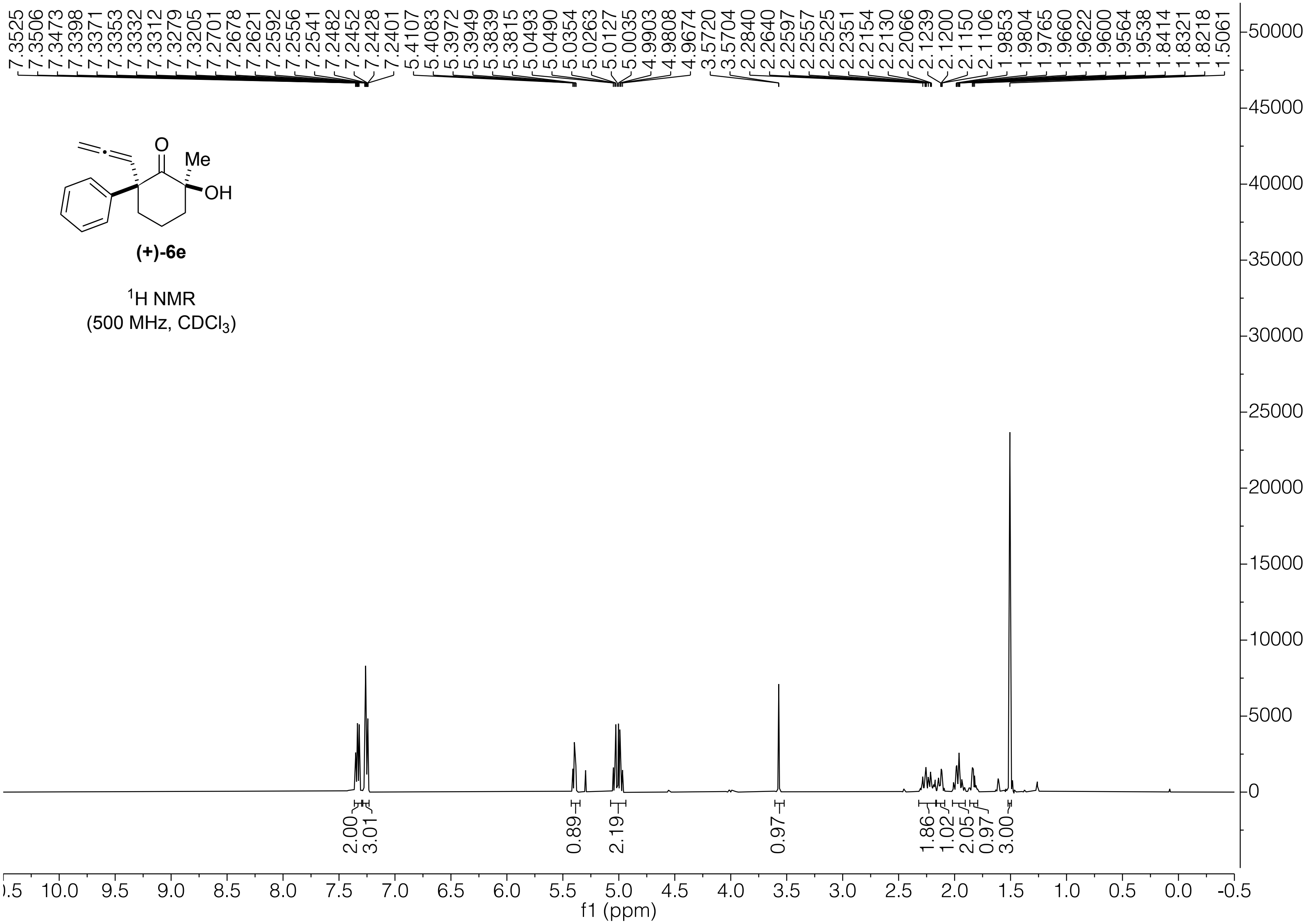

Me
OH
O
(+)-6e
^{1}H NMR
(500 MHz, CDCl$_3$)
f1 (ppm)

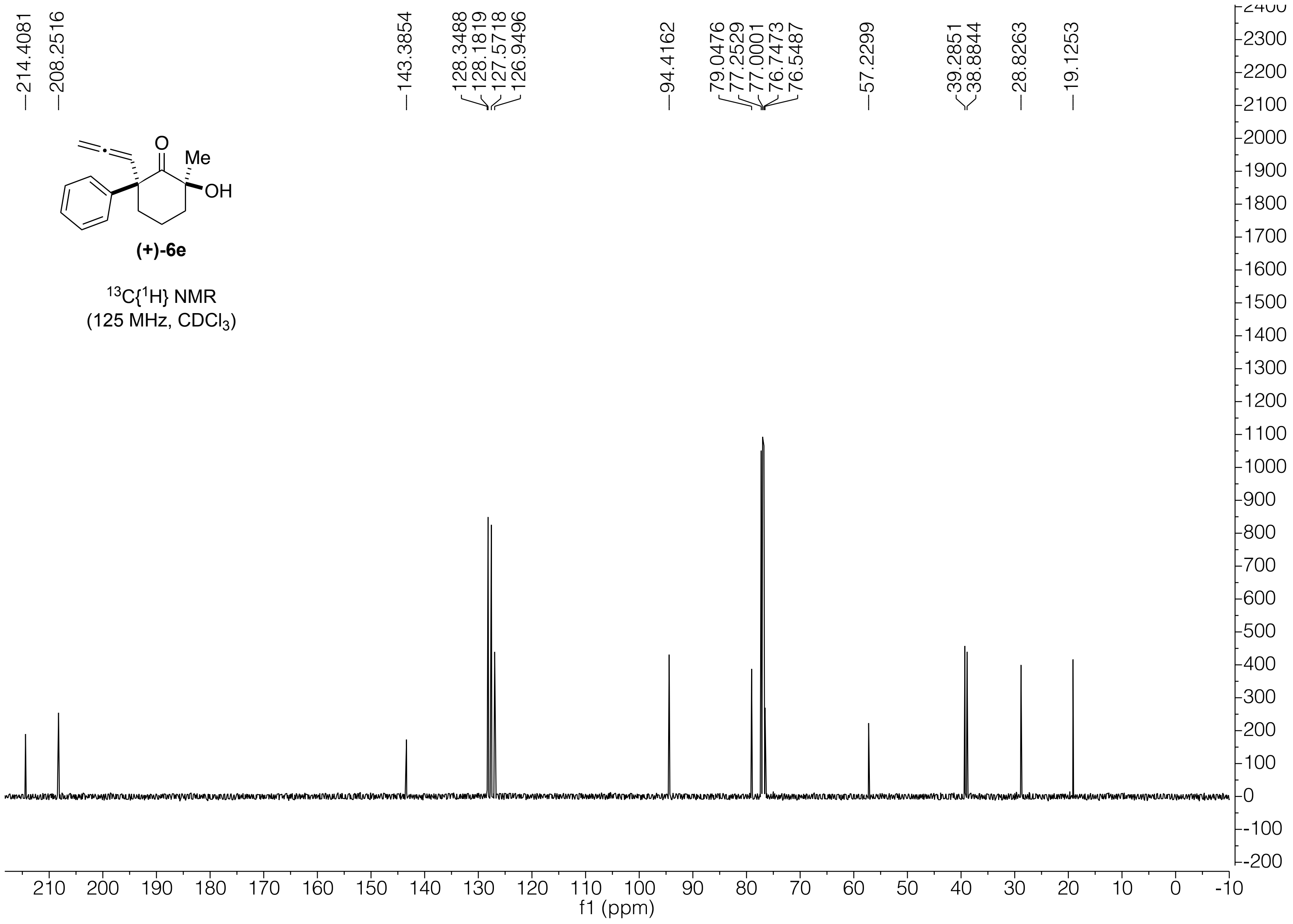
214.4081
208.2516
143.3854
128.3488
128.1819
127.5718
126.9496
94.4162
79.0476
77.2529
77.0001
76.7473
76.5487
57.2299
39.2851
38.8844
28.8263
19.1253
O
Me
OH
(+)-6e
$^{13}C\{^1H\}$ NMR
(125 MHz, $CDCl_3$)
f1 (ppm)

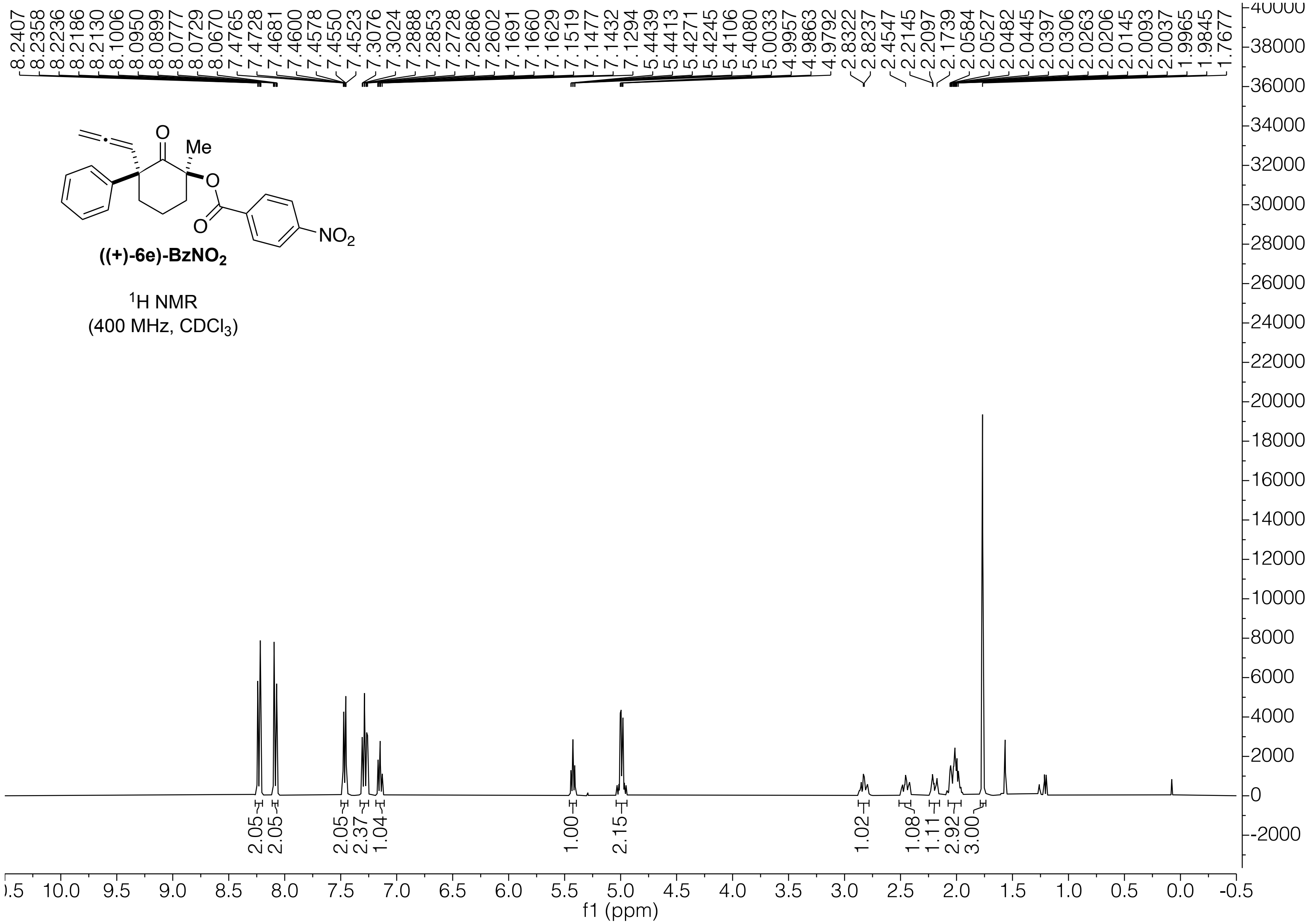
((+)-6e)-BzNO2
1H NMR
(400 MHz, CDCl3)
Me
O
NO2
f1 (ppm)

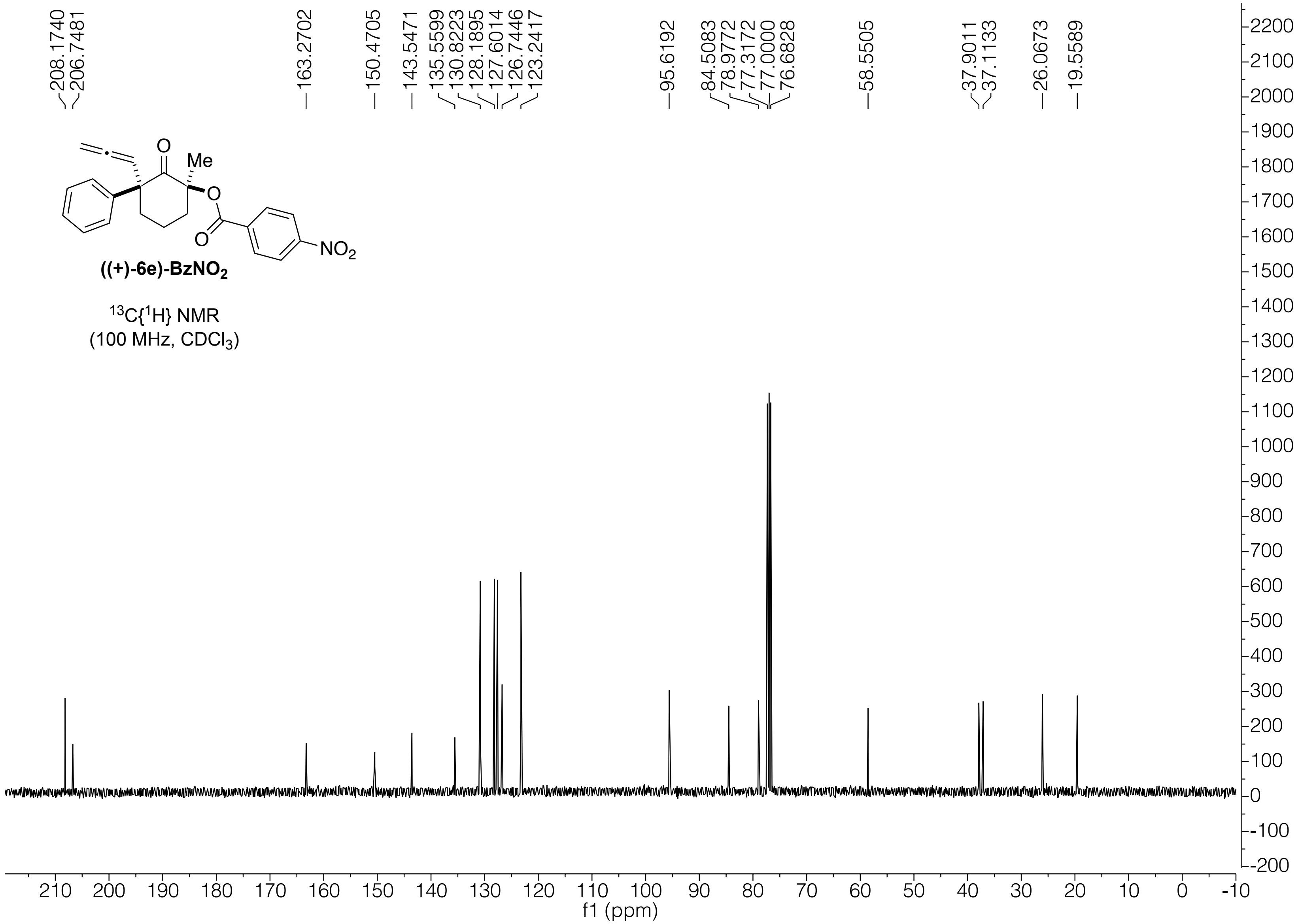
208.1740
206.7481
163.2702
150.4705
143.5471
135.5599
130.8223
128.1895
127.6014
126.7446
123.2417
95.6192
84.5083
78.9772
77.3172
77.0000
76.6828
58.5505
37.9011
37.1133
26.0673
19.5589
O
Me
O
O
NO_2
((+)-6e)-$BzNO_2$
$^{13}C\{^1H\}$ NMR
(100 MHz, $CDCl_3$)
f1 (ppm)

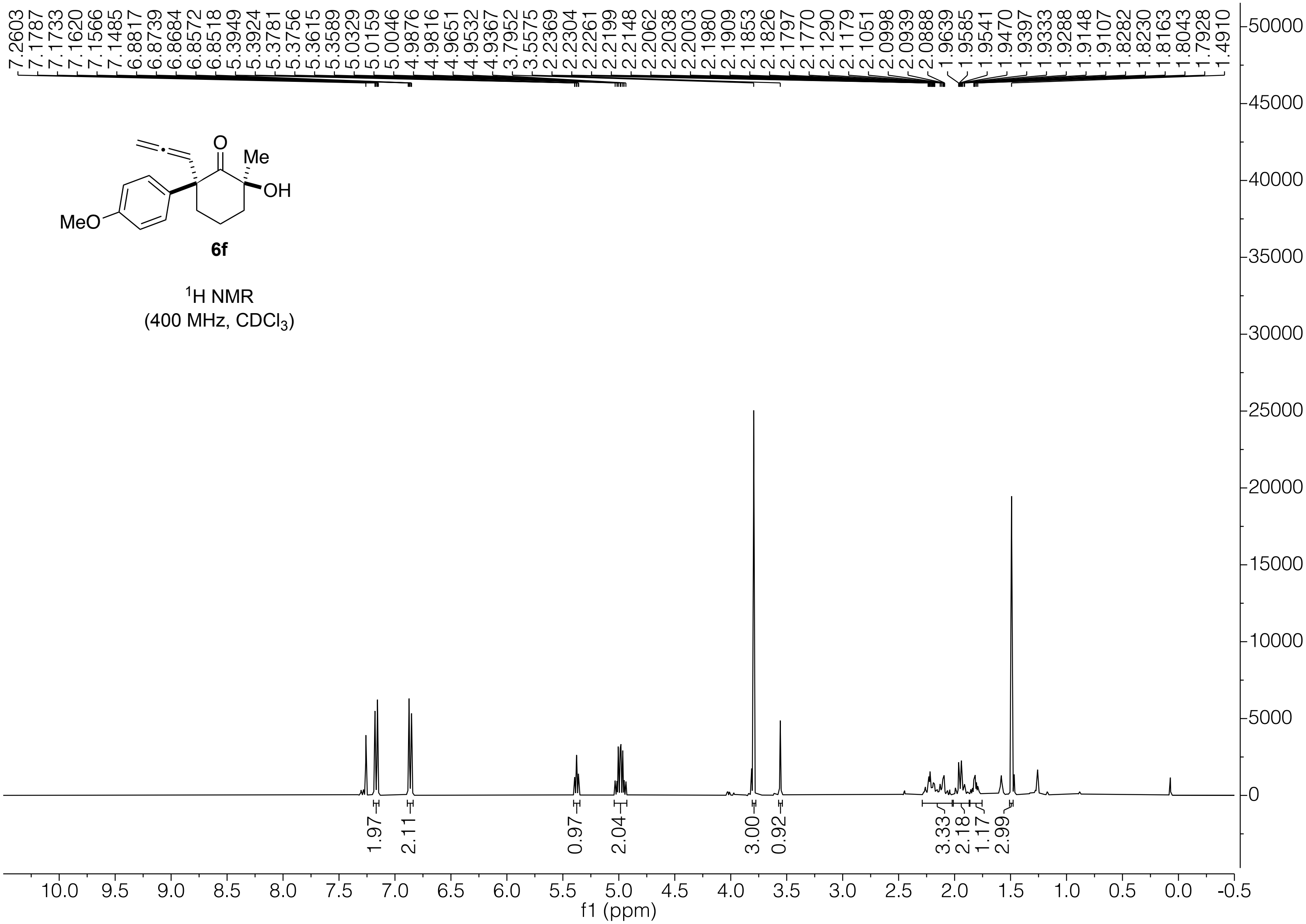

7.2603
7.1787
7.1733
7.1620
7.1566
7.1485
6.8817
6.8739
6.8684
6.8572
6.8518
5.3949
5.3924
5.3781
5.3756
5.3615
5.3589
5.0329
5.0159
5.0046
4.9876
4.9816
4.9651
4.9532
4.9367
3.7952
3.5575
2.2369
2.2304
2.2261
2.2199
2.2148
2.2062
2.2038
2.2003
2.1980
2.1909
2.1853
2.1826
2.1797
2.1770
2.1290
2.1179
2.1051
2.0998
2.0939
2.0888
1.9639
1.9585
1.9541
1.9470
1.9397
1.9333
1.9288
1.9148
1.9107
1.8282
1.8230
1.8163
1.8043
1.7928
1.4910
O
Me
OH
MeO
6f
^{1}H NMR
(400 MHz, $CDCl_3$)
1.97
2.11
0.97
2.04
3.00
0.92
3.33
2.18
1.17
2.99
f1 (ppm)

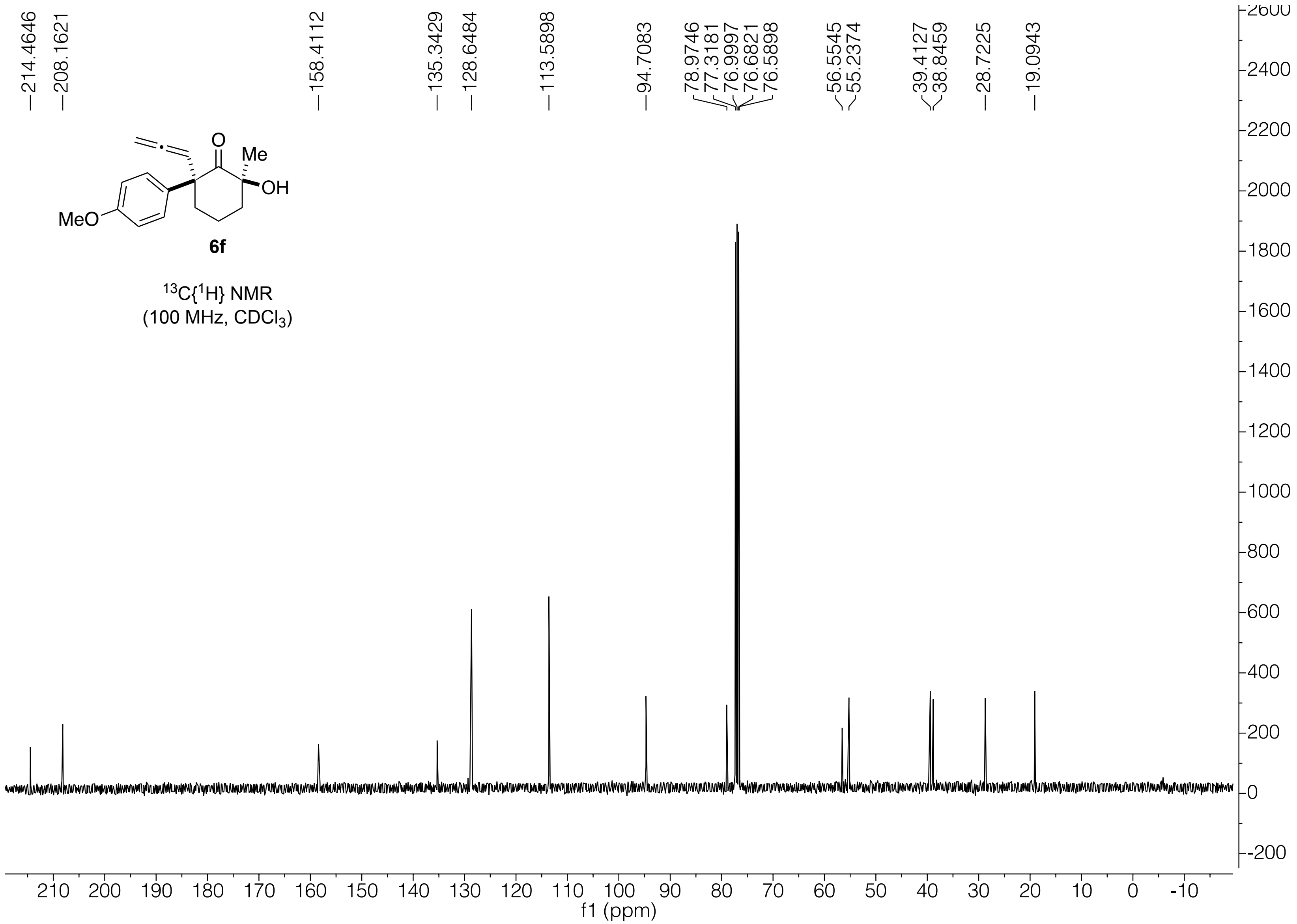
214.4646
208.1621
158.4112
135.3429
128.6484
113.5898
94.7083
78.9746
77.3181
76.9997
76.6821
76.5898
56.5545
55.2374
39.4127
38.8459
28.7225
19.0943
O
Me
OH
MeO
6f
$^{13}C\{^1H\}$ NMR
(100 MHz, $CDCl_3$)
f1 (ppm)

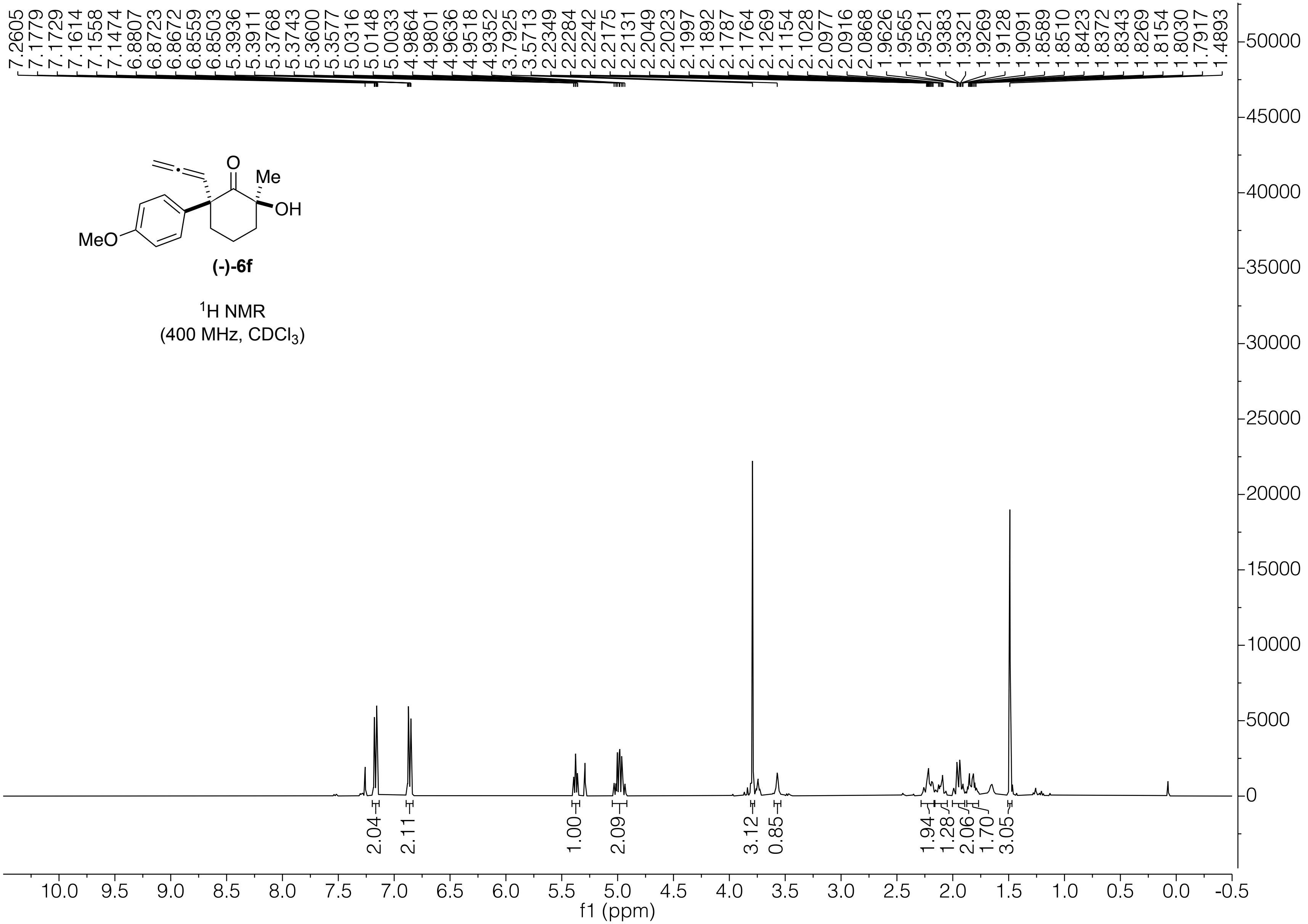

(-)-6f

^{1}H NMR
(400 MHz, $CDCl_3$)

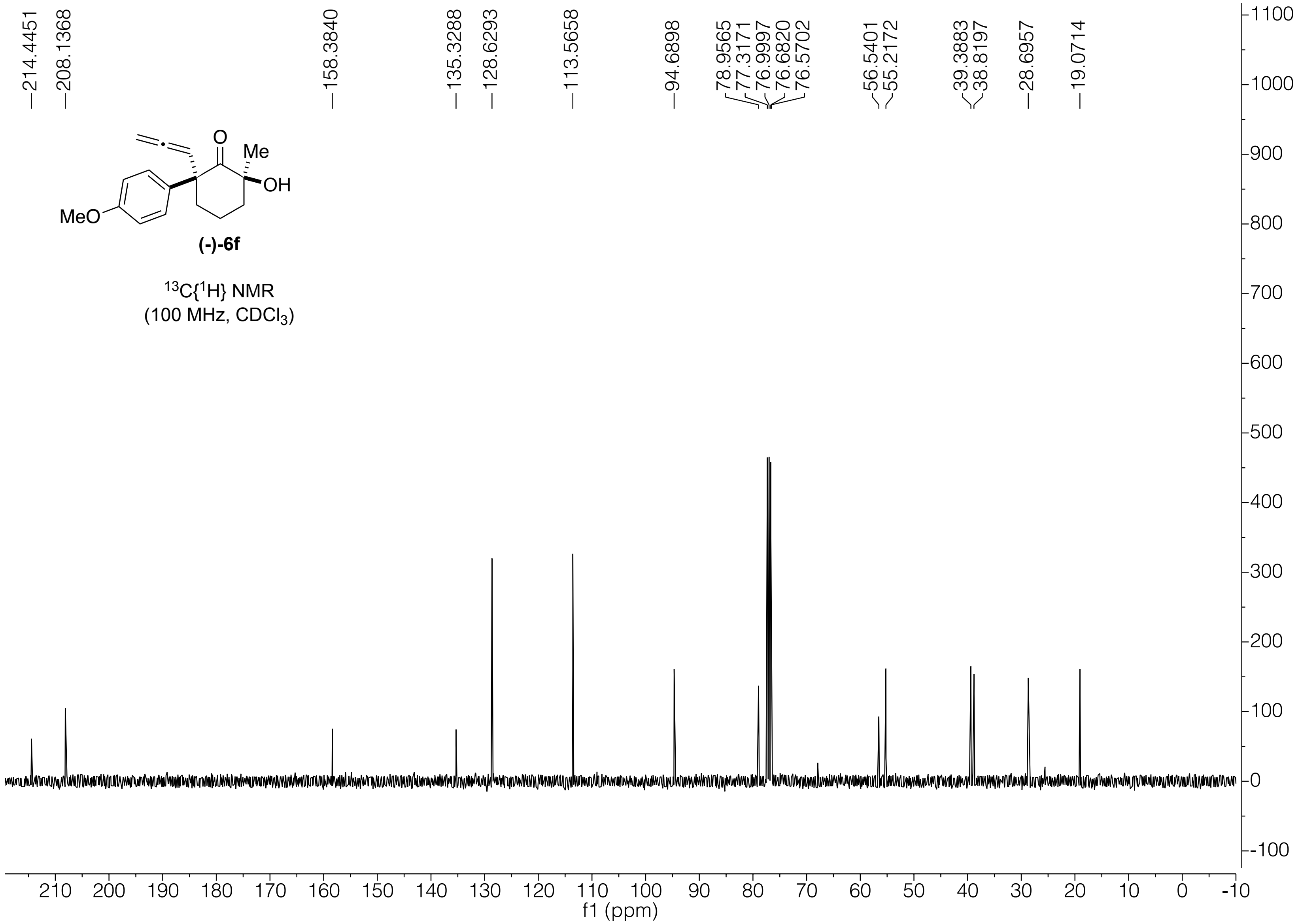

214.4451
208.1368
158.3840
135.3288
128.6293
113.5658
94.6898
78.9565
77.3171
76.9997
76.6820
76.5702
56.5401
55.2172
39.3883
38.8197
28.6957
19.0714
O
Me
OH
MeO
(-)-6f
$^{13}C\{^{1}H\}$ NMR
(100 MHz, $CDCl_3$)
f1 (ppm)

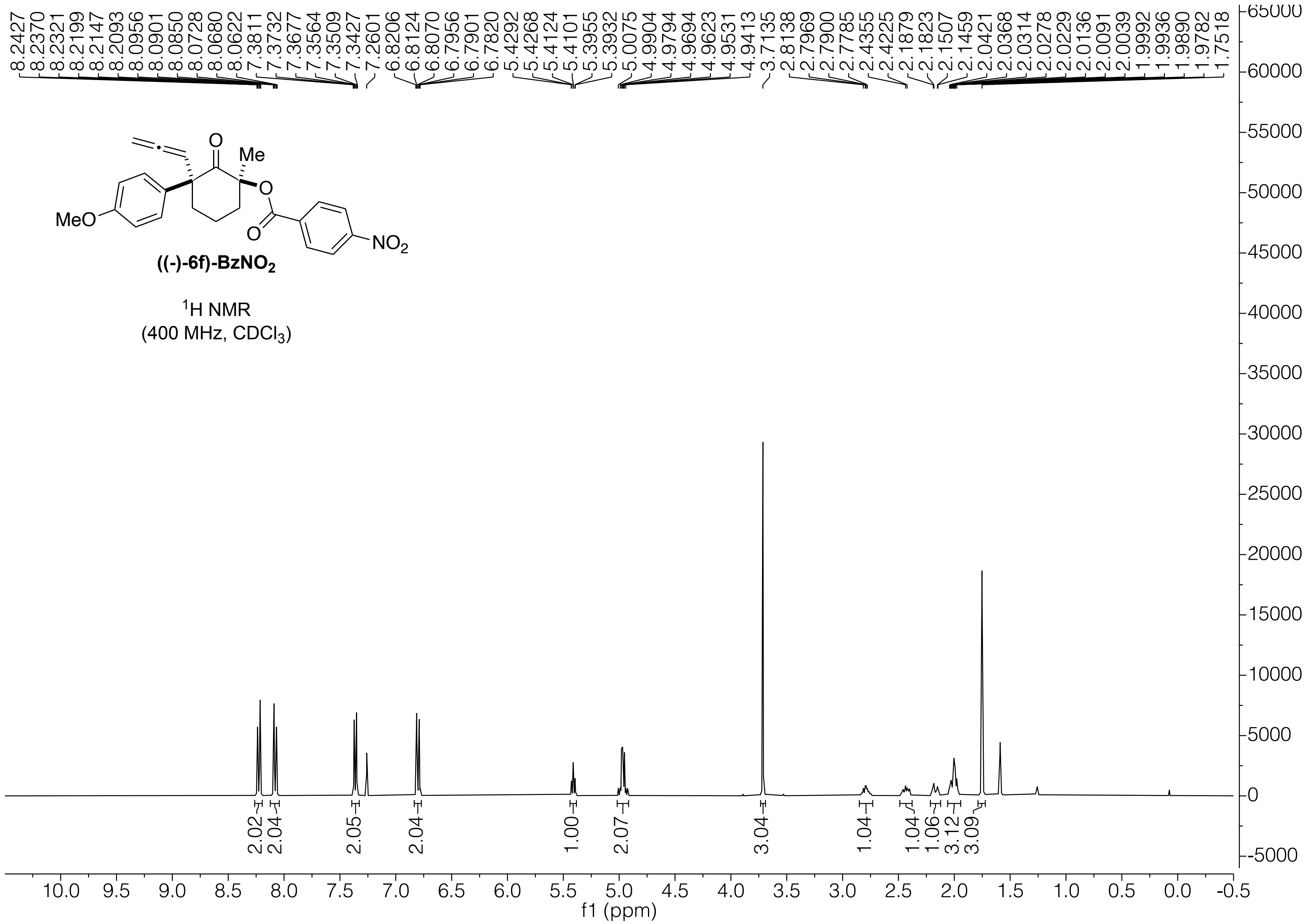

((-)-6f)-BzNO2
1H NMR
(400 MHz, CDCl3)
f1 (ppm)

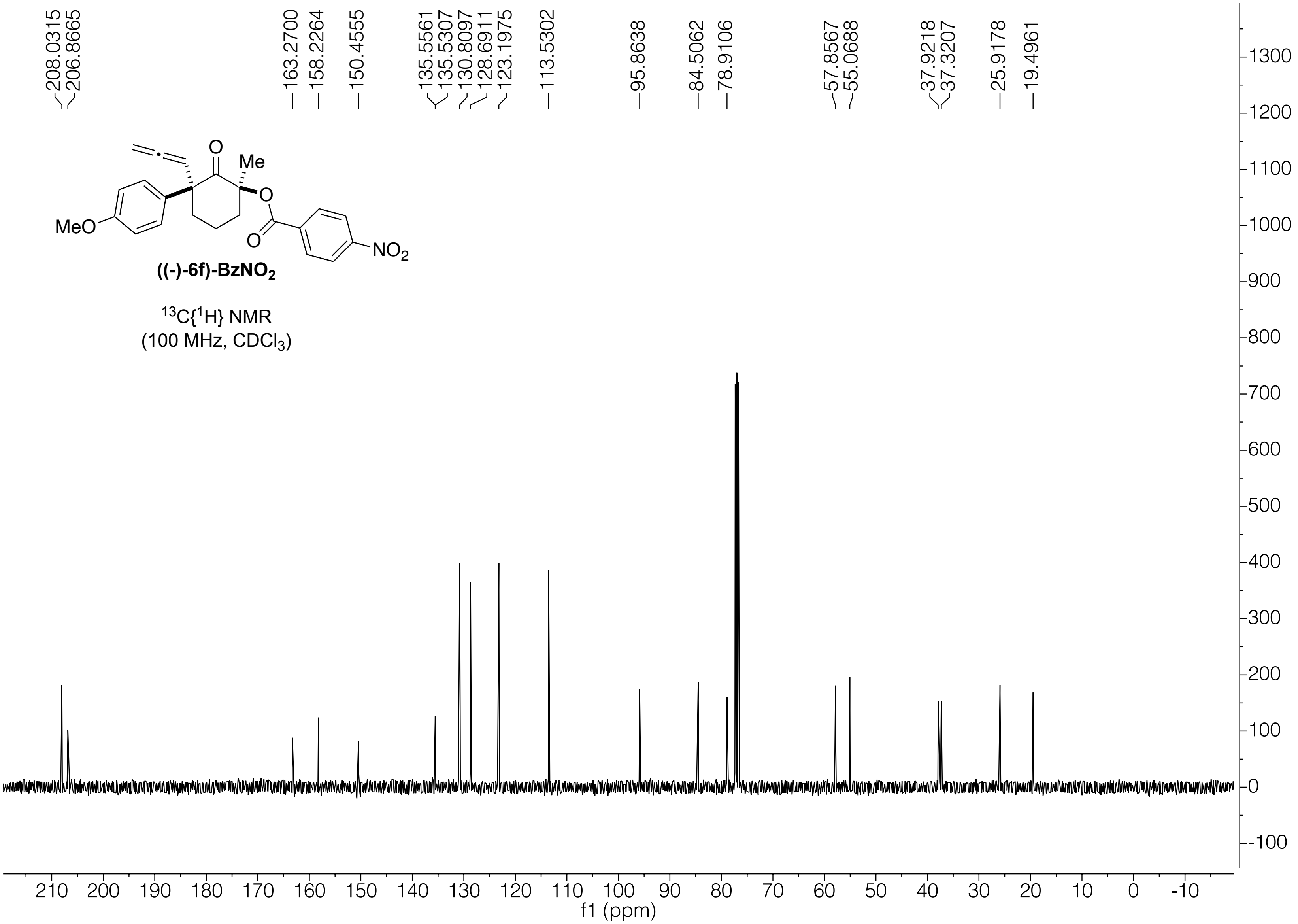
208.0315
206.8665
163.2700
158.2264
150.4555
135.5561
135.5307
130.8097
128.6911
123.1975
113.5302
95.8638
84.5062
78.9106
57.8567
55.0688
37.9218
37.3207
25.9178
19.4961
O
Me
MeO
O
O
NO_2
((-)-6f)-BzNO$_2$
$^{13}C\{^1H\}$ NMR
(100 MHz, $CDCl_3$)
f1 (ppm)

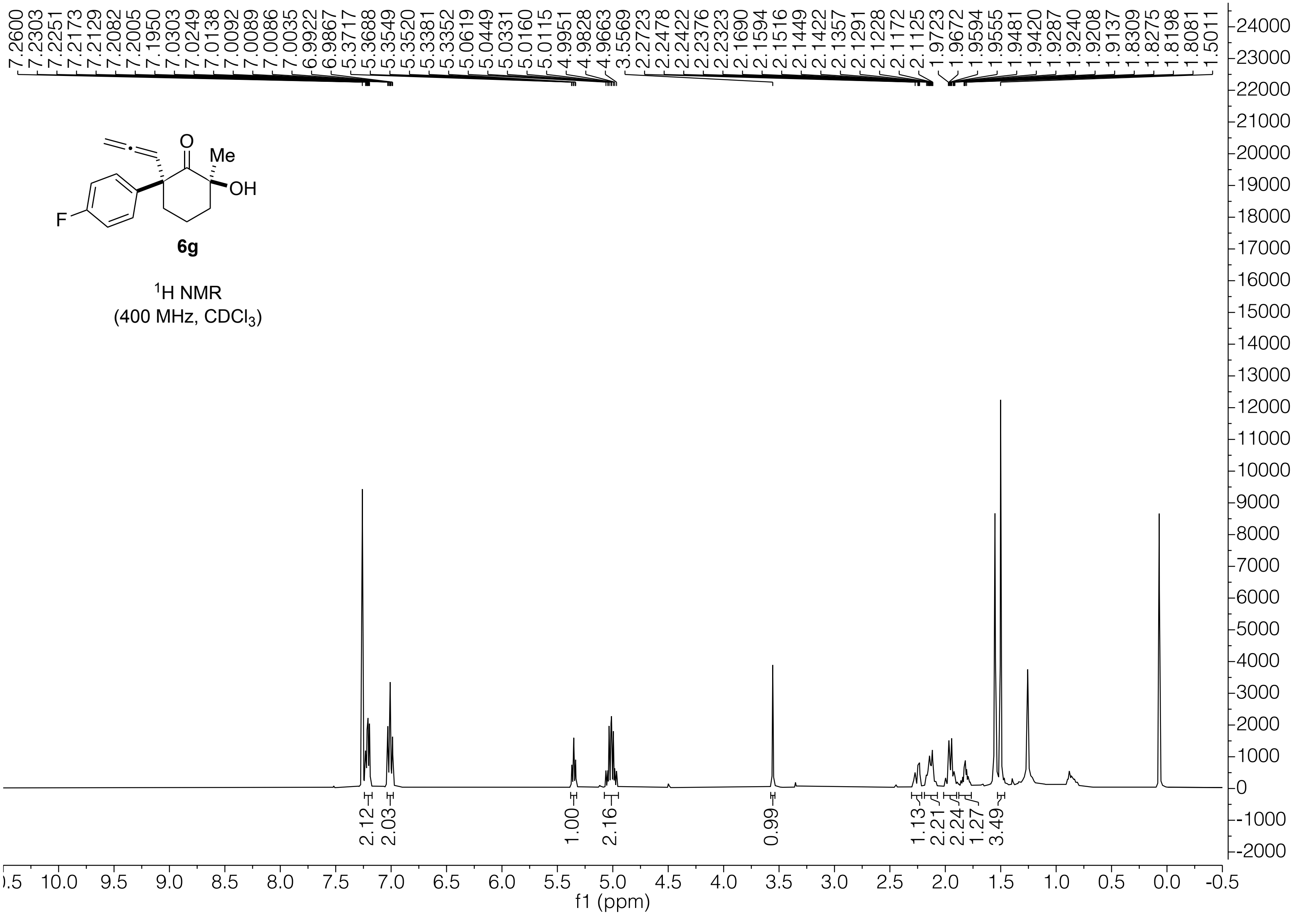

Me
OH
O
F
6g
1H NMR
(400 MHz, CDCl3)
f1 (ppm)

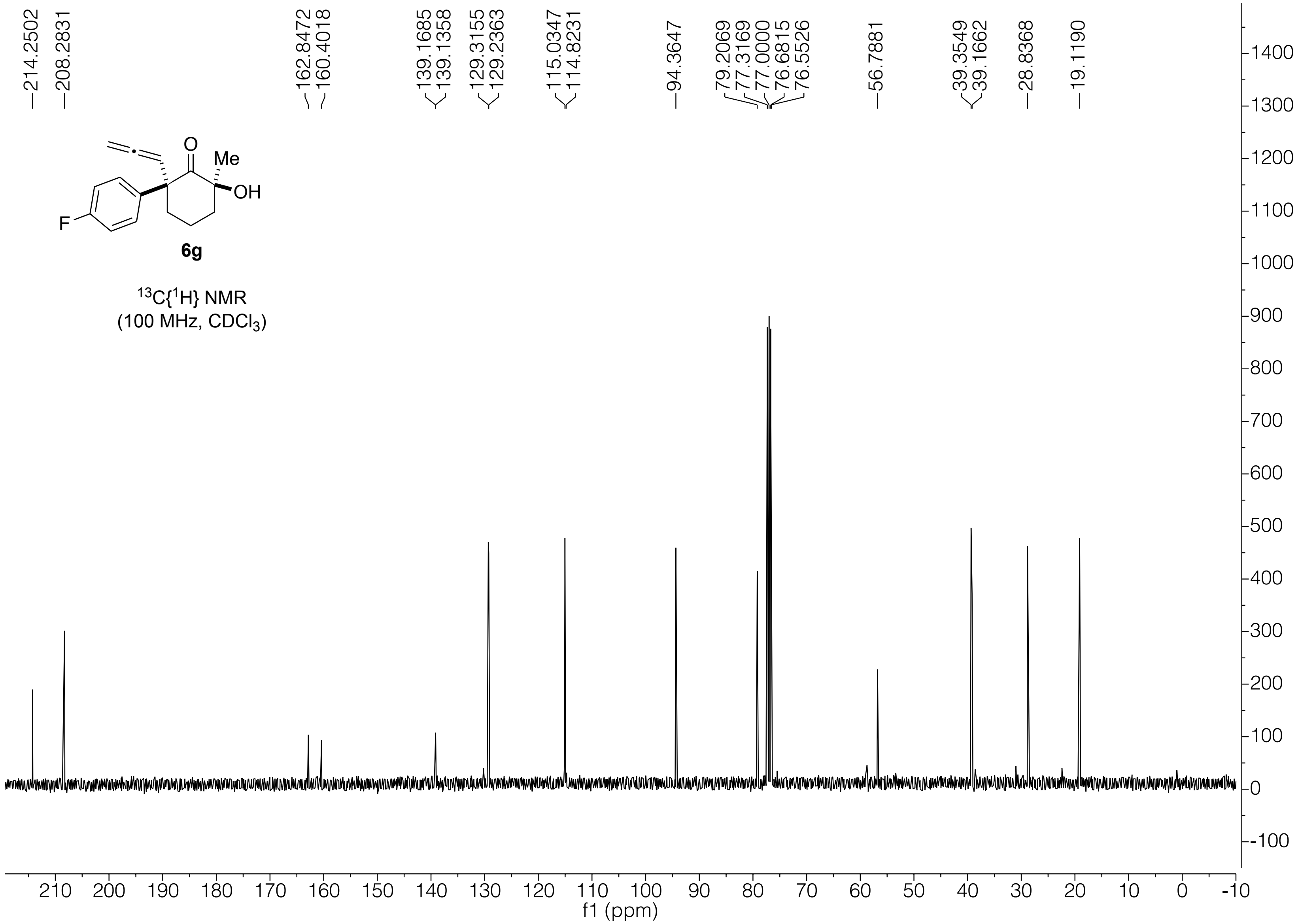

214.2502
208.2831
162.8472
160.4018
139.1685
139.1358
129.3155
129.2363
115.0347
114.8231
94.3647
79.2069
77.3169
77.0000
76.6815
76.5526
56.7881
39.3549
39.1662
28.8368
19.1190
O
Me
OH
F
6g
$^{13}C\{^{1}H\}$ NMR
(100 MHz, $CDCl_3$)
f1 (ppm)

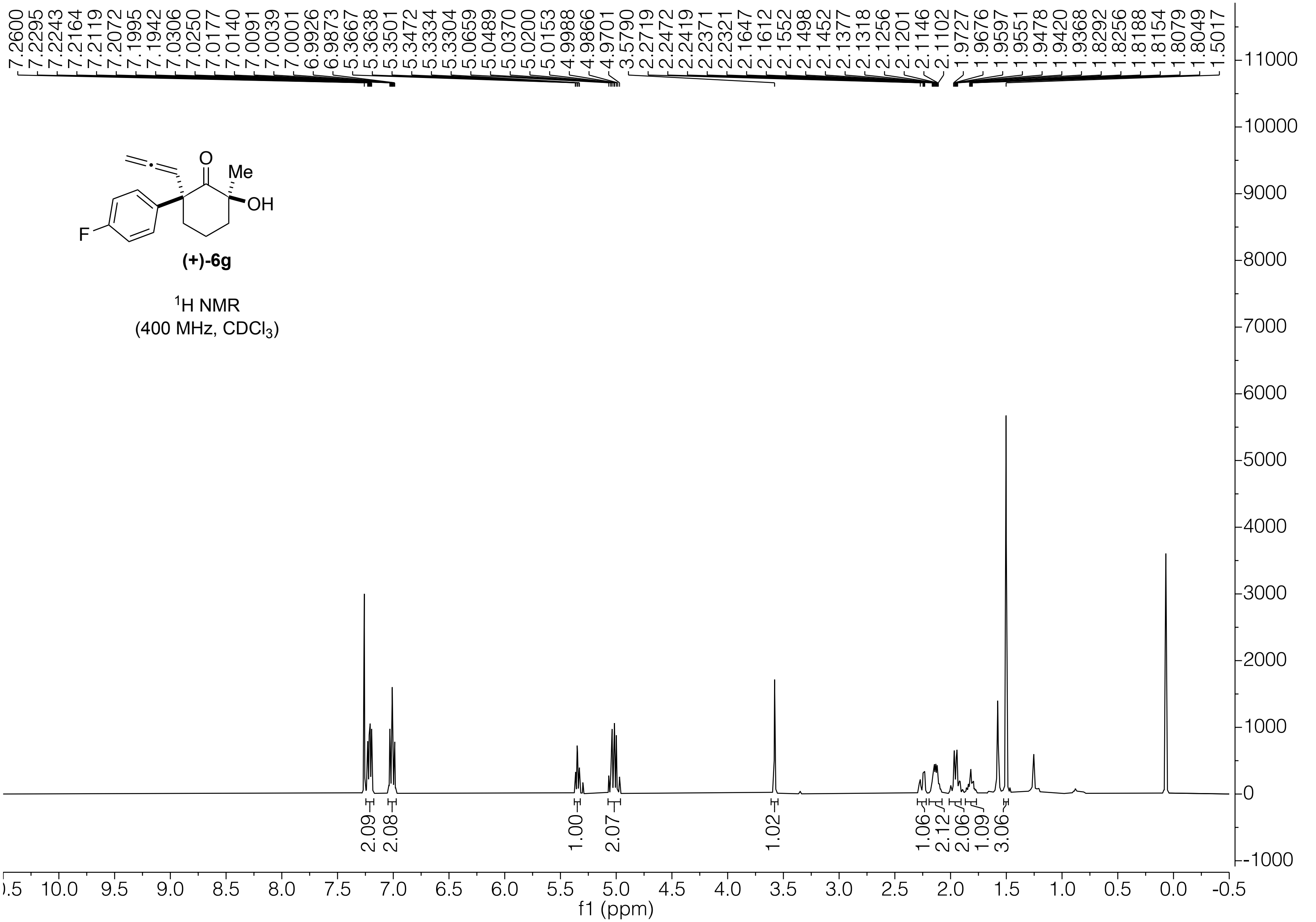

7.2600 7.2295 7.2243 7.2164 7.2119 7.2072 7.1995 7.1942 7.0306 7.0250 7.0177 7.0140 7.0091 7.0039 7.0001 6.9926 6.9873 5.3667 5.3638 5.3501 5.3472 5.3334 5.3304 5.0659 5.0489 5.0370 5.0200 5.0153 4.9988 4.9866 4.9701 3.5790 2.2719 2.2472 2.2419 2.2371 2.2321 2.1647 2.1612 2.1552 2.1498 2.1452 2.1377 2.1318 2.1256 2.1201 2.1146 2.1102 1.9727 1.9676 1.9597 1.9551 1.9478 1.9420 1.9368 1.8292 1.8256 1.8188 1.8154 1.8079 1.8049 1.5017
O
Me
OH
F
(+)-6g
^{1}H NMR
(400 MHz, CDCl$_3$)
2.09 2.08 1.00 2.07 1.02 1.06 2.12 2.06 1.09 3.06
f1 (ppm)

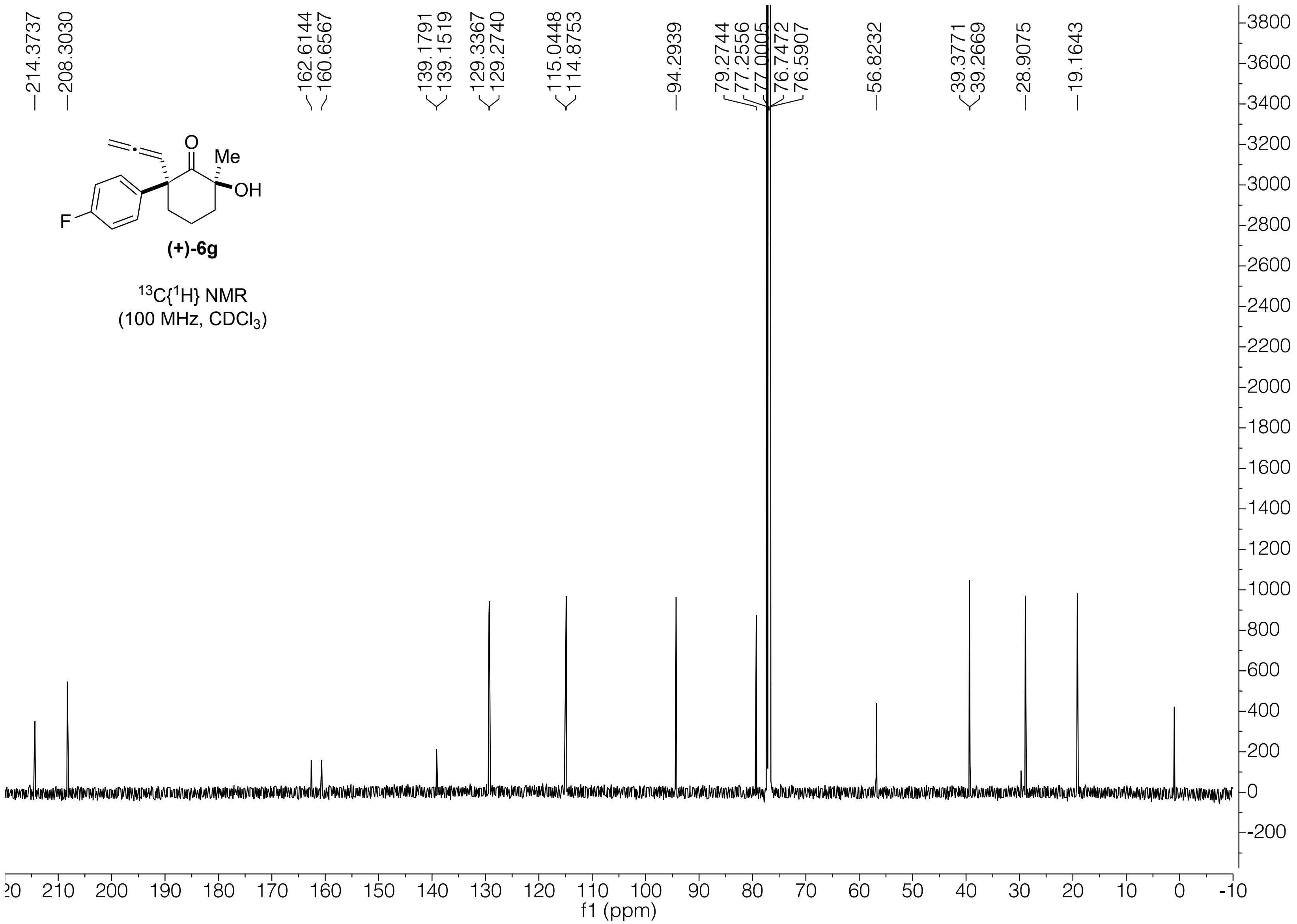

214.3737
208.3030
162.6144
160.6567
139.1791
139.1519
129.3367
129.2740
115.0448
114.8753
94.2939
79.2744
77.2556
77.0005
76.7472
76.5907
56.8232
39.3771
39.2669
28.9075
19.1643
O
Me
OH
F
(+)-6g
13C{1H} NMR
(100 MHz, CDCl3)
f1 (ppm)

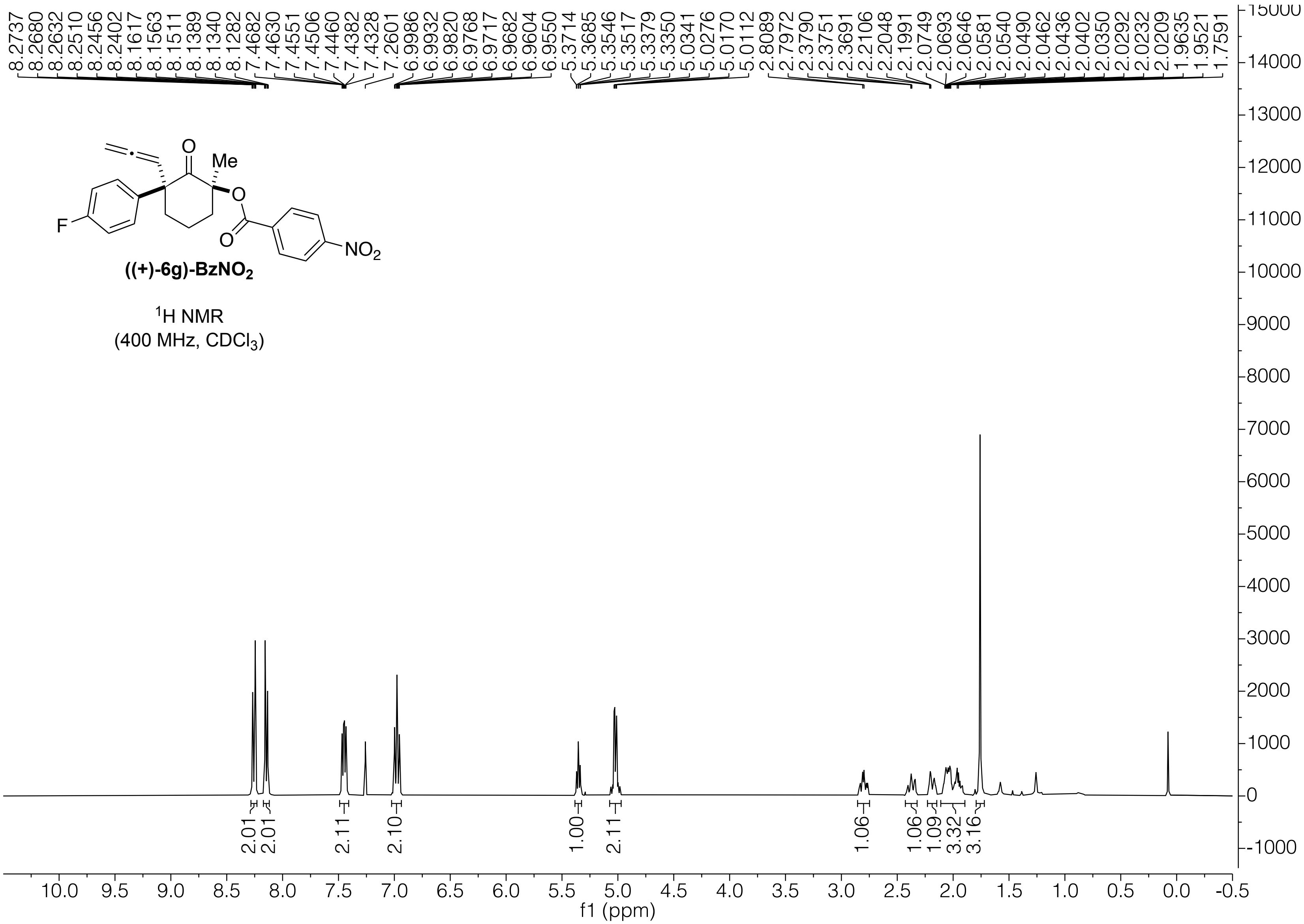

((+)-6g)-BzNO$_2$
^{1}H NMR
(400 MHz, CDCl$_3$)
Me
F
NO$_2$
8.2737
8.2680
8.2632
8.2510
8.2456
8.2402
8.1617
8.1563
8.1511
8.1389
8.1340
8.1282
7.4682
7.4630
7.4551
7.4506
7.4460
7.4382
7.4328
7.2601
6.9986
6.9932
6.9820
6.9768
6.9717
6.9682
6.9604
6.9550
5.3714
5.3685
5.3546
5.3517
5.3379
5.3350
5.0341
5.0276
5.0170
5.0112
2.8089
2.7972
2.3790
2.3751
2.3691
2.2106
2.2048
2.1991
2.0749
2.0693
2.0646
2.0581
2.0540
2.0490
2.0462
2.0436
2.0402
2.0350
2.0292
2.0232
2.0209
1.9635
1.9521
1.7591
2.01
2.01
2.11
2.10
1.00
2.11
1.06
1.06
1.09
3.32
3.16
f1 (ppm)

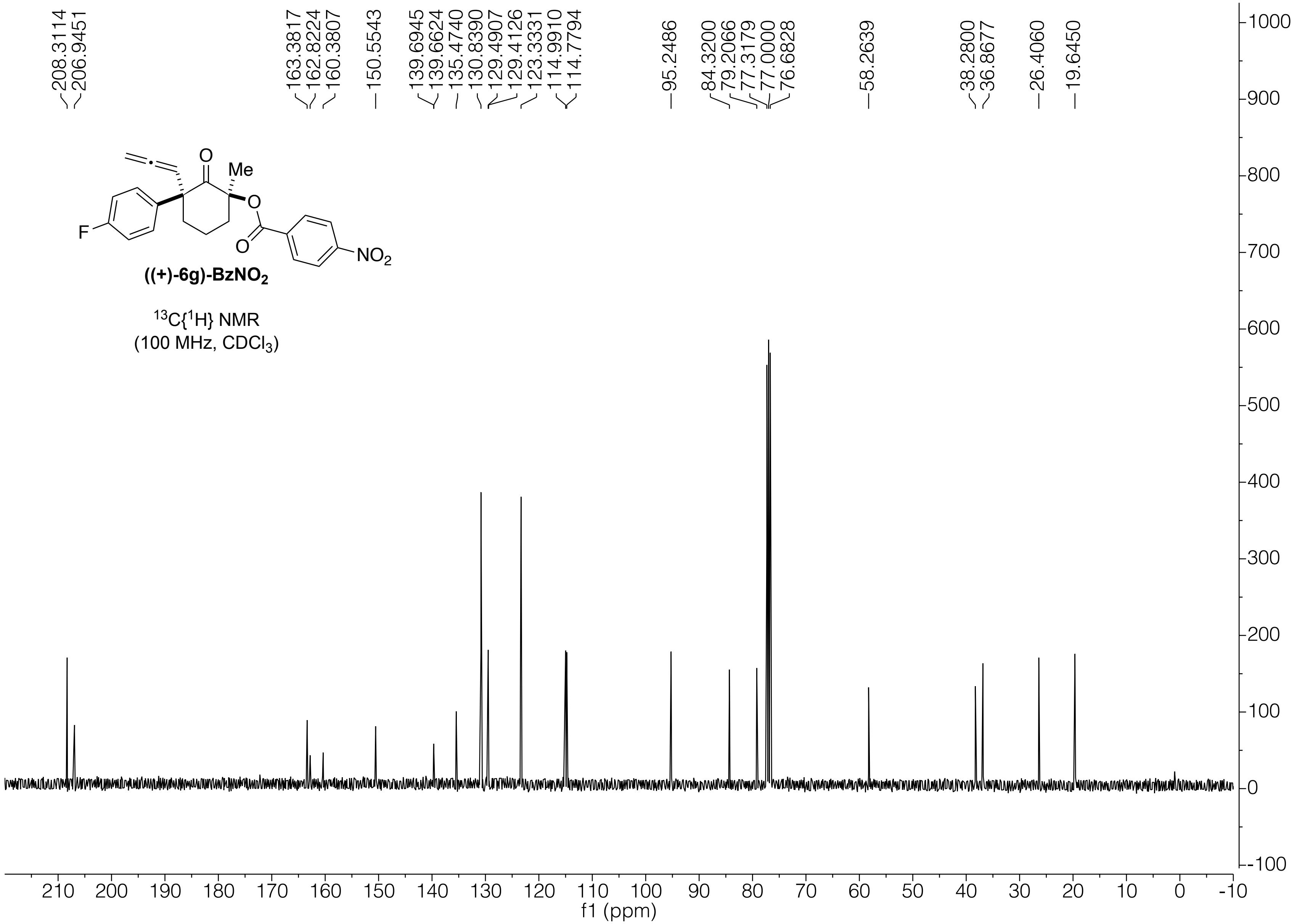

208.3114
206.9451
163.3817
162.8224
160.3807
150.5543
139.6945
139.6624
135.4740
130.8390
129.4907
129.4126
123.3331
114.9910
114.7794
95.2486
84.3200
79.2066
77.3179
77.0000
76.6828
58.2639
38.2800
36.8677
26.4060
19.6450
O
Me
O
F
O
NO_2
((+)-6g)-$BzNO_2$
$^{13}C\{^1H\}$ NMR
(100 MHz, $CDCl_3$)
f1 (ppm)

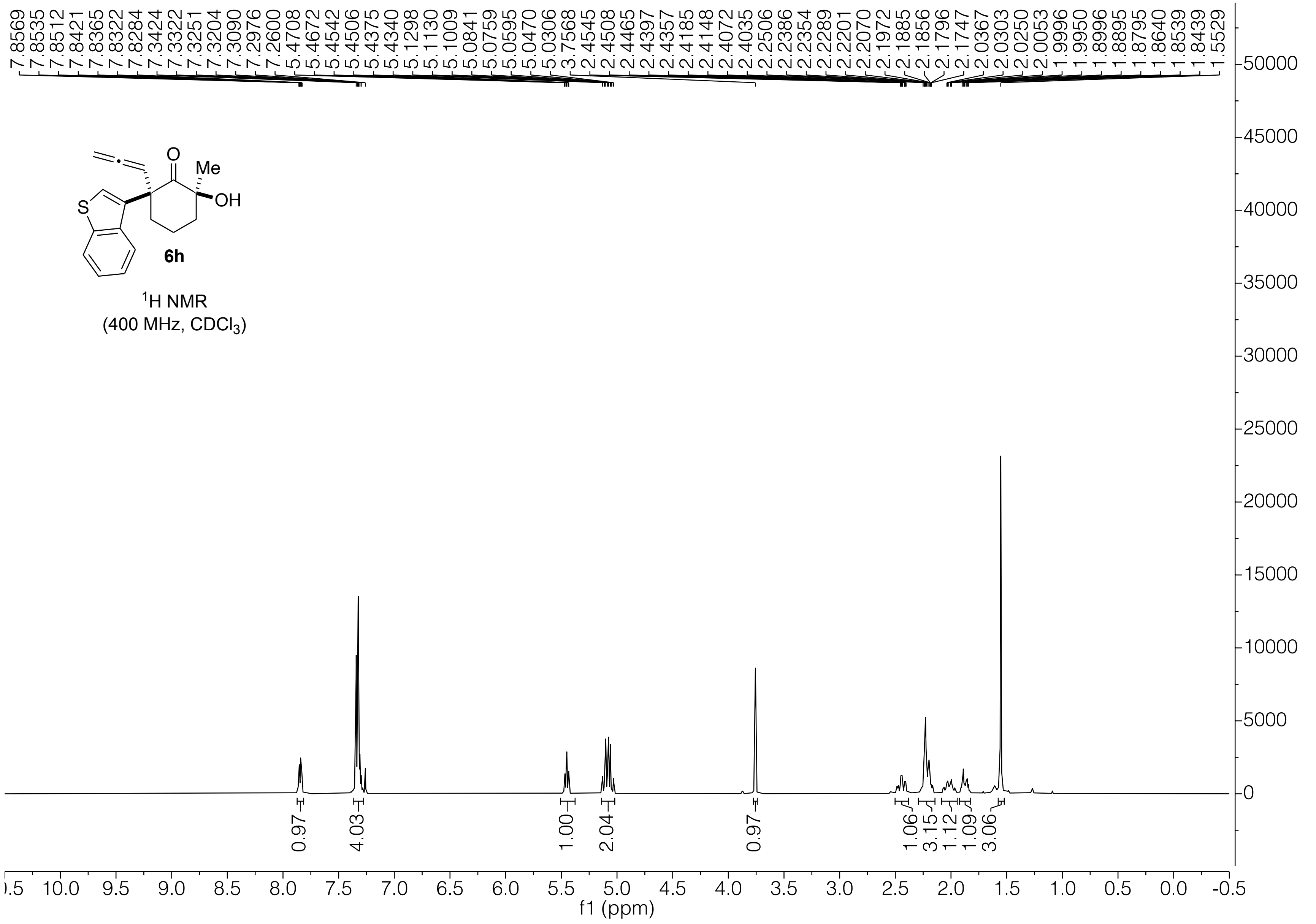

7.8569
7.8535
7.8512
7.8421
7.8365
7.8322
7.8284
7.3424
7.3322
7.3251
7.3204
7.3090
7.2976
7.2600
5.4708
5.4672
5.4542
5.4506
5.4375
5.4340
5.1298
5.1130
5.1009
5.0841
5.0759
5.0595
5.0470
5.0306
3.7568
2.4545
2.4508
2.4465
2.4397
2.4357
2.4185
2.4148
2.4072
2.4035
2.2506
2.2386
2.2354
2.2289
2.2201
2.2070
2.1972
2.1885
2.1856
2.1796
2.1747
2.0367
2.0303
2.0250
2.0053
1.9996
1.9950
1.8996
1.8895
1.8795
1.8640
1.8539
1.8439
1.5529
O
Me
OH
S
6h
^{1}H NMR
(400 MHz, $CDCl_3$)
0.97
4.03
1.00
2.04
0.97
1.06
3.15
1.12
1.09
3.06
50000
45000
40000
35000
30000
25000
20000
15000
10000
5000
0
10.0
9.5
9.0
8.5
8.0
7.5
7.0
6.5
6.0
5.5
5.0
4.5
4.0
3.5
3.0
2.5
2.0
1.5
1.0
0.5
0.0
-0.5
f1 (ppm)

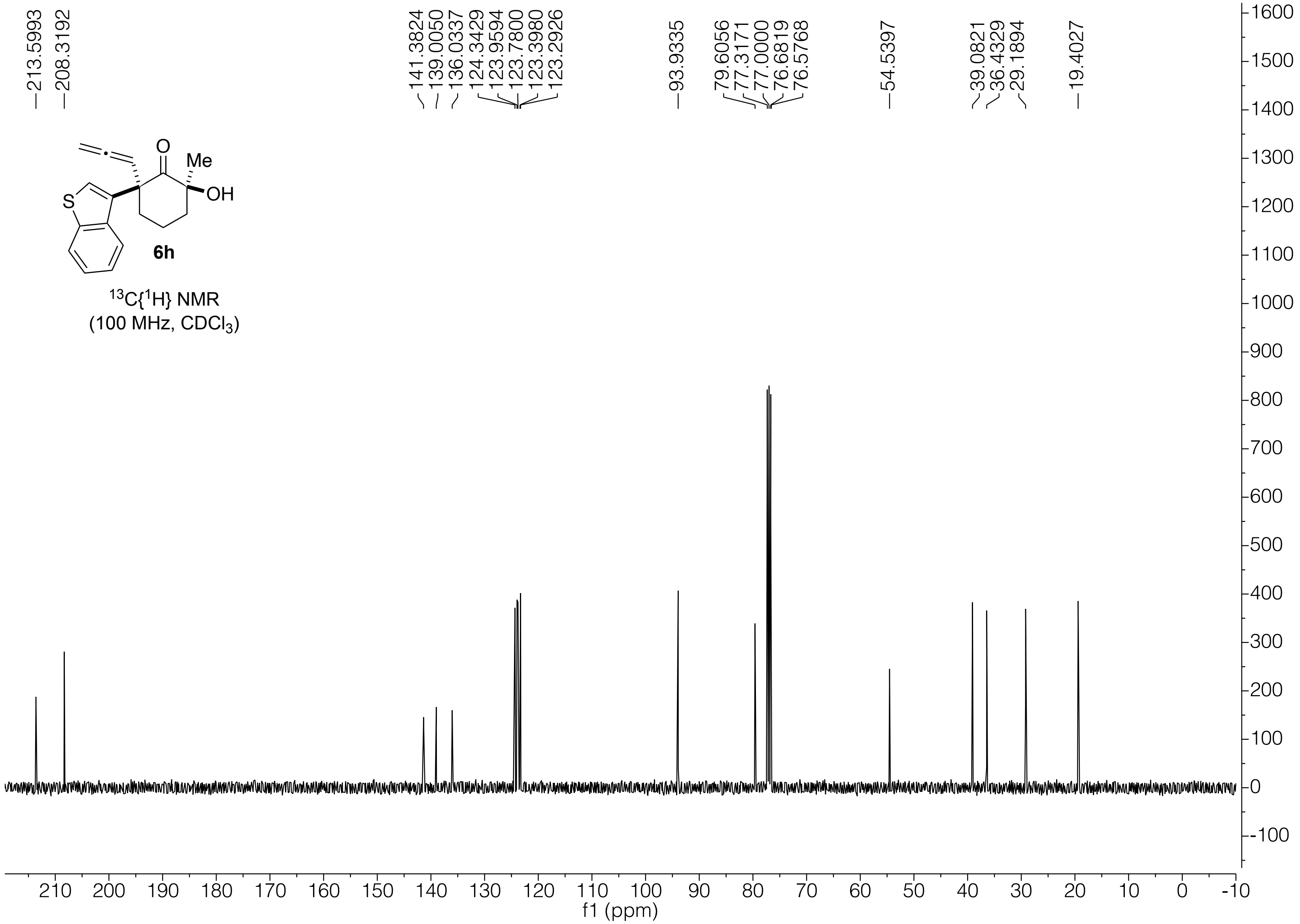

213.5993
208.3192
141.3824
139.0050
136.0337
124.3429
123.9594
123.7800
123.3980
123.2926
93.9335
79.6056
77.3171
77.0000
76.6819
76.5768
54.5397
39.0821
36.4329
29.1894
19.4027
O
Me
OH
S
6h
$^{13}C\{^1H\}$ NMR
(100 MHz, $CDCl_3$)
f1 (ppm)

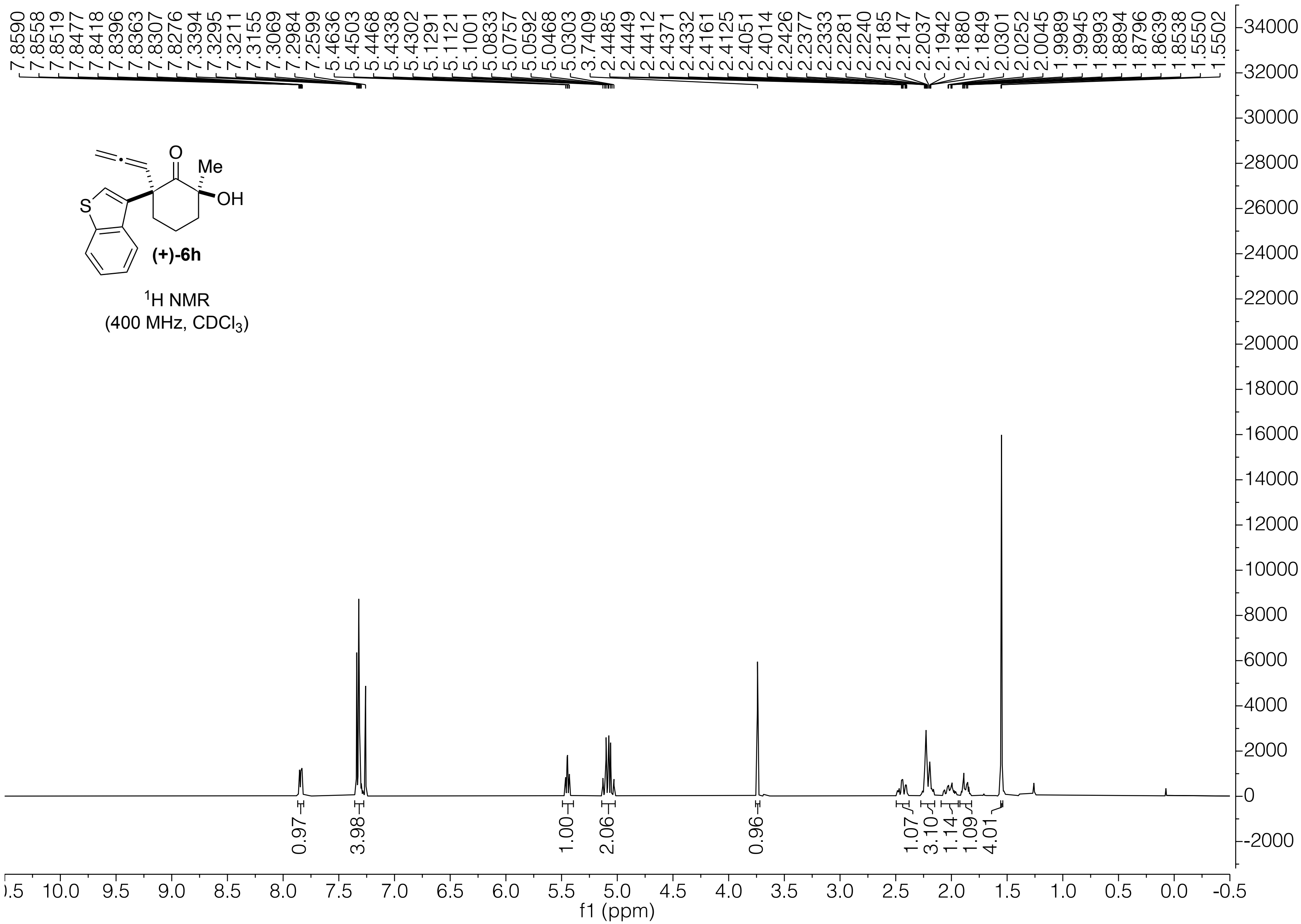
7.8590
7.8558
7.8519
7.8477
7.8418
7.8396
7.8363
7.8307
7.8276
7.3394
7.3295
7.3211
7.3155
7.3069
7.2984
7.2599
5.4636
5.4503
5.4468
5.4338
5.4302
5.1291
5.1121
5.1001
5.0833
5.0757
5.0592
5.0468
5.0303
3.7409
2.4485
2.4449
2.4412
2.4371
2.4332
2.4161
2.4125
2.4051
2.4014
2.2426
2.2377
2.2333
2.2281
2.2240
2.2185
2.2147
2.2037
2.1942
2.1880
2.1849
2.0301
2.0252
2.0045
1.9989
1.9945
1.8993
1.8894
1.8796
1.8639
1.8538
1.5550
1.5502
Me
OH
O
S
(+)-6h
^{1}H NMR
(400 MHz, $CDCl_3$)
0.97
3.98
1.00
2.06
0.96
1.07
3.10
1.14
1.09
4.01
f1 (ppm)

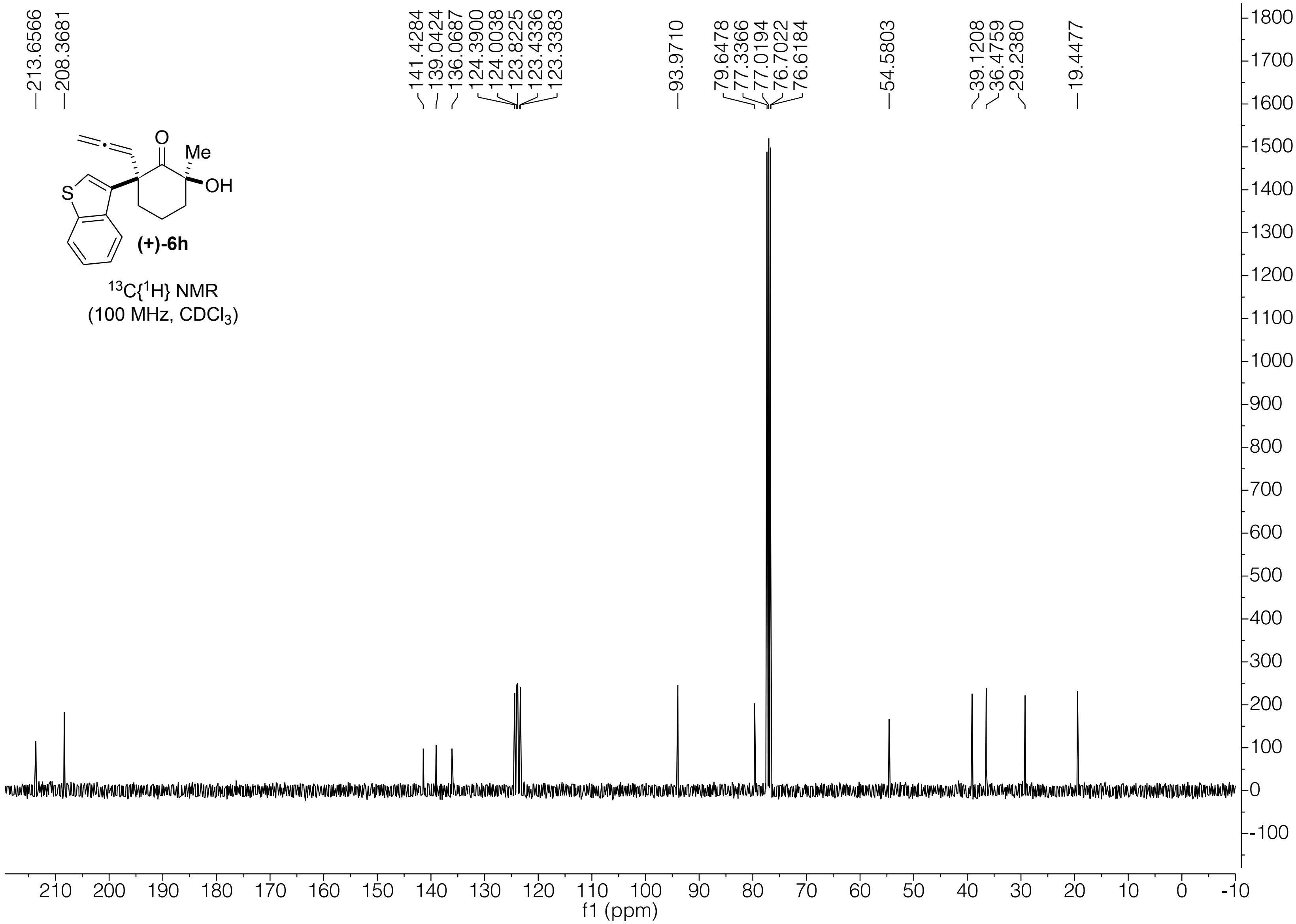

213.6566
208.3681
141.4284
139.0424
136.0687
124.3900
124.0038
123.8225
123.4336
123.3383
93.9710
79.6478
77.3366
77.0194
76.7022
76.6184
54.5803
39.1208
36.4759
29.2380
19.4477
O
Me
OH
S
(+)-6h
$^{13}C\{^{1}H\}$ NMR
(100 MHz, $CDCl_3$)
f1 (ppm)

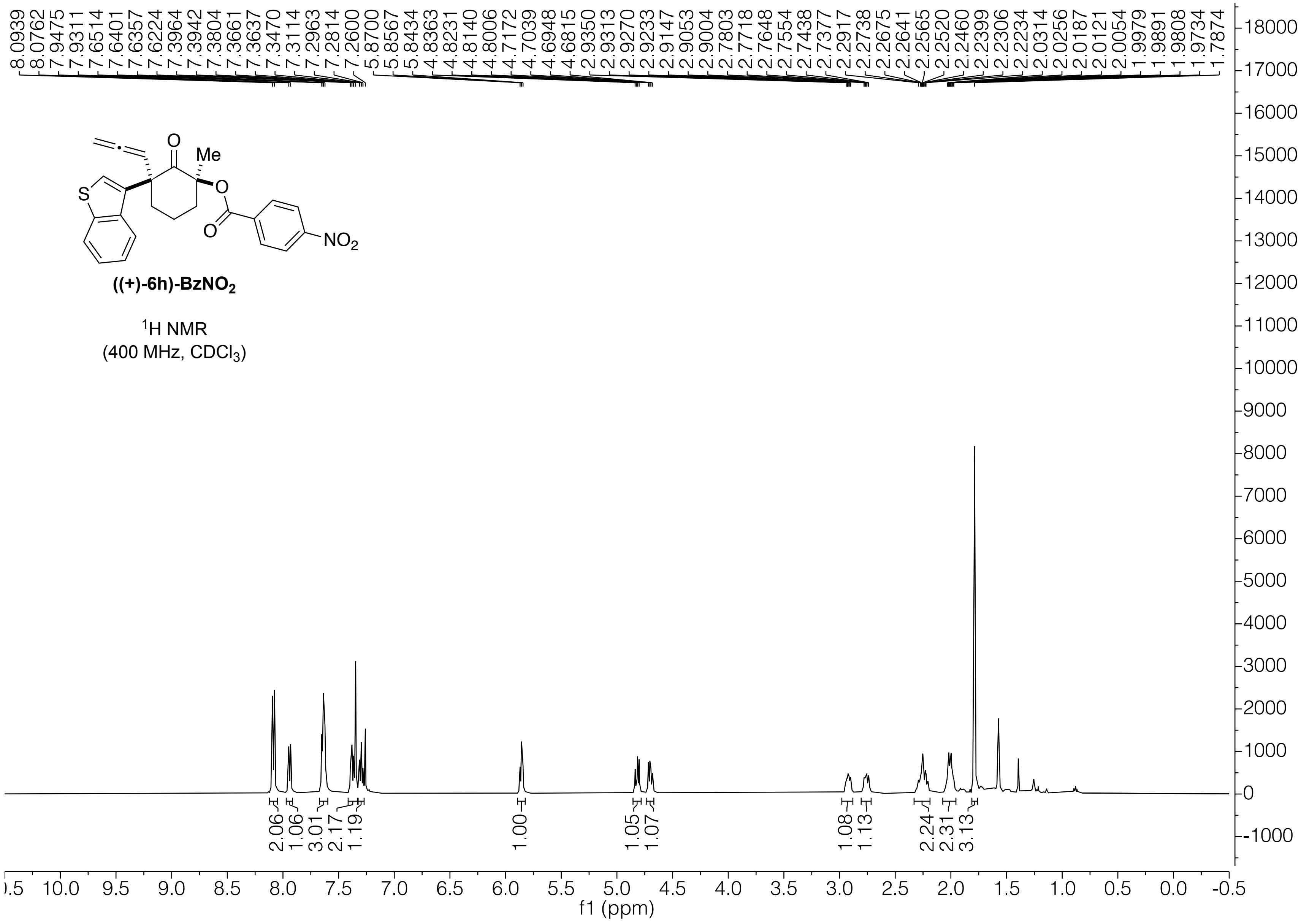

((+)-6h)-BzNO2
1H NMR
(400 MHz, CDCl3)
Me
O
S
NO2
f1 (ppm)

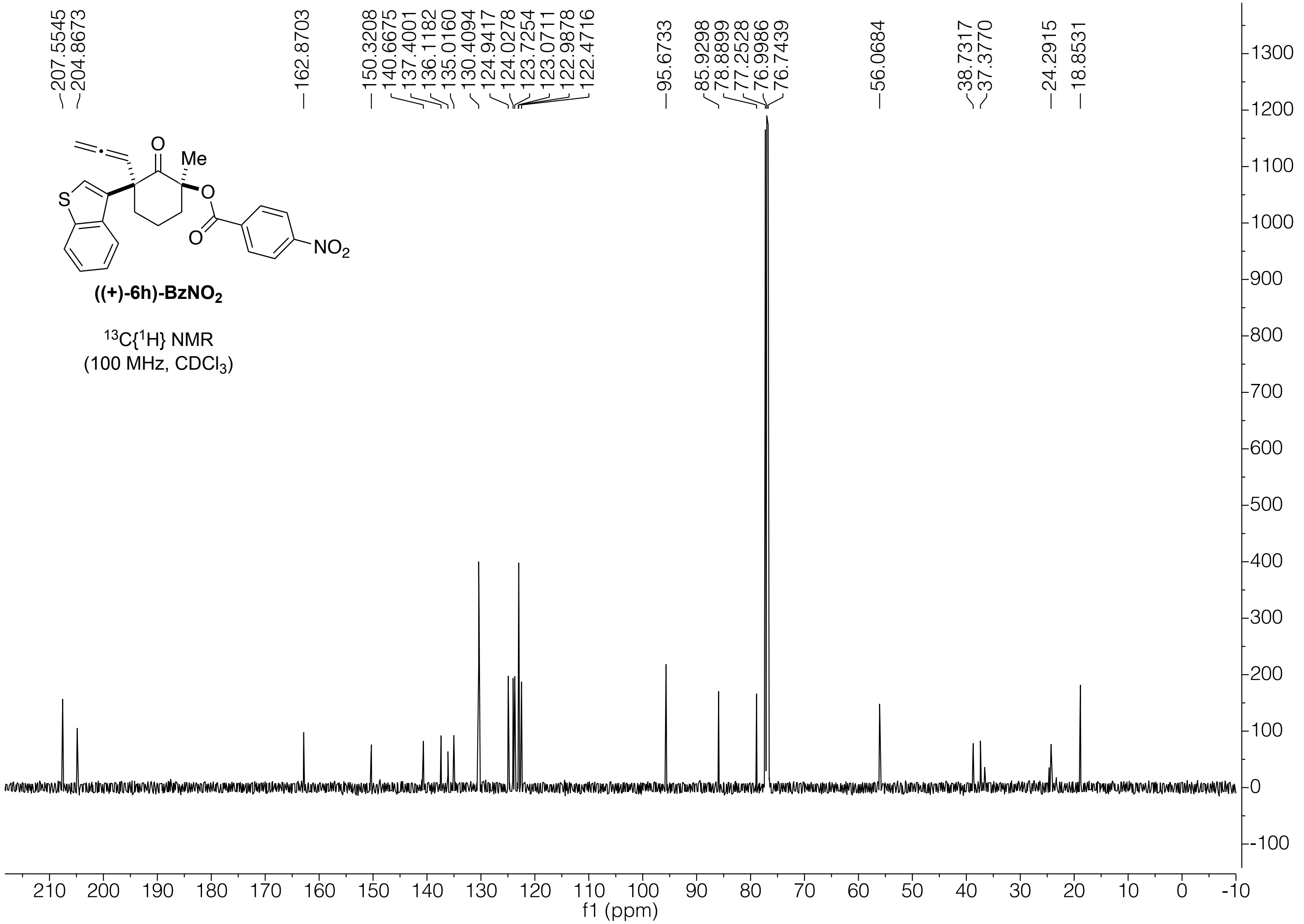
207.5545
204.8673
162.8703
150.3208
140.6675
137.4001
136.1182
135.0160
130.4094
124.9417
124.0278
123.7254
123.0711
122.9878
122.4716
95.6733
85.9298
78.8899
77.2528
76.9986
76.7439
56.0684
38.7317
37.3770
24.2915
18.8531
O
Me
S
O
O
NO2
((+)-6h)-BzNO2
13C{1H} NMR
(100 MHz, CDCl3)
f1 (ppm)

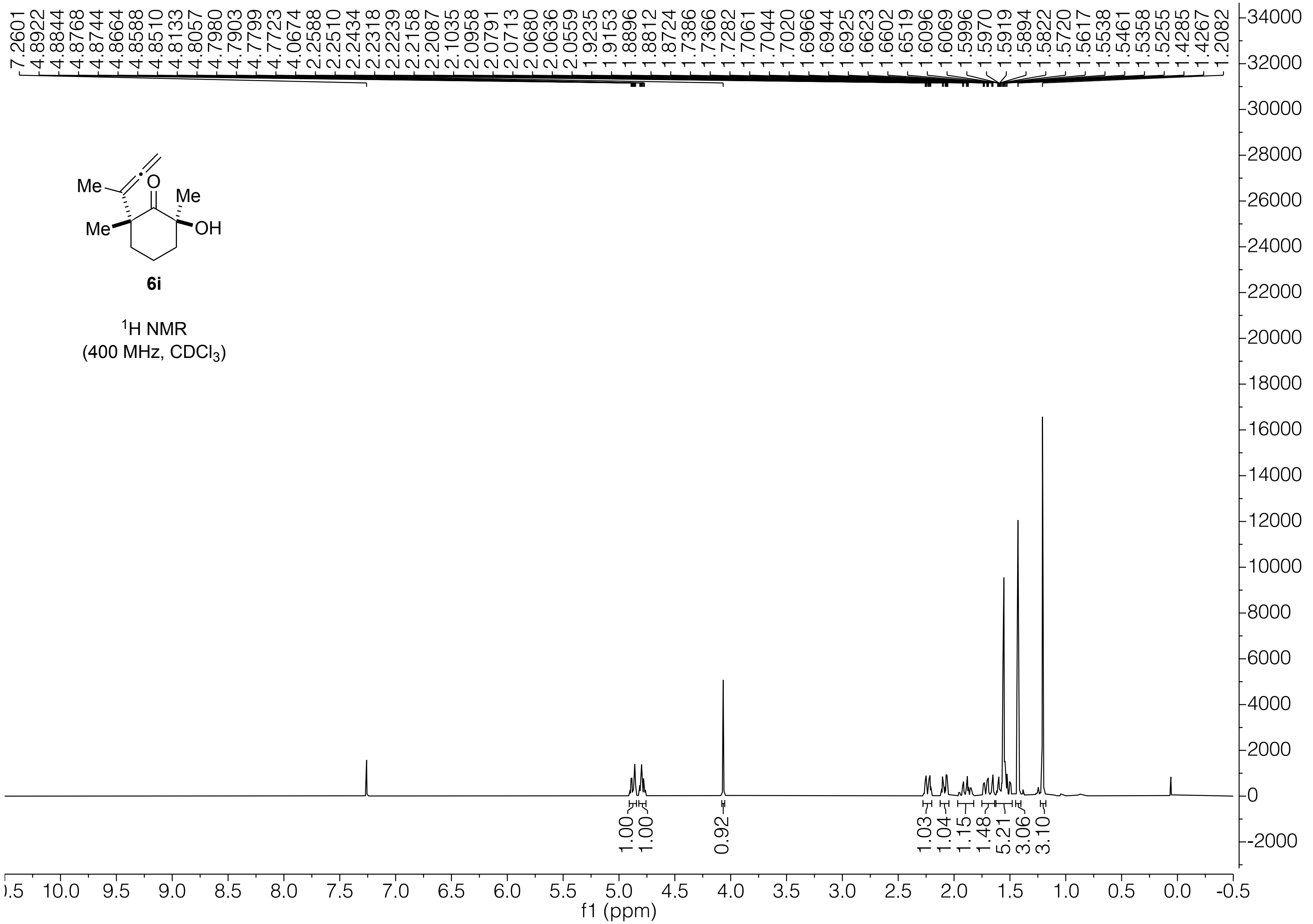

Me
O
Me
Me
OH
6i
1H NMR
(400 MHz, CDCl3)
f1 (ppm)

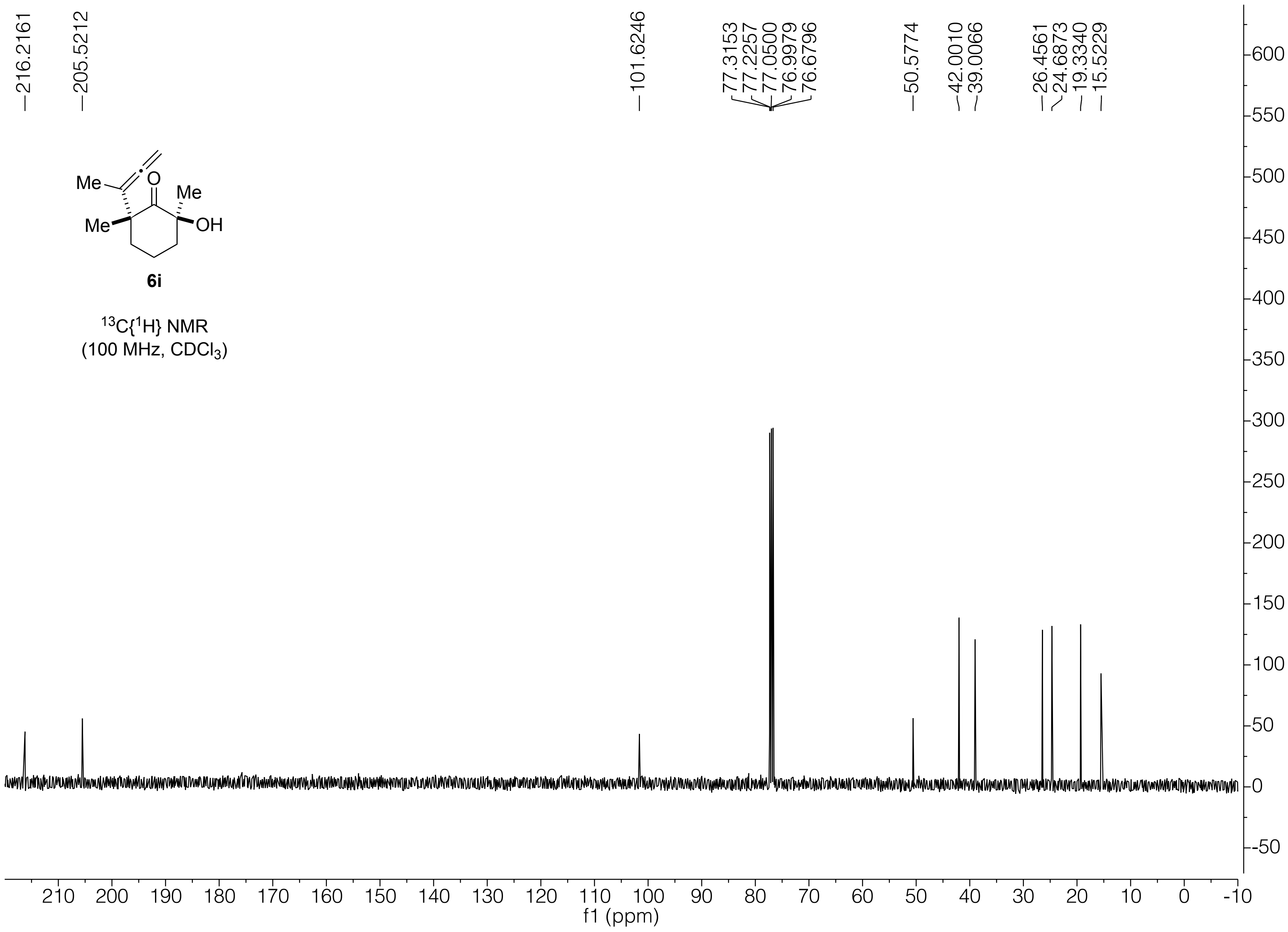

216.2161
205.5212
101.6246
77.3153
77.2257
77.0500
76.9979
76.6796
50.5774
42.0010
39.0066
26.4561
24.6873
19.3340
15.5229
Me
O
Me
Me
OH
6i
$^{13}C\{^1H\}$ NMR
(100 MHz, $CDCl_3$)
f1 (ppm)

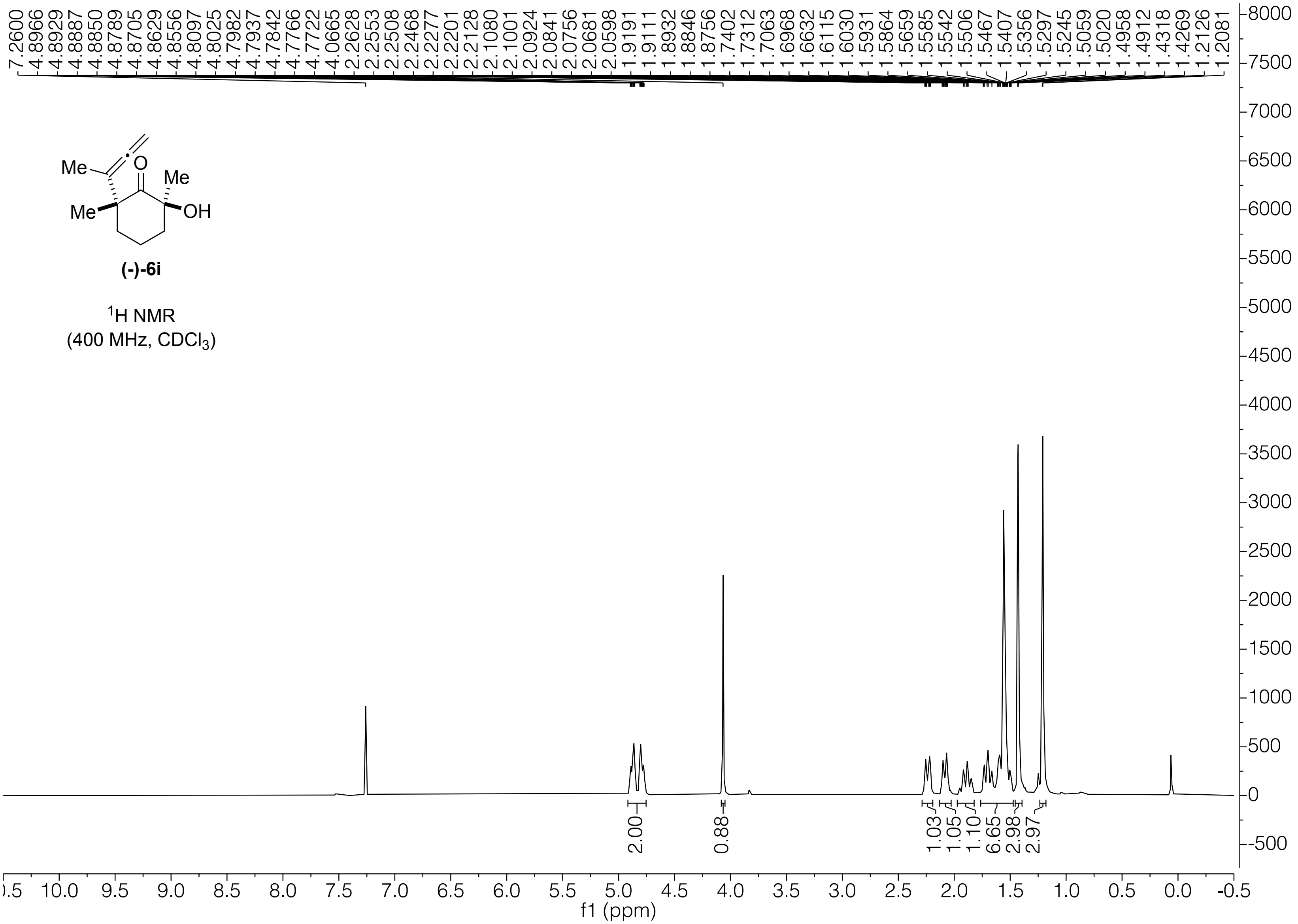

Me
O
Me
Me
OH
(-)-6i
^{1}H NMR
(400 MHz, $CDCl_3$)
f1 (ppm)

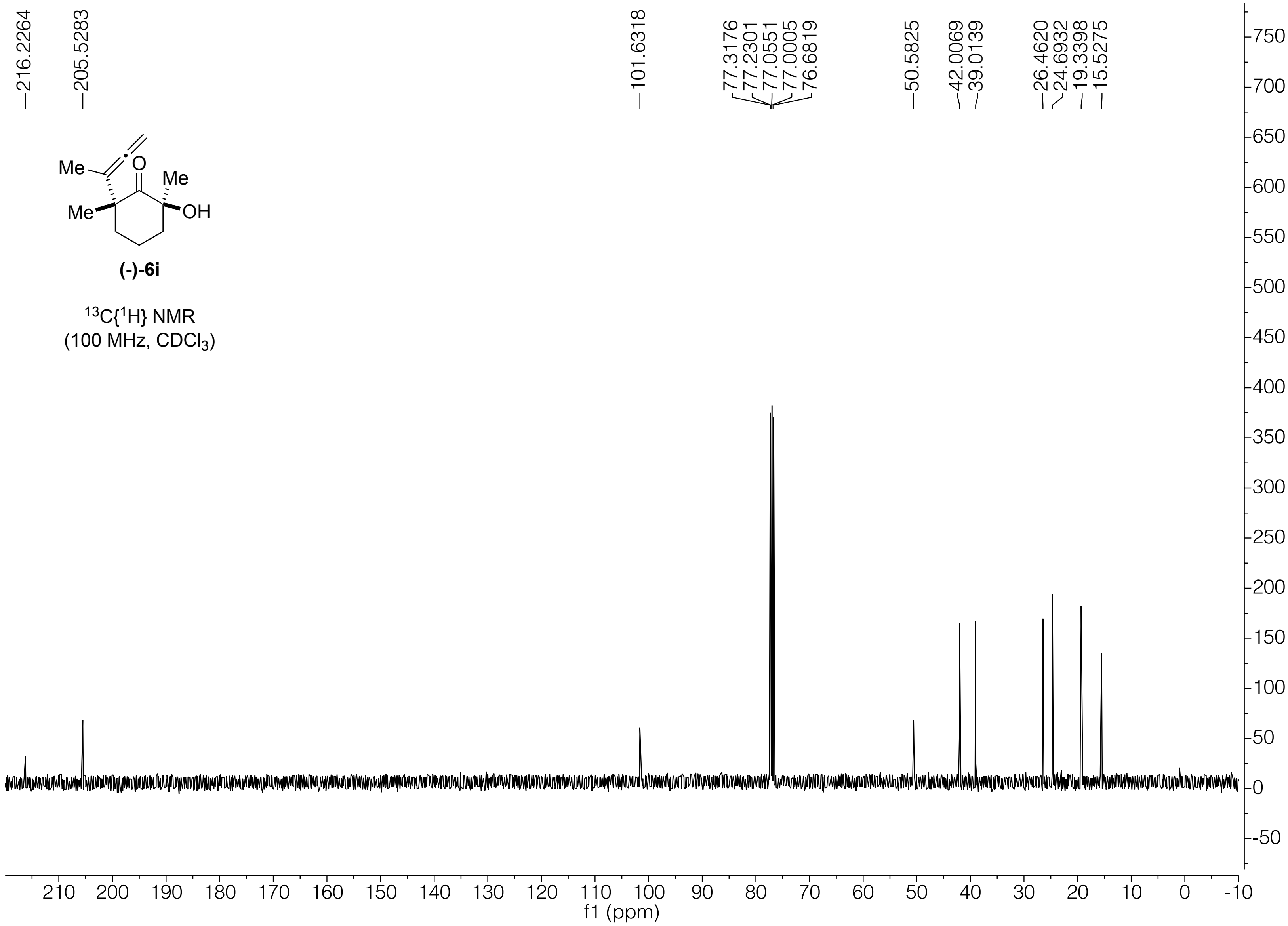

216.2264
205.5283
101.6318
77.3176
77.2301
77.0551
77.0005
76.6819
50.5825
42.0069
39.0139
26.4620
24.6932
19.3398
15.5275
Me
O
Me
Me
OH
(-)-6i
13C{1H} NMR
(100 MHz, CDCl3)
f1 (ppm)

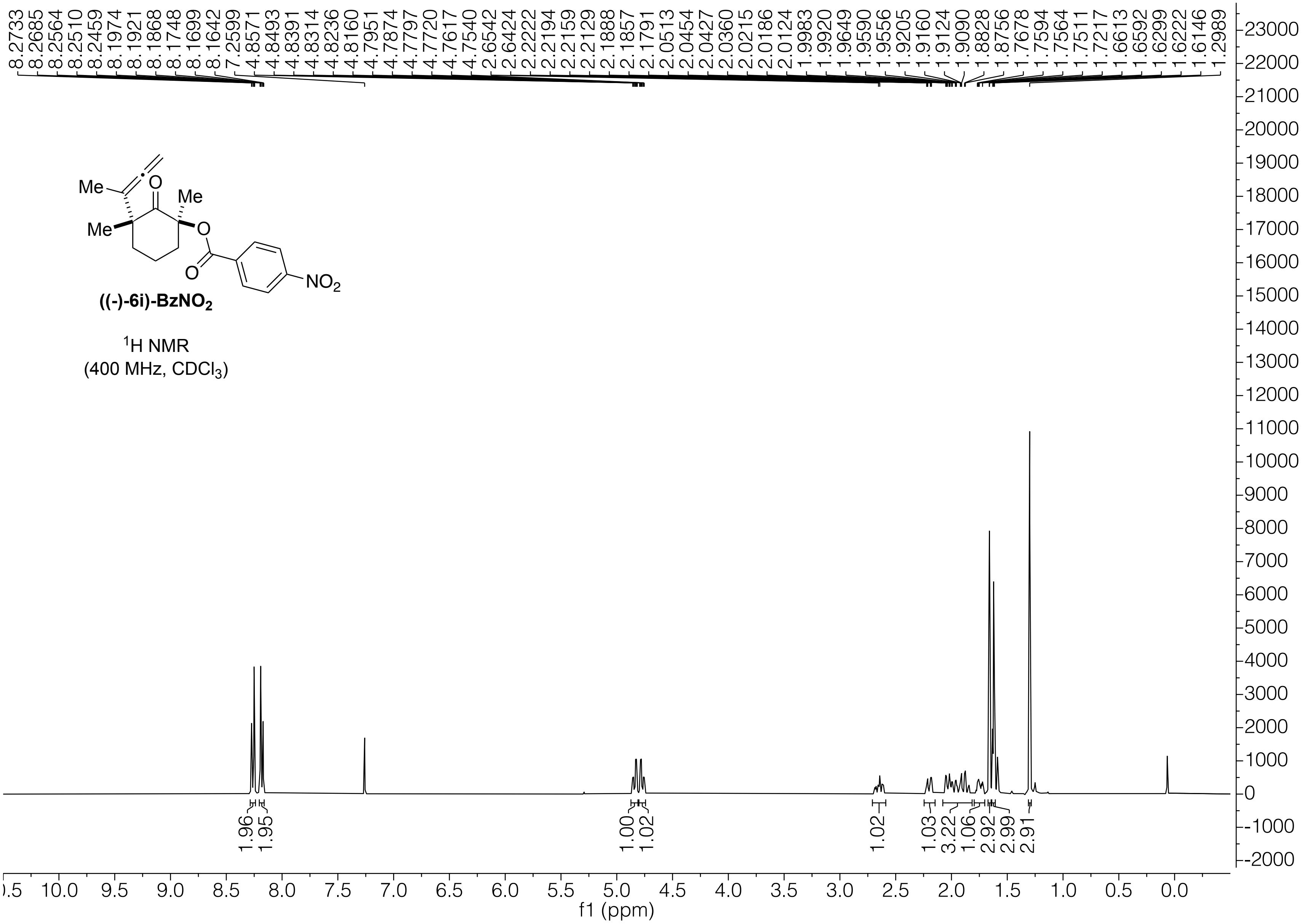
((-)-6i)-BzNO2
1H NMR
(400 MHz, CDCl3)
f1 (ppm)

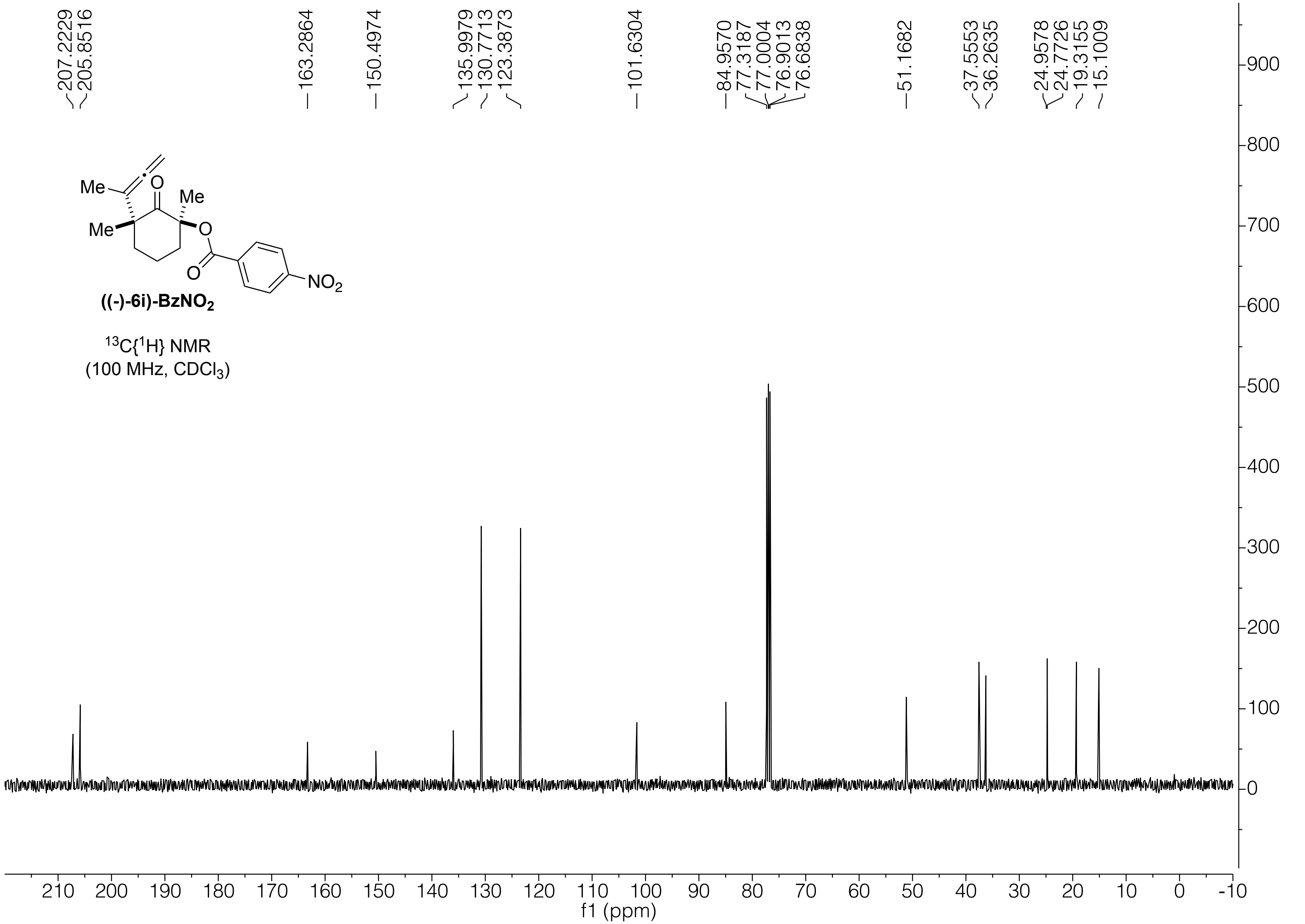
207.2229
205.8516
163.2864
150.4974
135.9979
130.7713
123.3873
101.6304
84.9570
77.3187
77.0004
76.9013
76.6838
51.1682
37.5553
36.2635
24.9578
24.7726
19.3155
15.1009
Me
O
Me
Me
O
O
NO2
((-)-6i)-BzNO2
13C{1H} NMR
(100 MHz, CDCl3)
900
800
700
600
500
400
300
200
100
0
210 200 190 180 170 160 150 140 130 120 110 100 90 80 70 60 50 40 30 20 10 0 -10
f1 (ppm)

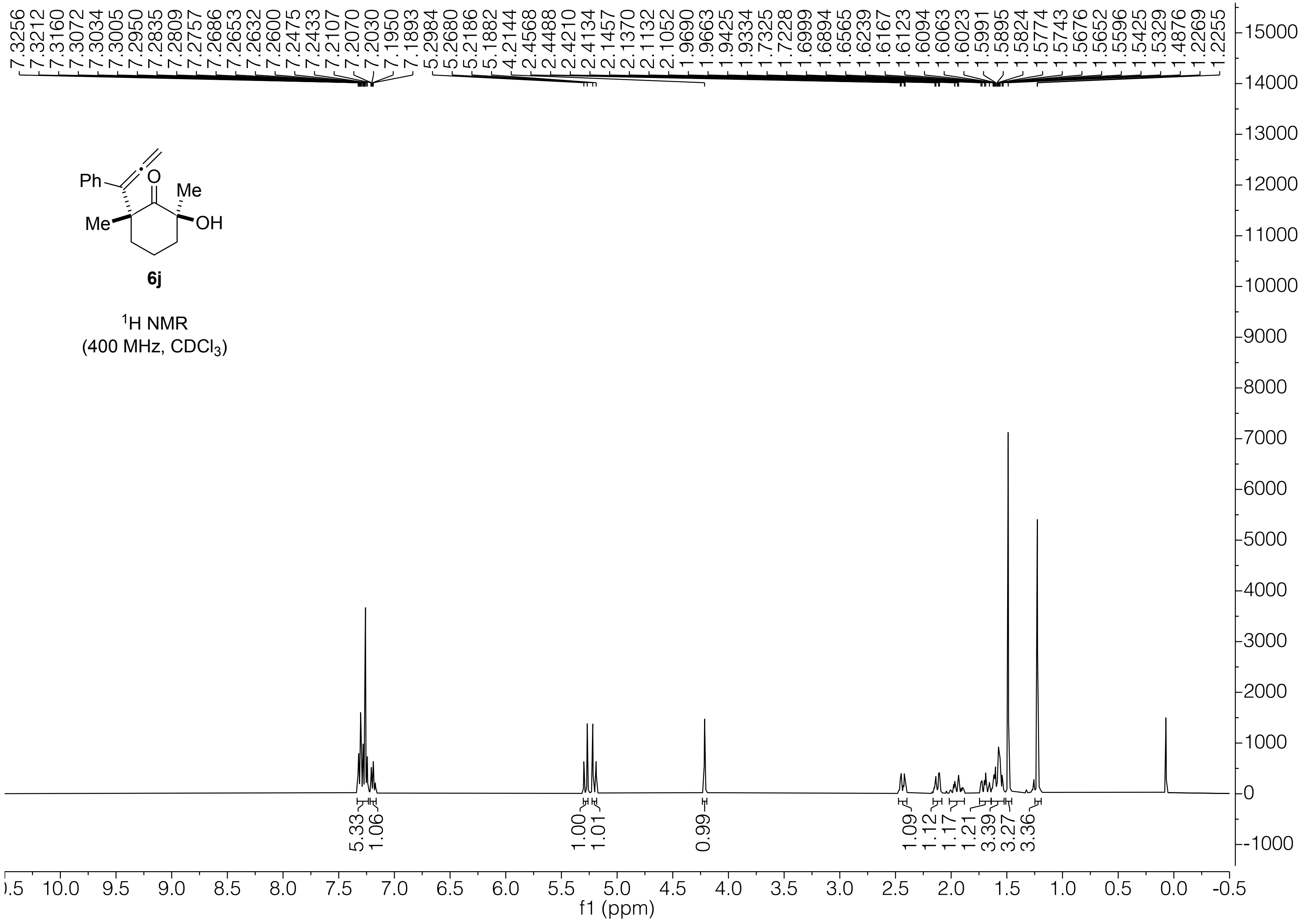
Ph
O
Me
Me
OH
6j
1H NMR
(400 MHz, CDCl3)
f1 (ppm)
5.33
1.06
1.00
1.01
0.99
1.09
1.12
1.17
1.21
3.39
3.27
3.36

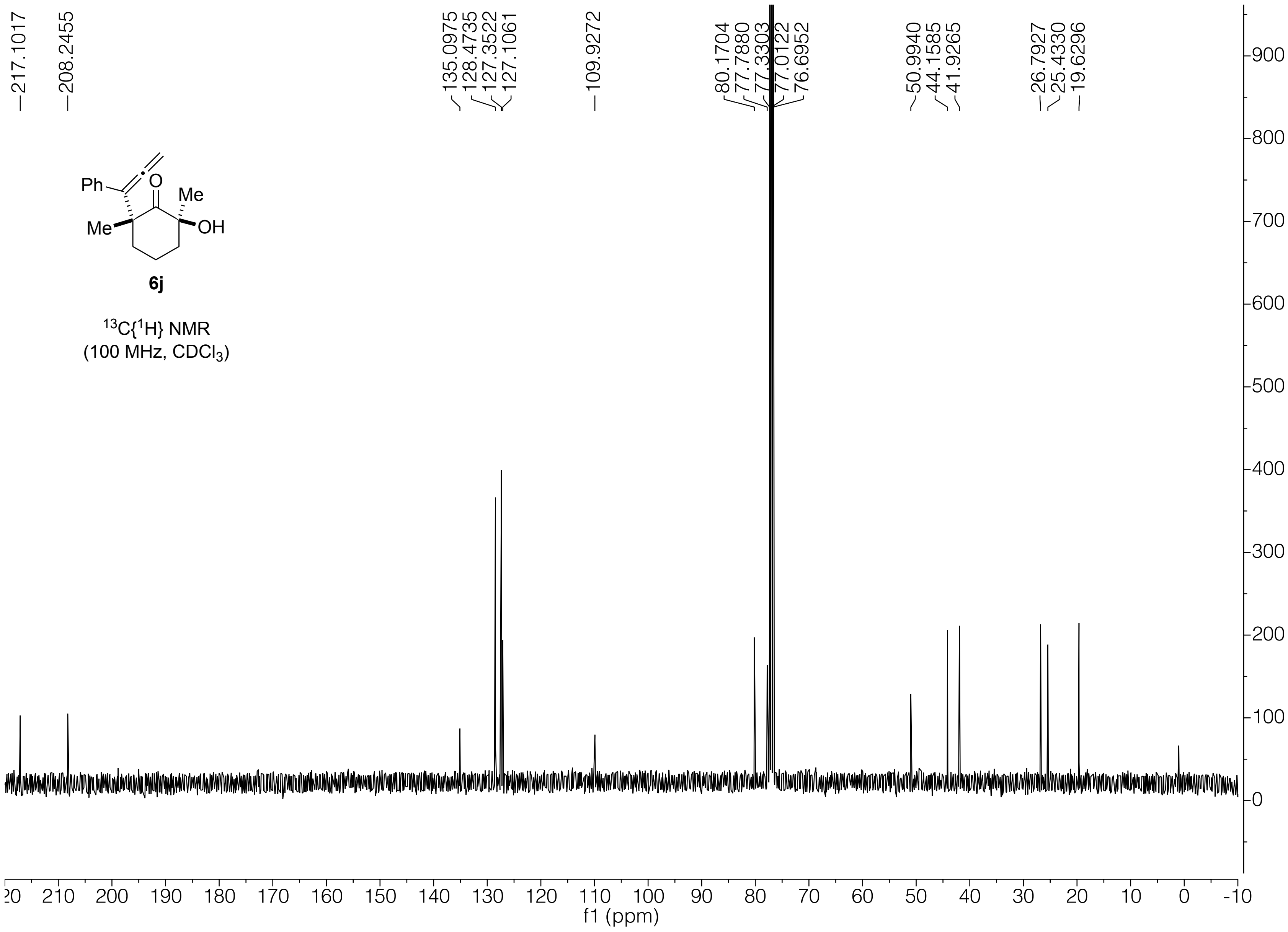

217.1017
208.2455
135.0975
128.4735
127.3522
127.1061
109.9272
80.1704
77.7880
77.3303
77.0122
76.6952
50.9940
44.1585
41.9265
26.7927
25.4330
19.6296
Ph
O
Me
Me
OH
6j
$^{13}C\{^1H\}$ NMR
(100 MHz, $CDCl_3$)
f1 (ppm)

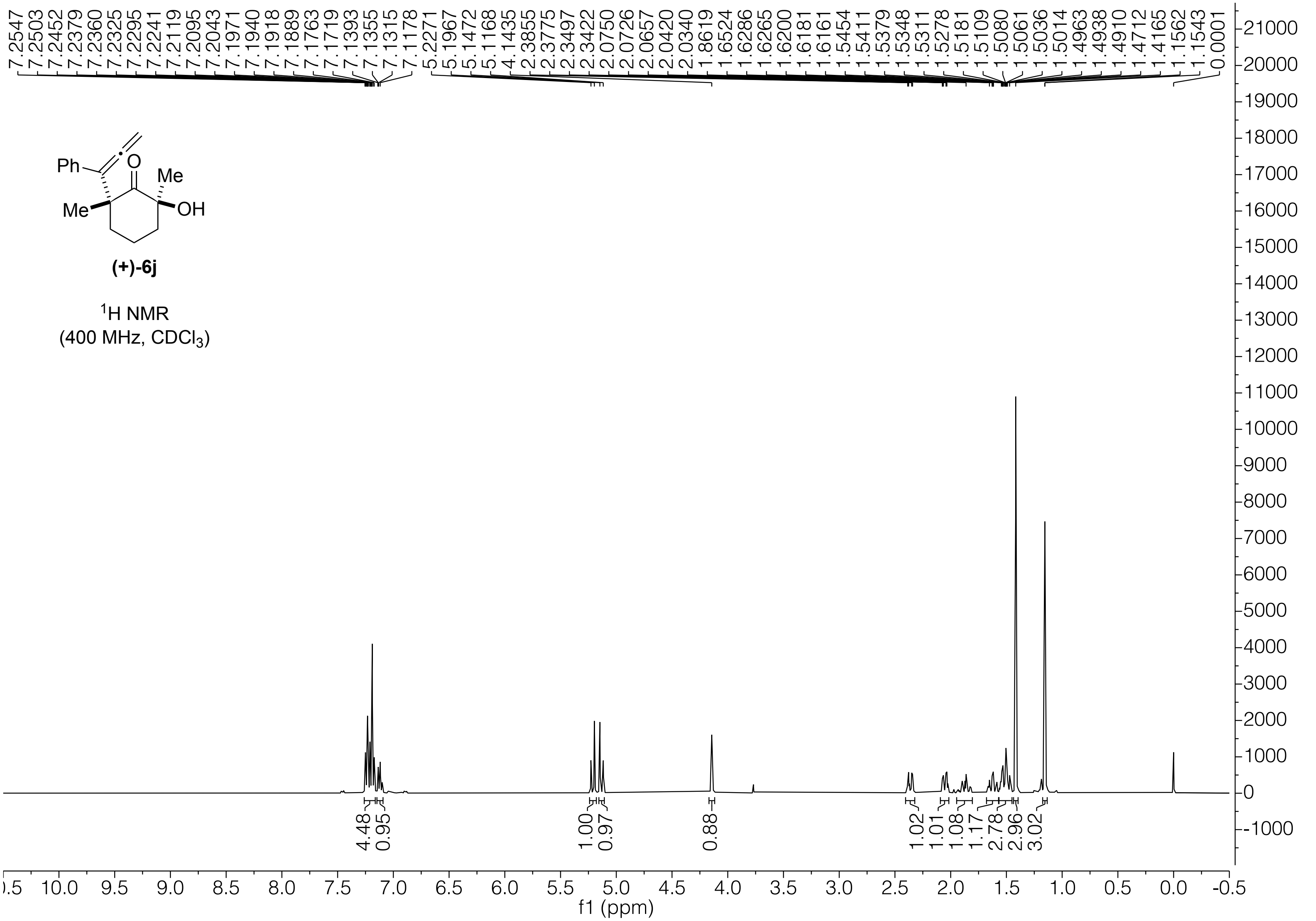

7.2547
7.2503
7.2452
7.2379
7.2360
7.2325
7.2295
7.2241
7.2119
7.2095
7.2043
7.1971
7.1940
7.1918
7.1889
7.1763
7.1719
7.1393
7.1355
7.1315
7.1178
5.2271
5.1967
5.1472
5.1168
4.1435
2.3855
2.3775
2.3497
2.3422
2.0750
2.0726
2.0657
2.0420
2.0340
1.8619
1.6524
1.6286
1.6265
1.6200
1.6181
1.6161
1.5454
1.5411
1.5379
1.5348
1.5311
1.5278
1.5181
1.5109
1.5080
1.5061
1.5036
1.5014
1.4963
1.4938
1.4910
1.4712
1.4165
1.1562
1.1543
0.0001
Ph
O
Me
Me
OH
(+)-6j
1H NMR
(400 MHz, CDCl3)
4.48
0.95
1.00
0.97
0.88
1.02
1.01
1.08
1.17
2.78
2.96
3.02
f1 (ppm)

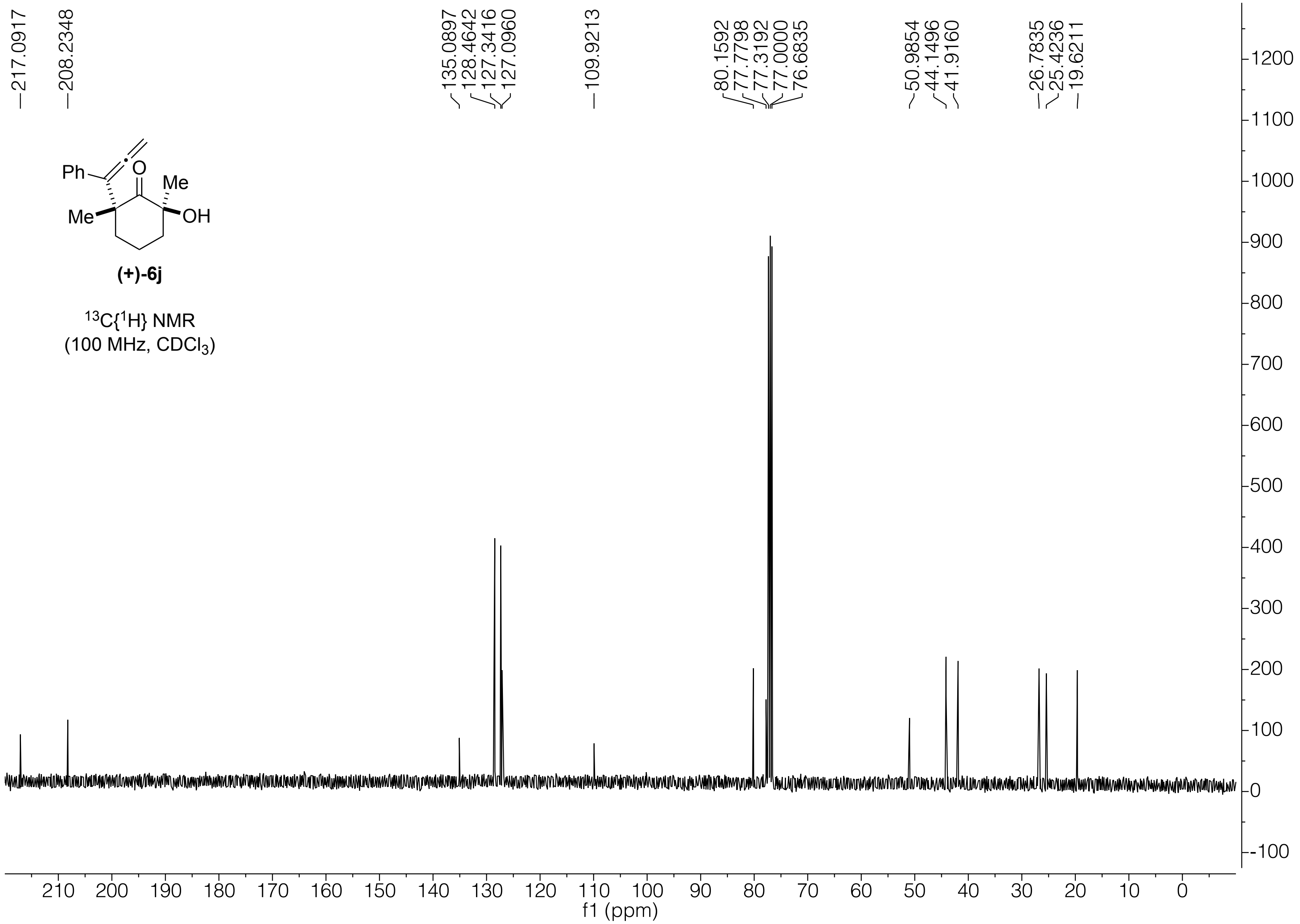
217.0917
208.2348
135.0897
128.4642
127.3416
127.0960
109.9213
80.1592
77.7798
77.3192
77.0000
76.6835
50.9854
44.1496
41.9160
26.7835
25.4236
19.6211
Ph
O
Me
Me
OH
(+)-6j
$^{13}C\{^{1}H\}$ NMR
(100 MHz, $CDCl_3$)
f1 (ppm)

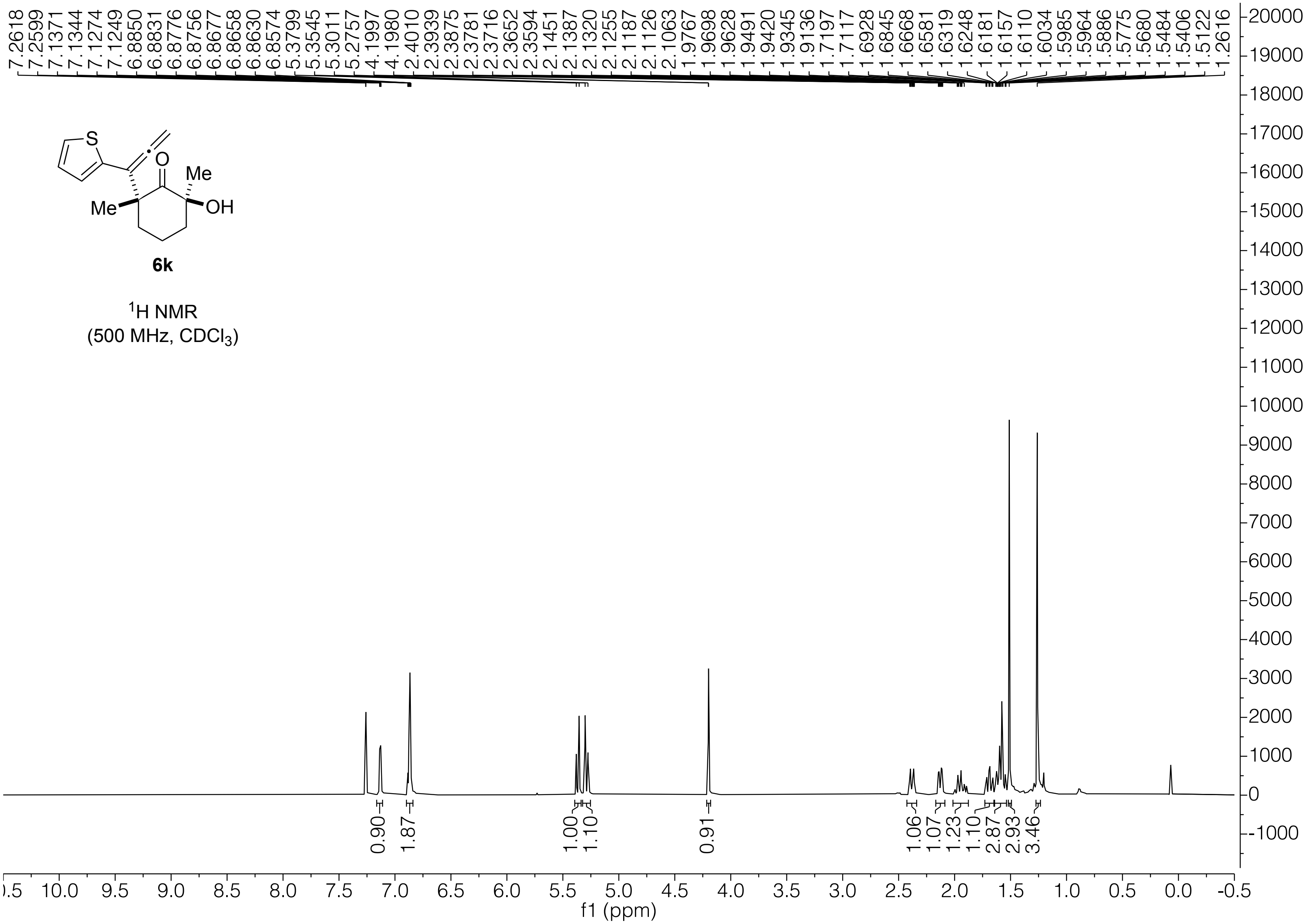
7.2618 7.2599 7.1371 7.1344 7.1274 7.1249 6.8850 6.8831 6.8776 6.8756 6.8677 6.8658 6.8630 6.8574 5.3799 5.3545 5.3011 5.2757 4.1997 4.1980 2.4010 2.3939 2.3875 2.3781 2.3716 2.3652 2.3594 2.1451 2.1387 2.1320 2.1255 2.1187 2.1126 2.1063 1.9767 1.9698 1.9628 1.9491 1.9420 1.9345 1.9136 1.7197 1.7117 1.6928 1.6845 1.6668 1.6581 1.6319 1.6248 1.6181 1.6157 1.6110 1.6034 1.5985 1.5964 1.5886 1.5775 1.5680 1.5484 1.5406 1.5122 1.2616
S
O
Me
OH
Me
6k
^{1}H NMR
(500 MHz, $CDCl_3$)
0.90
1.87
1.00
1.10
0.91
1.06
1.07
1.23
1.10
2.87
2.93
3.46
f1 (ppm)

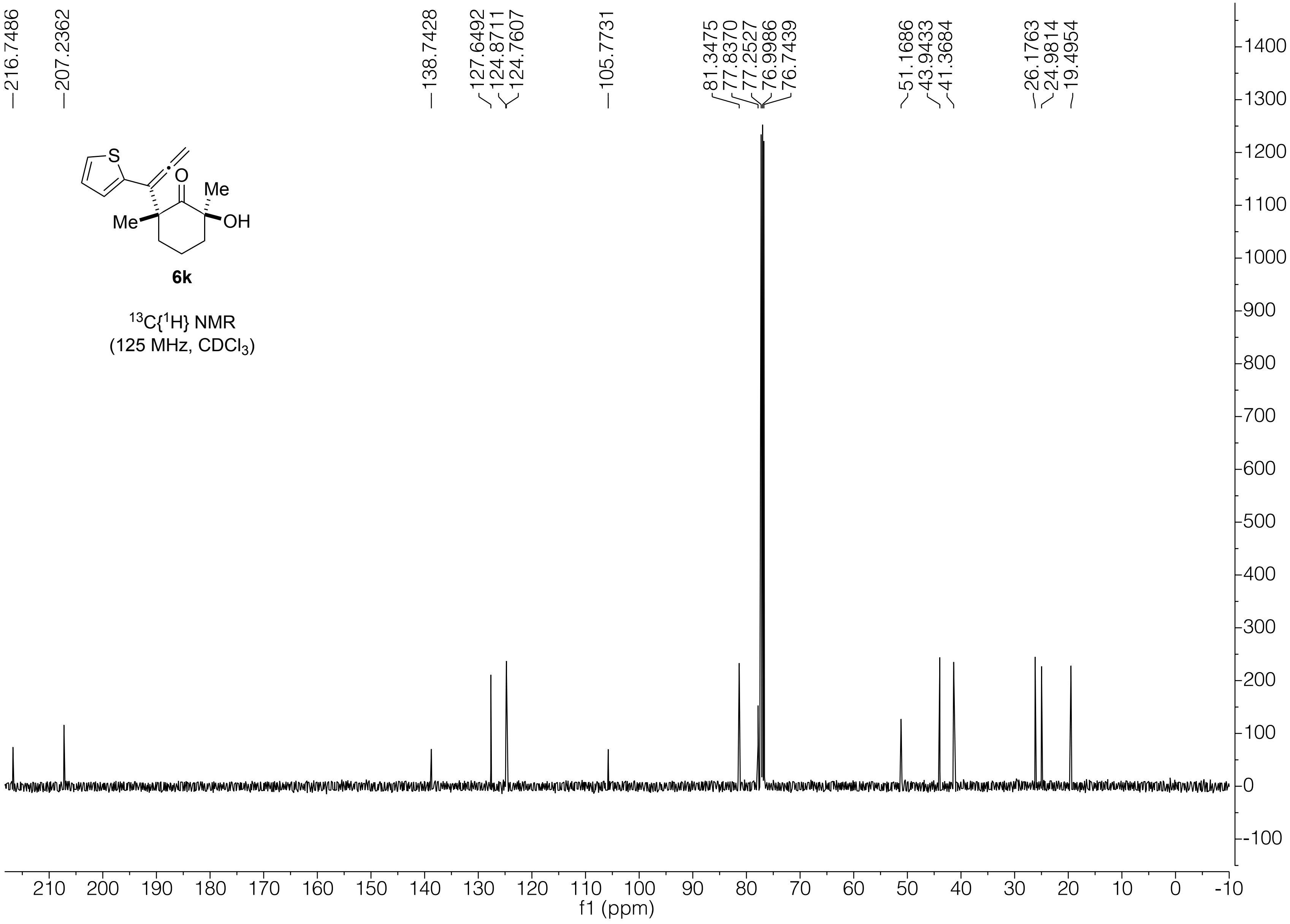

216.7486
207.2362
138.7428
127.6492
124.8711
124.7607
105.7731
81.3475
77.8370
77.2527
76.9986
76.7439
51.1686
43.9433
41.3684
26.1763
24.9814
19.4954
S
O
Me
Me
OH
6k
$^{13}C\{^1H\}$ NMR
(125 MHz, $CDCl_3$)
f1 (ppm)

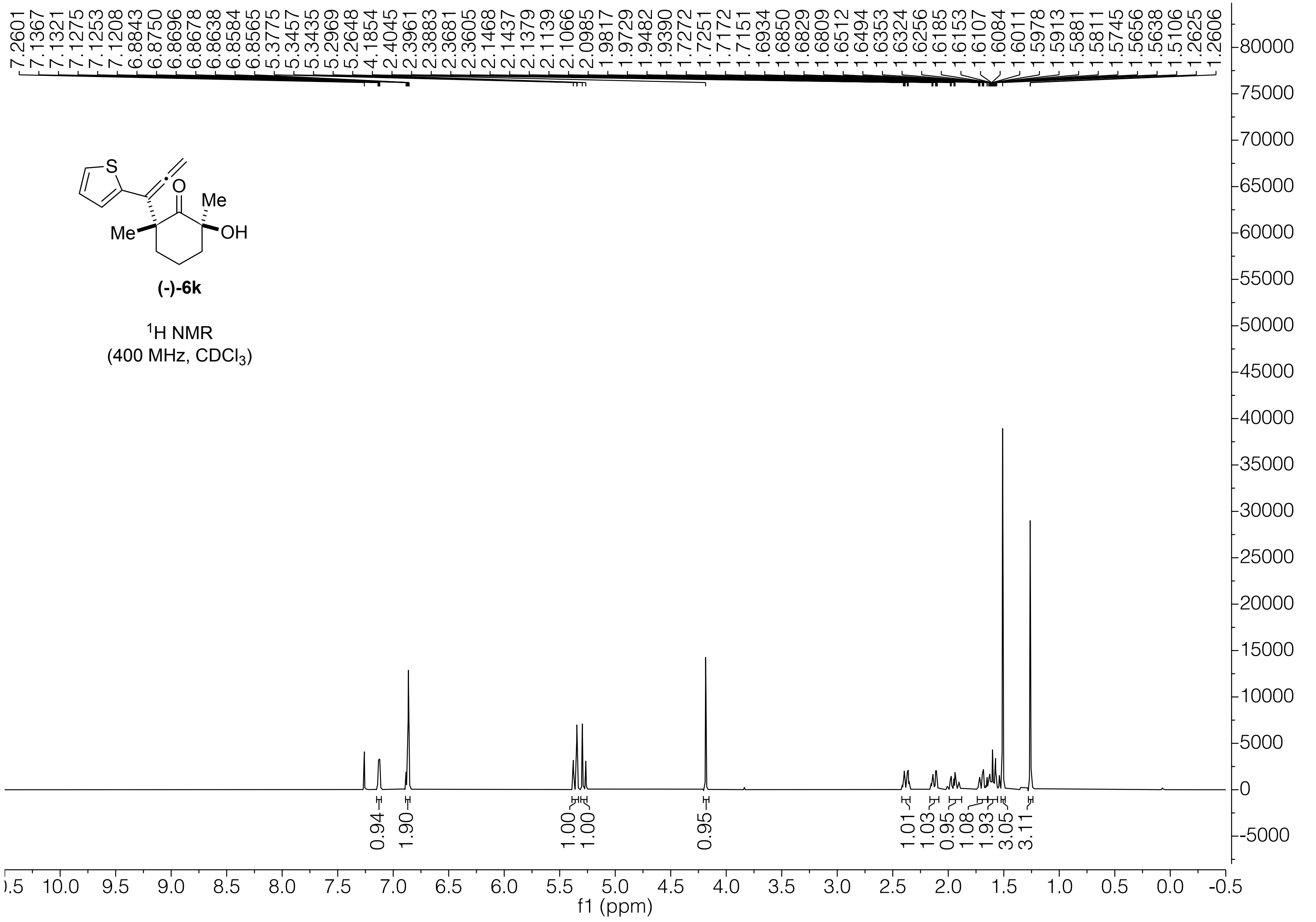
(-)-6k
^{1}H NMR
(400 MHz, $CDCl_3$)
f1 (ppm)

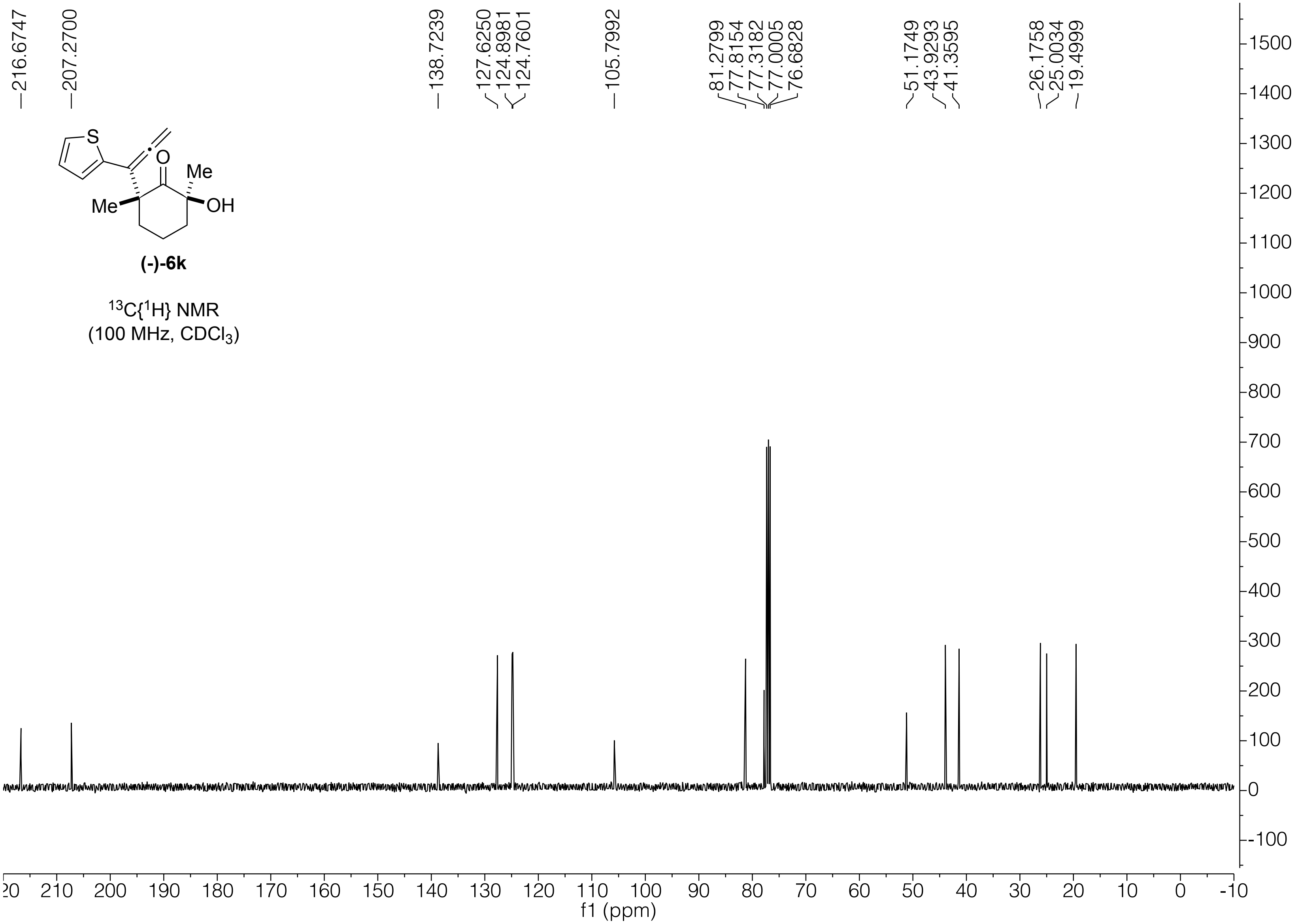
216.6747
207.2700
138.7239
127.6250
124.8981
124.7601
105.7992
81.2799
77.8154
77.3182
77.0005
76.6828
51.1749
43.9293
41.3595
26.1758
25.0034
19.4999
S
O
Me
OH
Me
(-)-6k
$^{13}C\{^1H\}$ NMR
(100 MHz, $CDCl_3$)
f1 (ppm)

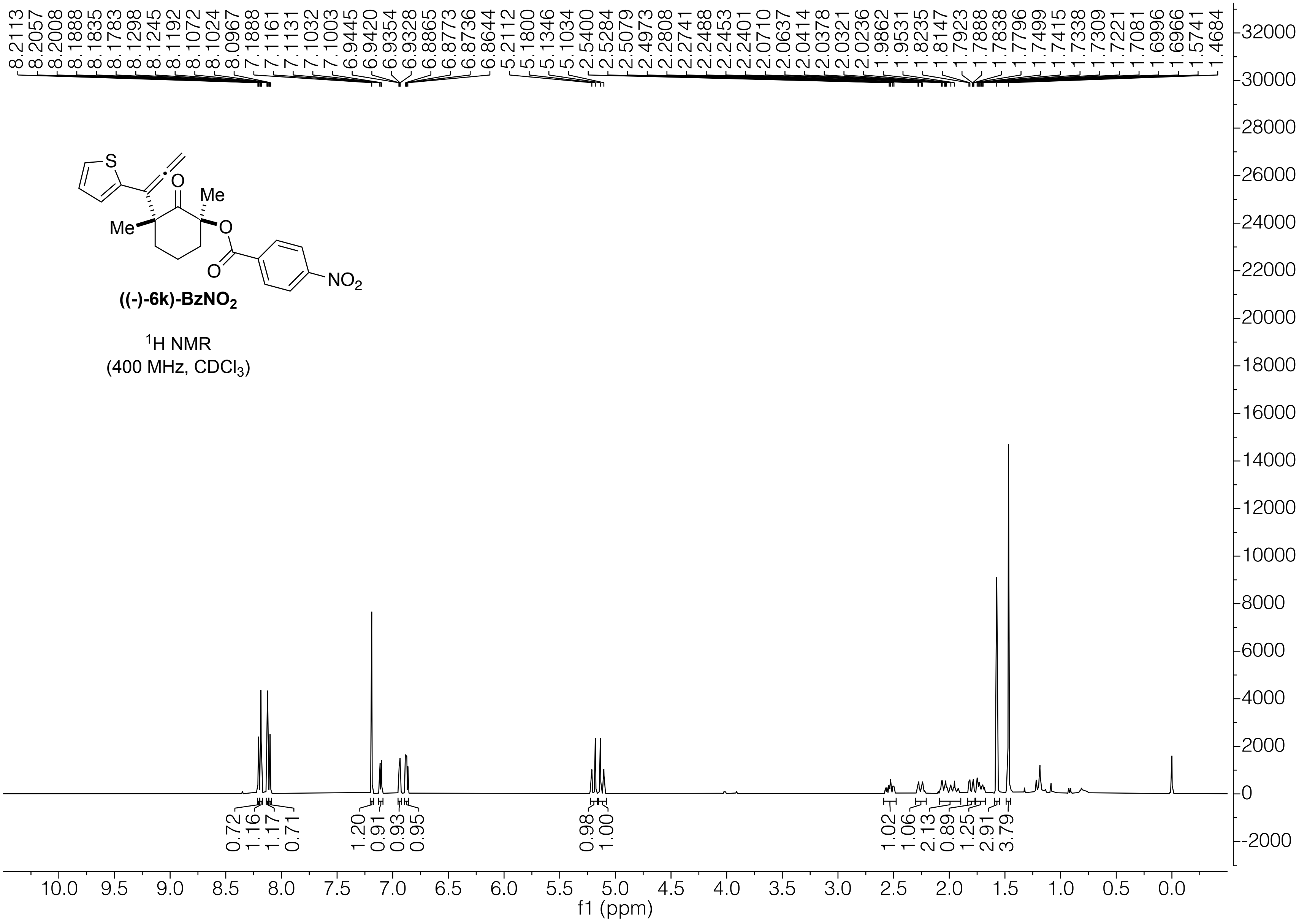
8.2113 8.2057 8.2008 8.1888 8.1835 8.1783 8.1298 8.1245 8.1192 8.1072 8.1024 8.0967 7.1888 7.1161 7.1131 7.1032 7.1003 6.9445 6.9420 6.9354 6.9328 6.8865 6.8773 6.8736 6.8644 5.2112 5.1800 5.1346 5.1034 2.5400 2.5284 2.5079 2.4973 2.2808 2.2741 2.2488 2.2453 2.2401 2.0710 2.0637 2.0414 2.0378 2.0321 2.0236 1.9862 1.9531 1.8235 1.8147 1.7923 1.7888 1.7838 1.7796 1.7499 1.7415 1.7338 1.7309 1.7221 1.7081 1.6996 1.6966 1.5741 1.4684
S
O
Me
Me
O
O
NO2
((-)-6k)-BzNO2
1H NMR
(400 MHz, CDCl3)
0.72 1.16 1.17 0.71 1.20 0.91 0.93 0.95 0.98 1.00 1.02 1.06 2.13 0.89 1.25 2.91 3.79
f1 (ppm)

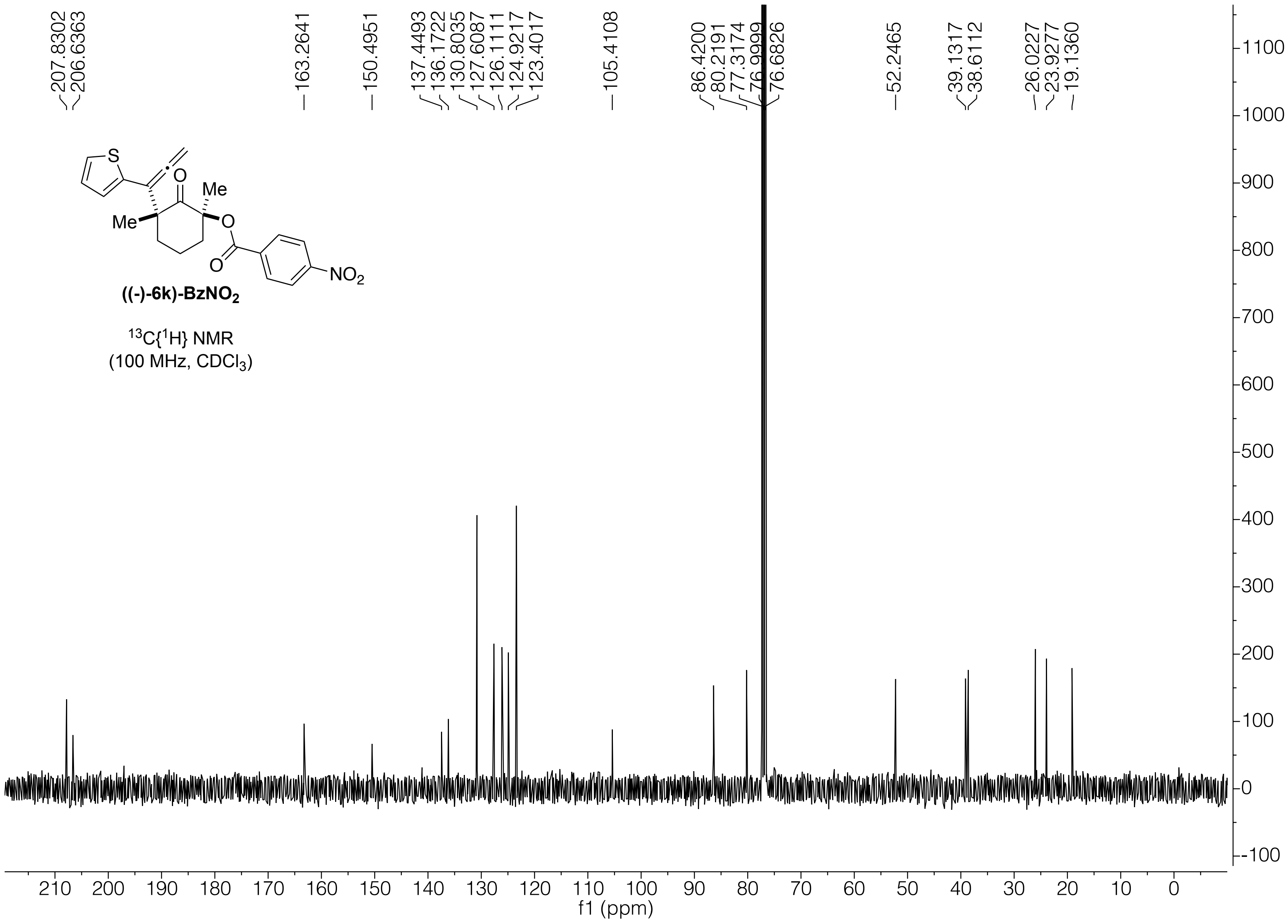

207.8302
206.6363
163.2641
150.4951
137.4493
136.1722
130.8035
127.6087
126.1111
124.9217
123.4017
105.4108
86.4200
80.2191
77.3174
76.9999
76.6826
52.2465
39.1317
38.6112
26.0227
23.9277
19.1360
S
O
Me
Me
O
O
NO_2
((-)-6k)-BzNO$_2$
$^{13}C\{^1H\}$ NMR
(100 MHz, $CDCl_3$)
f1 (ppm)

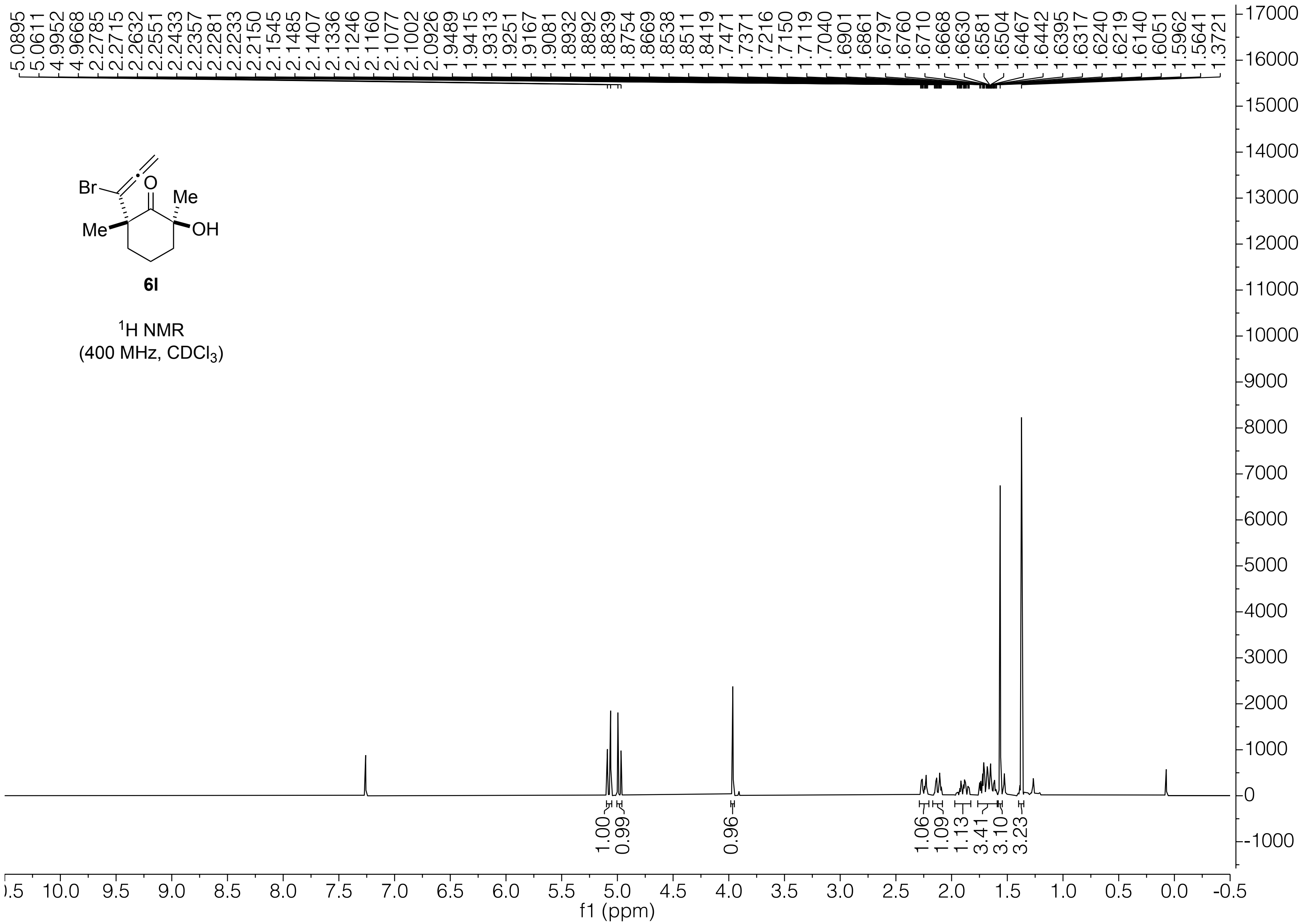

5.0895
5.0611
4.9952
4.9668
2.2785
2.2715
2.2632
2.2551
2.2433
2.2357
2.2281
2.2233
2.2150
2.1545
2.1485
2.1407
2.1336
2.1246
2.1160
2.1077
2.1002
2.0926
1.9489
1.9415
1.9313
1.9251
1.9167
1.9081
1.8932
1.8892
1.8839
1.8754
1.8669
1.8538
1.8511
1.8419
1.7471
1.7371
1.7216
1.7150
1.7119
1.7040
1.6901
1.6861
1.6797
1.6760
1.6710
1.6668
1.6630
1.6581
1.6504
1.6467
1.6442
1.6395
1.6317
1.6240
1.6219
1.6140
1.6051
1.5962
1.5641
1.3721
Br
O
Me
Me
OH
6l
1H NMR
(400 MHz, CDCl3)
1.00
0.99
0.96
1.06
1.09
1.13
3.41
3.10
3.23
f1 (ppm)

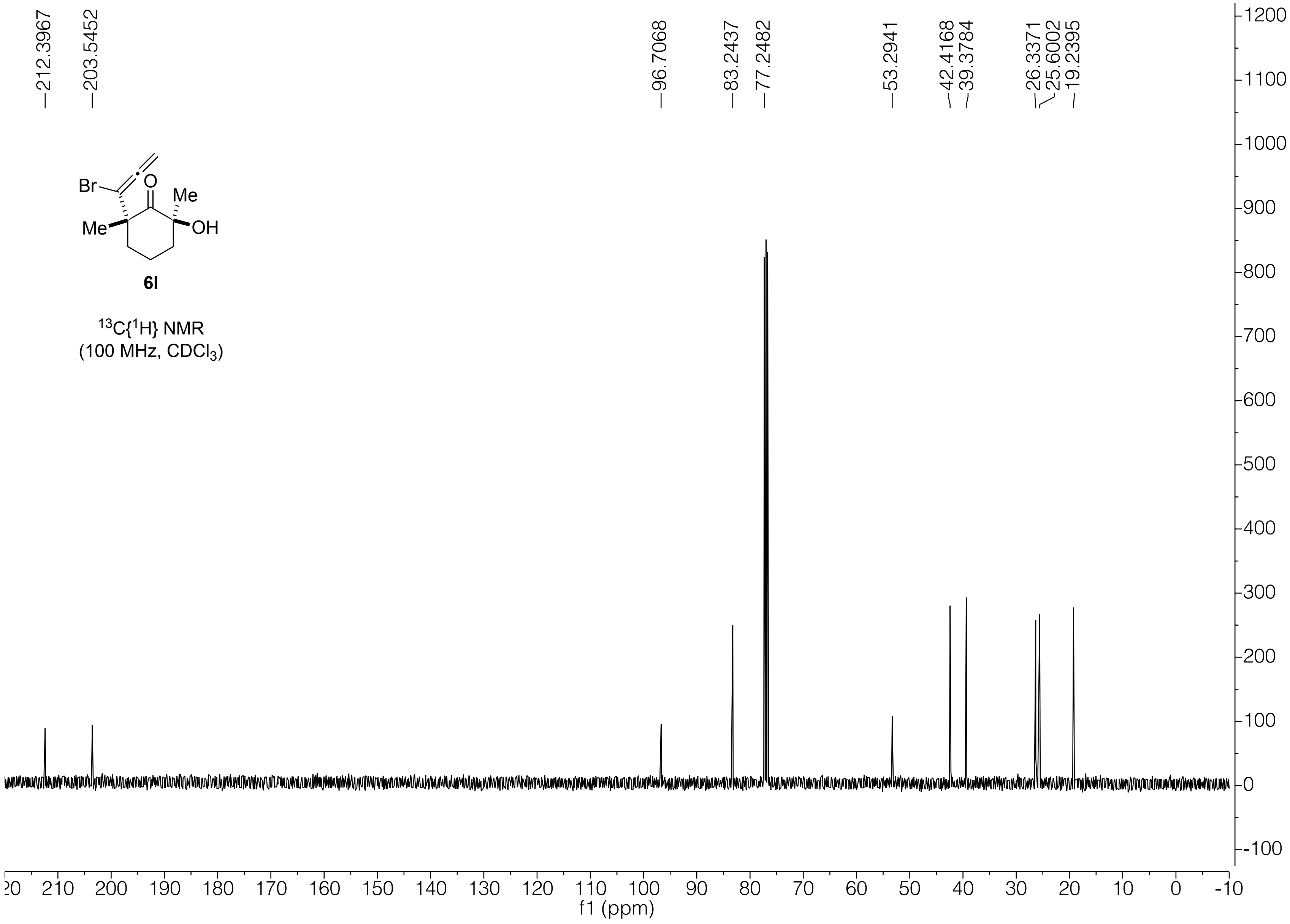
212.3967
203.5452
96.7068
83.2437
77.2482
53.2941
42.4168
39.3784
26.3371
25.6002
19.2395
Br
O
Me
Me
OH
6l
$^{13}C\{^1H\}$ NMR
(100 MHz, $CDCl_3$)
f1 (ppm)

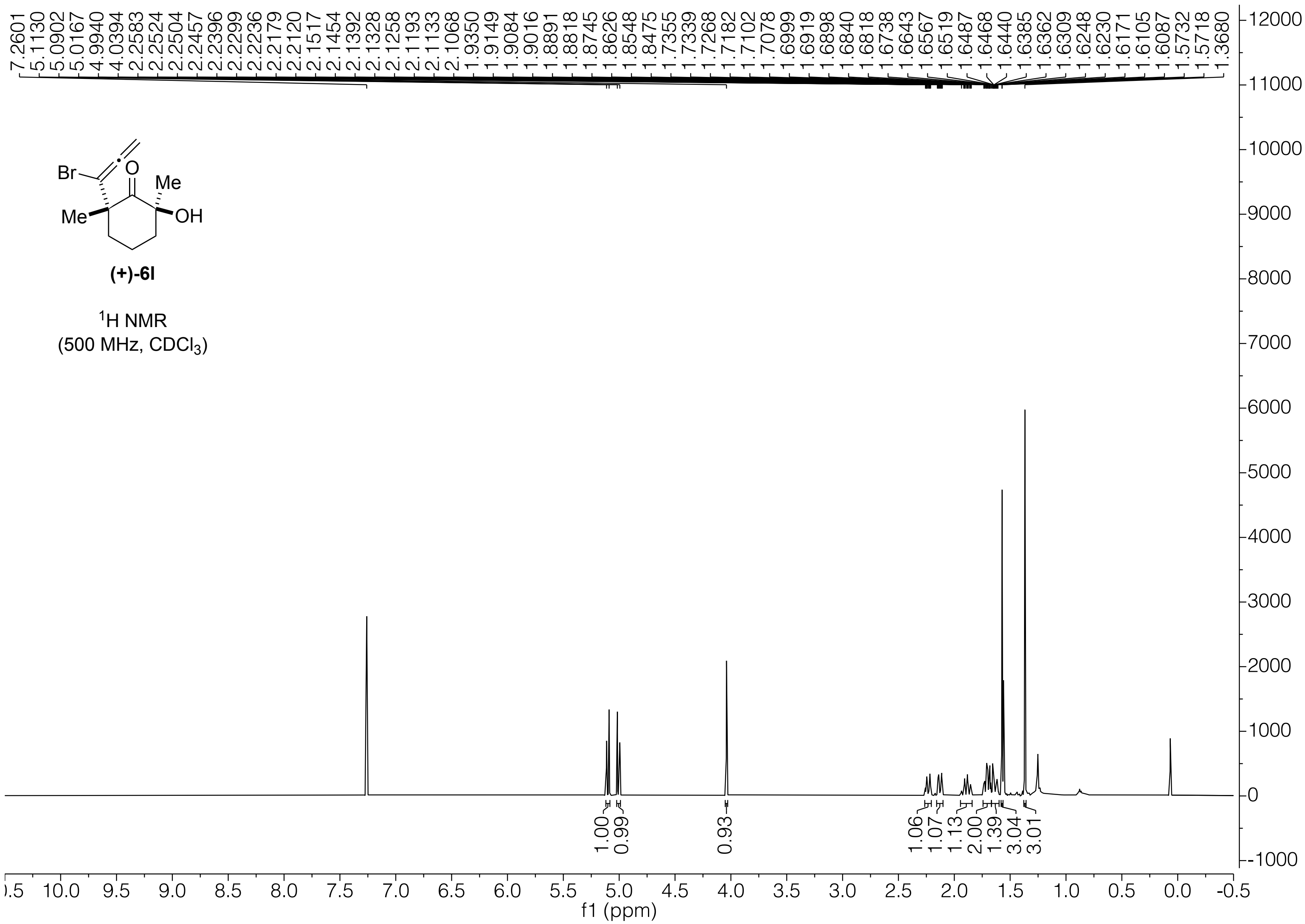
Br
O
Me
Me
OH
(+)-6l
^{1}H NMR
(500 MHz, CDCl$_3$)
7.2601
5.1130
5.0902
5.0167
4.9940
4.0394
2.2583
2.2524
2.2504
2.2457
2.2396
2.2299
2.2236
2.2179
2.2120
2.1517
2.1454
2.1392
2.1328
2.1258
2.1193
2.1133
2.1068
1.9350
1.9149
1.9084
1.9016
1.8891
1.8818
1.8745
1.8626
1.8548
1.8475
1.7355
1.7339
1.7268
1.7182
1.7102
1.7078
1.6999
1.6919
1.6898
1.6840
1.6818
1.6738
1.6643
1.6567
1.6519
1.6487
1.6468
1.6440
1.6385
1.6362
1.6309
1.6248
1.6230
1.6171
1.6105
1.6087
1.5732
1.5718
1.3680
1.00
0.99
0.93
1.06
1.07
1.13
2.00
1.39
3.04
3.01
f1 (ppm)

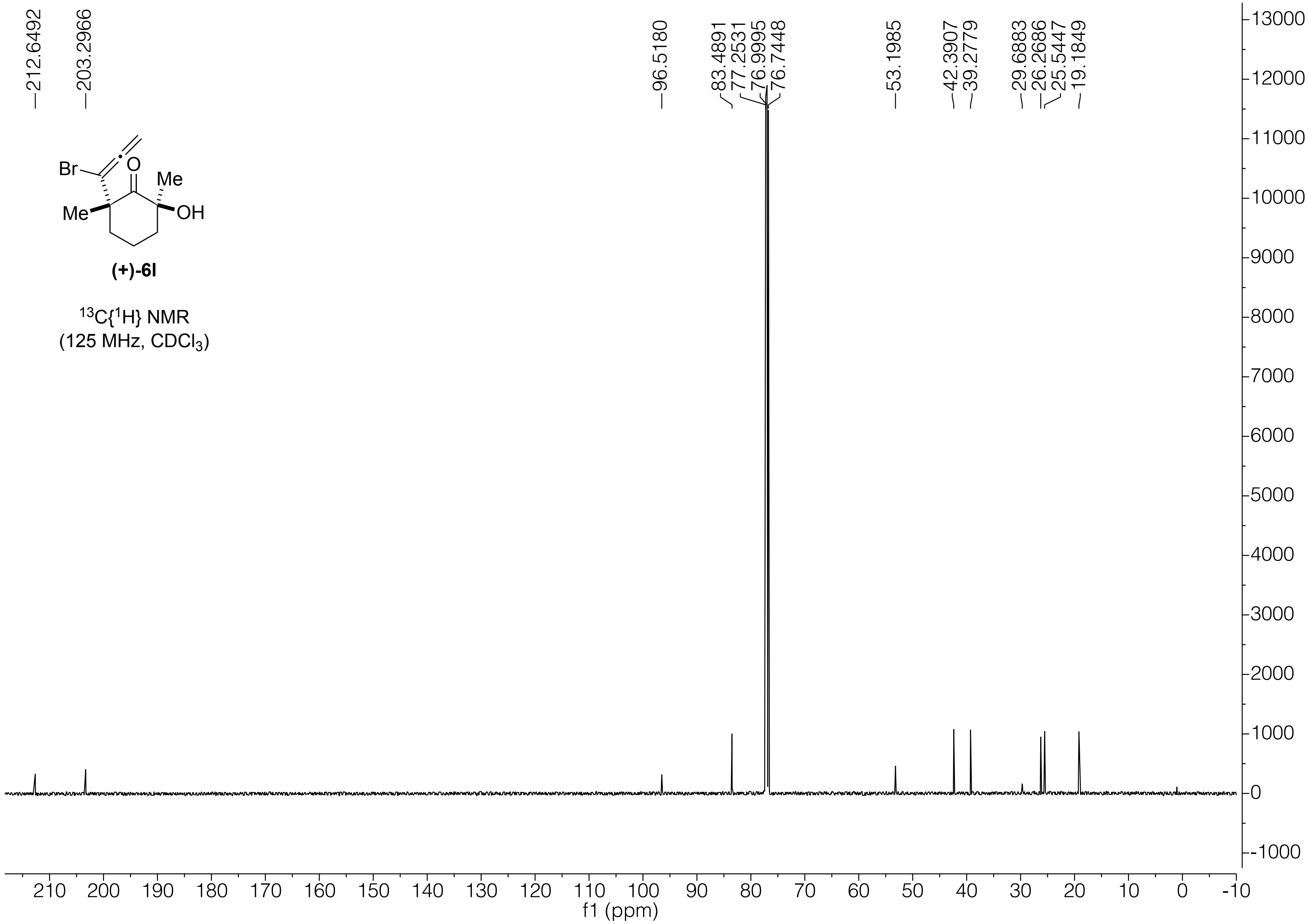
212.6492
203.2966
96.5180
83.4891
77.2531
76.9995
76.7448
53.1985
42.3907
39.2779
29.6883
26.2686
25.5447
19.1849
Br
O
Me
Me
OH
(+)-6l
$^{13}C\{^1H\}$ NMR
(125 MHz, $CDCl_3$)
f1 (ppm)

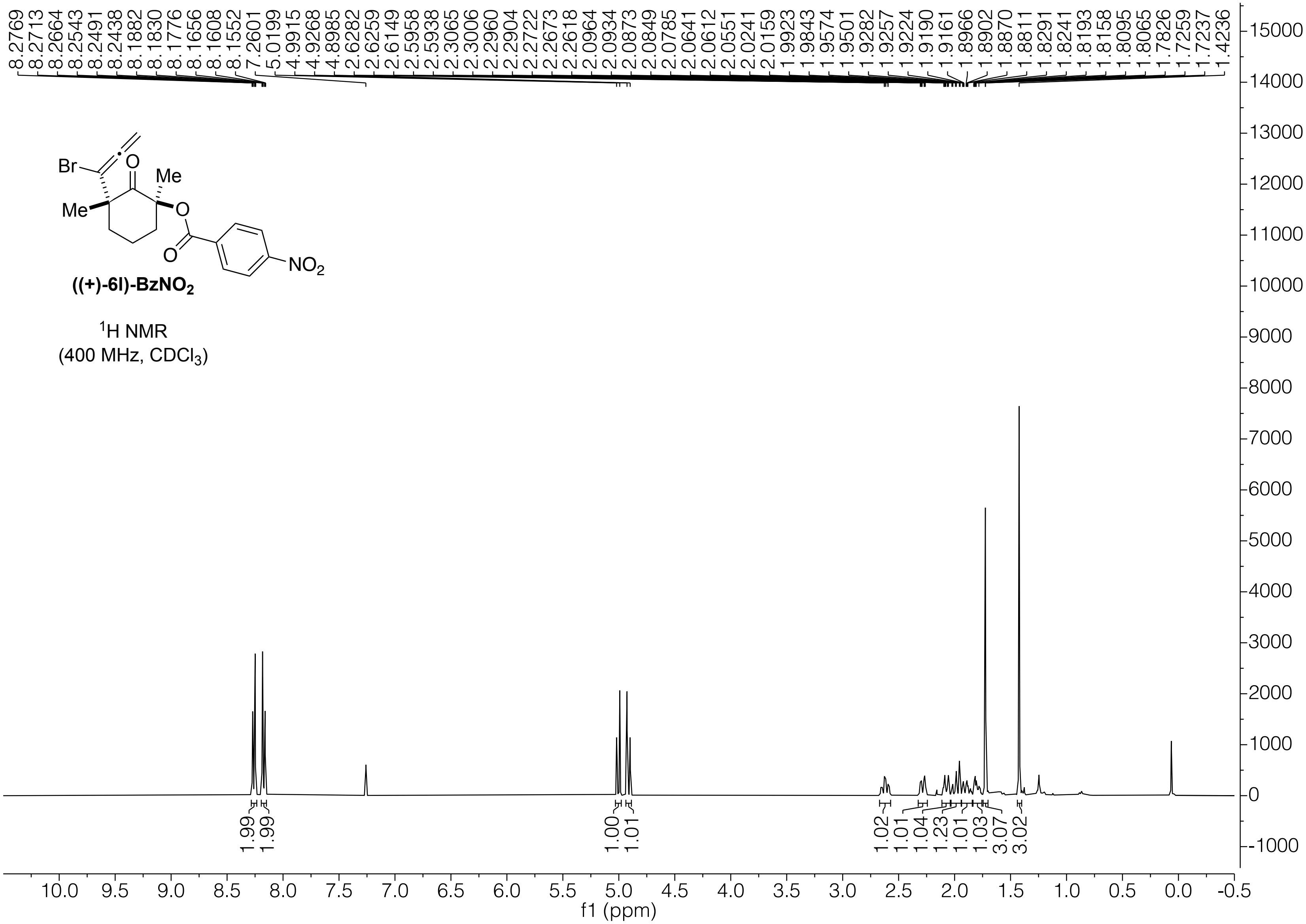

Br
O
Me
Me
O
O
NO_2
((+)-6l)-BzNO2
1H NMR
(400 MHz, CDCl3)
8.2769 8.2713 8.2664 8.2543 8.2491 8.2438 8.1882 8.1830 8.1776 8.1656 8.1608 8.1552 7.2601 5.0199 4.9915 4.9268 4.8985 2.6282 2.6259 2.6149 2.5958 2.5938 2.3065 2.3006 2.2960 2.2904 2.2722 2.2673 2.2618 2.0964 2.0934 2.0873 2.0849 2.0785 2.0641 2.0612 2.0551 2.0241 2.0159 1.9923 1.9843 1.9574 1.9501 1.9282 1.9257 1.9224 1.9190 1.9161 1.8966 1.8902 1.8870 1.8811 1.8291 1.8241 1.8193 1.8158 1.8095 1.8065 1.7826 1.7259 1.7237 1.4236
1.99 1.99 1.00 1.01 1.02 1.01 1.04 1.23 1.01 1.03 3.07 3.02
f1 (ppm)

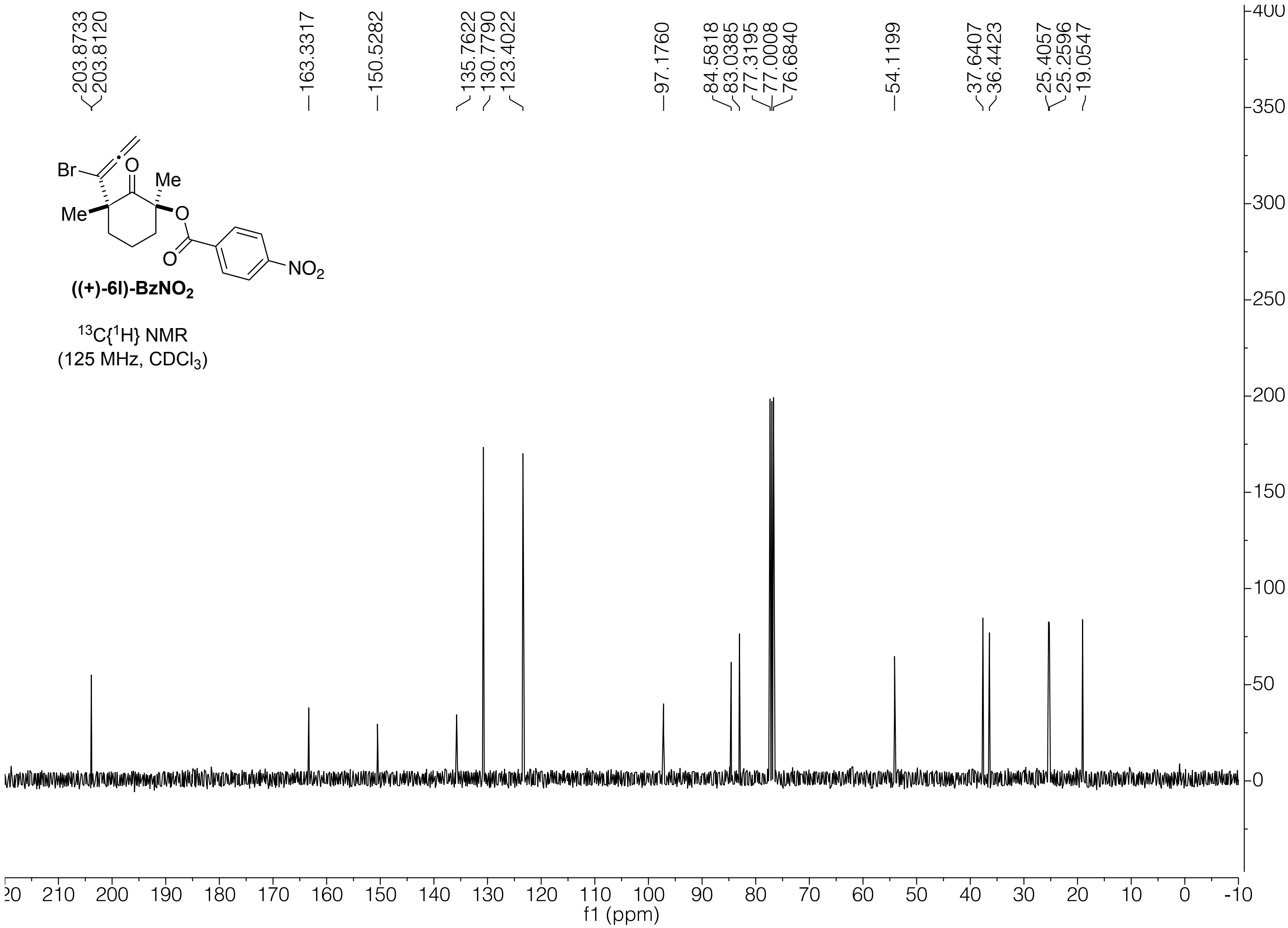

203.8733
203.8120
163.3317
150.5282
135.7622
130.7790
123.4022
97.1760
84.5818
83.0385
77.3195
77.0008
76.6840
54.1199
37.6407
36.4423
25.4057
25.2596
19.0547
Br
O
Me
Me
O
O
NO_2
((+)-6l)-$BzNO_2$
$^{13}C\{^1H\}$ NMR
(125 MHz, $CDCl_3$)
f1 (ppm)

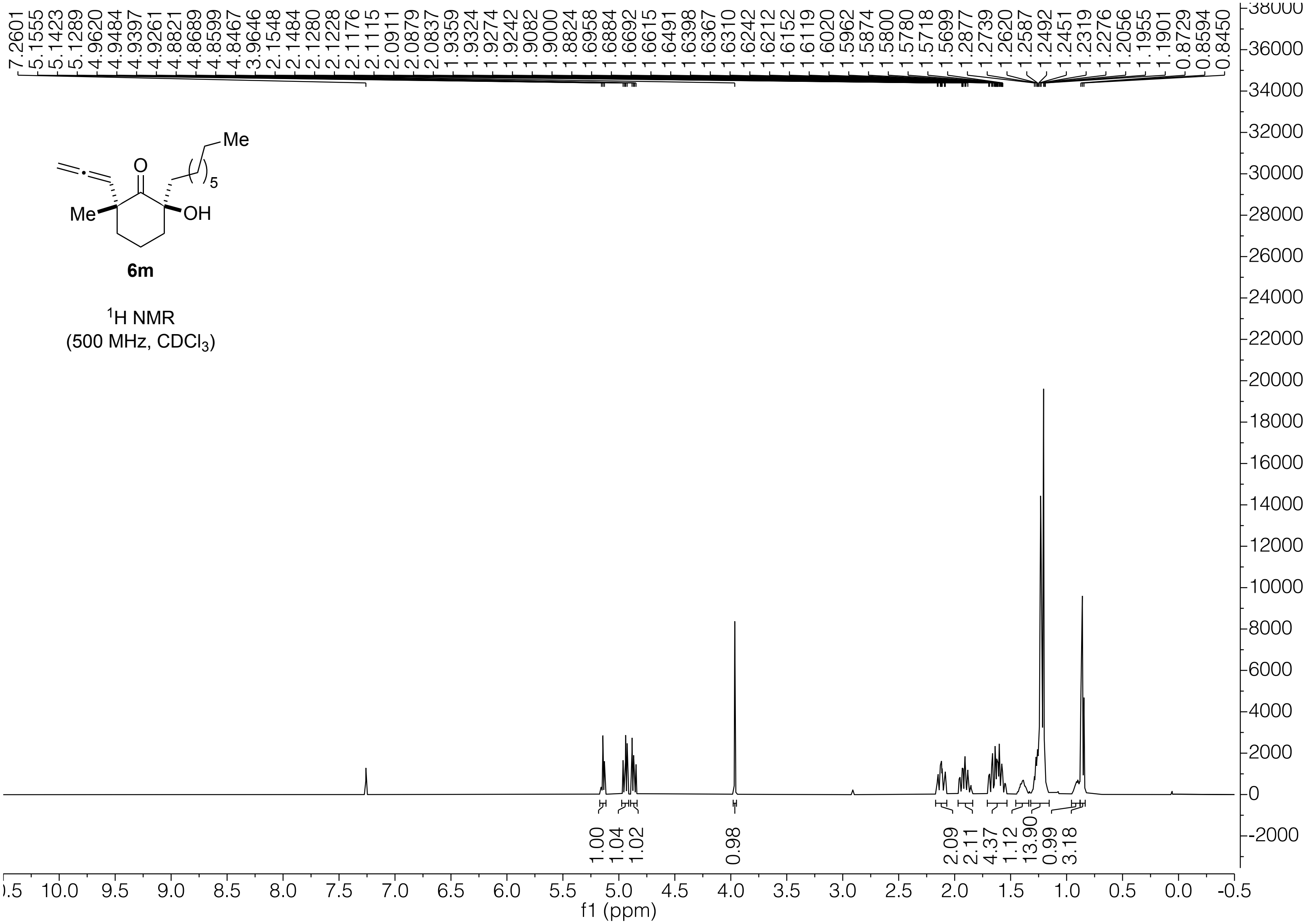

7.2601
5.1555
5.1423
5.1289
4.9620
4.9484
4.9397
4.9261
4.8821
4.8689
4.8599
4.8467
3.9646
2.1548
2.1484
2.1280
2.1228
2.1176
2.1115
2.0911
2.0879
2.0837
1.9359
1.9324
1.9274
1.9242
1.9082
1.9000
1.8824
1.6958
1.6884
1.6692
1.6615
1.6491
1.6398
1.6367
1.6310
1.6242
1.6212
1.6152
1.6119
1.6020
1.5962
1.5874
1.5800
1.5780
1.5718
1.5699
1.2877
1.2739
1.2620
1.2587
1.2492
1.2451
1.2319
1.2276
1.2056
1.1955
1.1901
0.8729
0.8594
0.8450
Me
O
5
Me
OH
6m
^{1}H NMR
(500 MHz, CDCl$_3$)
1.00
1.04
1.02
0.98
2.09
2.11
4.37
1.12
13.90
0.99
3.18
f1 (ppm)

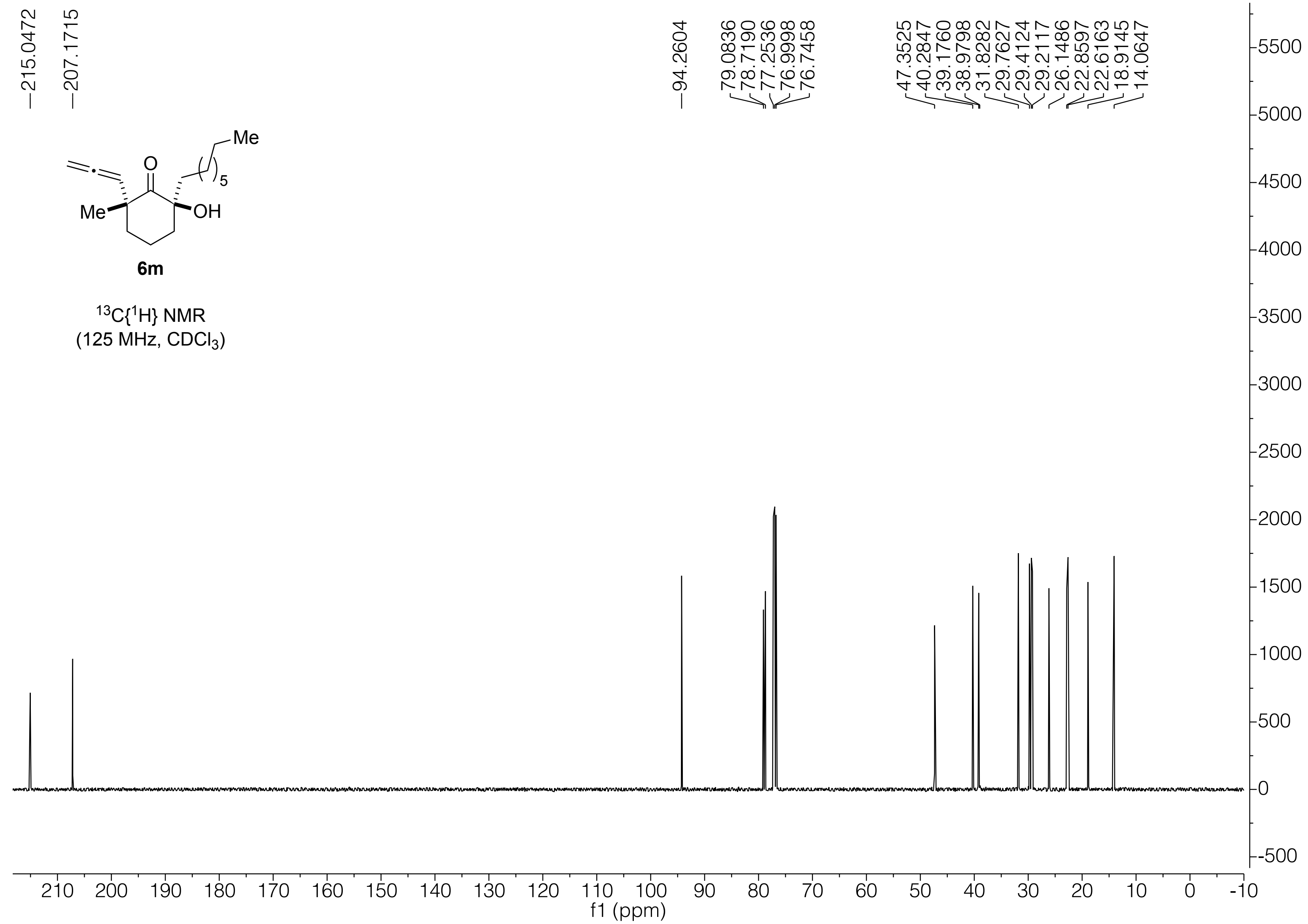

215.0472
207.1715
94.2604
79.0836
78.7190
77.2536
76.9998
76.7458
47.3525
40.2847
39.1760
38.9798
31.8282
29.7627
29.4124
29.2117
26.1486
22.8597
22.6163
18.9145
14.0647
Me
O
( )5
Me
OH
6m
$^{13}C\{^1H\}$ NMR
(125 MHz, $CDCl_3$)
f1 (ppm)

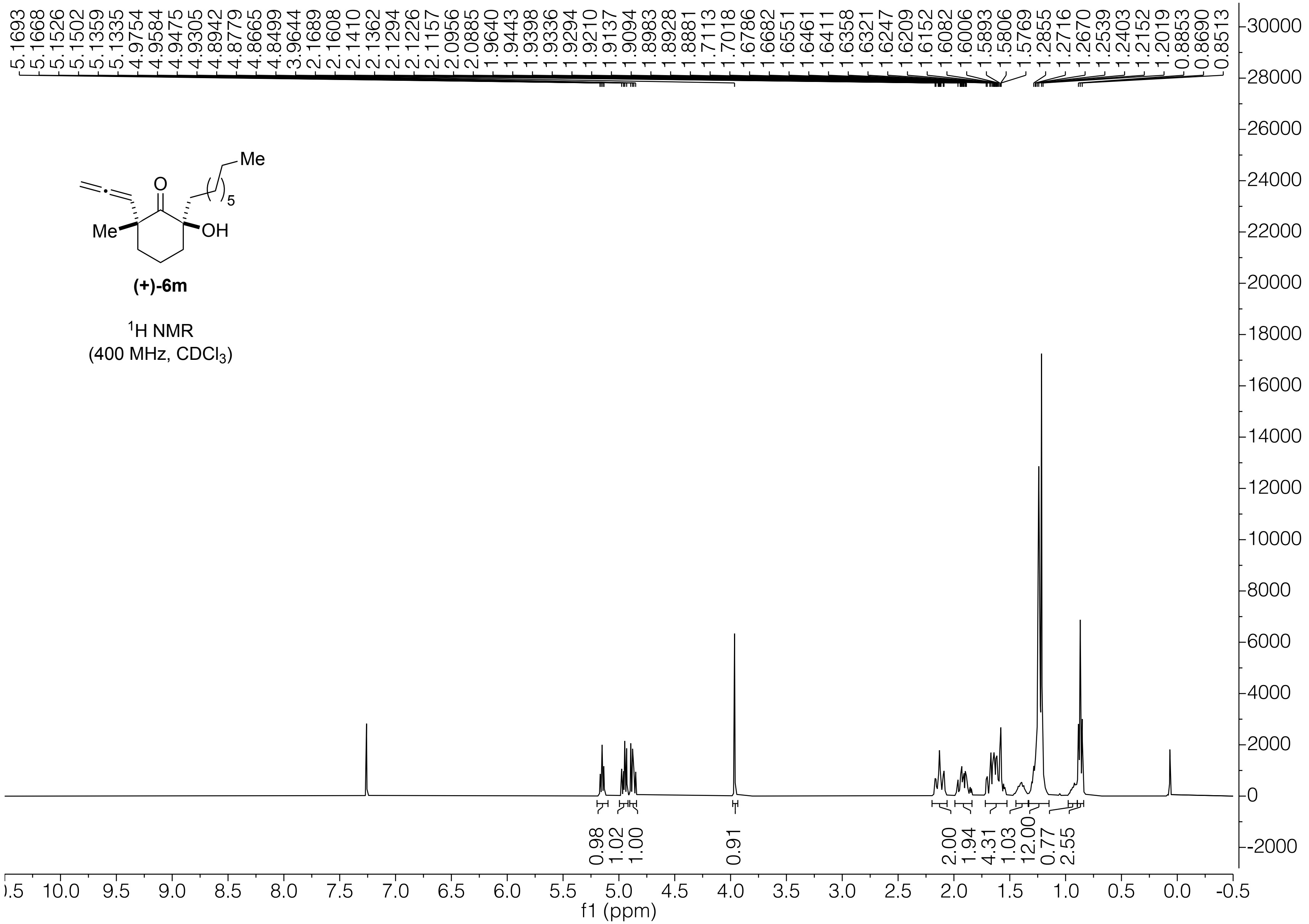

Me
O
Me
OH
5
(+)-6m
^{1}H NMR
(400 MHz, $CDCl_3$)
5.1693 5.1668 5.1526 5.1502 5.1359 5.1335 4.9754 4.9584 4.9475 4.9305 4.8942 4.8779 4.8665 4.8499 3.9644 2.1689 2.1608 2.1410 2.1362 2.1294 2.1226 2.1157 2.0956 2.0885 1.9640 1.9443 1.9398 1.9336 1.9294 1.9210 1.9137 1.9094 1.8983 1.8928 1.8881 1.7113 1.7018 1.6786 1.6682 1.6551 1.6461 1.6411 1.6358 1.6321 1.6247 1.6209 1.6152 1.6082 1.6006 1.5893 1.5806 1.5769 1.2855 1.2716 1.2670 1.2539 1.2403 1.2152 1.2019 0.8853 0.8690 0.8513
0.98 1.02 1.00 0.91 2.00 1.94 4.31 1.03 12.00 0.77 2.55
f1 (ppm)

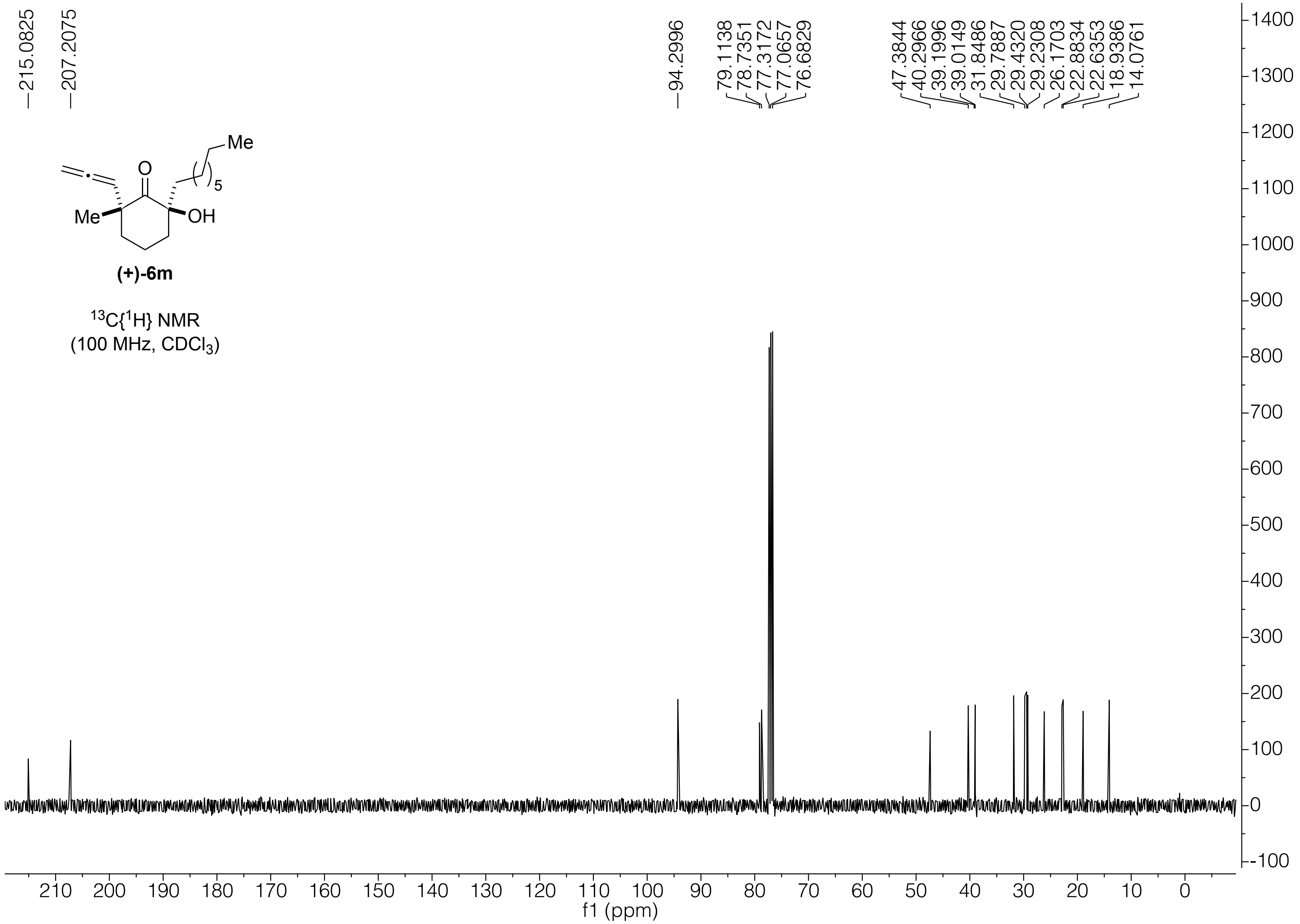

215.0825
207.2075
94.2996
79.1138
78.7351
77.3172
77.0657
76.6829
47.3844
40.2966
39.1996
39.0149
31.8486
29.7887
29.4320
29.2308
26.1703
22.8834
22.6353
18.9386
14.0761
Me
O
Me
OH
5
(+)-6m
$^{13}C\{^{1}H\}$ NMR
(100 MHz, $CDCl_3$)
f1 (ppm)

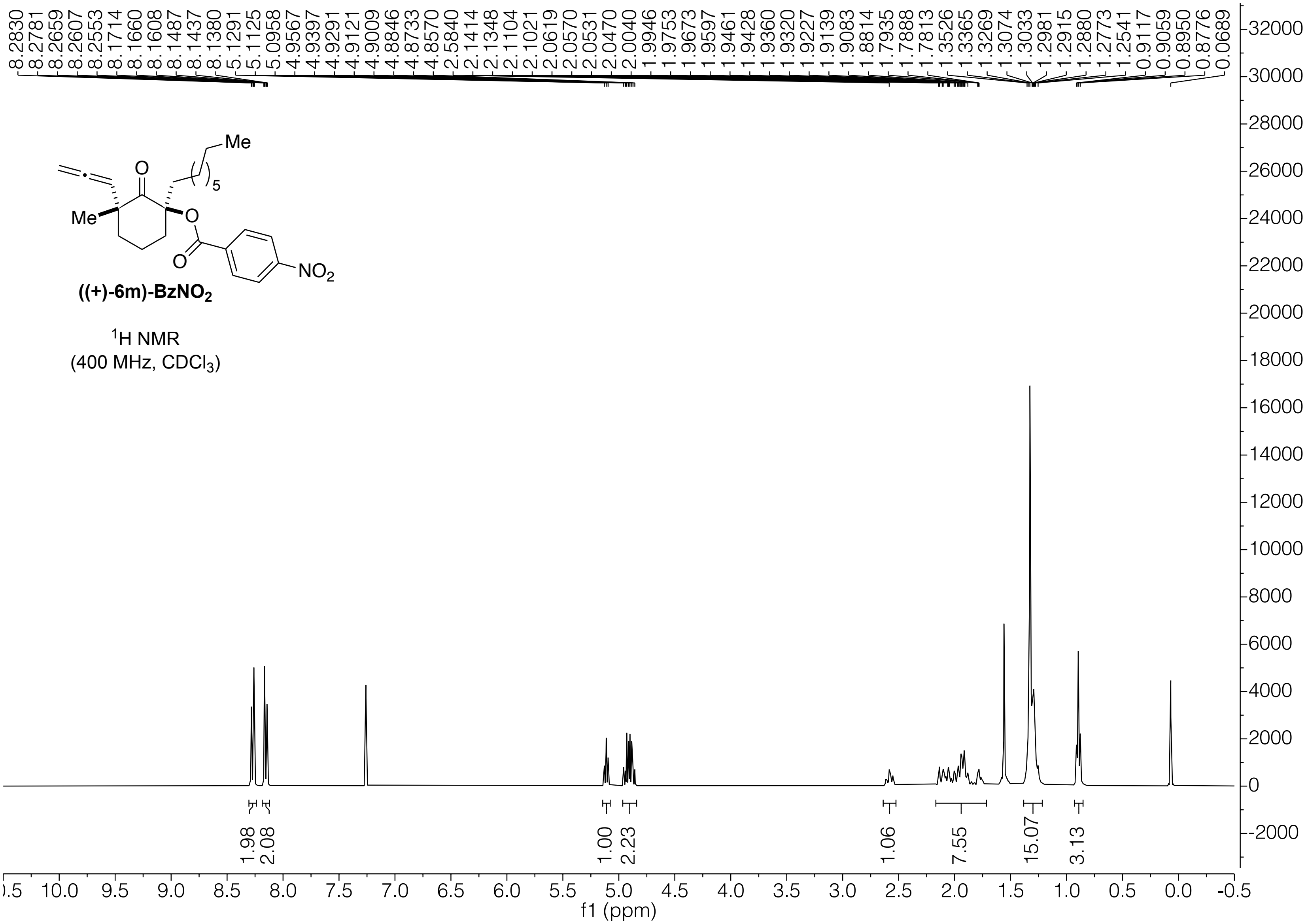
8.2830
8.2781
8.2659
8.2607
8.2553
8.1714
8.1660
8.1608
8.1487
8.1437
8.1380
5.1291
5.1125
5.0958
4.9567
4.9397
4.9291
4.9121
4.9009
4.8846
4.8733
4.8570
2.5840
2.1414
2.1348
2.1104
2.1021
2.0619
2.0570
2.0531
2.0470
2.0040
1.9946
1.9753
1.9673
1.9597
1.9461
1.9428
1.9360
1.9320
1.9227
1.9139
1.9083
1.8814
1.7935
1.7888
1.7813
1.3526
1.3365
1.3269
1.3074
1.3033
1.2981
1.2915
1.2880
1.2773
1.2541
0.9117
0.9059
0.8950
0.8776
0.0689
Me
O
Me
O
O
NO_2
5
((+)-6m)-$BzNO_2$
^1H NMR
(400 MHz, $CDCl_3$)
1.98
2.08
1.00
2.23
1.06
7.55
15.07
3.13
f1 (ppm)

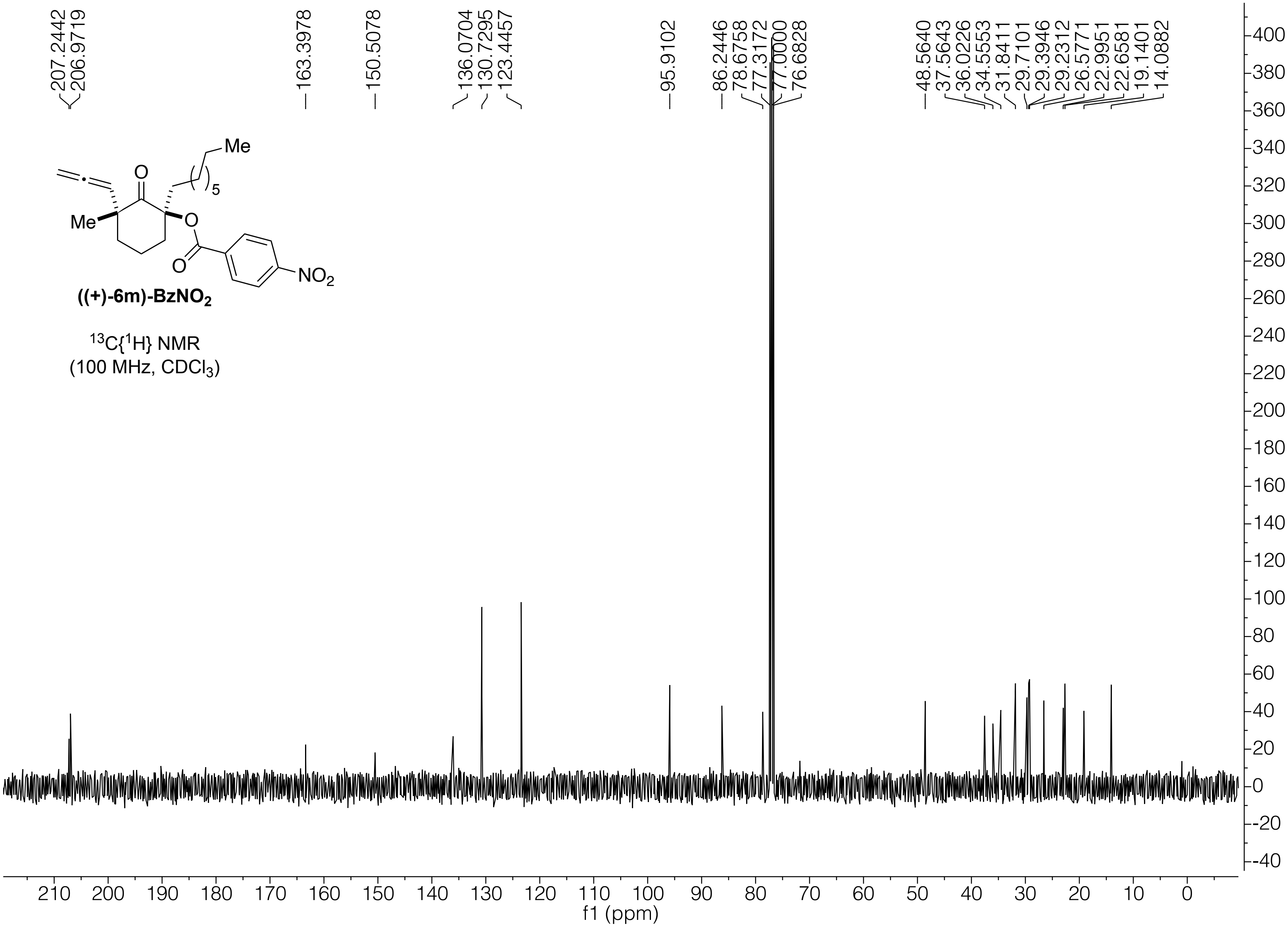

207.2442
206.9719
163.3978
150.5078
136.0704
130.7295
123.4457
95.9102
86.2446
78.6758
77.3172
77.0000
76.6828
48.5640
37.5643
36.0226
34.5553
31.8411
29.7101
29.3946
29.2312
26.5771
22.9951
22.6581
19.1401
14.0882
((+)-6m)-BzNO$_2$
$^{13}C\{^1H\}$ NMR
(100 MHz, $CDCl_3$)
f1 (ppm)

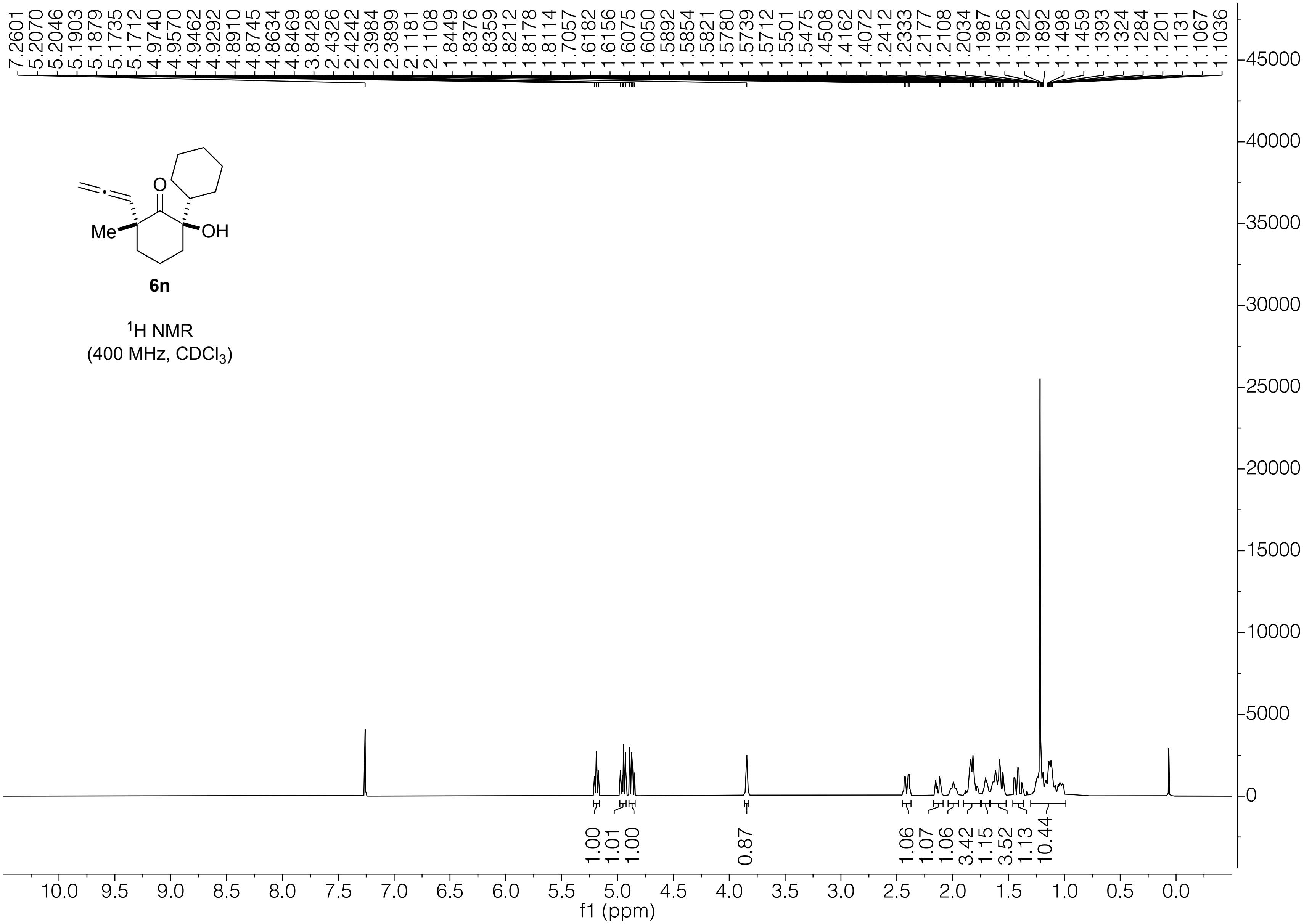
7.2601
5.2070
5.2046
5.1903
5.1879
5.1735
5.1712
4.9740
4.9570
4.9462
4.9292
4.8910
4.8745
4.8634
4.8469
3.8428
2.4326
2.4242
2.3984
2.3899
2.1181
2.1108
1.8449
1.8376
1.8359
1.8212
1.8178
1.8114
1.7057
1.6182
1.6156
1.6075
1.6050
1.5892
1.5854
1.5821
1.5780
1.5739
1.5712
1.5501
1.5475
1.4508
1.4162
1.4072
1.2412
1.2333
1.2177
1.2108
1.2034
1.1987
1.1956
1.1922
1.1892
1.1498
1.1459
1.1393
1.1324
1.1284
1.1201
1.1131
1.1067
1.1036
O
Me
OH
6n
^{1}H NMR
(400 MHz, $CDCl_3$)
1.00
1.01
1.00
0.87
1.06
1.07
1.06
3.42
1.15
3.52
1.13
10.44
45000
40000
35000
30000
25000
20000
15000
10000
5000
0
10.0 9.5 9.0 8.5 8.0 7.5 7.0 6.5 6.0 5.5 5.0 4.5 4.0 3.5 3.0 2.5 2.0 1.5 1.0 0.5 0.0
f1 (ppm)

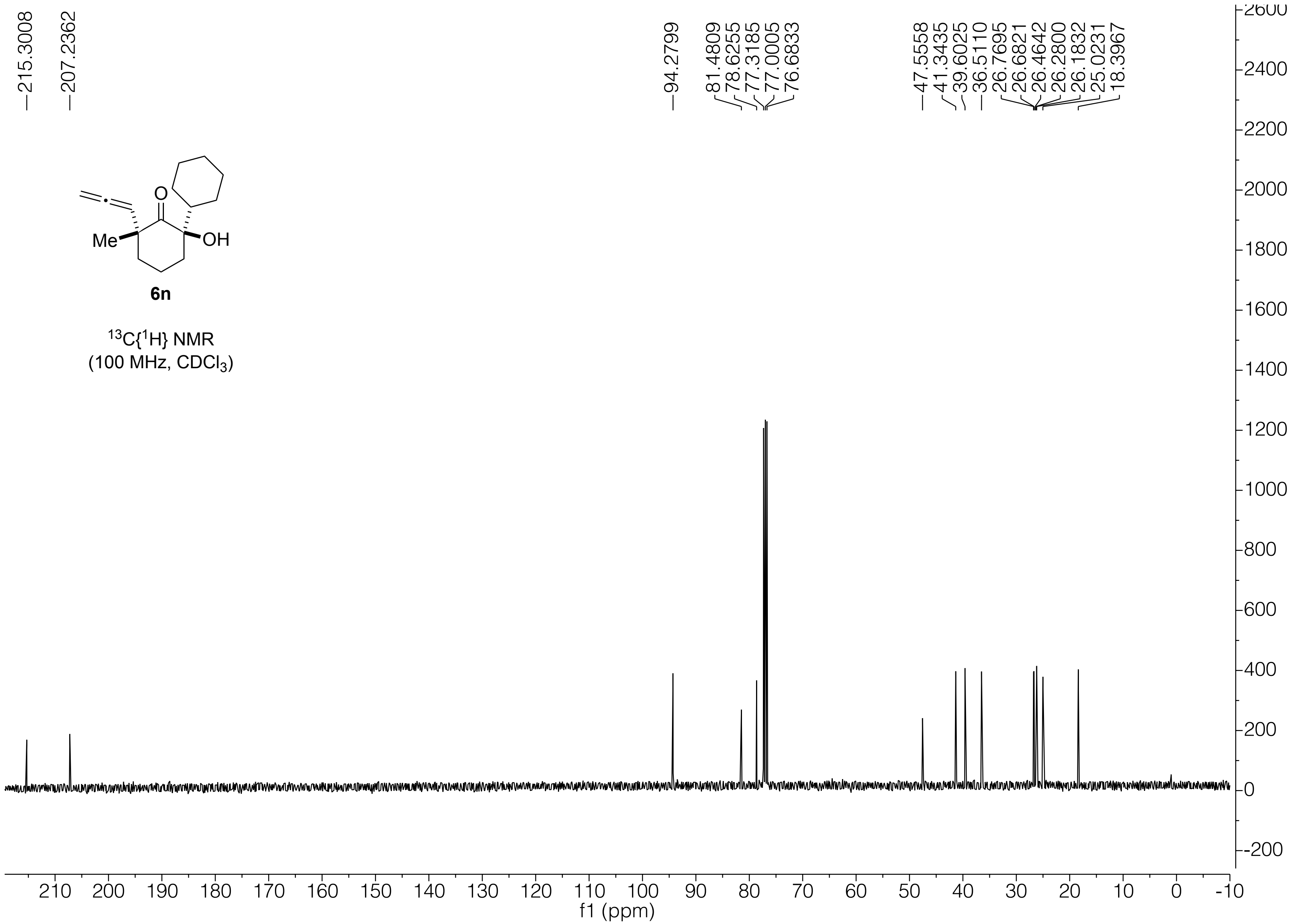
215.3008
207.2362
94.2799
81.4809
78.6255
77.3185
77.0005
76.6833
47.5558
41.3435
39.6025
36.5110
26.7695
26.6821
26.4642
26.2800
26.1832
25.0231
18.3967
O
Me
OH
6n
$^{13}C\{^1H\}$ NMR
(100 MHz, $CDCl_3$)
f1 (ppm)

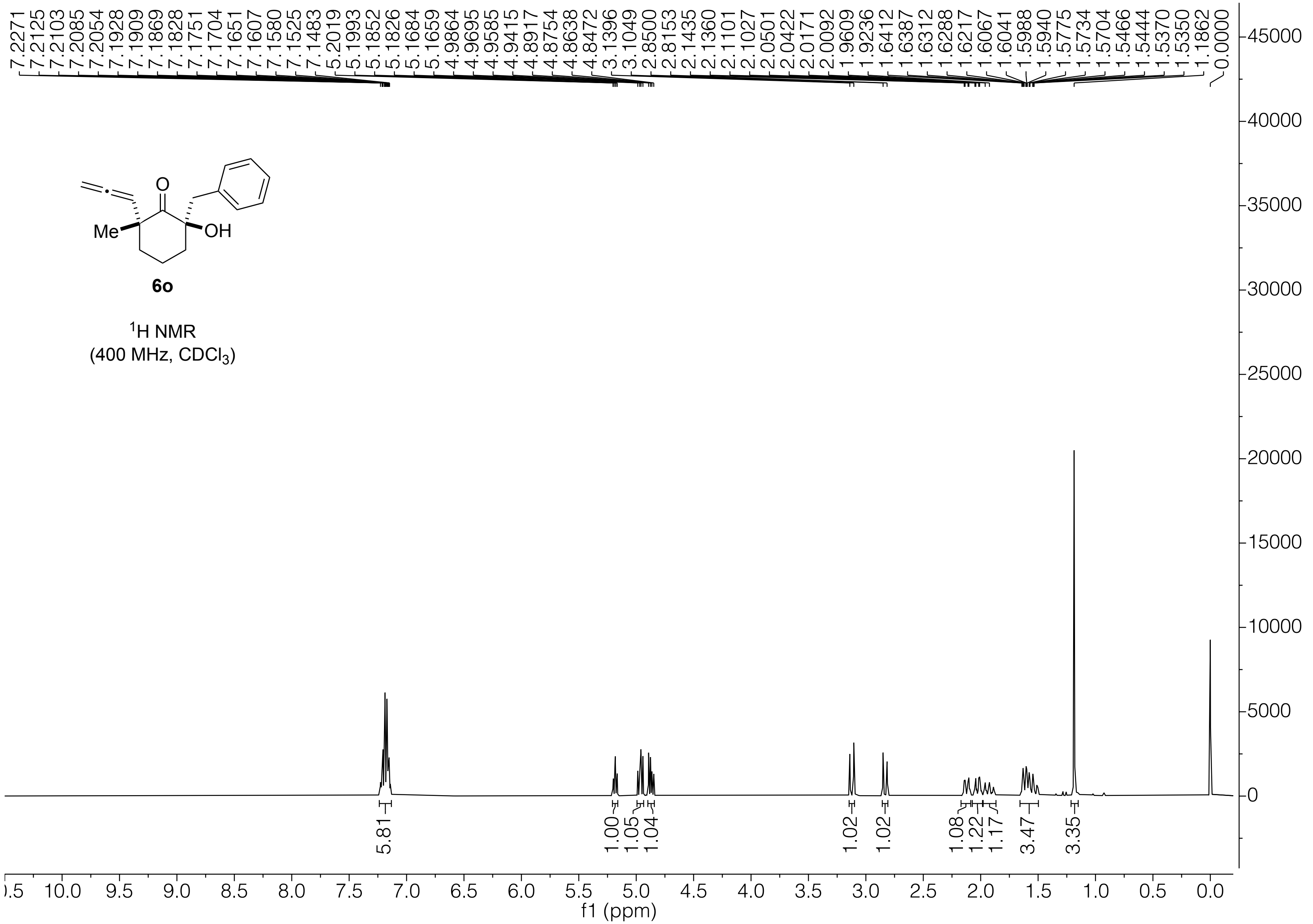
7.2271
7.2125
7.2103
7.2085
7.2054
7.1928
7.1909
7.1869
7.1828
7.1751
7.1704
7.1651
7.1607
7.1580
7.1525
7.1483
5.2019
5.1993
5.1852
5.1826
5.1684
5.1659
4.9864
4.9695
4.9585
4.9415
4.8917
4.8754
4.8638
4.8472
3.1396
3.1049
2.8500
2.8153
2.1435
2.1360
2.1101
2.1027
2.0501
2.0422
2.0171
2.0092
1.9609
1.9236
1.6412
1.6387
1.6312
1.6288
1.6217
1.6067
1.6041
1.5988
1.5940
1.5775
1.5734
1.5704
1.5466
1.5444
1.5370
1.5350
1.1862
0.0000
O
Me
OH
6o
^{1}H NMR
(400 MHz, $CDCl_3$)
5.81
1.00
1.05
1.04
1.02
1.02
1.08
1.22
1.17
3.47
3.35
f1 (ppm)

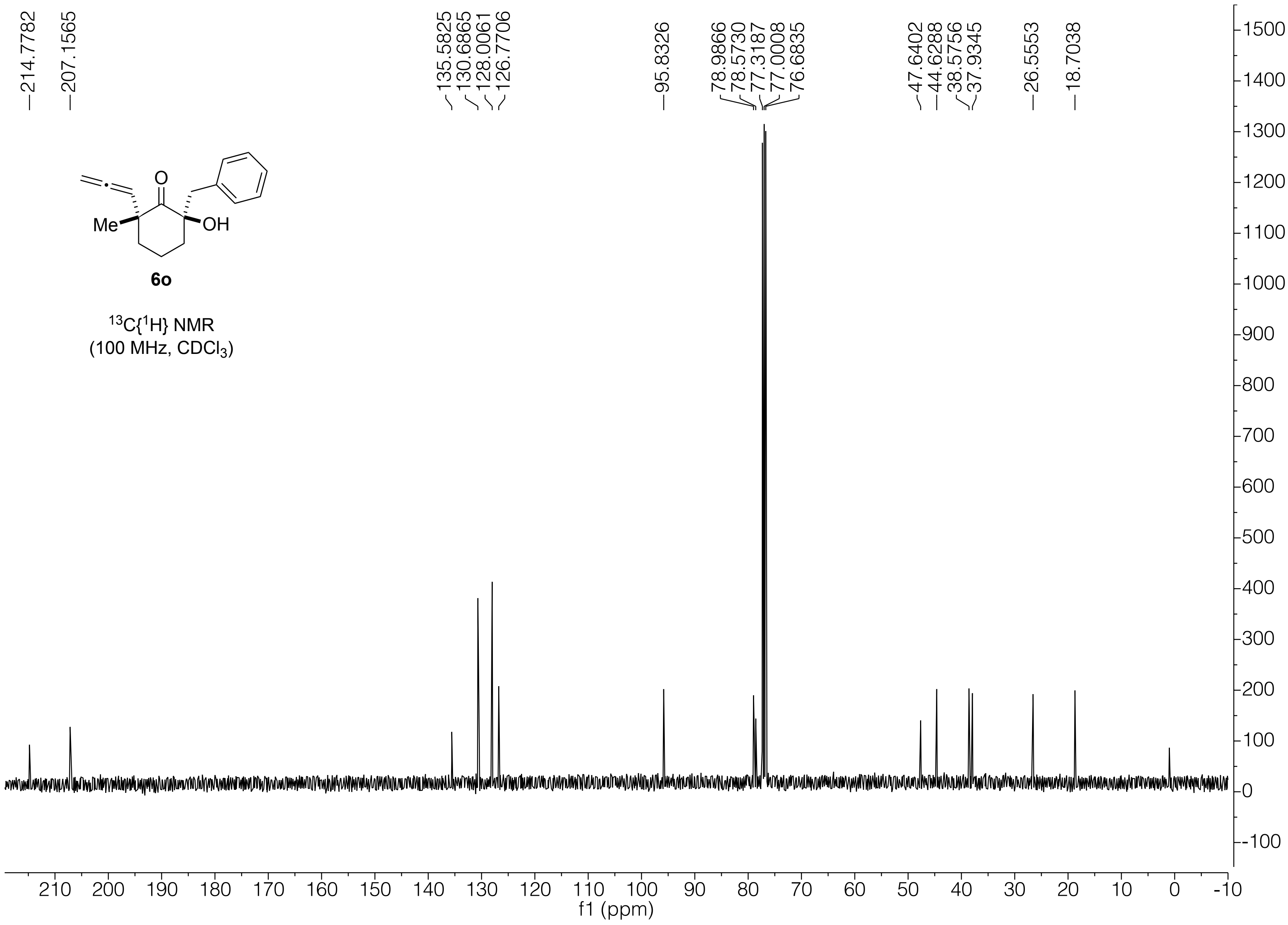
214.7782
207.1565
135.5825
130.6865
128.0061
126.7706
95.8326
78.9866
78.5730
77.3187
77.0008
76.6835
47.6402
44.6288
38.5756
37.9345
26.5553
18.7038
O
Me
OH
6o
$^{13}C\{^1H\}$ NMR
(100 MHz, $CDCl_3$)
f1 (ppm)

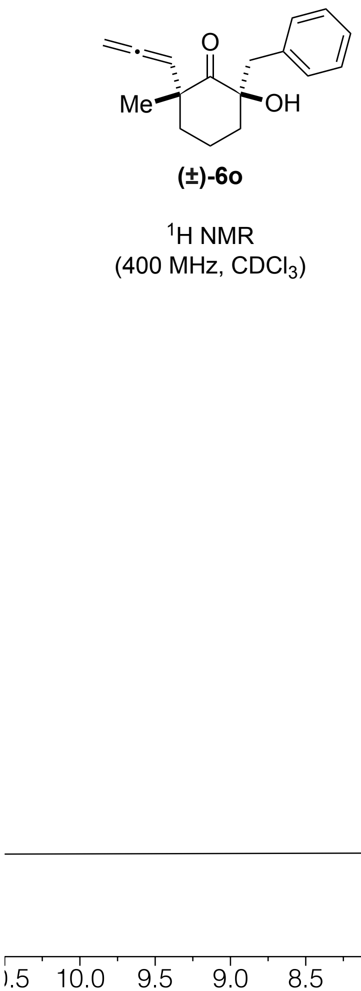

(±)-6o

^1H NMR
(400 MHz, $CDCl_3$)

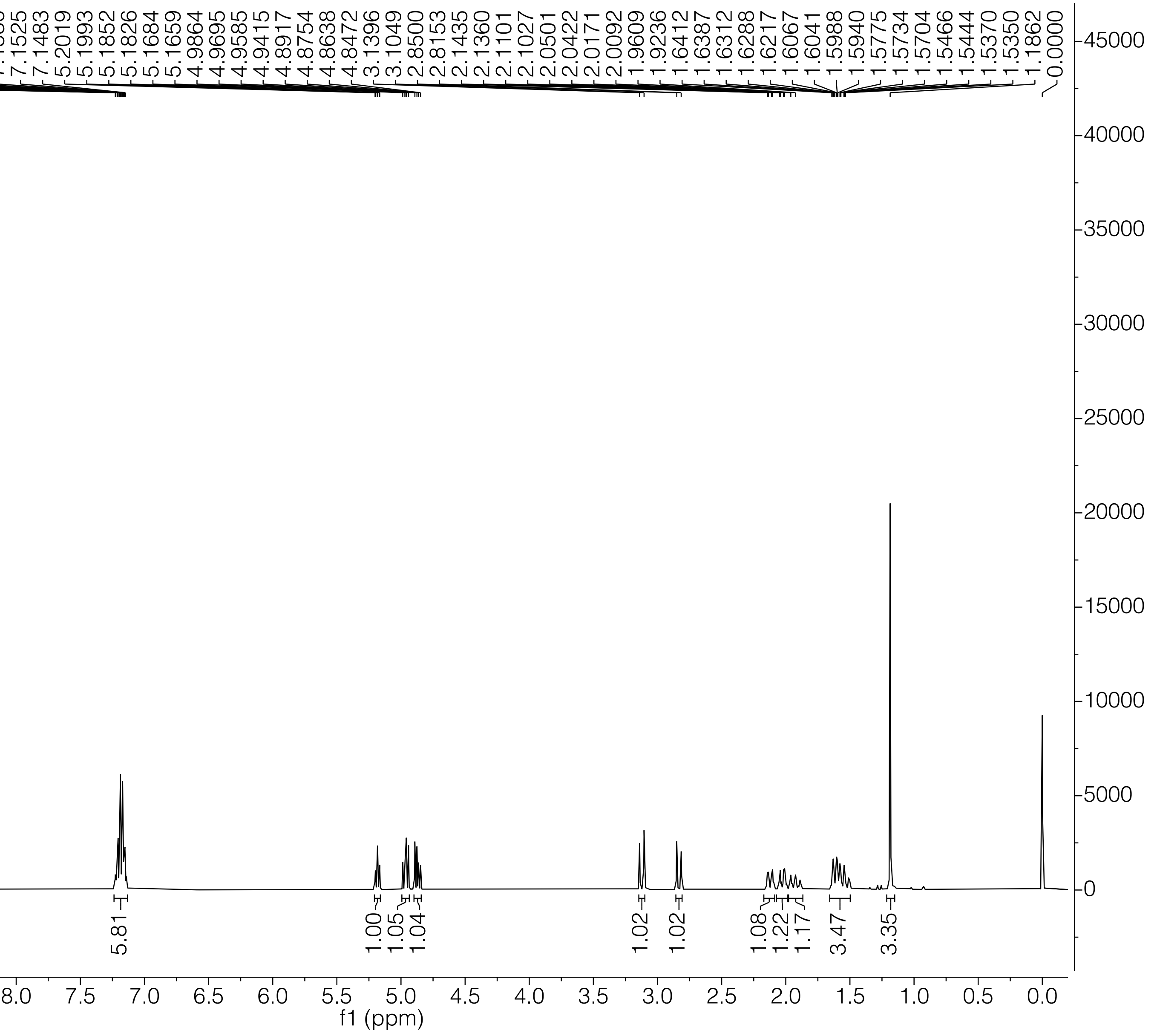

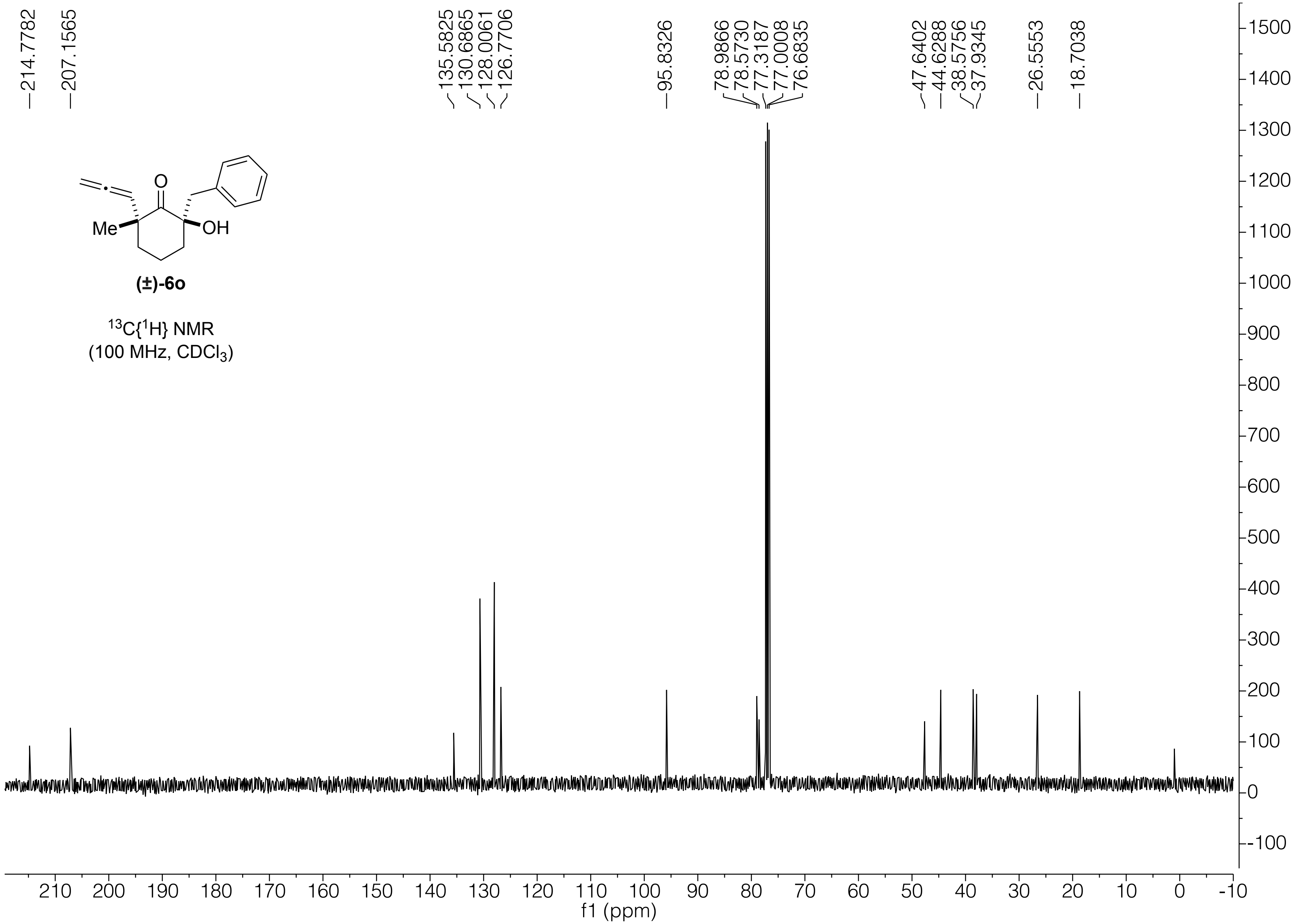
214.7782
207.1565
135.5825
130.6865
128.0061
126.7706
95.8326
78.9866
78.5730
77.3187
77.0008
76.6835
47.6402
44.6288
38.5756
37.9345
26.5553
18.7038
O
Me
OH
(±)-6o
$^{13}C\{^1H\}$ NMR
(100 MHz, $CDCl_3$)
f1 (ppm)

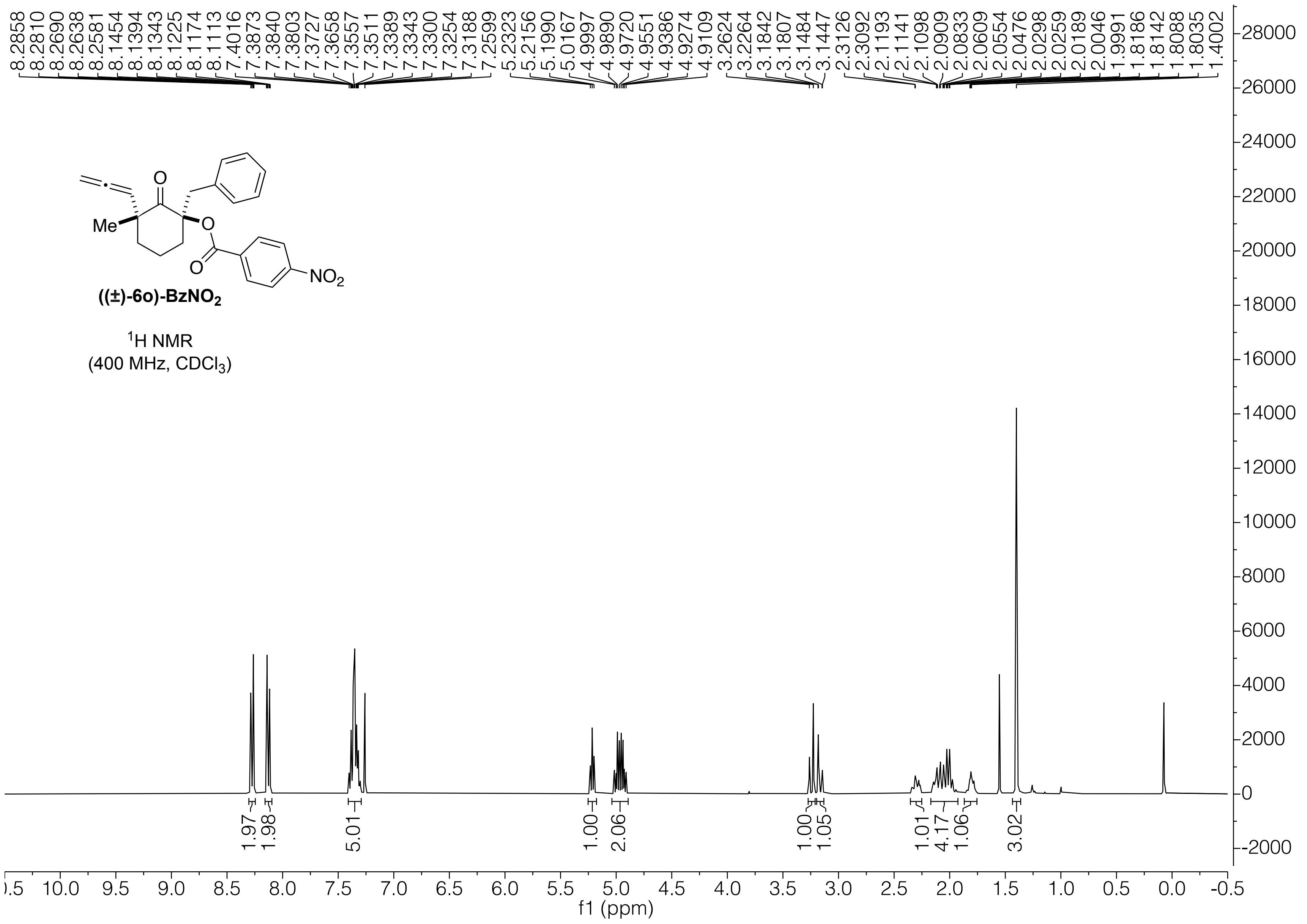

((±)-6o)-BzNO2
1H NMR
(400 MHz, CDCl3)
f1 (ppm)

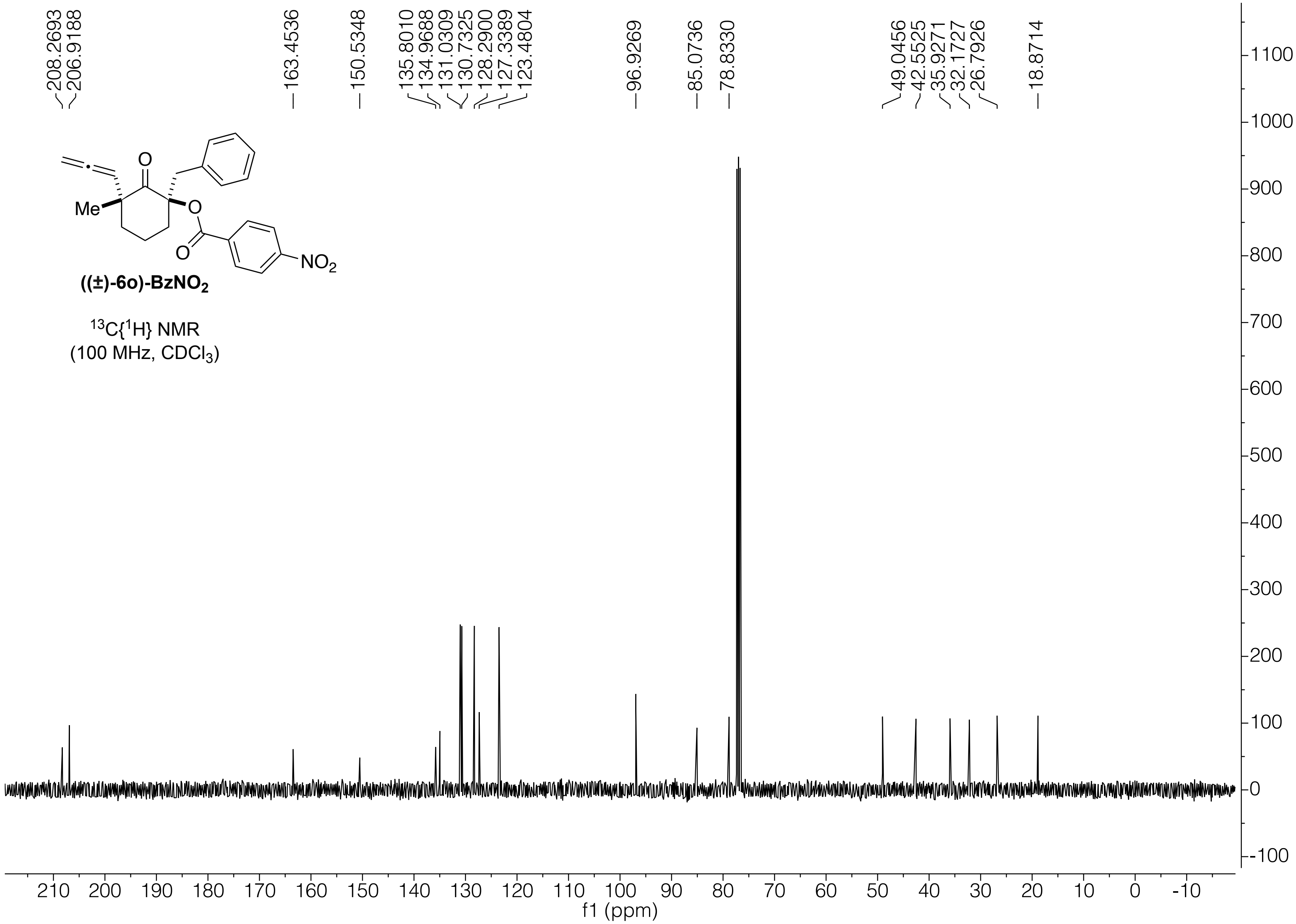
208.2693
206.9188
163.4536
150.5348
135.8010
134.9688
131.0309
130.7325
128.2900
127.3389
123.4804
96.9269
85.0736
78.8330
49.0456
42.5525
35.9271
32.1727
26.7926
18.8714
O
Me
O
O
NO2
((±)-6o)-BzNO2
13C{1H} NMR
(100 MHz, CDCl3)
1100
1000
900
800
700
600
500
400
300
200
100
0
-100
210
200
190
180
170
160
150
140
130
120
110
100
90
80
70
60
50
40
30
20
10
0
-10
f1 (ppm)

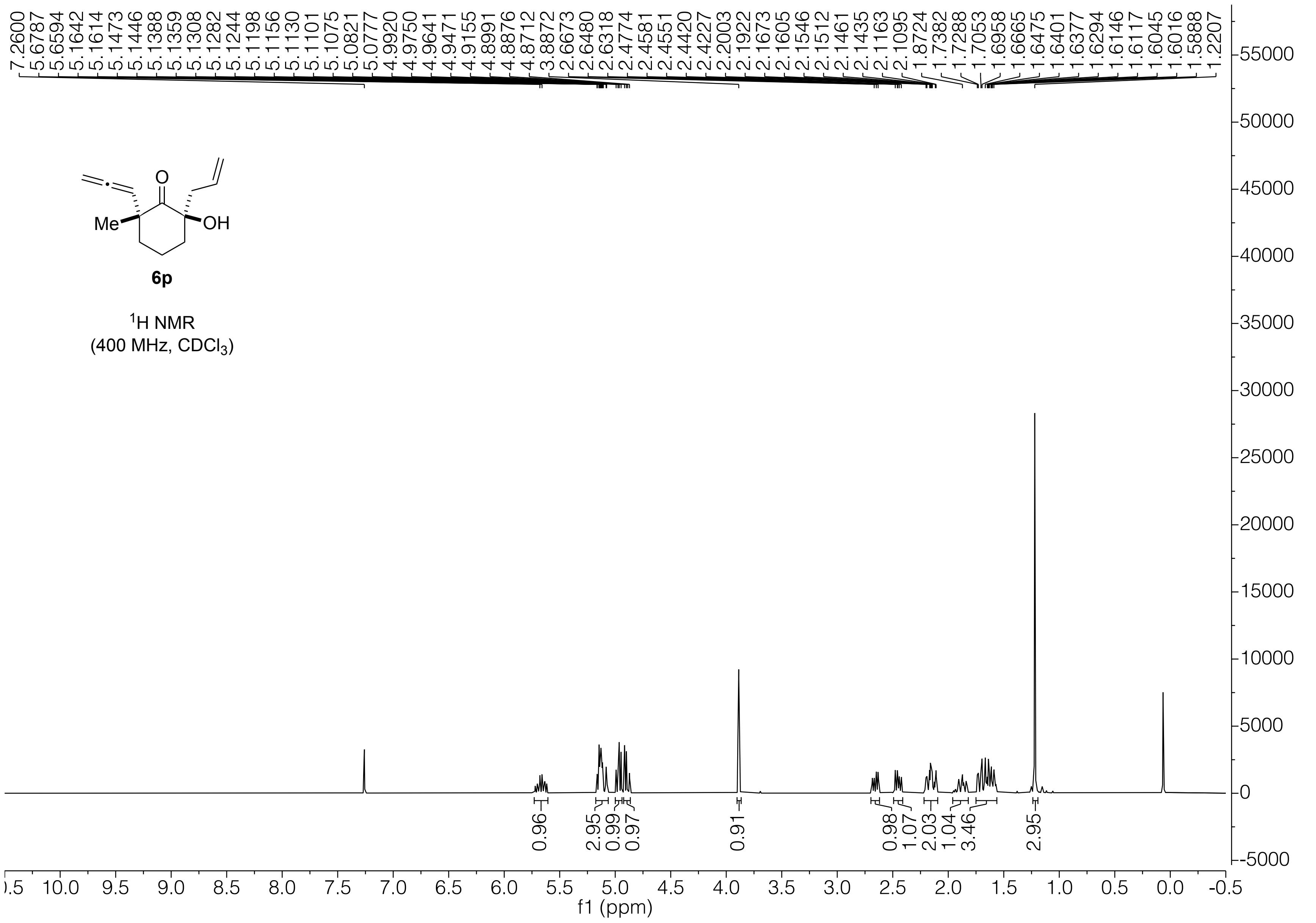
7.2600
5.6787
5.6594
5.1642
5.1614
5.1473
5.1446
5.1388
5.1359
5.1308
5.1282
5.1244
5.1198
5.1156
5.1130
5.1101
5.1075
5.0821
5.0777
4.9920
4.9750
4.9641
4.9471
4.9155
4.8991
4.8876
4.8712
3.8872
2.6673
2.6480
2.6318
2.4774
2.4581
2.4551
2.4420
2.4227
2.2003
2.1922
2.1673
2.1605
2.1546
2.1512
2.1461
2.1435
2.1163
2.1095
1.8724
1.7382
1.7288
1.7053
1.6958
1.6665
1.6475
1.6401
1.6377
1.6294
1.6146
1.6117
1.6045
1.6016
1.5888
1.2207
O
Me
OH
6p
^{1}H NMR
(400 MHz, $CDCl_3$)
0.96
2.95
0.99
0.97
0.91
0.98
1.07
2.03
1.04
3.46
2.95
f1 (ppm)

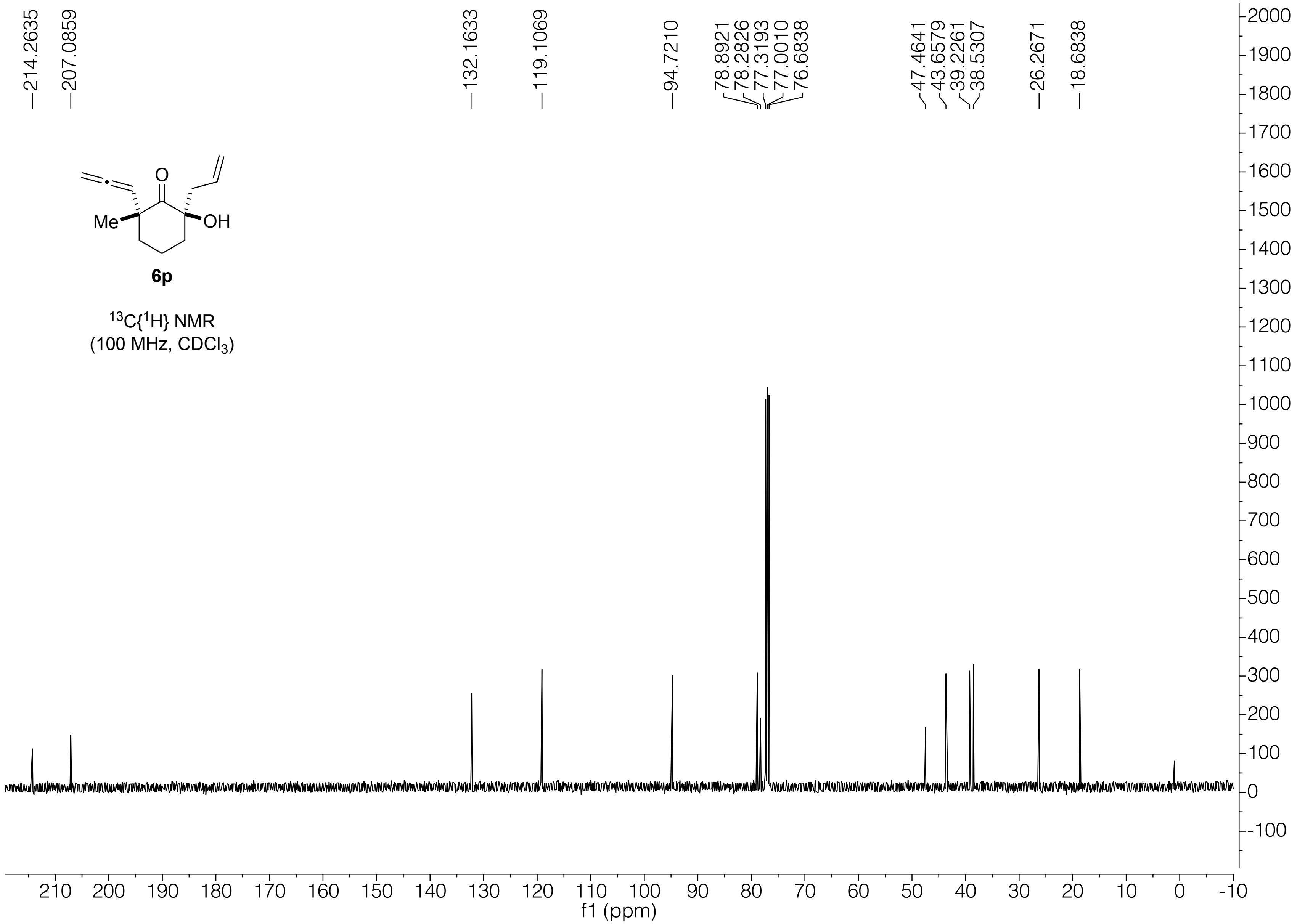
214.2635
207.0859
132.1633
119.1069
94.7210
78.8921
78.2826
77.3193
77.0010
76.6838
47.4641
43.6579
39.2261
38.5307
26.2671
18.6838
O
Me
OH
6p
$^{13}C\{^1H\}$ NMR
(100 MHz, $CDCl_3$)
f1 (ppm)

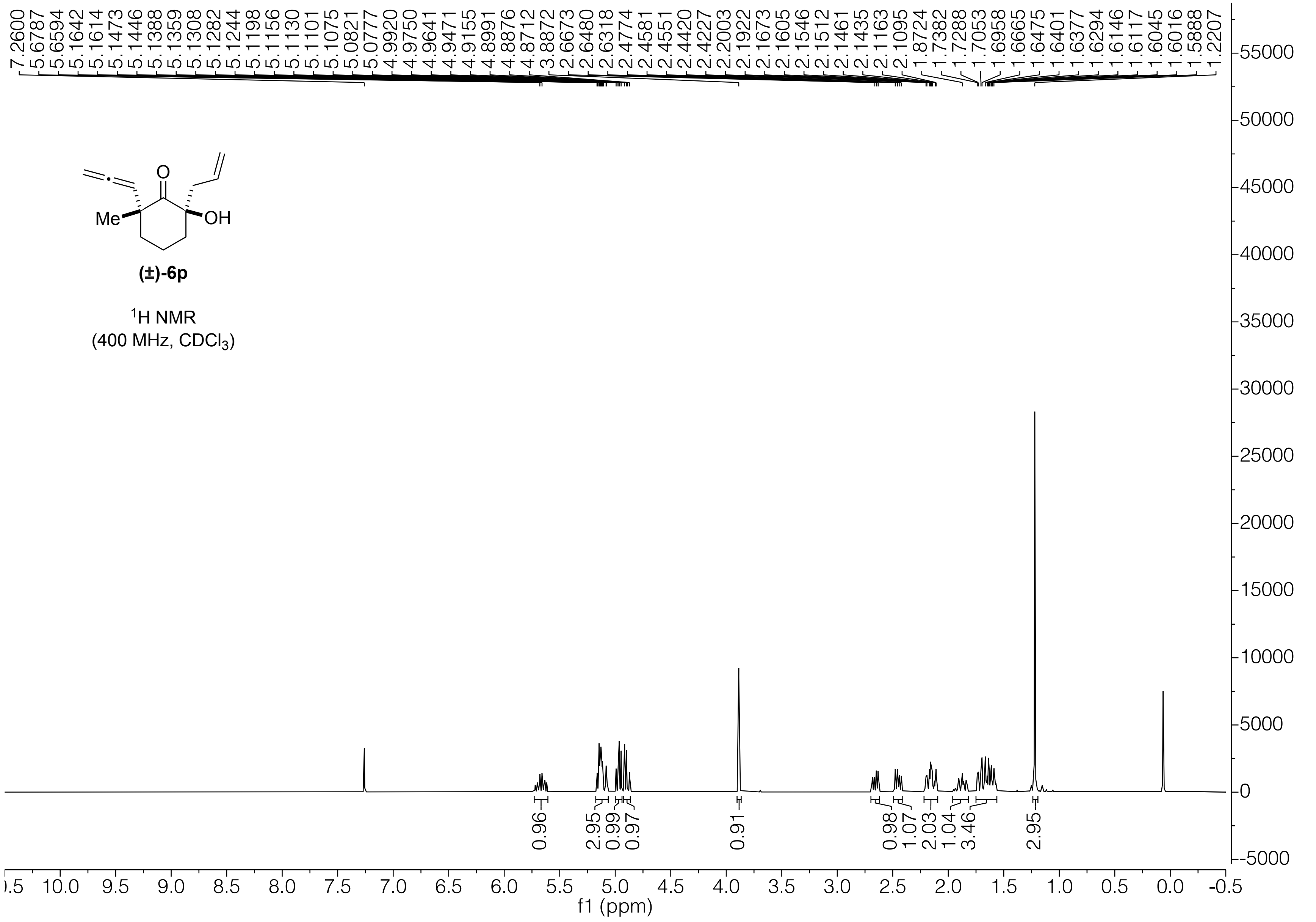

(±)-6p
^{1}H NMR
(400 MHz, $CDCl_3$)
7.2600
5.6787
5.6594
5.1642
5.1614
5.1473
5.1446
5.1388
5.1359
5.1308
5.1282
5.1244
5.1198
5.1156
5.1130
5.1101
5.1075
5.0821
5.0777
4.9920
4.9750
4.9641
4.9471
4.9155
4.8991
4.8876
4.8712
3.8872
2.6673
2.6480
2.6318
2.4774
2.4581
2.4551
2.4420
2.4227
2.2003
2.1922
2.1673
2.1605
2.1546
2.1512
2.1461
2.1435
2.1163
2.1095
1.8724
1.7382
1.7288
1.7053
1.6958
1.6665
1.6475
1.6401
1.6377
1.6294
1.6146
1.6117
1.6045
1.6016
1.5888
1.2207
0.96
2.95
0.99
0.97
0.91
0.98
1.07
2.03
1.04
3.46
2.95
f1 (ppm)

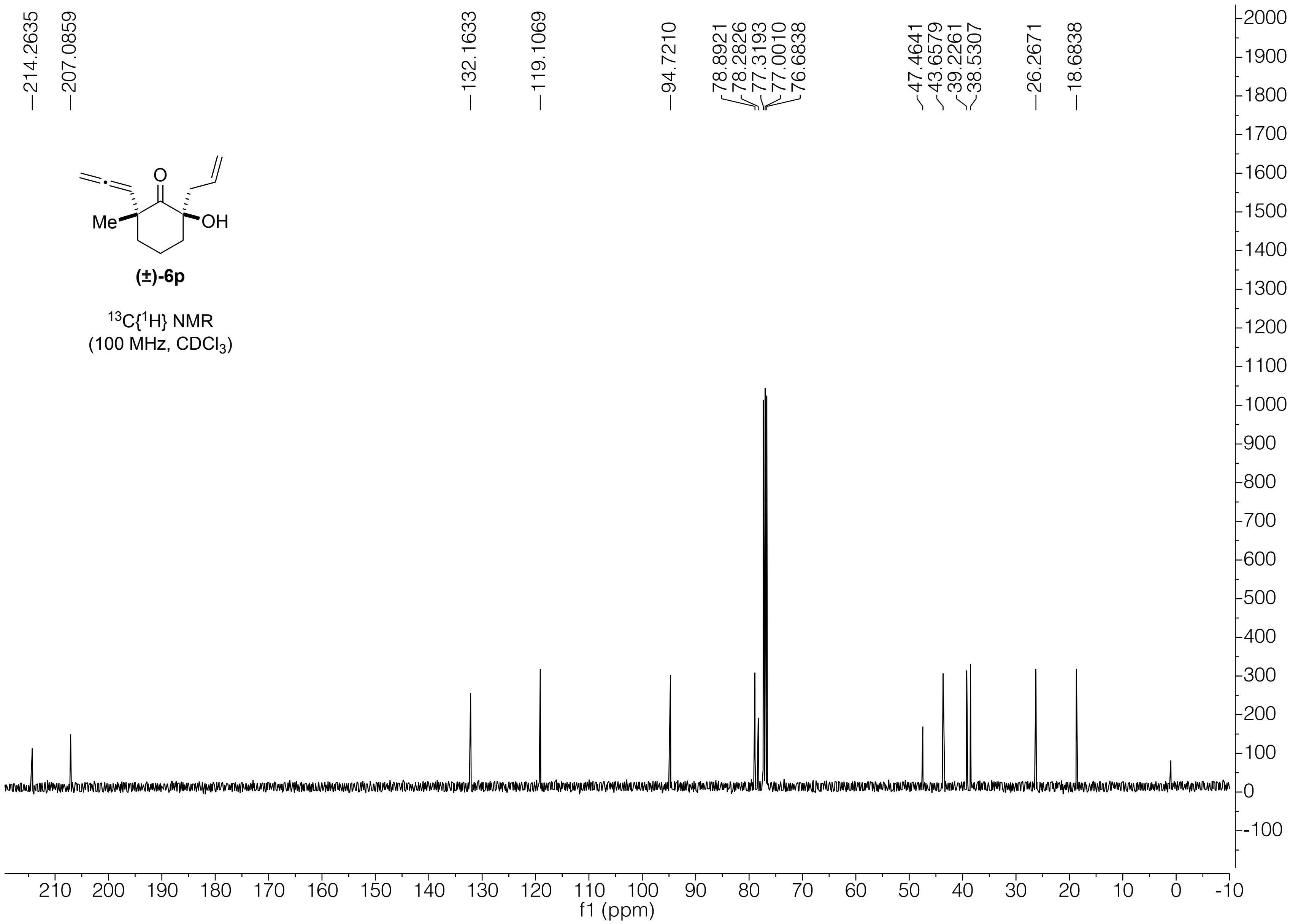

214.2635
207.0859
132.1633
119.1069
94.7210
78.8921
78.2826
77.3193
77.0010
76.6838
47.4641
43.6579
39.2261
38.5307
26.2671
18.6838
O
Me
OH
(±)-6p
13C{1H} NMR
(100 MHz, CDCl3)
f1 (ppm)

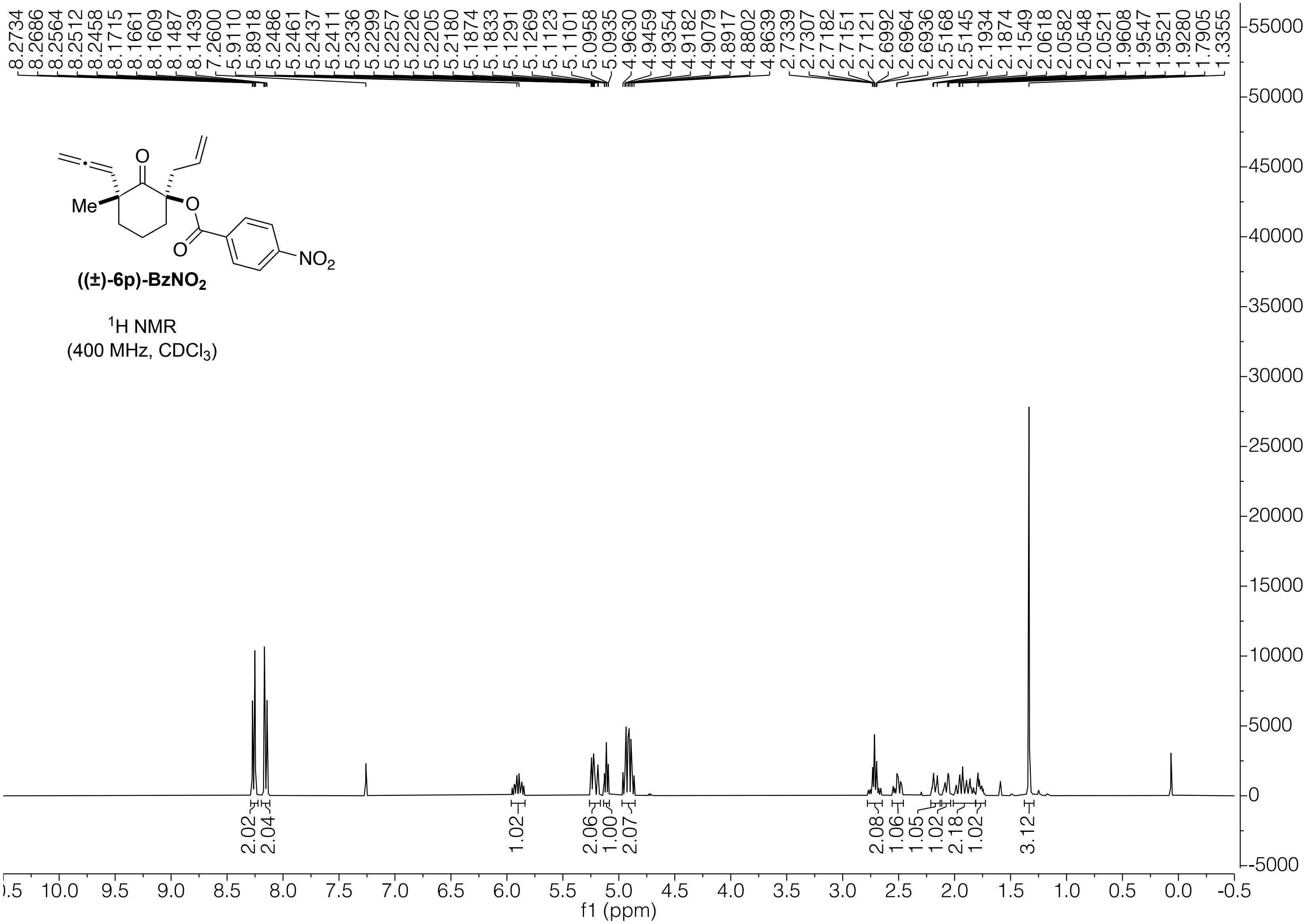

((±)-6p)-BzNO2
1H NMR
(400 MHz, CDCl3)
f1 (ppm)

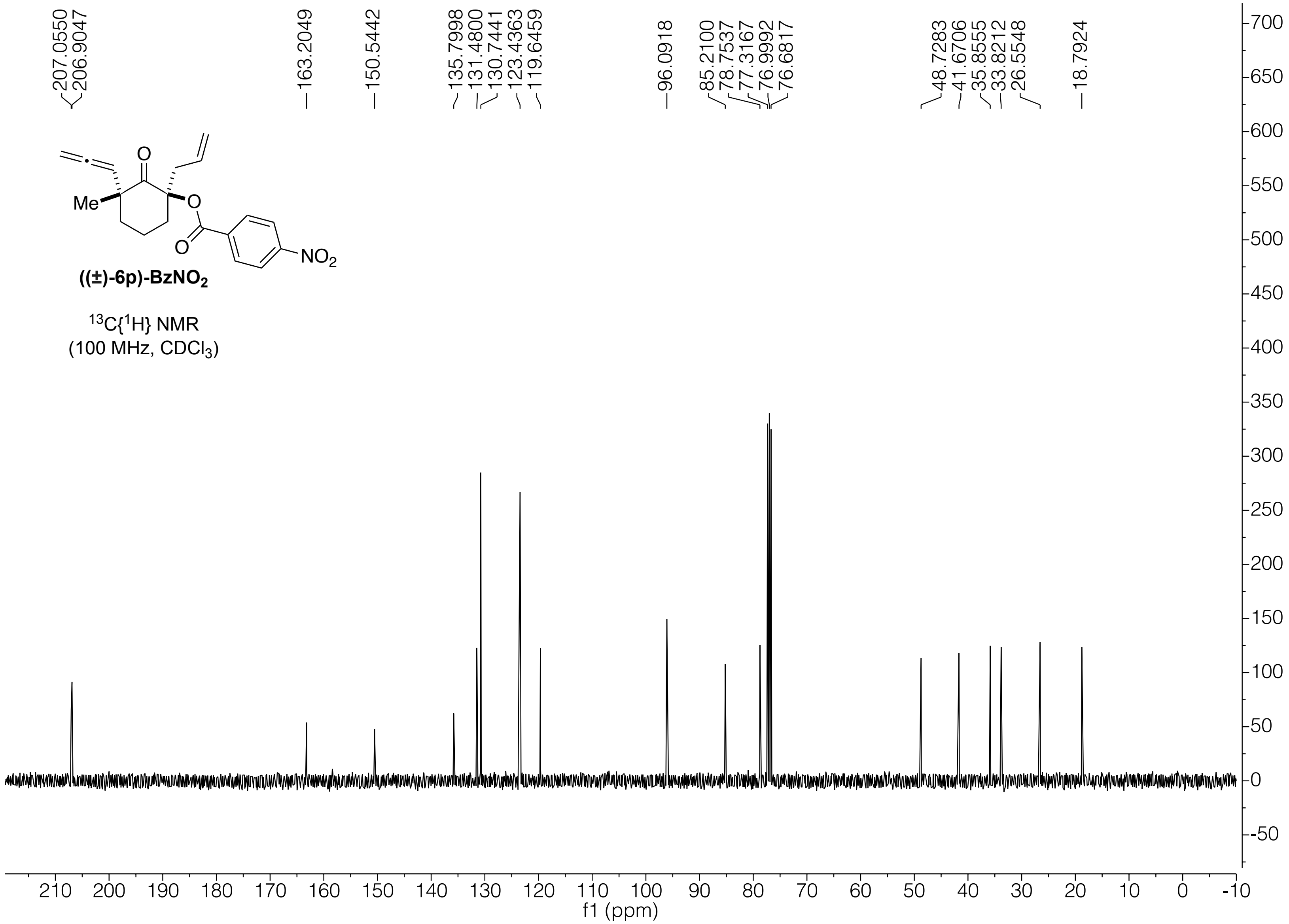

207.0550
206.9047
163.2049
150.5442
135.7998
131.4800
130.7441
123.4363
119.6459
96.0918
85.2100
78.7537
77.3167
76.9992
76.6817
48.7283
41.6706
35.8555
33.8212
26.5548
18.7924
O
Me
O
O
NO2
((±)-6p)-BzNO2
13C{1H} NMR
(100 MHz, CDCl3)
f1 (ppm)

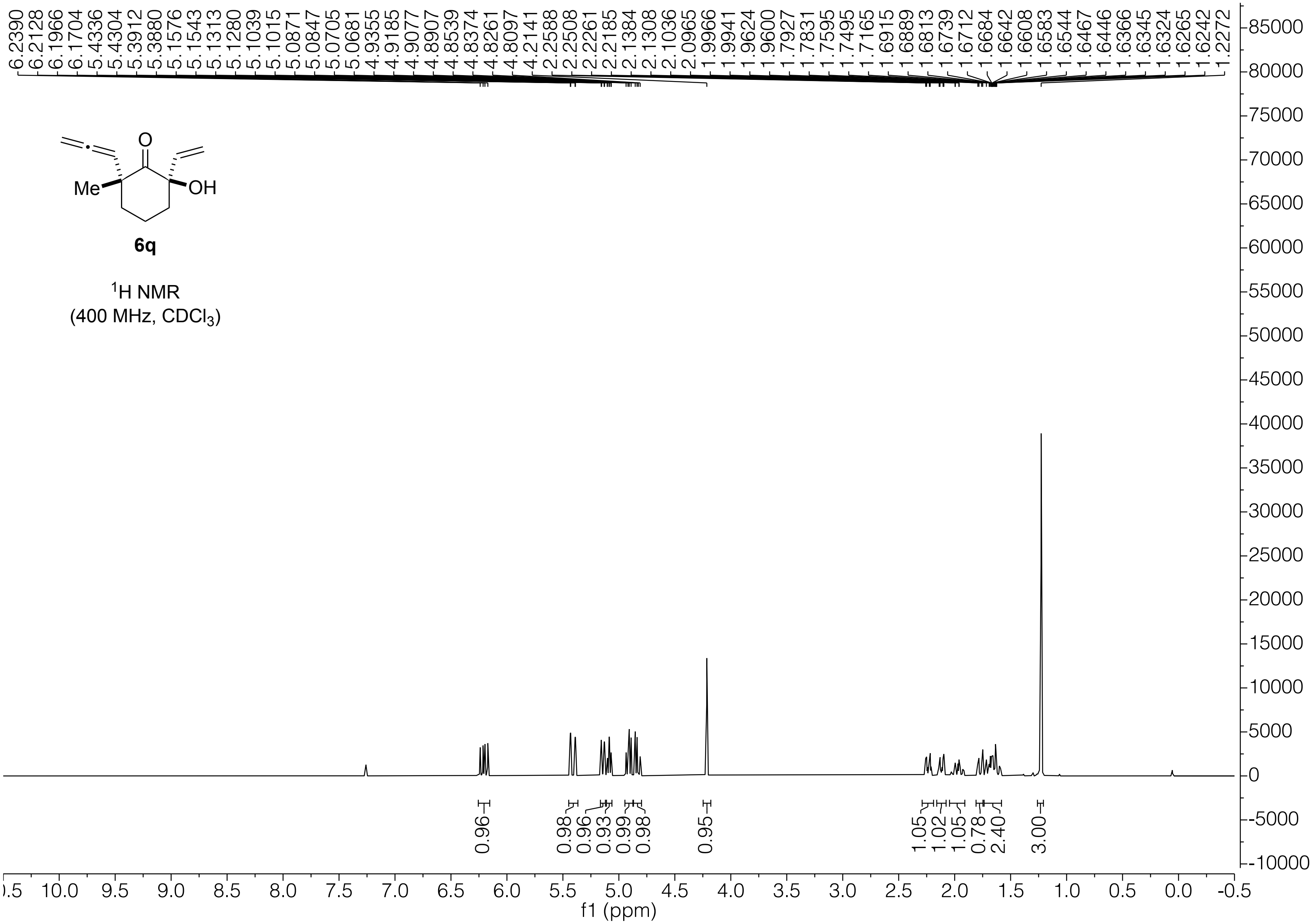

Me
OH
O
6q
¹H NMR
(400 MHz, CDCl₃)
f1 (ppm)

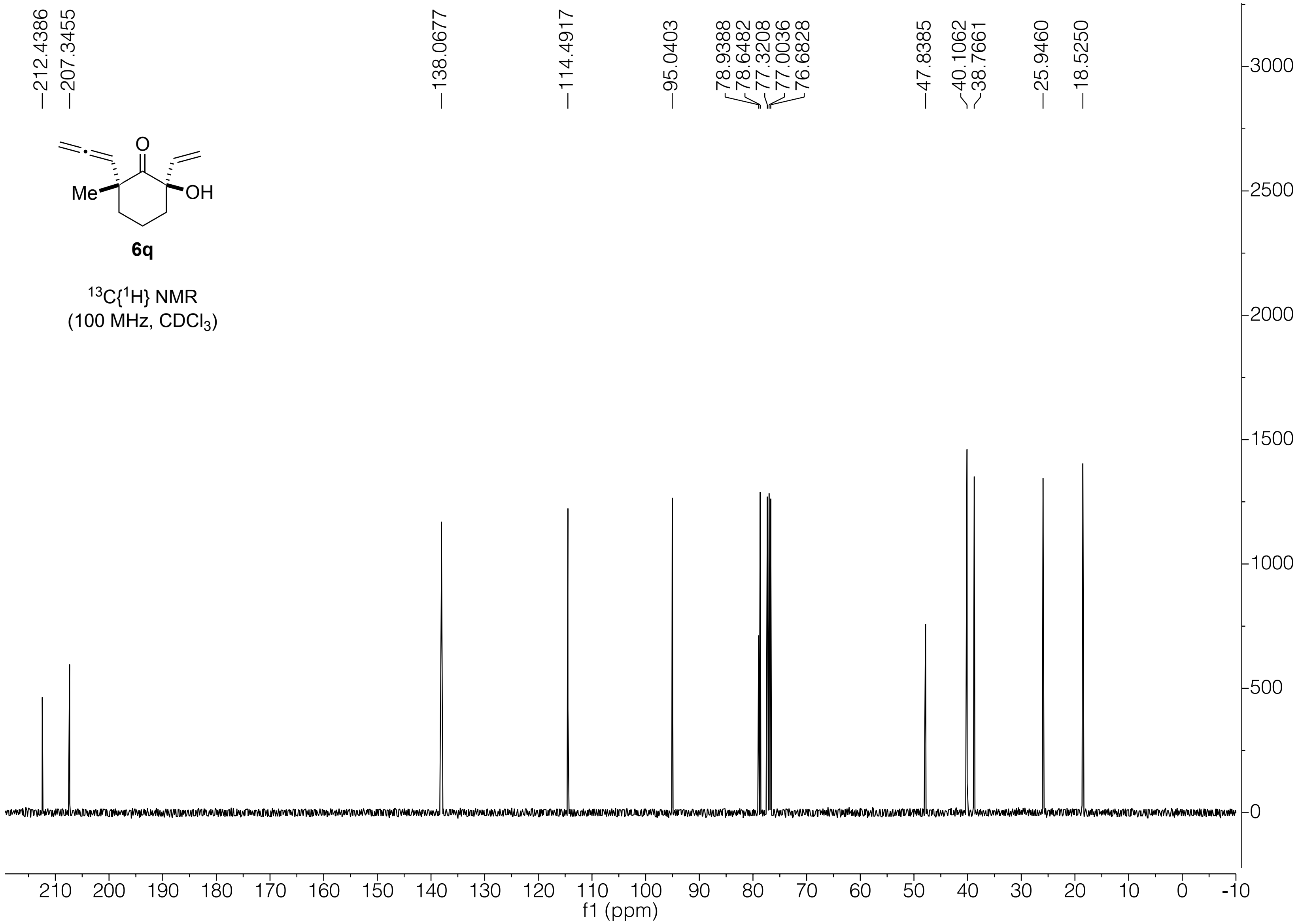
212.4386
207.3455
138.0677
114.4917
95.0403
78.9388
78.6482
77.3208
77.0036
76.6828
47.8385
40.1062
38.7661
25.9460
18.5250
O
Me
OH
6q
$^{13}C\{^1H\}$ NMR
(100 MHz, $CDCl_3$)
3000
2500
2000
1500
1000
500
0
210 200 190 180 170 160 150 140 130 120 110 100 90 80 70 60 50 40 30 20 10 0 -10
f1 (ppm)

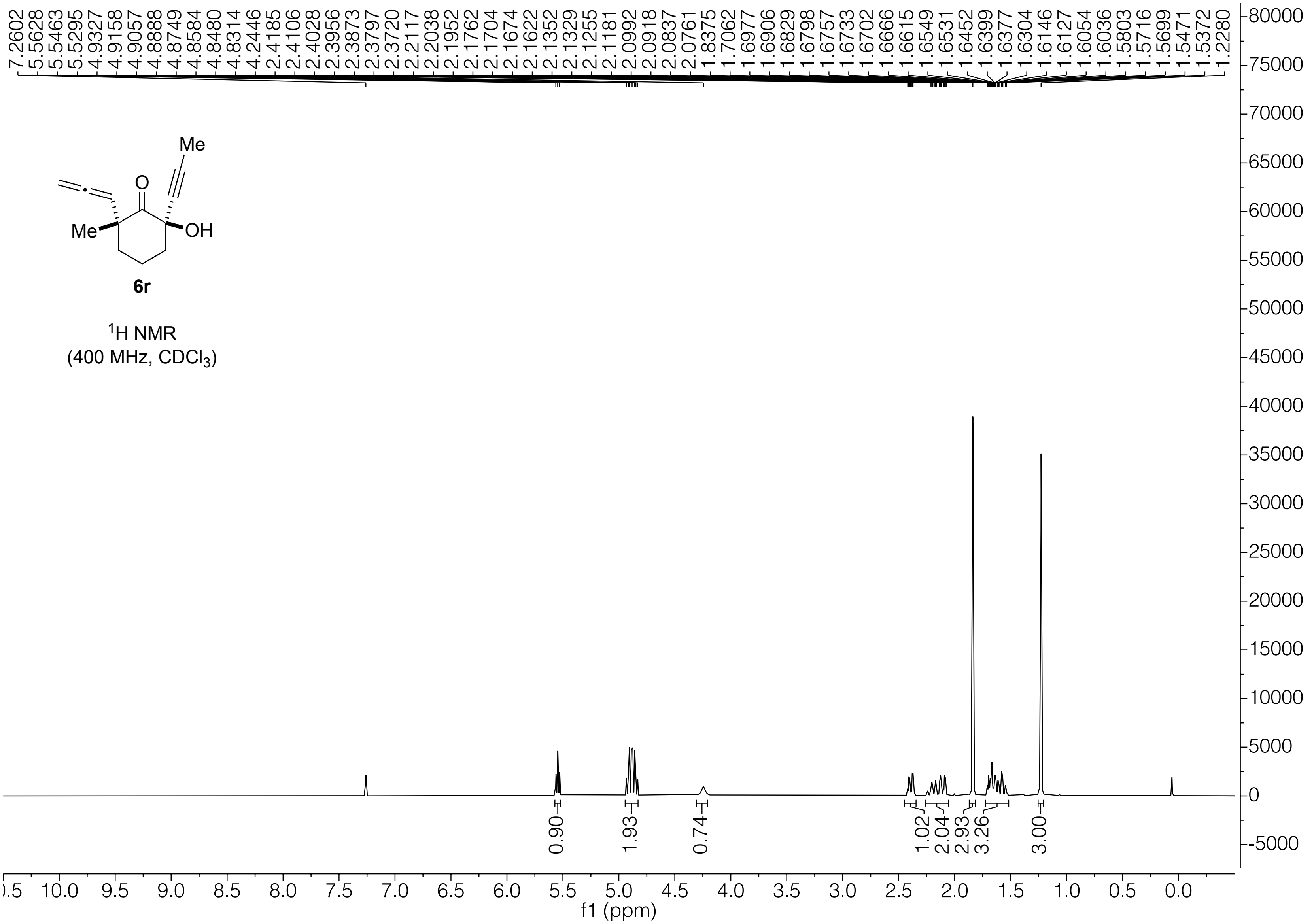

Me
O
Me
OH
6r
1H NMR
(400 MHz, CDCl3)
7.2602 5.5628 5.5463 5.5295 4.9327 4.9158 4.9057 4.8888 4.8749 4.8584 4.8480 4.8314 4.2446 4.2185 2.4106 2.4028 2.3956 2.3873 2.3797 2.3720 2.2117 2.2038 2.1952 2.1762 2.1704 2.1674 2.1622 2.1352 2.1329 2.1255 2.1181 2.0992 2.0918 2.0837 2.0761 1.8375 1.7062 1.6977 1.6906 1.6829 1.6798 1.6757 1.6733 1.6702 1.6666 1.6615 1.6549 1.6531 1.6452 1.6399 1.6377 1.6304 1.6146 1.6127 1.6054 1.6036 1.5803 1.5716 1.5699 1.5471 1.5372 1.2280
0.90 1.93 0.74 1.02 2.04 2.93 3.26 3.00
f1 (ppm)

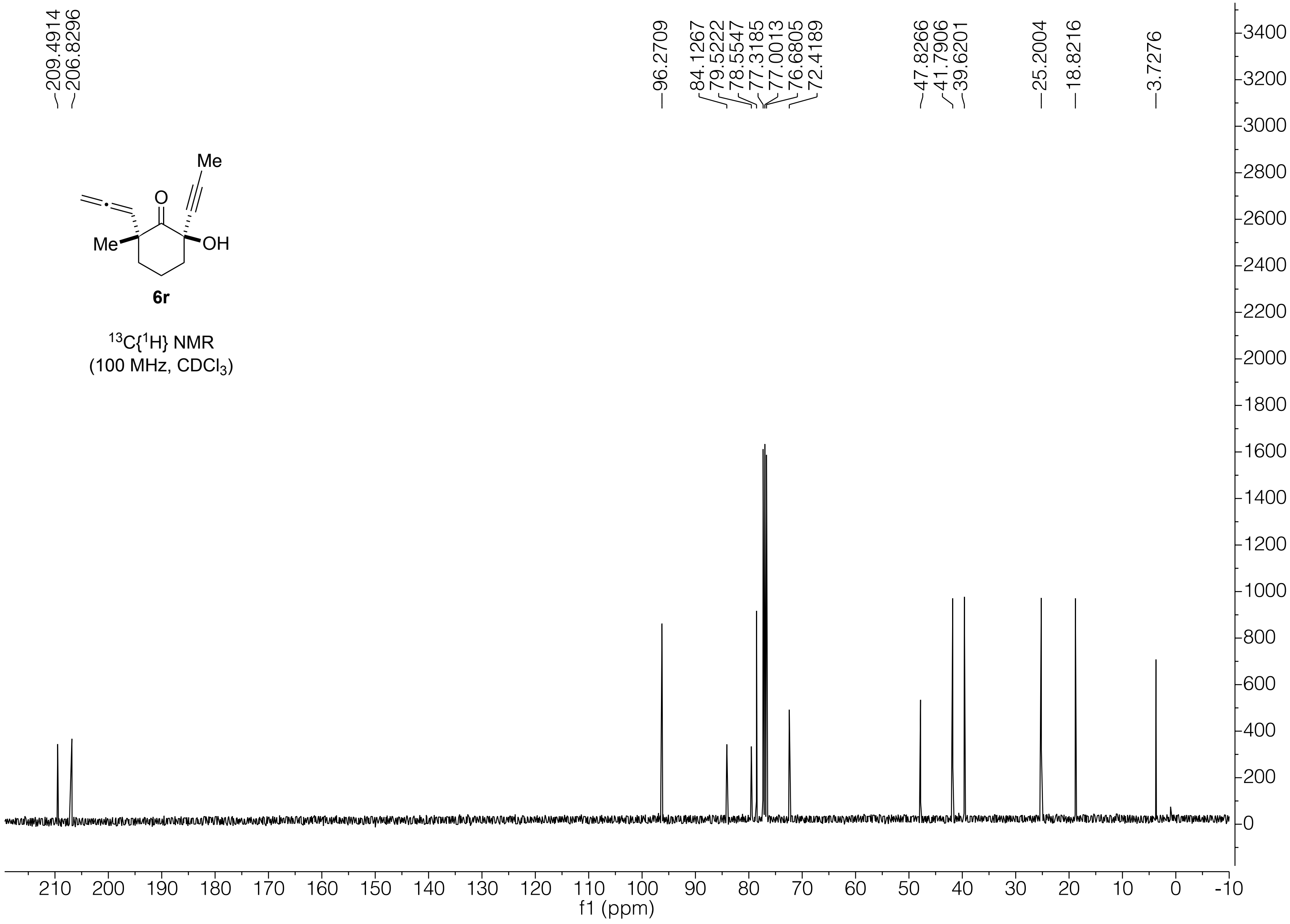
209.4914
206.8296
96.2709
84.1267
79.5222
78.5547
77.3185
77.0013
76.6805
72.4189
47.8266
41.7906
39.6201
25.2004
18.8216
3.7276
Me
O
OH
Me
6r
$^{13}C\{^{1}H\}$ NMR
(100 MHz, $CDCl_3$)
f1 (ppm)

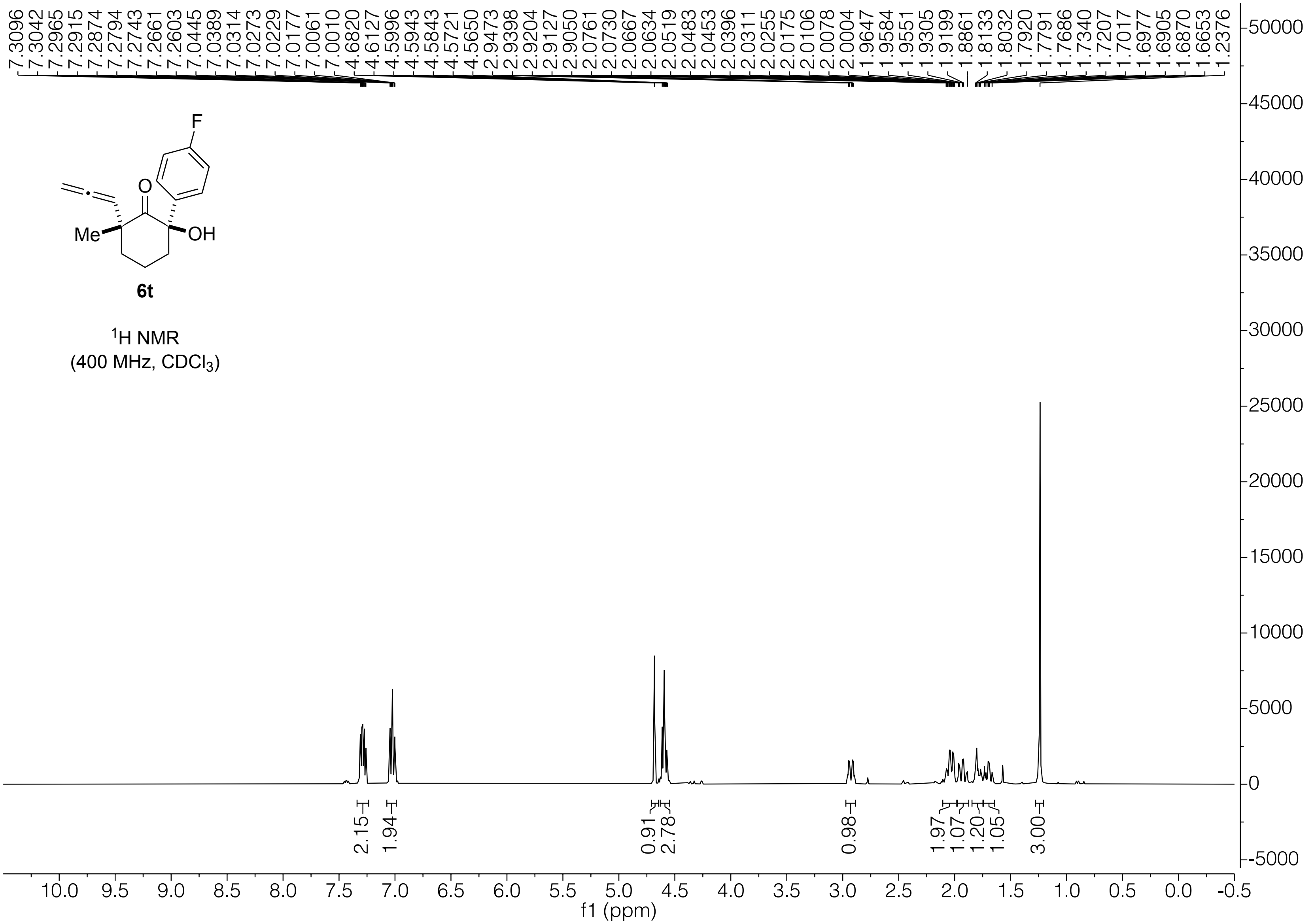

7.3096
7.3042
7.2965
7.2915
7.2874
7.2794
7.2743
7.2661
7.2603
7.0445
7.0389
7.0314
7.0273
7.0229
7.0177
7.0061
7.0010
4.6820
4.6127
4.5996
4.5943
4.5843
4.5721
4.5650
2.9473
2.9398
2.9204
2.9127
2.9050
2.0761
2.0730
2.0667
2.0634
2.0519
2.0483
2.0453
2.0396
2.0311
2.0255
2.0175
2.0106
2.0078
2.0004
1.9647
1.9584
1.9551
1.9305
1.9199
1.8861
1.8133
1.8032
1.7920
1.7791
1.7686
1.7340
1.7207
1.7017
1.6977
1.6905
1.6870
1.6653
1.2376
F
O
Me
OH
6t
1H NMR
(400 MHz, CDCl3)
2.15
1.94
0.91
2.78
0.98
1.97
1.07
1.20
1.05
3.00
f1 (ppm)

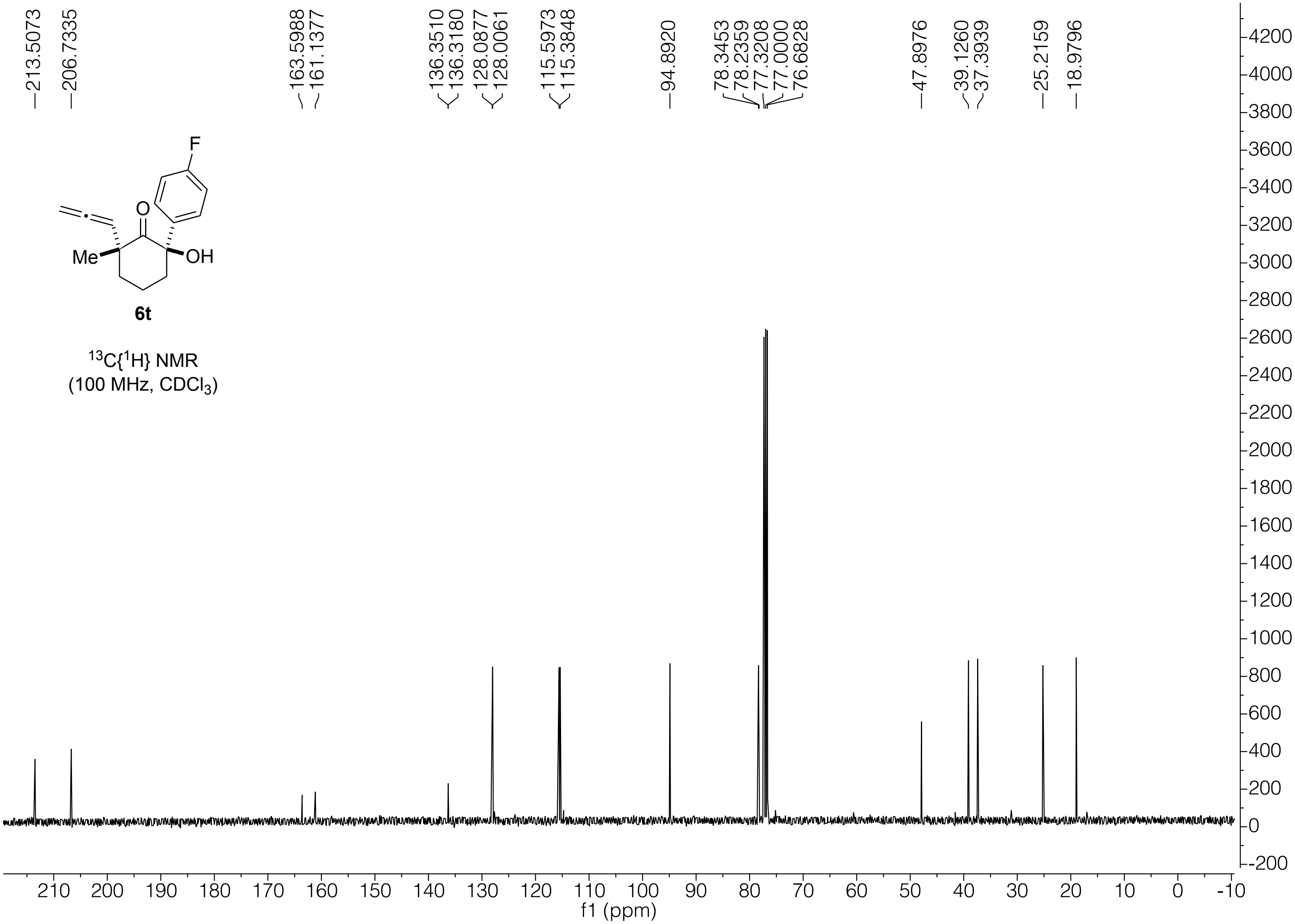

213.5073
206.7335
163.5988
161.1377
136.3510
136.3180
128.0877
128.0061
115.5973
115.3848
94.8920
78.3453
78.2359
77.3208
77.0000
76.6828
47.8976
39.1260
37.3939
25.2159
18.9796
F
O
Me
OH
6t
$^{13}C\{^{1}H\}$ NMR
(100 MHz, $CDCl_3$)
f1 (ppm)

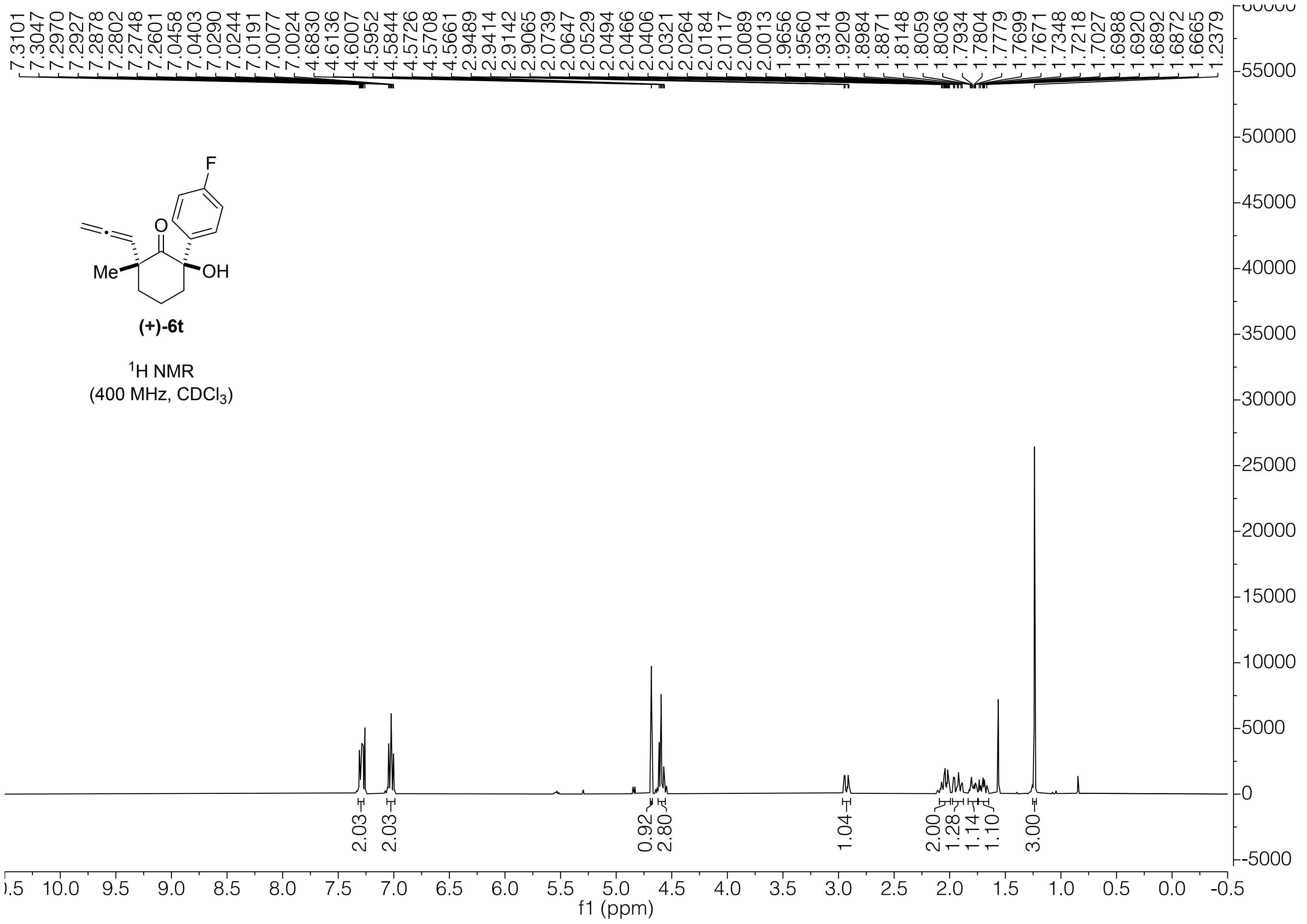

7.3101 7.3047 7.2970 7.2927 7.2878 7.2802 7.2748 7.2601 7.0458 7.0403 7.0290 7.0244 7.0191 7.0077 7.0024 4.6830 4.6136 4.6007 4.5952 4.5844 4.5726 4.5708 4.5661 2.9489 2.9414 2.9142 2.9065 2.0739 2.0647 2.0529 2.0494 2.0466 2.0406 2.0321 2.0264 2.0184 2.0117 2.0089 2.0013 1.9656 1.9560 1.9314 1.9209 1.8984 1.8871 1.8148 1.8059 1.8036 1.7934 1.7804 1.7779 1.7699 1.7671 1.7348 1.7218 1.7027 1.6988 1.6920 1.6892 1.6872 1.6665 1.2379
F
O
Me
OH
(+)-6t
¹H NMR
(400 MHz, CDCl3)
2.03
2.03
0.92
2.80
1.04
2.00
1.28
1.14
1.10
3.00
f1 (ppm)

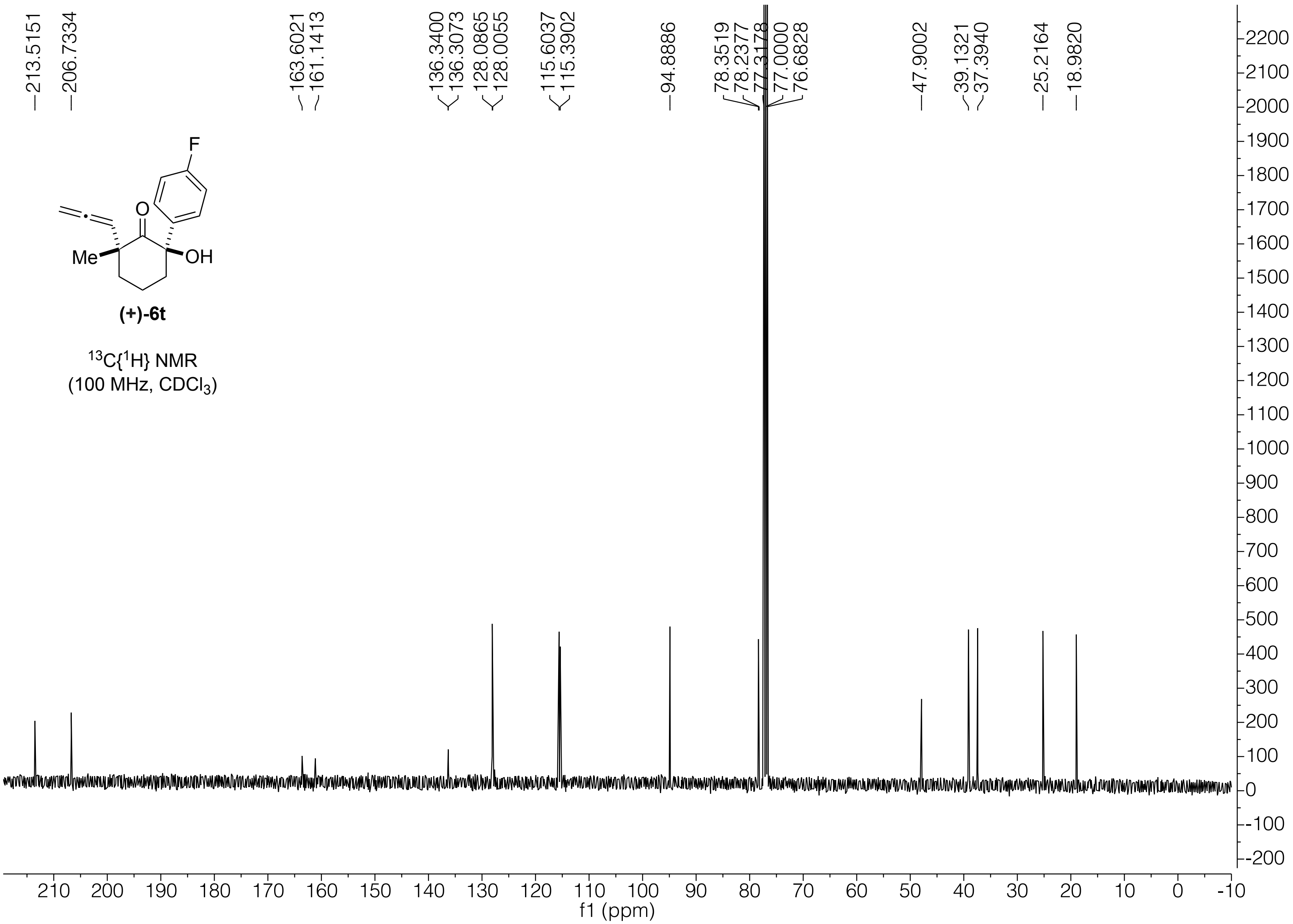

213.5151
206.7334
163.6021
161.1413
136.3400
136.3073
128.0865
128.0055
115.6037
115.3902
94.8886
78.3519
78.2377
77.3178
77.0000
76.6828
47.9002
39.1321
37.3940
25.2164
18.9820
F
O
Me
OH
(+)-6t
$^{13}C\{^{1}H\}$ NMR
(100 MHz, $CDCl_3$)
f1 (ppm)

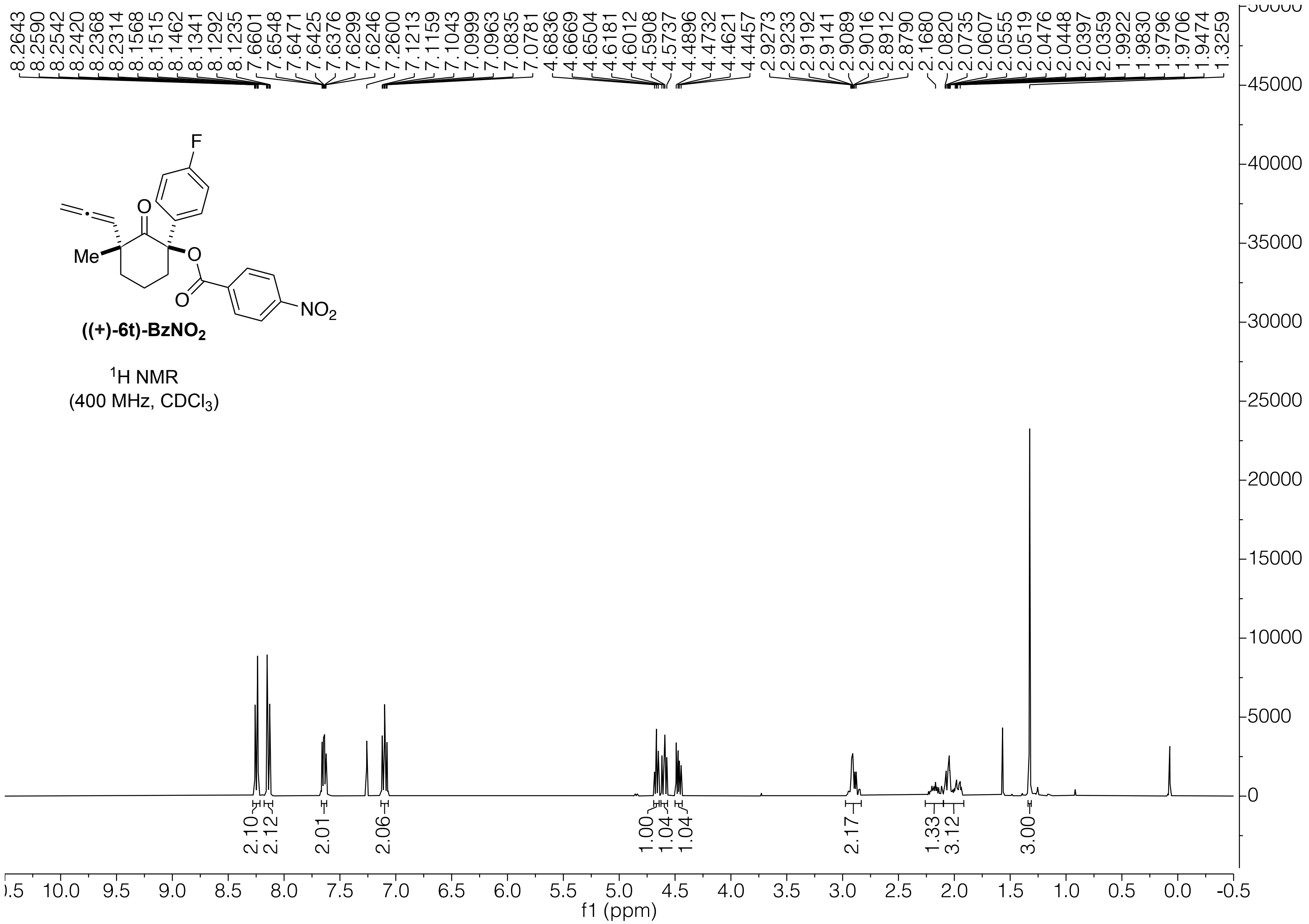

((+)-6t)-BzNO2
^{1}H NMR
(400 MHz, CDCl3)
f1 (ppm)

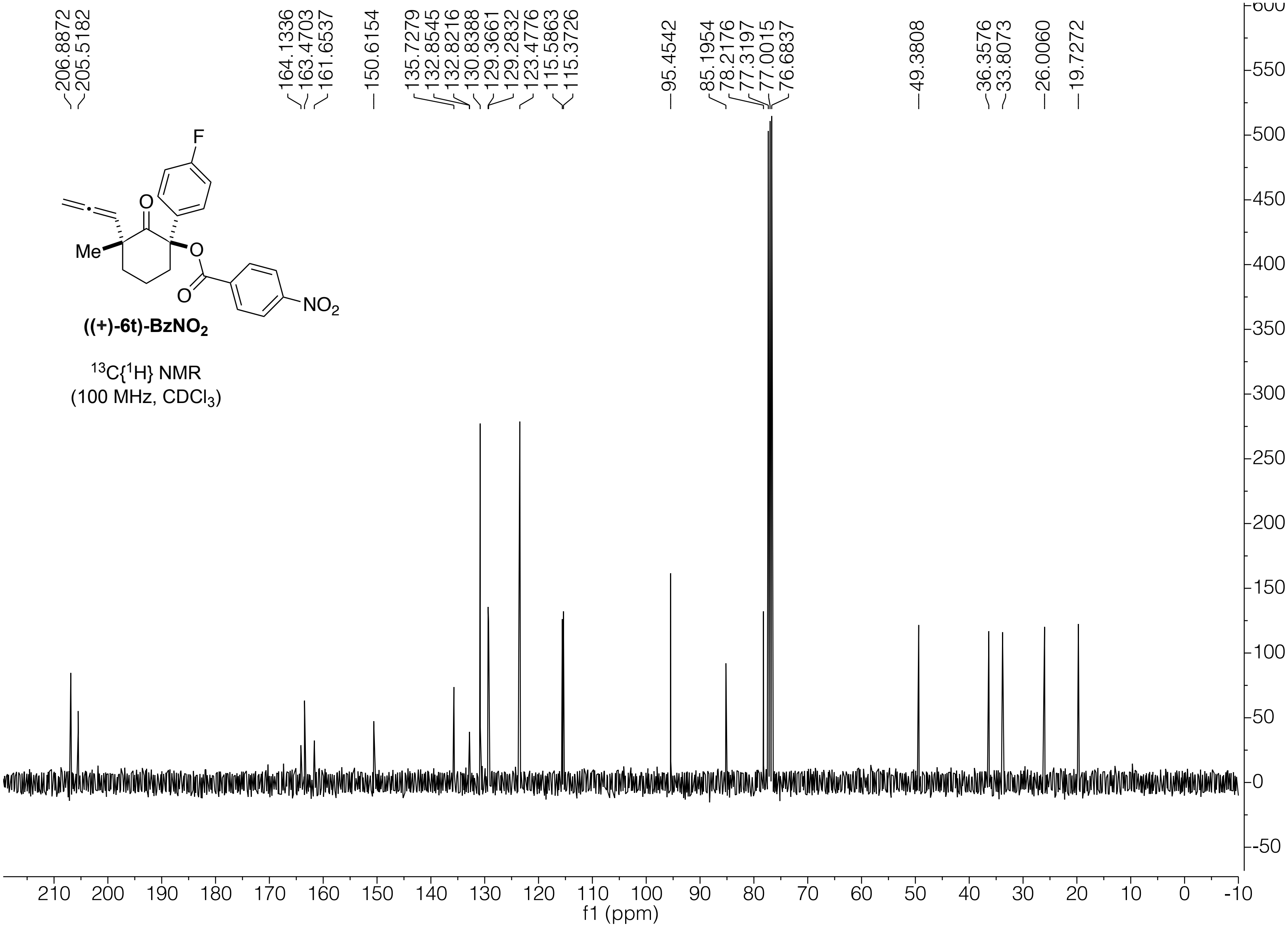

206.8872
205.5182
164.1336
163.4703
161.6537
150.6154
135.7279
132.8545
132.8216
130.8388
129.3661
129.2832
123.4776
115.5863
115.3726
95.4542
85.1954
78.2176
77.3197
77.0015
76.6837
49.3808
36.3576
33.8073
26.0060
19.7272
F
O
Me
O
O
NO2
((+)-6t)-BzNO2
13C{1H} NMR
(100 MHz, CDCl3)
f1 (ppm)

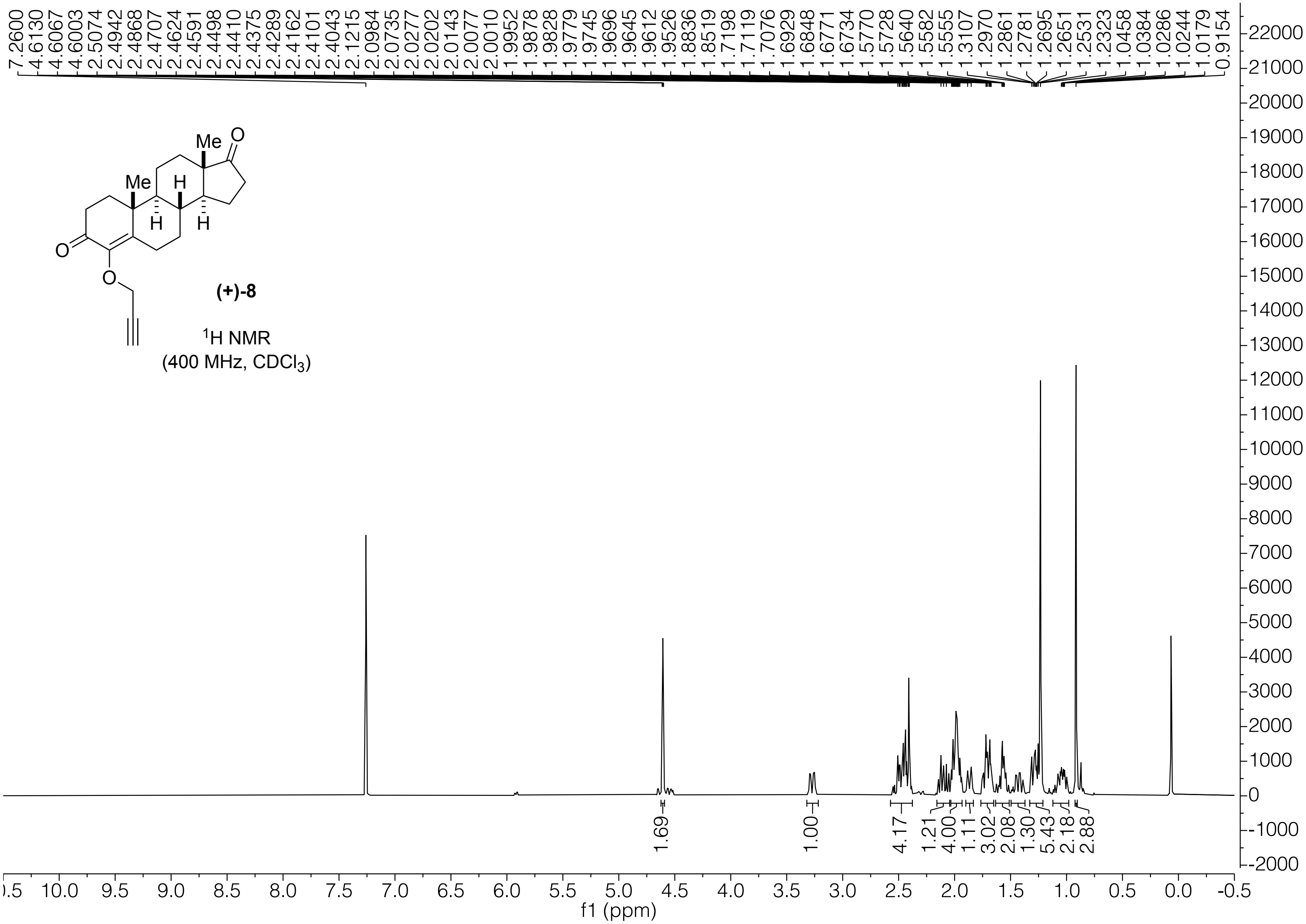

7.2600
4.6130
4.6067
4.6003
2.5074
2.4942
2.4868
2.4707
2.4624
2.4591
2.4498
2.4410
2.4375
2.4289
2.4162
2.4101
2.4043
2.1215
2.0984
2.0735
2.0277
2.0202
2.0143
2.0077
2.0010
1.9952
1.9878
1.9828
1.9779
1.9745
1.9696
1.9645
1.9612
1.9526
1.8836
1.8519
1.7198
1.7119
1.7076
1.6929
1.6848
1.6771
1.6734
1.5770
1.5728
1.5640
1.5582
1.5555
1.3107
1.2970
1.2861
1.2781
1.2695
1.2651
1.2531
1.2323
1.0458
1.0384
1.0286
1.0244
1.0179
0.9154
Me
O
Me
H
H
H
O
O
(+)-8
^{1}H NMR
(400 MHz, $CDCl_3$)
1.69
1.00
4.17
1.21
4.00
1.11
3.02
2.08
1.30
5.43
2.18
2.88
f1 (ppm)

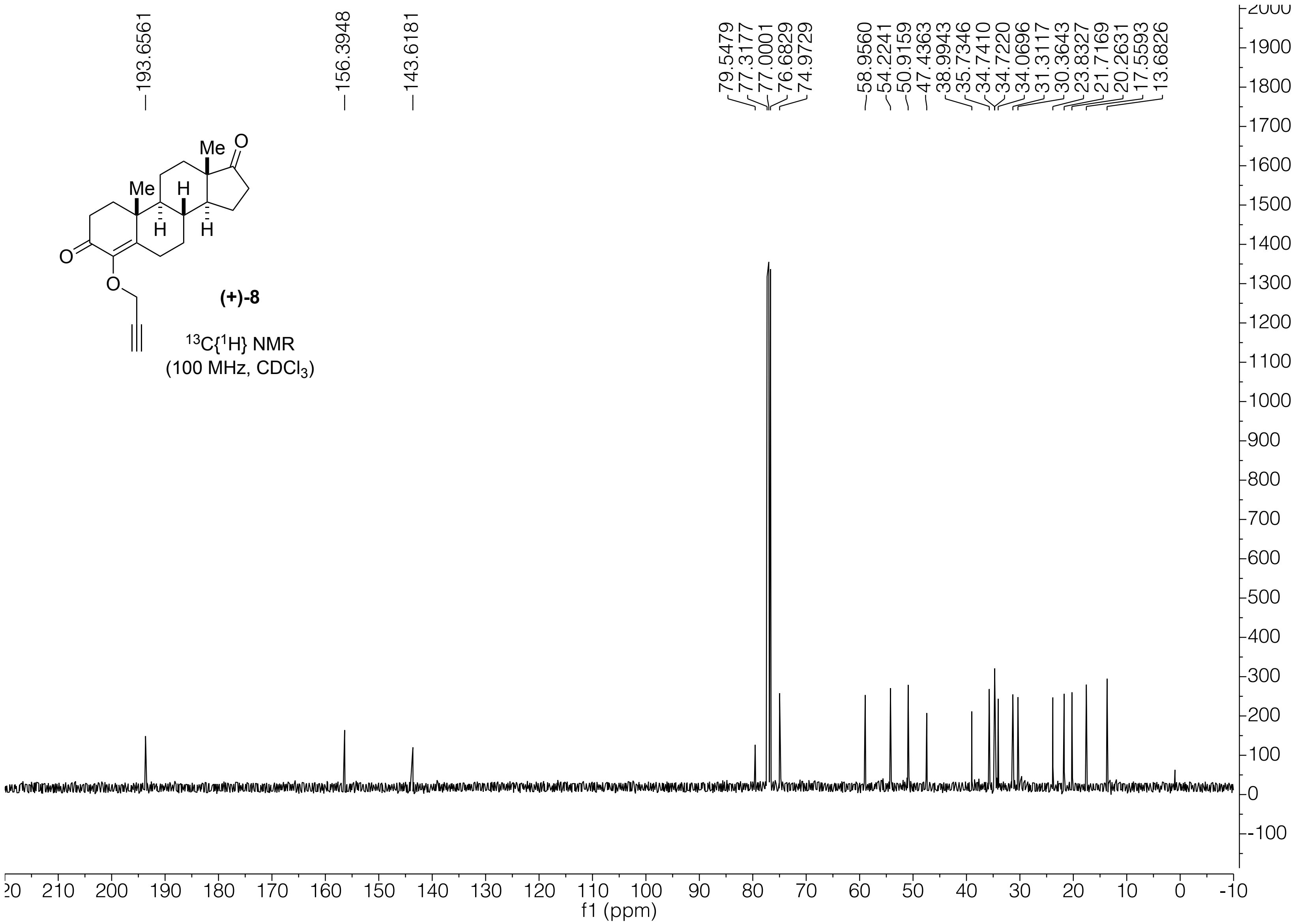

193.6561
156.3948
143.6181
79.5479
77.3177
77.0001
76.6829
74.9729
58.9560
54.2241
50.9159
47.4363
38.9943
35.7346
34.7410
34.7220
34.0696
31.3117
30.3643
23.8327
21.7169
20.2631
17.5593
13.6826
Me
O
Me
H
H
H
O
O
(+)-8
$^{13}C\{^{1}H\}$ NMR
(100 MHz, $CDCl_3$)
f1 (ppm)

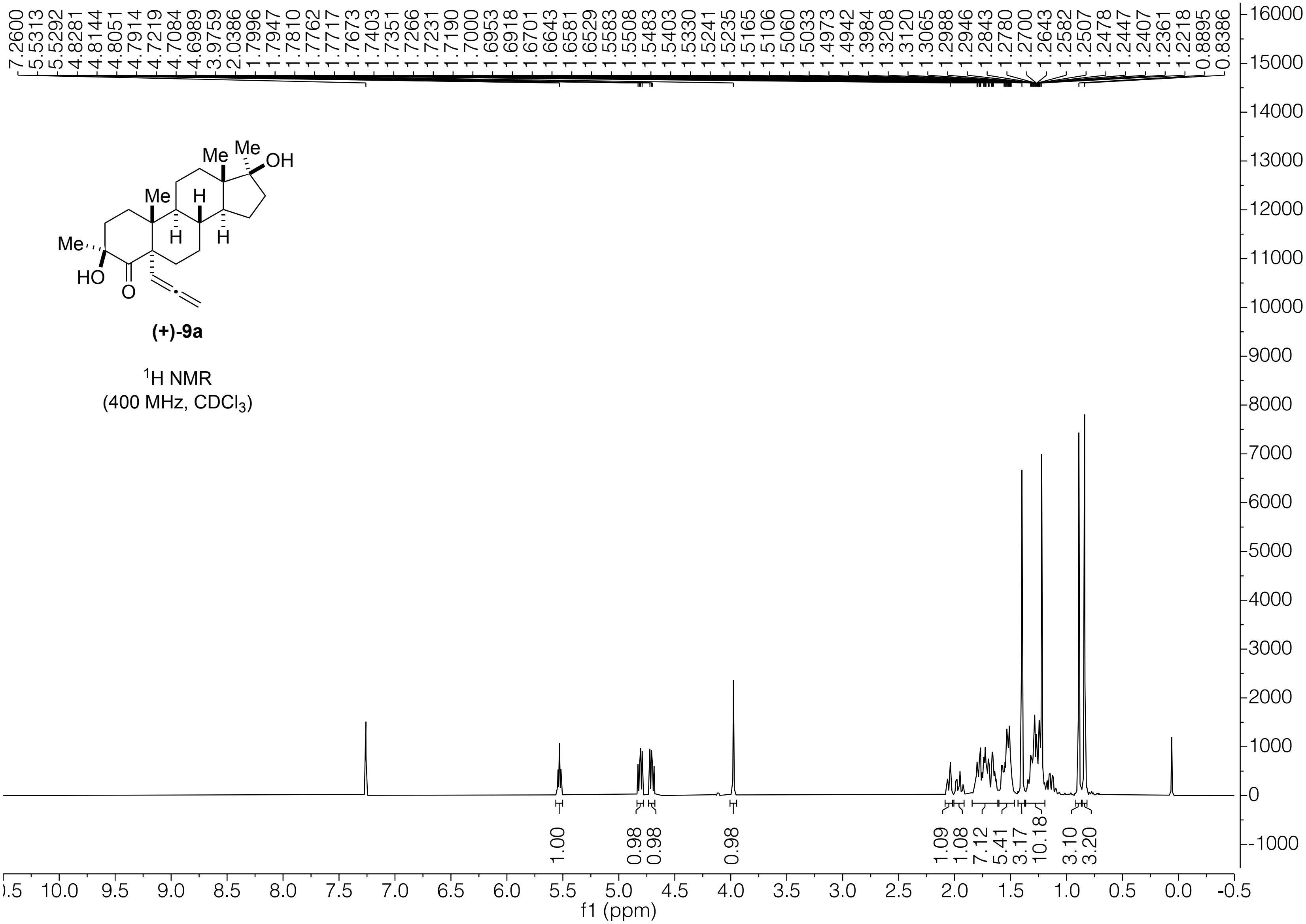
7.2600 5.5313 5.5292 4.8281 4.8144 4.8051 4.7914 4.7219 4.7084 4.6989 3.9759 2.0386 1.7996 1.7947 1.7810 1.7762 1.7717 1.7673 1.7403 1.7351 1.7266 1.7231 1.7190 1.7000 1.6953 1.6918 1.6701 1.6643 1.6581 1.6529 1.5583 1.5508 1.5483 1.5403 1.5330 1.5241 1.5235 1.5165 1.5106 1.5060 1.5033 1.4973 1.4942 1.3984 1.3208 1.3120 1.3065 1.2988 1.2946 1.2843 1.2780 1.2700 1.2643 1.2582 1.2507 1.2478 1.2447 1.2407 1.2361 1.2218 0.8895 0.8386
Me
Me
OH
Me
H
H
H
Me
HO
O
(+)-9a
1H NMR
(400 MHz, CDCl3)
1.00
0.98
0.98
0.98
1.09
1.08
7.12
5.41
3.17
10.18
3.10
3.20
f1 (ppm)

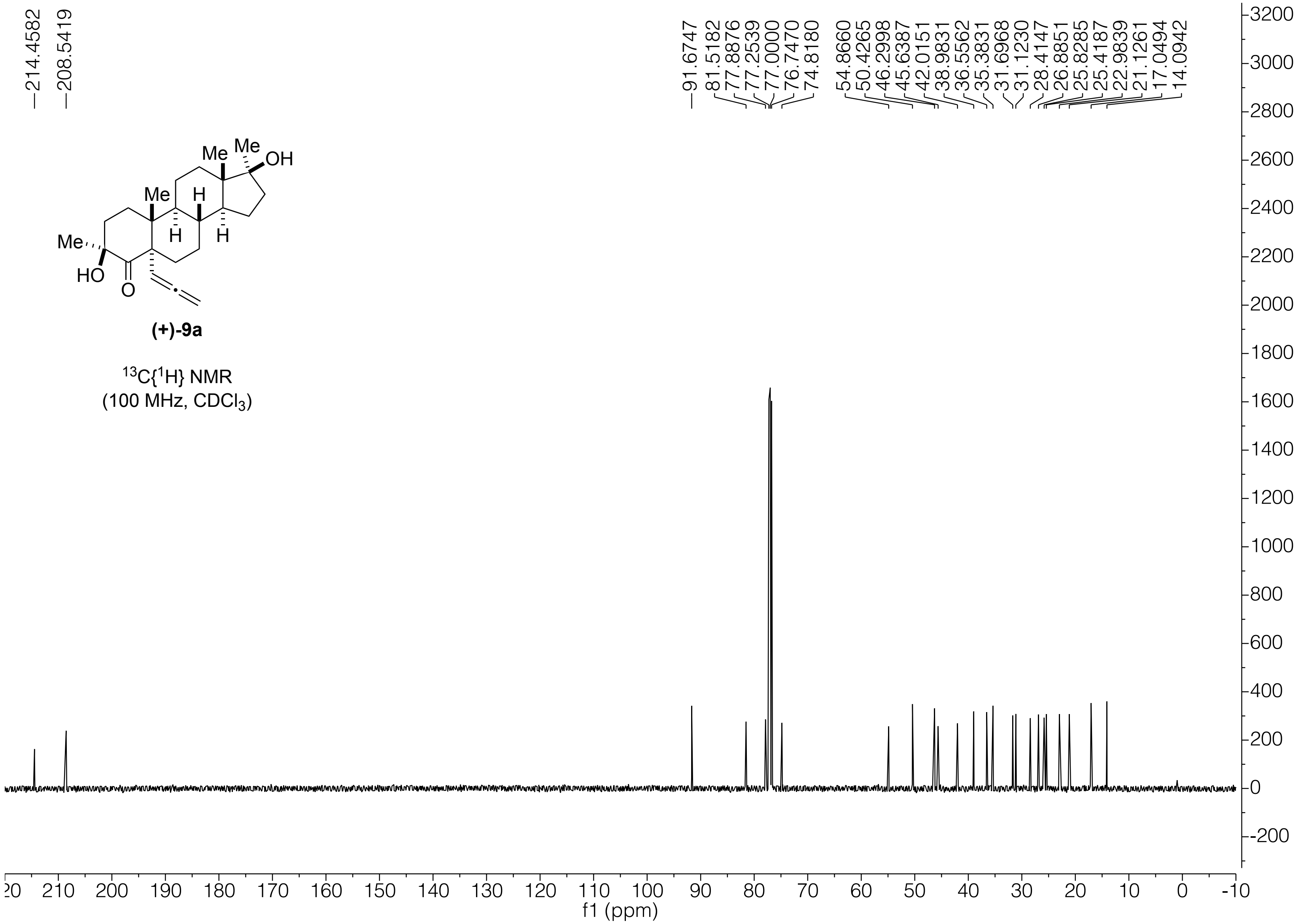
214.4582
208.5419
91.6747
81.5182
77.8876
77.2539
77.0000
76.7470
74.8180
54.8660
50.4265
46.2998
45.6387
42.0151
38.9831
36.5562
35.3831
31.6968
31.1230
28.4147
26.8851
25.8285
25.4187
22.9839
21.1261
17.0494
14.0942
(+)-9a
$^{13}C\{^{1}H\}$ NMR
(100 MHz, $CDCl_3$)
f1 (ppm)

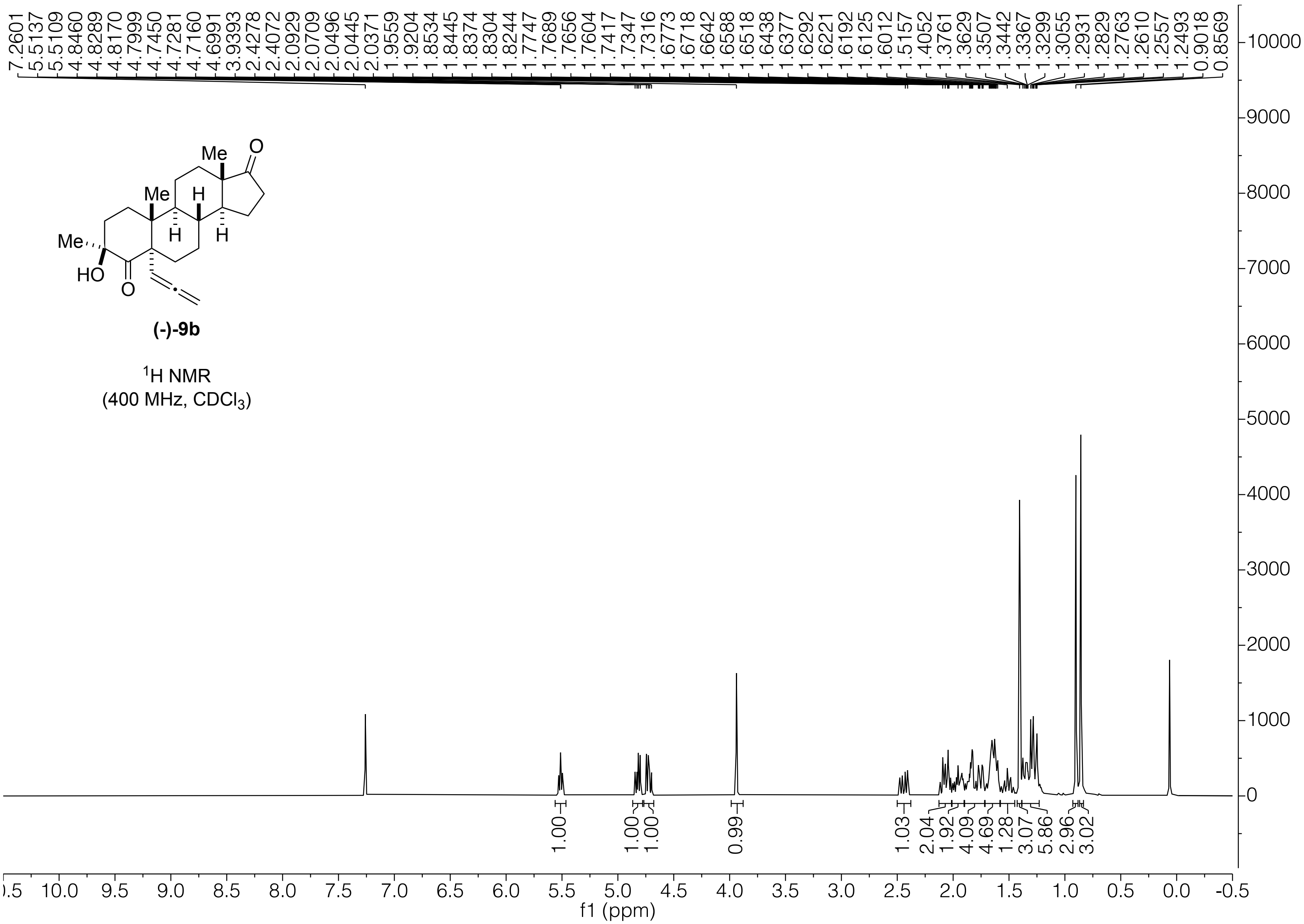

7.2601
5.5137
5.5109
4.8460
4.8289
4.8170
4.7999
4.7450
4.7281
4.7160
4.6991
3.9393
2.4278
2.4072
2.0929
2.0709
2.0496
2.0445
2.0371
1.9559
1.9204
1.8534
1.8445
1.8374
1.8304
1.8244
1.7747
1.7689
1.7656
1.7604
1.7417
1.7347
1.7316
1.6773
1.6718
1.6642
1.6588
1.6518
1.6438
1.6377
1.6292
1.6221
1.6192
1.6125
1.6012
1.5157
1.4052
1.3761
1.3629
1.3507
1.3442
1.3367
1.3299
1.3055
1.2931
1.2829
1.2763
1.2610
1.2557
1.2493
0.9018
0.8569
Me
O
Me
H
Me
H
H
HO
O
(-)-9b
1H NMR
(400 MHz, CDCl3)
1.00
1.00
1.00
0.99
1.03
2.04
1.92
4.09
4.69
1.28
3.07
5.86
2.96
3.02
10000
9000
8000
7000
6000
5000
4000
3000
2000
1000
0
10.0
9.5
9.0
8.5
8.0
7.5
7.0
6.5
6.0
5.5
5.0
4.5
4.0
3.5
3.0
2.5
2.0
1.5
1.0
0.5
0.0
-0.5
f1 (ppm)

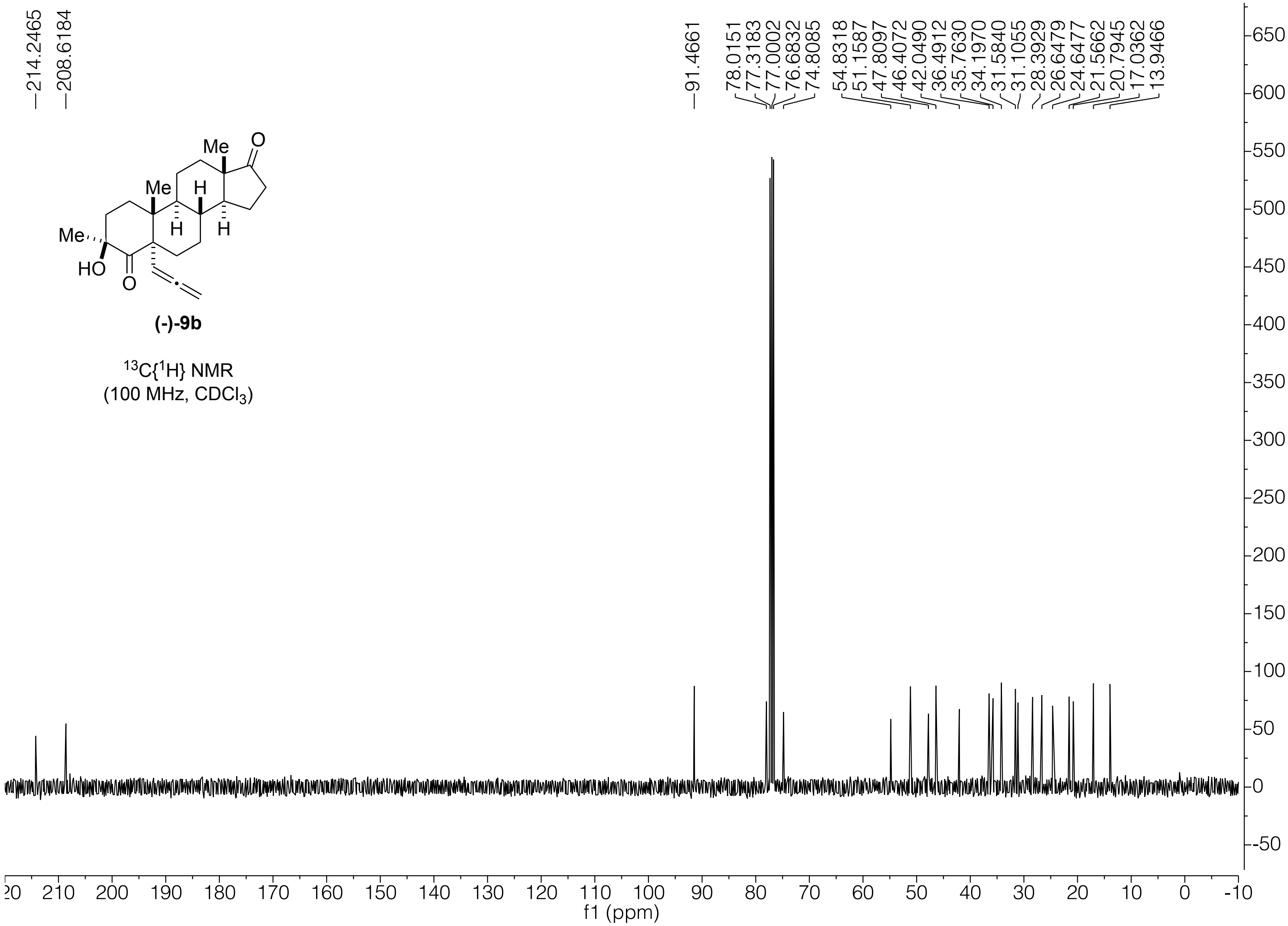

214.2465
208.6184
91.4661
78.0151
77.3183
77.0002
76.6832
74.8085
54.8318
51.1587
47.8097
46.4072
42.0490
36.4912
35.7630
34.1970
31.5840
31.1055
28.3929
26.6479
24.6477
21.5662
20.7945
17.0362
13.9466
Me
O
Me
H
Me
HO
O
H
H
(-)-9b
$^{13}C\{^{1}H\}$ NMR
(100 MHz, $CDCl_3$)
f1 (ppm)

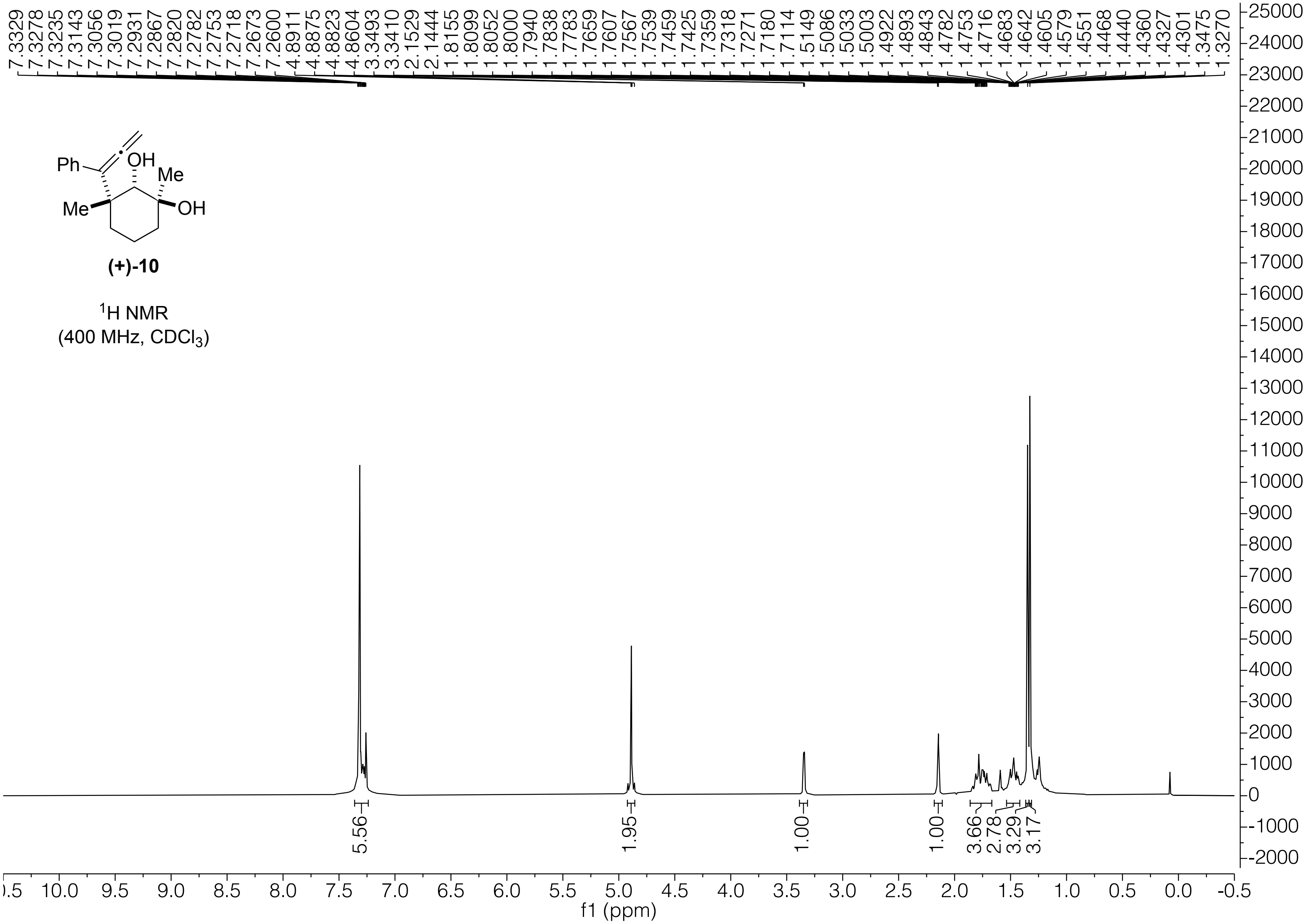

(+)-10
^{1}H NMR
(400 MHz, $CDCl_3$)
f1 (ppm)
5.56
1.95
1.00
1.00
3.66
2.78
3.29
3.17

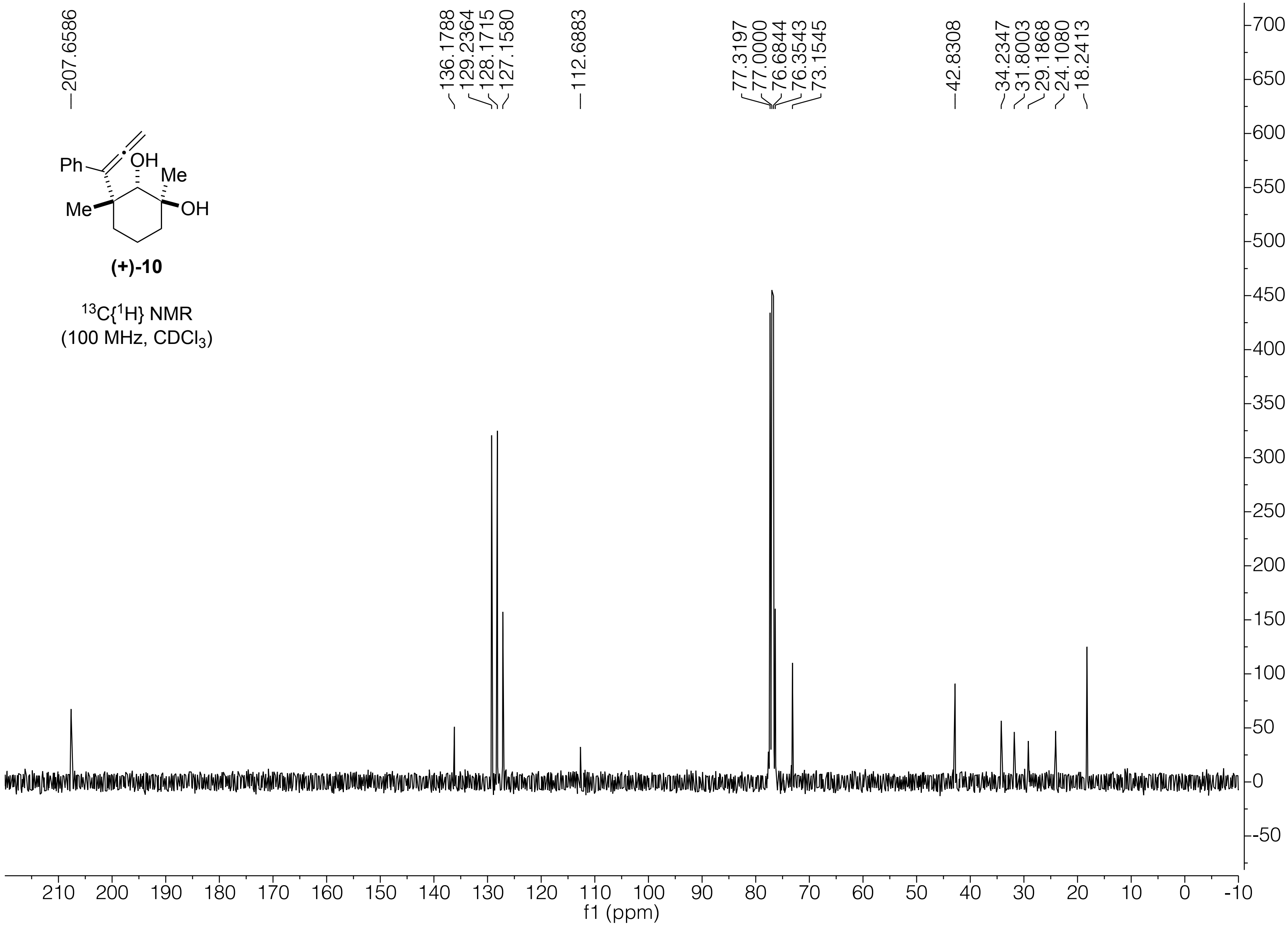
207.6586
136.1788
129.2364
128.1715
127.1580
112.6883
77.3197
77.0000
76.6844
76.3543
73.1545
42.8308
34.2347
31.8003
29.1868
24.1080
18.2413
Ph
OH
Me
Me
OH
(+)-10
13C{1H} NMR
(100 MHz, CDCl3)
f1 (ppm)

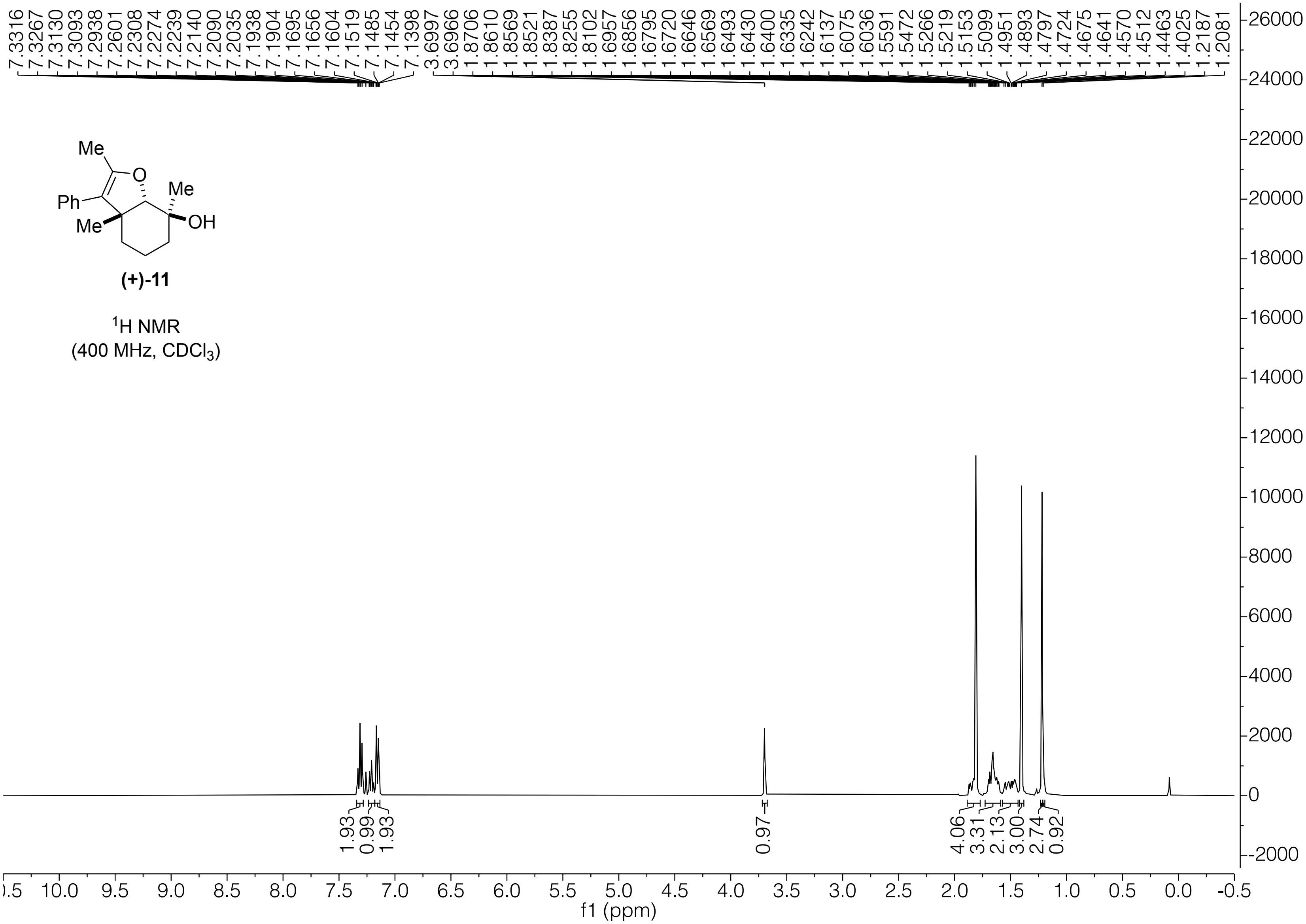

(+)-11

^{1}H NMR
(400 MHz, $CDCl_3$)

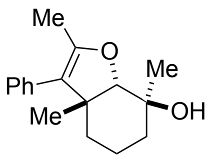

(+)-11

$^{13}C\{^1H\}$ NMR
(100 MHz, $CDCl_3$)

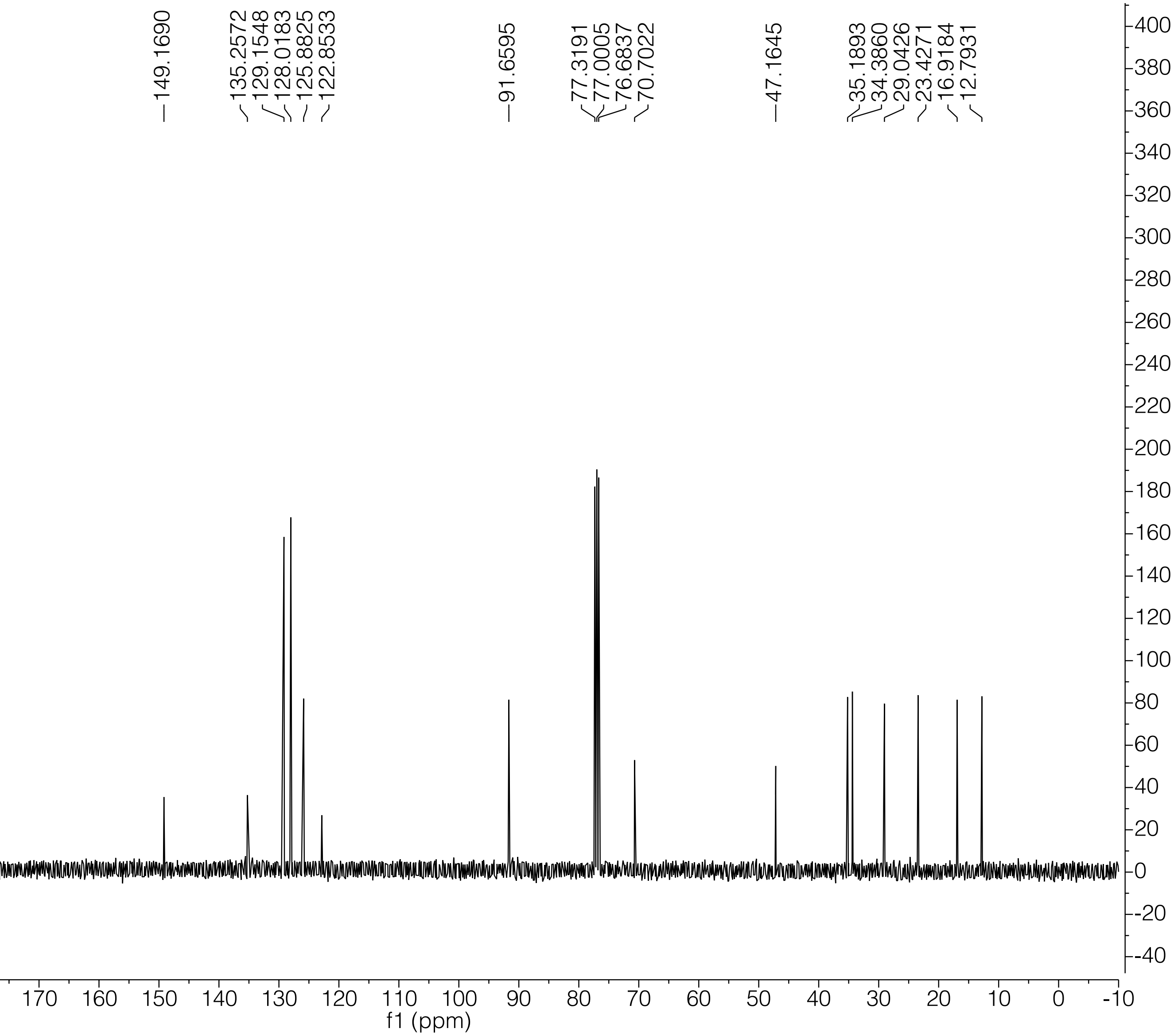

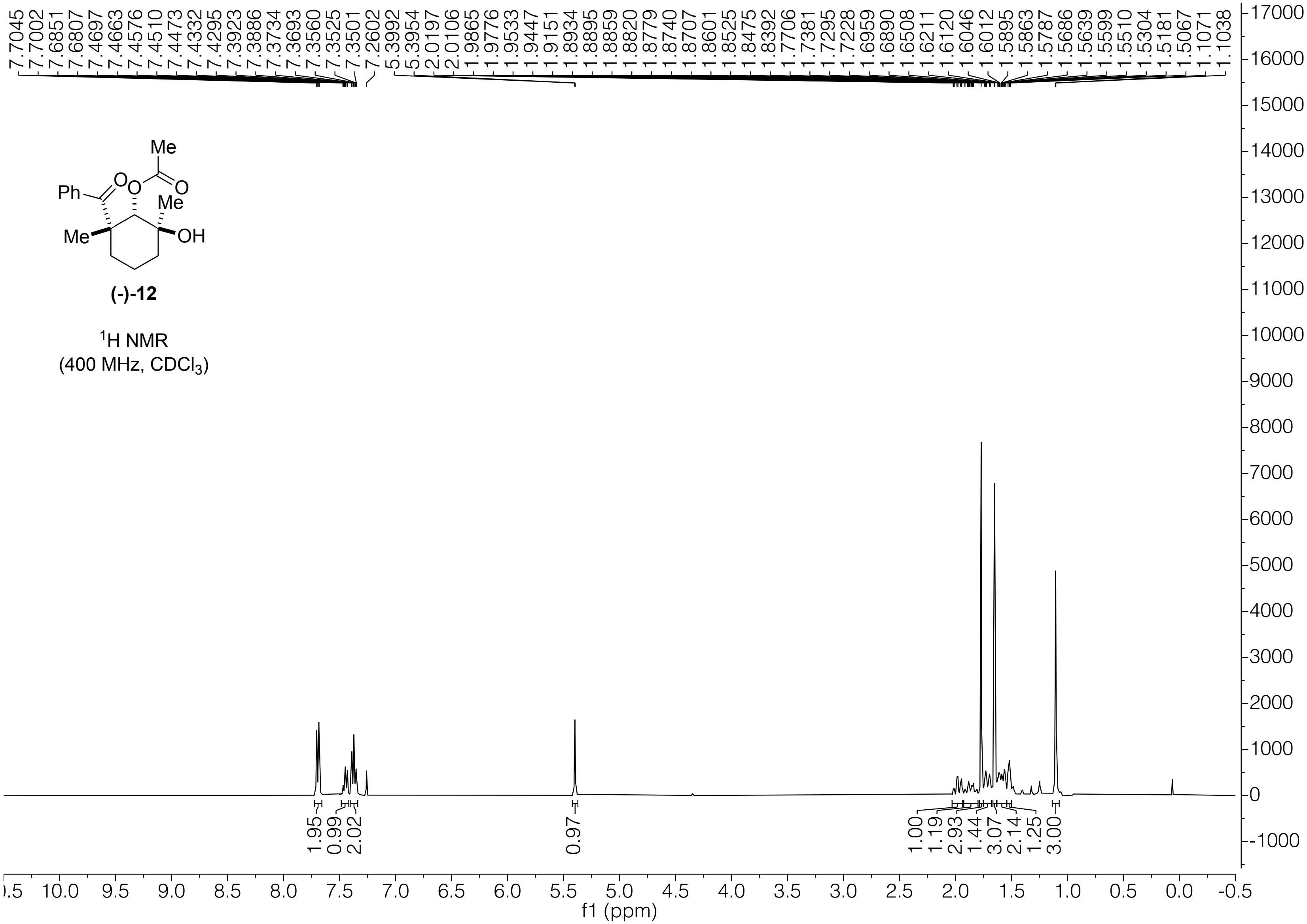

7.7045
7.7002
7.6851
7.6807
7.4697
7.4663
7.4576
7.4510
7.4473
7.4332
7.4295
7.3923
7.3886
7.3734
7.3693
7.3560
7.3525
7.3501
7.2602
5.3992
5.3954
2.0197
2.0106
1.9865
1.9776
1.9533
1.9447
1.9151
1.8934
1.8895
1.8859
1.8820
1.8779
1.8740
1.8707
1.8601
1.8525
1.8475
1.8392
1.7706
1.7381
1.7295
1.7228
1.6959
1.6890
1.6508
1.6211
1.6120
1.6046
1.6012
1.5895
1.5863
1.5787
1.5686
1.5639
1.5599
1.5510
1.5304
1.5181
1.5067
1.1071
1.1038
Me
Ph
O
O
O
Me
Me
OH
(-)-12
^{1}H NMR
(400 MHz, $CDCl_3$)
1.95
0.99
2.02
0.97
1.00
1.19
2.93
1.44
3.07
2.14
1.25
3.00
f1 (ppm)

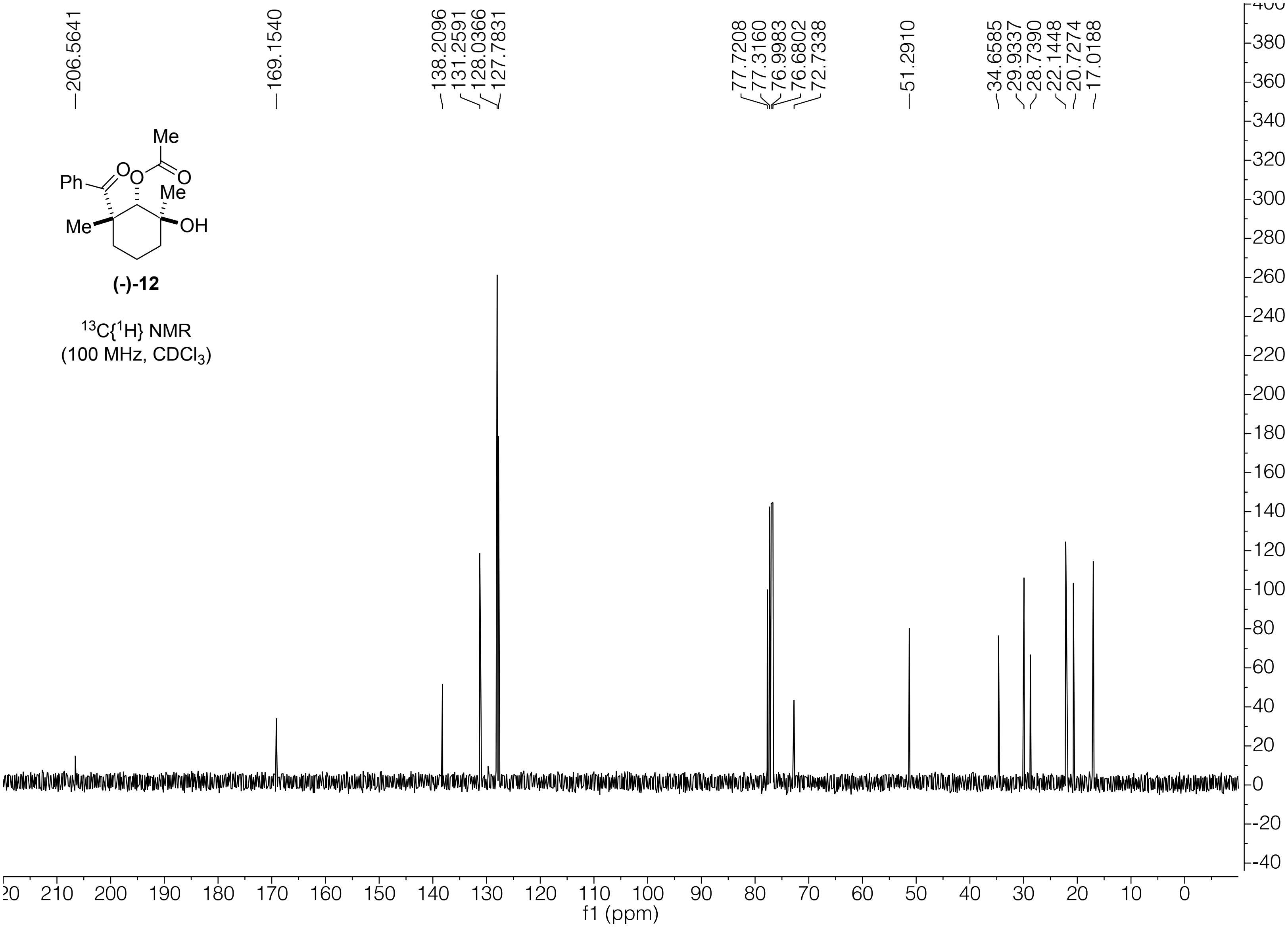

206.5641
169.1540
138.2096
131.2591
128.0366
127.7831
77.7208
77.3160
76.9983
76.6802
72.7338
51.2910
34.6585
29.9337
28.7390
22.1448
20.7274
17.0188
Me
O
Ph
O
O
Me
Me
OH
(-)-12
^{13}C{^{1}H} NMR
(100 MHz, $CDCl_3$)
f1 (ppm)

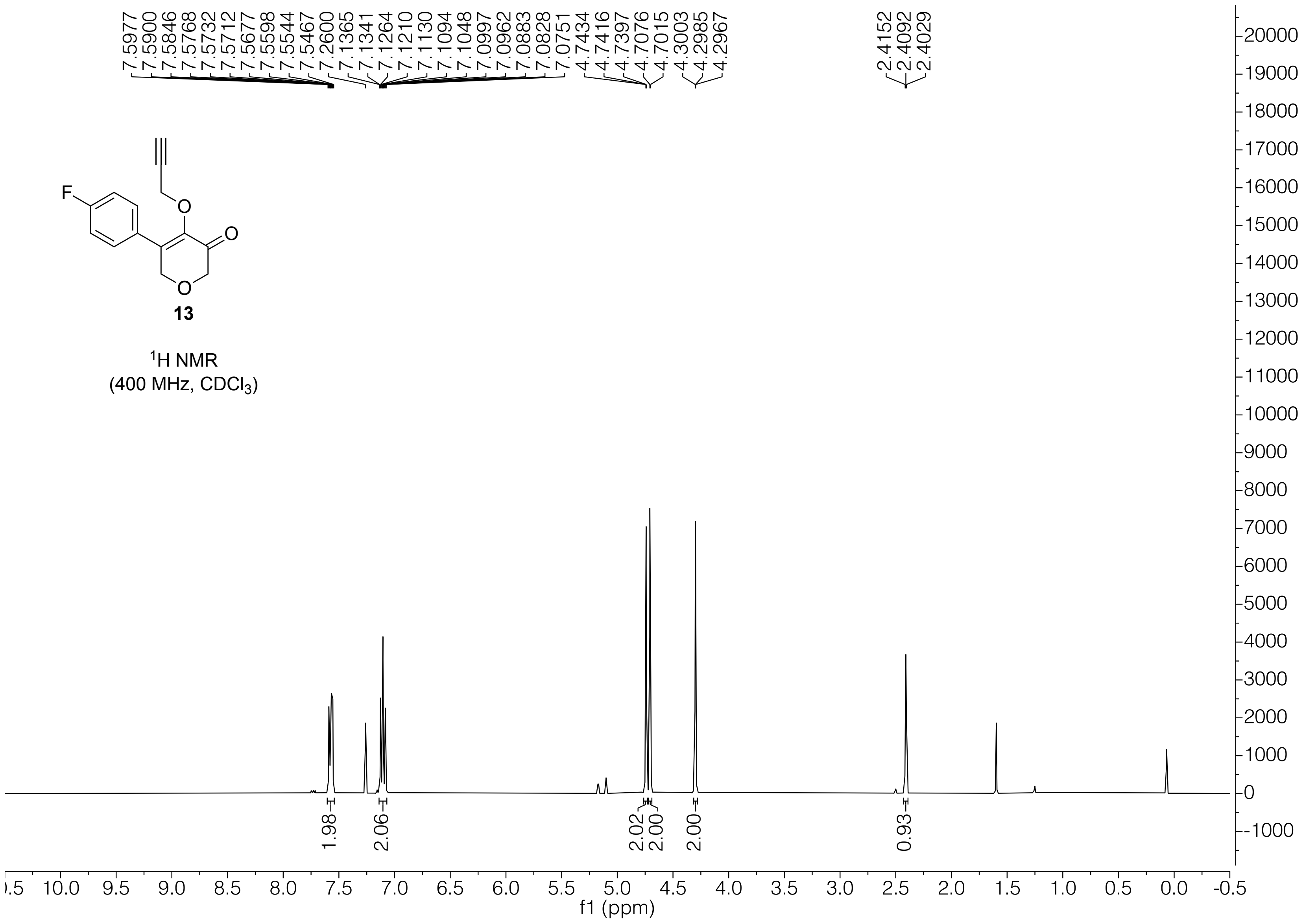

7.5977 7.5900 7.5846 7.5768 7.5732 7.5712 7.5677 7.5598 7.5544 7.5467 7.2600 7.1365 7.1341 7.1264 7.1210 7.1130 7.1094 7.1048 7.0997 7.0962 7.0883 7.0828 7.0751 4.7434 4.7416 4.7397 4.7076 4.7015 4.3003 4.2985 4.2967
2.4152 2.4092 2.4029
F
O
O
O
13
1H NMR
(400 MHz, CDCl3)
1.98 2.06 2.02 2.00 2.00 0.93
f1 (ppm)

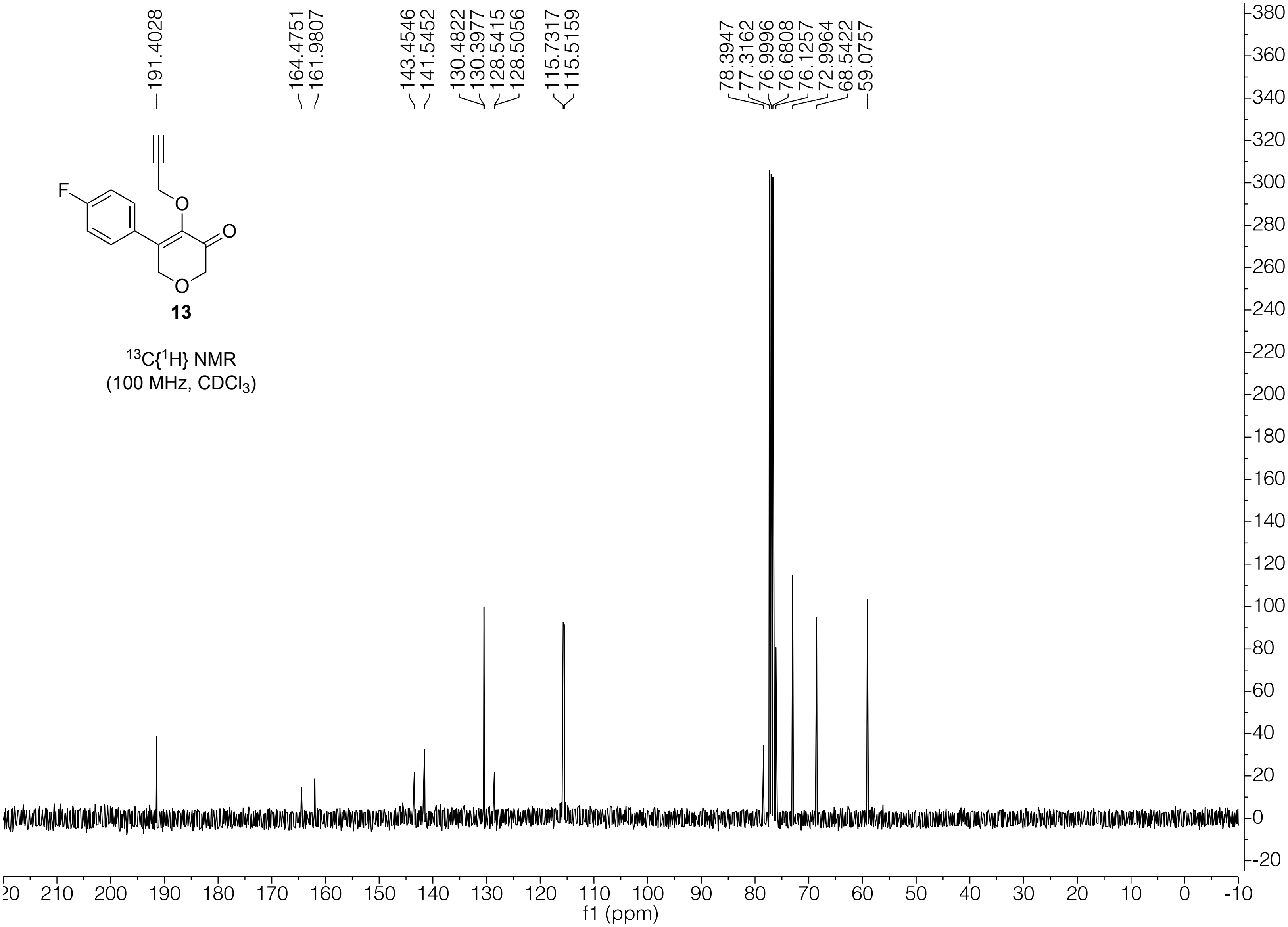
191.4028
164.4751
161.9807
143.4546
141.5452
130.4822
130.3977
128.5415
128.5056
115.7317
115.5159
78.3947
77.3162
76.9996
76.6808
76.1257
72.9964
68.5422
59.0757
F
O
O
O
13
$^{13}C\{^1H\}$ NMR
(100 MHz, $CDCl_3$)
f1 (ppm)

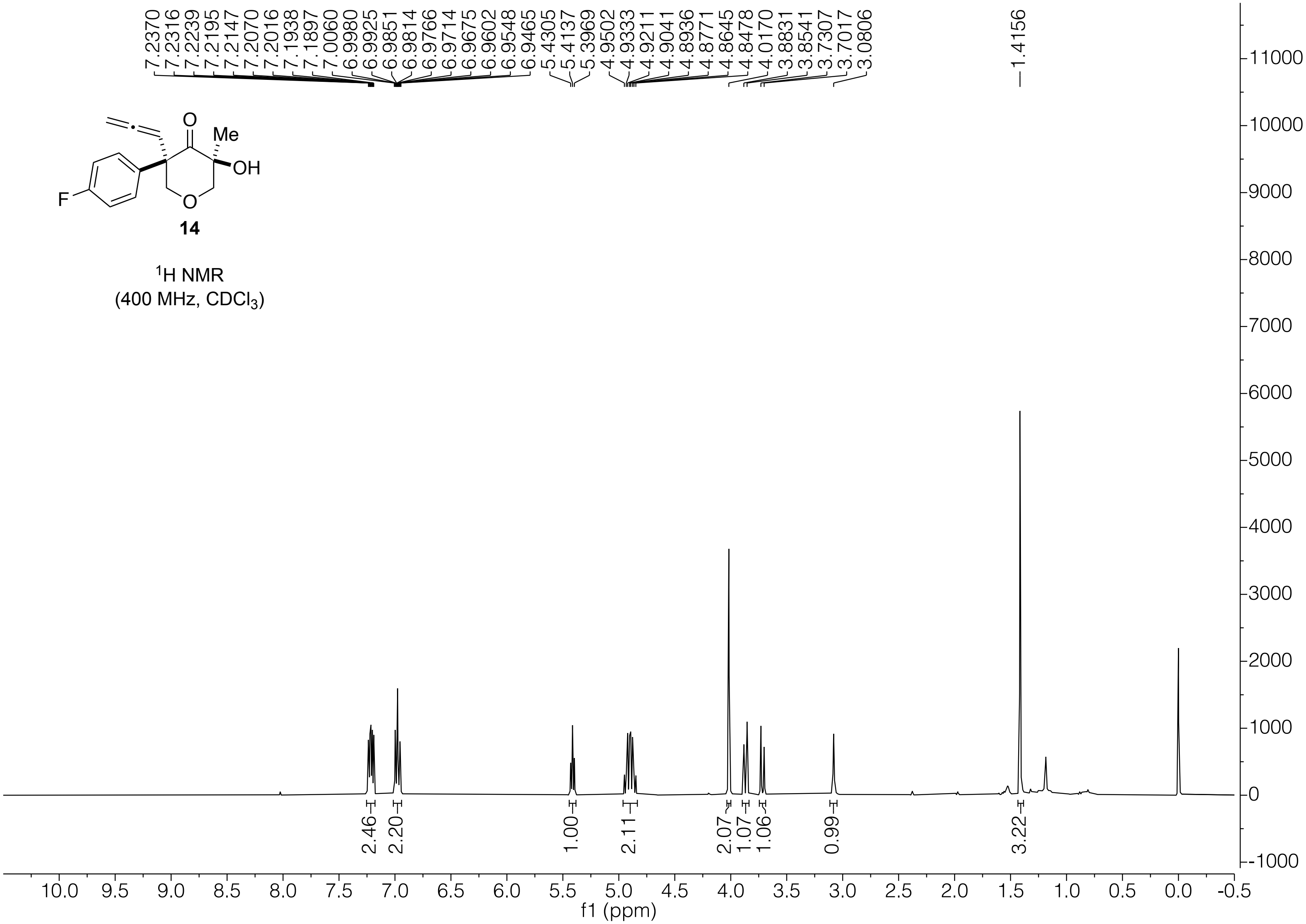
7.2370
7.2316
7.2239
7.2195
7.2147
7.2070
7.2016
7.1938
7.1897
7.0060
6.9980
6.9925
6.9851
6.9814
6.9766
6.9714
6.9675
6.9602
6.9548
6.9465
5.4305
5.4137
5.3969
4.9502
4.9333
4.9211
4.9041
4.8936
4.8771
4.8645
4.8478
4.0170
3.8831
3.8541
3.7307
3.7017
3.0806
1.4156
O
Me
OH
F
O
14
1H NMR
(400 MHz, CDCl3)
2.46
2.20
1.00
2.11
2.07
1.07
1.06
0.99
3.22
f1 (ppm)

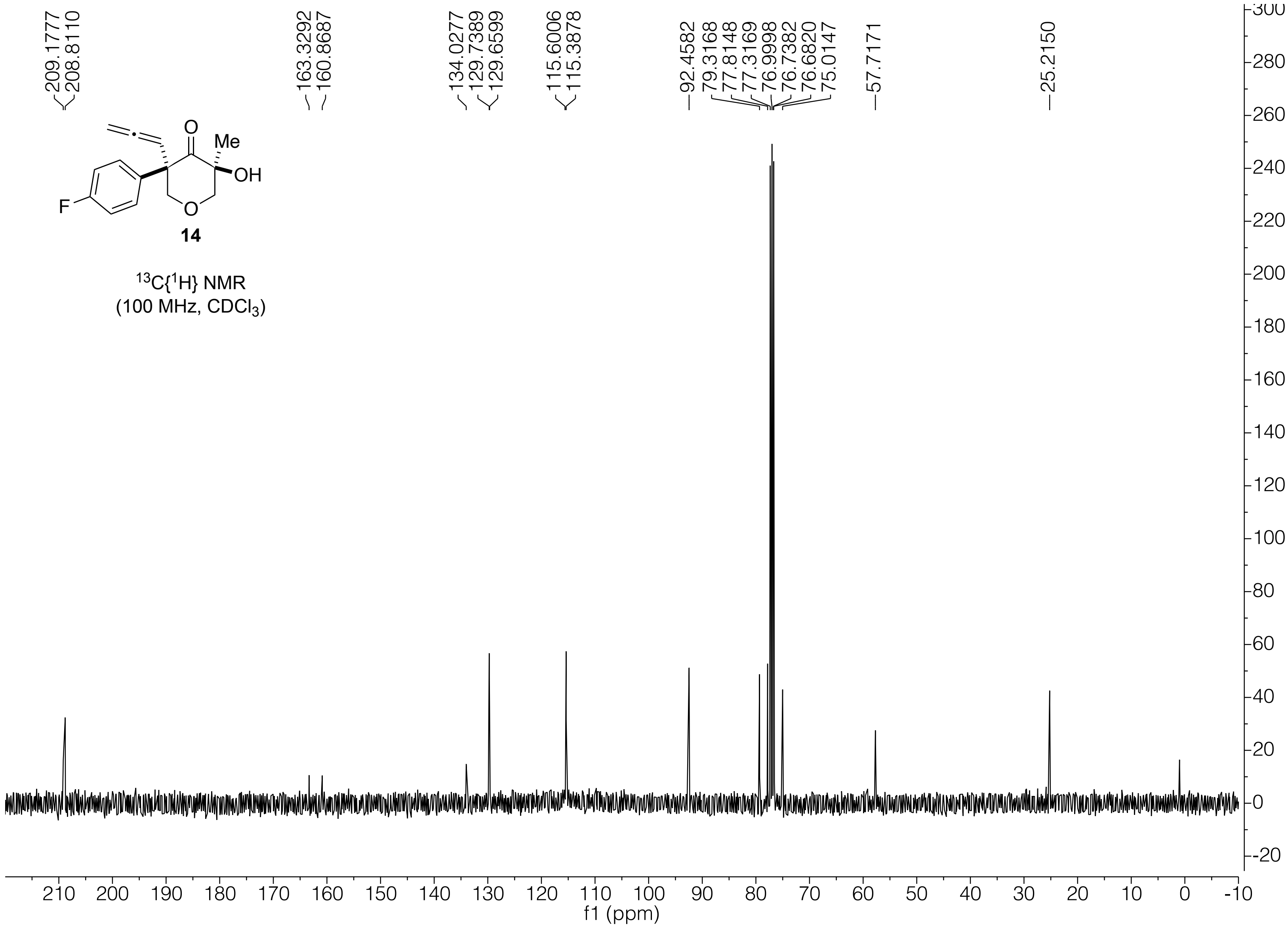

209.1777
208.8110
163.3292
160.8687
134.0277
129.7389
129.6599
115.6006
115.3878
92.4582
79.3168
77.8148
77.3169
76.9998
76.7382
76.6820
75.0147
57.7171
25.2150
O
Me
OH
F
O
14
$^{13}C\{^{1}H\}$ NMR
(100 MHz, $CDCl_3$)
f1 (ppm)

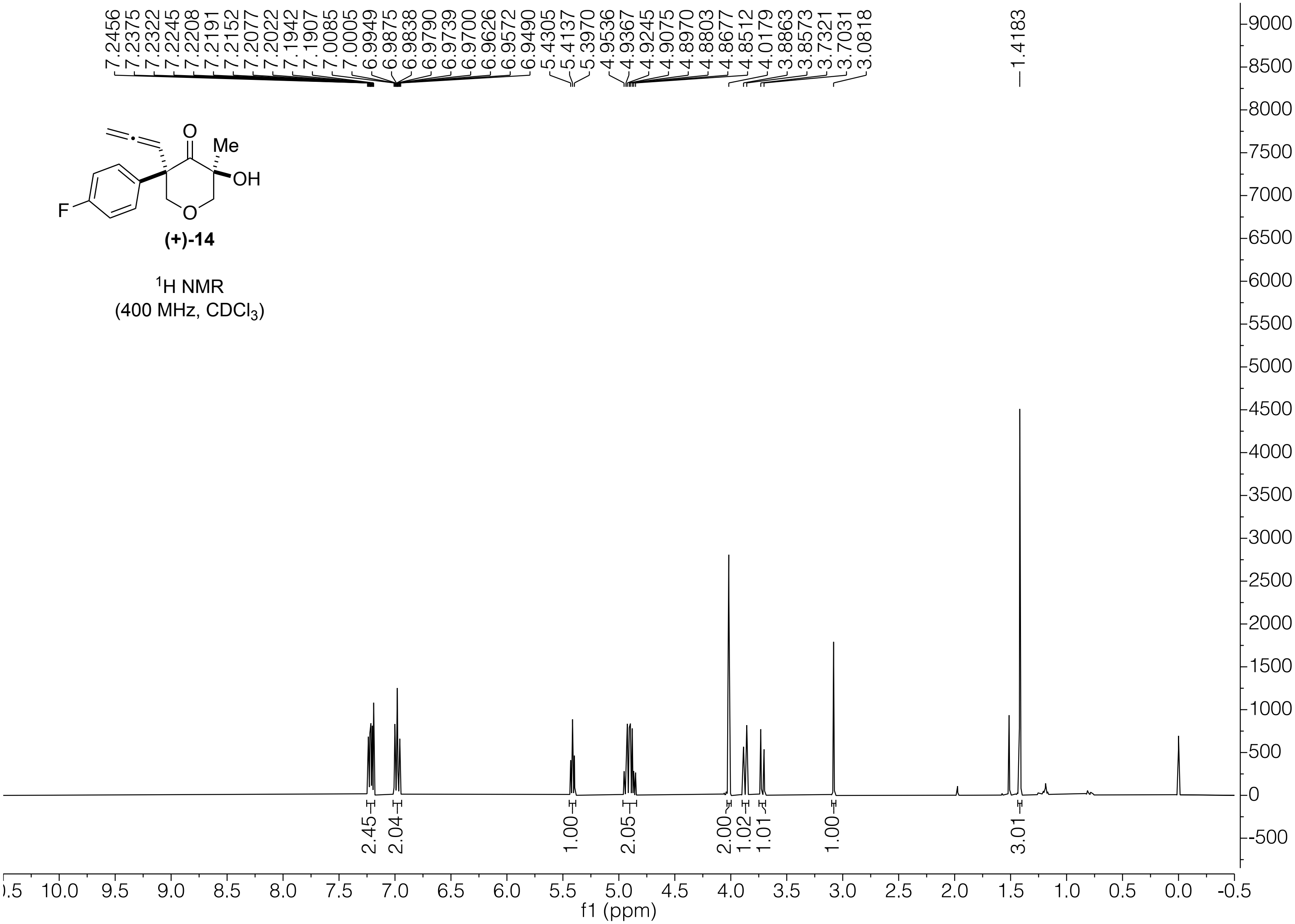

7.2456
7.2375
7.2322
7.2245
7.2208
7.2191
7.2152
7.2077
7.2022
7.1942
7.1907
7.0085
7.0005
6.9949
6.9875
6.9838
6.9790
6.9739
6.9700
6.9626
6.9572
6.9490
5.4305
5.4137
5.3970
4.9536
4.9367
4.9245
4.9075
4.8970
4.8803
4.8677
4.8512
4.0179
3.8863
3.8573
3.7321
3.7031
3.0818
1.4183
O
Me
OH
F
O
(+)-14
^{1}H NMR
(400 MHz, $CDCl_3$)
2.45
2.04
1.00
2.05
2.00
1.02
1.01
1.00
3.01
f1 (ppm)

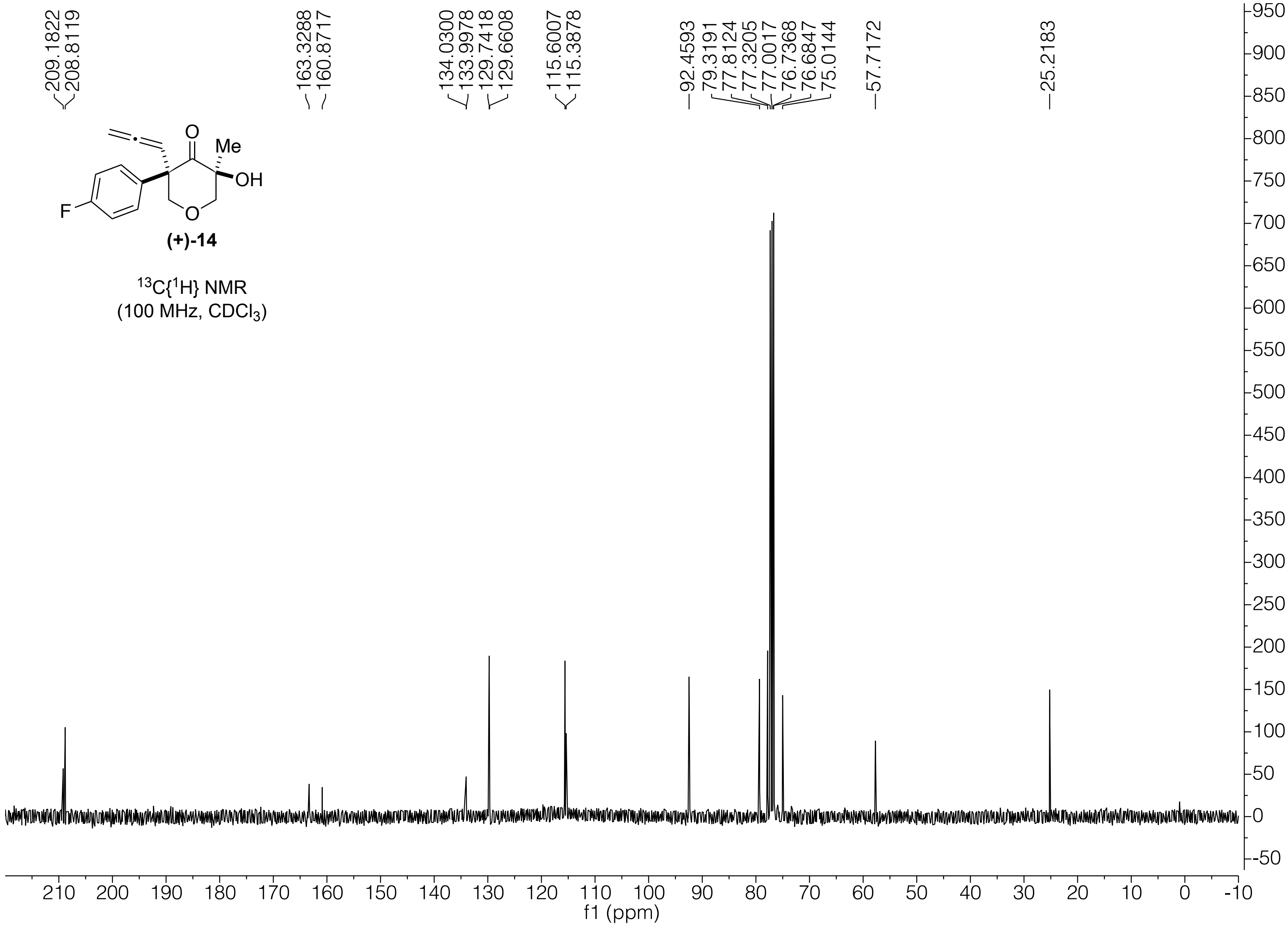
209.1822
208.8119
163.3288
160.8717
134.0300
133.9978
129.7418
129.6608
115.6007
115.3878
92.4593
79.3191
77.8124
77.3205
77.0017
76.7368
76.6847
75.0144
57.7172
25.2183
Me
OH
F
O
(+)-14
$^{13}C\{^{1}H\}$ NMR
(100 MHz, $CDCl_3$)
f1 (ppm)

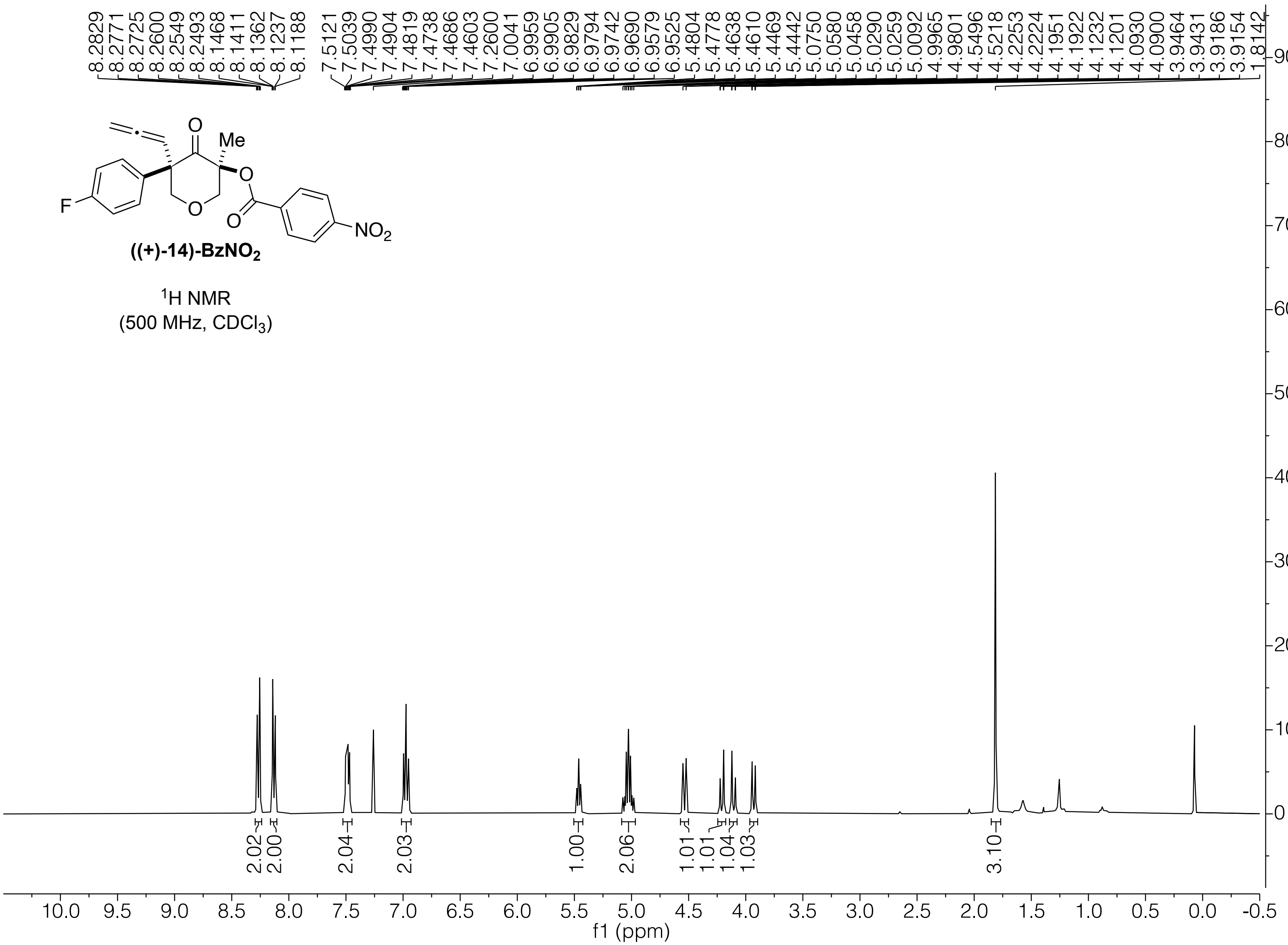
8.2829
8.2771
8.2725
8.2600
8.2549
8.2493
8.1468
8.1411
8.1362
8.1237
8.1188
7.5121
7.5039
7.4990
7.4904
7.4819
7.4738
7.4686
7.4603
7.2600
7.0041
6.9959
6.9905
6.9829
6.9794
6.9742
6.9690
6.9579
6.9525
5.4804
5.4778
5.4638
5.4610
5.4469
5.4442
5.0750
5.0580
5.0458
5.0290
5.0259
5.0092
4.9965
4.9801
4.5496
4.5218
4.2253
4.2224
4.1951
4.1922
4.1232
4.1201
4.0930
4.0900
3.9464
3.9431
3.9186
3.9154
1.8142
O
Me
O
F
O
O
NO_2
((+)-14)-BzNO$_2$
^{1}H NMR
(500 MHz, $CDCl_3$)
2.02
2.00
2.04
2.03
1.00
2.06
1.01
1.01
1.04
1.03
3.10
f1 (ppm)

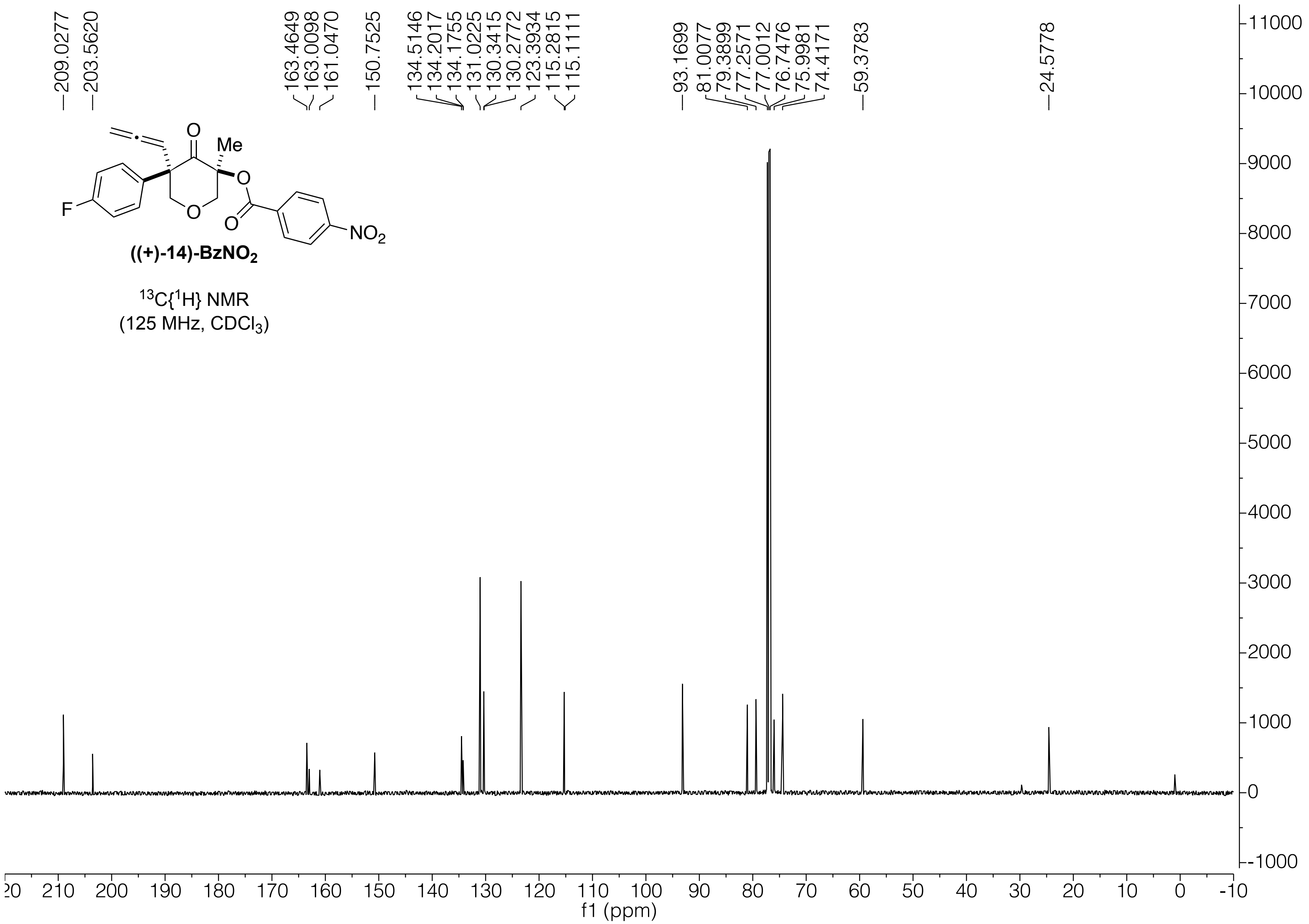
209.0277
203.5620
163.4649
163.0098
161.0470
150.7525
134.5146
134.2017
134.1755
131.0225
130.3415
130.2772
123.3934
115.2815
115.1111
93.1699
81.0077
79.3899
77.2571
77.0012
76.7476
75.9981
74.4171
59.3783
24.5778
Me
O
F
NO2
((+)-14)-BzNO2
13C{1H} NMR
(125 MHz, CDCl3)
f1 (ppm)
